# Composition and functional profiles of gut microbiota reflect the treatment stage, severity, and etiology of acute pancreatitis

**DOI:** 10.1128/spectrum.00829-23

**Published:** 2023-09-12

**Authors:** Zhenjiang Wang, Mingyi Guo, Jing Li, Chuangming Jiang, Sen Yang, Shizhuo Zheng, Mingzhe Li, Xinbo Ai, Xiaohong Xu, Wenbo Zhang, Xingxiang He, Yinan Wang, Yuping Chen

**Affiliations:** 1 Department of Gastroenterology, Zhuhai Hospital Affiliated with Jinan University (Zhuhai People’s Hospital), Zhuhai, China; 2 School of Management, University of Science and Technology of China, Hefei, Anhui, China; 3 Department of Research and Development, Shenzhen Byoryn Technology Co., Ltd., Shenzhen, China; 4 Department of Gastroenterology, Gaolangang Branch of Zhuhai People’s Hospital (Hospital of Gaolangang), Zhuhai, China; 5 College of Medicine and Biological Information Engineering, Northeastern University, Shenyang, China; 6 Department of Gastroenterology, The First Affiliated Hospital of Jinan University, Guangzhou, China; 7 Department of Obstetrics and Gynecology, Peking University Shenzhen Hospital, Shenzhen, China; Institut National de Santé Publique du Québec, Sainte-Anne-de-Bellevue, Québec, Canada

**Keywords:** acute pancreatitis, gut microbiota, metagenomic sequencing, intestinal metabolism

## Abstract

Acute pancreatitis (AP) is a type of digestive system disease with high mortality. Previous studies have shown that gut microbiota can participate in developing and treating acute pancreatitis by affecting the host’s metabolism. In this study, we followed 20 AP patients to generate longitudinal gut microbiota profiles and activity during disease (before treatment, on the third day of treatment, and 1 month after discharge). We analyzed species composition and metabolic pathways’ changes across the treatment phase, severity, and etiology. The diversity of the gut microbiome of patients with AP did not show much variation with treatment. In contrast, the metabolic functions of the gut microbiota, such as the essential chemical reactions that produce energy and maintain life, were partially reinstated after treatment. The severe AP (SAP) patients contained less beneficial bacteria (i.e., *Bacteroides xylanisolvens*, *Clostridium lavalense,* and *Roseburia inulinivorans*) and weaker sugar degradation function than mild AP patients before treatment. Moreover, etiology was one of the drivers of gut microbiome composition and explained the 3.54% variation in species’ relative abundance. The relative abundance of pathways related to lipid synthesis was higher in the gut of hyperlipidemia AP patients than in biliary AP patients. The composition and functional profiles of the gut microbiota reflect the severity and etiology of AP. Otherwise, we also identified bacterial species associated with SAP, i.e., *Oscillibacter* sp*.* 57_20*, Parabacteroides johnsonii, Bacteroides stercoris, Methanobrevibacter smithii, Ruminococcus lactaris, Coprococcus comes,* and *Dorea formicigenerans*, which have the potential to identify the SAP at an early stage.

**Importance:**

Acute pancreatitis (AP) is a type of digestive system disease with high mortality. Previous studies have shown that gut microbiota can participate in the development and treatment of acute pancreatitis by affecting the host’s metabolism. However, fewer studies acquired metagenomic sequencing data to associate species to functions intuitively and performed longitudinal analysis to explore how gut microbiota influences the development of AP. We followed 20 AP patients to generate longitudinal gut microbiota profiles and activity during disease and studied the differences in intestinal flora under different severities and etiologies. We have two findings. First, the gut microbiota profile has the potential to identify the severity and etiology of AP at an early stage. Second, gut microbiota likely acts synergistically in the development of AP. This study provides a reference for characterizing the driver flora of severe AP to identify the severity of acute pancreatitis at an early stage.

## INTRODUCTION

Acute pancreatitis (AP) is an acute inflammatory disease of the abdomen (1) with a high mortality rate (10%–30% in severe pancreatitis) (2, 3). It affects 34 people per 100,000 each year (4). Gallstones, triglycerides, alcohol, and some medications can cause AP. AP is characterized by a highly variable clinical course, making it difficult to predict its early stages. The mechanisms of AP and its treatment and outcome have been the research focus.

The gut microbiome-pancreas axis is supported by their anatomical connection via the pancreatic duct and bacterial translocation due to increased intestinal permeability (5). Studies have found that bacteria cultured from the necrotic pancreatic tissue, i.e., *Staphylococcus*, *Enterococcus*, *Escherichia coli,* and *Klebsiella*, may originate from the lower gastrointestinal tract (6 – 8). A study of the gut microbiomes of 45 AP patients and 44 healthy individuals showed that compared to healthy people, the relative abundance of *Proteobacteria* and *Bacteroidetes* was higher in patients with AP, while that of *Firmicutes* was lower (9). Disturbances in the gut microbiota may exacerbate the condition in patients with AP (10). The relative abundance of beneficial bacteria such as *Blautia* decreased in patients with severe AP (SAP) compared to mild AP (MAP) and healthy people (9). A study using 16S sequence for 30 hypertriglyceridemia-induced AP (HTG-AP) patients and 30 patients with AP induced by other causes found that the HTG-AP group had poorer microbial diversity and higher abundances of *Escherichia/Shigella* and *Enterococcus* (11). The gut flora of HTG-AP patients is more intolerant to oxidative stress, affecting the intestinal mucosa’s barrier function, and is more likely to cause other inflammation and complications (11). There is also evidence that gut microbiome composition and function have a potential association with AP severity (12), which implies that the gut microbiota has the potential to identify SAP. Although bacteria might not be the inciting reason for AP, it may be a sign of underlying pathology and play an essential role in the development and treatment of AP.

However, most of the above cross-sectional studies performed 16S rRNA sequencing of the intestinal flora for AP patients. Fewer studies acquired metagenomic sequencing data to associate species to function intuitively and performed longitudinal analysis to explore how gut microbiota influences the development of AP.

This study followed 20 AP patients with different etiologies and severities to generate longitudinal gut microbiota profiles and activity during disease (before, on the third day of treatment, and 1 month after discharge). Our metagenome sequencing data enabled us to carry out a longitudinal analysis of the gut microbiota function to explore the role of gut microbiota in the progression of AP. We obtained characteristic gut microbiota and functional profiles from patients with AP before and after treatment and patients with varying degrees of severity and different etiologies. We also found that severity was species composition-related factor and identified key bacterial species associated with SAP, which have the potential to serve as diagnostic biomarkers for SAP. Our analysis provides important insights into the complex interactions between gut microbiota and the host in acute pancreatitis.

## RESULTS

### Clinical characteristics and metagenomics sequencing data of patients with AP

We involved 20 AP patients aged 31–86, including 16 males and 4 females (Table S1). Among them, 13 were diagnosed as MAP, and 7 were severe SAP with organ failures. Patients were classified as BAP (*n* = 14) or HTG-AP (*n* = 6) based on the etiology. The serum procalcitonin (PCT), C-reactive protein (CRP), albumin (ALB), calcium (Ca), and fasting glucose (GLU) levels and the white blood cell (WBC) count were examined before treatment (pre-treatment), on the third day of treatment (on-treatment), and at 1 month return to the hospital for review (post-treatment). One month after discharge, all these clinical indicators of patients returned or tended to normal levels (Table 1; Fig. S1). No statistical differences were found in any of the indicators between MAP and SAP before treatment. However, on the third day of treatment, the serum CRP, PCR, fasting GLU levels, and WBC count of patients with SAP were higher than that of patients with MAP, while the serum ALB and Ca levels were lower in patients with SAP (*P* < 0.05; Fig. S2). All indicators showed aggravation in SAP patients at this time (Fig. S3). One month after discharge, all indicators of MAP patients returned to normal, while indicators of some SAP patients did not, suggesting the recovery of MAP was better than that of SAP. There was no indicator difference between HTG-AP and BAP patients at three treatment time points (Fig. S4 and S5).

**TABLE 1:** Biochemical indicators at various time points during treatment

Biochemical indicators	Pre-treatment	On-treatment	Post-treatment
WBC (10^9^/L)			
<4	1 (5.00%)	2 (10.00%)	2 (10.00%)
04–10	11 (55.00%)	9 (45.00%)	16 (80.00%)
>10	8 (40.00%)	9 (45.00%)	2 (10.00%)
ALB (g/L)			
<40	13 (65.00%)	14 (70.00%)	4 (20.00%)
40–55	7 (35.00%)	6 (30.00%)	16 (80.00%)
>55	0 (0.00%)	0 (0.00%)	0 (0.00%)
Fasting GLU (mmol/L)			
<3.9	0 (0.00%)	0 (0.00%)	1 (5.00%)
3.9–6.1	7 (35.00%)	8 (40.00%)	13 (65.00%)
>6.1	13 (65.00%)	12 (60.00%)	6 (30.00%)
Ca (mmol/L)			
<2.11	7 (35.00%)	10 (50.00%)	3 (15.00%)
2.11–2.52	13 (65.00%)	9 (45.00%)	13 (65.00%)
>2.52	0 (0.00%)	1 (5.00%)	4 (20.00%)
CRP (mg/L)			
0–10	4 (20.00%)	0 (0.00%)	11 (55.00%)
>10	16 (80.00%)	20 (100.00%)	9 (45.00%)
PCT (ng/mL)			
0–0.5	14 (70.00%)	9 (45.00%)	19 (95.00%)
>0.5	6 (30.00%)	11 (55.00%)	1 (5.00%)

### Drivers of gut microbiome composition of patients with AP

We collected 60 stool samples from these 20 patients before, during, and after treatment for metagenomes sequencing. We obtained an average of 73.40M reads per sample and filtered 12.39% of them, which were contaminated or low quality (Fig. S6). After filtering, more than 9 Gb of data per sample was used for the following analysis. Rarefaction curves gradually leveled off, indicating that the amount of sequencing data was large enough (Fig. S7). Sample correlations showed that AP012_1 and AP012_2 samples shared low similarity with all of the other samples (Spearman correlation, *ρ* = 0.191 for AP012_1 with other samples and *ρ* = 0.234 for AP012_2 with other samples; Fig. S8). These two samples were sequenced in another batch due to the first failed sequencing. So the differences were most likely due to the batch effects. Therefore, AP012 patients and their samples were excluded from subsequent analysis.

**Fig 1:**
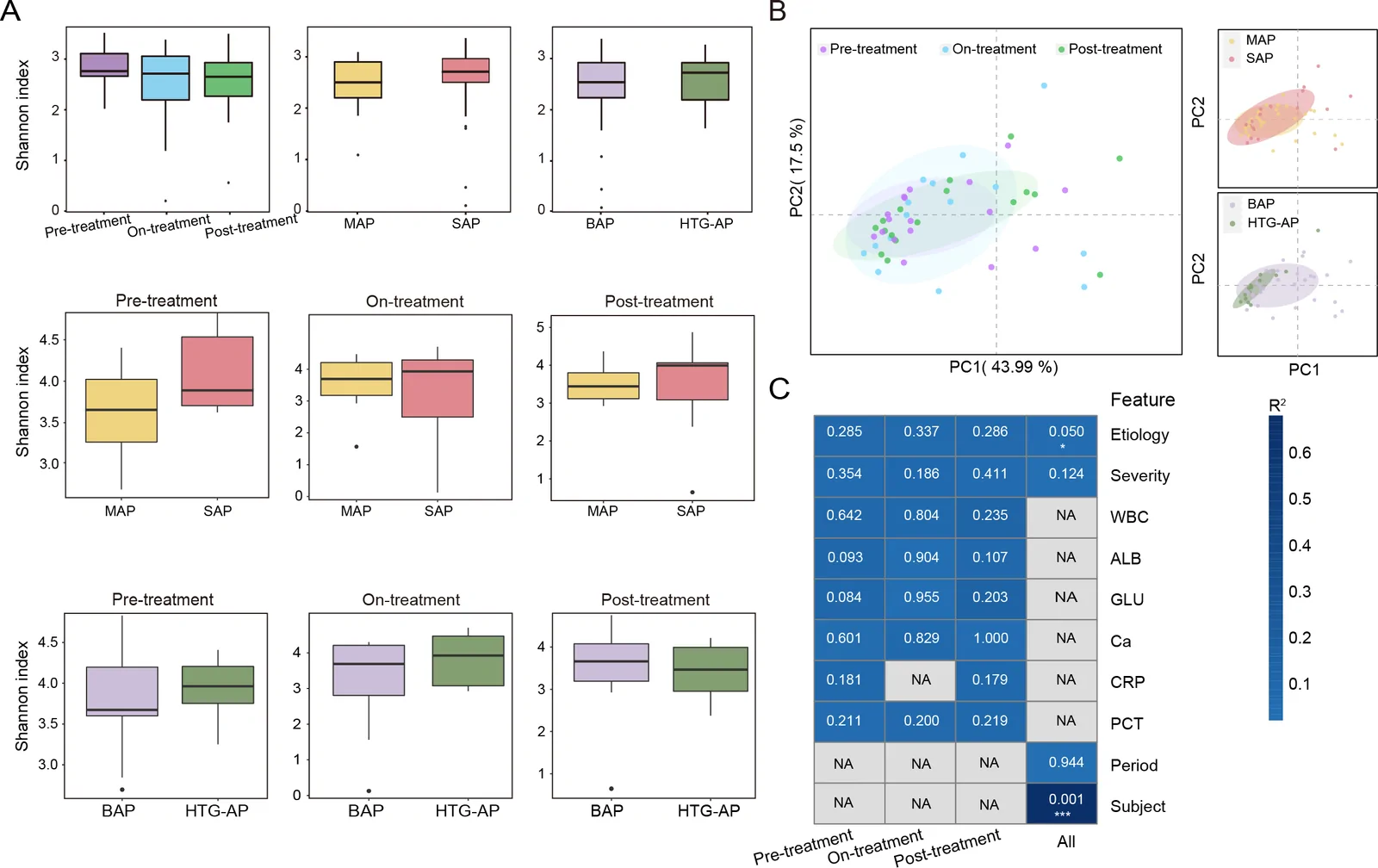
Diversity and drivers of gut microbiome composition of patients with AP. (**A**) Alpha-diversity (Shannon index) among the treatment phase, severity, and etiology groups at the species level. (**B**) PCoA of Bray-Curtis distance on all samples based on species level. (**C**) PERMANOVA test was used to detect the independent effects of clinical features on microbial community (Bray-Curtis distance, 999 permutations). The heat map was colored with *R* ^2^ values. *P* values were presented on each cell. Clinical features included subject, age, etiology, severity, WBC, ALB, GLU, Ca, CRP, and PCT. *P < 0.05, **P < 0.01, and ***P < 0.001.

The gut microbiome diversity of 19 patients with AP did not change much over time. Alpha-diversity (Shannon index) did not differ before and after treatment, between MAP and SAP, and between HTG-AP and BAP groups (*P* > 0.05; Fig. 1A). Taxonomy-based principal coordinate analysis (PCoA) showed the beta-diversity between different treatment phases, MAP and SAP groups, and HTG-AP and BAP groups (Fig. 1B). Samples from different treatment time points (*P* = 0.94) or different severity groups (*P* = 0.12) were mixed, whereas the HTG-AP and BAP samples tended to cluster with their respective groups (*P* = 0.05). We performed PERMANOVA to explain variance quantification and found that inter-individual variation contributed the majority of variance (66.7%, *P* = 0.001) in the relative abundance of species levels. Moreover, different etiologies explain 3.54% of the variation (*P* = 0.05; Fig. 1C). Other relatively large effects, including treatment time point and illness severity, accounted for a smaller proportion of variation, although these captured gut microbiome changes among and within patients, cross-sectionally and longitudinally.

### Differences in gut microbial species and function pre-, on-, and post-treatment

**Fig 2:**
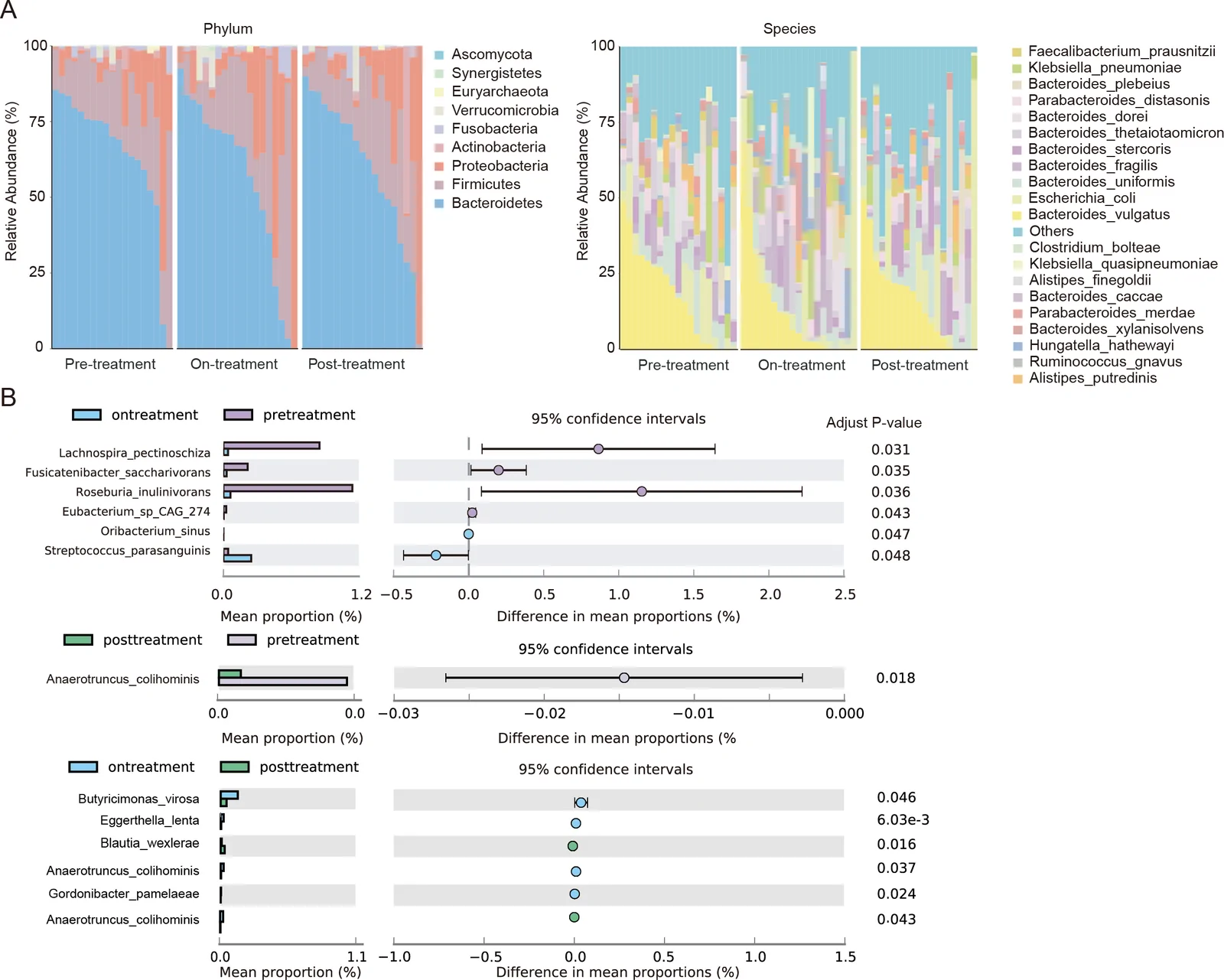
Differences in species abundance between treatment time, etiology, and severity. (**A**) Relative abundance distributions at phylum and species level in samples from individuals with AP at different stages. (**B**) Differential species of AP patients before, during, and after treatment.

The relative abundance of phylum and top 20 species did not change much over time (Fig. 2A). *Bacteroides* (59.75%) and *Firmicutes* (21.55%) were the main gut bacterial genera in patients with AP, followed by *Proteobacteria* (14.28%) and *Actinobacteria* (1.82%). At the species level, 474 species were detected, and the relative abundance of the top three species was *Bacteroides vulgatus* (15.55%), *Escherichia coli* (8.88%), and *Bacteroides uniformis* (6.51%; Table S2). Among them, the relative abundance of *Escherichia coli* increased with treatment (pre-treatment: 6.00%, on-treatment: 9.92%, and post-treatment: 10.72%). After species difference analysis, the relative abundance of four species, i.e., *Lachnospira pectinoschiza* (adjust *P* = 0.031), *Fusicatenibacter saccharivorans* (adjust *P* = 0.035), *Roseburia inulinivorans* (adjust *P* = 0.036), and *Eubacterium* sp. CAG:274 (adjust *P* = 0.043) decreased in on-treatment samples compared with pre-treatment samples (Fig. 2B). Conversely, the relative abundance of *Oribacterium sinus* and *Streptococcus parasanguinis* increased in on-treatment samples. *Anaerotruncus colihominis* also decreased after treatment (adjust *P* = 0.018, post-treatment vs pre-treatment). Six species differed in abundance between on- and post-treatment samples but were present in very low abundance (~0.1%).

**Fig 3:**
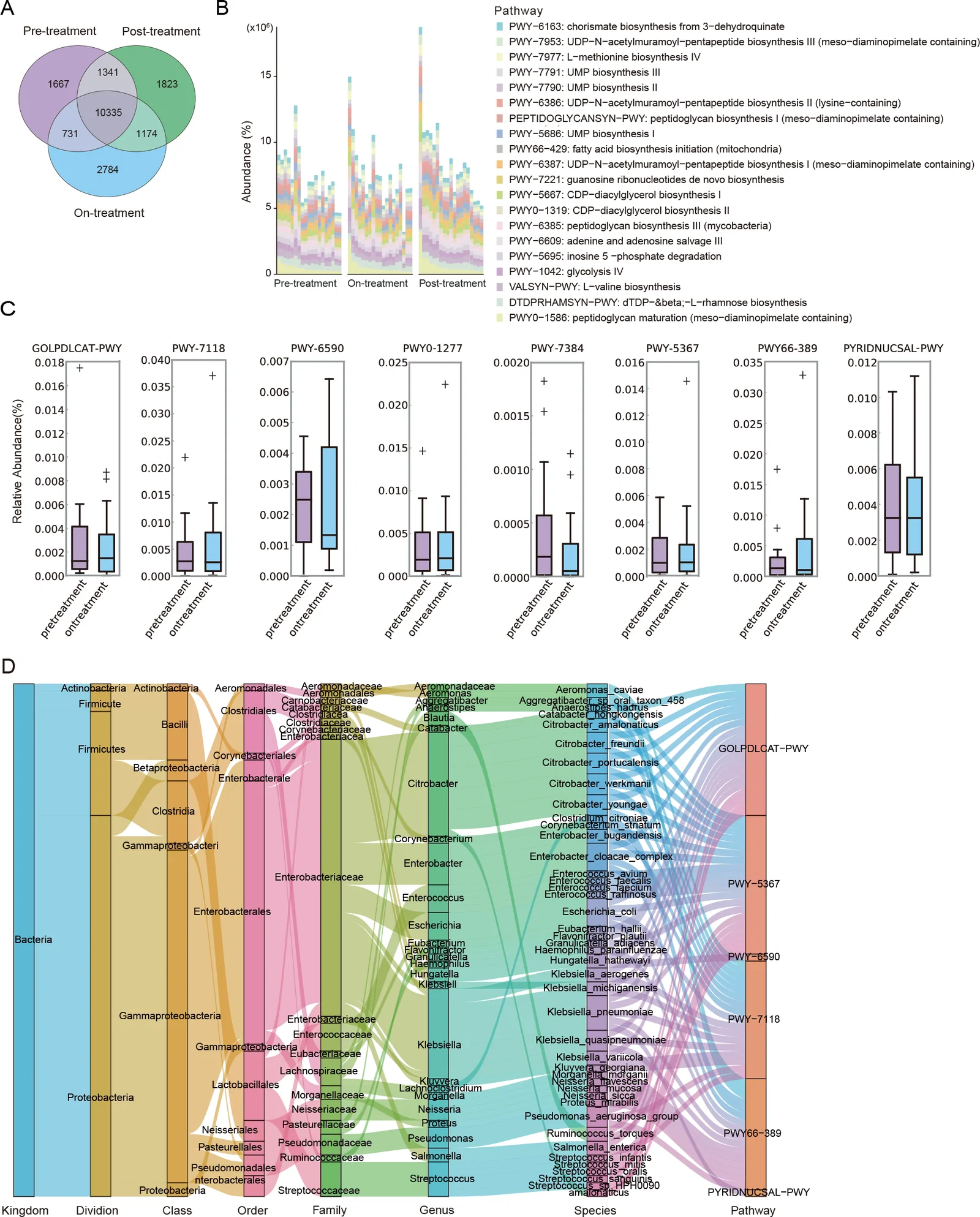
Gut microbial functions altered across treatment phase, severity, and etiology. (**A**) Wayne diagram at pathway level in samples from individuals with AP before, during, and after treatment. (**B**) Relative abundance distributions of the top 20 pathways in different treatment times. (**C**) Differential pathways in AP patients before and during treatment. Adjust *P* < 0.05. (**D**) The contributor species for the differential metabolic pathways.

The gut microbiota of patients with AP was involved in 19,855 metabolic pathways across three treatment time points and involved in the most unique pathways (*n* = 2784) on the third day of treatment (Fig. 3A; Table S4). The top five pathways were dTDP-β-L-rhamnose biosynthesis (DTDPRHAMSYN-PWY), peptidoglycan maturation (PWY0-1586), L-valine biosynthesis in glycolysis IVVALSYN-PWY (PWY-1042), and adenine and adenosine rescue III (PWY-6609; Fig. 3B). After treatment, the metabolic functions of gut microbes were partially restored. For example, the abundance of chitin deacetylation (PWY-7118) and petroselinate biosynthesis (PWY-5367) were increased on the third day of treatment (adjust *P* < 0.05). In contrast, the abundance of anaerobic energy metabolism of invertebrate mitochondria (PWY-7384), 3-(3-hydroxyphenyl) propanoate degradation (PWY0-1277), and NAD salvage pathway I (PNC VI cycle) (PYRIDNUCSAL-PWY) decreased significantly (Fig. 3C). The stratified pathway result showed that *Citrobacter* (13), *Enterobacter cloacae*, and *Enterococcus faecalis* (14) contributed to petroselinate biosynthesis. *Escherichia coli* and *Klebsiella pneumoniae* (15) were involved in chitin deacetylation (Fig. 3D).

### Beneficial bacteria decrease and sugar degradation weakened in SAP patients

**Fig 4:**
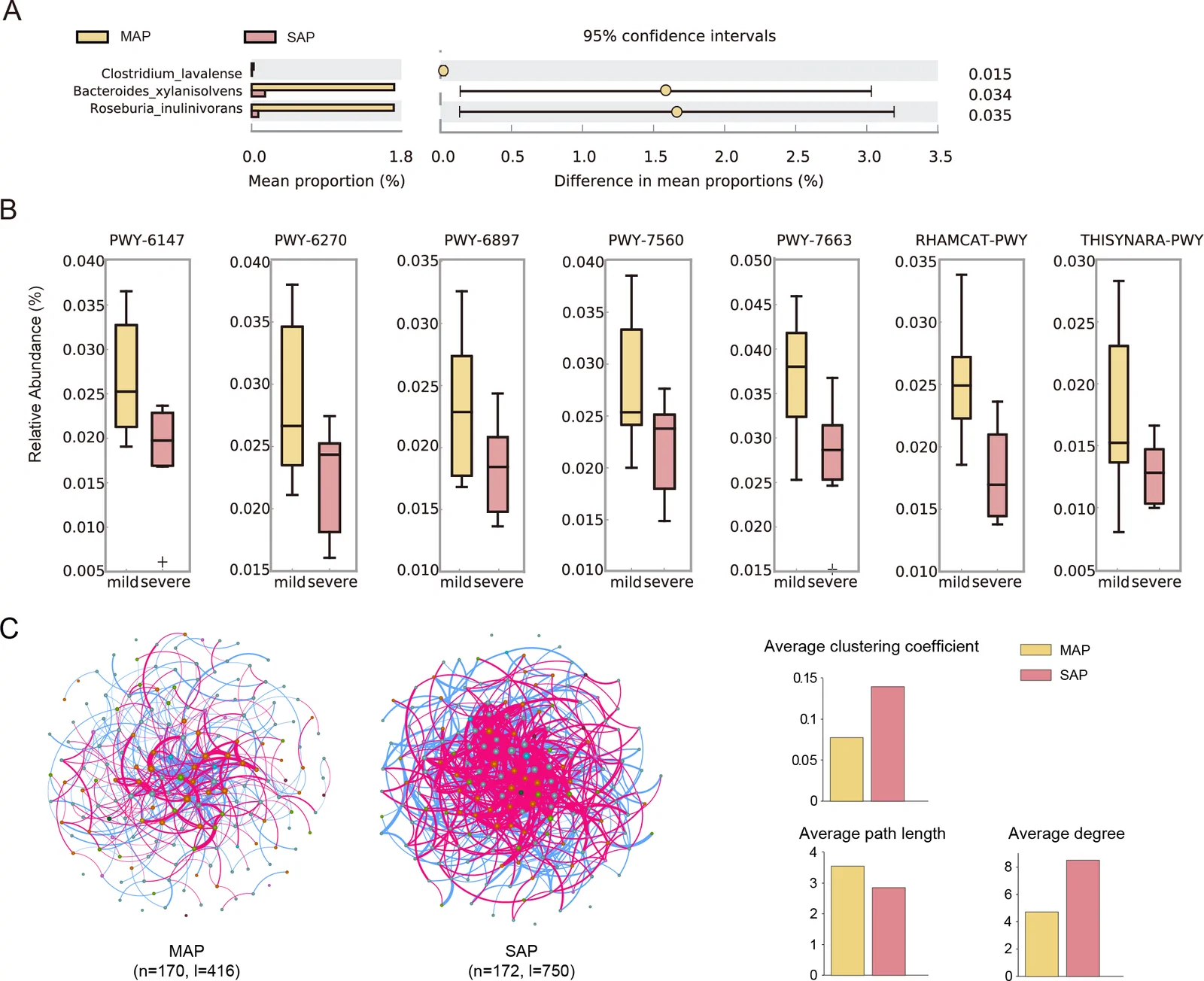
Differences in species, pathways, and networks between patients with MAP and SAP. (**A**) Differential species in samples from individuals with MAP and SAP before treatment. (**B**) Differential pathways in the relative abundance of gut microbiota in MAP and SAP patients. (**C**) Co-occurrence networks from individuals with MAP and SAP before treatment.

We compared the abundance of the species and functions between MAP and SAP patients. *Bacteroides xylanisolvens* (adjust *P* = 0.015 vs MAP group), *Clostridium lavalense* (adjust *P **=*** 0.034), and *Roseburia inulinivorans* (adjust *P **=*** 0.035) were reduced in the SAP group before treatment. *B. xylanisolvens* was also reduced in the SAP group on the third day of treatment (adjust *P* = 0.014; Fig. 4A). These three species possess probiotic qualities, show health-promoting phenotypes (16, 17), and can ferment carbohydrates to produce SCFAs (18, 19), which play an important role in inflammation and immune regulation (20, 21). This observation implied that the abundance of beneficial bacteria decreased in SAP compared to MAP.

Moreover, compared with the MAP group, the relative abundance of six pathways decreased in the SAP group before treatment (Fig. 4B; Table S5). These six pathways were 6-hydroxymethyl-dihydropterin diphosphate biosynthesis I (PWY-6147; adjust *P* = 0.025), L-rhamnose degradation I (RHAMCAT-PWY; adjust *P* = 0.009), gondoate biosynthesis of anaerobic flora (PWY-7663; adjust *P* = 0.033), methylerythritol phosphate pathway II (PWY-7560; adjust *P* = 0.040), thiamine diphosphate salvage II(PWY-6897; adjust *P* = 0.047), and isoprene biosynthesis I (PWY-6270; adjust *P* = 0.004).

Firmicutes, Bacteroidetes, and Proteobacteria formed the co-occurrence networks of SAP and MAP groups before treatment. The network of patients with MAP had 170 nodes and 416 links, while the SAP network had 172 nodes and 750 links. The SAP network showed a higher clustering coefficient (0.14 vs 0.08), a greater average degree (8.52 vs 4.73), and a shorter path length (2.86 vs 3.56) compared to the MAP network, indicating a more modular and complex network (Fig. 4C; Table S7). To explore the network function, we extracted the genes of all species in the network and mapped them to the Kyoto Encyclopedia of Genes and Genomes (KEGG) pathway. Species in SAP networks participated in bacterial motility (bacterial chemotaxis and flagellar assembly) and biofilm formation of opportunistic pathogens, i.e., *Pseudomonas aeruginosa*, *Escherichia coli*, and *Vibrio cholera* (Table S8).

### Lipid synthesis functions enhanced in HTG-AP

**Fig 5:**
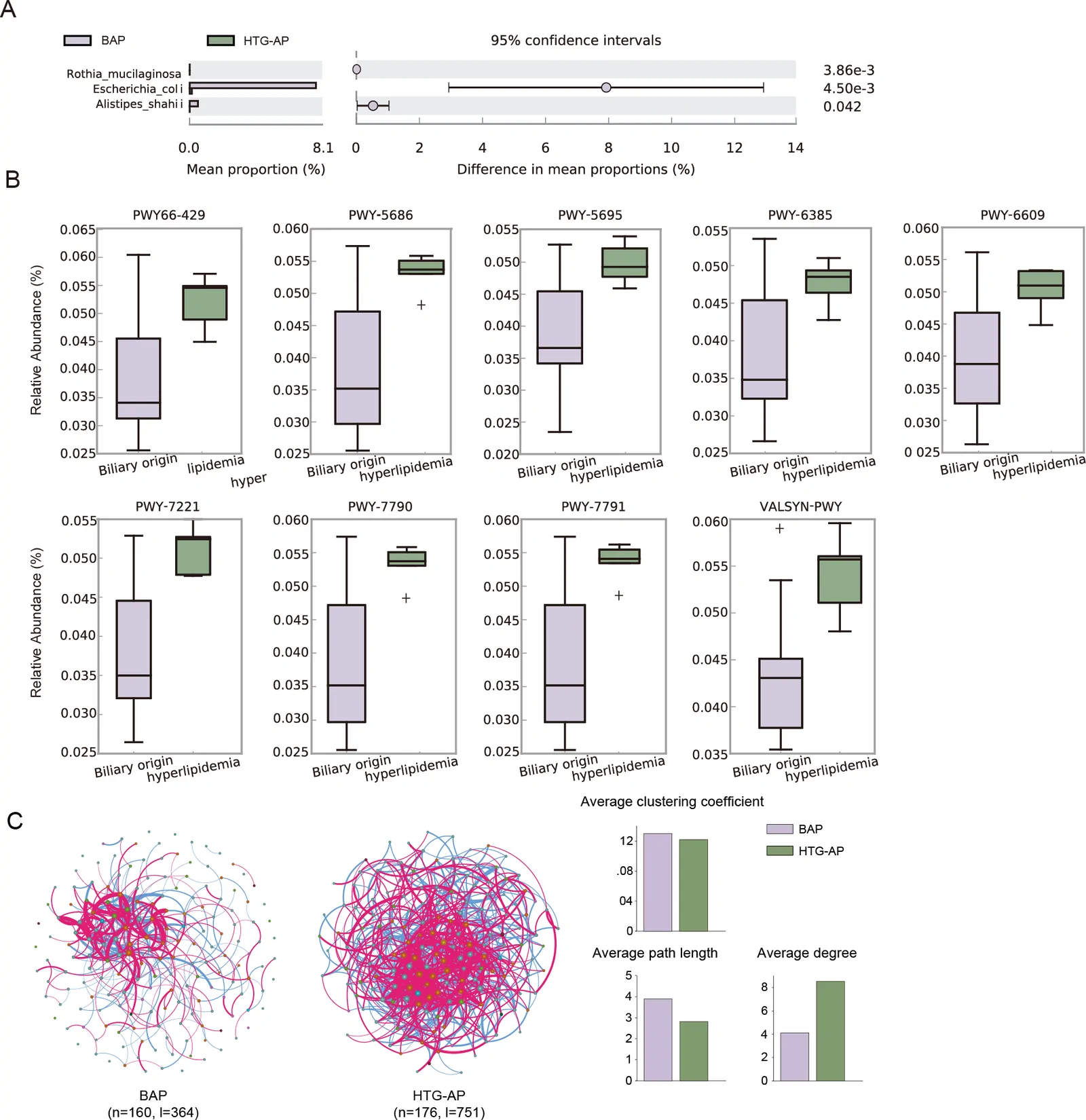
Differences in species, pathways, and networks between patients with BAP and HTG-AP. (**A**) Differential species in samples from individuals with BAP and HTG-AP before treatment. (**B**) Differential pathways between BAP patients and HTG-AP patients. (**C**) Co-occurrence network from individuals with BAP and HTG-AP before treatment.

There was little difference in species between the HTG-AP and BAP groups. Only *Alistipes shahii* (adjust *P* = 0.042, vs HTG-AP group) and *Escherichia coli* (adjust *P* = 0.0045) were enriched in the BAP group before treatment (Fig. 5A). Nine pathways were enriched in HTG-AP groups (adjust *P* < 0.05; Fig. 5B; Table S6). These pathways included the degradation of inosine 5′-phosphate (PWY-5695), the salvage of adenine and adenosine (PWY-6609), and the biosynthesis of UMP (PWY-5686, PWY-7790, and PWY-7791), fatty acid (PWY66-429), guanosine ribonucleotides (PWY-7221), L-valine (VALSYN-PWY), and peptidoglycan (PWY-6385).

There were 160 nodes and 364 links in the BAP network and 176 nodes and 751 links in the HTG-AP network. The HTG-AP network was more complex. The topological structure analysis indicated that the BAP network has a longer average path length (3.90 vs 2.82) and lower average degree (4.14 vs 8.53) than those in the HTG-AP network, suggesting the lower connectivity and information transferring efficiency in BAP patients (Fig. 5C; Table S7).

### Species correlated with clinical indicators

**Fig 6:**
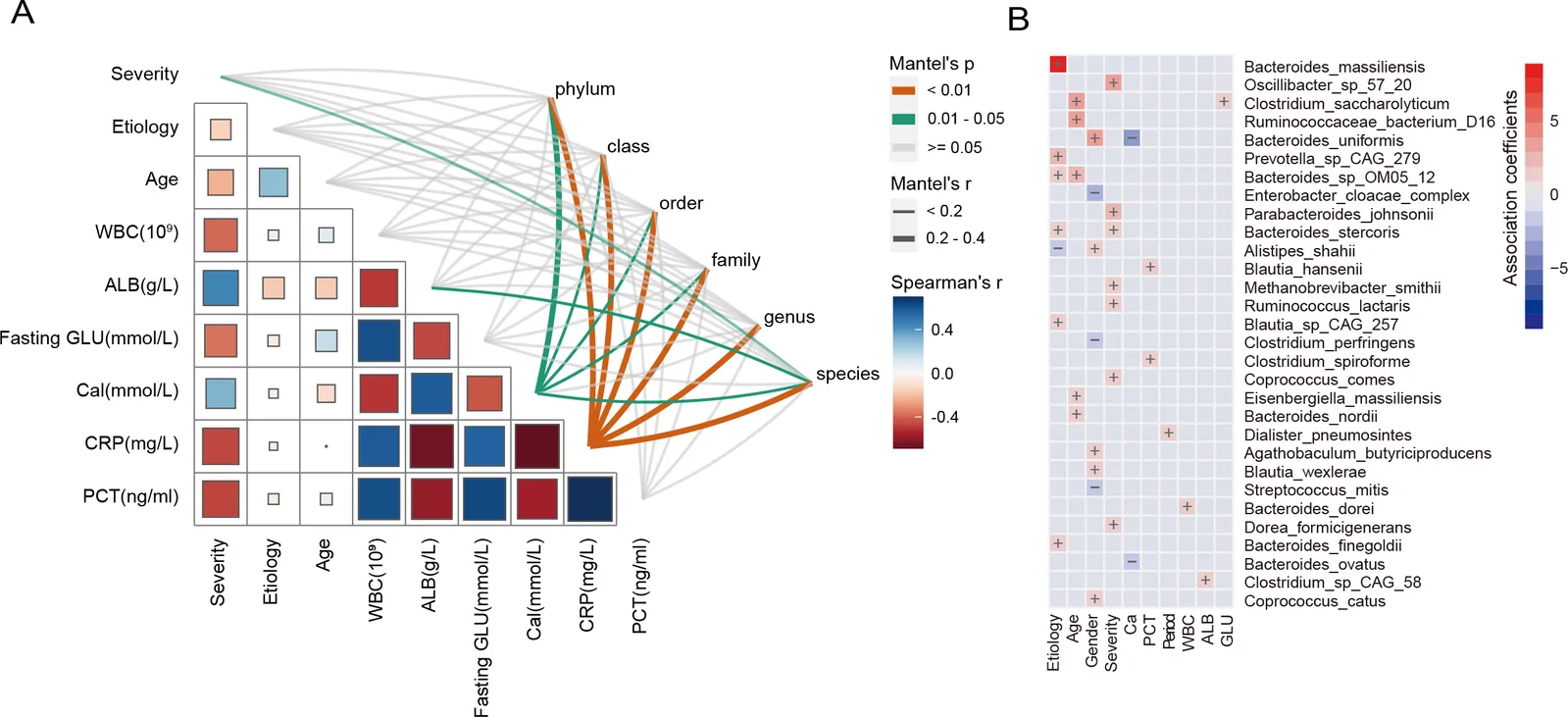
Associations between microbiome and clinical indicators. (**A**) Association between microbiome composition and clinical features. The Mantel test was used to find correlations among distance matrices of samples and clinical features (Bray-Curtis and Euclidean distance). Correlation values (Spearman) were presented on each cell. Independent variables include the taxonomy of phylum, class, order, family, genus, and species. (**B**) Associations between species and clinical indicators. Red represents a positive correlation, and blue represents a negative correlation. The color depth represents the size of the correlation. “+” represents a positive correlation, “−” represents a negative correlation.

To further identify the clinical characteristics most associated with the microbiome components, we correlated taxonomic community composition at phylum, class, order, family, genus, and species levels with nine clinical characteristics (Fig. 6A). Overall, serum CRP (Mantel test, *r* >0.2, *P* < 0.01) and Ca (Mantel test, *r* >0.2, *P* < 0.05) levels were the strongest correlates of all levels of taxonomic composition. The AP severity (*r* <0.2, *P* < 0.01) and serum ALB (*r* <0.2, *P* < 0.05) were related to species composition. Previous studies have demonstrated that serum CRP, Ca, and ALB were predictive markers of SAP. No significant correlation was found for AP etiology, age, WBC count, fasting GLU, and serum PCT.

MaAsLin2 analysis was conducted to identify viral species associated with clinical indicators. Severity, Ca, and ALB showed significant correlations with certain species. The SAP samples showed enrichment of *Oscillibacter* sp. 57_20, *Parabacteroides johnsonii*, *Bacteroides stercoris*, *Methanobrevibacter smithii*, *Ruminococcus lactaris*, *Coprococcus comes*, and *Dorea formicigenerans. Bacteroides uniformis* and *Bacteroides ovatus* were negatively related to Ca. *Clostridium* sp. CAG:58 was positively associated with ALB (Fig. 6B).

## DISCUSSION

AP is a disease closely related to digestion. Previous studies have shown that gut microbiota can participate in developing and treating acute pancreatitis by affecting the host’s metabolism and intestinal mucosal permeability. Interestingly, the diversity of the gut microbiome of patients with AP did not show much variation with treatment, while the metabolic functions of the gut microbiota were reinstated after treatment. Gut microbiota has functional redundancy; multiple species may participate in the same functional pathway (22, 23). It is possible that differential pathways may result from differences in the abundance of multiple species rather than a single significantly different species. Of the only six species that differed in relative abundance between pre- and on-treatment, *L. pectinoschiza* is a common and important anaerobic pectinophilic bacterium (24) that can only ferment pectin with a few related compounds. Pectin can inhibit the hydrolysis of substances such as triacylglycerols by pancreatic lipase (25, 26). One of the telltale signs of AP is the massive release of pancreatic lipase due to damaged acinus, and high levels of pancreatic lipase may induce and maintain ketosis or ketoacidosis secondary to AP (27). Thus, a decrease in the abundance of *L. pectinoschiza* after 3 days of treatment might increase intestinal pectin content and mitigate the damage caused by high pancreatic lipase. The differential metabolic pathways with increased abundance after treatment, i.e., chitin deacetylation, phytol degradation, and superpathway of *Clostridium acetobutylicum* acidogenic fermentation, alleviate the symptoms of pancreatitis in a manner that promotes accelerated metabolism. Chitin deacetylation is a pathway that primarily catabolizes the polysaccharide chitin (28). Superpathway of *Clostridium acetobutylicum* acidogenic fermentation is mainly distributed in *Clostridium acetobutylicum*, with butyric acid as the main product, and its contained pathway from acetyl-CoA to butanol-CoA is an important carbon metabolism channel (29). Petroselinate biosynthesis synthesizes salicylic acid with salicylate (30), and rock alginate is vital in inhibiting cholesterol absorption and promoting cholesterol excretion (31). Elevation of this pathway inhibits cholesterol uptake and, to some extent, relieves the stress of elevated lipids in patients with pancreatitis.

In addition, we found that the SAP patients contained less beneficial bacteria and weaker sugar degradation function than MAP patients before treatment. Structural disorders of the intestinal flora affect the metabolic profile of the host, particularly the degradation of sugars and lipids (32). AP causes the body’s sugar degradation mechanisms to be impaired, and up to 40% of patients develop new-onset diabetes after the first episode of AP (33). MAP and SAP differed significantly in the relative abundance of pathways regarding sugar degradation. The relative abundance of the differential pathways L-rhamnose degradation I and thiamine diphosphate salvage II was decreased in SAP compared to patients with MAP. Thus, the ability of rhamnose degradation in SAP became weaker. Thiamine diphosphate salvage II indirectly affects glucose metabolism by generating thiamin diphosphate, which are active derivatives of thiamin (34) and are important coenzymes of central metabolism (35, 36). When thiamin is deficient, the sugar oxidation of the body is blocked to form pyruvate and lactate accumulation, which affects the body’s energy supply. Besides, the abundance of three beneficial bacteria, i.e., *B. xylanisolvens*, *C. lavalense*, and *R. inulinivorans*, was decreased in SAP compared with MAP. These three species play an important role in inflammation and immune regulation (20, 21). Species in SAP co-occurrence networks were involved in bacterial motility and biofilm formation of opportunistic pathogens. This suggests that the gut microbiome of SAP patients tended to bacterial translocation and increase the growth of opportunistic pathogens. We speculated that the gut microbiome might exacerbate or indirectly exacerbate AP symptoms, as evidenced by previous research (11, 37, 38).

Furthermore, early recognition of the SAP form is a big challenge. AP severity and its predictive markers, i.e., serum CRP, Ca, and ALB levels, were taxonomic composition-related factors. Clinically, CRP, blood urea nitrogen, and serum creatinine are accepted indicators to predict an episode of SAP (39, 40). Other markers, such as PCT, interleukin 6 (IL-6), and acute phase proteins (LBP, SAA, PTX3), have shown some promising results (40). However, all of the above biomarkers are affected by other diseases and have a certain lag in evaluating inflammation in the body. CRP is greatly influenced by non-infectious factors such as cardiovascular disease (41, 42) and autoimmune disease (43). Patients with thyroid disease (44) and lung cancer (45) may experience increased PCT without significant inflammation in the body. Thus, the SAP-related species, i.e., *Oscillibacter* sp. 57_20*, Parabacteroides johnsonii, Bacteroides stercoris, Methanobrevibacter smithii, Ruminococcus lactaris, Coprococcus comes,* and *Dorea formicigenerans*, had the potential to be the novel predictive marker of SAP.

This study has several limitations, which are expected to be improved in future studies. On the one hand, we included relatively small sizes of cohorts, mainly because many patients were lost at follow-up. Inter-individual variation contributed 66.7% of the variance in our study’s relative abundance of species’ levels. This is a common problem in metagenomic studies. Lloyd-Price et al. (46) analyzed 2,965 stool metagenomes from 132 individuals, and its inter-individual variation accounted for 66.6% of the variance. Following the practice of some studies (47 – 49), we partially circumvented the sample size limitation by using strict inclusion criteria to reduce individual differences and metagenomic sequencing to maximize the information obtained from each sample. In addition, longitudinal studies can control for confounding factors (50 – 52). Some results consistent with prior studies and meet the AP status can validate our study’s reliability to some extent. For example, *Bacteroides* were the main gut bacterial genera in patients with AP (53), and the abundance of beneficial bacteria was decreased in SAP (54). The relative abundance of the fatty acid pathway, the initiator of fatty acid biosynthesis (28), is higher in patients with HTG-AP than in patients with BAP. This might be related to elevated lipids, such as blood triglycerides, in patients with HTG-AP (55). The relative abundance of L-valine, a pathway concerning L-valine biosynthesis, was lower in patients with cholestatic pancreatitis compared to patients with HTG-AP, which is consistent with the results obtained from gas chromatography-mass spectrometry-based (GC-MS-based) analysis (56). Meanwhile, we also noticed that Hu et al. (11) found that the HTG-AP group had poorer microbial diversity than the non-HTG-AP group. However, we found no difference in alpha diversity between HTG-AP and BAP. The possible reasons are as follows. First, in our study, the comparison group for HTG-AP patients was BAP patients, while Hu et al. included various non-HTG-AP patients. Second, Hu et al. used 16S sequencing data, while we used metagenomic data. Previous studies have found inconsistencies between alpha diversity calculated from metagenomic data and 16S sequencing data (8). Metagenomic sequencing data can identify more species than 16S sequencing data (57). On the other hand, this paper only conducts empirical analysis based on genome sequencing data and lacks animal experiments to explore further the mechanism underlying these observations. Given the above limitations, we plan to collect more samples from AP patients to track taxonomic and functional alterations of gut microbiota for the verification of the current findings. The new-enrolled samples are also used for fecal microbiota transplantation to explore the function of the target species and their influences on AP patients.

Overall, we explored the differences in the gut microbiota of AP patients cross-sectionally and longitudinally. The treatment improved the metabolic function of the patient’s gut microbiota but did not significantly alter the gut microbial composition. Moreover, the less beneficial bacteria and weaker sugar degradation function were the characteristic alterations in the gut microbiome of patients with SAP. The SAP-related species we identified have the potential to be biomarkers for the early identification of SAP.

## MATERIALS AND METHODS

### Patient recruitment and sample collection

In this study, we recruited 20 patients diagnosed with AP and categorized them by severity (7 SAP and 13 MAP) and etiology (14 BAP and 6 HTG-AP). The severity and etiology of AP were diagnosed based on the guidelines for diagnosing and treating acute pancreatitis in China (2021) (58). Exclusion criteria were as follows: (i) patients less than 18 years; (ii) pregnant and lactating women; (iii) patients with endoscopic retrograde cholangiopancreatography; (iv) AP caused by biliary and pancreatic tumors; (v) patients with an acute attack of chronic pancreatitis; (vi) patients with basic diseases such as severe cardiovascular disease, respiratory system disease, liver disease, kidney disease, and malignant tumor; and (vii) patients taking antibiotics within 3 months.

Stool samples were collected from AP patients at three time points: before treatment (pre-treatment), on the third day of treatment (on-treatment), and at 1 month return to the hospital for review (post-treatment). Sixty fresh stool samples were flash frozen in liquid nitrogen and stored at −80°C until metagenomic sequencing. The corresponding clinicians collected clinical and disease progression data (Table S1). The Ethics Committee of Zhuhai People’s Hospital (Zhuhai Hospital Affiliated with Jinan University) approved the research. All patients provided signed informed consent. We experimented according to the official guidelines issued by the National Health and Family Planning Commission of China.

### DNA extraction, library construction, and sequencing

DNA extraction, library construction, and sequencing were performed by BGI. Stool samples were shipped on dry ice to the laboratory for metagenomic sequencing. The microbial community DNA was extracted using MagPure Stool DNA KF Kit B (Magen, China) according to the manufacturer’s instructions. DNA was quantified with a Qubit Fluorometer (Invitrogen, USA). The quality of DNA was checked by running an aliquot on 1% agarose gel. DNA was randomly fragmented by Covaris and selected by magnetic beads to an average size of 200–400 bp to construct the library. The qualified libraries were sequenced on the MGI-SEQ2000 platform using the 150 bp pair-end configuration (MGI, Shenzhen, China).

### Sequence data processing

The raw reads were filtered to remove adapter sequences, low-quality reads (over 50% bases with Phred quality ≤5), unknown bases (>10% “N” bases), and host DNA (human, hg38) by using SOAPnuke v1.5.2 (59) and KneadData v0.10.0 (The Huttenhower lab, http://huttenhower.sph.harvard.edu/kneaddata). Taxonomic and functional profiling of microbial communities was generated with tools from the bioBakery meta-omics analysis environment (http://huttenhower.sph.harvard.edu/biobakery) (60). MetaPhlAn3 v3.0.14 was used for taxonomic classification and quantification (relative abundance) based on CHOCOPhlAn database v3.0.14. Functional genomic profiles, including gene families and pathways, were quantified from HUMAnN3 v3.0.1 (61). The abundance of gene families and pathways was normalized by humann2_renorm_table script.

### Metagenomic data analysis, statistics, and visualization

Data analysis and visualization were performed using R 4.1.1. We used vegan v2.6-2 to calculate the Shannon index to evaluate alpha diversity and plyr v1.8.7 to perform PCoA based on Bray-Curtis. The correlations of samples were calculated by Spearman analysis. We used the adonis function in the vegan package to conduct the PERMANOVA analysis to detect the independent effects within and between groups of patients. Heatmaps were generated by pheatmap v1.0.12. The significant differences in the abundance of the species and pathways were explored by STAMP v2.1.3 (http://kiwi.cs.dal.ca/Software/STAMP) and pgirmess v2.0.0 (R package), respectively. The networks were constructed and visualized by Gephi using the Fruchterman Reingold layout. The correlations of species were calculated by Sparse Correlations for Compositional data (62) using the SpiecEasi R package (*r* >0.20, *P* < 0.05). The network dissimilarity was identified following Mo et al. (63). The correlations between taxonomic community composition and nine clinical characteristics were analyzed by LinkET v0.0.3.7. To obtain the association between species and clinical indicators, we filtered out species with an average relative abundance <0.01 and focused our analysis on the remaining 176 species. The percentages of species were arcsin-square-root transformed by taking the arcsine of the square root of the abundances of species. The associations of individual species to each factor were performed by Multivariate Association with Linear Model (MaAsLin) (64) with parameters (normalization=“NONE,” transform=“NONE”) and other parameters as default parameters. The heatmap was plotted from the resultant importance value calculated by [−log(qval)*sign(coeff)]. The *P*-value was adjusted by the error detection rate . Significance testing of biochemical indicators was carried out with the Student’s *t*-test or ANOVA test with Bonferroni correction. The *P*-value or adjusted *P*-value less than 0.05 was considered significant.

## Data Availability

The data sets presented in this study can be found in online repositories. The names of the repository/repositories and accession number(s) can be found below: China National GeneBank DataBase (CNGBdb) with accession number CNP0003989.

## SUPPLEMENTAL MATERIAL

Supplemental Files spectrum.00829-23-s0001.pdf Supplemental figures and tables.

ASM does not own the copyrights to Supplemental Material that may be linked to, or accessed through, an article. The authors have granted ASM a non-exclusive, world-wide license to publish the Supplemental Material files. Please contact the corresponding author directly for reuse.
