# Erratum for Wang et al., “Composition and functional profiles of gut microbiota reflect the treatment stage, severity, and etiology of acute pancreatitis”

**DOI:** 10.1128/spectrum.01520-24

**Published:** 2024-09-05

**Authors:** Zhenjiang Wang, Mingyi Guo, Jing Li, Chuangming Jiang, Sen Yang, Shizhuo Zheng, Mingzhe Li, Xinbo Ai, Xiaohong Xu, Wenbo Zhang, Xingxiang He, Yinan Wang, Yuping Chen

## ERRATUM

Volume 11, no. 5 , e00829-23, 2023, https://doi.org/10.1128/spectrum.00829-23. Page 14, Ethics Approval: "Ethics Committee of the Affiliated Hospital of Qingdao University" should read "Ethics Committee of Zhuhai People’s Hospital (Zhuhai Hospital Affiliated with Jinan University)."

