# Supplementary material for: Composition and functional profiles of gut microbiota reflect the treatment stage, severity, and etiology of acute pancreatitis
Source: Microbiol Spectr. 2023 Sep 12;11(5):e00829-23. doi: 10.1128/spectrum.00829-23 (PMC10580821; doi:10.1128/spectrum.00829-23)
Supplement: Supplemental Files — Supplemental figures and tables. [file spectrum.00829-23-s0001.pdf]

Supplementary Figures

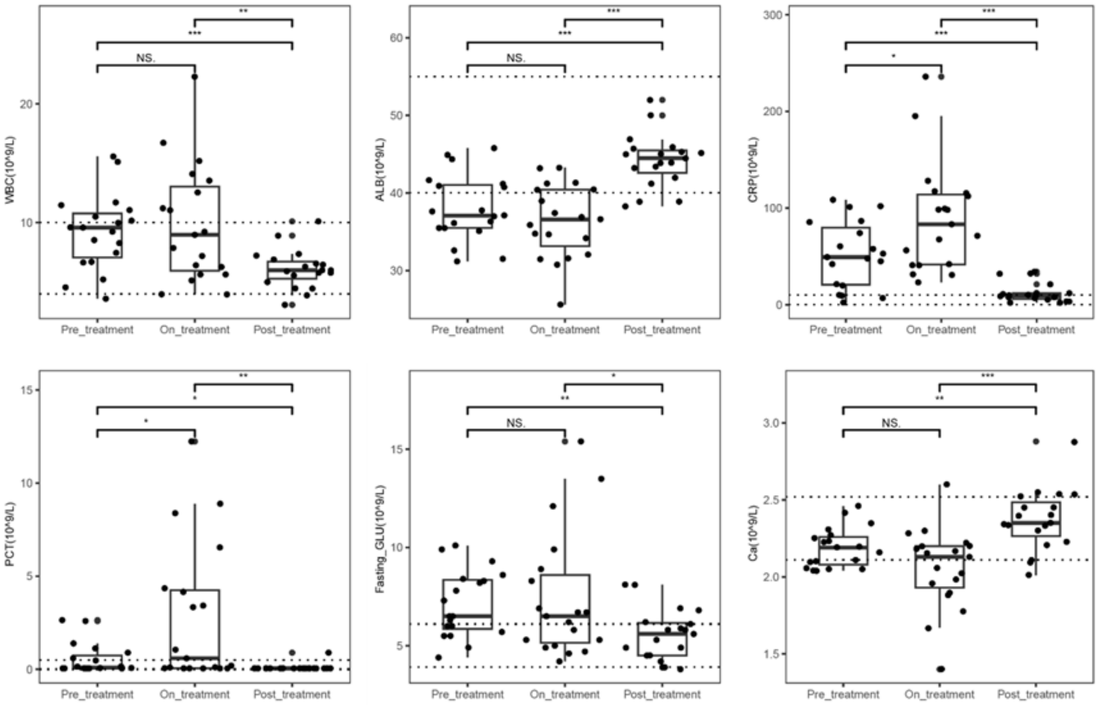

Figure S1. Clinical indicators variations before, on, and after treatment. Normal values are between the dashed lines. **P* < 0.05, ***P* < 0.01, ****P* < 0.001, NS. *P* > 0.05.

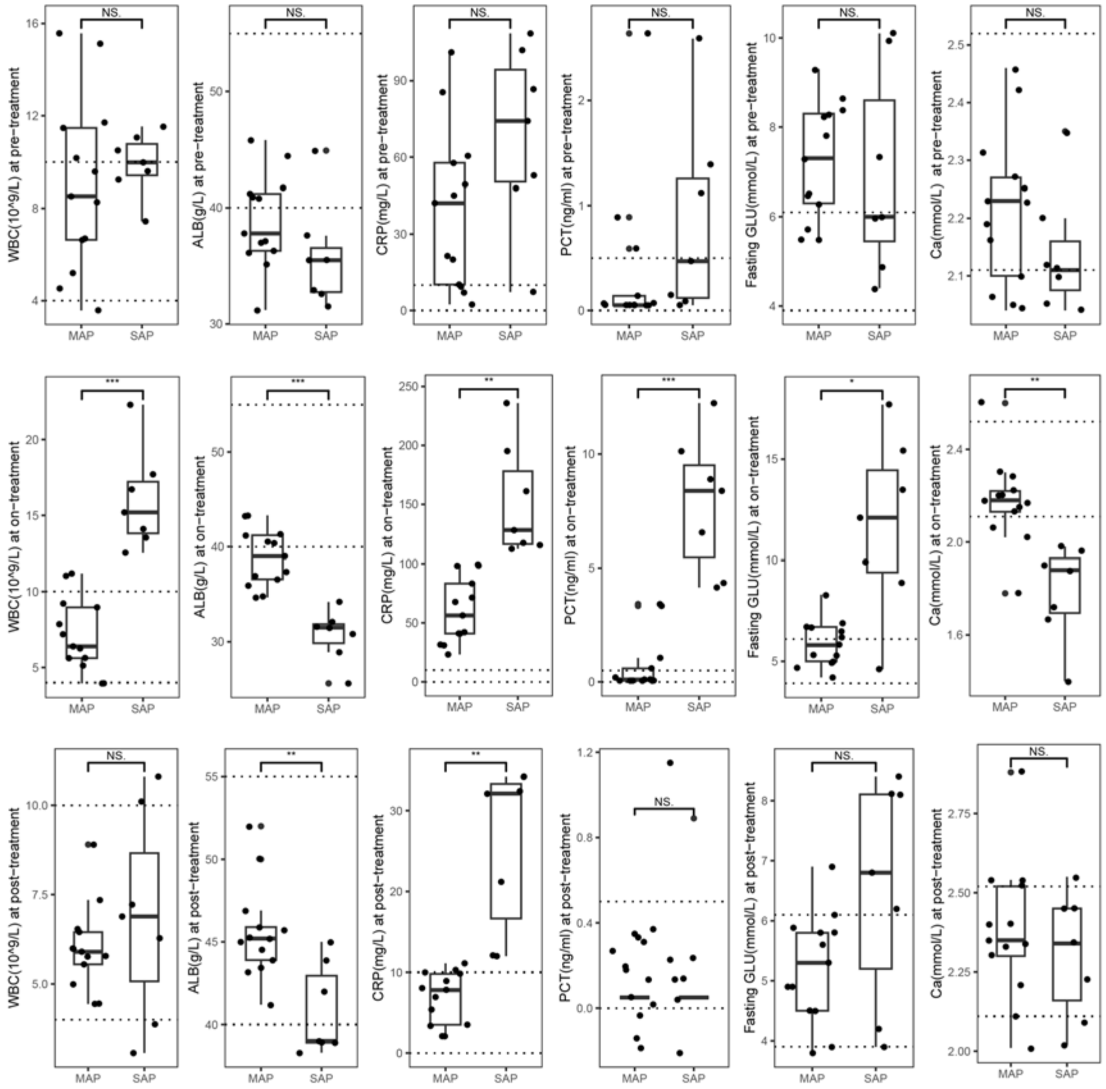

Figure S2. Differences in all clinical characteristics between MAP and SAP patients. Normal values are between the dashed lines. **P* < 0.05, ***P* < 0.01, ****P* < 0.001, NS. *P* > 0.05.

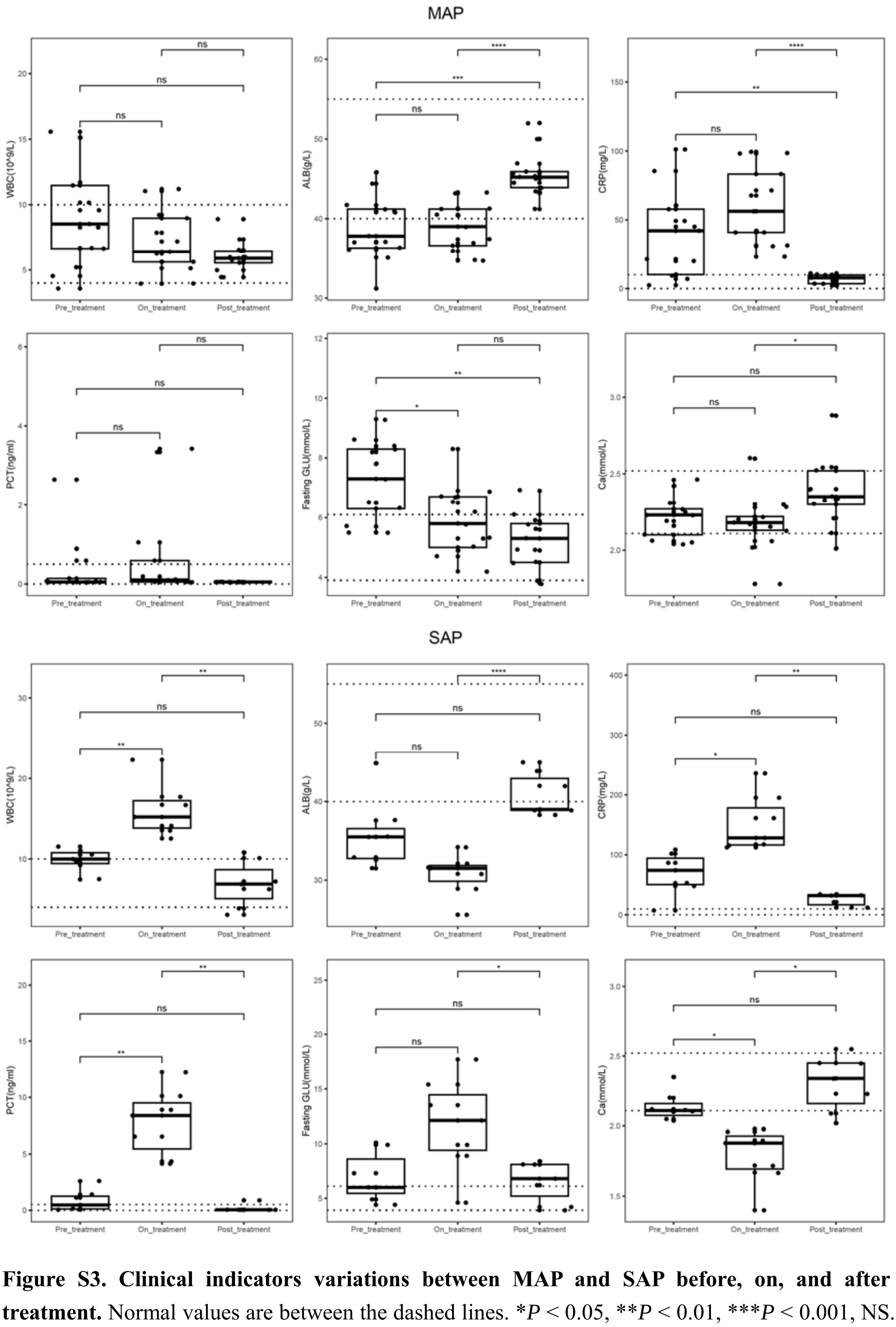

Figure S3. Clinical indicators variations between MAP and SAP before, on, and after treatment. Normal values are between the dashed lines. **P* < 0.05, ***P* < 0.01, ****P* < 0.001, NS. *P* > 0.05.

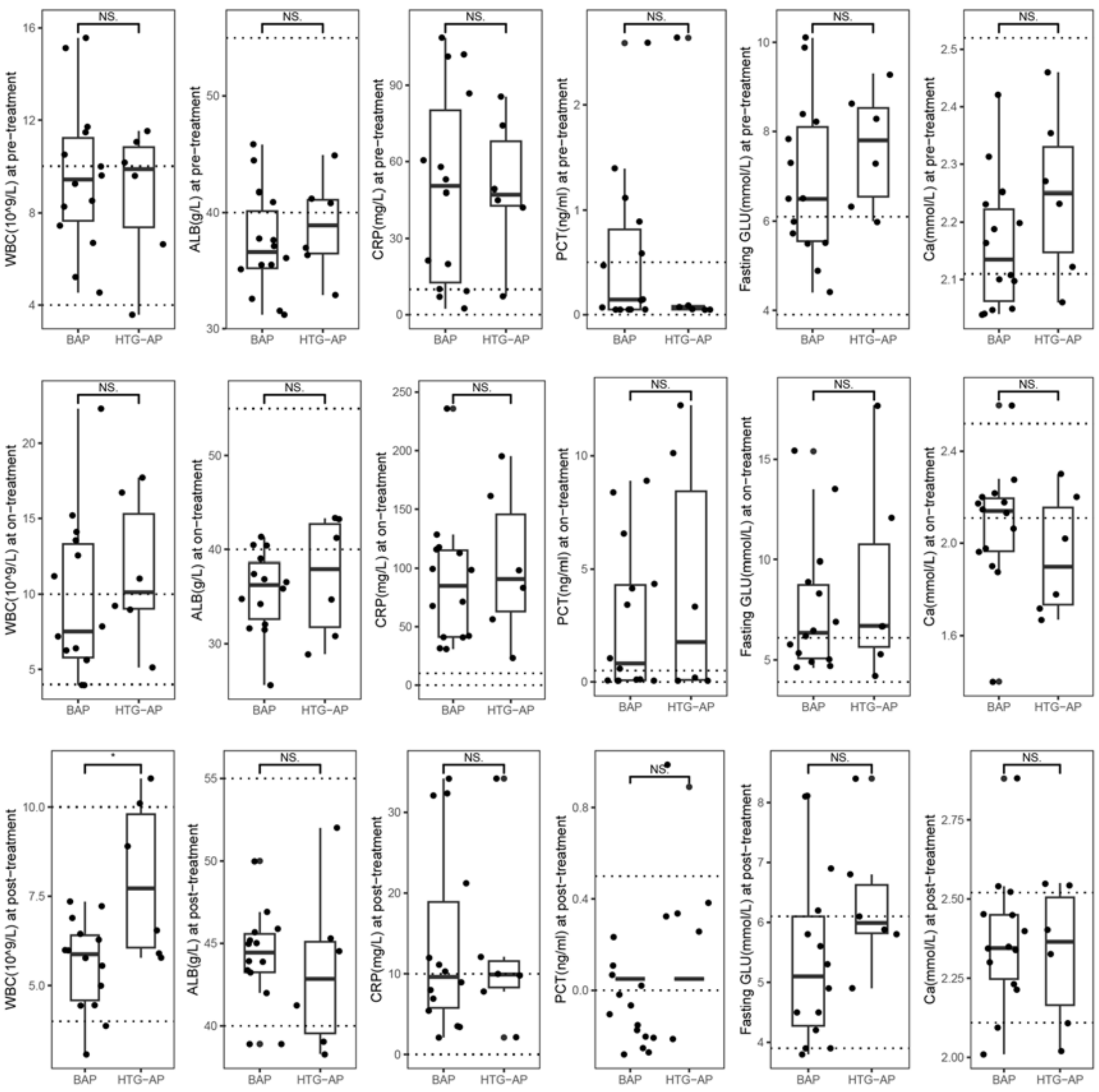

Figure S4. Differences in all clinical characteristics between BAP and HTG-AP groups. Normal values are between the dashed lines. *$P < 0.05$, **$P < 0.01$, ***$P < 0.001$, NS. $P > 0.05$.

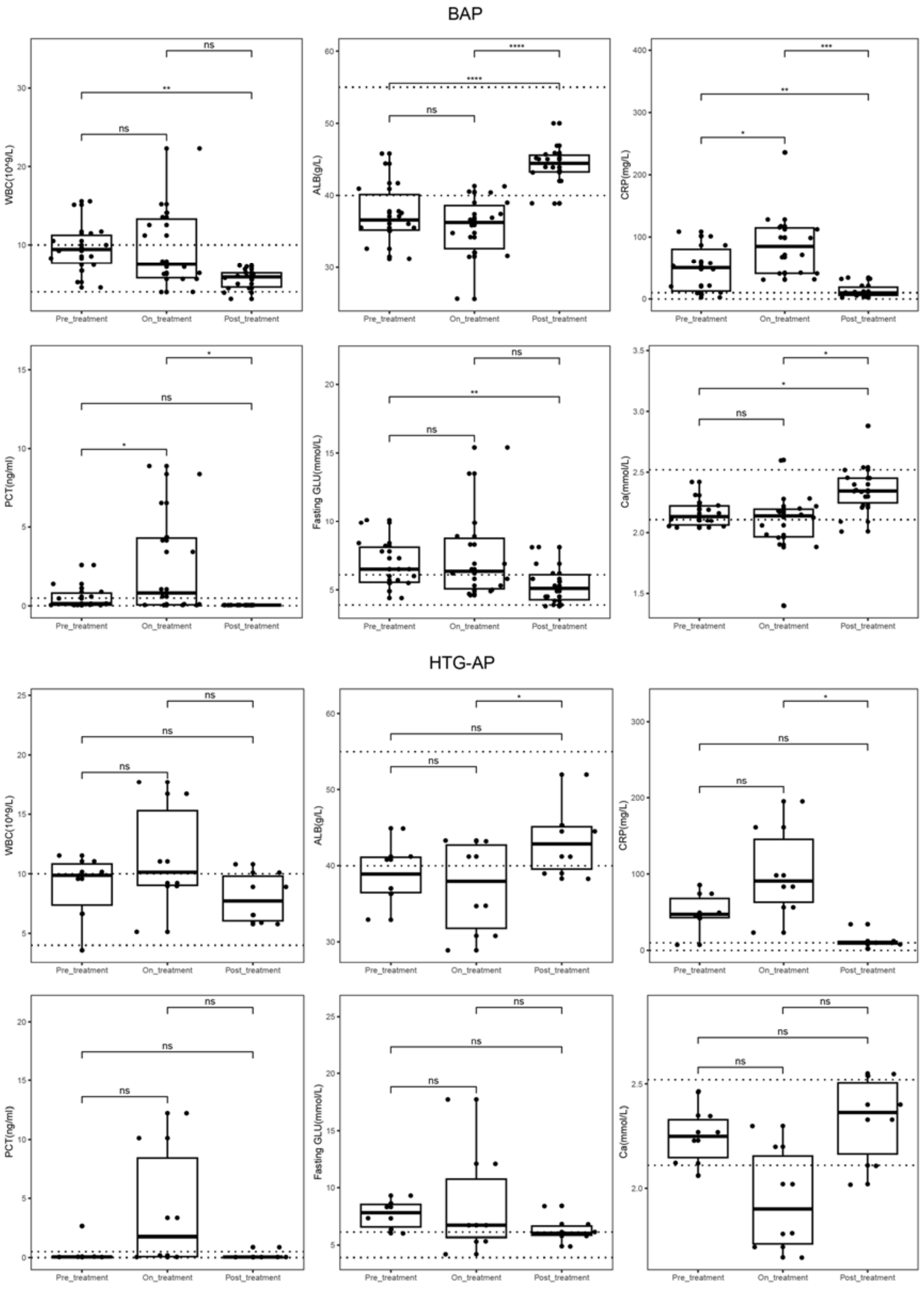

Figure S5. Variation of clinical indicators with treatment for BAP and HTG-AP samples. The clinical indexes of MAP and SAP patients before, during, and after treatment. Normal values are between the dashed lines. *P < 0.05, **P < 0.01, ***P < 0.001, NS. P > 0.05.

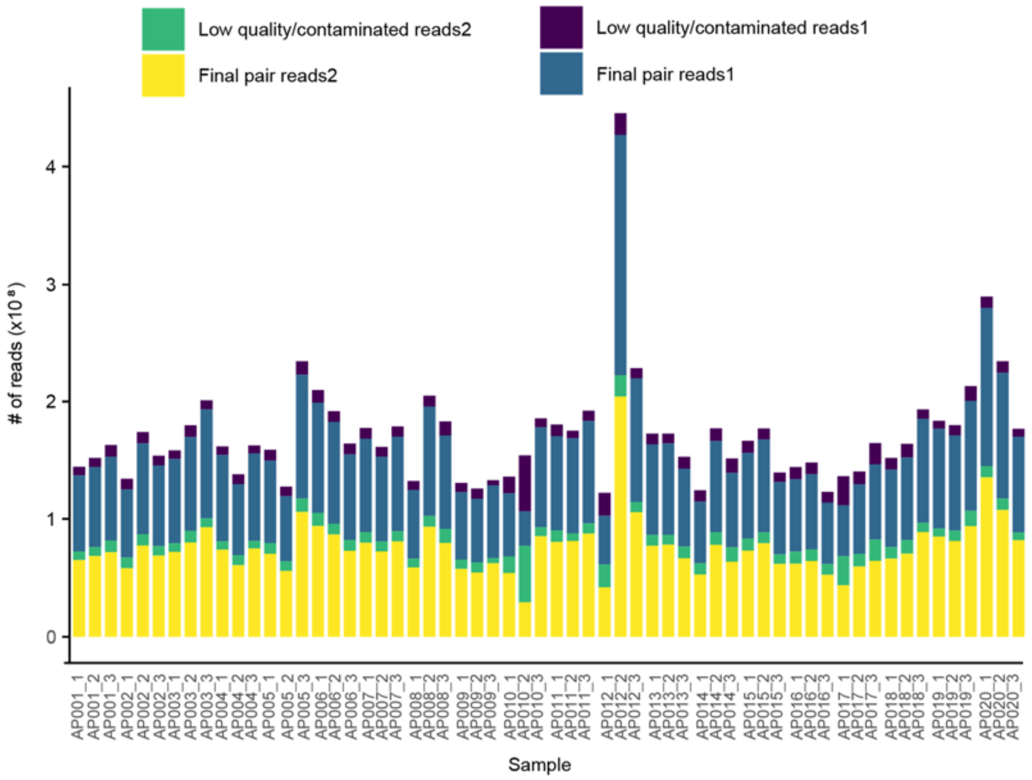

Figure S6. Quality control. We collected 60 fecal samples from AP patients at different phases of treatment. The green and purple indicates low quality and contaminated reads. The yellow and blue indicated high quality reads for microbial and functional annotation.

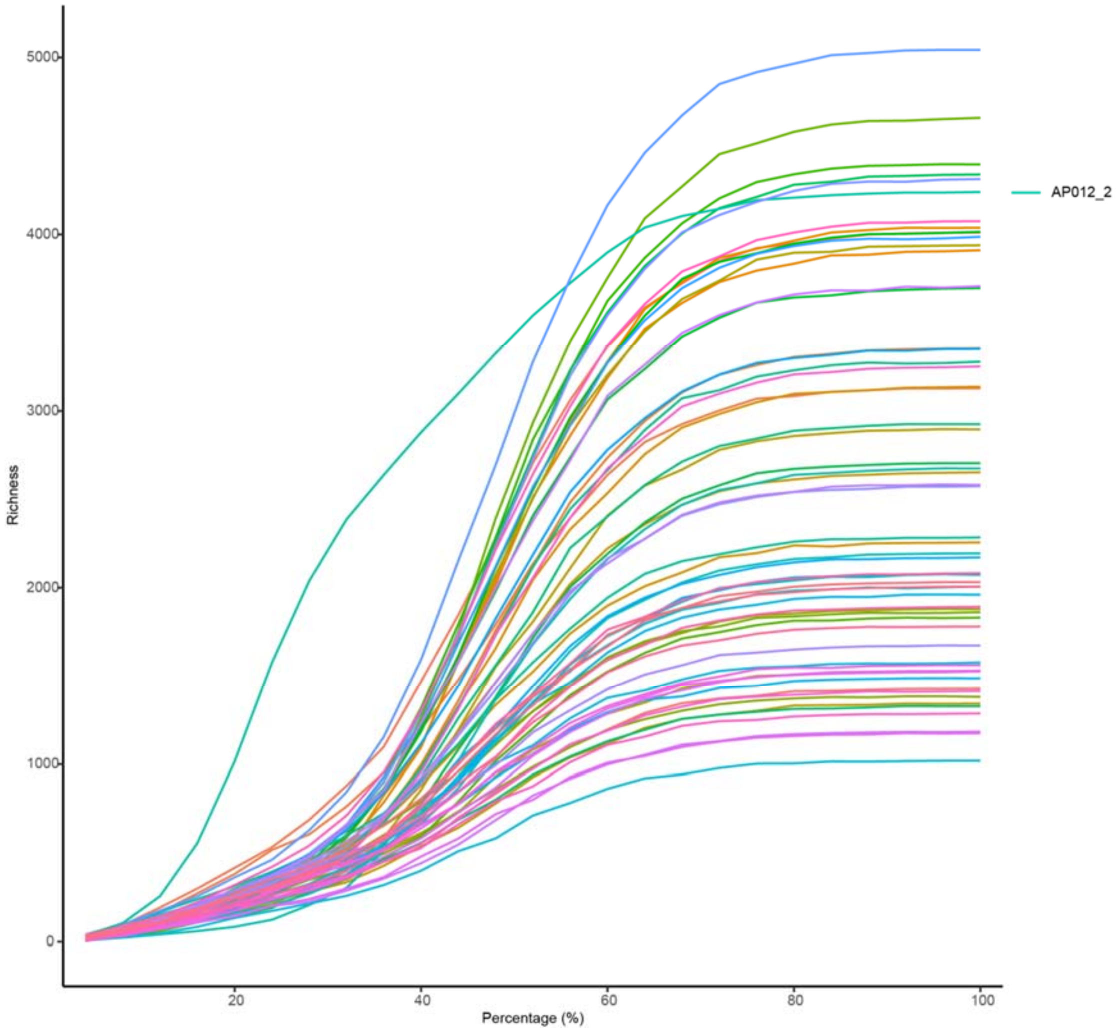

Figure S7. Rarefaction curve. Different colors represent different samples. The trend of the curve marked separately (AP012_2) is different.

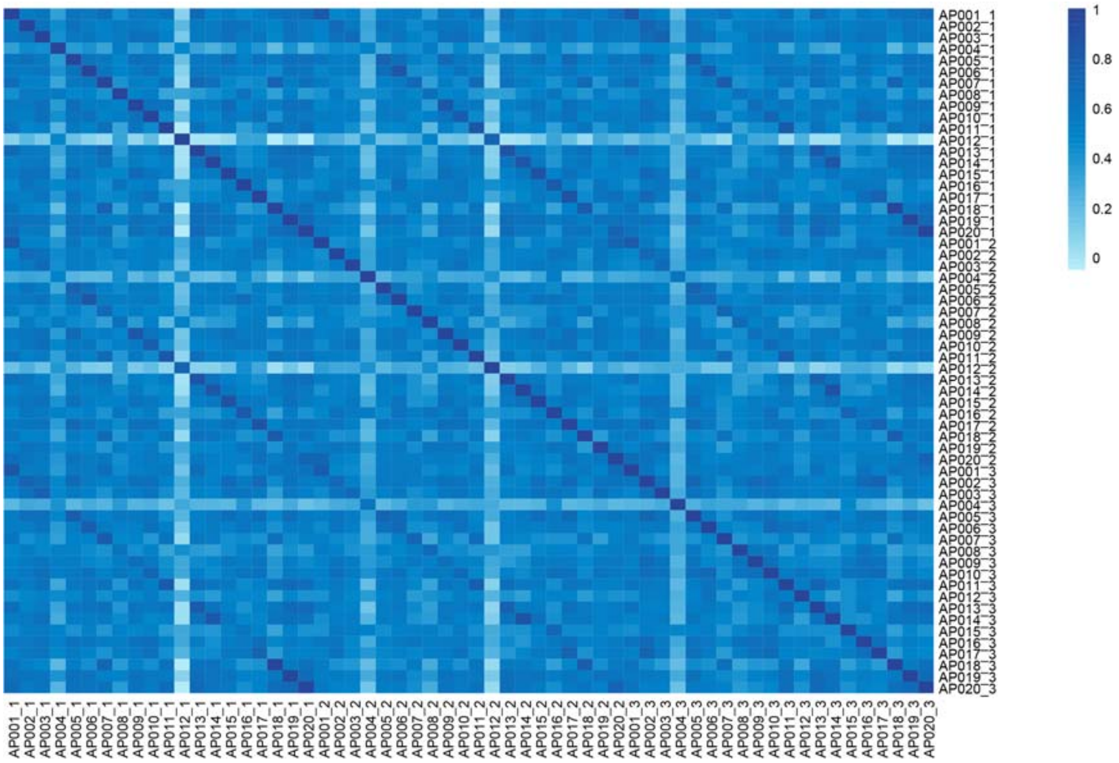

Figure S8. Spearman correlation of sample. Blue color from light to dark represents the correlation coefficient from small to large.

Table S1. Clinical information and biochemical indicators of patients with acute pancreatitis

Patient ID	MAP/SAP	P/HTG-A	Gender	Age	Treatment phase	WBC (10^9/L)(4-10)	ALB (g/L)(40-55)	CRP (mg/L)(0-10)	PCT (ng/ml)(0-0.5)	Fasting GLU (mmol/L)(3.9-6.1)	Ca (mmol/L)(2.11-2.52)
					Pre-treatment	5.21	44.4	9.4	0.05	8.2	2.31
AP001	MAP	BAP	Male	49	On-treatment	6.4	39	41	0.1	5.8	2.22
					Post-treatment	7.35	45	5.4	<0.05	4.5	2.35
					Pre-treatment	9.6	31.5	102	1.12	4.9	2.2
AP002	SAP	BAP	Male	48	On-treatment	12.54	25.6	128.2	8.89	8.9	1.98
					Post-treatment	7.22	38.9	12	<0.05	4.2	2.23
					Pre-treatment	11.7	31.2	10.2	0.89	6.5	2.16
AP003	MAP	BAP	Male	54	On-treatment	3.96	37.4	98.5	3.42	5.3	2.28
					Post-treatment	5.55	43.4	6.9	<0.05	5.6	2.88
					Pre-treatment	9.24	32.6	108.6	0.47	10.1	2.04
AP004	SAP	BAP	Female	49	On-treatment	14.1	32.1	235.9	4.35	9.9	1.4
					Post-treatment	3.07	45	34.2	<0.05	8.11	2.45
					Pre-treatment	9.58	40.8	45.1	0.07	8.3	2.23
AP005	MAP	HTG-AP	Male	48	On-treatment	11.04	41.2	98.2	0.19	6.7	2.2
					Post-treatment	5.78	52	9.8	<0.05	5.8	2.33
					Pre-treatment	3.58	37	85.5	2.64	8.6	2.06
AP006	MAP	HTG-AP	Male	36	On-treatment	8.97	34.7	83.3	0.05	5.3	1.78
					Post-treatment	5.9	41.2	10	<0.05	4.9	2.11
					Pre-treatment	7.45	37.6	47.9	2.59	4.4	2.1
AP007	SAP	BAP	Male	34	On-treatment	15.2	31.5	115.5	6.55	13.5	1.96
					Post-treatment	6.28	42	32.4	<0.05	3.9	2.45
					Pre-treatment	6.69	35.1	7	0.05	5.5	2.25
AP008	MAP	BAP	Female	48	On-treatment	5.62	40.4	42.1	0.05	4.9	2.2
					Post-treatment	5.77	43.2	2.1	<0.05	5.8	2.01
					Pre-treatment	15.12	45.8	60.5	0.05	7.8	2.42
AP009	MAP	BAP	Male	31	On-treatment	6.26	35.9	67.7	0.05	6.5	2.6
					Post-treatment	4.44	45.2	10.3	<0.05	6.9	2.52
					Pre-treatment	11.47	37.8	57.8	0.14	6.5	2.19
AP010	MAP	BAP	Male	38	On-treatment	7.19	36.9	71.4	0.11	5	2.15
					Post-treatment	5.99	43.9	8.9	<0.05	3.8	2.34
					Pre-treatment	10.16	41.2	49.3	0.05	9.3	2.27
AP011	MAP	HTG-AP	Male	41	On-treatment	9.22	43.2	56.3	3.34	6.7	2.02
					Post-treatment	8.9	44.5	7.8	<0.05	6.1	2.4
					Pre-treatment	11.53	32.9	7.22	0.05	7.3	2.12
AP012	SAP	HTG-AP	Female	47	On-treatment	17.71	28.9	161.2	10.12	17.7	1.72
					Post-treatment	10.8	39	34.2	<0.05	8.4	2.02
					Pre-treatment	8.53	37.1	101.2	0.59	7.3	2.04
AP013	MAP	BAP	Male	53	On-treatment	11.2	34.8	30.9	0.06	6.2	2.06
					Post-treatment	4.45	46.9	3.51	<0.05	4.9	2.3
					Pre-treatment	10.5	35.5	52.9	1.39	9.9	2.11
AP014	SAP	BAP	Male	86	On-treatment	22.3	31.6	112.4	8.38	15.4	1.88
					Post-treatment	3.87	38.9	32.1	<0.05	8.1	2.09
					Pre-treatment	11.05	44.9	74.2	0.09	6	2.35
AP015	SAP	HTG-AP	Male	32	On-treatment	16.72	30.8	195.2	12.24	12.1	1.67
					Post-treatment	10.1	38.3	12.1	0.89	6.8	2.55
					Pre-treatment	4.54	36.1	2.4	0.05	5.5	2.23
AP016	MAP	BAP	Male	60	On-treatment	3.95	40.5	40.9	1.05	4.7	2.18
					Post-treatment	5.98	45.9	3.4	<0.05	5.3	2.54
					Pre-treatment	8.27	40.9	20	0.05	8.4	2.05
AP017	MAP	BAP	Female	35	On-treatment	5.63	41.3	31.5	0.05	6.9	2.13
					Post-treatment	6.45	50	8	<0.05	3.9	2.21
					Pre-treatment	9.98	35.5	86.7	0.15	6	2.05
AP018	SAP	BAP	Male	67	On-treatment	13.54	34.2	117.4	4.15	4.6	1.9
					Post-treatment	6.89	43.9	21.2	<0.05	6.2	2.34
					Pre-treatment	15.57	41.7	21.4	0.07	5.7	2.1
AP019	MAP	BAP	Male	54	On-treatment	7.86	36.6	99.4	0.59	8.3	2.17
					Post-treatment	4.99	45.7	11.1	<0.05	4.5	2.4
					Pre-treatment	6.64	36.3	42.1	0.05	6.3	2.46
AP20	MAP	HTG-AP	Male	53	On-treatment	5.13	43.3	23.2	0.05	4.2	2.3
					Post-treatment	6.54	45.3	2.1	<0.05	5.88	2.54

MAP: mild acute pancreatitis, SAP: severe acute pancreatitis, BAP: biliary acute pancreatitis, HTG-AP: hypertriglyceridemia-induced acute pancreatitis, WBC: White

Table S2. Relative taxonomic abundances

ID	AP001_1	AP001_2	AP001_3	AP002_1	AP002_2	AP002_3
k__Bacteria	100	100	100	100	100	100
k__Bacteria|p__Actinobacteria	1.0178	1.63109	0.31028	0.06147	0.09439	1.00982
k__Bacteria|p__Actinobacteria|c__Actinobacteri	0.92979	1.38689	0.30128	0.00125	0.0341	0.0177
k__Bacteria|p__Actinobacteria|c__Actinobacteri	0.00481	0.06846	0.01383	0.00054	0.00826	0.00593
k__Bacteria|p__Actinobacteria|c__Actinobacteri	0.00481	0.06846	0.01383	0.00054	0.00826	0.00593
k__Bacteria|p__Actinobacteria|c__Actinobacteri	0	0	0	0	0	0
k__Bacteria|p__Actinobacteria|c__Actinobacteri	0	0	0	0	0	0
k__Bacteria|p__Actinobacteria|c__Actinobacteri	0.00481	0.06846	0.01383	0.00054	0.00826	0.00593
k__Bacteria|p__Actinobacteria|c__Actinobacteri	0	0	0	0	0	0
k__Bacteria|p__Actinobacteria|c__Actinobacteri	0	0	0.00011	0	0	0.00049
k__Bacteria|p__Actinobacteria|c__Actinobacteri	0	0	0	0	0	0
k__Bacteria|p__Actinobacteria|c__Actinobacteri	0	0	0	0	0	0
k__Bacteria|p__Actinobacteria|c__Actinobacteri	0	0	0	0	0	0
k__Bacteria|p__Actinobacteria|c__Actinobacteri	0	0	0	0	0	0
k__Bacteria|p__Actinobacteria|c__Actinobacteri	0.0019	0.02467	0.0066	0.00054	0.00464	0.00124
k__Bacteria|p__Actinobacteria|c__Actinobacteri	0	0	0	0	0	0
k__Bacteria|p__Actinobacteria|c__Actinobacteri	0.00055	0.0048	0.00165	0	0.00148	0.00024
k__Bacteria|p__Actinobacteria|c__Actinobacteri	0	0.00042	0.00038	0	0.00214	0
k__Bacteria|p__Actinobacteria|c__Actinobacteri	0.00236	0.00438	0.00468	0	0	0.00364
k__Bacteria|p__Actinobacteria|c__Actinobacteri	0	0.03378	0.00042	0	0	0
k__Bacteria|p__Actinobacteria|c__Actinobacteri	0	0	0	0	0	0
k__Bacteria|p__Actinobacteria|c__Actinobacteri	0	0.00043	0	0	0	0.00032
k__Bacteria|p__Actinobacteria|c__Actinobacteri	0	0	0	0	0	0
k__Bacteria|p__Actinobacteria|c__Actinobacteri	0	0	0	0	0	0
k__Bacteria|p__Actinobacteria|c__Actinobacteri	0	0	0	0	0	0
k__Bacteria|p__Actinobacteria|c__Actinobacteri	0	0	0	0	0	0
k__Bacteria|p__Actinobacteria|c__Actinobacteri	0.92385	1.30272	0.28263	0	0	0.00843
k__Bacteria|p__Actinobacteria|c__Actinobacteri	0.92385	1.30272	0.28263	0	0	0.00843
k__Bacteria|p__Actinobacteria|c__Actinobacteri	0	0	0	0	0	0
k__Bacteria|p__Actinobacteria|c__Actinobacteri	0	0	0	0	0	0
k__Bacteria|p__Actinobacteria|c__Actinobacteri	0	0	0	0	0	0
k__Bacteria|p__Actinobacteria|c__Actinobacteri	0	0	0	0	0	0
k__Bacteria|p__Actinobacteria|c__Actinobacteri	0.92385	1.30272	0.28263	0	0	0.00843

k__Bacteria|p__Actinobacteria|c__Actinobacteri	0.47385	0.32392	0.07188	0	0	0
k__Bacteria|p__Actinobacteria|c__Actinobacteri	0	0	0	0	0	0
k__Bacteria|p__Actinobacteria|c__Actinobacteri	0	0	0	0	0	0
k__Bacteria|p__Actinobacteria|c__Actinobacteri	0	0	0	0	0	0
k__Bacteria|p__Actinobacteria|c__Actinobacteri	0	0	0.00022	0	0	0.00843
k__Bacteria|p__Actinobacteria|c__Actinobacteri	0.45	0.9788	0.21054	0	0	0
k__Bacteria|p__Actinobacteria|c__Actinobacteri	0	0	0	0	0	0
k__Bacteria|p__Actinobacteria|c__Actinobacteri	0	0	0	0	0	0
k__Bacteria|p__Actinobacteria|c__Actinobacteri	0	0	0	0	0	0
k__Bacteria|p__Actinobacteria|c__Actinobacteri	0	0	0	0	0	0
k__Bacteria|p__Actinobacteria|c__Actinobacteri	0	0	0	0	0	0
k__Bacteria|p__Actinobacteria|c__Actinobacteri	0	0	0	0	0	0
k__Bacteria|p__Actinobacteria|c__Actinobacteri	0	0	0	0	0	0
k__Bacteria|p__Actinobacteria|c__Actinobacteri	0	0	0	0	0	0
k__Bacteria|p__Actinobacteria|c__Actinobacteri	0	0	0	0	0	0
k__Bacteria|p__Actinobacteria|c__Actinobacteri	0	0	0	0	0	0
k__Bacteria|p__Actinobacteria|c__Actinobacteri	0	0	0	0	0	0
k__Bacteria|p__Actinobacteria|c__Actinobacteri	0.00112	0.01571	0.00482	0.00071	0.02584	0.00334
k__Bacteria|p__Actinobacteria|c__Actinobacteri	0.00112	0.01571	0.00482	0.00071	0.02584	0.00334
k__Bacteria|p__Actinobacteria|c__Actinobacteri	0.00112	0.01571	0.00482	0.00071	0.02584	0.00334
k__Bacteria|p__Actinobacteria|c__Actinobacteri	0	0	0	0	0	0
k__Bacteria|p__Actinobacteria|c__Actinobacteri	0	0	0	0.00013	0	0.00076
k__Bacteria|p__Actinobacteria|c__Actinobacteri	0.00112	0.01571	0.00482	0.00058	0.02584	0.00258
k__Bacteria|p__Actinobacteria|c__Actinobacteri	0	0	0	0	0	0
k__Bacteria|p__Actinobacteria|c__Actinobacteri	0	0	0	0	0	0
k__Bacteria|p__Actinobacteria|c__Actinobacteri	0	0	0	0	0	0
k__Bacteria|p__Actinobacteria|c__Actinobacteri	0	0	0	0	0	0
k__Bacteria|p__Actinobacteria|c__Actinobacteri	0	0	0	0	0	0
k__Bacteria|p__Actinobacteria|c__Actinobacteri	0	0	0	0	0	0
k__Bacteria|p__Actinobacteria|c__Coriobacteriia	0.08801	0.2442	0.009	0.06022	0.06029	0.99211
k__Bacteria|p__Actinobacteria|c__Coriobacteriia	0.04425	0.19548	0.0024	0.02522	0.05735	0.96267
k__Bacteria|p__Actinobacteria|c__Coriobacteriia	0.00249	0.12558	0	0	0.00027	0
k__Bacteria|p__Actinobacteria|c__Coriobacteriia	0.00249	0.12434	0	0	0	0
k__Bacteria|p__Actinobacteria|c__Coriobacteriia	0	0	0	0	0	0
k__Bacteria|p__Actinobacteria|c__Coriobacteriia	0	0.05898	0	0	0	0

k__Bacteria|p__Actinobacteria|c__Coriobacterii	0.00249	0.06536	0	0	0	0
k__Bacteria|p__Actinobacteria|c__Coriobacterii	0	0.00124	0	0	0.00027	0
k__Bacteria|p__Actinobacteria|c__Coriobacterii	0	0	0	0	0	0
k__Bacteria|p__Actinobacteria|c__Coriobacterii	0	0.00124	0	0	0.00027	0
k__Bacteria|p__Actinobacteria|c__Coriobacterii	0.04176	0.06991	0.0024	0.02522	0.05708	0.96267
k__Bacteria|p__Actinobacteria|c__Coriobacterii	0	0	0	0.02522	0.05708	0.96249
k__Bacteria|p__Actinobacteria|c__Coriobacterii	0	0	0	0.02287	0.05708	0.61722
k__Bacteria|p__Actinobacteria|c__Coriobacterii	0	0	0	0.00235	0	0.32828
k__Bacteria|p__Actinobacteria|c__Coriobacterii	0	0	0	0	0	0.017
k__Bacteria|p__Actinobacteria|c__Coriobacterii	0.04176	0.06991	0.0024	0	0	0.00018
k__Bacteria|p__Actinobacteria|c__Coriobacterii	0.00037	0.00049	0	0	0	0.00018
k__Bacteria|p__Actinobacteria|c__Coriobacterii	0.0414	0.06941	0.0024	0	0	0
k__Bacteria|p__Actinobacteria|c__Coriobacterii	0.04376	0.04871	0.0066	0.035	0.00294	0.02944
k__Bacteria|p__Actinobacteria|c__Coriobacterii	0.04376	0.04871	0.0066	0.035	0.00294	0.02944
k__Bacteria|p__Actinobacteria|c__Coriobacterii	0.0156	0.01318	0.00343	0	0	0
k__Bacteria|p__Actinobacteria|c__Coriobacterii	0.0156	0.01318	0.00343	0	0	0
k__Bacteria|p__Actinobacteria|c__Coriobacterii	0.00457	0.00395	0.00089	0	0	0
k__Bacteria|p__Actinobacteria|c__Coriobacterii	0.00457	0.00395	0.00089	0	0	0
k__Bacteria|p__Actinobacteria|c__Coriobacterii	0.02116	0.02805	0.00174	0.03258	0.00294	0.02605
k__Bacteria|p__Actinobacteria|c__Coriobacterii	0.02116	0.02805	0.00174	0.03258	0.00294	0.02605
k__Bacteria|p__Actinobacteria|c__Coriobacterii	0	0	0	0	0	0
k__Bacteria|p__Actinobacteria|c__Coriobacterii	0	0	0	0	0	0
k__Bacteria|p__Actinobacteria|c__Coriobacterii	0.00242	0.00354	0.00054	0.00242	0	0.00339
k__Bacteria|p__Actinobacteria|c__Coriobacterii	0.00242	0.00354	0.00054	0.00242	0	0.00339
k__Bacteria|p__Actinobacteria|c__Coriobacterii	0	0	0	0	0	0
k__Bacteria|p__Actinobacteria|c__Coriobacterii	0	0	0	0	0	0
k__Bacteria|p__Bacteroidetes	69.06482	71.12769	62.43203	84.3306	92.42681	78.47789
k__Bacteria|p__Bacteroidetes|c__Bacteroidetes	0	0	0	0	0	0
k__Bacteria|p__Bacteroidetes|c__Bacteroidetes	0	0	0	0	0	0
k__Bacteria|p__Bacteroidetes|c__Bacteroidetes	0	0	0	0	0	0
k__Bacteria|p__Bacteroidetes|c__Bacteroidetes	0	0	0	0	0	0
k__Bacteria|p__Bacteroidetes|c__Bacteroidetes	0	0	0	0	0	0
k__Bacteria|p__Bacteroidetes|c__Bacteroidia	69.06482	71.12502	62.43203	84.3306	92.42681	78.47789
k__Bacteria|p__Bacteroidetes|c__Bacteroidia|o_	69.06482	71.12502	62.43203	84.3306	92.42681	78.47789
k__Bacteria|p__Bacteroidetes|c__Bacteroidia|o_	65.52159	62.53354	52.40902	68.41289	91.58155	73.22866

k__Bacteria|p__Bacteroidetes|c__Bacteroidia|o_	65.52159	62.53354	52.40902	68.41289	91.58155	73.22866
k__Bacteria|p__Bacteroidetes|c__Bacteroidia|o_	0	0	0	4.76631	0.00067	3.52483
k__Bacteria|p__Bacteroidetes|c__Bacteroidia|o_	0.07055	0.06637	0.84079	0	0	0
k__Bacteria|p__Bacteroidetes|c__Bacteroidia|o_	0	0	0	0	0	0
k__Bacteria|p__Bacteroidetes|c__Bacteroidia|o_	0	0	0.00013	10.95925	4.69375	5.34075
k__Bacteria|p__Bacteroidetes|c__Bacteroidia|o_	0	0	0	0	0	0
k__Bacteria|p__Bacteroidetes|c__Bacteroidia|o_	0	0	0	0	0	0
k__Bacteria|p__Bacteroidetes|c__Bacteroidia|o_	0	0	0	0	0	0
k__Bacteria|p__Bacteroidetes|c__Bacteroidia|o_	0	0	0	0	0	0
k__Bacteria|p__Bacteroidetes|c__Bacteroidia|o_	2.57876	11.7188	1.36661	3.19685	17.11976	2.34669
k__Bacteria|p__Bacteroidetes|c__Bacteroidia|o_	1.25308	1.55895	6.84114	0	0	0
k__Bacteria|p__Bacteroidetes|c__Bacteroidia|o_	0.03647	0.10933	0.09733	0	0	0
k__Bacteria|p__Bacteroidetes|c__Bacteroidia|o_	0.00054	0.00227	0.0022	0	0	0
k__Bacteria|p__Bacteroidetes|c__Bacteroidia|o_	0.61042	0.43879	0.697	0	0.00285	0.01304
k__Bacteria|p__Bacteroidetes|c__Bacteroidia|o_	0	0	0	0	0	0.00139
k__Bacteria|p__Bacteroidetes|c__Bacteroidia|o_	0	0	0	1.30528	1.74245	2.36337
k__Bacteria|p__Bacteroidetes|c__Bacteroidia|o_	0.00695	0.00452	0.00657	0	0	0
k__Bacteria|p__Bacteroidetes|c__Bacteroidia|o_	0	0	0	0	0	0
k__Bacteria|p__Bacteroidetes|c__Bacteroidia|o_	0	0	0	8.81947	54.89933	27.03738
k__Bacteria|p__Bacteroidetes|c__Bacteroidia|o_	1.2834	1.6254	3.62751	7.02992	2.81265	1.6856
k__Bacteria|p__Bacteroidetes|c__Bacteroidia|o_	20.83861	11.98095	5.97262	0.36424	9.01714	20.76674
k__Bacteria|p__Bacteroidetes|c__Bacteroidia|o_	31.09047	31.67915	20.96144	31.23713	1.16303	8.50519
k__Bacteria|p__Bacteroidetes|c__Bacteroidia|o_	7.75234	3.34901	11.99567	0.73445	0.12992	1.64368
k__Bacteria|p__Bacteroidetes|c__Bacteroidia|o_	0	0	0	0	0	0
k__Bacteria|p__Bacteroidetes|c__Bacteroidia|o_	0	0	0	0	0	0
k__Bacteria|p__Bacteroidetes|c__Bacteroidia|o_	0	0	0	0	0	0
k__Bacteria|p__Bacteroidetes|c__Bacteroidia|o_	0	0	0	0	0	0
k__Bacteria|p__Bacteroidetes|c__Bacteroidia|o_	0	0	0	0	0	0
k__Bacteria|p__Bacteroidetes|c__Bacteroidia|o_	0	0	0	0	0	0
k__Bacteria|p__Bacteroidetes|c__Bacteroidia|o_	1.08608	3.74507	1.37593	0.76686	0	0.1942
k__Bacteria|p__Bacteroidetes|c__Bacteroidia|o_	1.08107	3.67735	1.3672	0.76686	0	0.1942
k__Bacteria|p__Bacteroidetes|c__Bacteroidia|o_	0.00069	0.00448	0.00234	0	0	0
k__Bacteria|p__Bacteroidetes|c__Bacteroidia|o_	1.08039	3.67288	1.36486	0.76686	0	0.1942
k__Bacteria|p__Bacteroidetes|c__Bacteroidia|o_	0.005	0.06771	0.00873	0	0	0
k__Bacteria|p__Bacteroidetes|c__Bacteroidia|o_	0.005	0.06771	0.00873	0	0	0

k__Bacteria|p__Bacteroidetes|c__Bacteroidia|o_	0	0	0	0	0	0
k__Bacteria|p__Bacteroidetes|c__Bacteroidia|o_	0	0.07594	0	0	0	0
k__Bacteria|p__Bacteroidetes|c__Bacteroidia|o_	0	0.07594	0	0	0	0
k__Bacteria|p__Bacteroidetes|c__Bacteroidia|o_	0	0.03723	0	0	0	0
k__Bacteria|p__Bacteroidetes|c__Bacteroidia|o_	0	0.03871	0	0	0	0
k__Bacteria|p__Bacteroidetes|c__Bacteroidia|o_	0	0	0	0	0	0
k__Bacteria|p__Bacteroidetes|c__Bacteroidia|o_	0	0	0	0	0	0
k__Bacteria|p__Bacteroidetes|c__Bacteroidia|o_	0	0	0	0	0	0
k__Bacteria|p__Bacteroidetes|c__Bacteroidia|o_	1.32989	1.13637	7.27821	0.02157	0	0.00058
k__Bacteria|p__Bacteroidetes|c__Bacteroidia|o_	0	0.01243	0	0	0	0
k__Bacteria|p__Bacteroidetes|c__Bacteroidia|o_	0	0.01243	0	0	0	0
k__Bacteria|p__Bacteroidetes|c__Bacteroidia|o_	0	0	0	0	0	0
k__Bacteria|p__Bacteroidetes|c__Bacteroidia|o_	0	0	0	0	0	0
k__Bacteria|p__Bacteroidetes|c__Bacteroidia|o_	0	0	0	0	0	0
k__Bacteria|p__Bacteroidetes|c__Bacteroidia|o_	1.32989	1.12394	7.27821	0.02157	0	0.00058
k__Bacteria|p__Bacteroidetes|c__Bacteroidia|o_	0	0.00321	0	0.02157	0	0.00058
k__Bacteria|p__Bacteroidetes|c__Bacteroidia|o_	0	0	0	0	0	0
k__Bacteria|p__Bacteroidetes|c__Bacteroidia|o_	0	0	0	0	0	0
k__Bacteria|p__Bacteroidetes|c__Bacteroidia|o_	0	0	0	0	0	0
k__Bacteria|p__Bacteroidetes|c__Bacteroidia|o_	0	0	0	0	0	0
k__Bacteria|p__Bacteroidetes|c__Bacteroidia|o_	0	0.00688	0	0	0	0
k__Bacteria|p__Bacteroidetes|c__Bacteroidia|o_	0	0	0	0	0	0
k__Bacteria|p__Bacteroidetes|c__Bacteroidia|o_	0	0.01595	0	0	0	0
k__Bacteria|p__Bacteroidetes|c__Bacteroidia|o_	0	0.00065	0	0	0	0
k__Bacteria|p__Bacteroidetes|c__Bacteroidia|o_	0.00822	0	0	0	0	0
k__Bacteria|p__Bacteroidetes|c__Bacteroidia|o_	0	0.00128	0	0	0	0
k__Bacteria|p__Bacteroidetes|c__Bacteroidia|o_	0	0.06206	0	0	0	0
k__Bacteria|p__Bacteroidetes|c__Bacteroidia|o_	0	0.00247	0	0	0	0
k__Bacteria|p__Bacteroidetes|c__Bacteroidia|o_	0	0.00065	0	0	0	0
k__Bacteria|p__Bacteroidetes|c__Bacteroidia|o_	1.32167	1.03078	7.27821	0	0	0
k__Bacteria|p__Bacteroidetes|c__Bacteroidia|o_	0	0	0	0	0	0
k__Bacteria|p__Bacteroidetes|c__Bacteroidia|o_	0	0	0	0	0	0
k__Bacteria|p__Bacteroidetes|c__Bacteroidia|o_	0	0	0	0	0	0
k__Bacteria|p__Bacteroidetes|c__Bacteroidia|o_	0.36988	0.66871	0.3632	2.50586	0.08461	1.47227
k__Bacteria|p__Bacteroidetes|c__Bacteroidia|o_	0.36988	0.66871	0.3632	2.50586	0.08461	1.47227

k__Bacteria|p__Bacteroidetes|c__Bacteroidia|o_	0	0	0	2.42028	0.08299	1.45829
k__Bacteria|p__Bacteroidetes|c__Bacteroidia|o_	0.04285	0.12586	0.03336	0.01407	0	0
k__Bacteria|p__Bacteroidetes|c__Bacteroidia|o_	0	0	0	0	0	0
k__Bacteria|p__Bacteroidetes|c__Bacteroidia|o_	0	0	0	0	0	0
k__Bacteria|p__Bacteroidetes|c__Bacteroidia|o_	0.32703	0.54284	0.32985	0.07151	0.00162	0.01398
k__Bacteria|p__Bacteroidetes|c__Bacteroidia|o_	0	0	0	0	0	0
k__Bacteria|p__Bacteroidetes|c__Bacteroidia|o_	0.75739	2.9654	1.00567	12.62342	0.76065	3.58219
k__Bacteria|p__Bacteroidetes|c__Bacteroidia|o_	0.75739	2.9654	1.00567	12.62342	0.76065	3.58219
k__Bacteria|p__Bacteroidetes|c__Bacteroidia|o_	0.65855	1.50481	0.81444	6.66688	0.36844	2.44804
k__Bacteria|p__Bacteroidetes|c__Bacteroidia|o_	0.08092	1.40067	0.16842	1.27208	0.00088	0.32617
k__Bacteria|p__Bacteroidetes|c__Bacteroidia|o_	0.01306	0.04702	0.01554	0	0	0
k__Bacteria|p__Bacteroidetes|c__Bacteroidia|o_	0.00458	0.0129	0.00727	0.13575	0	0.22213
k__Bacteria|p__Bacteroidetes|c__Bacteroidia|o_	0.00027	0	0	4.54871	0.39133	0.58585
k__Bacteria|p__Bacteroidetes|c__Bacteroidia|o_	0	0	0	0	0	0
k__Bacteria|p__Bacteroidetes|c__Flavobacteriia	0	0.00267	0	0	0	0
k__Bacteria|p__Bacteroidetes|c__Flavobacteriia	0	0.00267	0	0	0	0
k__Bacteria|p__Bacteroidetes|c__Flavobacteriia	0	0.00267	0	0	0	0
k__Bacteria|p__Bacteroidetes|c__Flavobacteriia	0	0.00267	0	0	0	0
k__Bacteria|p__Bacteroidetes|c__Flavobacteriia	0	0.00057	0	0	0	0
k__Bacteria|p__Bacteroidetes|c__Flavobacteriia	0	0.0021	0	0	0	0
k__Bacteria|p__Firmicutes	26.49054	21.11724	30.98224	5.74117	2.0467	12.12343
k__Bacteria|p__Firmicutes|c__Bacilli	0.0304	2.58949	0.60771	0.1929	0.45135	0.59093
k__Bacteria|p__Firmicutes|c__Bacilli|o__Bacilla	0.00051	0.38802	0.00404	0.00346	0	0.00662
k__Bacteria|p__Firmicutes|c__Bacilli|o__Bacilla	0.00051	0.38802	0.00404	0.00346	0	0.00662
k__Bacteria|p__Firmicutes|c__Bacilli|o__Bacilla	0.00051	0.38802	0.00404	0.00346	0	0.00662
k__Bacteria|p__Firmicutes|c__Bacilli|o__Bacilla	0	0	0	0	0	0
k__Bacteria|p__Firmicutes|c__Bacilli|o__Bacilla	0	0.05097	0.00093	0.00227	0	0
k__Bacteria|p__Firmicutes|c__Bacilli|o__Bacilla	0	0.28677	0.00024	0	0	0.00133
k__Bacteria|p__Firmicutes|c__Bacilli|o__Bacilla	0.00051	0.05028	0.00287	0.00119	0	0.00528
k__Bacteria|p__Firmicutes|c__Bacilli|o__Lactob	0.02989	2.20147	0.60368	0.18944	0.45135	0.58431
k__Bacteria|p__Firmicutes|c__Bacilli|o__Lactob	0.00036	0.02516	0.00132	0	0	0
k__Bacteria|p__Firmicutes|c__Bacilli|o__Lactob	0.00036	0.02516	0.00132	0	0	0
k__Bacteria|p__Firmicutes|c__Bacilli|o__Lactob	0	0.01291	0	0	0	0
k__Bacteria|p__Firmicutes|c__Bacilli|o__Lactob	0.00036	0.01225	0.00132	0	0	0
k__Bacteria|p__Firmicutes|c__Bacilli|o__Lactob	0	0.32419	0	0	0.00023	0

k__Bacteria|p__Firmicutes|c__Bacilli|o__Lactob	0	0.32419	0	0	0.00023	0
k__Bacteria|p__Firmicutes|c__Bacilli|o__Lactob	0	0.00841	0	0	0.00023	0
k__Bacteria|p__Firmicutes|c__Bacilli|o__Lactob	0	0.31578	0	0	0	0
k__Bacteria|p__Firmicutes|c__Bacilli|o__Lactob	0	0	0	0.00432	0.20625	0
k__Bacteria|p__Firmicutes|c__Bacilli|o__Lactob	0	0	0	0.00432	0.20625	0
k__Bacteria|p__Firmicutes|c__Bacilli|o__Lactob	0	0	0	0	0	0
k__Bacteria|p__Firmicutes|c__Bacilli|o__Lactob	0	0	0	0.00112	0.00038	0
k__Bacteria|p__Firmicutes|c__Bacilli|o__Lactob	0	0	0	0	0	0
k__Bacteria|p__Firmicutes|c__Bacilli|o__Lactob	0	0	0	0	0	0
k__Bacteria|p__Firmicutes|c__Bacilli|o__Lactob	0	0	0	0	0.20586	0
k__Bacteria|p__Firmicutes|c__Bacilli|o__Lactob	0	0	0	0.00321	0	0
k__Bacteria|p__Firmicutes|c__Bacilli|o__Lactob	0	0	0	0	0	0
k__Bacteria|p__Firmicutes|c__Bacilli|o__Lactob	0	0	0	0	0	0
k__Bacteria|p__Firmicutes|c__Bacilli|o__Lactob	0	0	0	0	0	0
k__Bacteria|p__Firmicutes|c__Bacilli|o__Lactob	0	0	0	0	0	0
k__Bacteria|p__Firmicutes|c__Bacilli|o__Lactob	0	0	0	0.00708	0	0
k__Bacteria|p__Firmicutes|c__Bacilli|o__Lactob	0	0	0	0.00708	0	0
k__Bacteria|p__Firmicutes|c__Bacilli|o__Lactob	0	0	0	0	0	0
k__Bacteria|p__Firmicutes|c__Bacilli|o__Lactob	0	0	0	0	0	0
k__Bacteria|p__Firmicutes|c__Bacilli|o__Lactob	0	0	0	0	0	0
k__Bacteria|p__Firmicutes|c__Bacilli|o__Lactob	0	0	0	0	0	0
k__Bacteria|p__Firmicutes|c__Bacilli|o__Lactob	0	0	0	0	0	0
k__Bacteria|p__Firmicutes|c__Bacilli|o__Lactob	0	0	0	0	0	0
k__Bacteria|p__Firmicutes|c__Bacilli|o__Lactob	0	0	0	0	0	0
k__Bacteria|p__Firmicutes|c__Bacilli|o__Lactob	0	0	0	0	0	0
k__Bacteria|p__Firmicutes|c__Bacilli|o__Lactob	0	0	0	0.00708	0	0
k__Bacteria|p__Firmicutes|c__Bacilli|o__Lactob	0	0	0.01652	0	0	0
k__Bacteria|p__Firmicutes|c__Bacilli|o__Lactob	0	0	0	0	0	0
k__Bacteria|p__Firmicutes|c__Bacilli|o__Lactob	0	0	0	0	0	0
k__Bacteria|p__Firmicutes|c__Bacilli|o__Lactob	0	0	0	0	0	0
k__Bacteria|p__Firmicutes|c__Bacilli|o__Lactob	0	0	0.01652	0	0	0
k__Bacteria|p__Firmicutes|c__Bacilli|o__Lactob	0	0	0.00133	0	0	0
k__Bacteria|p__Firmicutes|c__Bacilli|o__Lactob	0	0	0.01519	0	0	0
k__Bacteria|p__Firmicutes|c__Bacilli|o__Lactob	0	0	0	0	0	0
k__Bacteria|p__Firmicutes|c__Bacilli|o__Lactob	0.02954	1.85212	0.58584	0.17804	0.24487	0.58431

k__Bacteria|p__Firmicutes|c__Bacilli|o__Lactob	0.02954	1.85212	0.58584	0.17804	0.24487	0.58431
k__Bacteria|p__Firmicutes|c__Bacilli|o__Lactob	0	0.02617	0.03664	0.00296	0.00044	0.00752
k__Bacteria|p__Firmicutes|c__Bacilli|o__Lactob	0	0.01798	0.05161	0	0.01251	0.02554
k__Bacteria|p__Firmicutes|c__Bacilli|o__Lactob	0	0.00043	0.00134	0.00151	0.00117	0.037
k__Bacteria|p__Firmicutes|c__Bacilli|o__Lactob	0	0	0	0	0	0
k__Bacteria|p__Firmicutes|c__Bacilli|o__Lactob	0	0.03468	0.05066	0.01335	0	0.01972
k__Bacteria|p__Firmicutes|c__Bacilli|o__Lactob	0.00057	0.07498	0.01847	0.00176	0.03136	0.02643
k__Bacteria|p__Firmicutes|c__Bacilli|o__Lactob	0	0	0.06337	0	0	0
k__Bacteria|p__Firmicutes|c__Bacilli|o__Lactob	0	0	0	0	0.0006	0
k__Bacteria|p__Firmicutes|c__Bacilli|o__Lactob	0.00777	0.19259	0.0171	0.03996	0	0.01502
k__Bacteria|p__Firmicutes|c__Bacilli|o__Lactob	0	0	0	0	0	0
k__Bacteria|p__Firmicutes|c__Bacilli|o__Lactob	0	0.10008	0.01892	0.02507	0.06875	0.03561
k__Bacteria|p__Firmicutes|c__Bacilli|o__Lactob	0.00593	1.18467	0.16111	0.03545	0.12821	0.30228
k__Bacteria|p__Firmicutes|c__Bacilli|o__Lactob	0	0	0	0	0	0
k__Bacteria|p__Firmicutes|c__Bacilli|o__Lactob	0	0	0	0	0.00045	0.0012
k__Bacteria|p__Firmicutes|c__Bacilli|o__Lactob	0.01499	0.17323	0.15471	0.05797	0	0.114
k__Bacteria|p__Firmicutes|c__Bacilli|o__Lactob	0	0.00244	0.00487	0	0	0
k__Bacteria|p__Firmicutes|c__Bacilli|o__Lactob	0	0	0	0	0	0
k__Bacteria|p__Firmicutes|c__Bacilli|o__Lactob	0.00027	0.00127	0.00055	0	0.00138	0
k__Bacteria|p__Firmicutes|c__Bacilli|o__Lactob	0	0.00036	0	0	0	0
k__Bacteria|p__Firmicutes|c__Bacilli|o__Lactob	0	0.00224	0	0	0	0
k__Bacteria|p__Firmicutes|c__Bacilli|o__Lactob	0	0.00227	0	0	0	0
k__Bacteria|p__Firmicutes|c__Bacilli|o__Lactob	0	0.0013	0	0	0	0
k__Bacteria|p__Firmicutes|c__Bacilli|o__Lactob	0	0	0	0	0	0
k__Bacteria|p__Firmicutes|c__Bacilli|o__Lactob	0	0	0	0	0	0
k__Bacteria|p__Firmicutes|c__Bacilli|o__Lactob	0	0.03743	0	0	0	0
k__Bacteria|p__Firmicutes|c__Bacilli|o__Lactob	0	0	0	0	0	0
k__Bacteria|p__Firmicutes|c__Bacilli|o__Lactob	0	0	0.00649	0	0	0
k__Bacteria|p__Firmicutes|c__Bacilli|o__Lactob	0	0	0	0	0	0
k__Bacteria|p__Firmicutes|c__Clostridia	22.73505	14.56305	28.29254	4.99099	1.09365	6.63884
k__Bacteria|p__Firmicutes|c__Clostridia|o__Clo	22.73505	14.56305	28.29254	4.99099	1.09365	6.63884
k__Bacteria|p__Firmicutes|c__Clostridia|o__Clo	0.00385	0.01887	0.00574	0.0013	0	0
k__Bacteria|p__Firmicutes|c__Clostridia|o__Clo	0.00385	0.01887	0.00574	0.0013	0	0
k__Bacteria|p__Firmicutes|c__Clostridia|o__Clo	0.00385	0.01887	0.00574	0.0013	0	0
k__Bacteria|p__Firmicutes|c__Clostridia|o__Clo	0.00033	0.00226	0.00297	0.00476	0	0

k__Bacteria|p__Firmicutes|c__Clostridia|o__Clo	0.00033	0.00226	0.00297	0.00476	0	0
k__Bacteria|p__Firmicutes|c__Clostridia|o__Clo	0.00033	0.00226	0.00297	0.00476	0	0
k__Bacteria|p__Firmicutes|c__Clostridia|o__Clo	0.04018	1.57171	0.01974	0.20414	0.20112	0.10401
k__Bacteria|p__Firmicutes|c__Clostridia|o__Clo	0	0	0	0	0	0
k__Bacteria|p__Firmicutes|c__Clostridia|o__Clo	0	0	0	0	0	0
k__Bacteria|p__Firmicutes|c__Clostridia|o__Clo	0.02575	0	0	0.09771	0.0495	0.05059
k__Bacteria|p__Firmicutes|c__Clostridia|o__Clo	0	0	0	0	0	0
k__Bacteria|p__Firmicutes|c__Clostridia|o__Clo	0.02575	0	0	0.09322	0.0495	0
k__Bacteria|p__Firmicutes|c__Clostridia|o__Clo	0	0	0	0	0	0
k__Bacteria|p__Firmicutes|c__Clostridia|o__Clo	0	0	0	0	0	0
k__Bacteria|p__Firmicutes|c__Clostridia|o__Clo	0	0	0	0	0	0
k__Bacteria|p__Firmicutes|c__Clostridia|o__Clo	0	0	0	0	0	0
k__Bacteria|p__Firmicutes|c__Clostridia|o__Clo	0	0	0	0	0	0
k__Bacteria|p__Firmicutes|c__Clostridia|o__Clo	0	0	0	0	0	0
k__Bacteria|p__Firmicutes|c__Clostridia|o__Clo	0	0	0	0.00449	0	0
k__Bacteria|p__Firmicutes|c__Clostridia|o__Clo	0	0	0	0	0	0
k__Bacteria|p__Firmicutes|c__Clostridia|o__Clo	0	0	0	0	0	0
k__Bacteria|p__Firmicutes|c__Clostridia|o__Clo	0	0	0	0	0	0
k__Bacteria|p__Firmicutes|c__Clostridia|o__Clo	0	0	0	0	0	0
k__Bacteria|p__Firmicutes|c__Clostridia|o__Clo	0	0	0	0	0	0.00268
k__Bacteria|p__Firmicutes|c__Clostridia|o__Clo	0	0	0	0	0	0.04791
k__Bacteria|p__Firmicutes|c__Clostridia|o__Clo	0.01443	1.57171	0.01974	0.10643	0.15162	0.05342
k__Bacteria|p__Firmicutes|c__Clostridia|o__Clo	0	0	0	0	0	0
k__Bacteria|p__Firmicutes|c__Clostridia|o__Clo	0.01443	1.57171	0.01974	0.10643	0.15162	0.05342
k__Bacteria|p__Firmicutes|c__Clostridia|o__Clo	0	0	0	0	0	0
k__Bacteria|p__Firmicutes|c__Clostridia|o__Clo	0	0	0	0	0	0
k__Bacteria|p__Firmicutes|c__Clostridia|o__Clo	0	0.00786	0.00122	0	0	0.00044
k__Bacteria|p__Firmicutes|c__Clostridia|o__Clo	0	0.00281	0	0	0	0
k__Bacteria|p__Firmicutes|c__Clostridia|o__Clo	0	0	0	0	0	0
k__Bacteria|p__Firmicutes|c__Clostridia|o__Clo	0	0.00019	0	0	0	0
k__Bacteria|p__Firmicutes|c__Clostridia|o__Clo	0	0	0	0	0	0
k__Bacteria|p__Firmicutes|c__Clostridia|o__Clo	0	0.00262	0	0	0	0
k__Bacteria|p__Firmicutes|c__Clostridia|o__Clo	0	0.00505	0.00122	0	0	0.00044
k__Bacteria|p__Firmicutes|c__Clostridia|o__Clo	0	0.00505	0.00122	0	0	0.00044
k__Bacteria|p__Firmicutes|c__Clostridia|o__Clo	0.1096	0.08018	0.19816	0	0	0

k__Bacteria|p__Firmicutes|c__Clostridia|o__Clo	0	0	0	0	0	0
k__Bacteria|p__Firmicutes|c__Clostridia|o__Clo	0	0	0	0	0	0
k__Bacteria|p__Firmicutes|c__Clostridia|o__Clo	0.1096	0.08018	0.19816	0	0	0
k__Bacteria|p__Firmicutes|c__Clostridia|o__Clo	0.1096	0.08018	0.19816	0	0	0
k__Bacteria|p__Firmicutes|c__Clostridia|o__Clo	0	0	0	0	0	0
k__Bacteria|p__Firmicutes|c__Clostridia|o__Clo	0	0	0	0	0	0
k__Bacteria|p__Firmicutes|c__Clostridia|o__Clo	0	0	0	0	0	0
k__Bacteria|p__Firmicutes|c__Clostridia|o__Clo	0	0	0	0	0	0
k__Bacteria|p__Firmicutes|c__Clostridia|o__Clo	8.25376	1.7351	8.77646	0.00957	0	0.37596
k__Bacteria|p__Firmicutes|c__Clostridia|o__Clo	0	0	0	0	0	0
k__Bacteria|p__Firmicutes|c__Clostridia|o__Clo	0	0	0	0	0	0
k__Bacteria|p__Firmicutes|c__Clostridia|o__Clo	0	0	0	0	0	0
k__Bacteria|p__Firmicutes|c__Clostridia|o__Clo	0	0	0	0	0	0
k__Bacteria|p__Firmicutes|c__Clostridia|o__Clo	8.25376	1.7351	8.77646	0.00957	0	0.37596
k__Bacteria|p__Firmicutes|c__Clostridia|o__Clo	0	0	0	0	0	0
k__Bacteria|p__Firmicutes|c__Clostridia|o__Clo	0	0	0	0	0	0
k__Bacteria|p__Firmicutes|c__Clostridia|o__Clo	4.20063	0.60738	7.48267	0	0	0
k__Bacteria|p__Firmicutes|c__Clostridia|o__Clo	0.27303	0.16012	0.04779	0	0	0.12917
k__Bacteria|p__Firmicutes|c__Clostridia|o__Clo	0	0.00025	0	0.00957	0	0
k__Bacteria|p__Firmicutes|c__Clostridia|o__Clo	0.05605	0.27755	0.02168	0	0	0
k__Bacteria|p__Firmicutes|c__Clostridia|o__Clo	0	0	0	0	0	0
k__Bacteria|p__Firmicutes|c__Clostridia|o__Clo	0	0	0	0	0	0
k__Bacteria|p__Firmicutes|c__Clostridia|o__Clo	0	0	0	0	0	0
k__Bacteria|p__Firmicutes|c__Clostridia|o__Clo	3.72405	0.6898	1.22432	0	0	0.24679
k__Bacteria|p__Firmicutes|c__Clostridia|o__Clo	0	0	0	0	0	0
k__Bacteria|p__Firmicutes|c__Clostridia|o__Clo	0	0	0	0	0	0
k__Bacteria|p__Firmicutes|c__Clostridia|o__Clo	0	0	0	0	0	0
k__Bacteria|p__Firmicutes|c__Clostridia|o__Clo	0	0	0	0	0	0
k__Bacteria|p__Firmicutes|c__Clostridia|o__Clo	7.87242	5.17828	12.42379	3.6052	0.45932	5.19227
k__Bacteria|p__Firmicutes|c__Clostridia|o__Clo	0.35876	0.62558	0.11718	0	0.01908	0.04637
k__Bacteria|p__Firmicutes|c__Clostridia|o__Clo	0	0	0	0	0	0
k__Bacteria|p__Firmicutes|c__Clostridia|o__Clo	0.35876	0.62558	0.11718	0	0.01908	0.04637
k__Bacteria|p__Firmicutes|c__Clostridia|o__Clo	0	0	0	0	0	0
k__Bacteria|p__Firmicutes|c__Clostridia|o__Clo	0	0	0	0	0	0
k__Bacteria|p__Firmicutes|c__Clostridia|o__Clo	0.82335	0.48417	0.7242	2.55968	0.28249	4.03838

k__Bacteria|p__Firmicutes|c__Clostridia|o__Clo	0	0	0	0	0	0.00928
k__Bacteria|p__Firmicutes|c__Clostridia|o__Clo	0	0	0	0	0	0
k__Bacteria|p__Firmicutes|c__Clostridia|o__Clo	0	0	0	0	0	0
k__Bacteria|p__Firmicutes|c__Clostridia|o__Clo	0.60059	0.28876	0.19588	0	0	0
k__Bacteria|p__Firmicutes|c__Clostridia|o__Clo	0	0	0	0	0	0.00179
k__Bacteria|p__Firmicutes|c__Clostridia|o__Clo	0	0	0	0	0.00317	0.02617
k__Bacteria|p__Firmicutes|c__Clostridia|o__Clo	0.11455	0.05872	0.50652	0	0.05651	0.57198
k__Bacteria|p__Firmicutes|c__Clostridia|o__Clo	0.00067	0.02561	0.00063	2.55968	0.22281	3.42917
k__Bacteria|p__Firmicutes|c__Clostridia|o__Clo	0.10753	0.11108	0.02117	0	0	0
k__Bacteria|p__Firmicutes|c__Clostridia|o__Clo	0	0	0	0	0	0
k__Bacteria|p__Firmicutes|c__Clostridia|o__Clo	0	0	0	0	0	0
k__Bacteria|p__Firmicutes|c__Clostridia|o__Clo	0	0	0	0	0	0
k__Bacteria|p__Firmicutes|c__Clostridia|o__Clo	0	0	0	0	0	0
k__Bacteria|p__Firmicutes|c__Clostridia|o__Clo	0	0	0	0	0	0
k__Bacteria|p__Firmicutes|c__Clostridia|o__Clo	0.29644	0.31587	0.1951	0.00264	0.01312	0.06997
k__Bacteria|p__Firmicutes|c__Clostridia|o__Clo	0.13347	0.1633	0.07618	0.00264	0.01312	0.06997
k__Bacteria|p__Firmicutes|c__Clostridia|o__Clo	0.16298	0.15257	0.11892	0	0	0
k__Bacteria|p__Firmicutes|c__Clostridia|o__Clo	0	0	0	0	0	0
k__Bacteria|p__Firmicutes|c__Clostridia|o__Clo	0.00692	0.02428	0.0022	0.03146	0	0.03708
k__Bacteria|p__Firmicutes|c__Clostridia|o__Clo	0.00692	0.02428	0.0022	0	0	0
k__Bacteria|p__Firmicutes|c__Clostridia|o__Clo	0	0	0	0.03146	0	0.03708
k__Bacteria|p__Firmicutes|c__Clostridia|o__Clo	0	0	0	0	0	0
k__Bacteria|p__Firmicutes|c__Clostridia|o__Clo	0	0	0	0	0	0
k__Bacteria|p__Firmicutes|c__Clostridia|o__Clo	0.56524	0.23254	0.78822	0	0	0
k__Bacteria|p__Firmicutes|c__Clostridia|o__Clo	0.56524	0.23254	0.78822	0	0	0
k__Bacteria|p__Firmicutes|c__Clostridia|o__Clo	0	0	0	0	0	0
k__Bacteria|p__Firmicutes|c__Clostridia|o__Clo	0	0	0	0	0	0
k__Bacteria|p__Firmicutes|c__Clostridia|o__Clo	0.17639	0.41612	0.13196	0.6167	0.14151	0.5618
k__Bacteria|p__Firmicutes|c__Clostridia|o__Clo	0.04268	0.08901	0.01828	0.0045	0.00202	0.02869
k__Bacteria|p__Firmicutes|c__Clostridia|o__Clo	0	0	0	0.00928	0.00915	0.10714
k__Bacteria|p__Firmicutes|c__Clostridia|o__Clo	0.01743	0.03759	0.00812	0.36559	0.10936	0.09813
k__Bacteria|p__Firmicutes|c__Clostridia|o__Clo	0.05344	0.10535	0.0361	0.00523	0	0.12684
k__Bacteria|p__Firmicutes|c__Clostridia|o__Clo	0.00025	0.00136	0.00687	0	0	0.03158
k__Bacteria|p__Firmicutes|c__Clostridia|o__Clo	0	0	0	0	0	0
k__Bacteria|p__Firmicutes|c__Clostridia|o__Clo	0.0484	0.0646	0.02753	0	0	0

k__Bacteria|p__Firmicutes|c__Clostridia|o__Clo	0	0	0	0	0	0
k__Bacteria|p__Firmicutes|c__Clostridia|o__Clo	0	0	0	0	0	0
k__Bacteria|p__Firmicutes|c__Clostridia|o__Clo	0.01419	0.11822	0.03506	0.2321	0.02098	0.16943
k__Bacteria|p__Firmicutes|c__Clostridia|o__Clo	0	0	0	0	0	0
k__Bacteria|p__Firmicutes|c__Clostridia|o__Clo	0	0	0	0	0	0
k__Bacteria|p__Firmicutes|c__Clostridia|o__Clo	0	0	0	0	0	0
k__Bacteria|p__Firmicutes|c__Clostridia|o__Clo	3.26056	2.16232	6.18004	0.02558	0	0.29348
k__Bacteria|p__Firmicutes|c__Clostridia|o__Clo	3.26056	2.16232	6.18004	0	0	0
k__Bacteria|p__Firmicutes|c__Clostridia|o__Clo	0	0	0	0.02558	0	0.29348
k__Bacteria|p__Firmicutes|c__Clostridia|o__Clo	0	0	0	0	0	0
k__Bacteria|p__Firmicutes|c__Clostridia|o__Clo	0	0	0	0	0	0
k__Bacteria|p__Firmicutes|c__Clostridia|o__Clo	0	0.00096	0.00333	0	0	0
k__Bacteria|p__Firmicutes|c__Clostridia|o__Clo	0	0.00096	0.00333	0	0	0
k__Bacteria|p__Firmicutes|c__Clostridia|o__Clo	0	0.00198	0	0	0	0
k__Bacteria|p__Firmicutes|c__Clostridia|o__Clo	0	0.00198	0	0	0	0
k__Bacteria|p__Firmicutes|c__Clostridia|o__Clo	0	0.00441	0	0	0	0
k__Bacteria|p__Firmicutes|c__Clostridia|o__Clo	0	0.00441	0	0	0	0
k__Bacteria|p__Firmicutes|c__Clostridia|o__Clo	0	0	0	0	0	0
k__Bacteria|p__Firmicutes|c__Clostridia|o__Clo	0	0	0	0	0	0
k__Bacteria|p__Firmicutes|c__Clostridia|o__Clo	0	0	0	0	0	0
k__Bacteria|p__Firmicutes|c__Clostridia|o__Clo	2.38476	0.90455	4.28155	0	0	0.00438
k__Bacteria|p__Firmicutes|c__Clostridia|o__Clo	1.94239	0.81374	0	0	0	0
k__Bacteria|p__Firmicutes|c__Clostridia|o__Clo	0.37102	0.06459	1.77762	0	0	0
k__Bacteria|p__Firmicutes|c__Clostridia|o__Clo	0.01697	0.00158	0	0	0	0.00415
k__Bacteria|p__Firmicutes|c__Clostridia|o__Clo	0.03395	0.01486	2.39218	0	0	0.00022
k__Bacteria|p__Firmicutes|c__Clostridia|o__Clo	0.02043	0.00977	0.11175	0	0	0
k__Bacteria|p__Firmicutes|c__Clostridia|o__Clo	0	0	0	0	0	0
k__Bacteria|p__Firmicutes|c__Clostridia|o__Clo	0	0.00248	0	0	0	0
k__Bacteria|p__Firmicutes|c__Clostridia|o__Clo	0	0.00248	0	0	0	0
k__Bacteria|p__Firmicutes|c__Clostridia|o__Clo	0	0.00302	0	0	0	0
k__Bacteria|p__Firmicutes|c__Clostridia|o__Clo	0	0.00302	0	0	0	0
k__Bacteria|p__Firmicutes|c__Clostridia|o__Clo	0	0	0	0	0	0
k__Bacteria|p__Firmicutes|c__Clostridia|o__Clo	0	0	0	0	0	0
k__Bacteria|p__Firmicutes|c__Clostridia|o__Clo	0	0	0	0.36914	0.00311	0.14082
k__Bacteria|p__Firmicutes|c__Clostridia|o__Clo	0	0	0	0.36914	0.00311	0.14082

k__Bacteria|p__Firmicutes|c__Clostridia|o__Clo	0	0	0	0	0	0
k__Bacteria|p__Firmicutes|c__Clostridia|o__Clo	0	0	0	0	0	0
k__Bacteria|p__Firmicutes|c__Clostridia|o__Clo	0	0	0	0	0	0
k__Bacteria|p__Firmicutes|c__Clostridia|o__Clo	0	0	0	0	0	0
k__Bacteria|p__Firmicutes|c__Clostridia|o__Clo	0.00565	0.20091	0.01722	0.15703	0.17524	0.00558
k__Bacteria|p__Firmicutes|c__Clostridia|o__Clo	0	0	0	0.15703	0.17524	0
k__Bacteria|p__Firmicutes|c__Clostridia|o__Clo	0	0	0	0.15703	0.17524	0
k__Bacteria|p__Firmicutes|c__Clostridia|o__Clo	0	0	0	0	0	0
k__Bacteria|p__Firmicutes|c__Clostridia|o__Clo	0	0	0	0	0	0
k__Bacteria|p__Firmicutes|c__Clostridia|o__Clo	0.00553	0.03739	0.01722	0	0	0.00558
k__Bacteria|p__Firmicutes|c__Clostridia|o__Clo	0.00553	0.03739	0.01722	0	0	0.00558
k__Bacteria|p__Firmicutes|c__Clostridia|o__Clo	0	0	0	0	0	0
k__Bacteria|p__Firmicutes|c__Clostridia|o__Clo	0	0	0	0	0	0
k__Bacteria|p__Firmicutes|c__Clostridia|o__Clo	0	0	0	0	0	0
k__Bacteria|p__Firmicutes|c__Clostridia|o__Clo	0	0	0	0	0	0
k__Bacteria|p__Firmicutes|c__Clostridia|o__Clo	0	0.16352	0	0	0	0
k__Bacteria|p__Firmicutes|c__Clostridia|o__Clo	0	0	0	0	0	0
k__Bacteria|p__Firmicutes|c__Clostridia|o__Clo	0	0.16352	0	0	0	0
k__Bacteria|p__Firmicutes|c__Clostridia|o__Clo	0.00012	0	0	0	0	0
k__Bacteria|p__Firmicutes|c__Clostridia|o__Clo	0.00012	0	0	0	0	0
k__Bacteria|p__Firmicutes|c__Clostridia|o__Clo	0	0	0	0	0	0
k__Bacteria|p__Firmicutes|c__Clostridia|o__Clo	0	0	0	0	0	0
k__Bacteria|p__Firmicutes|c__Clostridia|o__Clo	6.44926	5.76787	6.84726	1.00899	0.25797	0.96059
k__Bacteria|p__Firmicutes|c__Clostridia|o__Clo	0.30831	0.05988	0.04471	0	0.14446	0.13761
k__Bacteria|p__Firmicutes|c__Clostridia|o__Clo	0.30831	0.05988	0.04471	0	0.14446	0.13761
k__Bacteria|p__Firmicutes|c__Clostridia|o__Clo	0	0	0	0	0	0
k__Bacteria|p__Firmicutes|c__Clostridia|o__Clo	0	0	0	0	0	0
k__Bacteria|p__Firmicutes|c__Clostridia|o__Clo	0	0	0	0	0	0
k__Bacteria|p__Firmicutes|c__Clostridia|o__Clo	0	0	0	0	0	0
k__Bacteria|p__Firmicutes|c__Clostridia|o__Clo	0.05811	0.32041	0.04941	0.13367	0	0.01361
k__Bacteria|p__Firmicutes|c__Clostridia|o__Clo	0.05811	0.32041	0.04941	0.13367	0	0.01361
k__Bacteria|p__Firmicutes|c__Clostridia|o__Clo	4.10309	3.48609	4.54088	0	0.00015	0.00063
k__Bacteria|p__Firmicutes|c__Clostridia|o__Clo	4.10309	3.48609	4.54088	0	0.00015	0.00063
k__Bacteria|p__Firmicutes|c__Clostridia|o__Clo	1.19444	1.2889	0.13982	0.62395	0.10858	0.73271
k__Bacteria|p__Firmicutes|c__Clostridia|o__Clo	1.19444	1.2889	0.13982	0.62395	0.10858	0.73271

k__Bacteria|p__Firmicutes|c__Clostridia|o__Clo	0	0	0	0	0	0
k__Bacteria|p__Firmicutes|c__Clostridia|o__Clo	0.22015	0.06403	0.0963	0	0	0
k__Bacteria|p__Firmicutes|c__Clostridia|o__Clo	0.22015	0.06403	0.0963	0	0	0
k__Bacteria|p__Firmicutes|c__Clostridia|o__Clo	0	0	0	0	0	0
k__Bacteria|p__Firmicutes|c__Clostridia|o__Clo	0	0	0	0	0	0
k__Bacteria|p__Firmicutes|c__Clostridia|o__Clo	0	0	0	0	0	0
k__Bacteria|p__Firmicutes|c__Clostridia|o__Clo	0	0	0	0	0	0
k__Bacteria|p__Firmicutes|c__Clostridia|o__Clo	0	0	0	0	0	0
k__Bacteria|p__Firmicutes|c__Clostridia|o__Clo	0	0	0	0	0	0
k__Bacteria|p__Firmicutes|c__Clostridia|o__Clo	0	0	0	0	0	0
k__Bacteria|p__Firmicutes|c__Clostridia|o__Clo	0	0	0	0	0	0
k__Bacteria|p__Firmicutes|c__Clostridia|o__Clo	0	0	0	0	0	0
k__Bacteria|p__Firmicutes|c__Clostridia|o__Clo	0	0	0	0	0	0
k__Bacteria|p__Firmicutes|c__Clostridia|o__Clo	0	0	0	0	0	0
k__Bacteria|p__Firmicutes|c__Clostridia|o__Clo	0.41559	0.25855	1.86267	0	0	0
k__Bacteria|p__Firmicutes|c__Clostridia|o__Clo	0.14275	0.06379	0.49843	0	0	0
k__Bacteria|p__Firmicutes|c__Clostridia|o__Clo	0.27283	0.19476	1.36424	0	0	0
k__Bacteria|p__Firmicutes|c__Clostridia|o__Clo	0	0	0	0	0	0
k__Bacteria|p__Firmicutes|c__Clostridia|o__Clo	0	0	0	0	0	0
k__Bacteria|p__Firmicutes|c__Clostridia|o__Clo	0	0	0	0	0	0
k__Bacteria|p__Firmicutes|c__Clostridia|o__Clo	0.14957	0.29001	0.11348	0.25137	0.00478	0.07603
k__Bacteria|p__Firmicutes|c__Clostridia|o__Clo	0.14957	0.29001	0.11348	0.25137	0.00478	0.07603
k__Bacteria|p__Firmicutes|c__Erysipelotrichia	0.00847	0.19442	0.00852	0.19858	0.00768	0.07299
k__Bacteria|p__Firmicutes|c__Erysipelotrichia|o	0.00847	0.19442	0.00852	0.19858	0.00768	0.07299
k__Bacteria|p__Firmicutes|c__Erysipelotrichia|o	0.00847	0.19442	0.00852	0.19858	0.00768	0.07299
k__Bacteria|p__Firmicutes|c__Erysipelotrichia|o	0	0	0	0	0	0
k__Bacteria|p__Firmicutes|c__Erysipelotrichia|o	0	0	0	0	0	0
k__Bacteria|p__Firmicutes|c__Erysipelotrichia|o	0	0.0149	0	0	0	0
k__Bacteria|p__Firmicutes|c__Erysipelotrichia|o	0	0.0149	0	0	0	0
k__Bacteria|p__Firmicutes|c__Erysipelotrichia|o	0	0	0	0	0	0
k__Bacteria|p__Firmicutes|c__Erysipelotrichia|o	0	0	0	0	0	0
k__Bacteria|p__Firmicutes|c__Erysipelotrichia|o	0	0.12064	0	0.10029	0	0
k__Bacteria|p__Firmicutes|c__Erysipelotrichia|o	0	0.12064	0	0.10029	0	0
k__Bacteria|p__Firmicutes|c__Erysipelotrichia|o	0	0	0	0	0	0
k__Bacteria|p__Firmicutes|c__Erysipelotrichia|o	0	0	0	0	0	0

k__Bacteria|p__Firmicutes|c__Erysipelotrichia|o	0	0.00408	0	0	0	0
k__Bacteria|p__Firmicutes|c__Erysipelotrichia|o	0	0.00408	0	0	0	0
k__Bacteria|p__Firmicutes|c__Erysipelotrichia|o	0.00847	0.01965	0.00852	0.09109	0.00768	0.07299
k__Bacteria|p__Firmicutes|c__Erysipelotrichia|o	0.00418	0.01397	0.00205	0.08782	0	0
k__Bacteria|p__Firmicutes|c__Erysipelotrichia|o	0	0	0	0	0	0
k__Bacteria|p__Firmicutes|c__Erysipelotrichia|o	0.00429	0.00569	0.00646	0.00327	0.00768	0.07299
k__Bacteria|p__Firmicutes|c__Erysipelotrichia|o	0	0	0	0	0	0
k__Bacteria|p__Firmicutes|c__Erysipelotrichia|o	0	0	0	0	0	0
k__Bacteria|p__Firmicutes|c__Erysipelotrichia|o	0	0	0	0	0	0
k__Bacteria|p__Firmicutes|c__Erysipelotrichia|o	0	0	0	0	0	0
k__Bacteria|p__Firmicutes|c__Erysipelotrichia|o	0	0	0	0.0072	0	0
k__Bacteria|p__Firmicutes|c__Erysipelotrichia|o	0	0	0	0.0072	0	0
k__Bacteria|p__Firmicutes|c__Erysipelotrichia|o	0	0	0	0	0	0
k__Bacteria|p__Firmicutes|c__Erysipelotrichia|o	0	0	0	0	0	0
k__Bacteria|p__Firmicutes|c__Erysipelotrichia|o	0	0	0	0	0	0
k__Bacteria|p__Firmicutes|c__Erysipelotrichia|o	0	0	0	0	0	0
k__Bacteria|p__Firmicutes|c__Erysipelotrichia|o	0	0	0	0	0	0
k__Bacteria|p__Firmicutes|c__Erysipelotrichia|o	0	0	0	0	0	0
k__Bacteria|p__Firmicutes|c__Erysipelotrichia|o	0	0.03514	0	0	0	0
k__Bacteria|p__Firmicutes|c__Erysipelotrichia|o	0	0.03514	0	0	0	0
k__Bacteria|p__Firmicutes|c__Erysipelotrichia|o	0	0	0	0	0	0
k__Bacteria|p__Firmicutes|c__Erysipelotrichia|o	0	0	0	0	0	0
k__Bacteria|p__Firmicutes|c__Firmicutes_uncla	0.20216	0.60806	0.1288	0	0	0
k__Bacteria|p__Firmicutes|c__Firmicutes_uncla	0.20216	0.60806	0.1288	0	0	0
k__Bacteria|p__Firmicutes|c__Firmicutes_uncla	0.20216	0.60806	0.1288	0	0	0
k__Bacteria|p__Firmicutes|c__Firmicutes_uncla	0.20216	0.60806	0.1288	0	0	0
k__Bacteria|p__Firmicutes|c__Firmicutes_uncla	0	0	0	0	0	0
k__Bacteria|p__Firmicutes|c__Firmicutes_uncla	0.20189	0.60806	0.1288	0	0	0
k__Bacteria|p__Firmicutes|c__Firmicutes_uncla	0	0	0	0	0	0
k__Bacteria|p__Firmicutes|c__Firmicutes_uncla	0	0	0	0	0	0
k__Bacteria|p__Firmicutes|c__Firmicutes_uncla	0.00026	0	0	0	0	0
k__Bacteria|p__Firmicutes|c__Firmicutes_uncla	0	0	0	0	0	0
k__Bacteria|p__Firmicutes|c__Firmicutes_uncla	0	0	0	0	0	0
k__Bacteria|p__Firmicutes|c__Firmicutes_uncla	0	0	0	0	0	0
k__Bacteria|p__Firmicutes|c__Negativicutes	3.49481	2.87252	1.94459	0.35836	0.49403	4.81767

k__Bacteria|p__Firmicutes|c__Negativicutes|o_	0.29226	0.54783	1.46748	0.31548	0.47471	0.66947
k__Bacteria|p__Firmicutes|c__Negativicutes|o_	0.29226	0.54783	1.46748	0.31548	0.47471	0.66947
k__Bacteria|p__Firmicutes|c__Negativicutes|o_	0	0	0	0	0	0
k__Bacteria|p__Firmicutes|c__Negativicutes|o_	0	0	0	0	0	0
k__Bacteria|p__Firmicutes|c__Negativicutes|o_	0	0	0	0	0	0
k__Bacteria|p__Firmicutes|c__Negativicutes|o_	0.29226	0.54783	1.46748	0.31548	0.47471	0.66947
k__Bacteria|p__Firmicutes|c__Negativicutes|o_	0.29226	0.54783	1.46748	0.31548	0.47471	0.66947
k__Bacteria|p__Firmicutes|c__Negativicutes|o_	0	0	0	0	0	0
k__Bacteria|p__Firmicutes|c__Negativicutes|o_	0	0	0	0	0	3.4574
k__Bacteria|p__Firmicutes|c__Negativicutes|o_	0	0	0	0	0	3.4574
k__Bacteria|p__Firmicutes|c__Negativicutes|o_	0	0	0	0	0	3.4574
k__Bacteria|p__Firmicutes|c__Negativicutes|o_	0	0	0	0	0	3.14255
k__Bacteria|p__Firmicutes|c__Negativicutes|o_	0	0	0	0	0	0.31486
k__Bacteria|p__Firmicutes|c__Negativicutes|o_	0	0	0	0	0	0
k__Bacteria|p__Firmicutes|c__Negativicutes|o_	0	0	0	0	0	0
k__Bacteria|p__Firmicutes|c__Negativicutes|o_	0	0	0	0	0	0
k__Bacteria|p__Firmicutes|c__Negativicutes|o_	0	0	0	0	0	0
k__Bacteria|p__Firmicutes|c__Negativicutes|o_	0	0	0	0	0	0
k__Bacteria|p__Firmicutes|c__Negativicutes|o_	0	0	0	0	0	0
k__Bacteria|p__Firmicutes|c__Negativicutes|o_	0	0	0	0	0	0
k__Bacteria|p__Firmicutes|c__Negativicutes|o_	3.20255	2.32469	0.47711	0.04288	0.01932	0.6908
k__Bacteria|p__Firmicutes|c__Negativicutes|o_	3.20255	2.32469	0.47711	0.04288	0.01932	0.6908
k__Bacteria|p__Firmicutes|c__Negativicutes|o_	0.00273	0.00173	0	0	0	0
k__Bacteria|p__Firmicutes|c__Negativicutes|o_	0.00273	0.00173	0	0	0	0
k__Bacteria|p__Firmicutes|c__Negativicutes|o_	0	0.00738	0	0	0	0.00271
k__Bacteria|p__Firmicutes|c__Negativicutes|o_	0	0.00738	0	0	0	0.00271
k__Bacteria|p__Firmicutes|c__Negativicutes|o_	2.75157	1.55142	0.22509	0	0	0.00053
k__Bacteria|p__Firmicutes|c__Negativicutes|o_	0	0.00094	0	0	0	0.00013
k__Bacteria|p__Firmicutes|c__Negativicutes|o_	0.00076	0.09856	0	0	0	0.00041
k__Bacteria|p__Firmicutes|c__Negativicutes|o_	2.75082	1.45192	0.22509	0	0	0
k__Bacteria|p__Firmicutes|c__Negativicutes|o_	0.44659	0.51826	0.18325	0	0	0.03722
k__Bacteria|p__Firmicutes|c__Negativicutes|o_	0.44659	0.49946	0.18325	0	0	0
k__Bacteria|p__Firmicutes|c__Negativicutes|o_	0	0	0	0	0	0
k__Bacteria|p__Firmicutes|c__Negativicutes|o_	0	0.0188	0	0	0	0.03722
k__Bacteria|p__Firmicutes|c__Negativicutes|o_	0	0	0	0	0	0

k__Bacteria|p__Firmicutes|c__Negativicutes|o_	0	0	0	0	0	0
k__Bacteria|p__Firmicutes|c__Negativicutes|o_	0.00166	0.2459	0.06878	0.04288	0.01932	0.65034
k__Bacteria|p__Firmicutes|c__Negativicutes|o_	0.00046	0.15683	0.03069	0	0	0.21049
k__Bacteria|p__Firmicutes|c__Negativicutes|o_	0.00121	0.00876	0.00103	0	0	0.08437
k__Bacteria|p__Firmicutes|c__Negativicutes|o_	0	0.01011	0.00127	0	0	0.00526
k__Bacteria|p__Firmicutes|c__Negativicutes|o_	0	0.04849	0.02747	0.04288	0.01932	0.24781
k__Bacteria|p__Firmicutes|c__Negativicutes|o_	0	0	0	0	0	0
k__Bacteria|p__Firmicutes|c__Negativicutes|o_	0	0	0	0	0	0
k__Bacteria|p__Firmicutes|c__Negativicutes|o_	0	0.00754	0.00157	0	0	0.06982
k__Bacteria|p__Firmicutes|c__Negativicutes|o_	0	0.01418	0.00674	0	0	0.0326
k__Bacteria|p__Firmicutes|c__Tissierellia	0.01965	0.2897	0.00008	0.00035	0	0.003
k__Bacteria|p__Firmicutes|c__Tissierellia|o__Ti	0.01965	0.2897	0.00008	0.00035	0	0.003
k__Bacteria|p__Firmicutes|c__Tissierellia|o__Ti	0.01965	0.2897	0.00008	0.00035	0	0.003
k__Bacteria|p__Firmicutes|c__Tissierellia|o__Ti	0	0	0	0	0	0
k__Bacteria|p__Firmicutes|c__Tissierellia|o__Ti	0	0	0	0	0	0
k__Bacteria|p__Firmicutes|c__Tissierellia|o__Ti	0	0	0	0	0	0
k__Bacteria|p__Firmicutes|c__Tissierellia|o__Ti	0	0	0	0	0	0
k__Bacteria|p__Firmicutes|c__Tissierellia|o__Ti	0	0	0	0	0	0
k__Bacteria|p__Firmicutes|c__Tissierellia|o__Ti	0.01965	0.2897	0.00008	0.00035	0	0.003
k__Bacteria|p__Firmicutes|c__Tissierellia|o__Ti	0.01965	0.28828	0.00008	0.00035	0	0.003
k__Bacteria|p__Firmicutes|c__Tissierellia|o__Ti	0	0.00141	0	0	0	0
k__Bacteria|p__Firmicutes|c__Tissierellia|o__Ti	0	0	0	0	0	0
k__Bacteria|p__Firmicutes|c__Tissierellia|o__Ti	0	0	0	0	0	0
k__Bacteria|p__Firmicutes|c__Tissierellia|o__Ti	0	0	0	0	0	0
k__Bacteria|p__Firmicutes|c__Tissierellia|o__Ti	0	0	0	0	0	0
k__Bacteria|p__Fusobacteria	0.0186	0.20614	0.00265	0.89493	0.07642	3.72314
k__Bacteria|p__Fusobacteria|c__Fusobacteriia	0.0186	0.20614	0.00265	0.89493	0.07642	3.72314
k__Bacteria|p__Fusobacteria|c__Fusobacteriia|	0.0186	0.20614	0.00265	0.89493	0.07642	3.72314
k__Bacteria|p__Fusobacteria|c__Fusobacteriia|	0.0186	0.20526	0.00265	0.89493	0.07642	3.72314
k__Bacteria|p__Fusobacteria|c__Fusobacteriia|	0	0	0	0	0	0
k__Bacteria|p__Fusobacteria|c__Fusobacteriia|	0	0	0	0	0	0
k__Bacteria|p__Fusobacteria|c__Fusobacteriia|	0.0186	0.20526	0.00265	0.89493	0.07642	3.72314
k__Bacteria|p__Fusobacteria|c__Fusobacteriia|	0	0	0	0	0	0
k__Bacteria|p__Fusobacteria|c__Fusobacteriia|	0	0	0	0.75923	0.07517	3.7015
k__Bacteria|p__Fusobacteria|c__Fusobacteriia|	0	0	0	0	0	0

k__Bacteria|p__Fusobacteria|c__Fusobacteriia|	0	0.02892	0	0.00031	0	0
k__Bacteria|p__Fusobacteria|c__Fusobacteriia|	0	0.00512	0	0	0	0
k__Bacteria|p__Fusobacteria|c__Fusobacteriia|	0	0	0	0	0	0
k__Bacteria|p__Fusobacteria|c__Fusobacteriia|	0	0	0	0	0	0
k__Bacteria|p__Fusobacteria|c__Fusobacteriia|	0.0186	0.17121	0.00265	0.13538	0.00126	0.02164
k__Bacteria|p__Fusobacteria|c__Fusobacteriia|	0	0.00088	0	0	0	0
k__Bacteria|p__Fusobacteria|c__Fusobacteriia|	0	0.00088	0	0	0	0
k__Bacteria|p__Fusobacteria|c__Fusobacteriia|	0	0	0	0	0	0
k__Bacteria|p__Fusobacteria|c__Fusobacteriia|	0	0	0	0	0	0
k__Bacteria|p__Fusobacteria|c__Fusobacteriia|	0	0.00088	0	0	0	0
k__Bacteria|p__Fusobacteria|c__Fusobacteriia|	0	0	0	0	0	0
k__Bacteria|p__Fusobacteria|c__Fusobacteriia|	0	0	0	0	0	0
k__Bacteria|p__Lentisphaerae	0	0	0	0	0	0
k__Bacteria|p__Lentisphaerae|c__Lentisphaeria	0	0	0	0	0	0
k__Bacteria|p__Lentisphaerae|c__Lentisphaeria	0	0	0	0	0	0
k__Bacteria|p__Lentisphaerae|c__Lentisphaeria	0	0	0	0	0	0
k__Bacteria|p__Lentisphaerae|c__Lentisphaeria	0	0	0	0	0	0
k__Bacteria|p__Lentisphaerae|c__Lentisphaeria	0	0	0	0	0	0
k__Bacteria|p__Proteobacteria	3.17912	5.42342	6.24285	8.94567	5.35436	4.66572
k__Bacteria|p__Proteobacteria|c__Betaproteoba	2.71377	2.47885	1.51145	1.81428	1.67329	2.39081
k__Bacteria|p__Proteobacteria|c__Betaproteoba	2.71377	2.32756	1.51145	1.81428	1.67329	2.39081
k__Bacteria|p__Proteobacteria|c__Betaproteoba	0	0	0.00052	0	0	0
k__Bacteria|p__Proteobacteria|c__Betaproteoba	0	0	0.00052	0	0	0
k__Bacteria|p__Proteobacteria|c__Betaproteoba	0	0	0.00052	0	0	0
k__Bacteria|p__Proteobacteria|c__Betaproteoba	0	0	0	0.00024	0	0
k__Bacteria|p__Proteobacteria|c__Betaproteoba	0	0	0	0.00024	0	0
k__Bacteria|p__Proteobacteria|c__Betaproteoba	0	0	0	0.00024	0	0
k__Bacteria|p__Proteobacteria|c__Betaproteoba	0.01211	0.11839	0.00995	0	0	0
k__Bacteria|p__Proteobacteria|c__Betaproteoba	0.01211	0.11839	0.00995	0	0	0
k__Bacteria|p__Proteobacteria|c__Betaproteoba	0.01211	0.11839	0.00995	0	0	0
k__Bacteria|p__Proteobacteria|c__Betaproteoba	2.70166	2.20916	1.50098	1.81404	1.67329	2.39081
k__Bacteria|p__Proteobacteria|c__Betaproteoba	2.60687	2.12553	1.45084	0	0	0
k__Bacteria|p__Proteobacteria|c__Betaproteoba	2.60687	2.12553	1.45084	0	0	0
k__Bacteria|p__Proteobacteria|c__Betaproteoba	0	0	0	1.81404	1.67329	2.39081
k__Bacteria|p__Proteobacteria|c__Betaproteoba	0	0	0	1.81404	1.67329	2.39081

k__Bacteria|p__Proteobacteria|c__Betaproteoba	0.09479	0.08364	0.05014	0	0	0
k__Bacteria|p__Proteobacteria|c__Betaproteoba	0.09479	0.08364	0.05014	0	0	0
k__Bacteria|p__Proteobacteria|c__Betaproteoba	0	0.15129	0	0	0	0
k__Bacteria|p__Proteobacteria|c__Betaproteoba	0	0.15129	0	0	0	0
k__Bacteria|p__Proteobacteria|c__Betaproteoba	0	0.00616	0	0	0	0
k__Bacteria|p__Proteobacteria|c__Betaproteoba	0	0.00616	0	0	0	0
k__Bacteria|p__Proteobacteria|c__Betaproteoba	0	0.14513	0	0	0	0
k__Bacteria|p__Proteobacteria|c__Betaproteoba	0	0	0	0	0	0
k__Bacteria|p__Proteobacteria|c__Betaproteoba	0	0.11459	0	0	0	0
k__Bacteria|p__Proteobacteria|c__Betaproteoba	0	0.01354	0	0	0	0
k__Bacteria|p__Proteobacteria|c__Betaproteoba	0	0.00828	0	0	0	0
k__Bacteria|p__Proteobacteria|c__Betaproteoba	0	0.00872	0	0	0	0
k__Bacteria|p__Proteobacteria|c__Deltaproteob	0.15516	0.19132	0.1346	0.27091	0	0.16462
k__Bacteria|p__Proteobacteria|c__Deltaproteob	0.15516	0.19132	0.1346	0.27091	0	0.16462
k__Bacteria|p__Proteobacteria|c__Deltaproteob	0.15516	0.19132	0.1346	0.27091	0	0.16462
k__Bacteria|p__Proteobacteria|c__Deltaproteob	0.15516	0.19132	0.1346	0.02022	0	0.05699
k__Bacteria|p__Proteobacteria|c__Deltaproteob	0.15516	0.19132	0.1346	0.02022	0	0.05699
k__Bacteria|p__Proteobacteria|c__Deltaproteob	0	0	0	0.25068	0	0.10763
k__Bacteria|p__Proteobacteria|c__Deltaproteob	0	0	0	0	0	0
k__Bacteria|p__Proteobacteria|c__Deltaproteob	0	0	0	0.25068	0	0.10763
k__Bacteria|p__Proteobacteria|c__Epsilonprote	0	0.02459	0.00015	0	0	0
k__Bacteria|p__Proteobacteria|c__Epsilonprote	0	0.02459	0.00015	0	0	0
k__Bacteria|p__Proteobacteria|c__Epsilonprote	0	0.02459	0.00015	0	0	0
k__Bacteria|p__Proteobacteria|c__Epsilonprote	0	0.02459	0.00015	0	0	0
k__Bacteria|p__Proteobacteria|c__Epsilonprote	0	0.01728	0.00015	0	0	0
k__Bacteria|p__Proteobacteria|c__Epsilonprote	0	0	0	0	0	0
k__Bacteria|p__Proteobacteria|c__Epsilonprote	0	0	0	0	0	0
k__Bacteria|p__Proteobacteria|c__Epsilonprote	0	0	0	0	0	0
k__Bacteria|p__Proteobacteria|c__Epsilonprote	0	0.00731	0	0	0	0
k__Bacteria|p__Proteobacteria|c__Epsilonprote	0	0	0	0	0	0
k__Bacteria|p__Proteobacteria|c__Epsilonprote	0	0	0	0	0	0
k__Bacteria|p__Proteobacteria|c__Epsilonprote	0	0	0	0	0	0
k__Bacteria|p__Proteobacteria|c__Epsilonprote	0	0	0	0	0	0
k__Bacteria|p__Proteobacteria|c__Gammaprote	0.18983	2.63569	4.52851	6.86048	3.68107	2.1103
k__Bacteria|p__Proteobacteria|c__Gammaprote	0	0	0	0	0	0

k__Bacteria|p__Proteobacteria|c__Gammaprote	0	0	0	0	0	0
k__Bacteria|p__Proteobacteria|c__Gammaprote	0	0	0	0	0	0
k__Bacteria|p__Proteobacteria|c__Gammaprote	0	0	0	0	0	0
k__Bacteria|p__Proteobacteria|c__Gammaprote	0	0	0	0	0	0
k__Bacteria|p__Proteobacteria|c__Gammaprote	0	0	0	0	0	0
k__Bacteria|p__Proteobacteria|c__Gammaprote	0	0	0	0	0	0
k__Bacteria|p__Proteobacteria|c__Gammaprote	0	0	0	0	0	0
k__Bacteria|p__Proteobacteria|c__Gammaprote	0	0	0	0	0	0
k__Bacteria|p__Proteobacteria|c__Gammaprote	0	0	0	0	0	0
k__Bacteria|p__Proteobacteria|c__Gammaprote	0	0	0	0	0	0
k__Bacteria|p__Proteobacteria|c__Gammaprote	0	0	0	0	0	0
k__Bacteria|p__Proteobacteria|c__Gammaprote	0	0	0	0	0	0
k__Bacteria|p__Proteobacteria|c__Gammaprote	0	0	0	0	0	0
k__Bacteria|p__Proteobacteria|c__Gammaprote	0.18983	1.75169	4.44838	6.86048	3.68107	2.10024
k__Bacteria|p__Proteobacteria|c__Gammaprote	0	0	0	0	0	0
k__Bacteria|p__Proteobacteria|c__Gammaprote	0	0	0	0	0	0
k__Bacteria|p__Proteobacteria|c__Gammaprote	0	0	0	0	0	0
k__Bacteria|p__Proteobacteria|c__Gammaprote	0.18983	1.75169	4.44838	6.86048	3.68107	2.10024
k__Bacteria|p__Proteobacteria|c__Gammaprote	0.01233	0.09176	0	0	0	0
k__Bacteria|p__Proteobacteria|c__Gammaprote	0.00101	0.03848	0	0	0	0
k__Bacteria|p__Proteobacteria|c__Gammaprote	0	0	0	0	0	0
k__Bacteria|p__Proteobacteria|c__Gammaprote	0	0	0	0	0	0
k__Bacteria|p__Proteobacteria|c__Gammaprote	0	0.00513	0	0	0	0
k__Bacteria|p__Proteobacteria|c__Gammaprote	0.00191	0.00874	0	0	0	0
k__Bacteria|p__Proteobacteria|c__Gammaprote	0	0	0	0	0	0
k__Bacteria|p__Proteobacteria|c__Gammaprote	0	0	0	0	0	0
k__Bacteria|p__Proteobacteria|c__Gammaprote	0.00097	0.01344	0	0	0	0
k__Bacteria|p__Proteobacteria|c__Gammaprote	0	0.0003	0	0	0	0
k__Bacteria|p__Proteobacteria|c__Gammaprote	0	0	0	0	0	0
k__Bacteria|p__Proteobacteria|c__Gammaprote	0.00844	0.02568	0	0	0	0
k__Bacteria|p__Proteobacteria|c__Gammaprote	0	0	0	0	0	0
k__Bacteria|p__Proteobacteria|c__Gammaprote	0	0	0	0	0	0
k__Bacteria|p__Proteobacteria|c__Gammaprote	0	0	0.0243	0.00157	0	0
k__Bacteria|p__Proteobacteria|c__Gammaprote	0	0	0.00179	0	0	0
k__Bacteria|p__Proteobacteria|c__Gammaprote	0	0	0.01084	0.00157	0	0

k__Bacteria|p__Proteobacteria|c__Gammaprote	0	0	0.01167	0	0	0
k__Bacteria|p__Proteobacteria|c__Gammaprote	0.15583	1.58515	0.02258	6.83818	3.68107	2.10024
k__Bacteria|p__Proteobacteria|c__Gammaprote	0.15583	1.58515	0.02258	6.83818	3.68107	2.10024
k__Bacteria|p__Proteobacteria|c__Gammaprote	0	0	0	0	0	0
k__Bacteria|p__Proteobacteria|c__Gammaprote	0.02167	0.07478	4.39893	0.02072	0	0
k__Bacteria|p__Proteobacteria|c__Gammaprote	0	0.0064	0	0	0	0
k__Bacteria|p__Proteobacteria|c__Gammaprote	0	0	0	0	0	0
k__Bacteria|p__Proteobacteria|c__Gammaprote	0	0	0	0	0	0
k__Bacteria|p__Proteobacteria|c__Gammaprote	0.01945	0.05293	2.36269	0.01106	0	0
k__Bacteria|p__Proteobacteria|c__Gammaprote	0	0.00216	1.06942	0.0072	0	0
k__Bacteria|p__Proteobacteria|c__Gammaprote	0.00222	0.01329	0.96681	0.00246	0	0
k__Bacteria|p__Proteobacteria|c__Gammaprote	0	0	0	0	0	0
k__Bacteria|p__Proteobacteria|c__Gammaprote	0	0	0	0	0	0
k__Bacteria|p__Proteobacteria|c__Gammaprote	0	0	0	0	0	0
k__Bacteria|p__Proteobacteria|c__Gammaprote	0	0	0	0	0	0
k__Bacteria|p__Proteobacteria|c__Gammaprote	0	0	0.00258	0	0	0
k__Bacteria|p__Proteobacteria|c__Gammaprote	0	0	0.00258	0	0	0
k__Bacteria|p__Proteobacteria|c__Gammaprote	0	0	0	0	0	0
k__Bacteria|p__Proteobacteria|c__Gammaprote	0	0	0	0	0	0
k__Bacteria|p__Proteobacteria|c__Gammaprote	0	0	0	0	0	0
k__Bacteria|p__Proteobacteria|c__Gammaprote	0	0	0	0	0	0
k__Bacteria|p__Proteobacteria|c__Gammaprote	0	0	0	0	0	0
k__Bacteria|p__Proteobacteria|c__Gammaprote	0	0	0	0	0	0
k__Bacteria|p__Proteobacteria|c__Gammaprote	0	0	0	0	0	0
k__Bacteria|p__Proteobacteria|c__Gammaprote	0	0	0	0	0	0
k__Bacteria|p__Proteobacteria|c__Gammaprote	0	0	0	0	0	0
k__Bacteria|p__Proteobacteria|c__Gammaprote	0	0	0	0	0	0
k__Bacteria|p__Proteobacteria|c__Gammaprote	0	0.88399	0.08013	0	0	0.01005
k__Bacteria|p__Proteobacteria|c__Gammaprote	0	0.88399	0.08013	0	0	0.01005
k__Bacteria|p__Proteobacteria|c__Gammaprote	0	0.08831	0.00073	0	0	0
k__Bacteria|p__Proteobacteria|c__Gammaprote	0	0.08327	0	0	0	0
k__Bacteria|p__Proteobacteria|c__Gammaprote	0	0.00504	0.00073	0	0	0
k__Bacteria|p__Proteobacteria|c__Gammaprote	0	0.79569	0.0794	0	0	0.01005
k__Bacteria|p__Proteobacteria|c__Gammaprote	0	0.01066	0	0	0	0
k__Bacteria|p__Proteobacteria|c__Gammaprote	0	0	0	0	0	0

k__Bacteria|p__Proteobacteria|c__Gammaprote	0	0.61975	0.05067	0	0	0.01005
k__Bacteria|p__Proteobacteria|c__Gammaprote	0	0.01245	0.00027	0	0	0
k__Bacteria|p__Proteobacteria|c__Gammaprote	0	0	0	0	0	0
k__Bacteria|p__Proteobacteria|c__Gammaprote	0	0	0.00343	0	0	0
k__Bacteria|p__Proteobacteria|c__Gammaprote	0	0.15283	0.02504	0	0	0
k__Bacteria|p__Proteobacteria|c__Gammaprote	0	0	0	0	0	0
k__Bacteria|p__Proteobacteria|c__Gammaprote	0	0	0	0	0	0
k__Bacteria|p__Proteobacteria|c__Gammaprote	0	0	0	0	0	0
k__Bacteria|p__Proteobacteria|c__Gammaprote	0	0	0	0	0	0
k__Bacteria|p__Proteobacteria|c__Gammaprote	0	0	0	0	0	0
k__Bacteria|p__Proteobacteria|c__Gammaprote	0	0	0	0	0	0
k__Bacteria|p__Proteobacteria|c__Gammaprote	0	0	0	0	0	0
k__Bacteria|p__Proteobacteria|c__Proteobacter	0.12036	0.09297	0.06814	0	0	0
k__Bacteria|p__Proteobacteria|c__Proteobacter	0.12036	0.09297	0.06814	0	0	0
k__Bacteria|p__Proteobacteria|c__Proteobacter	0.12036	0.09297	0.06814	0	0	0
k__Bacteria|p__Proteobacteria|c__Proteobacter	0.12036	0.09297	0.06814	0	0	0
k__Bacteria|p__Proteobacteria|c__Proteobacter	0.12036	0.09297	0.06814	0	0	0
k__Bacteria|p__Synergistetes	0.22912	0.49442	0.0268	0.02616	0	0
k__Bacteria|p__Synergistetes|c__Synergistia	0.22912	0.49442	0.0268	0.02616	0	0
k__Bacteria|p__Synergistetes|c__Synergistia|o_	0.22912	0.49442	0.0268	0.02616	0	0
k__Bacteria|p__Synergistetes|c__Synergistia|o_	0.22912	0.49442	0.0268	0.02616	0	0
k__Bacteria|p__Synergistetes|c__Synergistia|o_	0.02452	0.20054	0.01885	0.02616	0	0
k__Bacteria|p__Synergistetes|c__Synergistia|o_	0.0052	0.06313	0.00229	0.02616	0	0
k__Bacteria|p__Synergistetes|c__Synergistia|o_	0.01933	0.13741	0.01656	0	0	0
k__Bacteria|p__Synergistetes|c__Synergistia|o_	0	0.0015	0	0	0	0
k__Bacteria|p__Synergistetes|c__Synergistia|o_	0	0.0015	0	0	0	0
k__Bacteria|p__Synergistetes|c__Synergistia|o_	0.2046	0.29238	0.00795	0	0	0
k__Bacteria|p__Synergistetes|c__Synergistia|o_	0.2005	0.28551	0.00795	0	0	0
k__Bacteria|p__Synergistetes|c__Synergistia|o_	0.0041	0.00687	0	0	0	0
k__Bacteria|p__Tenericutes	0	0	0	0	0	0
k__Bacteria|p__Tenericutes|c__Mollicutes	0	0	0	0	0	0
k__Bacteria|p__Tenericutes|c__Mollicutes|o__M	0	0	0	0	0	0
k__Bacteria|p__Tenericutes|c__Mollicutes|o__M	0	0	0	0	0	0
k__Bacteria|p__Tenericutes|c__Mollicutes|o__M	0	0	0	0	0	0
k__Bacteria|p__Tenericutes|c__Mollicutes|o__M	0	0	0	0	0	0

k__Bacteria|p__Verrucomicrobia	0	0	0.00315	0	0.00132	0
k__Bacteria|p__Verrucomicrobia|c__Verrucomic	0	0	0.00315	0	0.00132	0
k__Bacteria|p__Verrucomicrobia|c__Verrucomic	0	0	0.00315	0	0.00132	0
k__Bacteria|p__Verrucomicrobia|c__Verrucomic	0	0	0.00315	0	0.00132	0
k__Bacteria|p__Verrucomicrobia|c__Verrucomic	0	0	0.00315	0	0.00132	0
k__Bacteria|p__Verrucomicrobia|c__Verrucomic	0	0	0.00315	0	0.00132	0
k__Archaea	0	0	0	0	0	0
k__Archaea|p__Euryarchaeota	0	0	0	0	0	0
k__Archaea|p__Euryarchaeota|c__Methanobac	0	0	0	0	0	0
k__Archaea|p__Euryarchaeota|c__Methanobac	0	0	0	0	0	0
k__Archaea|p__Euryarchaeota|c__Methanobac	0	0	0	0	0	0
k__Archaea|p__Euryarchaeota|c__Methanobac	0	0	0	0	0	0
k__Archaea|p__Euryarchaeota|c__Methanobac	0	0	0	0	0	0
k__Archaea|p__Euryarchaeota|c__Methanobac	0	0	0	0	0	0
k__Archaea|p__Euryarchaeota|c__Methanobac	0	0	0	0	0	0
k__Archaea|p__Euryarchaeota|c__Methanobac	0	0	0	0	0	0
k__Eukaryota	0	0	0	0	0	0
k__Eukaryota|p__Ascomycota	0	0	0	0	0	0
k__Eukaryota|p__Ascomycota|c__Saccharomyc	0	0	0	0	0	0
k__Eukaryota|p__Ascomycota|c__Saccharomyc	0	0	0	0	0	0
k__Eukaryota|p__Ascomycota|c__Saccharomyc	0	0	0	0	0	0
k__Eukaryota|p__Ascomycota|c__Saccharomyc	0	0	0	0	0	0
k__Eukaryota|p__Ascomycota|c__Saccharomyc	0	0	0	0	0	0
k__Eukaryota|p__Ascomycota|c__Saccharomyc	0	0	0	0	0	0
k__Eukaryota|p__Ascomycota|c__Saccharomyc	0	0	0	0	0	0
k__Eukaryota|p__Ascomycota|c__Saccharomyc	0	0	0	0	0	0

AP003_1	AP003_2	AP003_3	AP004_1	AP004_2	AP004_3	AP005_1	AP005_2
100	100	100	100	99.78705	99.9923	100	100
0.06822	0.51562	0.10298	0.61818	1.15181	0.0021	0.31094	0.05724
0.01442	0.0059	0.09202	0.59032	1.15181	0.0021	0.01345	0.00718
0.00434	0.00197	0.08926	0.13286	0.09001	0	0.00431	0.00718
0.00434	0.00197	0.08926	0.13286	0.09001	0	0.00431	0.00718
0	0.00084	0	0	0	0	0	0.00621
0	0.00084	0	0	0	0	0	0.00621
0.00434	0.00113	0.08926	0.13286	0.09001	0	0.00431	0.00097
0	0	0	0	0	0	0	0
0	0	0.03335	0.07834	0.08813	0	0	0
0	0	0	0	0	0	0	0
0	0	0	0	0	0	0	0
0	0	0	0	0	0	0	0
0	0	0.00067	0	0	0	0	0
0.00043	0	0.00319	0.00979	0.0009	0	0.00179	0.00083
0	0	0.00785	0.00953	0	0	0	0
0	0	0.00245	0.01692	0.00006	0	0	0
0	0	0	0	0	0	0	0
0.00391	0	0.04044	0.00099	0	0	0.00252	0
0	0	0.0013	0.00598	0	0	0	0.00015
0	0	0	0	0	0	0	0
0	0	0	0.0113	0.00093	0	0	0
0	0.00113	0	0	0	0	0	0
0	0	0	0	0	0	0	0
0	0	0	0	0	0	0	0
0	0	0	0	0	0	0	0
0.01001	0.0001	0.00277	0.41473	0.00509	0.00072	0.00418	0
0.01001	0.0001	0.00277	0.41473	0.00509	0.00072	0.00418	0
0	0	0	0	0	0	0	0
0	0	0	0	0	0	0	0
0	0	0	0	0	0	0	0
0	0	0	0	0	0	0	0
0.01001	0	0	0.4106	0.00223	0	0.00418	0

0	0	0	0	0	0	0	0
0.00425	0	0	0	0	0	0	0
0	0	0	0	0	0	0	0
0	0	0	0	0	0	0	0
0	0	0	0.4106	0.00223	0	0	0
0	0	0	0	0	0	0	0
0.00577	0	0	0	0	0	0.00418	0
0	0	0	0	0	0	0	0
0	0	0	0	0	0	0	0
0	0.0001	0.00277	0.00413	0.00286	0.00072	0	0
0	0.0001	0.00277	0.00413	0.00286	0.00072	0	0
0	0	0	0.03693	1.05426	0.00139	0	0
0	0	0	0.03693	1.05426	0.00139	0	0
0	0	0	0.03693	1.05426	0.00139	0	0
0	0	0	0.03693	0	0	0	0
0	0	0	0	0	0	0	0
0	0	0	0	1.05426	0.00139	0	0
0.00006	0	0	0.0058	0.00244	0	0	0
0.00006	0	0	0.0058	0.00244	0	0	0
0.00006	0	0	0.0058	0.00244	0	0	0
0	0	0	0.00125	0	0	0	0
0	0	0	0	0	0	0	0
0.00006	0	0	0.00455	0.00244	0	0	0
0	0.00383	0	0	0	0	0.00497	0
0	0.00383	0	0	0	0	0.00497	0
0	0.00383	0	0	0	0	0.00497	0
0	0.00383	0	0	0	0	0.00497	0
0	0	0	0	0	0	0	0
0	0	0	0	0	0	0	0
0.05381	0.50972	0.01096	0.02786	0	0	0.29749	0.05006
0.00525	0	0.00256	0.02786	0	0	0.1209	0
0	0	0.0008	0.02786	0	0	0.00737	0
0	0	0.0008	0.00125	0	0	0.00737	0
0	0	0	0	0	0	0	0
0	0	0.0008	0.00125	0	0	0.00737	0

0	0	0	0	0	0	0	0
0	0	0	0.02661	0	0	0	0
0	0	0	0	0	0	0	0
0	0	0	0.02661	0	0	0	0
0.00525	0	0.00176	0	0	0	0.11354	0
0.00166	0	0.00176	0	0	0	0.11354	0
0.00063	0	0.00033	0	0	0	0.11091	0
0.00061	0	0.00134	0	0	0	0.00048	0
0.00042	0	0.0001	0	0	0	0.00215	0
0.00359	0	0	0	0	0	0	0
0	0	0	0	0	0	0	0
0.00359	0	0	0	0	0	0	0
0.04856	0.50972	0.0084	0	0	0	0.17658	0.05006
0.04856	0.50972	0.0084	0	0	0	0.17658	0.05006
0	0	0	0	0	0	0	0
0	0	0	0	0	0	0	0
0	0	0	0	0	0	0	0
0	0	0	0	0	0	0	0
0.04595	0.46876	0.00777	0	0	0	0.15756	0.0451
0.04595	0.46876	0.00777	0	0	0	0.15756	0.0451
0	0	0	0	0	0	0	0
0	0	0	0	0	0	0	0
0.0026	0.04096	0.00063	0	0	0	0.01902	0.00496
0.0026	0.04096	0.00063	0	0	0	0.01902	0.00496
0	0	0	0	0	0	0	0
0	0	0	0	0	0	0	0
59.14788	3.36964	25.36972	0.00954	0.00223	0.00766	63.54947	20.61378
0	0	0	0	0	0	0	0
0	0	0	0	0	0	0	0
0	0	0	0	0	0	0	0
0	0	0	0	0	0	0	0
0	0	0	0	0	0	0	0
59.14788	3.36964	25.36972	0.00954	0.00223	0.00766	63.54947	20.61378
59.14788	3.36964	25.36972	0.00954	0.00223	0.00766	63.54947	20.61378
52.96292	3.1613	23.00454	0.00954	0.0008	0.00766	35.80528	5.47107

52.96292	3.1613	23.00454	0.00954	0.0008	0.00766	35.80528	5.47107
0.09284	0	2.36506	0	0	0	0	0
0.19764	0	0	0	0	0	0.00841	0.00755
1.5081	0	0	0	0	0	0	0
21.30127	0.05092	4.02257	0	0	0	0.00017	0
0.00103	0	0.17734	0	0	0	0	0
0	0	0	0	0	0	0	0
0	0	0	0	0	0	0	0
0	0	0	0	0	0	0.15416	0
10.03033	0.1016	6.28894	0.00109	0	0.00101	0.05913	0.12218
0.02504	0.05258	0.00138	0	0	0	0.16789	0.00796
0	0	0	0	0	0	0	0
0	0	0	0	0	0	0	0
0.0258	0.00326	0.14118	0	0	0	1.02658	0.85947
11.06317	0	0	0	0	0	0	0
0	0	0	0	0	0	0	0
0.25459	0	0.5702	0	0	0	0	0
1.60368	0.25133	0.0701	0	0	0	0	0
0.00015	0	0	0.00118	0	0.00082	1.66699	0.04495
0.40217	0.45094	1.31105	0.0002	0	0.0005	1.94047	0.77306
2.18826	1.60972	1.4452	0.00287	0.00004	0.0013	3.52388	0.58104
2.06258	0.00091	5.9626	0.0042	0.00075	0.00402	27.19363	2.62012
2.20628	0.64003	0.64892	0	0	0	0.06397	0.45473
0	0	0.03985	0	0	0	0	0
0	0	0	0	0	0	0	0
0	0	0	0	0	0	0	0
0	0	0.03985	0	0	0	0	0
0	0	0.03985	0	0	0	0	0
0	0	0	0	0	0	0	0
0.53918	0.13416	0.00312	0	0	0	4.59182	0.84351
0.53918	0.13416	0.00312	0	0	0	4.59182	0.84351
0.00075	0.00195	0	0	0	0	0	0
0.53844	0.13221	0.00312	0	0	0	4.59182	0.84351
0	0	0	0	0	0	0	0
0	0	0	0	0	0	0	0

0	0	0	0	0	0	0	0
0	0	0	0	0	0	0	0
0	0	0	0	0	0	0	0
0	0	0	0	0	0	0	0
0	0	0	0	0	0	0	0
0	0	0	0	0	0	0	0
0	0	0	0	0	0	0	0
0	0	0	0	0	0	0	0
0.23387	0	1.41455	0	0.00143	0	0.81885	0.8298
0	0	0	0	0	0	0	0.00011
0	0	0	0	0	0	0	0.00011
0.22826	0	1.41208	0	0	0	0	0
0.09049	0	0.60065	0	0	0	0	0
0.13778	0	0.81142	0	0	0	0	0
0.00561	0	0.00247	0	0.00143	0	0.81885	0.82969
0	0	0	0	0	0	0.0051	0.00914
0	0	0	0	0	0	0	0
0	0	0.00247	0	0	0	0	0
0.00358	0	0	0	0	0	0	0
0	0	0	0	0.00143	0	0.81375	0.82055
0	0	0	0	0	0	0	0
0	0	0	0	0	0	0	0
0	0	0	0	0	0	0	0
0	0	0	0	0	0	0	0
0	0	0	0	0	0	0	0
0	0	0	0	0	0	0	0
0	0	0	0	0	0	0	0
0	0	0	0	0	0	0	0
0	0	0	0	0	0	0	0
0	0	0	0	0	0	0	0
0	0	0	0	0	0	0	0
0.00203	0	0	0	0	0	0	0
0	0	0	0	0	0	0	0
0.38942	0.07268	0.03459	0	0	0	12.07337	7.30099
0.38942	0.07268	0.03459	0	0	0	12.07337	7.30099

0.38942	0.07268	0.01779	0	0	0	11.30633	7.23731
0	0	0.0168	0	0	0	0.76683	0.063
0	0	0	0	0	0	0	0
0	0	0	0	0	0	0	0
0	0	0	0	0	0	0.00022	0.00058
0	0	0	0	0	0	0	0.0001
5.02248	0.0015	0.87307	0	0	0	10.26015	6.16841
5.02248	0.0015	0.87307	0	0	0	10.26015	6.16841
3.84377	0.0015	0.86657	0	0	0	6.01406	5.84321
0	0	0	0	0	0	4.24609	0.32511
0	0	0	0	0	0	0	0
0	0	0	0	0	0	0	0
1.17871	0	0.0065	0	0	0	0	0
0	0	0	0	0	0	0	0.00008
0	0	0	0	0	0	0	0
0	0	0	0	0	0	0	0
0	0	0	0	0	0	0	0
0	0	0	0	0	0	0	0
0	0	0	0	0	0	0	0
0	0	0	0	0	0	0	0
14.24619	84.39425	36.47494	72.02946	0.05509	1.4086	27.70158	9.37474
0.20954	12.41384	0.18329	1.58892	0.03489	1.4086	0.05763	0.04023
0.00519	0.00128	0.0148	0.03856	0.01126	0.00131	0	0
0.00519	0.00128	0.0148	0.03856	0.01126	0.00131	0	0
0.00519	0.00128	0.0148	0.03856	0.01126	0.00131	0	0
0	0	0	0	0	0	0	0
0	0	0	0.00947	0	0	0	0
0	0	0	0.01741	0	0.00131	0	0
0.00519	0.00128	0.0148	0.01168	0.01126	0	0	0
0.20436	12.41257	0.16849	1.55037	0.02363	1.40729	0.05763	0.04023
0	0	0	0	0.00325	0	0.00641	0.04023
0	0	0	0	0.00325	0	0.00641	0.04023
0	0	0	0	0.00182	0	0.00194	0.0075
0	0	0	0	0.00143	0	0.00448	0.03273
0	0	0	0	0	0	0	0

0	0	0	0	0	0	0	0
0	0	0	0	0	0	0	0
0	0	0	0	0	0	0	0
0.20227	12.41257	0.12858	1.43778	0.00014	1.40264	0	0
0.20227	12.41257	0.12858	1.43778	0.00014	1.40264	0	0
0.09094	0.00045	0	0	0	0	0	0
0	0	0	0.03403	0	0	0	0
0	0	0	0.71841	0.00014	0	0	0
0	0.00427	0	0.13212	0	0.32828	0	0
0	0	0	0	0	0	0	0
0	0	0	0.49894	0	1.07436	0	0
0	0	0	0.0096	0	0	0	0
0	0	0	0.00113	0	0	0	0
0	0	0	0	0	0	0	0
0.11133	12.40785	0.12858	0.04356	0	0	0	0
0	0	0	0	0	0	0	0
0	0	0	0	0	0	0	0
0	0	0	0	0	0	0	0
0	0	0	0	0	0	0	0
0	0	0	0	0	0	0	0
0	0	0	0	0	0	0	0
0	0	0	0	0	0	0	0
0	0	0	0	0	0	0	0
0	0	0	0	0	0	0	0
0	0	0	0	0	0	0	0
0	0	0	0	0	0	0	0
0	0	0	0	0	0	0	0
0	0	0	0	0	0	0	0
0	0	0	0	0	0	0	0
0	0	0	0	0	0	0	0
0	0	0	0	0	0	0	0
0	0	0	0	0	0	0	0
0	0	0	0	0	0	0	0
0	0	0	0	0	0	0	0
0.00209	0	0.03991	0.11258	0.02024	0.00466	0.05121	0

0.00209	0	0.03991	0.11258	0.02024	0.00466	0.05121	0
0	0	0.00476	0	0	0	0.00284	0
0	0	0.00502	0.00164	0.00116	0	0	0
0	0	0	0	0	0	0	0
0	0	0	0	0	0	0	0
0	0	0	0	0	0	0	0
0	0	0.00025	0.00974	0	0	0.02821	0
0	0	0	0	0	0	0	0
0	0	0	0	0	0	0	0
0	0	0	0.03171	0.00668	0.00466	0.00653	0
0	0	0	0	0	0	0	0
0.00209	0	0	0.02118	0.00161	0	0	0
0	0	0.0136	0.0046	0	0	0.00693	0
0	0	0	0	0	0	0	0
0	0	0	0	0	0	0	0
0	0	0.00595	0.0393	0.00098	0	0.0067	0
0	0	0	0.00331	0.00111	0	0	0
0	0	0	0	0	0	0	0
0	0	0.00431	0.00111	0.00837	0	0	0
0	0	0	0	0	0	0	0
0	0	0	0	0	0	0	0
0	0	0	0	0.00032	0	0	0
0	0	0	0	0	0	0	0
0	0	0	0	0	0	0	0
0	0	0	0	0	0	0	0
0	0	0	0	0	0	0	0
0	0	0	0	0	0	0	0
0	0	0.00602	0	0	0	0	0
0	0	0	0	0	0	0	0
13.32215	64.2707	24.60834	60.12212	0.00927	0	24.15533	7.81662
13.32215	64.2707	24.60834	60.12212	0.00927	0	24.15533	7.81662
0	0.00163	0	0	0	0	0.00828	0.00273
0	0.00163	0	0	0	0	0.00828	0.00273
0	0.00163	0	0	0	0	0.00828	0.00273
0	0	0	0	0	0	0	0

0	0	0	0	0	0	0	0
0	0	0	0	0	0	0	0
0.17774	22.42004	1.84555	41.88925	0	0	1.34638	2.80039
0	0	0	0	0	0	0	0
0	0	0	0	0	0	0	0
0.11828	0.00463	1.72936	30.77554	0	0	0.89853	0
0	0	0	0	0	0	0	0
0.11828	0.00463	1.72936	28.70884	0	0	0	0
0	0	0	0.00187	0	0	0	0
0	0	0	0.00027	0	0	0	0
0	0	0	0.11097	0	0	0	0
0	0	0	0.34208	0	0	0	0
0	0	0	0.23927	0	0	0	0
0	0	0	1.32707	0	0	0	0
0	0	0	0.04517	0	0	0	0
0	0	0	0	0	0	0.01277	0
0	0	0	0	0	0	0	0
0	0	0	0	0	0	0.88577	0
0	0	0	0	0	0	0	0
0	0	0	0	0	0	0	0
0	0	0	0	0	0	0	0
0.05946	22.41541	0.09627	11.11371	0	0	0.44785	2.80039
0	0	0	0	0	0	0	0
0.05946	22.41541	0.09627	11.11371	0	0	0.44785	2.80039
0	0	0.01993	0	0	0	0	0
0	0	0.01993	0	0	0	0	0
0	0.00309	0.00303	0.00489	0	0	0.00028	0
0	0.00309	0.00236	0.00489	0	0	0	0
0	0.00199	0	0	0	0	0	0
0	0	0	0	0	0	0	0
0	0.0011	0	0	0	0	0	0
0	0	0.00236	0.00489	0	0	0	0
0	0	0.00068	0	0	0	0.00028	0
0	0	0.00068	0	0	0	0.00028	0
0.00116	0	0	0	0	0	0.00358	0.00903

0.00116	0	0	0	0	0	0	0
0.00116	0	0	0	0	0	0	0
0	0	0	0	0	0	0	0
0	0	0	0	0	0	0	0
0	0	0	0	0	0	0.00358	0.00903
0	0	0	0	0	0	0.00358	0.00903
0	0	0	0	0	0	0	0
0	0	0	0	0	0	0	0
0.22149	0.01941	0	0.00437	0	0	6.30788	0.12106
0	0	0	0	0	0	0	0
0	0	0	0	0	0	0	0
0	0	0	0	0	0	0	0
0	0	0	0	0	0	0	0
0.22149	0.01941	0	0	0	0	6.30788	0.12106
0	0	0	0	0	0	0	0
0	0	0	0	0	0	0	0
0	0	0	0	0	0	0	0
0.02826	0	0	0	0	0	0.00712	0
0	0.01941	0	0	0	0	0.00395	0
0.19323	0	0	0	0	0	0.0548	0
0	0	0	0	0	0	5.92146	0.12106
0	0	0	0	0	0	0.32055	0
0	0	0	0	0	0	0	0
0	0	0	0	0	0	0	0
0	0	0	0	0	0	0	0
0	0	0	0	0	0	0	0
0	0	0	0.00437	0	0	0	0
0	0	0	0.00437	0	0	0	0
8.47392	32.14355	20.63938	17.07347	0.00927	0	7.46422	4.19239
0.44746	0.14789	4.18688	0	0	0	0.00779	0
0	0	0	0	0	0	0	0
0.44746	0.14789	4.18688	0	0	0	0.00779	0
0.05492	0	0	0.99974	0	0	0.42632	0
0.05492	0	0	0.99974	0	0	0.42632	0
0.23728	0.0089	10.0259	0.064	0	0	1.74203	0.78275

0	0.00615	0.00453	0	0	0	0	0
0	0	0	0	0	0	0.10661	0
0.01607	0	0.00005	0	0	0	0	0
0.04996	0	0.04224	0	0	0	0.03194	0
0	0	0	0.064	0	0	0	0
0	0	0	0	0	0	0.11168	0.23447
0.04533	0	0.46966	0	0	0	0.05589	0
0.12591	0.00275	9.50942	0	0	0	0.96578	0.54828
0	0	0	0	0	0	0.47012	0
0	0	0	0	0	0	0	0
0	0	0	0	0	0	0	0
0	0	0	0	0	0	0	0
0	0	0	0	0	0	0	0
0	0	0	0	0	0	0	0
0	0	0	0	0	0	0.07931	0
0	0	0	0	0	0	0.0315	0
0	0	0	0	0	0	0.04782	0
0	0	0	0	0	0	0	0
0	0	0	0	0	0	0.19742	0.00052
0	0	0	0	0	0	0	0
0	0	0	0	0	0	0.19742	0.00052
0.0037	0.01829	0.00519	0	0	0	0	0
0.0037	0.01829	0.00519	0	0	0	0	0
0	0	0	0.00535	0	0	0.20673	0
0	0	0	0.00535	0	0	0.20673	0
0	0	0	0	0	0	0	0
0	0	0	0	0	0	0	0
1.14777	29.87503	6.41677	16.00437	0.00927	0	1.57577	3.03385
0.03817	0.09582	0.11141	2.81995	0	0	0.03303	0.00737
0	0	0	0	0	0	0.00491	0.00094
0.28157	17.3207	3.7149	0.09437	0	0	0.9214	0.12779
0.13066	0.05566	0.12338	0.96789	0	0	0.0983	2.73122
0.30938	0.60487	1.04529	0	0	0	0.00412	0.10547
0	0	0	0	0	0	0	0
0.04261	0.00522	0.1579	0	0	0	0.00758	0.00078

0	0	0	0	0	0	0	0
0.01146	0	0.01958	0	0	0	0	0
0.33392	11.79276	1.24431	12.12216	0.00927	0	0.50642	0.06028
0	0	0	0	0	0	0	0
0	0	0	0	0	0	0	0
0	0	0	0	0	0	0.50692	0.00046
1.26824	0.0005	0	0	0	0	2.26225	0.27686
1.26824	0.0005	0	0	0	0	0	0
0	0	0	0	0	0	2.26225	0.27686
0	0	0	0	0	0	0	0
0	0	0	0	0	0	0.50692	0.00046
0	0	0	0	0	0	0	0
0	0	0	0	0	0	0	0
0	0	0	0	0	0	0	0
0	0	0	0	0	0	0	0
0	0	0.00072	0	0	0	0	0
0	0	0	0	0	0	0	0
0	0	0.00072	0	0	0	0	0
0	0.33162	0	0	0	0	0	0
0	0.33162	0	0	0	0	0	0
5.29721	0	0	0	0	0	0.39599	0.04568
0	0	0	0	0	0	0.08257	0
0	0	0	0	0	0	0	0
0	0	0	0	0	0	0.02885	0
5.29721	0	0	0	0	0	0.26636	0.01609
0	0	0	0	0	0	0	0
0	0	0	0	0	0	0.0182	0.02959
0.01734	1.76133	0.00392	0	0	0	0.02884	0.03532
0.01734	1.76133	0.00392	0	0	0	0.02884	0.03532
0	0	0	0	0	0	0	0
0	0	0	0	0	0	0	0
0	0	0	0	0	0	0	0
0	0	0	0	0	0	0	0
0	0	0	0	0	0	0.03486	0.01696
0	0	0	0	0	0	0.03486	0.01696

0	0	0	0	0	0	0	0
0	0	0	0	0	0	0	0
0	0	0	0	0	0	0	0
0	0	0	0	0	0	0	0
0.06362	8.37068	1.03407	0.11952	0	0	0.07635	0.0259
0.06362	8.36993	1.03407	0	0	0	0	0.00809
0.06362	8.36993	1.03407	0	0	0	0	0.00809
0	0.00075	0	0	0	0	0	0
0	0.00075	0	0	0	0	0	0
0	0	0	0.11921	0	0	0.06774	0.0178
0	0	0	0.11921	0	0	0.06774	0.0178
0	0	0	0	0	0	0	0
0	0	0	0	0	0	0	0
0	0	0	0	0	0	0	0
0	0	0	0	0	0	0	0
0	0	0	0	0	0	0.0086	0
0	0	0	0	0	0	0.00362	0
0	0	0	0	0	0	0.00498	0
0	0	0	0.00032	0	0	0	0
0	0	0	0.00032	0	0	0	0
0	0	0	0	0	0	0	0
0	0	0	0	0	0	0	0
4.38423	1.3123	1.08631	1.03062	0	0	8.94836	0.66513
0.06259	0	0	0	0	0	0.0007	0
0.06259	0	0	0	0	0	0.0007	0
0	0	0	0	0	0	0	0
0	0	0	0	0	0	0	0
0	0	0	0	0	0	0	0
0	0	0	0	0	0	0	0
0.02199	0	0	0.0559	0	0	0.37939	0.04612
0.02199	0	0	0.0559	0	0	0.37939	0.04612
2.99918	0.19946	0.00815	0	0	0	5.63693	0.06253
2.99918	0.19946	0.00815	0	0	0	5.63693	0.06253
1.16994	1.0094	0.86538	0.2049	0	0	1.81078	0.48496
1.16994	1.0094	0.86538	0.2049	0	0	1.81078	0.48496

0	0	0	0	0	0	0	0
0	0	0	0	0	0	0.0641	0
0	0	0	0	0	0	0.0641	0
0	0	0	0	0	0	0	0
0	0	0	0	0	0	0	0
0	0	0	0	0	0	0	0
0	0	0	0	0	0	0	0
0	0	0	0	0	0	0	0
0	0	0.0002	0.15538	0	0	0.0063	0.03959
0	0	0.0002	0.15538	0	0	0.00252	0
0	0	0	0	0	0	0	0
0	0	0	0	0	0	0	0
0	0	0	0	0	0	0.00378	0.03959
0	0	0	0	0	0	0	0
0.03215	0	0	0	0	0	0	0.00175
0	0	0	0	0	0	0	0
0.03215	0	0	0	0	0	0	0.00175
0	0	0	0	0	0	0	0
0	0	0	0	0	0	0	0
0	0	0	0	0	0	0	0
0.09837	0.10344	0.21258	0.61444	0	0	1.05016	0.0302
0.09837	0.10344	0.21258	0.61444	0	0	1.05016	0.0302
0.25543	7.10281	0.72572	8.38394	0.00031	0	0.39679	0.44733
0.25543	7.10281	0.72572	8.38394	0.00031	0	0.39679	0.44733
0.25543	7.10281	0.72572	8.38394	0.00031	0	0.39679	0.44733
0	0	0	0	0	0	0	0
0	0	0	0	0	0	0	0
0	0	0	0	0	0	0	0
0	0	0	0	0	0	0	0
0	0	0	0	0	0	0	0
0	0	0	0	0	0	0	0
0	0	0.00034	0	0	0	0.12358	0
0	0	0.00034	0	0	0	0.12358	0
0	0	0	0	0	0	0.01503	0.04906
0	0	0	0	0	0	0.01503	0.04906

0	0	0	0	0	0	0	0
0	0	0	0	0	0	0	0
0.25543	7.10281	0.72539	8.36155	0.00021	0	0.1364	0.39828
0.02042	6.46378	0.0406	4.39536	0	0	0.10635	0.29158
0	0	0	0	0	0	0	0
0.23501	0.63903	0.68479	3.96619	0.00021	0	0.03005	0.1067
0	0	0	0	0	0	0	0
0	0	0	0	0	0	0	0
0	0	0	0	0	0	0	0
0	0	0	0	0	0	0	0
0	0	0	0	0	0	0	0
0	0	0	0	0	0	0	0
0	0	0	0	0	0	0.12178	0
0	0	0	0	0	0	0.12178	0
0	0	0	0	0	0	0	0
0	0	0	0	0	0	0	0
0	0	0	0	0	0	0	0
0	0	0	0	0	0	0	0
0	0	0	0.00013	0	0	0	0
0	0	0	0.00013	0	0	0	0
0	0	0	0.02226	0.0001	0	0	0
0	0	0	0.02226	0.0001	0	0	0
0	0.00855	0	0	0	0	0.01222	0
0	0.00855	0	0	0	0	0.01222	0
0	0.00855	0	0	0	0	0.01222	0
0	0.00855	0	0	0	0	0.01222	0
0	0	0	0	0	0	0	0
0	0	0	0	0	0	0	0
0	0	0	0	0	0	0	0
0	0	0	0	0	0	0	0
0	0	0	0	0	0	0	0
0	0	0	0	0	0	0.01222	0
0	0.00855	0	0	0	0	0	0
0	0	0	0	0	0	0	0
0.45907	0.59579	10.9563	1.91668	0	0	3.07912	1.07055

0.44929	0.59579	10.94979	0	0	0	2.19439	0.92301
0.44929	0.59579	10.94979	0	0	0	2.19439	0.92301
0	0	0	0	0	0	0.07398	0.14641
0	0	0	0	0	0	0.07398	0.14641
0	0	0	0	0	0	0	0
0.44929	0.59579	10.94979	0	0	0	2.12041	0.7766
0.44929	0.59579	10.94979	0	0	0	2.12041	0.7766
0	0	0	0	0	0	0	0
0	0	0	0	0	0	0.81334	0.00221
0	0	0	0	0	0	0.81334	0.00221
0	0	0	0	0	0	0.81334	0.00221
0	0	0	0	0	0	0.5858	0.00221
0	0	0	0	0	0	0.22754	0
0	0	0	0	0	0	0	0
0	0	0	0	0	0	0	0
0	0	0	0	0	0	0	0
0	0	0	0	0	0	0	0
0	0	0	0	0	0	0	0
0	0	0	0	0	0	0	0
0	0	0	0	0	0	0	0
0.00978	0	0.00651	1.91668	0	0	0.07139	0.14533
0.00978	0	0.00651	1.91668	0	0	0.07139	0.14533
0	0	0	0	0	0	0	0
0	0	0	0	0	0	0	0
0	0	0	0.00509	0	0	0.00017	0
0	0	0	0.00509	0	0	0.00017	0
0	0	0	0	0	0	0	0
0	0	0	0	0	0	0	0
0	0	0	0	0	0	0	0
0	0	0	0	0	0	0	0
0	0	0.00038	0	0	0	0.01642	0.01343
0	0	0	0	0	0	0	0
0	0	0	0	0	0	0	0
0	0	0.00038	0	0	0	0.01642	0.01343
0	0	0	0	0	0	0	0

0	0	0	0	0	0	0	0
0.00978	0	0.00613	1.91159	0	0	0.05479	0.1319
0.00013	0	0	0.14995	0	0	0.00616	0
0	0	0	0.006	0	0	0	0
0	0	0	0.00118	0	0	0	0
0.00965	0	0.00613	1.75446	0	0	0.04864	0.1319
0	0	0	0	0	0	0	0
0	0	0	0	0	0	0	0
0	0	0	0	0	0	0	0
0	0	0	0	0	0	0	0
0	0.00256	0.00128	0.0178	0.01062	0	0.00049	0
0	0.00256	0.00128	0.0178	0.01062	0	0.00049	0
0	0.00256	0.00128	0.0178	0.01062	0	0.00049	0
0	0	0	0	0	0	0	0
0	0	0	0	0	0	0	0
0	0	0	0	0	0	0	0
0	0	0	0	0	0	0	0
0	0	0	0	0	0	0	0
0	0.00256	0.00128	0.0178	0.01062	0	0.00049	0
0	0.00256	0.00128	0.0178	0.01062	0	0.00049	0
0	0	0	0	0	0	0	0
0	0	0	0	0	0	0	0
0	0	0	0	0	0	0	0
0	0	0	0	0	0	0	0
0	0	0	0	0	0	0	0
1.35567	10.20572	3.68068	8.86625	0	0	0.59769	0.28493
1.35567	10.20572	3.68068	8.86625	0	0	0.59769	0.28493
1.35567	10.20572	3.68068	8.86625	0	0	0.59769	0.28493
1.35567	10.20572	3.68068	8.86625	0	0	0.59769	0.28493
0	0	0	0	0	0	0	0
0	0	0	0	0	0	0	0
1.35567	10.20572	3.68068	8.86625	0	0	0.59769	0.28493
0	0	0	0	0	0	0	0
1.33932	10.06535	0.45348	1.71224	0	0	0	0
0	0	0	0	0	0	0	0

0	0	0	0.00254	0	0	0	0
0	0	0	0	0	0	0	0
0	0	0	0	0	0	0	0
0	0	0	0	0	0	0.0039	0.00384
0.01635	0.14037	3.2272	7.15148	0	0	0.59379	0.2811
0	0	0	0	0	0	0	0
0	0	0	0	0	0	0	0
0	0	0	0	0	0	0	0
0	0	0	0	0	0	0	0
0	0	0	0	0	0	0	0
0	0	0	0	0	0	0	0
0	0	0	0	0	0	0	0
0	0	0	0	0	0	0	0
0	0	0	0	0	0	0	0
0	0	0	0	0	0	0	0
0	0	0	0	0	0	0	0
0	0	0	0	0	0	0	0
0	0	0	0	0	0	0	0
25.18203	1.51445	34.37168	18.47656	98.57792	98.57393	7.82927	69.6242
0	0	0	0	0.00106	0	0.08961	0.15545
0	0	0	0	0	0	0.07859	0.14647
0	0	0	0	0	0	0	0.10505
0	0	0	0	0	0	0	0.10505
0	0	0	0	0	0	0	0.10505
0	0	0	0	0	0	0	0
0	0	0	0	0	0	0	0
0	0	0	0	0	0	0	0
0	0	0	0	0	0	0	0
0	0	0	0	0	0	0	0
0	0	0	0	0	0	0	0
0	0	0	0	0	0	0.07859	0.04143
0	0	0	0	0	0	0	0
0	0	0	0	0	0	0	0
0	0	0	0	0	0	0.07859	0.04143
0	0	0	0	0	0	0.07859	0.04143

0	0	0	0	0	0	0	0
0	0	0	0	0	0	0	0
0	0	0	0	0.00106	0	0.01102	0.00898
0	0	0	0	0.00106	0	0.01102	0.00898
0	0	0	0	0	0	0	0
0	0	0	0	0	0	0	0
0	0	0	0	0.00106	0	0.01102	0.00898
0	0	0	0	0	0	0	0.00898
0	0	0	0	0	0	0.0077	0
0	0	0	0	0	0	0.00332	0
0	0	0	0	0	0	0	0
0	0	0	0	0.00106	0	0	0
0.40531	0	0.48485	0	0	0	0.13146	0.07055
0.40531	0	0.48485	0	0	0	0.13146	0.07055
0.40531	0	0.48485	0	0	0	0.13146	0.07055
0.2026	0	0.36107	0	0	0	0.13146	0.07055
0.2026	0	0.36107	0	0	0	0.13146	0.07055
0.20271	0	0.12378	0	0	0	0	0
0	0	0	0	0	0	0	0
0.20271	0	0.12378	0	0	0	0	0
0	0	0	0	0	0	0.002	0.00561
0	0	0	0	0	0	0.002	0.00561
0	0	0	0	0	0	0	0.00561
0	0	0	0	0	0	0	0.00561
0	0	0	0	0	0	0	0
0	0	0	0	0	0	0	0.00561
0	0	0	0	0	0	0	0
0	0	0	0	0	0	0	0
0	0	0	0	0	0	0	0
0	0	0	0	0	0	0	0
0	0	0	0	0	0	0.002	0
0	0	0	0	0	0	0.002	0
0	0	0	0	0	0	0.002	0
24.77672	1.51445	33.88683	18.47656	98.57686	98.57393	7.6062	69.3926
0	0	0.00889	0	0	0	0	0

0	0	0	0	0	0	0	0
0	0	0	0	0	0	0	0
0	0	0	0	0	0	0	0
0	0	0	0	0	0	0	0
0	0	0	0	0	0	0	0
0	0	0.00889	0	0	0	0	0
0	0	0.00889	0	0	0	0	0
0	0	0.00889	0	0	0	0	0
0	0	0	0	0	0	0	0.00082
0	0	0	0	0	0	0	0.00082
0	0	0	0	0	0	0	0.00082
0	0	0	0	0	0	0	0
0	0	0	0	0	0	0	0.00082
24.77672	1.51445	33.87794	18.43759	98.57686	98.57393	7.59463	69.39178
0	0	0	0	0	0	0	0
0	0	0	0	0	0	0	0
0	0	0	0	0	0	0	0
24.77672	1.51445	33.87794	18.40762	98.57686	98.57393	7.59463	69.39178
0	0	0	0.02977	0	0	0	0
0	0	0	0.02977	0	0	0	0
0	0	0	0	0	0	0	0
0	0	0	0	0	0	0	0
0	0	0	0	0	0	0	0
0	0	0	0	0	0	0	0
0	0	0	0	0	0	0	0
0	0	0	0	0	0	0	0
0	0	0	0	0	0	0	0
0	0	0	0	0	0	0	0
0	0	0	0	0	0	0	0
0	0	0	0	0	0	0	0
0	0	0	0	0	0	0	0
0	0	0	0	0	0	0	0
0	0	0	0	0	0	0	0
0	0	0	0	0	0	0	0
0	0	0	0	0	0	0	0

0	0	0	0	0	0	0	0
24.77672	1.51445	33.87794	3.72891	98.57686	89.16344	0.06346	0.01602
24.77672	1.51445	33.87794	3.72891	98.57686	89.16344	0.06346	0.01602
0	0	0	0	0	0	0	0
0	0	0	14.53954	0	9.41049	7.52901	69.37576
0	0	0	0	0	0	0	0
0	0	0	0.10538	0	0	0	0
0	0	0	0.00693	0	0	0	0
0	0	0	8.02497	0	7.84947	3.25424	29.34829
0	0	0	4.61741	0	1.03648	3.79767	36.0341
0	0	0	1.78484	0	0.52454	0.47709	3.99337
0	0	0	0.03628	0	0	0	0
0	0	0	0.03628	0	0	0	0
0	0	0	0	0	0	0.00216	0
0	0	0	0	0	0	0.00216	0
0	0	0	0	0	0	0	0
0	0	0	0	0	0	0	0
0	0	0	0.07312	0	0	0	0
0	0	0	0.07312	0	0	0	0
0	0	0	0	0	0	0	0
0	0	0	0	0	0	0	0
0	0	0	0	0	0	0	0
0	0	0	0.02997	0	0	0	0
0	0	0	0	0	0	0	0
0	0	0	0	0	0	0	0
0	0	0	0.02997	0	0	0	0
0	0	0	0.02997	0	0	0	0
0	0	0	0.03897	0	0	0.01157	0
0	0	0	0.03897	0	0	0.01157	0
0	0	0	0.00925	0	0	0	0
0	0	0	0.00925	0	0	0	0
0	0	0	0	0	0	0	0
0	0	0	0.02972	0	0	0.01157	0
0	0	0	0	0	0	0	0
0	0	0	0	0	0	0	0

0	0	0	0.02972	0	0	0.00349	0
0	0	0	0	0	0	0	0
0	0	0	0	0	0	0	0
0	0	0	0	0	0	0.00808	0
0	0	0	0	0	0	0	0
0	0	0	0	0	0	0	0
0	0	0	0	0	0	0	0
0	0	0	0	0	0	0	0
0	0	0	0	0	0	0	0
0	0	0	0	0	0	0	0
0	0	0	0	0	0	0	0
0	0	0	0	0	0	0	0
0	0	0	0	0	0	0	0
0	0	0	0	0	0	0	0
0	0	0	0	0	0	0	0
0	0	0	0	0	0	0	0
0	0	0	0	0	0	0	0
0	0.00032	0	0	0	0	0.01106	0.01791
0	0.00032	0	0	0	0	0.01106	0.01791
0	0.00032	0	0	0	0	0.01106	0.01791
0	0.00032	0	0	0	0	0.01106	0.01791
0	0	0	0	0	0	0.01106	0.01791
0	0	0	0	0	0	0	0
0	0	0	0	0	0	0.01106	0.01791
0	0.00032	0	0	0	0	0	0
0	0.00032	0	0	0	0	0	0
0	0	0	0	0	0	0	0
0	0	0	0	0	0	0	0
0	0	0	0	0	0	0	0
0	0	0	0	0	0	0	0
0	0	0	0	0	0	0	0
0	0	0	0	0	0	0	0
0	0	0	0	0	0	0	0
0	0	0	0	0	0	0	0
0	0	0	0	0	0	0	0

0	0	0	0	0	0	0	0.02719
0	0	0	0	0	0	0	0.02719
0	0	0	0	0	0	0	0.02719
0	0	0	0	0	0	0	0.02719
0	0	0	0	0	0	0	0.02719
0	0	0	0	0	0	0	0.02719
0	0	0	0	0	0	0	0
0	0	0	0	0	0	0	0
0	0	0	0	0	0	0	0
0	0	0	0	0	0	0	0
0	0	0	0	0	0	0	0
0	0	0	0	0	0	0	0
0	0	0	0	0	0	0	0
0	0	0	0	0	0	0	0
0	0	0	0	0	0	0	0
0	0	0	0	0	0	0	0
0	0	0	0	0.21295	0.0077	0	0
0	0	0	0	0.21295	0.0077	0	0
0	0	0	0	0.21295	0.0077	0	0
0	0	0	0	0.21295	0.0077	0	0
0	0	0	0	0.21295	0.0077	0	0
0	0	0	0	0.21295	0.0077	0	0
0	0	0	0	0.21295	0.0077	0	0
0	0	0	0	0	0	0	0
0	0	0	0	0	0	0	0
0	0	0	0	0	0	0	0

AP005_3	AP006_1	AP006_2	AP006_3	AP007_1	AP007_2	AP007_3	AP008_1
100	100	100	100	99.03864	99.91704	99.9474	100
0.26567	0.49368	0.49153	0.03687	2.33668	0.0757	0.52405	0.40952
0.10747	0.47382	0.23458	0.03034	2.21901	0.057	0.48283	0.37363
0.10368	0.0018	0.00306	0	0.00103	0.00814	0.02919	0.20644
0.10368	0.0018	0.00306	0	0.00103	0.00814	0.02919	0.20644
0	0	0	0	0	0	0	0
0	0	0	0	0	0	0	0
0.10368	0.0018	0.00306	0	0.00103	0.00814	0.02919	0.20644
0	0	0	0	0	0	0	0
0	0	0	0	0	0.00306	0.00694	0.01974
0	0	0	0	0	0	0	0
0	0	0	0	0	0	0	0
0	0	0	0	0	0	0	0.00017
0	0	0	0	0	0	0	0
0.06819	0	0.00182	0	0.00046	0.00026	0.002	0.01652
0.00048	0	0	0	0	0	0	0.00204
0.00357	0	0	0	0.00057	0.00067	0.00069	0.00881
0.00071	0	0	0	0	0	0	0.00022
0.00772	0.0018	0.00061	0	0	0.00134	0.01486	0.12581
0.0173	0	0.00009	0	0	0.00061	0.00163	0.02998
0	0	0	0	0	0	0	0
0.00571	0	0.00054	0	0	0.00221	0.00305	0
0	0	0	0	0	0	0	0.00057
0	0	0	0	0	0	0	0.0024
0	0	0	0	0	0	0	0
0	0	0	0	0	0	0	0.00018
0.00074	0.47202	0.22474	0.03034	2.21375	0.03501	0.44747	0.14906
0.00074	0.47202	0.22474	0.03034	2.21375	0.03501	0.44747	0.14906
0	0	0	0	0	0	0	0
0	0	0	0	0	0	0	0
0	0	0	0	0	0	0	0
0	0	0	0	0	0	0	0
0.00074	0.47202	0.22474	0.03034	2.21375	0.03501	0.44747	0.14906

0	0	0	0	0	0	0	0
0	0	0	0	0	0	0.0008	0
0	0	0	0	0	0	0	0
0	0	0	0	0	0	0	0
0	0	0	0	0	0	0	0
0.00074	0.03934	0.04974	0.02577	0.04202	0.0094	0.01104	0.14906
0	0.43269	0.175	0.00457	2.17173	0.02562	0.43563	0
0	0	0	0	0	0	0	0
0	0	0	0	0	0	0	0
0	0	0	0	0	0	0	0
0	0	0	0	0	0	0	0
0	0	0	0	0	0	0	0.00255
0	0	0	0	0	0	0	0.00255
0	0	0	0	0	0	0	0.00255
0	0	0	0	0	0	0	0.00255
0	0	0	0	0	0	0	0
0	0	0	0	0	0	0	0
0.0022	0	0.00081	0	0.00423	0.01385	0.00618	0.01558
0.0022	0	0.00081	0	0.00423	0.01385	0.00618	0.01558
0.0022	0	0.00081	0	0.00423	0.01385	0.00618	0.01558
0	0	0	0	0	0.00058	0	0.00171
0	0	0	0	0	0	0	0
0.0022	0	0.00081	0	0.00423	0.01327	0.00618	0.01387
0.00084	0	0.00598	0	0	0	0	0
0.00084	0	0.00598	0	0	0	0	0
0.00084	0	0.00598	0	0	0	0	0
0.00084	0	0.00598	0	0	0	0	0
0	0	0	0	0	0	0	0
0	0	0	0	0	0	0	0
0.1582	0.01986	0.25695	0.00653	0.11767	0.01869	0.04121	0.03588
0.00257	0	0.02082	0	0	0.00559	0.00124	0.00533
0.00162	0	0.02057	0	0	0.00559	0.00124	0.00533
0.00162	0	0.01908	0	0	0.00559	0.00124	0.00533
0	0	0	0	0	0	0	0
0.00162	0	0.00846	0	0	0.00545	0.00124	0.00506

0	0	0.01063	0	0	0.00014	0	0.00027
0	0	0.00149	0	0	0	0	0
0	0	0	0	0	0	0	0
0	0	0.00149	0	0	0	0	0
0.00095	0	0.00024	0	0	0	0	0
0.00087	0	0.00024	0	0	0	0	0
0.00034	0	0.00015	0	0	0	0	0
0.00038	0	0	0	0	0	0	0
0.00015	0	0.00009	0	0	0	0	0
0.00008	0	0	0	0	0	0	0
0.00008	0	0	0	0	0	0	0
0	0	0	0	0	0	0	0
0.15563	0.01986	0.23613	0.00653	0.11767	0.0131	0.03998	0.03055
0.15563	0.01986	0.23613	0.00653	0.11767	0.0131	0.03998	0.03055
0	0	0	0	0.05187	0	0.01249	0
0	0	0	0	0.05187	0	0.01249	0
0	0	0	0	0.01379	0	0.00379	0
0	0	0	0	0.01379	0	0.00379	0
0.13959	0.01746	0.21038	0.00631	0.03599	0.005	0.0183	0.02926
0.13959	0.01746	0.21038	0.00631	0.03599	0.005	0.0183	0.02926
0	0	0	0	0.00015	0	0	0
0	0	0	0	0.00015	0	0	0
0.01604	0.0024	0.02273	0.00023	0.01586	0.0081	0.00538	0.00129
0.01604	0.0024	0.02273	0.00023	0.01586	0.0081	0.00538	0.00129
0	0	0.00302	0	0	0	0	0
0	0	0.00302	0	0	0	0	0
64.93894	75.85009	80.75881	85.22582	47.40815	51.7437	34.75329	7.93421
0	0	0	0	0	0	0	0
0	0	0	0	0	0	0	0
0	0	0	0	0	0	0	0
0	0	0	0	0	0	0	0
0	0	0	0	0	0	0	0
64.93894	75.85009	80.75881	85.22582	47.40815	51.7437	34.75329	7.93421
64.93894	75.85009	80.75881	85.22582	47.40815	51.7437	34.75329	7.93421
28.40433	74.00968	65.49286	83.03957	31.18691	49.20188	30.04984	5.93257

28.40433	74.00968	65.49286	83.03957	31.18691	49.20188	30.04984	5.93257
0	0	0	0	0	0	0	0.19677
0.05338	2.74397	0.33031	3.23357	0.52477	0	0.06255	0
0	0	0	0	0	0	0	0
0	0.00043	0	0	0	0	0.00021	0.00164
0	0	0	0	0	0	0	0.00015
0	0	0	0	0	0	0	0
0	0	0	0	0	0	0	0
0	0	0	0	0	0	0	0
0.00056	8.16806	32.12541	4.70461	0.88648	0.00241	0.12946	0.05242
0.01725	0.12262	0.04099	0.30721	0.33662	0	0.0641	0
0	0	0	0	0	0	0	0
0	0.01248	0.04484	0.04627	0.01062	0	0.00027	0
0.25131	4.2109	0.69281	3.77012	2.45491	0.31397	1.49032	0.03125
0	0	0.00016	0	0	0	0	0.0003
0	0	0	0	0	0	0	0
0	0	0	0	0	0	0	0
0	0	0	0	0	0	0	0
1.58679	0.00188	0.00011	0.00062	2.56752	1.20022	5.96137	3.54919
0.38379	5.17963	10.73021	12.97966	2.74349	0.33204	1.97113	0.23639
1.3179	32.44077	6.0161	28.72197	10.13321	0.23701	4.61239	0.07191
24.64504	21.11217	15.50244	29.20522	11.51014	47.11425	15.74031	1.72673
0.1483	0.01677	0.00949	0.07033	0.01914	0.00198	0.01772	0.06581
0	0	0	0	0	0	0	0
0	0	0	0	0	0	0	0
0	0	0	0	0	0	0	0
0	0	0	0	0	0	0	0
0	0	0	0	0	0	0	0
0	0	0	0	0	0	0	0
0.42133	0.28129	1.33196	0.05238	2.77394	1.12045	0.88879	0
0.42133	0.28129	1.33196	0.05238	1.20112	0	0.03961	0
0	0	0	0	0	0	0	0
0.42133	0.28129	1.33196	0.05238	1.20112	0	0.03961	0
0	0	0	0	1.57282	1.12045	0.84919	0
0	0	0	0	0	0	0	0

0	0	0	0	1.57282	1.12045	0.84919	0
0	0	0	0	0	0	0	0
0	0	0	0	0	0	0	0
0	0	0	0	0	0	0	0
0	0	0	0	0	0	0	0
0	0	0	0	0	0	0	0
0	0	0	0	0	0	0	0
0	0	0	0	0	0	0	0
31.59604	0	0.0023	0	0	0	0	0.00299
0	0	0	0	0	0	0	0
0	0	0	0	0	0	0	0
0	0	0	0	0	0	0	0
0	0	0	0	0	0	0	0
0	0	0	0	0	0	0	0
31.59604	0	0.0023	0	0	0	0	0.00299
0	0	0	0	0	0	0	0.00299
0	0	0	0	0	0	0	0
0	0	0	0	0	0	0	0
0	0	0	0	0	0	0	0
31.59604	0	0	0	0	0	0	0
0	0	0	0	0	0	0	0
0	0	0	0	0	0	0	0
0	0	0	0	0	0	0	0
0	0	0	0	0	0	0	0
0	0	0	0	0	0	0	0
0	0	0	0	0	0	0	0
0	0	0	0	0	0	0	0
0	0	0	0	0	0	0	0
0	0	0	0	0	0	0	0
0	0	0	0	0	0	0	0
0	0	0	0	0	0	0	0
0	0	0	0	0	0	0	0
0	0	0.0023	0	0	0	0	0
1.16555	0.01315	0.06386	0	11.14918	0.22047	2.78429	0.00012
1.16555	0.01315	0.06386	0	11.14918	0.22047	2.78429	0.00012

0.70008	0	0	0	0	0	0	0
0.46547	0.01315	0.06386	0	0.01841	0	0.0032	0
0	0	0	0	0	0	0	0
0	0	0	0	8.33607	0.01231	2.01	0.00012
0	0	0	0	2.7947	0.20816	0.7711	0
0	0	0	0	0	0	0	0
3.3517	1.54597	13.86782	2.13387	2.29812	1.20091	1.03036	1.99853
3.3517	1.54597	13.86782	2.13387	2.29812	1.20091	1.03036	1.99853
3.03103	1.52477	13.57256	2.09647	1.43839	0.06604	0.87905	1.39412
0.32067	0	0	0	0.85491	1.12699	0.14967	0
0	0	0	0	0	0	0	0
0	0	0	0	0.0004	0	0	0
0	0	0	0	0.00442	0.00787	0.00164	0.6044
0	0.0212	0.29527	0.0374	0	0	0	0
0	0	0	0	0	0	0	0
0	0	0	0	0	0	0	0
0	0	0	0	0	0	0	0
0	0	0	0	0	0	0	0
0	0	0	0	0	0	0	0
0	0	0	0	0	0	0	0
31.63679	22.33695	6.8928	9.55475	28.9441	11.03761	44.30602	17.7438
0.31721	0.08516	0.07168	0.0306	0.67977	0.95247	0.1305	0.75297
0.00587	0	0.00375	0	0	0.02264	0.00624	0.10388
0.00587	0	0.00375	0	0	0.02264	0.00624	0.10388
0.00587	0	0.00375	0	0	0.02264	0.00624	0.10388
0	0	0	0	0	0	0	0
0	0	0	0	0	0.01465	0.00624	0.00334
0.00033	0	0	0	0	0	0	0.00959
0.00553	0	0.00375	0	0	0.00799	0	0.09095
0.31134	0.08516	0.06794	0.0306	0.67977	0.92983	0.12426	0.64909
0.01039	0	0.00405	0	0	0	0	0.02459
0.01039	0	0.00405	0	0	0	0	0.02459
0.00153	0	0.00174	0	0	0	0	0.00286
0.00885	0	0.00231	0	0	0	0	0.02173
0	0	0	0	0	0	0	0.00051

0	0	0	0	0	0	0	0.00051
0	0	0	0	0	0	0	0.00044
0	0	0	0	0	0	0	0.00006
0.06295	0	0.04853	0	0.02375	0.00031	0.05898	0.01605
0.06295	0	0.04853	0	0.02375	0.00031	0.05898	0.01605
0	0	0	0	0	0	0	0
0.02677	0	0.04853	0	0	0	0	0.00053
0	0	0	0	0	0	0	0
0.03618	0	0	0	0.02375	0.00031	0.02464	0
0	0	0	0	0	0	0	0.01196
0	0	0	0	0	0	0.03435	0
0	0	0	0	0	0	0	0.00356
0	0	0	0	0	0	0	0
0	0	0	0	0	0	0	0
0	0	0	0	0	0	0	0
0	0	0	0	0.12224	0.03012	0.01494	0.00314
0	0	0	0	0.12224	0.03012	0.01494	0.00314
0	0	0	0	0	0	0	0
0	0	0	0	0.00174	0	0	0
0	0	0	0	0.00616	0	0	0
0	0	0	0	0.09115	0.02623	0	0
0	0	0	0	0.01321	0	0	0.00314
0	0	0	0	0.00075	0.00389	0	0
0	0	0	0	0	0	0	0
0	0	0	0	0.00024	0	0.01494	0
0	0	0	0	0.00899	0	0	0
0	0	0	0	0	0	0	0
0	0	0	0	0	0	0	0
0	0	0	0	0	0	0	0
0	0	0	0	0	0	0	0
0	0	0	0	0	0	0	0
0	0	0	0	0	0	0	0
0	0	0	0	0	0	0	0
0	0	0	0	0	0	0	0
0.23801	0.08516	0.01536	0.0306	0.53378	0.8994	0.05033	0.60481

0.23801	0.08516	0.01536	0.0306	0.53378	0.8994	0.05033	0.60481
0.0013	0	0	0	0.00125	0	0	0.00326
0.0027	0	0	0	0	0	0	0.0139
0	0	0	0.00084	0	0.01308	0	0.00609
0	0	0	0	0	0	0	0
0.0082	0	0	0	0	0	0	0.00639
0.00291	0	0.00078	0	0	0.01025	0.00164	0.03946
0	0	0	0	0	0	0	0
0	0	0.00029	0	0.00246	0	0.0009	0.00425
0.00274	0.00263	0	0.00042	0	0.00299	0.01366	0.08998
0	0	0	0	0	0	0	0
0.00686	0	0	0	0.00816	0.07429	0.00168	0.26853
0.18252	0.01388	0.00702	0.00447	0.07857	0.78482	0.00771	0.09598
0	0	0	0	0	0	0	0
0	0	0	0	0	0	0	0
0.01832	0.06865	0.00727	0.02487	0.44334	0.01372	0.02393	0.04544
0.00747	0	0	0	0	0	0.00081	0.03088
0	0	0	0	0	0	0	0.00067
0.00068	0	0	0	0	0.00025	0	0
0	0	0	0	0	0	0	0
0	0	0	0	0	0	0	0
0	0	0	0	0	0	0	0
0	0	0	0	0	0	0	0
0	0	0	0	0	0	0	0
0	0	0	0	0	0	0	0
0.00432	0	0	0	0	0	0	0
0	0	0	0	0	0	0	0
0	0	0	0	0	0	0	0
0	0	0	0	0	0	0	0
12.3848	17.79178	5.93406	7.34452	24.29731	8.04707	42.9008	16.43441
12.3848	17.79178	5.93406	7.34452	24.29731	8.04707	42.9008	16.43441
0.03136	0.00052	0.16329	0	0.00805	0	0	0
0.03136	0.00052	0.16329	0	0.00805	0	0	0
0.03136	0.00052	0.16329	0	0.00805	0	0	0
0	0	0	0	0.00639	0	0	0

0	0	0	0	0.00639	0	0	0
0	0	0	0	0.00639	0	0	0
0.35165	0.08354	0.22463	0.22177	0.36704	0.0363	0.41163	0.37802
0	0.04151	0.01665	0.09264	0	0	0	0
0	0.04151	0.01665	0.09264	0	0	0	0
0.19328	0.00247	0	0.00235	0.17176	0.0363	0.40824	0.37261
0	0	0	0	0	0	0	0
0	0.00092	0	0.00235	0	0	0	0.20448
0	0	0	0	0	0	0	0
0	0	0	0	0	0	0	0
0	0	0	0	0	0	0.0025	0
0	0	0	0	0	0	0	0
0	0	0	0	0	0	0	0
0	0.00155	0	0	0	0	0	0.16813
0	0	0	0	0	0	0	0
0.19328	0	0	0	0	0	0	0
0	0	0	0	0.00063	0	0.37741	0
0	0	0	0	0	0	0	0
0	0	0	0	0.16481	0.0363	0.02833	0
0	0	0	0	0.00632	0	0	0
0	0	0	0	0	0	0	0
0.14161	0.03956	0.20799	0.12678	0.19528	0	0.00339	0.00541
0	0	0	0	0	0	0	0
0.14161	0.03956	0.20799	0.12678	0.19528	0	0.00339	0.00541
0.01677	0	0	0	0	0	0	0
0.01677	0	0	0	0	0	0	0
0.00331	0	0.00085	0	0	0.00063	0.00228	0.01227
0.0031	0	0.00014	0	0	0.00063	0.00147	0.00328
0	0	0.00014	0	0	0.00063	0	0
0	0	0	0	0	0	0	0
0	0	0	0	0	0	0	0
0.0031	0	0	0	0	0	0.00147	0.00328
0.00021	0	0.00071	0	0	0	0.00081	0.00898
0.00021	0	0.00071	0	0	0	0.00081	0.00898
0.05226	0.00546	0.03779	0.00059	0.26222	0	0.04398	0

0.00007	0	0	0	0.0016	0	0	0
0.00007	0	0	0	0.0016	0	0	0
0	0	0	0	0.26062	0	0.02709	0
0	0	0	0	0.26062	0	0.02709	0
0.05219	0.00546	0.03779	0.00059	0	0	0	0
0.05219	0.00546	0.03779	0.00059	0	0	0	0
0	0	0	0	0	0	0.01689	0
0	0	0	0	0	0	0.01689	0
0.00638	0.00713	0.07005	0	2.34708	0.349	12.75672	0.08002
0	0.0015	0	0	0	0	0	0
0	0.0015	0	0	0	0	0	0
0	0	0	0	0	0	0	0
0	0	0	0	0	0	0	0
0.00638	0.00563	0.07005	0	2.34708	0.349	12.75672	0.08002
0	0	0	0	0	0	0	0
0	0	0	0	0	0	0	0.04778
0	0	0	0	0.81005	0.00032	0.18749	0
0	0	0	0	0.3126	0.08083	0.2962	0
0.00572	0.00563	0.07005	0	0	0	0	0
0	0	0	0	0.16371	0.13508	0.03127	0
0.00067	0	0	0	0	0	0	0
0	0	0	0	0.59539	0.07923	4.7244	0
0	0	0	0	0.12638	0	0.09977	0
0	0	0	0	0.33832	0.05354	7.40706	0.03224
0	0	0	0	0	0	0	0
0	0	0	0	0.00062	0	0.01053	0
0	0	0	0	0	0	0	0
0	0	0	0	0	0	0	0
6.03766	9.6741	1.44914	3.33139	12.84808	5.04252	20.62847	14.37203
0	0	0	0	0.05195	0.00497	0.13128	0.00882
0	0	0	0	0	0	0	0.00882
0	0	0	0	0.05195	0.00497	0.13128	0
0.16318	0.0038	0.17389	0	0	0	0	0.01593
0.16318	0.0038	0.17389	0	0	0	0	0.01593
3.85594	0.87613	0.5204	0.20179	0.21417	0.05784	0.07455	13.9139

0	0	0	0	0	0	0	0
0.01149	0	0	0	0	0	0	0.02219
0	0	0	0	0	0	0	0
0	0	0	0	0.11497	0.04398	0.03233	0
0.00616	0.0419	0.01524	0	0	0	0	0
0.84799	0.09155	0.12093	0.00201	0	0	0	0.03766
0.4993	0.71396	0.17881	0.0542	0.02837	0.00835	0.02253	0
2.491	0.02872	0.20542	0.14559	0	0	0	13.85405
0	0	0	0	0.07084	0.00551	0.0197	0
0	0	0	0	0	0	0	0.0093
0	0	0	0	0	0	0	0.0093
0	0	0	0	0.47566	0.20296	0.40962	0
0	0	0	0	0.27404	0.04397	0.15496	0
0	0	0	0	0.20162	0.159	0.25466	0
0	0	0	0	0.37415	0.45057	0.18378	0
0	0	0	0	0.16166	0.03187	0.09597	0
0	0	0	0	0.21249	0.4187	0.08781	0
0	0	0	0	0	0	0	0
0.00022	0	0	0	0.00174	0	0	0.00412
0	0	0	0	0	0	0	0
0.00022	0	0	0	0.00174	0	0	0.00412
0	0	0	0	0	0	0	0
0	0	0	0	0	0	0	0
0	0	0	0	0.427	0	0.07967	0
0	0	0	0	0.427	0	0.07967	0
0	0	0	0	0	0	0	0
0	0	0	0	0	0	0	0
1.6844	0.37847	0.67951	3.12927	0.19173	0.01197	0.04111	0.15574
0.07283	0.00702	0.00506	0	0.01011	0	0	0.076
0.03146	0	0	0	0.00724	0	0.00108	0
1.18462	0.21931	0.36375	3.0734	0.09564	0.01176	0.01035	0.02537
0.03067	0	0	0	0.04036	0.00022	0.02202	0.01791
0.17353	0.08866	0.13917	0.04753	0.00247	0	0.00287	0
0	0	0	0	0	0	0	0
0.18354	0.02549	0.02105	0.00166	0.01845	0	0.00145	0.0005

0	0	0	0	0	0	0	0
0.00035	0	0	0	0	0	0	0.00061
0.00741	0.038	0.15049	0.00668	0.01747	0	0.00334	0.03535
0	0	0	0	0	0	0	0
0	0	0	0	0	0	0	0
0	0	0	0	2.2525	0.14986	6.37713	0.17194
0.01629	0	0	0	6.47648	1.45433	9.34985	0
0	0	0	0	6.47648	1.45433	9.34985	0
0.01629	0	0	0	0	0	0	0
0	0	0	0	0	0	0	0
0	0	0	0	2.2525	0.14986	6.37713	0.17194
0	0	0	0	0	0	0	0
0	0	0	0	0	0	0	0
0	0	0	0	0	0	0	0
0	0	0	0	0	0	0	0
0	0	0	0	0	0	0	0
0	0	0	0	0	0	0	0
0	0	0	0	0	0	0	0
0	0	0	0	0	0	0	0
0	0	0	0	0	0	0	0
0.02082	8.39917	0.05361	0	2.38269	2.71001	3.98147	0.09228
0	0	0	0	0.68306	0.77422	2.70015	0
0	0	0	0	1.33286	1.88778	0.75823	0
0	1.24739	0.00516	0	0.00983	0	0.08696	0.09228
0	7.15179	0.04845	0	0.33085	0.02625	0.40406	0
0	0	0	0	0.00518	0	0.00735	0
0.02082	0	0	0	0.02091	0.02175	0.02472	0
0.08478	0	0	0	0	0	0	0
0.08478	0	0	0	0	0	0	0
0	0	0	0	0	0	0	0
0	0	0	0	0	0	0	0
0	0	0	0	0	0	0	0
0	0	0	0	0	0	0	0
0.21203	0.01652	0.02173	0.00033	0	0	0	0
0.21203	0.01652	0.02173	0.00033	0	0	0	0

0	0.00032	0.00553	0	0.95257	0	2.43865	0
0	0.00032	0.00553	0	0.95257	0	2.43865	0
0	0	0	0	0.95257	0	2.43865	0
0	0.00032	0.00553	0	0	0	0	0
0.0819	0.007	0.0049	0.12576	0.00023	0	0	1.5545
0	0	0	0	0	0	0	1.53009
0	0	0	0	0	0	0	1.53009
0	0	0	0	0	0	0	0
0	0	0	0	0	0	0	0
0.0819	0.00653	0.00467	0.12576	0.00023	0	0	0.00227
0.0819	0.00653	0.00467	0.12576	0.00023	0	0	0.00227
0	0	0	0	0	0	0	0.01076
0	0	0	0	0	0	0	0.01076
0	0	0	0	0	0	0	0
0	0	0	0	0	0	0	0
0	0	0.00023	0	0	0	0	0
0	0	0	0	0	0	0	0
0	0	0.00023	0	0	0	0	0
0	0.00047	0	0	0	0	0	0
0	0.00047	0	0	0	0	0	0
0	0	0	0	0	0	0	0.01137
0	0	0	0	0	0	0	0.01137
5.82027	8.01371	3.97787	3.66501	7.50566	2.61862	6.61908	0.03757
0.08047	0	0	0	0.20815	0.17452	0.13701	0
0.08047	0	0	0	0.20815	0.17452	0.13701	0
0	0.00094	0.01118	0	0	0	0	0
0	0.00094	0.01118	0	0	0	0	0
0	0	0.01847	0	0	0	0	0
0	0	0.01847	0	0	0	0	0
0.08324	0.01089	0.1533	0	0.08103	0.00234	0.0062	0.02189
0.08324	0.01089	0.1533	0	0.08103	0.00234	0.0062	0.02189
0.27502	5.43831	3.337	2.86209	4.1306	0.66397	3.06267	0
0.27502	5.43831	3.337	2.86209	4.1306	0.66397	3.06267	0
4.43378	0.42144	0.13624	0.02839	0.0766	0.48245	0.03818	0.01569
4.43378	0.42144	0.13624	0.02839	0.0766	0.48245	0.03818	0.01569

0	0	0	0	0	0	0	0
0	0	0	0	0.08838	0.43965	0.25994	0
0	0	0	0	0.08838	0.43965	0.25994	0
0	0.00162	0.00015	0	0.01378	0	0.0009	0
0	0.00162	0.00015	0	0.01378	0	0.0009	0
0	0	0	0	0	0	0	0
0	0	0	0	0	0	0	0
0	0	0	0	0	0	0	0
0.29356	0	0	0	0.04536	0	0	0
0.00782	0	0	0	0.00481	0	0	0
0	0	0	0	0.00512	0	0	0
0	0	0	0	0	0	0	0
0.28575	0	0	0	0	0	0	0
0	0	0	0	0.03543	0	0	0
0	2.03779	0.03638	0.77454	2.82565	0.85502	3.11418	0
0	0	0	0	1.57878	0.04185	0.05854	0
0	2.03609	0.03638	0.77454	0.93111	0.59693	1.35532	0
0	0.0017	0	0	0.00096	0	0	0
0	0	0	0	0.3148	0.21624	1.70033	0
0	0	0	0	0	0	0	0
0.6542	0.10274	0.28515	0	0.0361	0.00067	0	0
0.6542	0.10274	0.28515	0	0.0361	0.00067	0	0
1.17492	0.14509	0.19867	0.09701	0.04841	0.00295	0.00044	0.45842
1.17492	0.14509	0.19867	0.09701	0.04841	0.00295	0.00044	0.45842
1.17492	0.14509	0.19867	0.09701	0.04841	0.00295	0.00044	0.45842
0	0	0	0	0	0	0	0.04902
0	0	0	0	0	0	0	0.04902
0	0	0	0	0	0	0	0
0	0	0	0	0	0	0	0
0	0	0	0	0	0	0	0
0	0	0	0	0	0	0	0
0	0	0.00322	0	0.00387	0	0	0
0	0	0.00322	0	0.00387	0	0	0
0.12819	0	0	0	0	0	0	0.0063
0.12819	0	0	0	0	0	0	0.0063

0	0	0	0	0	0	0	0
0	0	0	0	0	0	0	0
1.04673	0.1407	0.15022	0.09701	0.02671	0.00216	0.00044	0.4014
0.16637	0.00268	0.01164	0.08688	0.00647	0.00216	0.00044	0.19299
0	0.00395	0.00891	0	0	0	0	0
0.88035	0.13407	0.12968	0.01014	0.02024	0	0	0.20841
0	0.00438	0.04523	0	0	0	0	0
0	0.00438	0.04523	0	0	0	0	0
0	0	0	0	0	0.00079	0	0
0	0	0	0	0	0.00079	0	0
0	0	0	0	0	0	0	0
0	0	0	0	0	0	0	0
0	0	0	0	0	0	0	0
0	0	0	0	0	0	0	0
0	0	0	0	0.01784	0	0	0
0	0	0	0	0.01784	0	0	0
0	0	0	0	0	0	0	0
0	0	0	0	0	0	0	0
0	0	0	0	0	0	0	0.0017
0	0	0	0	0	0	0	0.0017
0	0	0	0	0	0	0	0
0	0	0	0	0	0	0	0
0	0.00524	0.004	0	2.68479	0.47845	0.35885	0
0	0.00524	0.004	0	2.68479	0.47845	0.35885	0
0	0.00524	0.004	0	2.68479	0.47845	0.35885	0
0	0.00524	0.004	0	2.68479	0.47845	0.35885	0
0	0	0	0	0	0	0	0
0	0	0	0	0	0	0	0
0	0	0	0	0	0	0	0
0	0	0	0	0	0	0	0
0	0	0	0	0	0	0	0
0	0	0	0	2.68479	0.47845	0.35821	0
0	0.00524	0.004	0	0	0	0	0
0	0	0	0	0	0	0.00063	0
17.75986	4.30969	0.67704	2.08261	1.22879	1.53076	0.90588	0.098

8.39281	4.30757	0.67283	2.07957	0.76987	1.33075	0.87283	0.00968
8.39281	4.30757	0.67283	2.07957	0.76987	1.33075	0.87283	0.00968
4.64314	3.30966	0.18926	0	0	0	0	0
4.64314	3.30966	0.18926	0	0	0	0	0
0	0	0	0	0	0	0	0
3.74967	0.99791	0.48356	2.07957	0.76987	1.33075	0.87283	0.00968
3.74967	0.99791	0.48356	2.07957	0.76987	1.33075	0.87283	0.00968
0	0	0	0	0	0	0	0
8.97081	0	0	0	0	0.00015	0	0
8.97081	0	0	0	0	0.00015	0	0
8.97081	0	0	0	0	0	0	0
8.2078	0	0	0	0	0	0	0
0.76301	0	0	0	0	0	0	0
0	0	0	0	0	0	0	0
0	0	0	0	0	0	0	0
0	0	0	0	0	0.00015	0	0
0	0	0	0	0	0	0	0
0	0	0	0	0	0.00015	0	0
0	0	0	0	0	0	0	0
0	0	0	0	0	0	0	0
0.39624	0.00212	0.00421	0.00304	0.45891	0.19986	0.03306	0.08832
0.39624	0.00212	0.00421	0.00304	0.45891	0.19986	0.03306	0.08832
0	0.00113	0	0	0	0	0	0
0	0.00113	0	0	0	0	0	0
0	0.0002	0.00084	0	0	0	0	0
0	0.0002	0.00084	0	0	0	0	0
0	0	0	0	0	0	0.03132	0
0	0	0	0	0	0	0.03132	0
0	0	0	0	0	0	0	0
0	0	0	0	0	0	0	0
0.00176	0	0	0	0.00286	0.10962	0	0
0	0	0	0	0	0	0	0
0	0	0	0	0	0	0	0
0.00176	0	0	0	0.00286	0.10962	0	0
0	0	0	0	0	0	0	0

0	0	0	0	0	0	0	0
0.39448	0.00079	0.00337	0.00304	0.45605	0.09024	0.00174	0.08832
0.00147	0.00079	0	0	0.22497	0.00517	0.00052	0.01403
0.02695	0	0	0.00073	0.02386	0.0063	0	0
0.00998	0	0	0	0.00781	0.00709	0.00028	0
0.30761	0	0.00337	0.00231	0.15991	0.03191	0	0.0738
0	0	0	0	0	0	0	0.00049
0.00348	0	0	0	0	0.03713	0.00031	0
0.04412	0	0	0	0.01898	0.00222	0.00063	0
0.00087	0	0	0	0.02052	0.00042	0	0
0	0	0.00736	0	0.00503	0.0259	0.00955	0
0	0	0.00736	0	0.00503	0.0259	0.00955	0
0	0	0.00736	0	0.00503	0.0259	0.00955	0
0	0	0	0	0	0	0	0
0	0	0	0	0	0	0	0
0	0	0	0	0	0	0	0
0	0	0	0	0	0	0	0
0	0	0	0	0	0	0	0
0	0	0.00736	0	0.00503	0.0259	0.00955	0
0	0	0.00736	0	0.00503	0.0259	0.00955	0
0	0	0	0	0	0	0	0
0	0	0	0	0	0	0	0
0	0	0	0	0	0	0	0
0	0	0	0	0	0	0	0
0	0	0	0	0	0	0	0
0.21262	0.2972	0.2305	0	0.0013	0.002	0	0
0.21262	0.2972	0.2305	0	0.0013	0.002	0	0
0.21262	0.2972	0.2305	0	0.0013	0.002	0	0
0.21262	0.2972	0.2305	0	0.0013	0.002	0	0
0	0	0	0	0	0	0	0
0	0	0	0	0	0	0	0
0.21262	0.2972	0.2305	0	0.0013	0.002	0	0
0	0	0	0	0	0	0	0
0	0	0	0	0	0	0	0
0	0	0	0	0	0	0	0

0	0	0	0	0.0013	0.00185	0	0
0	0	0	0	0	0.00015	0	0
0	0	0	0	0	0	0	0
0.00331	0	0	0	0	0	0	0
0.20931	0.2972	0.2305	0	0	0	0	0
0	0	0	0	0	0	0	0
0	0	0	0	0	0	0	0
0	0	0	0	0	0	0	0
0	0	0	0	0	0	0	0
0	0	0	0	0	0	0	0
0	0	0	0	0	0	0	0
0	0	0	0	0	0	0	0
0	0	0	0	0	0	0	0
0	0	0	0	0	0	0	0
0	0	0	0	0	0	0	0
0	0	0	0	0	0	0	0
0	0	0	0	0	0	0	0
0	0	0	0	0	0	0	0
2.94511	0.2715	0.40377	3.15687	19.71423	37.05803	20.21436	73.91247
0.128	0.01124	0.00197	0	0.10201	0	0	0
0.128	0.01124	0.00197	0	0.10201	0	0	0
0.02036	0	0	0	0.00006	0	0	0
0.02036	0	0	0	0.00006	0	0	0
0.02036	0	0	0	0.00006	0	0	0
0	0	0	0	0	0	0	0
0	0	0	0	0	0	0	0
0	0	0	0	0	0	0	0
0	0.01124	0.00197	0	0.10194	0	0	0
0	0.01124	0.00197	0	0.10194	0	0	0
0	0.01124	0.00197	0	0.10194	0	0	0
0.10764	0	0	0	0	0	0	0
0	0	0	0	0	0	0	0
0	0	0	0	0	0	0	0
0.10764	0	0	0	0	0	0	0
0.10764	0	0	0	0	0	0	0

0	0	0	0	0	0	0	0
0	0	0	0	0	0	0	0
0	0	0	0	0	0	0	0
0	0	0	0	0	0	0	0
0	0	0	0	0	0	0	0
0	0	0	0	0	0	0	0
0	0	0	0	0	0	0	0
0	0	0	0	0	0	0	0
0	0	0	0	0	0	0	0
0	0	0	0	0	0	0	0
0	0	0	0	0	0	0	0
0	0	0	0	0	0	0	0
1.40267	0.10144	0.01348	0.00576	0.07268	0	0	0.00533
1.40267	0.10144	0.01348	0.00576	0.07268	0	0	0.00533
1.40267	0.10144	0.01348	0.00576	0.07268	0	0	0.00533
1.40267	0.10144	0.01348	0.00576	0.07268	0	0	0.00533
1.40267	0.10144	0.01348	0.00576	0.07268	0	0	0.00533
0	0	0	0	0	0	0	0
0	0	0	0	0	0	0	0
0	0	0	0	0	0	0	0
0	0	0	0	0	0.05085	0	0
0	0	0	0	0	0.05085	0	0
0	0	0	0	0	0.05085	0	0
0	0	0	0	0	0.05085	0	0
0	0	0	0	0	0.04667	0	0
0	0	0	0	0	0	0	0
0	0	0	0	0	0	0	0
0	0	0	0	0	0.00255	0	0
0	0	0	0	0	0.00162	0	0
0	0	0	0	0	0	0	0
0	0	0	0	0	0	0	0
0	0	0	0	0	0	0	0
0	0	0	0	0	0	0	0
1.41443	0.15882	0.38832	3.1511	19.53955	37.00718	20.21436	73.90715
0	0	0	0	0	0	0.1285	0

0	0	0	0	0	0	0.1285	0
0	0	0	0	0	0	0.1285	0
0	0	0	0	0	0	0.12207	0
0	0	0	0	0	0	0.00643	0
0	0	0	0	0	0	0	0
0	0	0	0	0	0	0	0
0	0	0	0	0	0	0	0
0	0	0	0	0	0	0	0
0	0	0	0	0	0	0	0
0	0	0	0	0	0	0	0
0	0	0	0	0	0	0	0
0	0	0	0	0	0	0	0
0	0	0	0	0	0	0	0
1.40848	0.15882	0.38832	3.1511	19.52608	36.99023	20.08586	73.90715
0	0	0	0	0	0	0	0
0	0	0	0	0	0	0	0
0	0	0	0	0	0	0	0
1.40848	0.15882	0.38832	3.1511	19.51728	36.96377	19.94625	73.88698
0	0	0	0	0	0	0	0
0	0	0	0	0	0	0	0
0	0	0	0	0	0	0	0
0	0	0	0	0	0	0	0
0	0	0	0	0	0	0	0
0	0	0	0	0	0	0	0
0	0	0	0	0	0	0	0
0	0	0	0	0	0	0	0
0	0	0	0	0	0	0	0
0	0	0	0	0	0	0	0
0	0	0	0	0	0	0	0
0	0	0	0	0	0	0	0
0	0.00193	0	0	0	0	0	0
0	0.00193	0	0	0	0	0	0
0	0.00661	0	0	0	0	0	6.36428
0	0	0	0	0	0	0	0
0	0.00661	0	0	0	0	0	6.36428

0	0	0	0	0	0	0	0
1.40398	0.06569	0.27428	3.1511	14.67164	20.70082	19.71883	21.65841
1.40398	0.06569	0.27428	3.1511	14.67164	20.70082	19.71883	21.65841
0	0	0	0	0	0	0	0
0.00451	0.08459	0.11404	0	4.84564	16.26296	0.22742	45.86429
0	0	0	0	0	0	0	0
0	0	0	0	0	0	0.001	0.01857
0	0	0	0	0	0	0.00159	0.00183
0.00287	0.04289	0.0772	0	4.21858	14.1969	0.19234	38.54718
0	0.02934	0.01364	0	0.36883	1.07975	0.01874	4.77202
0.00163	0.01235	0.02319	0	0.25823	0.9863	0.01374	2.52469
0	0	0	0	0	0	0	0
0	0	0	0	0	0	0	0
0	0	0	0	0	0	0	0
0	0	0	0	0	0	0	0
0	0	0	0	0	0	0	0
0	0	0	0	0	0	0	0
0	0	0	0	0	0	0	0
0	0	0	0	0	0	0	0
0	0	0	0	0	0	0	0
0	0	0	0	0	0	0	0
0	0	0	0	0	0	0	0
0	0	0	0	0.0088	0.02645	0.13961	0.02017
0	0	0	0	0.00739	0.02645	0.09275	0.02017
0	0	0	0	0.00739	0.02645	0.09275	0.02017
0	0	0	0	0.00141	0	0.04686	0
0	0	0	0	0.00141	0	0.04686	0
0.00595	0	0	0	0.01348	0.01695	0	0
0.00595	0	0	0	0.01348	0.01695	0	0
0	0	0	0	0	0	0	0
0	0	0	0	0	0	0	0
0	0	0	0	0	0	0	0
0.00595	0	0	0	0.01348	0.01695	0	0
0	0	0	0	0	0	0	0
0	0	0	0	0	0	0	0

0.00437	0	0	0	0.01348	0.01695	0	0
0	0	0	0	0	0	0	0
0	0	0	0	0	0	0	0
0.00158	0	0	0	0	0	0	0
0	0	0	0	0	0	0	0
0	0	0	0	0	0	0	0
0	0	0	0	0	0	0	0
0	0	0	0	0	0	0	0
0	0	0	0	0	0	0	0
0	0	0	0	0	0	0	0
0	0	0	0	0	0	0	0
0	0	0	0	0	0	0	0
0	0	0	0	0	0	0	0
0	0	0	0	0	0	0	0
0	0	0	0	0	0	0	0
0	0	0	0	0	0	0	0
0	0	0	0	0	0	0	0
0.00086	0	0	0	0.06936	0	0.04993	0
0.00086	0	0	0	0.06936	0	0.04993	0
0.00086	0	0	0	0.06936	0	0.04993	0
0.00086	0	0	0	0.06936	0	0.04993	0
0.00086	0	0	0	0.03932	0	0.01492	0
0	0	0	0	0.03932	0	0.01492	0
0.00086	0	0	0	0	0	0	0
0	0	0	0	0	0	0	0
0	0	0	0	0	0	0	0
0	0	0	0	0.03004	0	0.03501	0
0	0	0	0	0.02984	0	0.03461	0
0	0	0	0	0.0002	0	0.0004	0
0	0	0	0	0	0	0	0
0	0	0	0	0	0	0	0
0	0	0	0	0	0	0	0
0	0	0	0	0	0	0	0
0	0	0	0	0	0	0	0
0	0	0	0	0	0	0	0

0	0.75058	11.2226	2.02569	0.56481	0	0.09975	0
0	0.75058	11.2226	2.02569	0.56481	0	0.09975	0
0	0.75058	11.2226	2.02569	0.56481	0	0.09975	0
0	0.75058	11.2226	2.02569	0.56481	0	0.09975	0
0	0.75058	11.2226	2.02569	0.56481	0	0.09975	0
0	0.75058	11.2226	2.02569	0.56481	0	0.09975	0
0	0	0	0	0.96136	0.06555	0.0526	0
0	0	0	0	0.96136	0.06555	0.0526	0
0	0	0	0	0.96136	0.06555	0.0526	0
0	0	0	0	0.96136	0.06555	0.0526	0
0	0	0	0	0.96136	0.06555	0.0526	0
0	0	0	0	0.96136	0.06555	0.0526	0
0	0	0	0	0	0	0	0
0	0	0	0	0.96136	0.06555	0.0526	0
0	0	0	0	0	0	0	0
0	0	0	0	0	0	0	0
0	0	0	0	0	0.01742	0	0
0	0	0	0	0	0.01742	0	0
0	0	0	0	0	0.01742	0	0
0	0	0	0	0	0.01742	0	0
0	0	0	0	0	0	0	0
0	0	0	0	0	0	0	0
0	0	0	0	0	0	0	0
0	0	0	0	0	0.01742	0	0
0	0	0	0	0	0.01742	0	0
0	0	0	0	0	0.01742	0	0

AP008_2	AP008_3	AP009_1	AP009_2	AP009_3	AP010_1	AP010_2	AP010_3
99.9969	100	100	100	100	100	100	100
0.31568	0.73123	0.44516	0.68339	0.14243	2.2669	0.6649	1.03975
0.28252	0.52753	0.00493	0.02876	0.13426	1.83125	0.06569	1.02808
0.03293	0.31417	0.00043	0	0.04947	0	0	0.0124
0.03293	0.31417	0.00043	0	0.04947	0	0	0.0124
0	0	0	0	0	0	0	0
0	0	0	0	0	0	0	0
0.03293	0.31417	0.00043	0	0.04947	0	0	0.0124
0	0	0	0	0	0	0	0
0	0.02268	0.00043	0	0.00479	0	0	0.00032
0	0.00018	0	0	0	0	0	0
0	0.00098	0	0	0	0	0	0
0	0.00329	0	0	0	0	0	0
0.00043	0.01105	0	0	0	0	0	0
0.00979	0.03153	0	0	0.0024	0	0	0
0.00123	0.00353	0	0	0	0	0	0
0.00026	0.00671	0	0	0	0	0	0
0	0.00173	0	0	0	0	0	0
0.00311	0.20816	0	0	0.04009	0	0	0.01208
0.01738	0.01543	0	0	0.00013	0	0	0
0.00011	0	0	0	0	0	0	0
0	0	0	0	0.00205	0	0	0
0	0.0034	0	0	0	0	0	0
0.00064	0	0	0	0	0	0	0
0	0.0055	0	0	0	0	0	0
0	0	0	0	0	0	0	0
0.11219	0.15259	0.0045	0.01006	0.00797	1.83125	0.06569	1.01377
0.11219	0.15259	0.0045	0.01006	0.00797	1.83125	0.06569	1.01377
0	0	0	0	0	0	0	0
0	0	0	0	0	0	0	0
0	0	0	0	0	0	0	0
0	0	0	0	0	0	0	0
0.11219	0.1521	0.00405	0.0007	0.00673	1.83125	0.06569	1.01377

0	0	0	0	0	0	0	0
0	0	0	0	0	0	0	0
0	0	0	0	0	0	0	0
0	0	0	0	0	0.00383	0.0034	0.00167
0.00377	0	0.00405	0.0007	0.00673	0	0	0
0.10842	0.1521	0	0	0	0.47578	0.06229	0.37441
0	0	0	0	0	1.35164	0	0.63769
0	0	0	0	0	0	0	0
0	0	0	0	0	0	0	0
0	0.00049	0.00044	0.00936	0.00124	0	0	0
0	0.00049	0.00044	0.00936	0.00124	0	0	0
0.00054	0.00546	0	0	0	0	0	0
0.00054	0.00546	0	0	0	0	0	0
0.00054	0.00546	0	0	0	0	0	0
0.00054	0.0019	0	0	0	0	0	0
0	0.00356	0	0	0	0	0	0
0	0	0	0	0	0	0	0
0.13559	0.05531	0	0.00871	0.07632	0	0	0.00165
0.13559	0.05531	0	0.00871	0.07632	0	0	0.00165
0.13559	0.05531	0	0.00871	0.07632	0	0	0.00165
0.01049	0.00612	0	0	0	0	0	0
0.00879	0.00149	0	0	0	0	0	0
0.11631	0.04771	0	0.00871	0.07632	0	0	0.00165
0.00126	0	0	0.00999	0.0005	0	0	0.00026
0.00126	0	0	0.00999	0.0005	0	0	0.00026
0	0	0	0.00999	0.0005	0	0	0
0	0	0	0.00999	0.0005	0	0	0
0.00126	0	0	0	0	0	0	0.00026
0.00126	0	0	0	0	0	0	0.00026
0.03316	0.20369	0.44023	0.65462	0.00817	0.43564	0.5992	0.01167
0.00622	0.19343	0.41758	0.46083	0.00684	0	0	0
0.00622	0.00753	0.03477	0.01931	0.00134	0	0	0
0.00622	0.00753	0	0.00657	0.00134	0	0	0
0	0	0	0.00034	0	0	0	0
0.00531	0.00719	0	0.00335	0	0	0	0

0.00092	0.00034	0	0.00287	0.00134	0	0	0
0	0	0.03477	0.01275	0	0	0	0
0	0	0.03477	0.01275	0	0	0	0
0	0	0	0	0	0	0	0
0	0.1859	0.38282	0.44152	0.00549	0	0	0
0	0.1859	0.3824	0.44152	0.00549	0	0	0
0	0.1853	0.37587	0.43903	0.00549	0	0	0
0	0	0.00153	0	0	0	0	0
0	0.0006	0.00501	0.00249	0	0	0	0
0	0	0.00041	0	0	0	0	0
0	0	0.00041	0	0	0	0	0
0	0	0	0	0	0	0	0
0.02694	0.01026	0.02265	0.19379	0.00133	0.43564	0.5992	0.01167
0.02694	0.01026	0.02265	0.19379	0.00133	0.43564	0.5992	0.01167
0	0	0	0	0	0	0	0
0	0	0	0	0	0	0	0
0	0	0	0	0	0	0	0
0	0	0	0	0	0	0	0
0.02558	0.01009	0.02045	0.17613	0.00133	0.39885	0.54314	0.01075
0.02558	0.01009	0.02045	0.17613	0.00133	0.39885	0.54314	0.01075
0	0	0	0	0	0	0	0
0	0	0	0	0	0	0	0
0.00136	0.00017	0.0022	0.01766	0	0.03679	0.05607	0.00092
0.00136	0.00017	0.0022	0.01766	0	0.03679	0.05607	0.00092
0	0	0	0	0	0	0	0
0	0	0	0	0	0	0	0
45.78106	47.03871	75.51641	66.54853	74.3095	74.38019	9.53912	74.47275
0	0	0	0.00068	0	0	0	0
0	0	0	0.00068	0	0	0	0
0	0	0	0.00068	0	0	0	0
0	0	0	0.00068	0	0	0	0
0	0	0	0.00068	0	0	0	0
45.78106	47.03871	75.51641	66.54785	74.3095	74.38019	9.53912	74.47275
45.78106	47.03871	75.51641	66.54785	74.3095	74.38019	9.53912	74.47275
37.67776	43.65985	64.73291	35.83918	70.51413	70.15312	8.91578	72.81032

37.67776	43.65985	64.73291	35.83918	70.51413	70.15312	8.91578	72.81032
2.149	0.78539	9.85985	3.91051	3.1756	8.63981	0.01044	0.7105
0	0	0.00005	0	0	0.10827	0	0.0003
0	0	0	0	0	0	0	0
0.00027	0.00108	28.31782	22.0599	25.00863	0.01753	0.00114	0.00039
0	0	0	0	0	0	0	0
0	0	0	0	0	0	0	0
0	0	0	0	0	0	0	0
0	0	0	0	0	0	0	0
0.24251	0.02725	0	0	0	0.01803	5.04987	0.00652
0	0	0	0	0	0	0	0
0	0	0	0	0	0	0	0
0	0	0.00154	0.00306	0	0.00306	0	0
0.30108	0.65188	0.05842	0.16722	0.20647	0.58813	0.03801	3.75408
0.00049	0	13.91664	4.79767	34.27655	0	0	0
0	0	0.36644	0.42147	0.11936	0	0	0
0	0	0	0	0	0	0	0
0	0	0	0	0	0	0	0
0.63681	2.89672	6.78916	2.0138	1.20965	0	0	0.00012
9.18465	3.76019	0.71991	1.30477	1.10977	1.72649	0.57508	0.6861
1.02793	11.68619	1.47918	0.69824	2.40853	8.60275	0.25266	13.47783
21.86552	22.89445	0.00313	0.003	0.01693	48.8919	2.73479	49.51087
2.2695	0.95669	3.22076	0.45953	2.98265	1.55714	0.25378	4.66362
0	0	0	0	0	0	0	0
0	0	0	0	0	0	0	0
0	0	0	0	0	0	0	0
0	0	0	0	0	0	0	0
0	0	0	0	0	0	0	0
0	0	0	0	0	0	0	0
0	0	0.84888	3.84677	0.02576	0	0	0
0	0	0.52342	0.82778	0.00946	0	0	0
0	0	0.23957	0.28062	0.00136	0	0	0
0	0	0.28385	0.54715	0.0081	0	0	0
0	0	0.32546	3.01899	0.0163	0	0	0
0	0	0	0	0	0	0	0

0	0	0.32546	3.01899	0.0163	0	0	0
0	0	0	0	0	0	0	0
0	0	0	0	0	0	0	0
0	0	0	0	0	0	0	0
0	0	0	0	0	0	0	0
0	0	0	0	0	0	0	0
0	0	0	0	0	0	0	0
0	0	0	0	0	0	0	0
0.023	0	0.07004	0.53759	0	0.51763	0.12407	0.00146
0.00205	0	0	0	0	0	0	0
0.00205	0	0	0	0	0	0	0
0	0	0	0	0	0	0	0
0	0	0	0	0	0	0	0
0	0	0	0	0	0	0	0
0.02095	0	0.07004	0.53759	0	0.51763	0.12407	0.00146
0	0	0.06923	0.46288	0	0.48865	0.12407	0.00146
0.00137	0	0	0	0	0	0	0
0	0	0	0	0	0.01578	0	0
0	0	0	0	0	0	0	0
0	0	0	0	0	0	0	0
0	0	0	0	0	0	0	0
0	0	0	0	0	0	0	0
0	0	0	0	0	0	0	0
0	0	0	0	0	0	0	0
0	0	0	0	0	0	0	0
0	0	0	0	0	0	0	0
0	0	0	0	0	0	0	0
0.01958	0	0	0	0	0	0	0
0	0	0	0	0	0	0	0
0	0	0	0	0	0	0	0
0	0	0	0	0	0	0	0
0	0	0	0	0	0	0	0
0	0	0.00081	0.07471	0	0.0132	0	0
0	0.23726	6.72503	20.74238	0.37001	0	0	0.00021
0	0.23726	6.72503	20.74238	0.37001	0	0	0.00021

0	0	1.04804	13.13169	0.29655	0	0	0
0	0	0.01069	0.0338	0	0	0	0
0	0	0	0	0	0	0	0
0	0.23726	5.61924	7.41939	0.07345	0	0	0.00021
0	0	0.04706	0.15751	0	0	0	0
0	0	0	0	0	0	0	0
8.0803	3.14159	3.13955	5.58192	3.39961	3.70944	0.49927	1.66076
8.0803	3.14159	3.13955	5.58192	3.39961	3.70944	0.49927	1.66076
3.51147	1.59405	1.42923	2.81444	1.92789	3.70944	0.49927	1.66029
0	0	0.70727	0.90039	0.01845	0	0	0
0.5374	0.00248	0.00068	0.00102	0.00322	0	0	0
0	0	0	0	0	0	0	0
4.03144	1.54506	1.00238	1.86608	1.45005	0	0	0.00047
0	0	0	0	0	0	0	0
0	0	0	0	0	0	0	0
0	0	0	0	0	0	0	0
0	0	0	0	0	0	0	0
0	0	0	0	0	0	0	0
0	0	0	0	0	0	0	0
0	0	0	0	0	0	0	0
18.94166	13.96532	19.13772	28.27318	14.11741	17.83914	54.36645	17.80218
3.92148	0.34855	0.22273	0.07594	0.69815	0.13557	0.83264	0.30868
0.20093	0.04857	0.00119	0.00036	0.00974	0.00392	0	0.00657
0.20093	0.04857	0.00119	0.00036	0.00974	0.00392	0	0.00657
0.20093	0.04857	0.00119	0.00036	0.00974	0.00392	0	0.00657
0	0	0	0.00036	0	0	0	0
0.01263	0.00786	0	0	0	0.00124	0	0
0.13447	0.00727	0	0	0	0	0	0
0.05383	0.03343	0.00119	0	0.00974	0.00267	0	0.00657
3.72055	0.29999	0.22154	0.07557	0.68841	0.13166	0.83264	0.30212
0.09713	0.01625	0	0.00417	0	0	0	0.00038
0.09713	0.01625	0	0.00417	0	0	0	0.00038
0.03756	0.0023	0	0.00174	0	0	0	0.00028
0.05958	0.01395	0	0.00243	0	0	0	0.0001
0.00979	0.00029	0	0	0	0	0	0

0.00979	0.00029	0	0	0	0	0	0
0.00123	0	0	0	0	0	0	0
0.00856	0.00029	0	0	0	0	0	0
0.7414	0	0	0.02523	0	0	0.83264	0
0.7414	0	0	0.02523	0	0	0.83264	0
0	0	0	0	0	0	0	0
0.00595	0	0	0	0	0	0.7833	0
0	0	0	0	0	0	0	0
0.73545	0	0	0.02523	0	0	0.04934	0
0	0	0	0	0	0	0	0
0	0	0	0	0	0	0	0
0	0	0	0	0	0	0	0
0	0	0	0	0	0	0	0
0	0	0	0	0	0	0	0
0	0	0	0	0	0	0	0
0.03579	0	0.00418	0	0.03084	0	0	0
0.03579	0	0.00418	0	0.03084	0	0	0
0	0	0	0	0.01034	0	0	0
0	0	0	0	0.00762	0	0	0
0	0	0	0	0	0	0	0
0	0	0	0	0	0	0	0
0	0	0	0	0	0	0	0
0	0	0.00418	0	0.00868	0	0	0
0.03579	0	0	0	0	0	0	0
0	0	0	0	0	0	0	0
0	0	0	0	0.00421	0	0	0
0.00385	0	0	0	0	0	0	0
0.00076	0	0	0	0	0	0	0
0.00032	0	0	0	0	0	0	0
0.00044	0	0	0	0	0	0	0
0.00309	0	0	0	0	0	0	0
0	0	0	0	0	0	0	0
0	0	0	0	0	0	0	0
0.00309	0	0	0	0	0	0	0
2.83258	0.28345	0.21736	0.04617	0.65757	0.13166	0	0.30174

2.83258	0.28345	0.21736	0.04617	0.65757	0.13166	0	0.30174
0.04583	0.00115	0	0.03116	0.0193	0	0	0.00034
0.00524	0.00233	0	0	0.02827	0	0	0.00573
0.03657	0.01465	0	0	0.0096	0	0	0.00022
0	0	0	0	0	0	0	0
0.32619	0.00293	0	0	0.03567	0	0	0
0.17348	0.03179	0	0.0017	0.04354	0	0	0.00759
0	0	0	0	0	0	0	0
0.00358	0	0	0	0	0	0	0
0.2843	0.08707	0.00141	0	0.05074	0.0031	0	0.0406
0	0	0	0	0	0	0	0
0.30773	0.01417	0	0	0.13931	0	0	0.01373
1.41999	0.03625	0.01146	0.00168	0.21573	0.031	0	0.01175
0	0	0	0	0	0.00378	0	0
0	0	0	0	0	0	0	0
0.15488	0.06103	0.06647	0.00479	0.06308	0.08482	0	0.21813
0.05478	0.03207	0	0	0.02342	0	0	0
0	0	0	0	0	0	0	0
0.00055	0	0	0	0.02791	0.00067	0	0
0	0	0	0	0	0	0	0.00365
0.00127	0	0	0	0	0	0	0
0.00434	0	0	0	0	0	0	0
0	0	0	0	0	0	0	0
0.00171	0	0	0	0	0	0	0
0	0	0	0	0.00101	0	0	0
0.00105	0	0	0	0	0	0	0
0	0	0.13802	0.00685	0	0	0	0
0	0	0	0	0	0.00828	0	0
0.01108	0	0	0	0	0	0	0
8.03269	12.49652	17.6778	23.88236	10.3836	14.9567	49.08111	13.15026
8.03269	12.49652	17.6778	23.88236	10.3836	14.9567	49.08111	13.15026
0	0	0	0.00244	0	0	0	0
0	0	0	0.00244	0	0	0	0
0	0	0	0.00244	0	0	0	0
0	0	0	0	0	0	0	0

0	0	0	0	0	0	0	0
0	0	0	0	0	0	0	0
0.71177	9.16752	0.67858	5.75836	0.04339	0.60353	26.20134	0.3946
0	0	0	0	0	0	0	0
0	0	0	0	0	0	0	0
0.60159	9.16353	0.48727	0.33318	0.03573	0.09905	0.15282	0.38858
0	0	0	0	0	0	0	0
0.48713	0	0	0	0	0.09905	0.15282	0.06548
0	0	0	0	0	0	0	0
0	0	0	0	0.02111	0	0	0
0	8.32245	0	0	0	0	0	0.00048
0	0	0	0	0	0	0	0
0	0.00599	0	0	0.00488	0	0	0.05948
0.11446	0.83355	0	0	0.00802	0	0	0.26313
0	0	0.00566	0	0	0	0	0
0	0.00155	0.15703	0.21287	0	0	0	0
0	0	0	0	0	0	0	0
0	0	0	0	0	0	0	0
0	0	0.32458	0.12031	0.00172	0	0	0
0	0	0	0	0	0	0	0
0	0	0	0	0	0	0	0
0.11018	0.004	0.19131	5.42519	0.00766	0.50447	26.04851	0.00603
0	0	0	0	0	0	0	0
0.11018	0.004	0.19131	5.42519	0.00766	0.50447	26.04851	0.00603
0	0	0	0	0	0	0	0
0	0	0	0	0	0	0	0
0.0021	0.0217	0	0	0.00201	0	0	0
0.0021	0.01474	0	0	0.00094	0	0	0
0.0021	0.0008	0	0	0	0	0	0
0	0	0	0	0	0	0	0
0	0	0	0	0	0	0	0
0	0.01394	0	0	0.00094	0	0	0
0	0.00696	0	0	0.00107	0	0	0
0	0.00696	0	0	0.00107	0	0	0
0	0	0.02229	0.00932	0	0	0	0

0	0	0	0.00242	0	0	0	0
0	0	0	0.00242	0	0	0	0
0	0	0	0	0	0	0	0
0	0	0	0	0	0	0	0
0	0	0.02229	0.0069	0	0	0	0
0	0	0.02229	0.0069	0	0	0	0
0	0	0	0	0	0	0	0
0	0	0	0	0	0	0	0
0	1.44057	0.82252	0.08648	0.05182	0.22206	0.11286	0
0	0	0	0	0	0	0	0
0	0	0	0	0	0	0	0
0	0	0	0	0	0	0	0
0	0	0	0	0	0	0	0
0	1.44057	0.82252	0.08648	0.05182	0.22206	0.11286	0
0	0.00046	0	0	0	0.09494	0.11286	0
0	0	0.00521	0	0	0.01401	0	0
0	1.44011	0.60308	0.01646	0	0	0	0
0	0	0.1405	0.07002	0.03345	0	0	0
0	0	0	0	0	0	0	0
0	0	0	0	0	0	0	0
0	0	0	0	0	0	0	0
0	0	0	0	0	0	0	0
0	0	0.06972	0	0	0	0	0
0	0	0.00354	0	0	0	0	0
0	0	0	0	0	0	0	0
0	0	0.00047	0	0.01838	0.11311	0	0
0	0	0	0	0	0	0	0
0	0	0	0	0	0	0	0
4.14342	1.38451	12.18247	12.42596	9.87762	8.09097	18.13306	8.63438
0	0.00246	0.33919	0.00634	0.06258	1.02945	0	0.00028
0	0.00246	0	0	0	0	0	0.00028
0	0	0.33919	0.00634	0.06258	1.02945	0	0
0.11019	0	0.02874	0.08995	0.01	0.24995	2.4984	0
0.11019	0	0.02874	0.08995	0.01	0.24995	2.4984	0
2.95361	0.3488	0.79161	1.19133	1.94474	1.08436	0.02688	7.33579

0	0	0	0	0.00227	0.00009	0	0
0	0.00078	0.10413	0.0414	0.00614	0.02378	0	0.00475
0	0	0	0.00262	0	0	0	0
0	0	0	0	0	0	0	0
0	0.00859	0.00028	0	0.00074	0	0	0
0.00188	0.02012	0.00364	0	0.00529	0.07423	0	0.24726
0	0.00579	0.19925	0.01801	0.63892	0.23901	0	0.51104
2.95172	0.2496	0.48432	1.1293	1.29138	0.58608	0.02688	4.24119
0	0.06392	0	0	0	0.16116	0	2.33155
0	0	0	0	0	0	0	0
0	0	0	0	0	0	0	0
0	0	0	0	0	0	0	0
0	0	0	0	0	0	0	0
0	0	0	0	0	0	0	0
0.14036	0.23799	0.13995	0.00025	0	0	0	0
0	0	0	0	0	0	0	0
0.14036	0.23052	0.13995	0.00025	0	0	0	0
0	0.00747	0	0	0	0	0	0
0.00077	0	0.04516	5.83676	0.00611	0	0	0
0	0	0	0	0	0	0	0
0.00077	0	0.04516	5.83676	0.00611	0	0	0
0.0003	0	0	0	0	0	0	0
0.0003	0	0	0	0	0	0	0
0	0	0.41054	0.02744	0	0.69188	0	0.8098
0	0	0.41054	0.02744	0	0.69188	0	0.8098
0	0.00116	0	0	0	0	0	0
0	0.00116	0	0	0	0	0	0
0.93821	0.34916	0.4385	4.85944	0.25109	1.60541	14.31433	0.3976
0.14825	0.02449	0.00412	0.01031	0.0007	0.00674	0.0143	0
0	0.00308	0	0	0	0.12346	0	0.00584
0.16158	0.14049	0.2296	2.98663	0.2266	0.60612	1.98252	0.36334
0.02439	0.03003	0.05131	0.17705	0.00405	0.24491	0.22727	0.02312
0	0.06363	0	0.41385	0.00181	0	0	0
0	0	0	0	0	0	0	0
0.00622	0.04911	0.05971	0.03204	0.00145	0.01031	0	0.0001

0	0	0	0	0	0	0	0
0	0	0.00048	0.00028	0	0.01565	0	0
0.59777	0.03832	0.09329	1.23928	0.01648	0.59822	12.09024	0.0052
0	0	0	0	0	0	0	0
0	0	0	0	0	0	0	0
0	0	3.15501	0.05855	0	0	0	0
0	0.30558	0.1245	0.1229	0.01315	0	0	0
0	0.30486	0	0	0	0	0	0
0	0	0.1245	0.1229	0.01315	0	0	0
0	0.00072	0	0	0	0	0	0
0	0	3.15501	0.05855	0	0	0	0
0	0	0	0	0	0	0	0
0	0	0	0	0	0	0	0
0	0	0	0	0.00207	0	0	0
0	0	0	0	0.00207	0	0	0
0	0.01528	0	0	0.00325	0	0	0
0	0.00959	0	0	0.00314	0	0	0
0	0.00569	0	0	0.0001	0	0	0
0	0	0	0	0	0	0	0
0	0	0	0	0	0	0	0
0	0.12224	6.67033	0.19374	7.58149	2.05196	0	0
0	0.11981	0.47342	0.01299	0.08287	0	0	0
0	0.00244	0.01126	0	0	0	0	0
0	0	0.44141	0.00191	0	0.89762	0	0
0	0	5.74424	0.17884	7.49862	1.15434	0	0
0	0	0	0	0	0	0	0
0	0	0	0	0	0	0	0
0	0	0.00378	0.01879	0.00016	0.66562	1.09975	0.05298
0	0	0.00378	0.01879	0.00016	0.66562	1.09975	0.05298
0	0	0	0	0	0	0	0
0	0	0	0	0	0	0	0
0	0.00184	0	0	0.00241	0	0	0
0	0.00184	0	0	0.00241	0	0	0
0	0	0.03516	0.02047	0.00057	0.71235	0.19369	0.03794
0	0	0.03516	0.02047	0.00057	0.71235	0.19369	0.03794

0	0	0	0	0	0	0	0
0	0	0	0	0	0	0	0
0	0	0	0	0	0	0	0
0	0	0	0	0	0	0	0
0.00718	0.35925	0.02612	0.02312	0.00164	0.6285	0	0.14294
0.00133	0	0	0	0	0	0	0
0.00133	0	0	0	0	0	0	0
0	0.00088	0	0	0	0	0	0
0	0.00088	0	0	0	0	0	0
0	0.01268	0.02588	0.02013	0	0.6285	0	0.14294
0	0.01268	0.02588	0.02013	0	0.6285	0	0.14294
0	0.22377	0	0	0	0	0	0
0	0.22377	0	0	0	0	0	0
0	0.11512	0	0	0	0	0	0
0	0.11512	0	0	0	0	0	0
0	0	0	0.00298	0	0	0	0
0	0	0	0	0	0	0	0
0	0	0	0.00298	0	0	0	0
0.00366	0.00232	0.00024	0	0.00164	0	0	0
0.00366	0.00232	0.00024	0	0.00164	0	0	0
0.00219	0.00448	0	0	0	0	0	0
0.00219	0.00448	0	0	0	0	0	0
3.16821	0.12298	3.94581	5.57667	0.40712	5.41164	4.63385	3.97833
0	0	0.09763	0.09096	0.16648	0.22391	0.03648	0
0	0	0.09763	0.09096	0.16648	0.22391	0.03648	0
0	0	0	0	0	0	0	0
0	0	0	0	0	0	0	0
0	0	0	0	0	0	0	0
0	0	0	0	0	0	0	0
0.43127	0.01417	0.04506	0.09202	0	0.20343	0.04197	0
0.43127	0.01417	0.04506	0.09202	0	0.20343	0.04197	0
0	0	1.30967	3.5637	0.14707	0.54951	2.78882	3.23315
0	0	1.30967	3.5637	0.14707	0.54951	2.78882	3.23315
1.64331	0.03395	2.39449	1.26947	0.0894	3.72462	0.07719	0.73077
1.64331	0.03395	2.39449	1.26947	0.0894	3.72462	0.07719	0.73077

0	0	0	0	0	0	0	0
0	0	0.01153	0.00914	0	0	0	0
0	0	0.01153	0.00914	0	0	0	0
0	0	0	0	0	0	0	0
0	0	0	0	0	0	0	0
0	0	0	0	0	0	0	0
0	0	0	0	0	0	0	0
0	0	0	0	0	0	0	0
0	0	0	0	0	0	0	0
0	0	0	0	0	0	0	0
0	0	0	0	0	0	0	0
0	0	0	0	0	0	0	0
0	0	0	0	0	0	0	0
0	0	0	0	0	0	0	0
0	0.05986	0	0	0	0	0	0
0	0	0	0	0	0	0	0
0	0.05986	0	0	0	0	0	0
0	0	0	0	0	0	0	0
0	0	0	0	0	0	0	0
0	0	0	0	0	0	0	0
1.09363	0.015	0.08743	0.55138	0.00417	0.71017	1.6894	0.01441
1.09363	0.015	0.08743	0.55138	0.00417	0.71017	1.6894	0.01441
0.31131	0.23491	0.18389	3.39629	0.24161	1.0497	4.37773	2.6154
0.31131	0.23491	0.18389	3.39629	0.24161	1.0497	4.37773	2.6154
0.31131	0.23491	0.18389	3.39629	0.24161	1.0497	4.37773	2.6154
0	0	0.00508	0	0	0.02075	0	0
0	0	0.00508	0	0	0.02075	0	0
0	0	0	0	0	0	0	0
0	0	0	0	0	0	0	0
0	0	0.00178	0	0.16341	0	0	0
0	0	0.00178	0	0.16341	0	0	0
0	0	0.00828	2.18713	0	0	0	0
0	0	0.00828	2.18713	0	0	0	0
0.00942	0.00105	0	0	0	0.17836	0.02923	0
0.00942	0.00105	0	0	0	0.17836	0.02923	0

0	0	0	0	0	0	0	0
0	0	0	0	0	0	0	0
0.29946	0.22858	0.14482	1.19216	0.07466	0.85059	4.3485	2.61437
0.07763	0.01172	0.14482	1.18873	0.03122	0.39167	2.85941	1.05277
0.01384	0	0	0	0	0	0	0
0.20799	0.21686	0	0.00343	0.04344	0.45893	1.48909	1.56161
0	0	0	0	0	0	0	0
0	0	0	0	0	0	0	0
0	0	0	0	0	0	0	0
0	0	0	0	0	0	0	0
0	0	0	0	0	0	0	0
0	0	0	0	0	0	0	0
0	0	0	0	0	0	0	0
0	0	0	0	0	0	0	0
0	0	0.02392	0.01699	0	0	0	0
0	0	0.02392	0.01699	0	0	0	0
0	0	0	0	0	0	0	0
0	0	0	0	0	0	0	0
0.00243	0.00528	0	0	0	0	0	0
0.00243	0.00528	0	0	0	0	0	0
0	0	0	0	0.00354	0	0	0.00103
0	0	0	0	0.00354	0	0	0.00103
0	0	0.12456	0.44546	0	0	0	0
0	0	0.12456	0.44546	0	0	0	0
0	0	0.12456	0.44546	0	0	0	0
0	0	0.12456	0.44546	0	0	0	0
0	0	0	0	0	0	0	0
0	0	0	0	0	0	0	0
0	0	0	0	0	0	0	0
0	0	0	0	0	0	0	0
0	0	0	0	0	0	0	0
0	0	0.12456	0.44546	0	0	0	0
0	0	0	0	0	0	0	0
0	0	0	0	0	0	0	0
6.67186	0.88207	0.92873	0.46659	2.79394	1.69633	0.07208	1.72783

1.65667	0.7293	0.3782	0.21779	2.4104	1.67035	0.05507	1.6753
1.65667	0.7293	0.3782	0.21779	2.4104	1.67035	0.05507	1.6753
0	0.00031	0	0	0	0	0	0
0	0	0	0	0	0	0	0
0	0.00031	0	0	0	0	0	0
1.65667	0.72899	0.3782	0.21779	2.4104	1.67035	0.05507	1.6753
1.65667	0.72899	0	0.00565	0.00462	1.67035	0.05507	1.6753
0	0	0.3782	0.21214	2.40578	0	0	0
0.00818	0.00617	0.24011	0.00485	0	0	0	0
0.00818	0.00617	0.24011	0.00485	0	0	0	0
0	0	0	0	0	0	0	0
0	0	0	0	0	0	0	0
0	0	0	0	0	0	0	0
0	0	0.24011	0.00485	0	0	0	0
0	0	0.24011	0.00485	0	0	0	0
0.00818	0.00617	0	0	0	0	0	0
0.00785	0	0	0	0	0	0	0
0.00012	0.00617	0	0	0	0	0	0
0.00021	0	0	0	0	0	0	0
0	0	0	0	0	0	0	0
5.00701	0.1466	0.31043	0.24395	0.38354	0.02599	0.017	0.05253
5.00701	0.1466	0.31043	0.24395	0.38354	0.02599	0.017	0.05253
0	0	0	0	0	0	0	0
0	0	0	0	0	0	0	0
0.01155	0	0	0	0	0	0	0
0.01155	0	0	0	0	0	0	0
0.01103	0	0	0.00477	0	0.01025	0.017	0.00324
0.00094	0	0	0	0	0	0	0
0.01009	0	0	0.00477	0	0.01025	0.017	0.00324
0	0	0	0	0	0	0	0
0.0158	0.01544	0.31043	0.23663	0.07005	0.00272	0	0
0	0	0.31043	0.23243	0.05267	0	0	0
0	0	0	0	0	0	0	0
0.0158	0.01544	0	0.0042	0.01739	0.00272	0	0
0	0	0	0	0	0	0	0

0	0	0	0	0	0	0	0
4.96863	0.13116	0	0.00254	0.31348	0.01301	0	0.0493
0.79773	0.02099	0	0	0.11734	0.01177	0	0.02493
0.12404	0.00267	0	0	0.03428	0	0	0.00539
0.23901	0.01487	0	0	0.00747	0	0	0
3.15932	0.07753	0	0.00254	0.1231	0.00124	0	0.01462
0.03032	0	0	0	0	0	0	0
0.00034	0	0	0	0	0	0	0
0.17404	0.0046	0	0	0.03129	0	0	0.00435
0.44382	0.0105	0	0	0	0	0	0
0.00431	0.00327	0	0.00655	0.00011	0.00083	0.00289	0
0.00431	0.00327	0	0.00655	0.00011	0.00083	0.00289	0
0.00431	0.00327	0	0.00655	0.00011	0.00083	0.00289	0
0	0	0	0.00405	0	0	0	0
0	0	0	0	0	0	0	0
0	0	0	0.00405	0	0	0	0
0	0	0	0.0012	0	0.00066	0.00289	0
0	0	0	0.0012	0	0.00066	0.00289	0
0.00431	0.00327	0	0.0013	0.00011	0	0	0
0.00431	0.00327	0	0.0013	0.00011	0	0	0
0	0	0	0	0	0	0	0
0	0	0	0	0	0.00017	0	0
0	0	0	0	0	0	0	0
0	0	0	0	0	0.00017	0	0
0	0	0	0	0	0	0	0
0.57623	0.04111	3.78843	1.3307	10.53062	1.33907	8.63312	2.11027
0.57623	0.04111	3.78843	1.3307	10.53062	1.33907	8.63312	2.11027
0.57623	0.04111	3.78843	1.3307	10.53062	1.33907	8.63312	2.11027
0.57502	0.03789	3.78843	1.3307	10.53062	1.33907	8.63312	2.11027
0	0.00053	0	0	0	0	0	0
0	0.00053	0	0	0	0	0	0
0.57502	0.03736	3.78843	1.3307	10.53062	1.33907	8.63312	2.11027
0.00123	0	0	0	0	0	0	0
0	0	3.78098	1.31536	10.5294	0	0	0
0	0	0	0	0	0	0	0

0.52923	0.00942	0	0.00119	0	0	0	0
0.04457	0.02794	0	0	0	0	0	0
0	0	0	0	0	0	0	0
0	0	0.00073	0	0	0	0	0
0	0	0.00673	0.01415	0.00122	1.33907	8.63312	2.11027
0.0012	0.00323	0	0	0	0	0	0
0.0012	0.00323	0	0	0	0	0	0
0	0.00064	0	0	0	0	0	0
0.00071	0.00025	0	0	0	0	0	0
0	0	0	0	0	0	0	0
0.00042	0	0	0	0	0	0	0
0.00008	0.00234	0	0	0	0	0	0
0	0	0	0	0	0	0	0
0	0	0	0	0	0	0	0
0	0	0	0	0	0	0	0
0	0	0	0	0	0	0	0
0	0	0	0	0	0	0	0
0	0	0	0	0	0	0	0
34.38228	38.22236	1.11228	3.1642	0.90004	4.17471	26.79641	4.57473
0.48212	0.15945	0	0	0	0.35516	0	0
0.1967	0.14899	0	0	0	0.35516	0	0
0.17459	0.12526	0	0	0	0	0	0
0.17459	0.12526	0	0	0	0	0	0
0.17459	0.12526	0	0	0	0	0	0
0	0	0	0	0	0	0	0
0	0	0	0	0	0	0	0
0	0	0	0	0	0	0	0
0	0	0	0	0	0.35516	0	0
0	0	0	0	0	0.35516	0	0
0	0	0	0	0	0.35516	0	0
0.02211	0.02373	0	0	0	0	0	0
0	0	0	0	0	0	0	0
0	0	0	0	0	0	0	0
0.02211	0.02373	0	0	0	0	0	0
0.02211	0.02373	0	0	0	0	0	0

0	0	0	0	0	0	0	0
0	0	0	0	0	0	0	0
0.28542	0.01046	0	0	0	0	0	0
0.28542	0.01046	0	0	0	0	0	0
0.22142	0	0	0	0	0	0	0
0.22142	0	0	0	0	0	0	0
0.06399	0.01046	0	0	0	0	0	0
0	0	0	0	0	0	0	0
0.0293	0.00849	0	0	0	0	0	0
0.02049	0	0	0	0	0	0	0
0.0142	0.00197	0	0	0	0	0	0
0	0	0	0	0	0	0	0
0.11529	0.03175	0.04556	0.02075	0	0.17638	0.05664	0.00281
0.11529	0.03175	0.04556	0.02075	0	0.17638	0.05664	0.00281
0.11529	0.03175	0.04556	0.02075	0	0.17638	0.05664	0.00281
0.11529	0.03175	0.04556	0.02075	0	0.17638	0.05664	0.00281
0.11529	0.03175	0.04556	0.02075	0	0.17638	0.05664	0.00281
0	0	0	0	0	0	0	0
0	0	0	0	0	0	0	0
0	0	0	0	0	0	0	0
0.07278	0.01787	0.0173	0.0117	0.00285	0.16575	0	0
0.07278	0.01787	0.0173	0.0117	0.00285	0.16575	0	0
0.07278	0.01787	0.0173	0.0117	0.00285	0.16575	0	0
0.07278	0.01787	0.0173	0.0117	0.00285	0.16575	0	0
0.05399	0.01709	0	0	0.00285	0	0	0
0.00058	0	0	0	0	0	0	0
0	0	0	0	0	0.14431	0	0
0	0	0	0	0	0	0	0
0.01821	0.00078	0	0	0	0	0	0
0	0	0.0173	0.0117	0	0.02144	0	0
0	0	0	0	0	0	0	0
0	0	0	0	0	0	0	0
0	0	0	0	0	0	0	0
33.71209	38.0133	1.04942	3.13176	0.89719	3.47741	26.73977	4.57192
0	0.00542	0	0	0	0	0	0

0	0.00542	0	0	0	0	0	0
0	0.00542	0	0	0	0	0	0
0	0	0	0	0	0	0	0
0	0	0	0	0	0	0	0
0	0.00542	0	0	0	0	0	0
0	0	0	0	0	0	0	0
0	0	0	0	0	0	0	0
0	0	0	0	0	0	0	0
0.03133	0.00232	0	0	0	0	0	0
0.03133	0.00232	0	0	0	0	0	0
0.03133	0.00232	0	0	0	0	0	0
0.00627	0.00115	0	0	0	0	0	0
0.02506	0.00117	0	0	0	0	0	0
27.45095	36.10118	1.04942	3.13176	0.0277	3.47547	26.72251	4.49827
0	0.00664	0	0	0	0	0	0
0	0.00664	0	0	0	0	0	0
0	0.00664	0	0	0	0	0	0
26.94329	36.09454	1.04942	3.12578	0.0277	3.47547	26.72251	4.49827
0	0.02354	0	0	0.00542	0	0	0
0	0	0	0	0	0	0	0
0	0.00221	0	0	0	0	0	0
0	0	0	0	0	0	0	0
0	0	0	0	0	0	0	0
0	0.01238	0	0	0.00393	0	0	0
0	0	0	0	0	0	0	0
0	0	0	0	0	0	0	0
0	0.00223	0	0	0	0	0	0
0	0	0	0	0	0	0	0
0	0	0	0	0	0	0	0
0	0.00672	0	0	0.00148	0	0	0
0	0	0	0	0	0	0	0
0	0	0	0	0	0	0	0
0.00103	4.84522	0	0	0	0	0	0
0	0	0	0	0	0	0	0
0.00103	4.84522	0	0	0	0	0	0

0	0	0	0	0	0	0	0
8.23614	29.8524	1.04392	3.11916	0.02104	3.47547	26.72251	4.49827
8.23614	29.8524	1.04392	3.11916	0.02104	3.47547	26.72251	4.49827
0	0	0	0	0	0	0	0
18.70612	1.37339	0.0055	0.00663	0.00124	0	0	0
0	0.01334	0	0	0	0	0	0
0	0	0	0	0	0	0	0
0	0	0	0	0	0	0	0
16.06869	0.58481	0.0055	0.00663	0	0	0	0
1.70071	0.58217	0	0	0	0	0	0
0.93672	0.19306	0	0	0.00124	0	0	0
0	0	0	0	0	0	0	0
0	0	0	0	0	0	0	0
0	0	0	0	0	0	0	0
0	0	0	0	0	0	0	0
0	0	0	0	0	0	0	0
0	0	0	0	0	0	0	0
0	0	0	0	0	0	0	0
0	0	0	0	0	0	0	0
0	0	0	0.00598	0	0	0	0
0	0	0	0.00598	0	0	0	0
0	0	0	0.00598	0	0	0	0
0.50766	0	0	0	0	0	0	0
0.50766	0	0	0	0	0	0	0
0.50766	0	0	0	0	0	0	0
0	0	0	0	0	0	0	0
0	0	0	0	0	0	0	0
6.22981	1.90438	0	0	0.86949	0.00194	0.00927	0.07365
6.22981	1.90438	0	0	0.86949	0.00194	0.00927	0.07365
0.62459	0.19525	0	0	0	0	0	0.001
0.41337	0.16328	0	0	0	0	0	0.001
0.21122	0.03197	0	0	0	0	0	0
5.60522	1.70913	0	0	0.86949	0.00194	0.00927	0.07265
0	0.00685	0	0	0	0	0	0
0	0.00233	0	0	0	0	0	0

5.60522	1.6984	0	0	0.84565	0.00172	0.00927	0.06075
0	0	0	0	0	0	0	0
0	0.00155	0	0	0	0	0	0
0	0	0	0	0.02384	0.00022	0	0.01189
0	0	0	0	0	0	0	0
0	0	0	0	0	0	0.008	0
0	0	0	0	0	0	0.008	0
0	0	0	0	0	0	0.008	0
0	0	0	0	0	0	0.008	0
0	0	0	0	0	0	0	0
0	0	0	0	0	0	0	0
0	0	0	0	0	0	0	0
0	0	0	0	0	0	0	0
0	0	0	0	0	0	0	0
0	0	0	0	0	0	0	0
0	0	0	0	0	0	0	0
0	0	0	0	0	0	0	0
0	0.00127	0	0	0	0	0	0
0	0.00127	0	0	0	0	0	0
0	0.00127	0	0	0	0	0	0
0	0.00127	0	0	0	0	0	0
0	0	0	0	0	0	0	0
0	0	0	0	0	0	0	0
0	0	0	0	0	0	0	0
0	0	0	0	0	0	0	0
0	0	0	0	0	0	0	0
0	0.00127	0	0	0	0	0	0
0	0.00127	0	0	0	0	0	0
0	0	0	0	0	0	0	0
0	0	0	0	0	0	0	0
0	0	0	0	0	0	0	0
0	0	0	0	0	0	0	0
0	0	0	0	0	0	0	0
0	0	0	0	0	0	0	0
0	0	0	0	0	0	0	0

0	0	0	0	0	0	0	0.00033
0	0	0	0	0	0	0	0.00033
0	0	0	0	0	0	0	0.00033
0	0	0	0	0	0	0	0.00033
0	0	0	0	0	0	0	0.00033
0	0	0	0	0	0	0	0.00033
0	0	0	0	0	0	0	0
0	0	0	0	0	0	0	0
0	0	0	0	0	0	0	0
0	0	0	0	0	0	0	0
0	0	0	0	0	0	0	0
0	0	0	0	0	0	0	0
0	0	0	0	0	0	0	0
0	0	0	0	0	0	0	0
0	0	0	0	0	0	0	0
0	0	0	0	0	0	0	0
0.0031	0	0	0	0	0	0	0
0.0031	0	0	0	0	0	0	0
0.0031	0	0	0	0	0	0	0
0.0031	0	0	0	0	0	0	0
0	0	0	0	0	0	0	0
0	0	0	0	0	0	0	0
0	0	0	0	0	0	0	0
0.0031	0	0	0	0	0	0	0
0.0031	0	0	0	0	0	0	0
0.0031	0	0	0	0	0	0	0

AP011_1	AP011_2	AP011_3	AP013_1	AP013_2	AP013_3	AP014_1	AP014_2
100	99.93978	100	100	100	100	99.99866	99.99722
1.93693	6.04031	2.7563	0.33956	0.6431	0.72081	0.14769	0.36028
1.81974	4.83339	2.49033	0.08468	0.01441	0.57712	0.01123	0.04271
0.00108	0.03015	0.00329	0.00268	0.00083	0	0	0.00579
0.00108	0.03015	0.00329	0.00268	0.00083	0	0	0.00579
0	0	0	0	0	0	0	0.00308
0	0	0	0	0	0	0	0.00308
0.00108	0.03015	0.00329	0.00268	0.00083	0	0	0.00271
0	0	0	0	0	0	0	0
0	0	0	0	0	0	0	0
0	0	0	0	0	0	0	0
0	0	0	0	0	0	0	0
0	0	0	0	0	0	0	0
0	0	0	0	0	0	0	0
0	0	0	0.0021	0.00007	0	0	0
0	0	0	0	0	0	0	0
0	0	0	0	0	0	0	0
0	0.00132	0	0	0	0	0	0
0	0	0	0.00058	0	0	0	0
0	0.00221	0	0	0.00076	0	0	0
0	0.00086	0	0	0	0	0	0
0.00108	0.02576	0.00329	0	0	0	0	0
0	0	0	0	0	0	0	0
0	0	0	0	0	0	0	0.00271
0	0	0	0	0	0	0	0
0	0	0	0	0	0	0	0
1.81866	4.79893	2.48704	0.0801	0.01248	0.57712	0	0.00087
1.81866	4.79893	2.48704	0.0801	0.01248	0.57712	0	0.00087
0	0	0	0	0	0	0	0
0	0	0	0	0	0	0	0
0	0	0	0	0	0	0	0
0	0	0	0	0	0	0	0
1.81531	4.77306	2.48704	0.0801	0.01248	0.57712	0	0.00087

0	0	0	0	0	0.00104	0	0
0	0	0	0	0	0	0	0
0	0	0	0	0	0	0	0
0	0	0	0	0	0	0	0
0	0.00101	0	0	0	0	0	0
0.63303	0.7126	2.36411	0.00543	0.00332	0.00292	0	0.00087
1.18228	4.05945	0.12294	0.07468	0.00916	0.57316	0	0
0	0	0	0	0	0	0	0
0	0	0	0	0	0	0	0
0.00334	0.02587	0	0	0	0	0	0
0.00334	0.02587	0	0	0	0	0	0
0	0	0	0	0	0	0	0
0	0	0	0	0	0	0	0
0	0	0	0	0	0	0	0
0	0	0	0	0	0	0	0
0	0	0	0	0	0	0	0
0	0	0	0	0	0	0	0
0	0.00431	0	0.00189	0.0011	0	0.01123	0.03604
0	0.00431	0	0.00189	0.0011	0	0.01123	0.03604
0	0.00431	0	0.00189	0.0011	0	0.01123	0.03604
0	0	0	0	0	0	0	0.00102
0	0.00431	0	0	0	0	0.00015	0
0	0	0	0.00189	0.0011	0	0.01108	0.03502
0	0	0	0	0	0	0	0
0	0	0	0	0	0	0	0
0	0	0	0	0	0	0	0
0	0	0	0	0	0	0	0
0	0	0	0	0	0	0	0
0	0	0	0	0	0	0	0
0.11719	1.20692	0.26597	0.25488	0.62868	0.14369	0.13647	0.31757
0.10331	0.87605	0.22969	0.18052	0.18353	0.13321	0.09718	0.16047
0	0.00796	0	0	0	0	0	0
0	0.00796	0	0	0	0	0	0
0	0	0	0	0	0	0	0
0	0.00796	0	0	0	0	0	0

0	0	0	0	0	0	0	0
0	0	0	0	0	0	0	0
0	0	0	0	0	0	0	0
0	0	0	0	0	0	0	0
0.10331	0.86809	0.22969	0.18052	0.18353	0.13321	0.09718	0.16047
0.10331	0.86798	0.22969	0.18052	0.18353	0.13321	0.09718	0.16047
0.0894	0.84689	0.22655	0.18052	0.18353	0.13321	0.09718	0.16047
0.00336	0.00471	0.0006	0	0	0	0	0
0.01054	0.01639	0.00254	0	0	0	0	0
0	0.00011	0	0	0	0	0	0
0	0.00011	0	0	0	0	0	0
0	0	0	0	0	0	0	0
0.01388	0.33087	0.03628	0.07436	0.44515	0.01048	0.03929	0.15711
0.01388	0.33087	0.03628	0.07436	0.44515	0.01048	0.03929	0.15711
0.00334	0.01812	0	0.02298	0.01045	0.00458	0.00014	0
0.00334	0.01812	0	0.02298	0.01045	0.00458	0.00014	0
0.00056	0.00366	0	0.00644	0.00326	0.00031	0	0
0.00056	0.00366	0	0.00644	0.00326	0.00031	0	0
0.00865	0.20477	0.02727	0.03815	0.38018	0.00517	0.0379	0.14827
0.00865	0.20477	0.02727	0.03815	0.38018	0.00517	0.0379	0.14827
0	0.00032	0	0	0.00007	0	0	0
0	0.00032	0	0	0.00007	0	0	0
0.00134	0.10399	0.00901	0.0068	0.0512	0.00042	0.00125	0.00883
0.00134	0.10399	0.00901	0.0068	0.0512	0.00042	0.00125	0.00883
0	0	0	0	0	0	0	0
0	0	0	0	0	0	0	0
70.10969	74.33106	68.66265	75.30629	72.66905	77.93315	83.57551	70.76873
0	0	0	0	0	0	0	0
0	0	0	0	0	0	0	0
0	0	0	0	0	0	0	0
0	0	0	0	0	0	0	0
0	0	0	0	0	0	0	0
70.10969	74.33106	68.66265	75.30629	72.66905	77.93315	83.57551	70.76873
70.10969	74.33106	68.66265	75.30629	72.66905	77.93315	83.57551	70.76873
46.56422	30.56495	57.23497	51.97555	49.86091	66.37601	66.09381	41.06396

46.56422	30.56495	57.23497	51.97555	49.86091	66.37601	66.09381	41.06396
0	0	0	0	0	0.00064	7.32368	1.76049
0	0	0	0.10425	0.2622	1.18225	0.04305	0.00045
0	0	0	0	0	0	0	0
0.00101	0.00151	0.00098	0.00084	0	0.00535	12.30689	11.41954
0.02704	0.17364	4.27978	0	0	0	0	0
0	0	0	0.00009	0	0	0	0
0	0	0	0	0	0	0	0
0.53053	0.12517	0.17943	0	0	0	0	0
4.75979	0.1001	1.62077	8.14948	7.75841	4.06687	12.15317	13.90131
2.61663	0.70626	0.78464	0.75672	0.31208	0.28452	0.54255	0.08193
2.28401	6.00039	2.12729	0	0	0	0	0
0	0	0.06758	0.00942	0.00769	0.00318	0.18654	0.03294
3.4162	1.91749	1.58197	0.5037	0.06932	2.67832	0.23736	0.4742
0.00028	0.00052	0.00039	0.00032	0	0.00079	0.16247	0.00327
0.05832	0.04904	0.09486	0.65779	2.95095	0.0714	0	0
0	0	0	0.00212	0.00011	0.00883	0	0
0	0	0	0	0	0	0.2032	0.32074
2.98341	0.76132	16.44498	0	0	0	0.75233	0.35406
3.62896	2.13959	6.09481	4.98158	12.75278	3.17553	26.66398	10.17909
21.17367	13.25665	2.57249	12.42017	3.95582	33.80301	5.42457	2.52162
5.08116	5.33241	21.38298	24.28535	21.79008	19.27064	0.01021	0.00867
0.0032	0.00085	0.002	0.10372	0.00145	1.82469	0.0838	0.00564
1.54212	0.09528	0.13017	0	0	0	0.01653	0.00877
0	0	0	0	0	0	0	0
0	0	0	0	0	0	0	0
1.54212	0.09528	0.13017	0	0	0	0.01653	0.00877
0	0	0	0	0	0	0	0
1.54212	0.09528	0.13017	0	0	0	0.01653	0.00877
0.28684	1.039	1.24038	3.71204	3.47717	1.03393	4.04428	10.2402
0.09136	0.91603	0.53705	3.1816	2.65422	0.56137	1.78068	2.45071
0	0	0	0.00542	0.00197	0.00114	0.00169	0.00055
0.09136	0.91603	0.53705	3.17618	2.65225	0.56022	1.77899	2.45016
0.19548	0.12297	0.70332	0.53045	0.82295	0.47257	2.2636	7.78949
0	0	0	0	0	0	0	0

0.19548	0.12297	0.70332	0.53045	0.82295	0.47257	2.2636	7.78949
0	0	0	0.00054	0.00242	0	0	0
0	0	0	0	0	0	0	0
0	0	0	0	0	0	0	0
0	0	0	0	0	0	0	0
0	0	0	0	0	0	0	0
0	0	0	0.00054	0.00242	0	0	0
0	0	0	0.00054	0.00242	0	0	0
0.00057	0	0.00307	0	0.0011	0	0.0071	0.00397
0	0	0	0	0	0	0	0
0	0	0	0	0	0	0	0
0	0	0	0	0	0	0	0
0	0	0	0	0	0	0	0
0	0	0	0	0	0	0	0
0.00057	0	0.00307	0	0.0011	0	0.0071	0.00397
0	0	0	0	0	0	0.0071	0.00397
0	0	0	0	0	0	0	0
0	0	0	0	0.0011	0	0	0
0	0	0	0	0	0	0	0
0.00057	0	0	0	0	0	0	0
0	0	0	0	0	0	0	0
0	0	0.00307	0	0	0	0	0
0	0	0	0	0	0	0	0
0	0	0	0	0	0	0	0
0	0	0	0	0	0	0	0
0	0	0	0	0	0	0	0
0	0	0	0	0	0	0	0
0	0	0	0	0	0	0	0
0	0	0	0	0	0	0	0
0	0	0	0	0	0	0	0
0	0	0	0	0	0	0	0
0	0	0	0	0	0	0	0
0	0	0	0	0	0	0	0
15.12638	16.45769	4.88285	14.09548	8.72586	6.26008	2.92461	9.05106
15.12638	16.45769	4.88285	14.09548	8.72586	6.26008	2.92461	9.05106

7.45391	6.60709	1.0995	0.78588	0.51803	0.13549	0.05947	0.51339
0	0.00045	0.00403	0.06507	0.60531	0.03857	0.01112	0.05304
0	0	0	1.83634	1.71996	0.25879	0	0
7.67248	9.85016	3.77932	9.3621	5.67109	5.52106	1.68913	7.26466
0	0	0	2.04609	0.21148	0.30617	1.16489	1.21997
0	0	0	0	0	0	0	0
6.58955	26.17414	5.17122	5.52268	10.60159	4.26312	10.48917	10.40077
6.58955	26.17414	5.17122	5.52268	10.60159	4.26312	10.48917	10.40077
0.21587	0.40034	3.52469	4.53564	6.85951	4.17227	5.27453	5.5421
0.06868	0.01242	0.18006	0.87784	1.558	0.07231	0.59925	2.07032
0	0	0	0.10603	2.18174	0.01083	4.57936	2.12638
0	0	0	0.00144	0.00123	0.00772	0	0
6.305	25.76137	1.46646	0.00173	0.00112	0	0.02443	0.64236
0	0	0	0	0	0	0.01161	0.0196
0	0	0	0	0	0	0	0
0	0	0	0	0	0	0	0
0	0	0	0	0	0	0	0
0	0	0	0	0	0	0	0
0	0	0	0	0	0	0	0
0	0	0	0	0	0	0	0
21.42369	13.85965	11.1324	12.68018	10.15954	11.06725	12.13667	25.73933
0.03562	0.32836	0.03819	0.18334	0.06009	0.09186	0.39664	0.13222
0	0	0	0.00178	0	0	0	0
0	0	0	0.00178	0	0	0	0
0	0	0	0.00178	0	0	0	0
0	0	0	0	0	0	0	0
0	0	0	0	0	0	0	0
0	0	0	0	0	0	0	0
0	0	0	0.00178	0	0	0	0
0.03562	0.32836	0.03819	0.18156	0.06009	0.09186	0.39664	0.13222
0	0	0	0	0	0	0	0
0	0	0	0	0	0	0	0
0	0	0	0	0	0	0	0
0	0	0	0	0	0	0	0
0	0	0	0	0	0	0	0.00253

0	0	0	0	0	0	0	0.00253
0	0	0	0	0	0	0	0.00253
0	0	0	0	0	0	0	0
0	0.17967	0.01563	0.02727	0.05041	0.02598	0.31572	0.11495
0	0.17967	0.01563	0.02727	0.05041	0.02598	0.31572	0.11495
0	0	0	0	0	0	0	0
0	0	0	0	0	0	0	0
0	0	0	0	0	0	0	0
0	0.17967	0.01563	0.00496	0.03381	0.00731	0.31572	0.11495
0	0	0	0.02231	0.01661	0.01866	0	0
0	0	0	0	0	0	0	0
0	0	0	0	0	0	0	0
0	0	0	0	0	0	0	0
0	0	0	0	0	0	0	0
0	0	0	0	0	0	0	0
0.00698	0.07239	0	0	0	0	0.00126	0
0.00698	0.07239	0	0	0	0	0.00126	0
0	0	0	0	0	0	0	0
0	0	0	0	0	0	0	0
0	0	0	0	0	0	0	0
0	0	0	0	0	0	0	0
0	0	0	0	0	0	0	0
0	0	0	0	0	0	0	0
0	0	0	0	0	0	0	0
0.00327	0	0	0	0	0	0	0
0.0037	0.07239	0	0	0	0	0.00126	0
0	0	0	0	0	0	0	0
0	0	0	0	0	0	0	0
0	0	0	0	0	0	0	0
0	0	0	0	0	0	0	0
0	0	0	0	0	0	0	0
0	0	0	0	0	0	0	0
0	0	0	0	0	0	0	0
0	0	0	0	0	0	0	0
0.02864	0.0763	0.02256	0.15428	0.00968	0.06588	0.07966	0.01474

0.02864	0.0763	0.02256	0.15428	0.00968	0.06588	0.07966	0.01474
0	0.00113	0	0.0028	0.00088	0	0	0
0	0	0	0	0	0	0	0
0	0	0	0	0	0	0	0
0	0.0001	0	0	0	0	0	0
0	0	0	0	0	0	0.01054	0
0	0	0	0.00034	0.00347	0	0	0.01474
0	0	0	0	0	0	0	0
0	0	0	0	0	0	0	0
0	0	0	0	0	0	0	0
0	0.00426	0	0	0	0	0	0
0	0.0003	0.00097	0.00625	0	0	0.00213	0
0.00863	0.01575	0.02159	0.0259	0	0.00884	0.03888	0
0.00514	0.00211	0	0.00414	0	0	0	0
0	0	0	0	0	0	0	0
0.01341	0.0515	0	0.11486	0.00533	0.05626	0.02811	0
0	0	0	0	0	0	0	0
0	0	0	0	0	0	0	0
0	0	0	0	0	0	0	0
0	0	0	0	0	0	0	0
0	0	0	0	0	0	0	0
0	0	0	0	0	0	0	0
0	0	0	0	0	0	0	0
0	0	0	0	0	0	0	0
0	0	0	0	0	0	0	0
0	0	0	0	0	0	0	0
0	0	0	0	0	0	0	0
0.00145	0.00115	0	0	0	0.00078	0	0
0	0	0	0	0	0	0	0
20.35225	12.82571	10.41629	10.14805	5.72996	8.1237	10.37048	24.30773
20.35225	12.82571	10.41629	10.14805	5.72996	8.1237	10.37048	24.30773
0	0	0	0.00117	0.00653	0.00103	0	0.00541
0	0	0	0.00117	0.00653	0.00103	0	0.00541
0	0	0	0.00117	0.00653	0.00103	0	0.00541
0	0	0	0.0042	0.07161	0	0	0.00823

0	0	0	0.0042	0.07161	0	0	0.00823
0	0	0	0.0042	0.07161	0	0	0.00823
0.6333	0.06133	0.21903	0.42303	1.21988	0.45702	0.11756	0.27221
0	0	0	0	0	0	0	0
0	0	0	0	0	0	0	0
0.06835	0.04309	0.0127	0.30057	0.11026	0.42914	0.02741	0.23321
0	0	0	0.00395	0	0	0	0
0.00549	0.00099	0.0127	0.04994	0.03437	0.01392	0.00798	0.07979
0	0	0	0	0	0	0	0
0	0	0	0	0	0	0	0
0	0	0	0	0	0	0	0
0	0	0	0	0	0	0	0
0	0	0	0	0	0	0	0
0	0	0	0	0	0	0	0
0	0	0	0	0	0	0	0
0	0	0	0.17314	0.07589	0.07166	0.01943	0.15342
0	0	0	0	0	0	0	0
0.06286	0.0421	0	0	0	0	0	0
0	0	0	0.07354	0	0.34356	0	0
0	0	0	0	0	0	0	0
0	0	0	0	0	0	0	0
0.56495	0.01824	0.20633	0.12128	1.10935	0.02787	0.09015	0.03901
0	0	0	0	0.00092	0	0	0
0.56495	0.01824	0.20633	0.12128	1.10842	0.02787	0.09015	0.03901
0	0	0	0.00117	0.00028	0	0	0
0	0	0	0.00117	0.00028	0	0	0
0	0.00499	0	0	0	0	0	0
0	0.00244	0	0	0	0	0	0
0	0	0	0	0	0	0	0
0	0	0	0	0	0	0	0
0	0	0	0	0	0	0	0
0	0.00244	0	0	0	0	0	0
0	0.00255	0	0	0	0	0	0
0	0.00255	0	0	0	0	0	0
0.33419	0.0236	0.1495	0.00774	0.00473	0.02754	0.81247	0.59794

0	0	0	0.00586	0.00025	0.00036	0.2699	0.11726
0	0	0	0.00586	0.00025	0.00036	0.2699	0.11726
0.31082	0.0236	0.04463	0.00177	0	0.01194	0.54242	0.46824
0.31082	0.0236	0.04463	0.00177	0	0.01194	0.54242	0.46824
0.02337	0	0.10487	0.00011	0.00449	0.01524	0.00015	0.01243
0.02337	0	0.10487	0.00011	0.00449	0.01524	0.00015	0.01243
0	0	0	0	0	0	0	0
0	0	0	0	0	0	0	0
0.50604	0.26816	0.4797	2.64224	0.20567	1.76868	0.20571	0.00093
0	0	0	0.00092	0	0	0	0
0	0	0	0.00092	0	0	0	0
0	0	0	0	0	0	0	0
0	0	0	0	0	0	0	0
0.50604	0.26816	0.4797	2.64132	0.20567	1.76868	0.20571	0.00093
0	0	0	0.00169	0.00527	0.01257	0	0
0	0	0	0	0	0	0	0
0	0	0.00261	2.01495	0.18436	0.10646	0.20533	0
0.08084	0.07567	0.38187	0.00363	0	0	0	0
0	0	0	0.00179	0	0	0.00038	0.00093
0.03784	0.06267	0.08332	0.05188	0.00125	0.04623	0	0
0	0	0	0	0	0	0	0
0	0	0	0.32963	0.01478	0.14486	0	0
0.10281	0.04253	0	0.1231	0	0	0	0
0.13833	0.05247	0	0.1128	0	1.45856	0	0
0	0	0	0	0	0	0	0
0.14623	0.03482	0.0119	0.00185	0	0	0	0
0	0	0	0	0	0	0	0
0	0	0	0	0	0	0	0
12.04554	6.74399	1.18152	2.67239	1.58962	3.13796	3.04956	7.79018
0.25955	0.34045	0.01821	0.20297	0.07659	0.00614	0	0
0	0	0	0	0	0.00614	0	0
0.25955	0.34045	0.01821	0.20297	0.07659	0	0	0
0	0	0	0	0	0	0.16678	0.16317
0	0	0	0	0	0	0.16678	0.16317
0.32431	0.44917	0.38014	0.38978	0.36997	0.2062	0.91625	3.27954

0	0	0.00063	0	0	0.00017	0	0
0	0	0	0	0	0	0.00049	0
0	0	0	0.00354	0	0	0	0
0.0546	0.05447	0.06608	0.11238	0.02233	0.03251	0.007	0
0	0	0	0	0	0	0	0.0019
0	0	0	0.00726	0	0	0	0
0.09458	0.34822	0.09455	0.06468	0.00589	0.09013	0.00141	0
0.12355	0.03842	0.21889	0.15625	0.32692	0.08338	0.90735	3.27764
0.05159	0.00807	0	0.04567	0.01483	0	0	0
0	0	0	0	0	0	0	0
0	0	0	0	0	0	0	0
0.0434	0.0224	0.02991	0.30081	0.07482	0.17704	0.06089	0.07637
0.0034	0.0003	0.00655	0.30081	0.07482	0.17704	0.05229	0.07637
0.04	0.02211	0.02336	0	0	0	0.0086	0
0.44222	0.20464	0.09744	0.03563	0.00185	0	0.14619	0.05932
0.22272	0.10869	0.0311	0.03563	0.00185	0	0.12509	0.05558
0.2195	0.09595	0.06634	0	0	0	0	0
0	0	0	0	0	0	0.0211	0.00374
0.02707	0	0.00219	0.02545	0.10921	0.06487	0.11145	0.70908
0	0	0	0.0208	0.10353	0.06121	0.1111	0.66843
0.02707	0	0.00219	0.00466	0.00568	0.00366	0.00036	0.04065
0	0	0	0	0	0.00099	0.00104	0.00573
0	0	0	0	0	0.00099	0.00104	0.00573
1.48929	0.17688	0	0.09822	0.00274	0.45809	0	0
1.48929	0.17688	0	0.09822	0.00274	0.45809	0	0
0	0	0	0	0	0	0	0
0	0	0	0	0	0	0	0
0.22301	0.11967	0.59348	0.74851	0.73904	0.45394	1.63513	3.41949
0.00439	0.00014	0.00511	0.16314	0.02961	0.01539	0.00834	0.00792
0.06166	0.01953	0.02356	0.06447	0.024	0.05023	0.02345	0.0195
0.06044	0.01329	0.124	0.22874	0.16211	0.25627	1.24012	1.54596
0.02979	0.02088	0.11329	0.25008	0.46069	0.08254	0.02056	0.00294
0	0	0	0	0	0	0	0
0	0	0	0	0	0	0	0
0.00356	0.00036	0.00102	0.03515	0.01526	0.04874	0.00234	0.00099

0	0	0	0	0	0	0.10472	1.35169
0	0	0	0.00193	0.00693	0	0	0.00148
0.06257	0.06547	0.32651	0.00502	0.04044	0.00077	0.2356	0.48901
0.00059	0	0	0	0	0	0	0
0	0	0	0	0	0	0	0
2.67562	0.53132	0	0	0	0	0	0
1.45521	0.15786	0.00031	0	0	0	0	0
1.45521	0.15786	0.00031	0	0	0	0	0
0	0	0	0	0	0	0	0
0	0	0	0	0	0	0	0
2.67562	0.53132	0	0	0	0	0	0
0	0	0	0	0	0	0	0
0	0	0	0	0	0	0	0
0	0	0	0	0	0.0016	0.00109	0.00307
0	0	0	0	0	0.0016	0.00109	0.00307
0	0	0	0	0	0	0	0
0	0	0	0	0	0	0	0
0	0	0	0	0	0	0	0
0	0	0	0	0	0	0	0
0	0	0	0	0	0	0	0
5.1056	4.74158	0.00238	0.84743	0	1.7376	0.00417	0
4.03619	4.60057	0	0.26254	0	0.58979	0	0
0.10501	0.0225	0	0.19303	0	0.07667	0	0
0.01713	0.0021	0	0.01951	0	1.07113	0	0
0.94694	0.11641	0.00238	0.37236	0	0	0.00417	0
0	0	0	0	0	0	0	0
0.00033	0	0	0	0	0	0	0
0	0	0.05745	0.02357	0.21539	0.03151	0.00657	0.07439
0	0	0.05745	0.02357	0.21539	0.03151	0.00657	0.07439
0	0	0	0	0	0	0	0
0	0	0	0	0	0	0	0
0	0	0	0	0	0	0	0
0	0	0	0	0	0	0	0
0.00025	0	0	0	0	0	0	0
0.00025	0	0	0	0	0	0	0

0	0	0	0	0	0	0.83472	0.61613
0	0	0	0	0	0	0.83472	0.61613
0	0	0	0	0	0	0.83472	0.61578
0	0	0	0	0	0	0	0.00035
0	0.00135	0.03229	0	0.0454	0.02548	0.30877	0.05477
0	0	0	0	0	0	0.30877	0.05477
0	0	0	0	0	0	0.30877	0.05477
0	0	0	0	0	0	0	0
0	0	0	0	0	0	0	0
0	0	0.03229	0	0.0155	0.02548	0	0
0	0	0.03229	0	0.0155	0.02548	0	0
0	0	0	0	0	0	0	0
0	0	0	0	0	0	0	0
0	0	0	0	0	0	0	0
0	0	0	0	0	0	0	0
0	0.00135	0	0	0.0299	0	0	0
0	0	0	0	0	0	0	0
0	0.00135	0	0	0.0299	0	0	0
0	0	0	0	0	0	0	0
0	0	0	0	0	0	0	0
0	0	0	0	0	0	0	0
0	0	0	0	0	0	0	0
6.83319	5.72229	8.35424	4.39728	2.58653	2.70599	5.04169	14.96194
0.10757	0.08665	0.27755	0.05324	0	0.12313	0.00738	0
0.10757	0.08665	0.27755	0.05324	0	0.12313	0.00738	0
0	0	0	0.00033	0.00159	0	0.00066	0.00569
0	0	0	0.00033	0.00159	0	0.00066	0.00569
0.00058	0.00137	0.00201	0	0	0	0	0.00081
0.00058	0.00137	0.00201	0	0	0	0	0.00081
0.06478	0.02244	0.01733	0.27002	0.05112	0.00976	0.24661	0.62845
0.06478	0.02244	0.01733	0.27002	0.05112	0.00976	0.24661	0.62845
4.70961	3.9721	2.59366	2.12451	0.6104	0.69732	3.70079	11.29004
4.70961	3.9721	2.59366	2.12451	0.6104	0.69732	3.70079	11.29004
0.44761	0.01726	1.92887	0.04427	0.04656	0.14237	0.38519	0.70803
0.44761	0.01726	1.92887	0.04427	0.04656	0.14237	0.38519	0.70803

0	0	0	0	0	0	0	0
0.04762	0.03315	0.00289	0.14989	0.05535	1.52458	0	0
0.04762	0.03315	0.00289	0.14989	0.05535	1.52458	0	0
0	0	0	0.00342	0	0.02775	0	0.00072
0	0	0	0.00342	0	0.02775	0	0.00072
0.00202	0	0.00175	0.00143	0.02294	0.00054	0	0
0.00113	0	0.00175	0.00085	0	0.00054	0	0
0.00089	0	0	0.00057	0.02294	0	0	0
0.01303	0.05396	0.026	1.56485	0.91074	0.02556	0.27745	1.07218
0.01303	0.05396	0.026	0.00875	0.00007	0.00887	0	0
0	0	0	0.00531	0.0136	0	0.00535	0.01512
0	0	0	1.21743	0.00976	0.01669	0.02979	0
0	0	0	0.00006	0	0	0.24231	1.05706
0	0	0	0.33331	0.8873	0	0	0
1.37568	1.4713	3.25476	0.00912	0	0	0.00083	0
0	0	0	0.00912	0	0	0	0
1.20212	1.42996	2.96862	0	0	0	0.00083	0
0	0	0	0	0	0	0	0
0.17356	0.04134	0.28614	0	0	0	0	0
0	0	0	0	0	0	0	0
0.0647	0.06405	0.24943	0.1762	0.88783	0.15497	0.42278	1.25602
0.0647	0.06405	0.24943	0.1762	0.88783	0.15497	0.42278	1.25602
0.08474	0.06441	0.57774	0.06968	0.99824	0.08716	0.14782	0.14044
0.08474	0.06441	0.57774	0.06968	0.99824	0.08716	0.14782	0.14044
0.08474	0.06441	0.57774	0.06968	0.99824	0.08716	0.14782	0.14044
0	0	0	0	0	0	0	0
0	0	0	0	0	0	0	0
0	0	0	0	0	0	0	0
0	0	0	0	0	0	0	0
0	0	0	0	0	0	0.02326	0
0	0	0	0	0	0	0.02326	0
0.05099	0.00543	0.26105	0.0008	0	0	0	0
0.05099	0.00543	0.26105	0.0008	0	0	0	0
0	0	0.01869	0.00031	0	0.00428	0.03016	0.06733
0	0	0.01869	0.00031	0	0.00428	0.03016	0.06733

0	0	0	0	0	0	0	0
0	0	0	0	0	0	0	0
0.02426	0.04534	0.29801	0.03932	0.16093	0.07572	0.09269	0.05899
0.02426	0.04369	0.03504	0	0.09315	0.02459	0	0
0	0	0	0	0	0	0.0026	0
0	0.00165	0.26297	0.03932	0.06777	0.05113	0.0901	0.05899
0	0	0	0	0.8155	0.00716	0	0
0	0	0	0	0.8155	0.00716	0	0
0	0	0	0	0	0	0	0
0	0	0	0	0	0	0	0
0	0	0	0.01508	0.01311	0	0	0
0	0	0	0.01508	0.01311	0	0	0
0.00852	0.01364	0	0	0	0	0	0
0.00852	0.01364	0	0	0	0	0	0
0	0	0	0.01417	0.0087	0	0.00171	0.01413
0	0	0	0.01417	0.0087	0	0.00171	0.01413
0.00098	0	0	0	0	0	0	0
0.00098	0	0	0	0	0	0	0
0	0	0	0	0	0	0	0
0	0	0	0	0	0	0	0
0	0	0	0	0	0	0	0
0	0	0	0	0	0	0	0
0.94228	0.59734	0.03935	1.32303	2.01548	0.42272	0.11853	0.58767
0.94228	0.59734	0.03935	1.32303	2.01548	0.42272	0.11853	0.58767
0.94228	0.59734	0.03935	1.32303	2.01548	0.42272	0.11853	0.58767
0.94228	0.59734	0.03935	1.32303	2.01548	0.42272	0.11853	0.58767
0.45704	0.23679	0	0.05749	0.00443	0	0	0
0.19129	0.27553	0.00132	0	0	0	0	0
0	0	0	0	0	0	0	0
0	0	0	0	0	0	0	0
0	0	0	0	0	0	0	0
0.29395	0.0835	0.03803	1.26554	2.01106	0.42272	0.11853	0.58767
0	0.00152	0	0	0	0	0	0
0	0	0	0	0	0	0	0
0.0088	0.04198	0.06083	0.95154	1.34803	2.3418	1.1032	0.57127

0	0	0	0.92845	1.34803	2.27674	0.58727	0.26684
0	0	0	0.92845	1.34803	2.27674	0.58727	0.26684
0	0	0	0	0	0	0	0
0	0	0	0	0	0	0	0
0	0	0	0	0	0	0	0
0	0	0	0.92845	1.34803	2.27674	0.58727	0.26684
0	0	0	0.92845	1.34803	2.27674	0.58727	0.26684
0	0	0	0	0	0	0	0
0.00013	0	0	0	0	0	0.30405	0.25768
0.00013	0	0	0	0	0	0.30405	0.25768
0	0	0	0	0	0	0.30405	0.25768
0	0	0	0	0	0	0	0
0	0	0	0	0	0	0.30405	0.25768
0	0	0	0	0	0	0	0
0	0	0	0	0	0	0	0
0.00013	0	0	0	0	0	0	0
0	0	0	0	0	0	0	0
0.00013	0	0	0	0	0	0	0
0	0	0	0	0	0	0	0
0	0	0	0	0	0	0	0
0.00867	0.04198	0.06083	0.02309	0	0.06507	0.21188	0.04675
0.00867	0.04198	0.06083	0.02309	0	0.06507	0.21188	0.04675
0	0	0	0	0	0	0	0
0	0	0	0	0	0	0	0
0	0	0	0	0	0	0	0
0	0	0	0	0	0	0	0
0.00073	0.00157	0	0.00736	0	0	0	0
0.00073	0.00157	0	0	0	0	0	0
0	0	0	0	0	0	0	0
0	0	0	0.00736	0	0	0	0
0	0	0	0	0	0	0.00189	0.00485
0	0	0	0	0	0	0.00189	0.00485
0	0	0	0	0	0	0	0
0	0	0	0	0	0	0	0
0	0	0	0	0	0	0	0

0	0	0	0	0	0	0	0
0.00794	0.04041	0.06083	0.01573	0	0.06507	0.20998	0.0419
0.0001	0.00334	0	0.0106	0	0.05449	0.00088	0
0	0.00204	0	0	0	0	0.00907	0
0	0	0	0	0	0	0	0
0.00784	0.03263	0.06083	0.00483	0	0.01057	0.19826	0.0419
0	0	0	0	0	0	0	0
0	0	0	0	0	0	0	0
0	0.0024	0	0.0003	0	0	0.00177	0
0	0	0	0	0	0	0	0
0	0.00184	0	0.00454	0.00773	0	0	0
0	0.00184	0	0.00454	0.00773	0	0	0
0	0.00184	0	0.00454	0.00773	0	0	0
0	0	0	0	0	0	0	0
0	0	0	0	0	0	0	0
0	0	0	0	0	0	0	0
0	0	0	0	0	0	0	0
0	0	0	0	0	0	0	0
0	0.00184	0	0.00454	0.00773	0	0	0
0	0.00184	0	0.00454	0.00773	0	0	0
0	0	0	0	0	0	0	0
0	0	0	0	0	0	0	0
0	0	0	0	0	0	0	0
0	0	0	0	0	0	0	0
0	0	0	0	0	0	0	0
0.37732	1.87531	0.40034	1.37758	0.15461	2.00552	0	0
0.37732	1.87531	0.40034	1.37758	0.15461	2.00552	0	0
0.37732	1.87531	0.40034	1.37758	0.15461	2.00552	0	0
0.37732	1.87531	0.40034	1.37758	0.15461	2.00552	0	0
0	0	0	0	0	0	0	0
0	0	0	0	0	0	0	0
0.37732	1.87531	0.40034	1.37758	0.15461	2.00552	0	0
0	0	0	0	0	0	0	0
0.37732	1.87531	0.40034	0	0	0	0	0
0	0	0	0	0	0	0	0

0	0	0	0.00191	0	0	0	0
0	0	0	0	0	0	0	0
0	0	0	0	0	0	0	0
0	0	0	0.00077	0	0.01206	0	0
0	0	0	1.3749	0.15461	1.99346	0	0
0	0	0	0	0	0	0	0
0	0	0	0	0	0	0	0
0	0	0	0	0	0	0	0
0	0	0	0	0	0	0	0
0	0	0	0	0	0	0	0
0	0	0	0	0	0	0	0
0	0	0	0	0	0	0	0
0	0	0	0.00321	0.00584	0	0.00047	0
0	0	0	0.00321	0.00584	0	0.00047	0
0	0	0	0.00321	0.00584	0	0.00047	0
0	0	0	0.00321	0.00584	0	0.00047	0
0	0	0	0.00321	0.00584	0	0.00047	0
0	0	0	0.00321	0.00584	0	0.00047	0
0.36334	0.12318	2.17887	6.47046	2.73987	8.27213	4.08496	3.00456
0.05784	0.00225	0.27697	0.34987	0.38383	2.75243	0.00169	0
0.05784	0.00225	0.27697	0.34987	0.38383	2.74904	0.00169	0
0	0	0	0	0	0	0	0
0	0	0	0	0	0	0	0
0	0	0	0	0	0	0	0
0	0	0	0	0	0	0	0
0	0	0	0	0	0	0	0
0	0	0	0	0	0	0	0
0	0	0	0.10185	0.04334	0.24474	0.00169	0
0	0	0	0.10185	0.04334	0.24474	0.00169	0
0	0	0	0.10185	0.04334	0.24474	0.00169	0
0.05784	0.00225	0.27697	0.24802	0.34049	2.5043	0	0
0.04186	0.00225	0.21854	0.24755	0.33805	2.49497	0	0
0.04186	0.00225	0.21854	0.24755	0.33805	2.49497	0	0
0	0	0	0	0	0	0	0
0	0	0	0	0	0	0	0

0.01598	0	0.05842	0.00047	0.00244	0.00933	0	0
0.01598	0	0.05842	0.00047	0.00244	0.00933	0	0
0	0	0	0	0	0.00339	0	0
0	0	0	0	0	0.00339	0	0
0	0	0	0	0	0	0	0
0	0	0	0	0	0	0	0
0	0	0	0	0	0.00339	0	0
0	0	0	0	0	0	0	0
0	0	0	0	0	0.00339	0	0
0	0	0	0	0	0	0	0
0	0	0	0	0	0	0	0
0	0	0	0	0	0	0	0
0	0.00671	0.32578	0.19391	0.3705	0.17583	0.01646	0.16173
0	0.00671	0.32578	0.19391	0.3705	0.17583	0.01646	0.16173
0	0.00671	0.32578	0.19391	0.3705	0.17583	0.01646	0.16173
0	0.00671	0.32578	0.16736	0.10449	0.17583	0.01646	0.16173
0	0.00671	0.32578	0.16736	0.10449	0.17583	0.01646	0.16173
0	0	0	0.02655	0.26601	0	0	0
0	0	0	0.02655	0.26601	0	0	0
0	0	0	0	0	0	0	0
0	0	0	0	0	0	0	0
0	0	0	0	0	0	0	0
0	0	0	0	0	0	0	0
0	0	0	0	0	0	0	0
0	0	0	0	0	0	0	0
0	0	0	0	0	0	0	0
0	0	0	0	0	0	0	0
0	0	0	0	0	0	0	0
0	0	0	0	0	0	0	0
0	0	0	0	0	0	0	0
0	0	0	0	0	0	0	0
0	0	0	0	0	0	0	0
0	0	0	0	0	0	0	0
0.0032	0.09425	0.00028	5.91638	1.97507	5.23719	4.06681	2.84283
0	0	0	0.14571	0.02255	0.46769	0	0

0	0	0	0	0	0	0	0
0	0	0	0	0	0	0	0
0	0	0	0	0	0	0	0
0	0	0	0	0	0	0	0
0	0	0	0	0	0	0	0
0	0	0	0.14571	0.02255	0.46769	0	0
0	0	0	0.14571	0.02255	0.46769	0	0
0	0	0	0.14571	0.02255	0.46769	0	0
0	0	0	0	0	0	0	0
0	0	0	0	0	0	0	0
0	0	0	0	0	0	0	0
0	0	0	0	0	0	0	0
0	0	0	0	0	0	0	0
0.0032	0.09425	0.00028	5.75267	1.95252	4.76905	4.06395	2.84208
0	0	0	0	0	0	0	0
0	0	0	0	0	0	0	0
0	0	0	0	0	0	0	0
0.0032	0.09425	0.00028	5.75267	1.95252	4.76905	4.06395	2.84208
0	0	0	0.00131	0.00163	4.72256	0	0
0	0	0	0.00131	0.00163	4.72256	0	0
0	0	0	0	0	0	0	0
0	0	0	0	0	0	0	0
0	0	0	0	0	0	0	0
0	0	0	0	0	0	0	0
0	0	0	0	0	0	0	0
0	0	0	0	0	0	0	0
0	0	0	0	0	0	0	0
0	0	0	0	0	0	0	0
0	0	0	0	0	0	0	0
0	0	0	0	0	0	0	0
0	0	0	0	0	0	0	0
0	0	0	0	0	0	0	0
0	0	0	1.39553	0.54492	0	0	0
0	0	0	1.39553	0.54492	0	0	0
0	0	0	0	0	0	0	0

0	0	0	0	0	0	0	0
0.0032	0.09366	0.00028	0.73614	0.41693	0.00522	3.96978	2.84208
0.0032	0.09366	0.00028	0.73614	0.41693	0.00522	3.96978	2.84208
0	0	0	0	0	0	0	0
0	0.00059	0	3.61969	0.98903	0.04126	0.09418	0
0	0	0	0	0	0	0	0
0	0	0	0	0	0	0	0
0	0	0	0.00378	0.00241	0	0	0
0	0	0	2.72204	0.76559	0.02784	0.07395	0
0	0.00059	0	0.23	0.05803	0.00297	0.0092	0
0	0	0	0.66387	0.16301	0.01045	0.01104	0
0	0	0	0	0	0	0	0
0	0	0	0	0	0	0	0
0	0	0	0	0	0	0	0
0	0	0	0	0	0	0	0
0	0	0	0	0	0	0	0
0	0	0	0	0	0	0	0
0	0	0	0	0	0	0	0
0	0	0	0	0	0	0	0
0	0	0	0	0	0	0	0
0	0	0	0	0	0	0	0
0	0	0	0	0	0	0	0
0	0	0	0	0	0	0	0
0	0	0	0	0	0	0	0
0	0	0	0	0	0	0	0
0	0	0	0	0	0	0	0
0	0	0	0	0	0	0	0
0	0	0	0.00152	0	0.00046	0	0
0	0	0	0.00152	0	0.00046	0	0
0	0	0	0	0	0	0	0
0	0	0	0	0	0	0	0
0	0	0	0	0	0	0	0
0	0	0	0.00152	0	0.00046	0	0
0	0	0	0	0	0	0	0
0	0	0	0	0	0	0	0

0	0	0	0.00152	0	0.00046	0	0
0	0	0	0	0	0	0	0
0	0	0	0	0	0	0	0
0	0	0	0	0	0	0	0
0	0	0	0	0	0	0	0
0	0	0	0.01649	0	0	0.00285	0.00075
0	0	0	0	0	0	0	0
0	0	0	0	0	0	0	0
0	0	0	0	0	0	0	0
0	0	0	0.01649	0	0	0.00285	0.00075
0	0	0	0.01649	0	0	0.00285	0.00075
0	0	0	0.01649	0	0	0.00285	0.00075
0.3023	0.01997	1.57585	0.0103	0.01047	0.10668	0	0
0.3023	0.01997	1.57585	0.0103	0.01047	0.10668	0	0
0.3023	0.01997	1.57585	0.0103	0.01047	0.10668	0	0
0.3023	0.01997	1.57585	0.0103	0.01047	0.10668	0	0
0.3023	0.01997	1.57585	0.0103	0.01047	0.10668	0	0
0.09792	0.02809	0	0.83099	1.62776	0	0.05255	0.12432
0.09792	0.02809	0	0.83099	1.62776	0	0.05255	0.12432
0.09792	0.02809	0	0.83099	1.62776	0	0.05255	0.12432
0.09792	0.02809	0	0.83099	1.62776	0	0.05255	0.12432
0	0.00205	0	0.83099	1.62776	0	0.05255	0.12432
0	0.00205	0	0.07493	0.27317	0	0	0.00133
0	0	0	0.75606	1.3546	0	0.05255	0.12298
0	0	0	0	0	0	0	0
0	0	0	0	0	0	0	0
0.09792	0.02604	0	0	0	0	0	0
0.097	0.02604	0	0	0	0	0	0
0.00092	0	0	0	0	0	0	0
0	0	0	0	0	0	0	0
0	0	0	0	0	0	0	0
0	0	0	0	0	0	0	0
0	0	0	0	0	0	0	0
0	0	0	0	0	0	0	0
0	0	0	0	0	0	0	0

5.69111	3.68217	14.86942	2.99173	12.00023	0.00114	0.0008	0
5.69111	3.68217	14.86942	2.99173	12.00023	0.00114	0.0008	0
5.69111	3.68217	14.86942	2.99173	12.00023	0.00114	0.0008	0
5.69111	3.68217	14.86942	2.99173	12.00023	0.00114	0.0008	0
5.69111	3.68217	14.86942	2.99173	12.00023	0.00114	0.0008	0
5.69111	3.68217	14.86942	2.99173	12.00023	0.00114	0.0008	0
0	0	0	0	0	0	0.00134	0.00278
0	0	0	0	0	0	0.00134	0.00278
0	0	0	0	0	0	0.00134	0.00278
0	0	0	0	0	0	0.00134	0.00278
0	0	0	0	0	0	0.00134	0.00278
0	0	0	0	0	0	0.00134	0.00278
0	0	0	0	0	0	0.00134	0.00278
0	0	0	0	0	0	0	0
0	0	0	0	0	0	0	0
0	0	0	0	0	0	0	0
0	0.06022	0	0	0	0	0	0
0	0.06022	0	0	0	0	0	0
0	0.06022	0	0	0	0	0	0
0	0.06022	0	0	0	0	0	0
0	0.06022	0	0	0	0	0	0
0	0.06022	0	0	0	0	0	0
0	0.06022	0	0	0	0	0	0
0	0	0	0	0	0	0	0
0	0	0	0	0	0	0	0
0	0	0	0	0	0	0	0

AP014_3	AP015_1	AP015_2	AP015_3	AP016_1	AP016_2	AP016_3	AP017_1
100	100	100	99.99793	100	99.9931	100	100
0.33046	0.24194	2.31275	0.02717	7.69993	1.51122	1.00014	1.18447
0.07573	0.07188	1.66027	0.02717	7.42573	1.49648	0.59534	0.99697
0.01159	0.01957	0.05427	0.00102	0.03139	0.03572	0.0416	0.00914
0.01159	0.01957	0.05427	0.00102	0.03139	0.03572	0.0416	0.00914
0	0	0	0	0.00069	0	0	0
0	0	0	0	0.00069	0	0	0
0.01159	0.01957	0.05427	0.00102	0.03071	0.03572	0.0416	0.00914
0	0.00024	0.00147	0	0.00057	0	0	0
0	0	0	0	0	0	0.00465	0
0	0	0	0	0	0	0	0
0	0	0	0	0	0	0	0
0	0	0	0	0	0	0	0
0	0	0	0	0	0	0	0
0.01015	0.00391	0.00949	0	0.00699	0.01539	0.00312	0.00457
0	0	0	0	0	0	0.00075	0
0.00144	0	0	0	0.00234	0.00417	0.00162	0.00269
0	0.00056	0	0	0.00057	0.00343	0	0
0	0.00799	0.00204	0	0	0	0.03056	0
0	0.00044	0.01155	0	0.00765	0.0022	0	0
0	0	0.00024	0	0	0	0	0
0	0.00302	0.02464	0.00102	0.01259	0.01054	0.00065	0.00189
0	0	0.001	0	0	0	0	0
0	0.0034	0.00384	0	0	0	0.00009	0
0	0	0	0	0	0	0.00017	0
0	0	0	0	0	0	0	0
0	0.03593	1.52595	0.00202	7.37838	1.45871	0.55373	0.98087
0	0.03593	1.52595	0.00202	7.37838	1.45871	0.55373	0.98087
0	0	0	0	0	0	0	0
0	0	0	0	0	0	0	0
0	0	0	0	0	0	0	0
0	0	0	0	0	0	0	0
0	0.03548	1.5051	0.00202	7.37838	1.45871	0.55373	0.98087

0	0.00018	0	0.00029	0	0	0	0.59948
0	0	0	0	0	0	0	0
0	0	0	0	0	0	0	0.16019
0	0	0	0	0	0	0	0.00831
0	0	0	0	0.00149	0	0.00464	0
0	0.01023	1.28483	0.00173	7.35104	1.45871	0.31434	0.15367
0	0.02507	0.22027	0	0.02585	0	0.23476	0.05923
0	0	0	0	0	0	0	0
0	0	0	0	0	0	0	0
0	0.00046	0.02085	0	0	0	0	0
0	0.00046	0.02085	0	0	0	0	0
0	0	0.00037	0	0	0	0	0
0	0	0.00037	0	0	0	0	0
0	0	0.00037	0	0	0	0	0
0	0	0.00037	0	0	0	0	0
0	0	0	0	0	0	0	0
0	0	0	0	0	0	0	0
0.06413	0	0.02336	0.02413	0.00562	0.00205	0	0.00695
0.06413	0	0.02336	0.02413	0.00562	0.00205	0	0.00695
0.06413	0	0.02336	0.02413	0.00562	0.00205	0	0.00695
0.00278	0	0	0	0	0	0	0
0.00156	0	0.00215	0	0	0	0	0
0.0598	0	0.0212	0.02413	0.00562	0.00205	0	0.00695
0	0.01638	0.05633	0	0.01034	0	0	0
0	0.01638	0.05633	0	0.01034	0	0	0
0	0.01638	0.05633	0	0.01034	0	0	0
0	0.01638	0.05633	0	0.01034	0	0	0
0	0	0	0	0	0	0	0
0	0	0	0	0	0	0	0
0.25473	0.17006	0.65248	0	0.2742	0.01474	0.40481	0.1875
0.12772	0.11755	0.3596	0	0.01822	0.01218	0.39374	0.09937
0	0	0.00752	0	0.01674	0.01218	0.00165	0.01048
0	0	0.00752	0	0.01674	0.01053	0.00165	0.01048
0	0	0	0	0	0	0	0
0	0	0.00448	0	0.01205	0.00779	0.00165	0.00875

0	0	0.00304	0	0.00469	0.00274	0	0.00173
0	0	0	0	0	0.00165	0	0
0	0	0	0	0	0	0	0
0	0	0	0	0	0.00165	0	0
0.12772	0.11755	0.35208	0	0.00149	0	0.39209	0.0889
0.12586	0.11697	0.24456	0	0.00149	0	0.39209	0.0889
0.12586	0.10601	0.21397	0	0.00149	0	0.39107	0.0879
0	0.00672	0.01473	0	0	0	0	0.00022
0	0.00424	0.01586	0	0	0	0.00102	0.00077
0.00186	0.00058	0.10752	0	0	0	0	0
0	0	0.0014	0	0	0	0	0
0.00186	0.00058	0.10612	0	0	0	0	0
0.12701	0.05251	0.29288	0	0.25598	0.00256	0.01107	0.08813
0.12701	0.05251	0.29288	0	0.25598	0.00256	0.01107	0.08813
0.01151	0	0	0	0	0	0	0
0.01151	0	0	0	0	0	0	0
0.0017	0	0	0	0	0	0	0
0.0017	0	0	0	0	0	0	0
0.10509	0.04691	0.2309	0	0.23544	0.00256	0.01068	0.08291
0.10509	0.04691	0.2309	0	0.23544	0.00256	0.01068	0.08291
0	0	0	0	0	0	0	0
0	0	0	0	0	0	0	0
0.00871	0.00559	0.06198	0	0.01995	0	0.00039	0.00522
0.00871	0.00559	0.06198	0	0.01995	0	0.00039	0.00522
0	0	0	0	0.00058	0	0	0
0	0	0	0	0.00058	0	0	0
84.89056	79.49305	38.11772	90.0085	64.82559	82.12959	49.32522	78.3065
0	0	0	0	0	0	0	0
0	0	0	0	0	0	0	0
0	0	0	0	0	0	0	0
0	0	0	0	0	0	0	0
0	0	0	0	0	0	0	0
84.89056	79.49305	38.11772	90.0085	64.82559	82.12959	49.32522	78.3065
84.89056	79.49305	38.11772	90.0085	64.82559	82.12959	49.32522	78.3065
71.23631	72.29152	28.3077	89.45041	60.97076	70.90102	48.06776	75.9397

71.23631	72.29152	28.3077	89.45041	60.97076	70.90102	48.06776	75.9397
0.31041	0.28613	0.06615	0	0	0	0	0.37801
0.08485	0	0	0	0	0	0	0
0	0	0	0	0	0	0	2.75007
1.44424	26.37339	9.31941	12.94628	0.00276	0.00563	0.00103	0.00469
0	0.26623	0.27308	0	0	0	0	0
0	0	0	0	0	0	0	0
0	0	0	0	0	0	0	0
0	0.34269	0.10412	0	0	0	0	0
14.79649	0.02289	0.00193	0.00124	1.23497	0.68097	1.34597	16.31488
17.93974	0	0	0	0	0	0	0.01353
0	3.35361	1.39286	2.4066	0	0	0	0
0.1203	0	0.00135	0	0.10745	0.00878	0.01652	0
0.6417	0.11031	0.07925	0.01344	0	0	0	4.90614
2.65937	5.73385	0.65194	48.80892	0.0004	0.00186	0.00276	0
0	0.04884	0.07041	0	0	0	0	0
0	0	0	0	0	0	0	0
1.68767	0	0.00465	0	0	0	0	0
9.73317	16.64277	11.56074	18.97444	0.00217	0.00593	0.00152	4.97372
6.7793	0.59188	0.61056	0.01011	14.36695	0.38301	1.1733	18.88773
15.01483	1.45689	1.71212	0.00038	0.71504	0.03061	0.84227	0.42752
0.00945	16.91848	2.41408	0.72306	42.59127	68.67609	42.96872	27.277
0.01478	0.14359	0.04505	5.56595	1.94974	1.10813	1.71566	0.00641
0.05565	0.05322	0.19109	0	0	0	0	0
0	0.05322	0.19109	0	0	0	0	0
0	0.05322	0.19109	0	0	0	0	0
0.05565	0	0	0	0	0	0	0
0	0	0	0	0	0	0	0
0.05565	0	0	0	0	0	0	0
2.4802	1.84036	1.49513	0	0	0	0	0.07504
1.21375	1.66477	1.3199	0	0	0	0	0.07504
0.00142	1.05109	0.80669	0	0	0	0	0.0377
1.21233	0.61368	0.5132	0	0	0	0	0.03734
1.26645	0.17559	0.17523	0	0	0	0	0
0	0	0	0	0	0	0	0

1.26645	0.17559	0.17523	0	0	0	0	0
0	0	0	0	0	0	0	0.00071
0	0	0	0	0	0	0	0.00071
0	0	0	0	0	0	0	0
0	0	0	0	0	0	0	0.00071
0	0	0	0	0	0	0	0
0	0	0	0	0	0	0	0
0	0	0	0	0	0	0	0
0.00089	0.37418	0.09393	0	0.00054	0	0	0.04802
0	0	0	0	0	0	0	0.02291
0	0	0	0	0	0	0	0.02291
0	0.16076	0.08678	0	0	0	0	0
0	0	0	0	0	0	0	0
0	0.16076	0.08678	0	0	0	0	0
0.00089	0.21342	0.00714	0	0.00054	0	0	0.0251
0.00089	0.21342	0.00714	0	0.00054	0	0	0
0	0	0	0	0	0	0	0
0	0	0	0	0	0	0	0
0	0	0	0	0	0	0	0
0	0	0	0	0	0	0	0
0	0	0	0	0	0	0	0.00038
0	0	0	0	0	0	0	0
0	0	0	0	0	0	0	0.00426
0	0	0	0	0	0	0	0.00012
0	0	0	0	0	0	0	0
0	0	0	0	0	0	0	0.00049
0	0	0	0	0	0	0	0.01901
0	0	0	0	0	0	0	0.00084
0	0	0	0	0	0	0	0
0	0	0	0	0	0	0	0
0	0	0	0	0	0	0	0
0	0	0	0	0	0	0	0
0	0	0	0	0	0	0	0
7.62715	0.20501	0.81063	0	0.06224	0	0.00075	0.8665
7.62715	0.20501	0.81063	0	0.06224	0	0.00075	0.8665

0.4079	0.18663	0.62944	0	0	0	0	0.00386
0.06747	0.0031	0	0	0.06224	0	0.00075	0.00487
0	0	0	0	0	0	0	0.00907
3.62123	0	0	0	0	0	0	0.68654
3.53054	0.01528	0.18118	0	0	0	0	0.16217
0	0	0	0	0	0	0	0
3.49036	4.72875	7.21926	0.55809	3.79206	11.22857	1.25671	1.37653
3.49036	4.72875	7.21926	0.55809	3.79206	11.22857	1.25671	1.37653
2.43429	3.89968	6.54178	0.24576	3.79206	11.22857	1.25671	1.16159
0.24284	0.39053	0.60139	0	0	0	0	0.21494
0.59147	0.00592	0.00189	0	0	0	0	0
0	0.20637	0.04843	0.31233	0	0	0	0
0.21784	0.22625	0.02577	0	0	0	0	0
0.00392	0	0	0	0	0	0	0
0	0	0	0	0	0	0	0
0	0	0	0	0	0	0	0
0	0	0	0	0	0	0	0
0	0	0	0	0	0	0	0
0	0	0	0	0	0	0	0
0	0	0	0	0	0	0	0
14.4336	15.316	57.46831	3.00713	8.72926	2.21131	32.44653	19.17693
0.34241	0.09054	0.37308	0.14174	0.52628	0.60341	3.14806	1.74047
0.00458	0.00554	0.00772	0.0017	0.00631	0.00243	0.00345	0.00339
0.00458	0.00554	0.00772	0.0017	0.00631	0.00243	0.00345	0.00339
0.00458	0.00554	0.00772	0.0017	0.00631	0.00243	0.00345	0.00339
0	0.00017	0	0	0	0	0	0
0	0	0.0006	0	0	0	0	0
0	0	0	0	0.00039	0	0.00225	0
0.00458	0.00536	0.00712	0.0017	0.00591	0.00243	0.00121	0.00339
0.33783	0.08501	0.36537	0.14004	0.51997	0.60097	3.14461	1.73709
0	0	0	0	0	0.00902	0	0
0	0	0	0	0	0.00902	0	0
0	0	0	0	0	0.00269	0	0
0	0	0	0	0	0.00633	0	0
0	0	0.00199	0	0.00222	0.00199	0	0.02151

0	0	0.00199	0	0.00222	0.00199	0	0.02151
0	0	0.00199	0	0.00222	0.00199	0	0.0165
0	0	0	0	0	0	0	0.00501
0.2977	0	0.27837	0	0.05224	0.27237	0	0
0.2977	0	0.27837	0	0.05224	0.27237	0	0
0	0	0	0	0	0	0	0
0	0	0	0	0.02773	0.02226	0	0
0	0	0	0	0	0	0	0
0.2977	0	0.27837	0	0.0189	0.25011	0	0
0	0	0	0	0.0056	0	0	0
0	0	0	0	0	0	0	0
0	0	0	0	0	0	0	0
0	0	0	0	0	0	0	0
0	0	0	0	0	0	0	0
0	0	0	0	0	0	0	0
0	0	0	0	0.3451	0.00354	0	0
0	0	0	0	0.3451	0.00354	0	0
0	0	0	0	0	0	0	0
0	0	0	0	0	0	0	0
0	0	0	0	0.3451	0.00354	0	0
0	0	0	0	0	0	0	0
0	0	0	0	0	0	0	0
0	0	0	0	0	0	0	0
0	0	0	0	0	0	0	0
0	0	0	0	0	0	0	0
0	0	0	0	0	0	0	0
0	0	0	0	0	0	0	0
0	0	0	0	0	0	0	0
0	0	0	0	0	0	0	0
0	0	0	0	0	0	0	0
0	0	0	0	0	0	0	0
0	0	0	0	0	0	0	0
0	0	0	0	0	0	0	0
0	0	0	0	0	0	0	0
0.04013	0.08501	0.08501	0.14004	0.12042	0.31404	3.14461	1.71558

0.04013	0.08501	0.08501	0.14004	0.12042	0.31404	3.14461	1.71558
0.02652	0	0	0	0	0	0	0.04085
0.01361	0	0	0.00191	0	0.00735	0	0
0	0	0	0	0	0	0	0
0	0	0	0	0	0	0	0
0	0	0	0	0	0	0.00133	0
0	0.0005	0.0135	0.1377	0.06723	0.02421	0	0.33061
0	0	0	0	0	0	0	0
0	0	0	0	0	0	0	0
0	0.00022	0	0	0.00362	0.00476	0	0.01342
0	0	0.00101	0	0	0	0	0
0	0	0.00216	0	0	0	0.00556	0.06401
0	0.00638	0.00529	0	0.01225	0.27738	0.15385	0.40845
0	0	0	0	0	0	0	0.00631
0	0	0	0	0	0	0	0
0	0.07791	0.06212	0	0.03732	0	2.98387	0.84208
0	0	0.00094	0.00042	0	0	0	0
0	0	0	0	0	0	0	0
0	0	0	0	0	0	0	0
0	0	0	0	0	0	0	0
0	0	0	0	0	0	0	0
0	0	0	0	0	0.00034	0	0.00499
0	0	0	0	0	0	0	0
0	0	0	0	0	0	0	0.00131
0	0	0	0	0	0	0	0
0	0	0	0	0	0	0	0
0	0	0	0	0	0	0	0
0	0	0	0	0	0	0	0.00355
0	0	0	0	0	0	0	0
12.2366	12.32203	50.72403	1.67223	5.37645	1.29796	27.11746	11.17106
12.2366	12.32203	50.72403	1.67223	5.37645	1.29796	27.11746	11.17106
0	0.00567	0.05147	0	0	0	0	0
0	0.00567	0.05147	0	0	0	0	0
0	0.00567	0.05147	0	0	0	0	0
0.00251	0.00075	0.03018	0	0	0	0	0

0.00251	0.00075	0.03018	0	0	0	0	0
0.00251	0.00075	0.03018	0	0	0	0	0
0.21002	3.19601	7.14766	0.022	0.51105	0.09447	0.17283	0.09
0	0	0	0	0	0	0	0
0	0	0	0	0	0	0	0
0.12316	0.49935	2.41448	0.022	0.50057	0.02534	0.13601	0.08397
0	0	0	0	0	0	0	0
0.05766	0.02724	0.67399	0.00247	0.50057	0.02534	0.05309	0.00037
0	0	0	0.01953	0	0	0	0.08359
0	0	0	0	0	0	0.00146	0
0	0	0	0	0	0	0.07889	0
0	0	0	0	0	0	0	0
0	0	0	0	0	0	0	0
0	0	0	0	0	0	0.00257	0
0	0	0.00023	0	0	0	0	0
0.0655	0.26482	0.06793	0	0	0	0	0
0	0	0	0	0	0	0	0
0	0.05604	1.62959	0	0	0	0	0
0	0.15125	0.04274	0	0	0	0	0
0	0	0	0	0	0	0	0
0	0	0	0	0	0	0	0
0.08686	2.69666	4.73318	0	0.01048	0.06913	0.03682	0.00603
0	0	0	0	0	0	0	0
0.08686	2.69666	4.73318	0	0.01048	0.06913	0.03682	0.00603
0	0	0	0	0	0	0	0
0	0	0	0	0	0	0	0
0	0.00097	0.01638	0	0.00147	0	0	0.00068
0	0	0.00495	0	0	0	0	0.00068
0	0	0.00018	0	0	0	0	0
0	0	0	0	0	0	0	0
0	0	0	0	0	0	0	0
0	0	0.00477	0	0	0	0	0.00068
0	0.00097	0.01142	0	0.00147	0	0	0
0	0.00097	0.01142	0	0.00147	0	0	0
0.23793	0.00544	0.09279	0	0	0	0.00617	0

0.04054	0	0.03143	0	0	0	0.00617	0
0.04054	0	0.03143	0	0	0	0.00617	0
0.185	0	0	0	0	0	0	0
0.185	0	0	0	0	0	0	0
0.01238	0.00544	0.06135	0	0	0	0	0
0.01238	0.00544	0.06135	0	0	0	0	0
0	0	0	0	0	0	0	0
0	0	0	0	0	0	0	0
0.00217	0.18713	0.75982	0	0	0	16.71792	0.70931
0	0	0.03293	0	0	0	0	0
0	0	0.03293	0	0	0	0	0
0	0	0	0	0	0	0	0
0	0	0	0	0	0	0	0
0.00217	0.18713	0.72689	0	0	0	16.71792	0.70931
0	0.00171	0.0041	0	0	0	0	0
0	0.03909	0.00726	0	0	0	0	0
0	0.06037	0.26667	0	0	0	0	0.40796
0	0	0	0	0	0	0.06868	0.00131
0.00217	0	0.00573	0	0	0	0	0
0	0	0	0	0	0	0	0
0	0	0	0	0	0	0	0
0	0	0	0	0	0	0	0
0	0	0	0	0	0	0.16873	0
0	0.08597	0.44312	0	0	0	16.48051	0.30004
0	0	0	0	0	0	0	0
0	0	0	0	0	0	0	0
0	0	0	0	0	0	0	0
0	0	0	0	0	0	0	0
3.92776	3.94281	28.62468	1.18002	2.91556	1.15963	8.47954	3.20554
0	0.03443	0.09251	0	0	0	0.29297	0.0561
0	0	0	0	0	0	0	0
0	0.03443	0.09251	0	0	0	0.29297	0.0561
0.03756	0.16978	0.03127	0	0	0	0	0
0.03756	0.16978	0.03127	0	0	0	0	0
0.1389	3.1906	20.36197	1.09041	1.34375	1.00693	1.62846	1.39028

0	0	0.0017	0	0	0	0	0
0	0.29296	5.90567	0	0	0.00019	0.01107	0
0.00148	0	0	0	0	0	0	0
0.01333	0	0.0008	0	0	0	0	0
0.01644	0.00126	0.00364	0	0	0	0	0
0	0.32098	0.4925	1.08981	0	0	0	0
0.0015	0.00092	0.00391	0	0	0	0.19964	0.00396
0.10614	2.57448	13.95374	0.0006	1.34375	1.00674	1.41775	1.38632
0	0	0	0	0	0	0	0
0	0	0	0	0	0	0	0
0	0	0	0	0	0	0	0
0.49819	0	0	0	0	0	0	0
0.49355	0	0	0	0	0	0	0
0.00464	0	0	0	0	0	0	0
0	0.07187	0.04118	0	0	0	0.55265	0
0	0.05333	0.03816	0	0	0	0.32399	0
0	0.01854	0.00302	0	0	0	0.22866	0
0	0	0	0	0	0	0	0
0.05745	0.01188	0.00114	0	0	0	0	0
0.00697	0.00284	0.00034	0	0	0	0	0
0.05048	0.00904	0.0008	0	0	0	0	0
0.00474	0	0	0	0	0	0	0
0.00474	0	0	0	0	0	0	0
0	0.0099	0.08742	0	0	0	0.95159	0.05689
0	0.0099	0.08742	0	0	0	0.95159	0.05689
0	0	0	0	0	0	0	0.00029
0	0	0	0	0	0	0	0.00029
3.16381	0.28895	6.52071	0.08961	1.35516	0.08186	0.15188	0.26737
0.00234	0.06641	0.15937	0	0	0	0.01537	0.00483
0.02373	0	0	0	0	0	0	0
1.97257	0.10914	4.31234	0.08961	1.35516	0.08186	0.13651	0.2549
0.02933	0.02371	0.63182	0	0	0	0	0.00233
0	0	0	0	0	0	0	0
0	0	0	0	0	0	0	0
0.00421	0.00012	0.24297	0	0	0	0	0.00531

1.04247	0	0	0	0	0	0	0
0.00031	0.00012	0.00046	0	0	0	0	0
0.08884	0.08945	1.17375	0	0	0	0	0
0	0	0	0	0	0	0	0
0	0	0	0	0	0	0	0
0	0	0	0	0	0	0	0
0	0.01873	0.02598	0	0	0	0.25482	0
0	0.01685	0.02598	0	0	0	0.25482	0
0	0	0	0	0	0	0	0
0	0.00189	0	0	0	0	0	0
0	0	0	0	0	0	0	0
0	0	0	0	0	0	0	0
0	0	0	0	0	0	0	0
0	0	0	0	0	0	0	0
0	0	0	0	0	0	0	0
0	0	0.00181	0	0	0	0	0.00011
0	0	0	0	0	0	0	0
0	0	0.00181	0	0	0	0	0.00011
0	0	0	0	0.00233	0.01412	0	0
0	0	0	0	0.00233	0.01412	0	0
0	0.00358	0.01326	0	0	0	4.45118	0.97227
0	0	0	0	0	0	0.00807	0
0	0	0	0	0	0	0.00198	0
0	0	0	0	0	0	4.42569	0
0	0.00358	0.01326	0	0	0	0.01545	0.97227
0	0	0	0	0	0	0	0
0	0	0	0	0	0	0	0
0.02711	0	1.41542	0	0	0	0	0
0.02711	0	1.41542	0	0	0	0	0
0	0	0	0	0	0	0	0
0	0	0	0	0	0	0	0
0	0	0	0	0	0	0	0
0	0	0	0	0	0	0	0
0	0.14309	0.03199	0	0.21432	0.05672	0.19599	0.46222
0	0.14309	0.03199	0	0.21432	0.05672	0.19599	0.46222

0.53192	0.19054	3.3714	0	0	0	0	0
0.53192	0.19054	3.3714	0	0	0	0	0
0.53192	0	0	0	0	0	0	0
0	0.19054	3.3714	0	0	0	0	0
0.15723	0	0.00577	0	0.00195	0	0.00754	0.03892
0.15723	0	0	0	0	0	0	0
0.15723	0	0	0	0	0	0	0
0	0	0	0	0	0	0	0
0	0	0	0	0	0	0	0
0	0	0.00523	0	0	0	0.00754	0
0	0	0.00523	0	0	0	0.00754	0
0	0	0	0	0	0	0	0
0	0	0	0	0	0	0	0
0	0	0	0	0	0	0	0
0	0	0	0	0	0	0	0
0	0	0.00054	0	0.00195	0	0	0.03892
0	0	0	0	0	0	0	0
0	0	0.00054	0	0.00195	0	0	0.03892
0	0	0	0	0	0	0	0
0	0	0	0	0	0	0	0
0	0	0	0	0	0	0	0
0	0	0	0	0	0	0	0
7.16707	4.79271	10.62389	0.47021	1.94643	0.04387	1.73347	7.12661
0	0.06936	0	0	0	0	0.08502	0.0175
0	0.06936	0	0	0	0	0.08502	0.0175
0.00256	0	0	0	0	0	0	0
0.00256	0	0	0	0	0	0	0
0.01237	0	0.17731	0	0	0	0	0
0.01237	0	0.17731	0	0	0	0	0
0.07477	0.67495	0.06769	0	0	0	0	0.00193
0.07477	0.67495	0.06769	0	0	0	0	0.00193
4.25782	0.01435	6.48812	0.0023	1.92974	0.00518	1.43192	6.70083
4.25782	0.01435	6.48812	0.0023	1.92974	0.00518	1.43192	6.70083
1.50408	2.82986	2.94702	0	0.00765	0.02615	0.19682	0.40139
1.50408	2.82986	2.94702	0	0.00765	0.02615	0.19682	0.40139

0	0	0	0	0	0	0	0
0	0	0	0	0	0	0	0
0	0	0	0	0	0	0	0
0.04201	0.00035	0.04236	0	0	0	0	0
0.04201	0.00035	0.04236	0	0	0	0	0
0	0	0	0	0	0	0	0
0	0	0	0	0	0	0	0
0	0	0	0	0	0	0	0
0.15448	0.02634	0.13786	0	0	0	0	0
0.00771	0.02634	0.13786	0	0	0	0	0
0.0045	0	0	0	0	0	0	0
0	0	0	0	0	0	0	0
0.14228	0	0	0	0	0	0	0
0	0	0	0	0	0	0	0
0.00202	0	0.00007	0	0	0	0	0
0	0	0	0	0	0	0	0
0.00202	0	0	0	0	0	0	0
0	0	0	0	0	0	0	0
0	0	0	0	0	0	0	0
0	0	0.00007	0	0	0	0	0
1.11696	1.1775	0.76346	0.46791	0.00903	0.01253	0.0197	0.00497
1.11696	1.1775	0.76346	0.46791	0.00903	0.01253	0.0197	0.00497
0.12576	0.33143	2.81461	0.78622	2.00349	0.10708	0.24417	0.20052
0.12576	0.33143	2.81461	0.78622	2.00349	0.10708	0.24417	0.20052
0.12576	0.33143	2.81461	0.78622	2.00349	0.10708	0.24417	0.20052
0	0.03932	0.0117	0	0	0	0	0
0	0.03932	0.0117	0	0	0	0	0
0	0	0	0	0	0	0	0
0	0	0	0	0	0	0	0
0.05115	0.00781	0.0103	0.11663	0	0	0	0
0.05115	0.00781	0.0103	0.11663	0	0	0	0
0	0.11101	0.04967	0	0.00669	0.00867	0	0
0	0.11101	0.04967	0	0.00669	0.00867	0	0
0.03253	0.04343	0.00934	0	0	0	0	0.00118
0.03253	0.04343	0.00934	0	0	0	0	0.00118

0	0	0	0	0	0	0	0
0	0	0	0	0	0	0	0
0.01969	0.12866	2.73331	0.6696	1.99446	0.09841	0.24417	0.19619
0	0.11049	0.19057	0.05682	1.95481	0.08893	0.0404	0.04341
0	0.00543	1.93628	0	0	0	0	0.00318
0.01969	0.01274	0.60646	0.61278	0.03966	0.00948	0.20377	0.14961
0	0	0	0	0	0	0	0
0	0	0	0	0	0	0	0
0	0	0	0	0	0	0	0
0	0	0	0	0	0	0	0
0	0	0	0	0	0	0	0
0	0	0	0	0	0	0	0
0	0	0	0	0	0	0	0
0	0	0	0	0	0	0	0
0.02239	0	0	0	0	0	0	0.00315
0.02239	0	0	0	0	0	0	0.00315
0	0	0	0	0	0	0	0
0	0	0	0	0	0	0	0
0	0.00121	0.00028	0	0.00233	0	0	0
0	0.00121	0.00028	0	0.00233	0	0	0
0	0	0	0	0	0	0	0
0	0	0	0	0	0	0	0
0.33607	0	0	0	0	0	0	0
0.33607	0	0	0	0	0	0	0
0.33607	0	0	0	0	0	0	0
0.33607	0	0	0	0	0	0	0
0	0	0	0	0	0	0	0
0	0	0	0	0	0	0	0
0	0	0	0	0	0	0	0
0	0	0	0	0	0	0	0
0	0	0	0	0	0	0	0
0.33607	0	0	0	0	0	0	0
0	0	0	0	0	0	0	0
0	0	0	0	0	0	0	0
1.39275	2.56202	3.55416	0.40693	0.81306	0.20232	1.93683	6.06488

0.65064	1.59253	0.7666	0.40693	0.81306	0.20232	0.8504	1.91238
0.65064	1.59253	0.7666	0.40693	0.81306	0.20232	0.8504	1.91238
0	0.11015	0	0	0	0	0	1.64218
0	0.11015	0	0	0	0	0	1.64218
0	0	0	0	0	0	0	0
0.65064	1.48238	0.7666	0.40693	0.81306	0.20232	0.8504	0.2702
0.65064	1.48238	0.7666	0.40693	0.81306	0.20232	0.8504	0.2702
0	0	0	0	0	0	0	0
0.72546	0.96343	2.78412	0	0	0	0	1.54791
0.72546	0.96343	2.78412	0	0	0	0	1.54791
0.72546	0.96343	2.78412	0	0	0	0	1.54791
0	0.62198	2.23976	0	0	0	0	1.03012
0.72546	0.34145	0.54435	0	0	0	0	0.51779
0	0	0	0	0	0	0	0
0	0	0	0	0	0	0	0
0	0	0	0	0	0	0	0
0	0	0	0	0	0	0	0
0	0	0	0	0	0	0	0
0	0	0	0	0	0	0	0
0	0	0	0	0	0	0	0
0.01665	0.00606	0.00344	0	0	0	1.08643	2.6046
0.01665	0.00606	0.00344	0	0	0	1.08643	2.6046
0	0	0	0	0	0	0	0
0	0	0	0	0	0	0	0
0	0	0	0	0	0	0	0
0	0	0	0	0	0	0	0
0	0	0	0	0	0	0	0.27114
0	0	0	0	0	0	0	0.00123
0	0	0	0	0	0	0	0.07355
0	0	0	0	0	0	0	0.19635
0.0115	0.00413	0.00139	0	0	0	0.00023	1.16072
0.0115	0	0	0	0	0	0	0.09323
0	0	0	0	0	0	0	0
0	0.00413	0.00139	0	0	0	0.00023	1.06749
0	0	0	0	0	0	0	0

0	0	0	0	0	0	0	0
0.00515	0.00193	0.00205	0	0	0	1.0862	1.17274
0.00515	0.00193	0	0	0	0	0.3315	0.76728
0	0	0.00205	0	0	0	0.36408	0.09677
0	0	0	0	0	0	0.08194	0.04772
0	0	0	0	0	0	0.16852	0.16297
0	0	0	0	0	0	0	0
0	0	0	0	0	0	0.00042	0.00109
0	0	0	0	0	0	0.13974	0.09574
0	0	0	0	0	0	0	0.00118
0	0.00997	0.00242	0	0.00998	0.00054	0	0
0	0.00997	0.00242	0	0.00998	0.00054	0	0
0	0.00997	0.00242	0	0.00998	0.00054	0	0
0	0.00114	0	0	0	0	0	0
0	0	0	0	0	0	0	0
0	0.00114	0	0	0	0	0	0
0	0	0	0	0	0	0	0
0	0	0	0	0	0	0	0
0	0	0.00242	0	0.00998	0.00054	0	0
0	0	0.00242	0	0.00677	0.00054	0	0
0	0	0	0	0.00321	0	0	0
0	0.00883	0	0	0	0	0	0
0	0	0	0	0	0	0	0
0	0.00883	0	0	0	0	0	0
0	0	0	0	0	0	0	0
0	3.6388	0.8075	2.76449	0	0.00054	0.42923	0.29835
0	3.6388	0.8075	2.76449	0	0.00054	0.42923	0.29835
0	3.6388	0.8075	2.76449	0	0.00054	0.42923	0.29835
0	3.6388	0.8075	2.76449	0	0.00054	0.42923	0.28777
0	0	0	0	0	0	0	0
0	0	0	0	0	0	0	0
0	3.6388	0.8075	2.76449	0	0.00054	0.42923	0.28777
0	0	0	0	0	0	0	0
0	3.6388	0.8075	2.76449	0	0	0	0
0	0	0	0	0	0	0	0

0	0	0	0	0	0	0	0
0	0	0	0	0	0	0	0.02196
0	0	0	0	0	0	0	0
0	0	0	0	0	0	0	0
0	0	0	0	0	0.00054	0.42923	0.2658
0	0	0	0	0	0	0	0.01058
0	0	0	0	0	0	0	0.01058
0	0	0	0	0	0	0	0
0	0	0	0	0	0	0	0
0	0	0	0	0	0	0	0.01058
0	0	0	0	0	0	0	0
0	0	0	0	0	0	0	0
0	0	0	0	0	0	0	0
0	0	0	0	0	0	0	0
0	0	0	0	0	0	0	0
0	0	0	0	0	0	0	0
0	0	0	0	0	0	0	0
0	0	0	0	0	0	0	0
0.33435	1.22598	1.22998	4.19064	18.74522	14.14043	16.79888	1.03374
0.04634	0	0.15515	0	0	0	0.02632	0.0427
0.04634	0	0.15251	0	0	0	0.02632	0.0148
0	0	0	0	0	0	0	0
0	0	0	0	0	0	0	0
0	0	0	0	0	0	0	0
0	0	0	0	0	0	0	0
0	0	0	0	0	0	0	0
0	0	0	0	0	0	0	0
0.04634	0	0.15251	0	0	0	0	0
0.04634	0	0.15251	0	0	0	0	0
0.04634	0	0.15251	0	0	0	0	0
0	0	0	0	0	0	0.02632	0.0148
0	0	0	0	0	0	0	0
0	0	0	0	0	0	0	0
0	0	0	0	0	0	0.02632	0.0148
0	0	0	0	0	0	0.02632	0.0148

0	0	0	0	0	0	0	0
0	0	0	0	0	0	0	0
0	0	0.00264	0	0	0	0	0.02789
0	0	0.00264	0	0	0	0	0.02789
0	0	0	0	0	0	0	0.00929
0	0	0	0	0	0	0	0.00929
0	0	0.00264	0	0	0	0	0.0186
0	0	0	0	0	0	0	0
0	0	0.00264	0	0	0	0	0.01481
0	0	0	0	0	0	0	0
0	0	0	0	0	0	0	0
0	0	0	0	0	0	0	0.00379
0.08213	0.92616	0.88555	0	0.02655	0.0008	0.00629	0.02512
0.08213	0.92616	0.88555	0	0.02655	0.0008	0.00629	0.02512
0.08213	0.92616	0.88555	0	0.02655	0.0008	0.00629	0.02512
0.08213	0.16239	0.06978	0	0.02655	0.0008	0.00629	0.02512
0.08213	0.16239	0.06978	0	0.02655	0.0008	0.00629	0.02512
0	0.76377	0.81577	0	0	0	0	0
0	0	0	0	0	0	0	0
0	0.76377	0.81577	0	0	0	0	0
0	0	0	0	0	0	0.00169	0.05252
0	0	0	0	0	0	0.00169	0.05252
0	0	0	0	0	0	0.00169	0.05252
0	0	0	0	0	0	0.00169	0.05252
0	0	0	0	0	0	0.00169	0.0524
0	0	0	0	0	0	0	0
0	0	0	0	0	0	0	0
0	0	0	0	0	0	0	0.00012
0	0	0	0	0	0	0	0
0	0	0	0	0	0	0	0
0	0	0	0	0	0	0	0
0	0	0	0	0	0	0	0
0	0	0	0	0	0	0	0
0.20588	0.29983	0.18928	4.19064	18.71866	14.13964	16.76458	0.91341
0	0	0	0	0	0	0	0

0	0	0	0	0	0	0	0
0	0	0	0	0	0	0	0
0	0	0	0	0	0	0	0
0	0	0	0	0	0	0	0
0	0	0	0	0	0	0	0
0	0	0	0	0	0	0	0
0	0	0	0	0	0	0	0
0	0	0	0	0	0	0	0
0	0	0	0	0	0	0	0
0	0	0	0	0	0	0	0
0	0	0	0	0	0	0	0
0	0	0	0	0	0	0	0
0	0	0	0	0	0	0	0
0.20588	0.29983	0.18928	4.19064	18.71866	14.13964	16.4268	0.6182
0	0	0	0	0	0	0	0
0	0	0	0	0	0	0	0
0	0	0	0	0	0	0	0
0.20588	0.29983	0.18928	4.19064	18.71866	14.13964	16.4268	0.6182
0	0	0	0	0	0	0.33385	0
0	0	0	0	0	0	0	0
0	0	0	0	0	0	0	0
0	0	0	0	0	0	0.00425	0
0	0	0	0	0	0	0	0
0	0	0	0	0	0	0	0
0	0	0	0	0	0	0	0
0	0	0	0	0	0	0.18944	0
0	0	0	0	0	0	0	0
0	0	0	0	0	0	0	0
0	0	0	0	0	0	0.11964	0
0	0	0	0	0	0	0.02053	0
0	0	0	0	0	0	0	0
0	0	0	0	0	0	0	0
0	0	0	0	0	0	0.2704	0
0	0	0	0	0	0	0.26885	0
0	0	0	0	0	0	0	0

0	0	0	0	0	0	0.00155	0
0.20588	0.29983	0.18928	4.13562	18.71533	14.13885	0.0146	0.6182
0.20588	0.29983	0.18928	4.13562	18.71533	14.13885	0.0146	0.6182
0	0	0	0	0	0	0	0
0	0	0	0.05502	0.00333	0.00079	15.80795	0
0	0	0	0	0	0	0	0
0	0	0	0	0	0	0	0
0	0	0	0	0	0	0	0
0	0	0	0.04271	0.00227	0.00079	11.67335	0
0	0	0	0	0	0	0.30252	0
0	0	0	0.01231	0.00106	0	3.83208	0
0	0	0	0	0	0	0	0
0	0	0	0	0	0	0	0
0	0	0	0	0	0	0	0
0	0	0	0	0	0	0	0
0	0	0	0	0	0	0	0
0	0	0	0	0	0	0	0
0	0	0	0	0	0	0	0
0	0	0	0	0	0	0	0
0	0	0	0	0	0	0	0
0	0	0	0	0	0	0	0
0	0	0	0	0	0	0	0
0	0	0	0	0	0	0	0
0	0	0	0	0	0	0	0
0	0	0	0	0	0	0	0
0	0	0	0	0	0	0	0
0	0	0	0	0	0	0	0
0	0	0	0	0	0	0.33778	0.29521
0	0	0	0	0	0	0.33778	0.29521
0	0	0	0	0	0	0	0
0	0	0	0	0	0	0	0
0	0	0	0	0	0	0	0
0	0	0	0	0	0	0.33778	0.29521
0	0	0	0	0	0	0	0
0	0	0	0	0	0	0	0

0	0	0	0	0	0	0.3323	0.29521
0	0	0	0	0	0	0.00166	0
0	0	0	0	0	0	0	0
0	0	0	0	0	0	0	0
0	0	0	0	0	0	0.00382	0
0	0	0	0	0	0	0	0
0	0	0	0	0	0	0	0
0	0	0	0	0	0	0	0
0	0	0	0	0	0	0	0
0	0	0	0	0	0	0	0
0	0	0	0	0	0	0	0
0	0	0	0	0	0	0	0
0	0	0	0	0	0	0	0
0	0	0	0	0	0	0	0
0	0	0	0	0	0	0	0
0	0	0	0	0	0	0	0
0	0	0	0	0	0	0	0
0.01103	0.08423	0.06374	0	0	0	0	0
0.01103	0.08423	0.06374	0	0	0	0	0
0.01103	0.08423	0.06374	0	0	0	0	0
0.01103	0.08423	0.06374	0	0	0	0	0
0.01103	0.08423	0.06374	0	0	0	0	0
0	0.02285	0.01527	0	0	0	0	0
0.01103	0.06138	0.04847	0	0	0	0	0
0	0	0	0	0	0	0	0
0	0	0	0	0	0	0	0
0	0	0	0	0	0	0	0
0	0	0	0	0	0	0	0
0	0	0	0	0	0	0	0
0	0	0	0	0	0	0	0
0	0	0	0	0	0	0	0
0	0	0	0	0	0	0	0
0	0	0	0	0	0	0	0
0	0	0	0	0	0	0	0
0	0	0	0	0	0	0	0

0	0	0	0	0	0	0	0
0	0	0	0	0	0	0	0
0	0	0	0	0	0	0	0
0	0	0	0	0	0	0	0
0	0	0	0	0	0	0	0
0	0	0	0	0	0	0	0
0	0	0	0	0	0	0	0
0	0	0	0	0	0	0	0
0	0	0	0	0	0	0	0
0	0	0	0	0	0	0	0
0	0	0	0	0	0	0	0
0	0	0	0	0	0	0	0
0	0	0	0	0	0	0	0
0	0	0	0	0	0	0	0
0	0	0	0	0	0	0	0
0	0	0	0	0	0	0	0
0	0	0	0.00207	0	0.0069	0	0
0	0	0	0.00207	0	0.0069	0	0
0	0	0	0.00207	0	0.0069	0	0
0	0	0	0.00207	0	0.0069	0	0
0	0	0	0	0	0	0	0
0	0	0	0	0	0	0	0
0	0	0	0	0	0	0	0
0	0	0	0.00207	0	0.0069	0	0
0	0	0	0.00207	0	0.0069	0	0
0	0	0	0.00207	0	0.0069	0	0

AP017_2	AP017_3	AP018_1	AP018_2	AP018_3	AP019_1	AP019_2	AP019_3
100	100	97.67721	98.08602	98.76995	99.99948	100	99.99937
1.77453	55.03769	0.18097	0.03838	0.30557	0.44863	0.06626	0.41664
1.27365	54.5062	0.01084	0.02089	0.01345	0.02127	0.01232	0.01823
0.0003	0.00183	0.002	0.01436	0.00463	0.02041	0.00782	0.01765
0.0003	0.00183	0.002	0.01436	0.00463	0.02041	0.00782	0.01765
0	0	0	0	0	0	0	0
0	0	0	0	0	0	0	0
0.0003	0.00183	0.002	0.01436	0.00463	0.02041	0.00782	0.01765
0	0	0	0	0	0	0	0
0	0.00096	0	0	0	0.00696	0	0.00447
0	0	0	0	0	0	0	0
0	0	0	0	0	0	0	0
0	0	0	0	0	0	0	0
0	0	0	0	0	0	0	0
0	0.00042	0.00149	0.00344	0.00087	0.00292	0.00035	0.00227
0	0	0	0	0	0	0	0
0.0003	0.00044	0	0	0	0.0029	0	0.00231
0	0	0	0	0	0.00063	0	0.00026
0	0	0.00044	0.00106	0.00087	0.00534	0	0.00615
0	0	0	0.00152	0.00036	0.00017	0.00553	0.00111
0	0	0	0	0	0	0	0
0	0	0.00006	0.00835	0.00057	0.0015	0.00195	0.00106
0	0	0	0	0	0	0	0
0	0	0	0	0.00196	0	0	0
0	0	0	0	0	0	0	0
0	0	0	0	0	0	0	0
1.27335	54.48584	0	0.00405	0	0.00031	0.00212	0.00028
1.27335	54.48584	0	0.00405	0	0.00031	0.00212	0.00028
0	0.00048	0	0	0	0	0	0
0	0.00048	0	0	0	0	0	0
0	0	0	0	0	0	0.00212	0
0	0	0	0	0	0	0.00212	0
1.26497	54.21615	0	0.00405	0	0.00031	0	0.00028

0.86818	24.92678	0	0	0	0	0	0
0	0	0	0	0	0	0	0
0.12818	0.29853	0	0	0	0	0	0
0.00639	2.77231	0	0	0	0	0	0
0	0	0	0.00357	0	0.00031	0	0.00028
0.1678	16.28041	0	0.00048	0	0	0	0
0.09442	9.93812	0	0	0	0	0	0
0.00839	0.26921	0	0	0	0	0	0
0.00839	0.26921	0	0	0	0	0	0
0	0	0	0	0	0	0	0
0	0	0	0	0	0	0	0
0	0	0	0	0	0	0	0
0	0	0	0	0	0	0	0
0	0	0	0	0	0	0	0
0	0	0	0	0	0	0	0
0	0	0	0	0	0	0	0
0	0	0	0	0	0	0	0
0	0.01854	0.00884	0.00248	0.00882	0.00054	0.00238	0.00031
0	0.01854	0.00884	0.00248	0.00882	0.00054	0.00238	0.00031
0	0.01854	0.00884	0.00248	0.00882	0.00054	0.00238	0.00031
0	0	0	0	0	0	0	0
0	0	0	0	0	0	0	0
0	0.01854	0.00884	0.00248	0.00882	0.00054	0.00238	0.00031
0	0	0	0	0	0	0	0
0	0	0	0	0	0	0	0
0	0	0	0	0	0	0	0
0	0	0	0	0	0	0	0
0	0	0	0	0	0	0	0
0	0	0	0	0	0	0	0
0.50088	0.53149	0.17013	0.01749	0.29212	0.42736	0.05394	0.39841
0.10476	0.52379	0	0	0	0.35894	0.01208	0.33642
0.00213	0	0	0	0	0	0.01208	0.00012
0.00213	0	0	0	0	0	0.01208	0.00012
0	0	0	0	0	0	0	0
0.00213	0	0	0	0	0	0.00835	0.00012

0	0	0	0	0	0	0.00373	0
0	0	0	0	0	0	0	0
0	0	0	0	0	0	0	0
0	0	0	0	0	0	0	0
0.10263	0.52379	0	0	0	0.35894	0	0.3363
0.10263	0.52369	0	0	0	0.35855	0	0.33581
0.10163	0.52141	0	0	0	0.28038	0	0.27388
0.0008	0.00069	0	0	0	0.02194	0	0.01709
0.0002	0.00159	0	0	0	0.05623	0	0.04484
0	0.0001	0	0	0	0.00039	0	0.00049
0	0.0001	0	0	0	0.00039	0	0.00049
0	0	0	0	0	0	0	0
0.39612	0.0077	0.17013	0.01749	0.29212	0.06842	0.04186	0.06199
0.39612	0.0077	0.17013	0.01749	0.29212	0.06842	0.04186	0.06199
0	0	0.09175	0.00495	0.15493	0	0	0
0	0	0.09175	0.00495	0.15493	0	0	0
0	0	0.02379	0.00133	0.03761	0	0	0
0	0	0.02379	0.00133	0.03761	0	0	0
0.36853	0.0074	0.02883	0.00215	0.05184	0.06095	0.03899	0.05505
0.36853	0.0074	0.02883	0.00215	0.05184	0.06095	0.03899	0.05505
0	0	0.00043	0	0.00086	0	0	0
0	0	0.00043	0	0.00086	0	0	0
0.02759	0.0003	0.02533	0.00906	0.04689	0.00747	0.00288	0.00694
0.02759	0.0003	0.02533	0.00906	0.04689	0.00747	0.00288	0.00694
0	0	0	0	0	0	0	0
0	0	0	0	0	0	0	0
67.01439	28.54564	52.29163	56.93752	51.65554	62.42236	72.33596	57.70653
0	0	0	0	0	0	0	0
0	0	0	0	0	0	0	0
0	0	0	0	0	0	0	0
0	0	0	0	0	0	0	0
0	0	0	0	0	0	0	0
67.01439	28.54564	52.29163	56.93752	51.65554	62.42236	72.33596	57.70653
67.01439	28.54564	52.29163	56.93752	51.65554	62.42236	72.33596	57.70653
58.92929	25.56556	32.66067	50.25171	31.92384	45.32109	43.77595	43.05896

58.92929	25.56556	32.66067	50.25171	31.92384	45.32109	43.77595	43.05896
0.92794	0.00011	1.36356	0.04935	1.32233	0	0.00078	0
0	0	0	0	0	0.3091	0	0.28358
0.48021	0.42906	0	0	0	0	0	0
0.00201	0.0014	4.54279	16.5326	5.8741	7.86596	0.07932	10.10011
0	0	0.68663	0.04446	0.66536	2.52412	0.11623	1.89044
0	0	0	0	0	0	0	0
0	0	0	0	0	0	0.00073	0
0	0	0.06837	0.00097	0.06542	0	0	0
28.57338	21.26734	0	0.00368	0.00263	3.96163	0.01737	7.40347
0.00988	0	1.82345	0.74576	1.9164	0	0	0
0	0	0	0	0	0	0	0
0	0	0.00172	0	0.00315	0.0353	0.10661	0.01427
2.45003	0.06785	0.75826	0.54937	0.81626	3.35716	0.10295	2.67791
0.00049	0.0018	12.44255	19.7579	8.57135	0	0	0
0	0	0.01015	0	0.01143	0.55874	4.40655	0.31275
0	0	0	0	0	0	0	0
0	0	0	0	0	0.04833	0.26548	0.0361
1.69567	0.13602	2.75359	3.1366	2.8184	0.00207	0.00241	0
12.75195	0.01151	0.08396	2.19866	0.09194	5.23415	29.13007	2.92084
0.4622	0.03065	8.11286	7.22751	9.73552	4.94017	0.21707	4.99529
11.57087	3.61982	0.00433	0.004	0.01912	15.69456	6.48744	11.91388
0.00467	0	0.00843	0.00083	0.01044	0.78979	2.84295	0.51033
0	0	2.24695	1.28379	2.56051	0	0	0
0	0	2.24695	1.28379	2.56051	0	0	0
0	0	2.24695	1.28379	2.56051	0	0	0
0	0	0	0	0	0	0	0
0	0	0	0	0	0	0	0
0	0	0	0	0	0	0	0
0.57188	0	0.80693	5.12085	0.70597	0.67325	0.01177	0.46029
0.57188	0	0.46172	2.31014	0.43259	0.57069	0.01177	0.39129
0.15301	0	0.00028	0	0.00054	0.48814	0.01024	0.33172
0.41887	0	0.46144	2.31014	0.43205	0.08255	0.00153	0.05957
0	0	0.3452	2.81071	0.27338	0.10256	0	0.069
0	0	0	0	0	0	0	0

0	0	0.3452	2.81071	0.27338	0.10256	0	0.069
0	0	0.00106	0	0.03208	0	0.0014	0
0	0	0	0	0.02841	0	0.0014	0
0	0	0	0	0	0	0	0
0	0	0	0	0	0	0.0014	0
0	0	0	0	0.02841	0	0	0
0	0	0.00106	0	0.00367	0	0	0
0	0	0.00106	0	0.00367	0	0	0
0.00082	0.38263	0.03628	0.01848	0.13411	0.01284	0.00057	0.00021
0	0	0	0	0	0	0	0
0	0	0	0	0	0	0	0
0	0	0	0	0	0	0	0
0	0	0	0	0	0	0	0
0	0	0	0	0	0	0	0
0.00082	0.38263	0.03628	0.01848	0.13411	0.01284	0.00057	0.00021
0	0.36928	0.00409	0.00572	0.0053	0.01248	0	0.00009
0	0	0	0	0	0	0	0
0	0	0.00018	0	0.03731	0	0	0
0	0	0.01666	0	0.04658	0	0	0
0	0	0	0	0	0	0	0.00011
0	0	0	0	0	0	0	0
0	0.01029	0.01213	0.01276	0.03442	0	0	0
0	0	0	0	0	0	0	0
0	0	0	0	0	0	0	0
0	0	0	0	0	0	0	0
0	0	0	0	0	0	0	0
0	0	0	0	0	0	0	0
0	0	0	0	0	0	0	0
0	0	0	0	0	0	0	0
0	0	0	0	0	0	0	0
0	0	0	0	0.00061	0.00037	0.00057	0
0	0	0.00323	0	0.00989	0	0	0
0.00082	0.00306	0	0	0	0	0	0
4.61734	0.00261	14.27018	0.12822	14.29291	5.5609	0.70565	4.52493
4.61734	0.00261	14.27018	0.12822	14.29291	5.5609	0.70565	4.52493

0.03442	0	1.08679	0.01305	1.43	0	0	0
0.10654	0	0.04182	0	0.04594	0.01941	0.01159	0.01405
0.06431	0	0	0	0	0	0	0
4.07106	0.00261	12.54576	0.11431	12.33241	5.33067	0.04979	4.39951
0.341	0	0.59581	0.00086	0.48455	0.21083	0.64427	0.11136
0	0	0	0	0	0	0	0
2.89506	2.59483	2.26957	0.13448	2.00613	10.85427	27.84062	9.66215
2.89506	2.59483	2.26957	0.13448	2.00613	10.85427	27.84062	9.66215
1.60739	2.58661	0.43427	0.03561	0.42275	5.46757	13.16739	4.31745
1.28767	0.00822	0.09105	0	0.08107	2.2875	5.07703	3.51593
0	0	0	0	0	0.07321	4.96592	0.07068
0	0	0	0	0	0	0	0
0	0	1.74425	0.09887	1.50232	3.02599	4.63028	1.75808
0	0	0	0	0	0	0	0
0	0	0	0	0	0	0	0
0	0	0	0	0	0	0	0
0	0	0	0	0	0	0	0
0	0	0	0	0	0	0	0
0	0	0	0	0	0	0	0
0	0	0	0	0	0	0	0
29.39724	15.19266	35.64737	27.48343	38.73686	21.6998	17.90761	24.8831
0.14876	3.1723	0.17498	1.03061	0.18063	0.16414	1.16481	0.13736
0	0.02491	0	0.10655	0.00134	0	0.00239	0
0	0.02491	0	0.10655	0.00134	0	0.00239	0
0	0.02491	0	0.10655	0.00134	0	0.00239	0
0	0	0	0	0.00013	0	0	0
0	0.00535	0	0.10513	0	0	0	0
0	0.00033	0	0	0	0	0	0
0	0.01922	0	0.00143	0.00121	0	0.00239	0
0.14876	3.14739	0.17498	0.92406	0.17929	0.16414	1.16242	0.13736
0	0	0	0	0	0	0.00614	0
0	0	0	0	0	0	0.00614	0
0	0	0	0	0	0	0.00301	0
0	0	0	0	0	0	0.00314	0
0.00067	0.00032	0	0	0	0	0.00019	0

0.00067	0.00032	0	0	0	0	0.00019	0
0.00067	0	0	0	0	0	0	0
0	0.00032	0	0	0	0	0.00019	0
0.00261	0	0.00155	0	0.00819	0.01588	1.134	0.01313
0.00261	0	0.00155	0	0.00819	0.01588	1.134	0.01313
0	0	0	0	0	0	0	0
0	0	0	0	0	0	0.51367	0
0	0	0	0	0	0	0	0
0.00261	0	0.00155	0	0.00819	0.01195	0.12872	0.00319
0	0	0	0	0	0	0	0
0	0	0	0	0	0.00394	0.2136	0.00994
0	0	0	0	0	0	0	0
0	0	0	0	0	0	0	0
0	0	0	0	0	0	0.27801	0
0	0	0	0	0	0	0	0
0	0	0.00734	0	0.0093	0	0	0
0	0	0.00734	0	0.0093	0	0	0
0	0	0	0	0	0	0	0
0	0	0	0	0	0	0	0
0	0	0	0	0	0	0	0
0	0	0	0	0	0	0	0
0	0	0	0	0	0	0	0
0	0	0	0	0	0	0	0
0	0	0	0	0	0	0	0
0	0	0.00734	0	0.0093	0	0	0
0	0	0	0	0	0	0	0
0	0	0	0	0	0	0	0
0	0	0	0	0	0	0	0
0	0	0	0	0	0	0	0
0	0	0	0	0	0	0	0
0	0	0	0	0	0	0	0
0	0	0	0	0	0	0	0
0	0	0	0	0	0	0	0
0	0	0	0	0	0	0	0
0.14547	3.14707	0.16609	0.92406	0.16181	0.14826	0.02208	0.12423

0.14547	3.14707	0.16609	0.92406	0.16181	0.14826	0.02208	0.12423
0.055	0.00095	0	0	0	0	0	0
0	0.00413	0	0	0	0	0	0
0	0.00157	0	0	0	0	0	0
0	0	0	0	0	0	0	0
0	0.0008	0.0019	0	0.00244	0	0	0
0.03016	0.00707	0.00347	0.18425	0.00076	0.00377	0.02142	0.00112
0	0	0	0	0	0	0	0
0	0	0	0	0	0	0	0
0.00298	0.05488	0	0.20277	0	0.00217	0	0.00092
0	0	0	0	0	0	0	0
0.00968	0.00484	0.01395	0	0.00987	0.00143	0	0
0.00665	0.09039	0.04748	0.48869	0.05125	0.0047	0.00066	0.00182
0.0129	0	0	0	0	0.12702	0	0.11395
0	0.00275	0	0	0	0	0	0
0.02811	2.95711	0.0993	0.04683	0.09587	0.00917	0	0.00642
0	0.00393	0	0	0.00163	0	0	0
0	0	0	0	0	0	0	0
0	0.00208	0	0	0	0	0	0
0	0.00135	0	0	0	0	0	0
0	0	0	0	0	0	0	0
0	0	0	0	0	0	0	0
0	0	0	0	0	0	0	0
0	0	0	0	0	0	0	0
0	0	0	0	0	0	0	0
0	0	0	0	0	0	0	0
0	0.00953	0	0	0	0	0	0
0	0.0057	0	0.00152	0	0	0	0
0	0	0	0	0	0	0	0
25.85126	6.68775	33.89403	25.78302	36.76646	18.37657	15.03385	21.89953
25.85126	6.68775	33.89403	25.78302	36.76646	18.37657	15.03385	21.89953
0.01758	0	0.00622	0	0.00974	0.00208	0.00011	0.00306
0.01758	0	0.00622	0	0.00974	0.00208	0.00011	0.00306
0.01758	0	0.00622	0	0.00974	0.00208	0.00011	0.00306
0.00312	0	0.00238	0	0.00453	0	0	0

0.00312	0	0.00238	0	0.00453	0	0	0
0.00312	0	0.00238	0	0.00453	0	0	0
0.12179	0.59859	0.67784	3.80357	0.9465	1.21871	4.07996	2.39759
0	0	0	0	0	0	0	0
0	0	0	0	0	0	0	0
0.02678	0.32589	0.66953	3.77738	0.93614	0.14677	2.01199	0.09047
0.00176	0	0	0	0	0	0	0
0.02502	0.32198	0	0.02155	0.00057	0.13004	2.01199	0.08553
0	0	0	0	0	0	0	0
0	0	0	0	0	0.01107	0	0.00022
0	0	0	0	0	0.00566	0	0.00471
0	0	0	0	0	0	0	0
0	0	0	0	0	0	0	0
0	0.00391	0	0	0	0	0	0
0	0	0	0	0	0	0	0
0	0	0.64372	3.73902	0.87184	0	0	0
0	0	0	0	0	0	0	0
0	0	0	0	0	0	0	0
0	0	0.0258	0.01682	0.06373	0	0	0
0	0	0	0	0	0	0	0
0	0	0	0	0	0	0	0
0.09501	0.2727	0.00831	0.02619	0.01036	1.06	2.06798	2.29033
0	0	0	0	0	0	0	0
0.09501	0.2727	0.00831	0.02619	0.01036	1.06	2.06798	2.29033
0	0	0	0	0	0.01194	0	0.01679
0	0	0	0	0	0.01194	0	0.01679
0	0.00073	0	0.00085	0	0.00007	0	0
0	0.00073	0	0.00085	0	0	0	0
0	0	0	0	0	0	0	0
0	0	0	0	0	0	0	0
0	0	0	0	0	0	0	0
0	0.00073	0	0.00085	0	0	0	0
0	0	0	0	0	0.00007	0	0
0	0	0	0	0	0.00007	0	0
0.00431	0	0.11975	0.3077	0.1516	0.09309	0	0.0959

0	0	0.00084	0.05808	0.00149	0.00456	0	0.00359
0	0	0.00084	0.05808	0.00149	0.00456	0	0.00359
0	0	0.00321	0.12801	0.00599	0.01094	0	0.01262
0	0	0.00321	0.12801	0.00599	0.01094	0	0.01262
0.00431	0	0.11571	0.12161	0.14411	0.07759	0	0.0797
0.00431	0	0.11571	0.12161	0.14411	0.07759	0	0.0797
0	0	0	0	0	0	0	0
0	0	0	0	0	0	0	0
0.10773	0.50927	8.75745	1.64303	7.32909	0.31782	0	0.28904
0	0	0	0	0	0	0	0
0	0	0	0	0	0	0	0
0	0	0	0.00172	0	0	0	0
0	0	0	0.00172	0	0	0	0
0.10773	0.50927	8.75745	1.64131	7.32909	0.31782	0	0.28904
0.00124	0	0	0	0	0.02044	0	0.026
0	0	0	0	0	0	0	0
0.03356	0	0.35956	0.00658	0.32712	0.05359	0	0.07908
0.00017	0.00168	0.35405	0.05779	0.39105	0	0	0
0.00147	0	0	0	0	0.00246	0	0.00255
0	0	0.1144	0.05762	0.12559	0	0	0
0	0	7.70976	1.51409	6.2931	0	0	0
0	0	0	0	0	0	0	0
0	0	0.07133	0.0022	0.09773	0	0	0
0.07129	0.50759	0	0	0	0.24133	0	0.18142
0	0	0.00008	0	0.00019	0	0	0
0	0	0.14827	0.00304	0.0943	0	0	0
0	0	0	0	0	0	0	0
0	0	0	0	0	0	0	0
12.43834	1.85615	13.94777	6.97376	18.26564	9.75728	10.13439	11.11761
0.01307	0.37752	0.28635	0.02458	0.17438	0	0.00308	0.00022
0	0	0	0	0	0	0.00308	0.00022
0.01307	0.37752	0.28635	0.02458	0.17438	0	0	0
0	0	0	0	0	0.33221	0.14195	0.60946
0	0	0	0	0	0.33221	0.14195	0.60946
8.41311	0.63368	1.63411	0.39902	2.04733	6.31898	2.42862	7.16993

0	0	0	0	0	0.00136	0	0.00237
0	0	0	0	0	0	0	0
0	0	0.01845	0.08037	0.01807	0.00569	0	0.01025
0	0	0.81283	0.10702	0.79386	0.36451	0	0.30832
0	0	0	0	0	0.00465	0	0.00331
0	0	0	0	0	0.02039	0	0.01835
0.00013	0.0188	0.15007	0.06744	0.14211	0.0708	0	0.06612
8.41298	0.61488	0.00793	0.01942	0.01082	5.85159	2.42862	6.76121
0	0	0.64483	0.12477	1.08247	0	0	0
0	0	0	0	0	0	0	0
0	0	0	0	0	0	0	0
0	0	0.67313	0.04968	0.88521	0	0	0
0	0	0.21871	0.03687	0.27983	0	0	0
0	0	0.45442	0.01281	0.60537	0	0	0
0	0	0.85682	0.16948	1.24754	0	0	0
0	0	0.25443	0.06339	0.39026	0	0	0
0	0	0.60239	0.10608	0.85728	0	0	0
0	0	0	0	0	0	0	0
0.00267	0.0089	0.04903	0.44667	0.05807	0.0308	0	0.04115
0.00267	0.0089	0	0.03187	0	0	0	0
0	0	0.04903	0.41481	0.05807	0.0308	0	0.04115
0	0	0	0	0	0	0.00115	0
0	0	0	0	0	0	0.00115	0
0.02072	0	0.38146	0.01649	0.25762	0	0	0
0.02072	0	0.38146	0.01649	0.25762	0	0	0
0	0	0	0	0	0	0	0
0	0	0	0	0	0	0	0
1.75899	0.64779	0.06703	2.66399	0.13266	1.02246	7.54942	0.83275
0.01583	0.00104	0.00045	0.04266	0.0013	0.04884	0.02062	0.03439
0	0	0.01501	0.09221	0.0297	0.06931	0	0.05673
1.41822	0.52847	0.00498	0.19183	0.01834	0.6309	4.7017	0.50701
0.09512	0.028	0.04545	2.29907	0.07894	0.13017	0.01402	0.12236
0	0	0	0	0	0	0	0
0	0	0	0	0	0	0	0.0004
0.003	0.00527	0.00025	0.02423	0.00209	0.07062	0	0.06202

0	0	0	0	0	0	0	0
0	0	0	0	0	0.00083	0	0.0009
0.22682	0.08501	0	0.01399	0	0.07177	2.81307	0.04895
0	0	0	0	0	0	0	0
0	0	0.00089	0	0.0023	0	0	0
0	0	5.96047	0.05456	8.30168	0.58936	0	0.80065
0	0	3.39098	0.17791	4.54289	0	0	0
0	0	3.39098	0.17791	4.54289	0	0	0
0	0	0	0	0	0	0	0
0	0	0	0	0	0	0	0
0	0	5.96047	0.05456	8.30168	0.58936	0	0.80065
0	0	0	0	0	0	0	0
0	0	0	0	0	0	0	0
0	0	0	0	0	0.04172	0.00247	0.05454
0	0	0	0	0	0.04172	0.00247	0.05454
0.00143	0	0	0.0012	0	0	0.00319	0
0	0	0	0	0	0	0	0
0.00143	0	0	0.0012	0	0	0.00319	0
0	0	0	0	0	0	0	0
0	0	0	0	0	0	0	0
0.00121	0	0.64727	2.96892	0.61575	0.74401	0	0.99713
0	0	0.25864	0.13238	0.22457	0	0	0
0	0	0.19598	2.04457	0.22064	0	0	0
0	0	0.00032	0.0007	0	0	0	0
0.00121	0	0.15264	0.78747	0.12972	0.74401	0	0.99713
0	0	0	0	0	0	0	0
0	0	0.03971	0.0038	0.04082	0	0	0
0	0	0	0	0	0.49724	0.00451	0.27687
0	0	0	0	0	0.49724	0.00451	0.27687
0	0	0	0	0	0	0	0
0	0	0	0	0	0	0	0
0	0	0	0.00022	0	0	0	0
0	0	0	0.00022	0	0	0	0
2.22714	0.18825	0.00111	0.00104	0.0025	0.1805	0	0.33492
2.22714	0.18825	0.00111	0.00104	0.0025	0.1805	0	0.33492

0	0	3.99045	1.2884	3.54136	0	0	0
0	0	3.99045	1.2884	3.54136	0	0	0
0	0	3.07204	0.75838	2.634	0	0	0
0	0	0.91841	0.53002	0.90736	0	0	0
0.07719	0.00544	0	0	0	0.1365	0.66962	0.0206
0	0	0	0	0	0	0.6634	0
0	0	0	0	0	0	0.6634	0
0	0	0	0	0	0	0	0
0	0	0	0	0	0	0	0
0	0	0	0	0	0.1365	0	0.0206
0	0	0	0	0	0.1365	0	0.0206
0	0	0	0	0	0	0	0
0	0	0	0	0	0	0	0
0	0	0	0	0	0	0	0
0	0	0	0	0	0	0	0
0.07719	0	0	0	0	0	0.00623	0
0	0	0	0	0	0	0	0
0.07719	0	0	0	0	0	0.00623	0
0	0.00544	0	0	0	0	0	0
0	0.00544	0	0	0	0	0	0
0	0	0	0	0	0	0	0
0	0	0	0	0	0	0	0
13.0812	3.71757	6.39217	11.76571	6.518	6.85102	0.14977	7.97573
0.00344	0.09892	0.09013	0.05455	0.1356	0.09464	0	0.18453
0.00344	0.09892	0.09013	0.05455	0.1356	0.09464	0	0.18453
0	0	0	0	0	0	0	0
0	0	0	0	0	0	0	0
0	0	0.00944	0.01593	0.0159	0	0	0
0	0	0.00944	0.01593	0.0159	0	0	0
0.01317	0	0.02654	0.9574	0.02637	0.05137	0	0.08586
0.01317	0	0.02654	0.9574	0.02637	0.05137	0	0.08586
12.40887	3.36341	2.76175	0.64038	3.40682	0.0002	0	0
12.40887	3.36341	2.76175	0.64038	3.40682	0.0002	0	0
0.62989	0.19602	0.22938	1.04413	0.19661	6.34852	0.1439	7.37894
0.62989	0.19602	0.2206	1.01661	0.18219	6.34852	0.1439	7.37894

0	0	0.00878	0.02752	0.01441	0	0	0
0	0	0.12251	0.54584	0.17048	0	0	0
0	0	0.12251	0.54584	0.17048	0	0	0
0	0	0	0	0	0	0	0
0	0	0	0	0	0	0	0
0	0	0	0	0	0.0056	0	0.00648
0	0	0	0	0	0	0	0
0	0	0	0	0	0.0056	0	0.00648
0.0012	0	0.1195	0.06705	0.08327	0.01428	0	0.02733
0.0012	0	0.1195	0.01997	0.08327	0.00444	0	0.01032
0	0	0	0.04708	0	0.00984	0	0.01701
0	0	0	0	0	0	0	0
0	0	0	0	0	0	0	0
0	0	0	0	0	0	0	0
0	0	2.65218	0.10671	2.15202	0	0	0
0	0	0.71662	0.00424	0.56754	0	0	0
0	0	1.31591	0.02778	0.97378	0	0	0
0	0	0	0	0	0	0	0
0	0	0.61965	0.07468	0.61069	0	0	0
0	0	0	0	0	0	0	0
0.02463	0.05922	0.38074	8.33373	0.33093	0.33641	0.00587	0.2926
0.02463	0.05922	0.38074	8.33373	0.33093	0.33641	0.00587	0.2926
0.29323	1.03783	0.10587	0.06316	0.19474	0.4584	0.08928	0.45156
0.29323	1.03783	0.10587	0.06316	0.19474	0.4584	0.08928	0.45156
0.29323	1.03783	0.10587	0.06316	0.19474	0.4584	0.08928	0.45156
0	0	0	0	0	0	0	0
0	0	0	0	0	0	0	0
0	0	0	0	0	0	0	0
0	0	0	0	0	0	0	0
0	0	0	0	0	0	0	0
0	0	0	0	0	0	0	0
0	0.00921	0.01271	0	0.01201	0.06733	0.01212	0.10238
0	0.00921	0.01271	0	0.01201	0.06733	0.01212	0.10238
0.01808	0.01648	0.03235	0	0.03223	0.11591	0	0.14207
0.01808	0.01648	0.03235	0	0.03223	0.11591	0	0.14207

0	0	0	0	0	0	0	0
0	0	0	0	0	0	0	0
0.27359	0.90989	0.00103	0.05695	0.00227	0.27049	0.07716	0.20637
0.16677	0.12615	0.00103	0	0.00227	0.02557	0	0.02225
0	0	0	0	0	0.03567	0.02331	0.00947
0.10682	0.78374	0	0.05695	0	0.20926	0.05385	0.17465
0	0	0	0	0	0	0	0.00035
0	0	0	0	0	0	0	0.00035
0	0	0	0	0	0	0	0
0	0	0	0	0	0	0	0
0	0	0	0	0	0	0	0
0	0	0	0	0	0	0	0
0	0	0	0	0	0	0	0
0	0	0	0	0	0	0	0
0.00155	0.01827	0.05977	0.0062	0.14822	0	0	0
0.00155	0.01827	0.05977	0.0062	0.14822	0	0	0
0	0	0	0	0	0	0	0
0	0	0	0	0	0	0	0
0	0	0	0	0	0	0	0
0	0	0	0	0	0	0	0
0	0.08398	0	0	0	0.00467	0	0.00039
0	0.08398	0	0	0	0.00467	0	0.00039
0.00147	0	1.00066	0.38608	1.15188	0.3246	0	0.28886
0.00147	0	1.00066	0.38608	1.15188	0.3246	0	0.28886
0.00147	0	1.00066	0.38608	1.15188	0.3246	0	0.28886
0.00147	0	1.00066	0.38608	1.15188	0.3246	0	0.28886
0	0	0.2511	0.05641	0.2629	0	0	0
0	0	0.01259	0.00667	0.0131	0.05551	0	0.07727
0	0	0.00028	0.00328	0.0005	0	0	0
0	0	0	0	0	0.26909	0	0.21159
0	0	0	0	0	0	0	0
0.00147	0	0.69106	0.31762	0.83428	0	0	0
0	0	0	0	0	0	0	0
0	0	0.04563	0.00212	0.0411	0	0	0
3.10252	4.28564	0.47184	0.22056	0.44157	2.37608	1.61596	2.10579

2.3707	0.09039	0.34581	0.22056	0.36931	2.36824	1.24008	2.10235
2.3707	0.09039	0.34581	0.22056	0.36931	2.36824	1.24008	2.10235
1.71085	0.01484	0	0	0	0	0	0
1.71085	0.01484	0	0	0	0	0	0
0	0	0	0	0	0	0	0
0.65985	0.07554	0.34581	0.22056	0.36931	2.36824	1.24008	2.10235
0.65985	0.07554	0.34581	0.22056	0.36931	2.36824	1.24008	2.10235
0	0	0	0	0	0	0	0
0.30055	1.8544	0	0	0	0	0	0
0.30055	1.8544	0	0	0	0	0	0
0.30055	1.8544	0	0	0	0	0	0
0.26623	1.20674	0	0	0	0	0	0
0.03431	0.64767	0	0	0	0	0	0
0	0	0	0	0	0	0	0
0	0	0	0	0	0	0	0
0	0	0	0	0	0	0	0
0	0	0	0	0	0	0	0
0	0	0	0	0	0	0	0
0	0	0	0	0	0	0	0
0	0	0	0	0	0	0	0
0.43128	2.34085	0.12603	0	0.07226	0.00784	0.37588	0.00345
0.43128	2.34085	0.12603	0	0.07226	0.00784	0.37588	0.00345
0	0	0	0	0	0.00174	0	0.00206
0	0	0	0	0	0.00174	0	0.00206
0	0	0	0	0	0	0.00433	0
0	0	0	0	0	0	0.00433	0
0.14297	0.02639	0.00288	0	0.00113	0	0.11743	0
0	0	0.00288	0	0.00113	0	0.00151	0
0.06564	0	0	0	0	0	0.11593	0
0.07732	0.02639	0	0	0	0	0	0
0.07104	2.2786	0	0	0	0.00252	0.02354	0
0.04082	2.2786	0	0	0	0	0	0
0	0	0	0	0	0	0	0
0.03022	0	0	0	0	0.00252	0.02354	0
0	0	0	0	0.00206	0	0	0

0	0	0	0	0.00206	0	0	0
0.21727	0.03587	0.12315	0	0.06908	0.00358	0.23058	0.00139
0.09672	0.00794	0.05536	0	0.02055	0.00358	0.02163	0.00139
0.01307	0.01118	0.00229	0	0.00262	0	0.00517	0
0.00442	0.00565	0.00784	0	0.00375	0	0.0002	0
0.09048	0.00137	0.05603	0	0.04215	0	0.19795	0
0	0	0	0	0	0	0	0
0	0	0.00164	0	0	0	0	0
0.01258	0.00974	0	0	0	0	0.00562	0
0	0	0	0	0	0	0	0
0	0.00915	0	0	0.00157	0	0.00372	0
0	0.00915	0	0	0.00157	0	0.00372	0
0	0.00915	0	0	0.00157	0	0.00372	0
0	0.00915	0	0	0	0	0	0
0	0.00915	0	0	0	0	0	0
0	0	0	0	0	0	0	0
0	0	0	0	0	0	0	0
0	0	0	0	0	0	0	0
0	0	0	0	0	0	0.00372	0
0	0	0	0	0	0	0.00372	0
0	0	0	0	0	0	0	0
0	0	0	0	0.00157	0	0	0
0	0	0	0	0.00132	0	0	0
0	0	0	0	0	0	0	0
0	0	0	0	0.00025	0	0	0
1.05292	0.24572	0	0.02744	0	1.40162	4.27164	1.14073
1.05292	0.24572	0	0.02744	0	1.40162	4.27164	1.14073
1.05292	0.24572	0	0.02744	0	1.40162	4.27164	1.14073
1.05292	0.24572	0	0.02744	0	1.40162	4.27164	1.14073
0	0	0	0	0	0	0	0
0	0	0	0	0	0	0	0
1.05292	0.24572	0	0.02744	0	1.40162	4.27164	1.14073
0	0	0	0	0	0	0	0
0	0	0	0	0	0	0	0
0	0	0	0	0	0	0	0

0	0	0	0	0	0	0	0
0	0	0	0.02744	0	0	0	0
0	0	0	0	0	0	0	0
0	0	0	0	0	0	0	0
1.05292	0.24572	0	0	0	1.40162	4.27164	1.14073
0	0	0	0	0	0	0	0
0	0	0	0	0	0	0	0
0	0	0	0	0	0	0	0
0	0	0	0	0	0	0	0
0	0	0	0	0	0	0	0
0	0	0	0	0	0	0	0
0	0	0	0	0	0	0	0
0	0	0	0	0	0	0	0
0	0	0	0	0	0	0	0
0	0	0	0	0	0	0	0
0	0	0	0	0	0	0	0
0	0	0	0	0	0	0	0
0	0	0	0	0	0	0	0
0.76091	0.97088	9.48469	13.51489	7.97489	14.01782	5.41853	15.83438
0.02512	0.01097	2.57568	0.01327	1.74932	1.04783	0.20557	1.14923
0.01425	0.01097	2.57568	0.01327	1.74828	1.04783	0.20185	1.14923
0	0	0	0	0	0	0	0
0	0	0	0	0	0	0	0
0	0	0	0	0	0	0	0
0	0	0	0	0	0	0	0
0	0	0	0	0	0	0	0
0	0	0	0	0	0	0	0
0	0	0.20145	0.00978	0.25081	0.07068	0	0.06158
0	0	0.20145	0.00978	0.25081	0.07068	0	0.06158
0	0	0.20145	0.00978	0.25081	0.07068	0	0.06158
0.01425	0.01097	2.37424	0.00349	1.49747	0.97714	0.20185	1.08765
0	0	2.36526	0.00349	1.49063	0.97303	0.2014	1.0809
0	0	2.36526	0.00349	1.49063	0.97303	0.2014	1.0809
0.01425	0.01097	0	0	0	0	0	0
0.01425	0.01097	0	0	0	0	0	0

0	0	0.00897	0	0.00684	0.00411	0.00045	0.00676
0	0	0.00897	0	0.00684	0.00411	0.00045	0.00676
0.01087	0	0	0	0.00104	0	0.00372	0
0.01087	0	0	0	0.00104	0	0.00372	0
0.00178	0	0	0	0	0	0.00372	0
0.00178	0	0	0	0	0	0.00372	0
0.00909	0	0	0	0.00104	0	0	0
0	0	0	0	0	0	0	0
0.00198	0	0	0	0.00038	0	0	0
0	0	0	0	0	0	0	0
0	0	0	0	0	0	0	0
0.00711	0	0	0	0.00066	0	0	0
0.04656	0.00354	0.15713	0.02481	0.19738	0.09817	0.02455	0.09177
0.04656	0.00354	0.15713	0.02481	0.19738	0.09817	0.02455	0.09177
0.04656	0.00354	0.15713	0.02481	0.19738	0.09817	0.02455	0.09177
0.04656	0.00354	0.15713	0.02481	0.19738	0.09817	0.02455	0.09177
0.04656	0.00354	0.15713	0.02481	0.19738	0.09817	0.02455	0.09177
0	0	0	0	0	0	0	0
0	0	0	0	0	0	0	0
0	0	0	0	0	0	0	0
0.00241	0	0	0.21781	0	0	0.00061	0
0.00241	0	0	0.21781	0	0	0.00061	0
0.00241	0	0	0.21781	0	0	0.00061	0
0.00241	0	0	0.21781	0	0	0.00061	0
0.00241	0	0	0.21781	0	0	0	0
0	0	0	0	0	0	0.00061	0
0	0	0	0	0	0	0	0
0	0	0	0	0	0	0	0
0	0	0	0	0	0	0	0
0	0	0	0	0	0	0	0
0	0	0	0	0	0	0	0
0	0	0	0	0	0	0	0
0	0	0	0	0	0	0	0
0.68682	0.95637	6.66267	13.259	5.97207	12.82676	5.17864	14.54303
0	0	0	0	0	0	0	0

0	0	0	0	0	0	0	0
0	0	0	0	0	0	0	0
0	0	0	0	0	0	0	0
0	0	0	0	0	0	0	0
0	0	0	0	0	0	0	0
0	0	0	0	0	0	0	0
0	0	0	0	0	0	0	0
0	0	0	0	0	0	0	0
0	0	0	0	0	0	0	0
0	0	0	0	0	0	0	0
0	0	0	0	0	0	0	0
0	0	0	0	0	0	0	0
0	0	0	0	0	0	0	0
0.65266	0.94922	6.61729	10.19341	5.9487	12.82676	5.17864	14.54303
0	0	0	0	0	0	0	0
0	0	0	0	0	0	0	0
0	0	0	0	0	0	0	0
0.65266	0.94922	6.61729	10.19341	5.9487	12.82676	5.17864	14.54303
0	0	0	0.01043	0	0	0	0
0	0	0	0	0	0	0	0
0	0	0	0	0	0	0	0
0	0	0	0	0	0	0	0
0	0	0	0	0	0	0	0
0	0	0	0	0	0	0	0
0	0	0	0.01043	0	0	0	0
0	0	0	0	0	0	0	0
0	0	0	0	0	0	0	0
0	0	0	0	0	0	0	0
0	0	0	0	0	0	0	0
0	0	0	0	0	0	0	0
0	0	0	0	0	0	0	0
0	0	0	0	0	0	0	0
0	0	0	0	0	0	0	0
0	0	0	0	0	0	0	0
0	0	0	0	0	0	0	0

0	0	0	0	0	0	0	0
0.65266	0.94922	0.37489	0.18606	0.15293	12.65453	5.17864	14.23835
0.65266	0.94922	0.37489	0.18606	0.15293	12.65453	5.17864	14.2373
0	0	0	0	0	0	0	0.00105
0	0	6.2424	9.99692	5.79577	0.17223	0	0.30468
0	0	0	0.09948	0	0	0	0
0	0	0	0	0	0	0	0
0	0	0	0	0	0	0	0
0	0	4.6788	7.50671	4.37517	0.13093	0	0.23475
0	0	0.48124	0.7572	0.38959	0.00774	0	0.01895
0	0	1.08236	1.63353	1.03101	0.03356	0	0.05098
0	0	0	0	0	0	0	0
0	0	0	0	0	0	0	0
0	0	0	0	0	0	0	0
0	0	0	0	0	0	0	0
0	0	0	0	0	0	0	0
0	0	0	0	0	0	0	0
0	0	0	0	0	0	0	0
0	0	0	0	0	0	0	0
0	0	0	0	0	0	0	0
0	0	0	0	0	0	0	0
0	0	0	0	0	0	0	0
0	0	0	0	0	0	0	0
0	0	0	0	0	0	0	0
0	0	0	0	0	0	0	0
0	0	0	0	0	0	0	0
0	0	0	0	0	0	0	0
0.03416	0.00715	0.04538	0.00482	0.02337	0	0	0
0.03416	0.00715	0.04538	0.00482	0.02337	0	0	0
0	0	0	0	0	0	0	0
0	0	0	0	0	0	0	0
0	0	0	0	0	0	0	0
0.03416	0.00715	0.04538	0.00482	0.02337	0	0	0
0	0	0	0	0	0	0	0
0	0	0	0	0	0	0	0

0.03416	0.00715	0.04538	0.00482	0.02154	0	0	0
0	0	0	0	0	0	0	0
0	0	0	0	0	0	0	0
0	0	0	0	0.00183	0	0	0
0	0	0	0	0	0	0	0
0	0	0	3.06077	0	0	0	0
0	0	0	0	0	0	0	0
0	0	0	0	0	0	0	0
0	0	0	0	0	0	0	0
0	0	0	3.06077	0	0	0	0
0	0	0	3.06077	0	0	0	0
0	0	0	3.06077	0	0	0	0
0	0	0.08921	0	0.05612	0.04507	0.00916	0.05035
0	0	0.08921	0	0.05612	0.04507	0.00916	0.05035
0	0	0.08921	0	0.05612	0.04507	0.00916	0.05035
0	0	0.08921	0	0.05612	0.04507	0.00916	0.05035
0	0	0.08921	0	0.05612	0.04507	0.00916	0.05035
0	0	0.07255	0.08435	0.09709	0.00925	0	0.01797
0	0	0.07255	0.08435	0.09709	0.00925	0	0.01797
0	0	0.07255	0.08435	0.09709	0.00925	0	0.01797
0	0	0.07255	0.08435	0.09709	0.00925	0	0.01797
0	0	0.02882	0.01343	0.03365	0.00925	0	0.01797
0	0	0.01776	0.00956	0.0176	0	0	0
0	0	0.01106	0.00387	0.01605	0.00925	0	0.01797
0	0	0	0	0.00023	0	0	0
0	0	0	0	0.00023	0	0	0
0	0	0.04373	0.07092	0.06322	0	0	0
0	0	0.04373	0.07092	0.06293	0	0	0
0	0	0	0	0.00029	0	0	0
0	0.00741	0	0	0	0	0	0
0	0.00741	0	0	0	0	0	0
0	0.00741	0	0	0	0	0	0
0	0.00741	0	0	0	0	0	0
0	0.00741	0	0	0	0	0	0
0	0.00741	0	0	0	0	0	0

0	0	0	0	0	0	0	0
0	0	0	0	0	0	0	0
0	0	0	0	0	0	0	0
0	0	0	0	0	0	0	0
0	0	0	0	0	0	0	0
0	0	0	0	0	0	0	0
0	0	2.32279	1.91398	1.23005	0	0	0
0	0	2.32279	1.91398	1.23005	0	0	0
0	0	2.32279	1.91398	1.23005	0	0	0
0	0	2.32279	1.91398	1.23005	0	0	0
0	0	2.32279	1.91398	1.23005	0	0	0
0	0	2.3026	1.9056	1.2091	0	0	0
0	0	0	0	0	0	0	0
0	0	2.3026	1.9056	1.2091	0	0	0
0	0	0.02019	0.00838	0.02095	0	0	0
0	0	0.02019	0.00838	0.02095	0	0	0
0	0	0	0	0	0.00052	0	0.00063
0	0	0	0	0	0.00052	0	0.00063
0	0	0	0	0	0.00052	0	0.00063
0	0	0	0	0	0.00052	0	0.00063
0	0	0	0	0	0	0	0
0	0	0	0	0	0	0	0
0	0	0	0	0	0	0	0
0	0	0	0	0	0.00052	0	0.00063
0	0	0	0	0	0.00052	0	0.00063
0	0	0	0	0	0.00052	0	0.00063

AP020_1	AP020_2	AP020_3
100	100	100
0.04076	0.05655	0.08382
0.031	0.03506	0.06805
0	0.0048	0
0	0.0048	0
0	0	0
0	0	0
0	0.0048	0
0	0	0
0	0	0
0	0	0
0	0	0
0	0	0
0	0	0
0	0.00287	0
0	0	0
0	0	0
0	0	0
0	0	0
0	0.00193	0
0	0	0
0	0	0
0	0	0
0	0	0
0	0	0
0	0	0
0.031	0.03019	0.06805
0.031	0.03019	0.06805
0	0	0
0	0	0
0	0	0
0	0	0
0.031	0.03019	0.06805

0	0	0
0	0	0
0	0	0
0	0	0
0	0	0
0.031	0.03019	0.06805
0	0	0
0	0	0
0	0	0
0	0	0
0	0	0
0	0	0
0	0	0
0	0	0
0	0	0
0	0	0
0	0	0
0	0.00007	0
0	0.00007	0
0	0.00007	0
0	0	0
0	0	0
0	0.00007	0
0	0	0
0	0	0
0	0	0
0	0	0
0	0	0
0	0	0
0.00976	0.02149	0.01577
0.00395	0.00597	0.00603
0	0.0002	0
0	0.0002	0
0	0	0
0	0.0002	0

0	0	0
0	0	0
0	0	0
0	0	0
0.00395	0.00577	0.00603
0.00395	0.00577	0.00603
0.00395	0.00577	0.00603
0	0	0
0	0	0
0	0	0
0	0	0
0	0	0
0.00581	0.01552	0.00974
0.00581	0.01552	0.00974
0.0035	0.00251	0.00659
0.0035	0.00251	0.00659
0.00082	0.00061	0.00155
0.00082	0.00061	0.00155
0.00135	0.0095	0.0016
0.00135	0.0095	0.0016
0	0	0
0	0	0
0.00014	0.00129	0
0.00014	0.00129	0
0	0.00161	0
0	0.00161	0
85.52464	83.72484	80.74614
0	0	0
0	0	0
0	0	0
0	0	0
0	0	0
85.52464	83.72484	80.74614
85.52464	83.72484	80.74614
67.76315	60.92915	62.97679

67.76315	60.92915	62.97679
6.18699	2.29655	4.24415
1.36553	2.97274	1.95056
0	0	0
0.1428	0.48769	0.19625
0	0	0
0.00013	0	0.00015
1.88949	1.70951	1.6556
0	0	0
0.10654	0.33975	0.06958
1.96077	1.74453	3.10685
1.6044	3.75983	1.56874
0.02766	0.06305	0.03248
0.74863	0.53066	0.95485
0	0	0
0.63042	3.40351	0.60863
0	0	0
2.59772	2.56357	3.93959
10.37088	13.38691	13.25289
1.0754	3.17338	1.19323
4.81618	7.91281	5.8451
29.21876	12.25141	21.84792
5.02083	4.33324	2.51022
0	0	0
0	0	0
0	0	0
0	0	0
0	0	0
0	0	0
0.69968	0.86623	0.86649
0.69968	0.86623	0.86649
0.00273	0.00585	0.00226
0.69696	0.86038	0.86423
0	0	0
0	0	0

0	0	0
0	0	0
0	0	0
0	0	0
0	0	0
0	0	0
0	0	0
0	0	0
8.28141	13.38875	7.39229
0	0.00253	0
0	0.00253	0
0	0	0
0	0	0
0	0	0
8.28141	13.38621	7.39229
0.00093	0.00171	0
0	0.03891	0
0	0	0
0	0	0
0	0	0
0	0.00549	0
0	0	0
0	0.1534	0
0	0	0
0	0	0
0	0.00756	0
0	0.00821	0
0	0	0
0	0	0
0	0	0
8.28048	13.17093	7.39229
0	0	0
0	0	0
0.05105	0.28199	0.06287
0.05105	0.28199	0.06287

0	0	0
0.04957	0.28062	0.06112
0	0	0
0	0	0
0.00144	0.00132	0.00175
0.00004	0.00005	0
8.72935	8.25874	9.4477
8.72935	8.25874	9.4477
7.26965	7.41421	7.96212
0.31021	0.0211	0.34074
0.00245	0.00092	0.00316
0	0	0
1.14704	0.82251	1.14168
0	0	0
0	0	0
0	0	0
0	0	0
0	0	0
0	0	0
0	0	0
13.4361	13.64101	18.32824
0.01751	1.54173	0.03198
0	0.02402	0
0	0.02402	0
0	0.02402	0
0	0	0
0	0.007	0
0	0.00086	0
0	0.01616	0
0.01751	1.51771	0.03198
0	0	0
0	0	0
0	0	0
0	0	0
0	0	0

0	0	0
0	0	0
0	0	0
0	0	0
0	0	0
0	0	0
0	0	0
0	0	0
0	0	0
0	0	0
0	0	0
0	0	0
0	0	0
0	0	0
0	0	0
0.00694	0.00875	0.00543
0.00694	0.00875	0.00543
0	0	0
0	0	0
0	0.00225	0
0	0	0
0	0	0
0	0	0
0	0	0
0.00694	0	0.00543
0	0.0065	0
0	0	0
0	0	0
0	0	0
0	0	0
0	0	0
0	0	0
0	0	0
0	0	0
0.01057	1.50896	0.02655

0.01057	1.50896	0.02655
0	0.26924	0
0	0.00177	0
0	0	0
0	0	0
0	0.01063	0
0	0.09428	0
0	0	0
0	0.00134	0
0.00016	0.03922	0
0	0	0
0.00026	0.33397	0.00111
0.01	0.66322	0.02531
0	0	0
0	0	0
0.00007	0.01491	0
0.00008	0.00919	0.00013
0	0	0
0	0.0012	0
0	0.00485	0
0	0	0
0	0	0
0	0	0
0	0	0
0	0.02792	0
0	0	0
0	0	0
0	0.03723	0
0	0	0
12.2119	3.73317	16.50196
12.2119	3.73317	16.50196
0.0016	0.00053	0.00387
0.0016	0.00053	0.00387
0.0016	0.00053	0.00387
0	0	0

0	0	0
0	0	0
0.24023	0.36299	0.3491
0	0	0
0	0	0
0.20664	0.27041	0.27863
0	0	0
0.16702	0.27041	0.25568
0	0	0
0	0	0
0	0	0
0	0	0
0	0	0
0	0	0
0	0	0
0.03881	0	0.02221
0	0	0
0	0	0
0	0	0
0.00081	0	0.00074
0	0	0
0.03359	0.09258	0.07047
0	0	0
0.03359	0.09258	0.07047
0	0	0
0	0	0
0.00055	0.00915	0.00098
0	0	0
0	0	0
0	0	0
0	0	0
0	0	0
0.00055	0.00915	0.00098
0.00055	0.00915	0.00098
0.05204	0.00613	0.1232

0.00307	0.00309	0.00791
0.00307	0.00309	0.00791
0.03554	0.00304	0.09229
0.03554	0.00304	0.09229
0.01342	0	0.023
0.01342	0	0.023
0	0	0
0	0	0
1.09772	0.03546	1.67555
0	0	0
0	0	0
0	0	0
0	0	0
1.09772	0.03546	1.67555
0	0	0
0	0	0
0.75925	0.00213	1.10452
0	0	0
0.00026	0.00082	0.00042
0.00908	0	0.01831
0	0	0
0.32913	0.03251	0.5523
0	0	0
0	0	0
0	0	0
0	0	0
0	0	0
0	0	0
5.42428	2.79969	5.83839
0.0085	0.04907	0.00693
0.00147	0	0.00073
0.00702	0.04907	0.0062
0	0	0
0	0	0
0.23091	0.37642	0.25611

0	0	0
0	0	0
0.02262	0.00464	0.0108
0.00443	0.01076	0.0023
0.00944	0.0009	0.01309
0	0	0
0.00767	0.26139	0.01031
0.18675	0.09872	0.2196
0	0	0
0	0	0
0	0	0
0.03256	0	0.04363
0.00277	0	0.00394
0.02979	0	0.03969
0.00109	0.0019	0.00255
0	0	0
0.00109	0.0019	0.00255
0	0	0
0	0	0
0	0	0
0	0	0
0	0	0
0	0	0
0.01097	0	0.01243
0.01097	0	0.01243
0	0	0
0	0	0
0.61894	1.07753	1.11739
0.00203	0.00705	0.00523
0.045	0.03754	0.07858
0.437	0.91773	0.77435
0.05175	0.07617	0.10473
0	0	0
0	0	0
0.00304	0.00263	0.00347

0.04884	0.00152	0.08821
0.00464	0.00008	0.00223
0.02664	0.03483	0.06058
0	0	0
0	0	0
1.94841	0.02435	2.29118
0	0	0
0	0	0
0	0	0
0	0	0
1.94841	0.02435	2.29118
0	0	0
0	0	0
0	0	0
0	0	0
0	0.00062	0
0	0	0
0	0.00062	0
0	0	0
0	0	0
2.51815	1.26969	2.0393
2.36649	1.20692	1.92684
0	0	0
0.14708	0	0.10785
0.00458	0.06277	0.0046
0	0	0
0	0	0
0.05083	0	0.06518
0.05083	0	0.06518
0	0	0
0	0	0
0	0	0
0	0	0
0.00394	0.00012	0.00368
0.00394	0.00012	0.00368

0	0	0
0	0	0
0	0	0
0	0	0
0	0.00108	0
0	0	0
0	0	0
0	0	0
0	0	0
0	0	0
0	0	0
0	0	0
0	0	0
0	0	0
0	0	0
0	0.00108	0
0	0	0
0	0.00108	0
0	0	0
0	0	0
0	0	0
0	0	0
5.39549	0.51814	8.51086
0.03003	0.02755	0.09265
0.03003	0.02755	0.09265
0	0	0
0	0	0
0	0	0
0	0	0
0.00563	0.12429	0.02103
0.00563	0.12429	0.02103
3.48457	0.25631	5.87865
3.48457	0.25631	5.87865
0.7083	0.04588	1.16335
0.7083	0.04588	1.16335

0	0	0
0.00254	0	0.00165
0.00254	0	0.00165
0	0	0
0	0	0
0	0	0
0	0	0
0	0	0
0.03453	0	0.0231
0.03453	0	0.0231
0	0	0
0	0	0
0	0	0
0	0	0
1.06839	0.05662	1.12789
0.14714	0	0.09868
0.83868	0.05662	1.00568
0.08217	0	0.02352
0.00039	0	0
0	0	0
0.0615	0.00749	0.20254
0.0615	0.00749	0.20254
0.21915	0.03391	0.10793
0.21915	0.03391	0.10793
0.21915	0.03391	0.10793
0	0	0
0	0	0
0	0	0
0	0	0
0	0	0
0	0	0
0.01219	0.00097	0.00021
0.01219	0.00097	0.00021
0	0	0
0	0	0

0	0	0
0	0	0
0.18634	0.03073	0.06914
0.18468	0.01265	0.06614
0	0	0
0.00166	0.01808	0.003
0	0	0
0	0	0
0	0	0
0	0	0
0	0	0
0	0	0
0	0	0
0	0	0
0.02062	0.00221	0.03858
0.02062	0.00221	0.03858
0	0	0
0	0	0
0	0	0
0	0	0
0	0	0
0	0	0
0.3574	0.03285	0.62145
0.3574	0.03285	0.62145
0.3574	0.03285	0.62145
0.3574	0.03285	0.62145
0.00086	0	0.00182
0	0	0
0	0	0
0	0	0
0	0	0
0.35565	0.03285	0.61716
0	0	0
0.0009	0	0.00246
0.63014	8.27557	1.06493

0.62896	3.64863	1.06373
0.62896	3.64863	1.06373
0.03079	2.89528	0.04336
0.03079	2.89528	0.04336
0	0	0
0.59817	0.75335	1.02036
0.59817	0.75335	1.02036
0	0	0
0	0.00064	0
0	0.00064	0
0	0	0
0	0	0
0	0	0
0	0	0
0	0	0
0	0.00064	0
0	0	0
0	0	0
0	0	0
0	0.00064	0
0.00118	4.62631	0.0012
0.00118	4.62631	0.0012
0	0.00118	0
0	0.00118	0
0	0.00264	0
0	0.00264	0
0	0.23312	0
0	0.00401	0
0	0.22911	0
0	0	0
0.00118	0.838	0.0012
0.00108	0.67222	0.00113
0.00009	0.00267	0.00007
0	0.16311	0
0	0	0

0	0	0
0	3.55137	0
0	1.89005	0
0	0.22759	0
0	0.07019	0
0	1.09491	0
0	0	0
0	0	0
0	0.22958	0
0	0.03904	0
0	0.02378	0
0	0.02378	0
0	0.02378	0
0	0	0
0	0	0
0	0	0
0	0	0
0	0	0
0	0.02378	0
0	0.02378	0
0	0	0
0	0	0
0	0	0
0	0	0
0	0	0
0.18047	1.61295	0.10237
0.18047	1.61295	0.10237
0.18047	1.61295	0.10237
0.18047	1.61295	0.10237
0	0	0
0	0	0
0.18047	1.61295	0.10237
0	0	0
0	0	0
0	0.0056	0

0	0.12888	0
0	0.1288	0
0	0.06413	0
0	0	0
0.18047	1.28554	0.10237
0	0	0
0	0	0
0	0	0
0	0	0
0	0	0
0	0	0
0	0	0
0	0	0
0	0	0
0	0	0
0	0	0
0	0	0
0	0	0
0.77678	0.7369	0.64463
0.32497	0.07252	0.37005
0.32497	0.05981	0.37005
0	0	0
0	0	0
0	0	0
0	0	0
0	0	0
0	0	0
0.04722	0.01602	0.05095
0.04722	0.01602	0.05095
0.04722	0.01602	0.05095
0.27775	0.04379	0.3191
0.26197	0.04062	0.30089
0.26197	0.04062	0.30089
0	0	0
0	0	0

0.01578	0.00317	0.01821
0.01578	0.00317	0.01821
0	0.01272	0
0	0.01272	0
0	0	0
0	0	0
0	0.01272	0
0	0	0
0	0.00577	0
0	0.00065	0
0	0.0052	0
0	0.0011	0
0.14414	0.06135	0.14643
0.14414	0.06135	0.14643
0.14414	0.06135	0.14643
0.14266	0.06067	0.14514
0.14266	0.06067	0.14514
0.00148	0.00068	0.00129
0.00148	0.00068	0.00129
0	0	0
0	0.00219	0
0	0.00219	0
0	0.00219	0
0	0.00219	0
0	0.00032	0
0	0.0005	0
0	0.00137	0
0	0	0
0	0	0
0	0	0
0	0	0
0	0	0
0	0	0
0.29427	0.59969	0.1142
0	0	0

0	0	0
0	0	0
0	0	0
0	0	0
0	0	0
0	0	0
0	0	0
0	0	0
0	0	0
0	0	0
0	0	0
0	0	0
0	0	0
0.29411	0.49591	0.1142
0	0	0
0	0	0
0	0	0
0.29411	0.49591	0.1142
0	0	0
0	0	0
0	0	0
0	0	0
0	0	0
0	0	0
0	0	0
0	0	0
0	0	0
0	0	0
0	0	0
0	0	0
0	0	0
0	0	0
0	0	0
0	0	0
0	0	0

0	0	0
0.29411	0.37555	0.1142
0.29411	0.37555	0.1142
0	0	0
0	0.12037	0
0	0.01075	0
0	0	0
0	0	0
0	0.09733	0
0	0.00203	0
0	0.01025	0
0	0	0
0	0	0
0	0	0
0	0	0
0	0	0
0	0	0
0	0	0
0	0	0
0	0	0
0	0	0
0	0	0
0	0	0
0	0	0
0	0	0
0	0	0
0	0	0
0.00016	0.10378	0
0.00016	0.10378	0
0	0.01673	0
0	0.01472	0
0	0.00201	0
0.00016	0.08705	0
0	0.00132	0
0	0	0

0.00016	0.0816	0
0	0	0
0	0	0
0	0.00412	0
0	0	0
0	0	0
0	0	0
0	0	0
0	0	0
0	0	0
0	0	0
0	0	0
0.01341	0.00114	0.01396
0.01341	0.00114	0.01396
0.01341	0.00114	0.01396
0.01341	0.00114	0.01396
0.01341	0.00114	0.01396
0.04125	0.22774	0.0948
0.04125	0.22774	0.0948
0.04125	0.22774	0.0948
0.04125	0.22774	0.0948
0.04125	0.15796	0.0948
0.00477	0.00697	0.00739
0.03648	0.15098	0.08741
0	0.0022	0
0	0.0022	0
0	0.06758	0
0	0.065	0
0	0.00259	0
0	0	0
0	0	0
0	0	0
0	0	0
0	0	0
0	0	0

0	0	0
0	0	0
0	0	0
0	0	0
0	0	0
0	0	0
0	0	0
0	0	0
0	0	0
0	0	0
0	0	0
0	0	0
0	0	0
0	0	0
0	0	0
0	0	0
0	0	0
0	0	0
0	0	0
0	0	0
0	0	0
0	0	0
0	0	0
0	0	0
0	0	0
0	0	0

Table S3. Differential species across treatment phase, severity, and etiology

SampleID	Adjust P-values	Difference between means	95.0% lower CI	95.0% upper CI	Comparison groups	Enriched group
Eubacterium_sp_CAG_274	0.042620773	0.02361105	0.000868053	0.046354048	Pre vs. On-treatment	Pre-treatment
Fusicatenibacter_saccharivorans	0.03525162	0.199433696	0.015298144	0.383569248	Pre vs. On-treatment	Pre-treatment
Lachnospira_pectinoschiza	0.030816166	0.865322608	0.08929132	1.641353896	Pre vs. On-treatment	Pre-treatment
Oribacterium_sinus	0.046590666	-0.000428421	-0.00084964	-0.0000072	Pre vs. On-treatment	On-treatment
Roseburia_inulinivorans	0.035832265	1.153351435	0.08510331	2.22159956	Pre vs. On-treatment	Pre-treatment
Streptococcus_parasanguinis	0.04822434	-0.217766306	-0.433655691	-0.00187692	Pre vs. On-treatment	On-treatment
Anaerotruncus_colihominis	0.018179304	-0.014672481	-0.026556517	-0.002788444	Pre vs. Post-treatment	Pre-treatment
Eggerthella_lenta	0.006031167	0.018081956	0.005809009	0.030354902	On vs. Post-treatment	On-treatment
Gordonibacter_pamelaeae	0.023582869	0.002422857	0.000355426	0.004490289	On vs. Post-treatment	On-treatment
Butyricimonas_virosa	0.046404889	0.081964064	0.001418959	0.162509169	On vs. Post-treatment	On-treatment
Blautia_wexlerae	0.016081794	-0.021772329	-0.039129232	-0.004415427	On vs. Post-treatment	Post-treatment
Agathobaculum_butyriciproducens	0.04306713	-0.006655564	-0.013090222	-0.000220906	On vs. Post-treatment	Post-treatment
Anaerotruncus_colihominis	0.036817469	0.019174737	0.00130709	0.037042385	On vs. Post-treatment	On-treatment
Bacteroides_xylanisolvens	0.034155028	1.585618349	0.138597217	3.032639481	MAP[1] vs. SAP[2]	MAP
Clostridium_lavalense	0.014691339	0.020494869	0.004619461	0.036370278	MAP vs. SAP	MAP
Roseburia_inulinivorans	0.035349907	1.663361046	0.134424199	3.192297893	MAP vs. SAP	MAP
s__Alistipes_shahii	0.04233647	0.087769741	0.003530193	0.172009289	BAP[3] vs. HGT-AP[4]	BAP
s__Escherichia_coli	0.004498098	1.326004074	0.490101724	2.161906424	BAP vs. HGT-AP	BAP
s__Rothia_mucilaginosa	0.003863597	0.00070631	0.000271182	0.001141437	BAP vs. HGT-AP	BAP

[1] MAP: mild acute pancreatitis

[2] SAP: severe acute pancreatitis

[3] BP: biliary acute pancreatitis

[4] HP: hypertriglyceridemia-induced pancreatitis

Table S4. The abundance of pathways

Pathway	AP001_1	AP001_2	AP001_3	AP002_1	AP002_2	AP002_3	AP003_1	AP003_2	AP003_3	AP004_1	AP004_2	AP004_3
UNMAPPED	10455143	12948455	13418634	10683290	11304146	11545220	17443825	21186519	29740253	24095191	8611683	11562749
UNINTEGRATED	49587904	58547905	58632983	68926055	81876008	71185898	1E+08	63851000	1.33E+08	63888285	1.73E+08	2.14E+08
DTDPRHAMSYN-PWY: dTDP-β-L-rham	51512.61	53550.74	52724.6	44049.4	60888.22	41884.28	54311.73	27530.28	48411.4	27182.09	46857.44	14980.94
PWY66-429: fatty acid biosynthesis initiation (	38308.81	40572.63	41327.5	29904.42	38030.45	36685.54	38632.07	23103.35	44308.41	24449.65	54597.16	67607.24
VALSYN-PWY: L-valine biosynthesis	37385.83	40469.86	40573.87	29573.49	43491.7	40354.47	53221.75	27359.59	66831.38	33823.87	90162.27	112157.9
PWY-5686: UMP biosynthesis I	36353.13	37628.09	40020.6	26177.68	38741.58	34995.32	38206.13	26932.37	44785.42	26905.79	39398.38	49219.72
PWY-7790: UMP biosynthesis II	36353.13	37628.09	40020.6	26177.68	38741.58	34995.32	38206.13	26932.37	44785.42	26905.79	31892.87	40241.77
PWY-7791: UMP biosynthesis III	36353.13	37628.09	40020.6	26177.68	38741.58	34995.32	38206.13	26932.37	44785.42	26905.79	31892.87	40241.77
PWY-1042: glycolysis IV	35590.75	42208.94	38866	33478.15	34826.11	34299.39	47052.73	36651.12	60623.89	41010.84	105266.9	122915.2
PWY-6609: adenine and adenosine salvage I	35575.61	37575.63	37877.2	27023.19	39073.18	35647.02	40810.71	36623.37	53320.19	38568	53908.07	67388.42
PWY-6163: chorismate biosynthesis from 3-d	34885.27	35520.58	37174.38	26349.34	39696.51	34811.31	34992.64	26493.81	42775.98	26385.38	44223.16	58749.93
NONMEVIPP-PWY: methylerythritol phospha	34684.95	34880.17	36235.42	25752.49	39136.44	35368.1	36283.27	21409.03	42574.29	20903.19	45668.52	51241.95
HISTSYN-PWY: L-histidine biosynthesis	33983.21	33583.26	32156.41	23605.3	40113.8	32771.96	33195.23	16384.48	36331.22	18104.12	36241.91	45924.81
PWY-6385: peptidoglycan biosynthesis III (my	33940.41	36076.55	36219.53	26928.17	41530	37220.7	39982.33	25292.36	50130.44	24422.34	70813.78	86930.59
PWY-6386: UDP-N-acetylmuramoyl-pentape	33737.99	35399.35	36491.51	25447.47	38510.52	35874.49	38236.35	24277.7	47227.6	23008.48	61373.95	73568.37
PWY-7229: superpathway of adenosine nucle	33530.99	38356.71	36158.21	29433.66	33088.6	33529.28	38609.87	20504.1	50833.85	21308.01	36133.61	42326.83
PWY-7221: guanosine ribonucleotides de nov	33495.79	37181.59	37662.72	28352.85	39550.06	35696.1	38955.22	29394.55	51747.91	29275.88	53403.99	65705.53
PWY-6700: queuosine biosynthesis I (de nov	33481.11	34873.2	35535.74	24019.27	44051.75	35305.17	34432.37	20661.47	36987.12	17291.2	50757.93	65550.54
PWY-6387: UDP-N-acetylmuramoyl-pentape	33477.93	35261.94	36243.26	26037.77	40305.23	35912.58	39124.66	24173.87	48211.15	23126.18	66974.11	83197.5
PWY-5695: inosine 5 -phosphate degradation	33418.26	37819.37	35586	29279.11	42754.45	35924.51	46100.59	18090.97	62503.95	21540.22	89510.87	115178
PWY-6121: 5-aminoimidazole ribonucleotide	33250.95	35682.45	34936.69	25731.87	34329.4	33379.84	38371.65	25052.8	49068.6	23259.54	76552.33	88723.04
PEPTIDOGLYCANSYN-PWY: peptidoglycan	32993.1	34890.28	36156.09	25900.01	40184.49	35858.5	38091.9	24255.81	47889.27	23306.73	65750.36	80947.34
PWY-7953: UDP-N-acetylmuramoyl-pentape	32544.6	34175.6	35698.22	25246.34	38376.55	35951.99	37917.27	18274.45	47258.48	16090.2	65569.92	75634.15
PWY-6122: 5-aminoimidazole ribonucleotide	32477.12	35394.83	34138.22	25074.43	32221.49	32250.46	38832.05	25452.64	47557.51	24101.7	78387.6	90298.17
PWY-6277: superpathway of 5-aminoimidazo	32477.12	35394.83	34138.22	25074.43	32221.49	32250.46	38832.05	25452.64	47557.51	24101.7	78387.6	90298.17
PWY0-1586: peptidoglycan maturation (meso	32252.09	37157.14	34893.2	36100.55	49784.29	42026.23	83195.36	32701.7	122042.5	45741.44	265044.9	326898.4
COA-PWY-1: superpathway of coenzyme A b	32031.7	33982.74	33606.34	24639.11	39322.97	34274.76	34102.87	20939.79	41104.91	19886.58	32687.76	41898.26
ARO-PWY: chorismate biosynthesis I	31979.53	33328.24	35803	25726.25	37397.25	33047.91	35879.26	25721.56	44973.09	25774.59	51807.83	67693.66
TRNA-CHARGING-PWY: tRNA charging	31568.09	34665.97	35309.37	25000.79	34045.76	33192.32	37965.39	22633.37	46441.47	23562.74	62137.32	77314.91
PWY-5667: CDP-diacylglycerol biosynthesis I	31439.32	33582.84	34117.46	26596.88	40080.41	34626.71	41328.04	24008.49	56777.14	21921.69	99172.65	155829.4
PWY0-1319: CDP-diacylglycerol biosynthesis	31439.32	33686.28	34122.31	26596.88	40092.49	34635.29	41328.04	24008.49	56777.14	21924.59	99172.65	155830.1
PWY-3841: folate transformations II (plants)	31434.78	34007.25	34094.27	29406.57	42802.86	39265.12	36652.04	29410.55	46129.8	26324.62	42938.16	57083.95
COA-PWY: coenzyme A biosynthesis I (proka	30854.27	32933.42	32334.45	24206.16	36906.38	33287.64	34082.19	19405.67	42154.21	18847.74	34702.35	44212.91

PWY-7851: coenzyme A biosynthesis II (euka	30854.27	32814.97	32334.45	23900.82	36266.89	32234.42	32005.26	19330.26	38406.09	18399.8	29671.1	38525.26
PWY-7977: L-methionine biosynthesis IV	30227.68	33820.13	31687.89	28729.42	47641.78	36262.05	40806.96	18249.2	52217.82	22138.85	63099.13	82360.58
PWY-7238: sucrose biosynthesis II	29993.12	26518.11	34117.72	16918.34	39429.68	33856.29	36606.07	28001.62	50812.72	34438.67	81678.11	98190.3
PWY-5097: L-lysine biosynthesis VI	29981.18	30098.3	32281.35	23256.87	31007.1	28284.3	33726.97	23911.29	44773.17	31378.89	40810.98	53228.39
PWY-2942: L-lysine biosynthesis III	29932.18	31751.41	29290.68	25372.41	37830.75	31886.03	34073.87	23091.58	46506.02	31278.77	48365.62	66430
PWY-6126: superpathway of adenosine nucle	29838.62	35008.4	32506.7	27785.26	27347.61	28828.63	34592.04	16478.79	46817.11	16609.86	36134.53	41826.28
COMPLETE-ARO-PWY: superpathway of arc	29838.16	31337.12	33476.96	22843.07	26725.37	28610.64	34054.4	24233.32	43896.92	24487.25	53431.64	67786.73
PWY-5973: cis-vaccenate biosynthesis	29769.2	32593.27	30844.47	27649.98	40711.66	32508.29	32019.67	9980.443	28717.63	11224.63	36908.69	45542.11
PANTO-PWY: phosphopantothenate biosynth	29741.65	29965.34	29639	26628.22	43270.85	34016.05	34918.52	16338.98	39821.6	12434.78	51173.18	74314.74
PWY-6123: inosine-5 -phosphate biosynthesis	29716.07	32897.94	31306.15	24958.3	38728.8	32544.99	29142.94	12782.71	38812.89	11137.19	48888.31	62060.32
ANAGLYCOLYSIS-PWY: glycolysis III (from g	29404.75	32744.27	33848.91	25435.96	29461.39	29559.24	37517.04	27005.88	48077.11	28681.58	72081.15	81070.46
PWY-7199: pyrimidine deoxyribonucleosides	29354.88	30304.42	30754.36	25708.34	36532.06	32706.69	29199.36	13658.04	30129.74	14445.49	38968.81	50869.06
1CMET2-PWY: folate transformations III (E. c	29238.77	33367.71	31896.47	29714.78	41595.69	36271.61	37991.96	23989.39	46799.52	24562.16	43968.72	61489.3
PANTOSYN-PWY: superpathway of coenzym	29003.35	30810.97	30323.37	24515.25	38907.51	32684.97	32638.63	15465.55	39076.19	11152.37	38234.69	51925.84
PWY-6124: inosine-5 -phosphate biosynthesis	28960.49	32311.7	30388.74	24772.71	38242.51	31783.64	28087.5	11750.37	29609.36	10108.69	32642.7	44808.11
ILEUSYN-PWY: L-isoleucine biosynthesis I (fr	28698.2	30353.93	31370.75	24386.4	42144.16	35984.13	37465.72	21952.42	50669.82	25130.94	70937.41	91332.88
PWY-6151: S-adenosyl-L-methionine salvage	28687.55	27228.89	27137.29	17675.95	34513.28	30654.87	28356.46	23619.53	35118.33	25569.07	48904.41	83323.96
PWY-7663: gondoate biosynthesis (anaerobic	27787.69	30800.42	28526.98	26491.86	40638.74	31041.16	32941.08	8747.279	49182.72	13975.87	112219.8	137168.4
THRESYN-PWY: superpathway of L-threonin	27643.03	30567.46	29816.64	25539.11	43497.82	32901.04	35581.36	9428.799	45415.59	17898.91	68093.71	76400.88
RIBOSYN2-PWY: flavin biosynthesis I (bacter	27362.62	28963.99	28227.83	21309.46	24146.92	22025.88	28317.45	12839.01	29450.44	13438.07	18062.23	31694.66
PWY-7228: superpathway of guanosine nucle	27347.51	33126.43	29790.48	27859.63	25063.77	26344.29	33630.26	15600.86	47309.27	15190.17	53375.84	61324.14
BRANCHED-CHAIN-AA-SYN-PWY: superpat	27309.24	28679.9	29875.06	23558.21	41070.69	34801.91	34679.81	20298.39	47119.07	22886.21	64865.29	83679.03
PWY-724: superpathway of L-lysine, L-threon	26632.74	28207.91	27290.86	21001.91	28354.58	25409.46	33039.19	19034.17	38371.16	24800.06	45796.85	54511.62
PWY-6703: preQ0 biosynthesis	26631.83	26359.94	28523.05	23316.71	38380.45	31249.99	27092.79	5164.15	31129.74	5930.758	30307.1	41686.41
PWY-841: superpathway of purine nucleotide	26205.52	29787.71	29100.05	20905.73	21122.83	25894.2	32682.45	16191.64	41554.61	15294.46	49345.92	59641.61
ASPASN-PWY: superpathway of L-aspartate	25836.29	30630.13	23305.87	24870.32	39330.8	28343.76	34540.9	4960.546	38055.17	14782.2	90098.19	117888.2
PWY-6125: superpathway of guanosine nucle	25449.48	31159.59	27909.63	26570.58	22291.44	24003.88	31060.2	13545.99	44216.63	13087.92	49971.25	56705.15
PYRIDNUCSYN-PWY: NAD de novo biosynth	24841.64	26576.62	27584.82	22210.36	25870.41	25278.13	30466.66	14472.89	34309.39	14630.82	55499.93	58968.3
PWY-5103: L-isoleucine biosynthesis III	24779.71	25932.02	27115.06	21527.72	40491.04	33190.03	31049.82	18641.88	42482.87	20618.51	60136.92	77892.2
SER-GLYSYN-PWY: superpathway of L-serir	24361.19	25926.68	23246.09	22109.23	31835.26	29712.87	31457.01	20410.83	41822.55	25421.56	57207.96	76343.43
CALVIN-PWY: Calvin-Benson-Bassham cycle	23061.75	29624.3	28108.74	23606.96	26215.47	26722.1	44269.87	38664.02	62793.73	44849.45	86875.18	103003.8
ARGININE-SYN4-PWY: L-ornithine biosynthe	22876.85	22757.46	21639.89	19804.97	35356.17	25664.2	20513.48	1390.345	23758.24	2094.464	48099.79	53314.51
GLYCOGENSYNTH-PWY: glycogen biosynth	22740.31	23829.44	28282.09	14991	14662.39	19355.92	27245.15	26601.08	44804.45	30792.92	56605.76	74928.25
PWY-7220: adenosine deoxyribonucleotides (	22329.88	28952.9	23973.98	26566.73	18126.03	20415.47	28786.54	10458.34	42489.7	10095.8	50783.32	55886.22
PWY-7222: guanosine deoxyribonucleotides (	22329.88	28952.9	23973.98	26566.73	18126.03	20415.47	28786.54	10458.34	42489.7	10095.8	50783.32	55886.22

PWY-6270: isoprene biosynthesis I	21932.22	23713	28362.37	21144.13	29121.63	21059.45	29752.98	14533.62	38364.01	14749.76	27242.71	30931.92
PWY-6147: 6-hydroxymethyl-dihydropterin dip	21672.79	23455.29	22273.32	19655.54	24554.95	21996.75	23602.66	4876.854	24175.69	5587.183	37120.36	41610.08
RHAMCAT-PWY: L-rhamnose degradation I	21405.55	18300.06	24737.52	18098.39	14578.33	18158.79	22989.45	12089.19	27583.59	12652.9	49515.44	66862.56
PWY-7560: methylerythritol phosphate pathwa	21063.69	22984.02	27845.02	21115.57	28427.67	20052.63	30307.79	14317.94	40297.73	14742.55	45668.52	51241.95
PWY-3001: superpathway of L-isoleucine bios	20866.64	23785.31	21986.61	14823.29	19964.11	22912.12	30981.46	12216.57	43647.92	20148.21	68728.03	78901.07
PWY-5941: glycogen degradation II	20728.34	22592.02	26186.77	17198.27	29411.45	22237.78	31542.28	26651.04	45172.27	31793.47	79144.19	102400.6
PWY-6897: thiamine diphosphate salvage II	20646.87	20327.85	21075.99	11908.06	15698.6	19149	21944.37	10470.4	22666.38	12523.92	39141.82	54570.96
PHOSLIPSYN-PWY: superpathway of phosph	20622.12	20616.66	20967.54	17371	21734.83	18205.36	36197.54	10733.86	45861.04	13708.6	93259.78	125536.5
HISDEG-PWY: L-histidine degradation I	20326.22	22540.88	21071.31	18915.42	34671.21	22851.73	12768.25	8128.551	9396.771	9348.037	254.3206	3210.658
PWY0-1296: purine ribonucleosides degradat	19963.35	19294.02	24576.17	11059.92	10016.25	19413.26	36741.45	37629.5	55031.93	39514.36	60614.36	76791.3
ARGSYN-PWY: L-arginine biosynthesis I (via	19696.76	20531.92	23405.45	11079.64	10605.47	18308.96	27141.49	24236.88	43176.83	28050.19	62512.72	73823.02
GLYCOLYSIS: glycolysis I (from glucose 6-ph	19464.51	19450.12	14780.17	20889.5	25418.83	21917.85	35357.88	11877.49	44911.77	17127.59	74333.67	89443.06
PWY-5030: L-histidine degradation III	19445.36	19835.69	18477.8	15611.58	31795.99	20479.26	11689.64	1739.117	8570.878	0	13.19672	20.3064
PWY-5121: superpathway of geranylgeranyl d	19345.45	20356.2	17772.77	16822.66	10032.5	13423.28	20167.24	777.8516	21725.62	4434.228	4541.362	11965.98
PWY-5484: glycolysis II (from fructose 6-phos	19341.25	19255.8	14574.56	20674.76	25351.82	21502.95	33302.36	10320.81	41299.52	14728.95	66875.23	80298.13
CITRULBIO-PWY: L-citrulline biosynthesis	19311.45	20596.3	15612.99	13538.68	13328.17	19144.27	21025.61	1291.162	28060.43	3057.804	58365.97	61407.06
PWY-7357: thiamine phosphate formation fro	19224.02	17603.99	20098	9153.951	11668.25	16574.28	20065.28	16741.67	26032.13	15476.23	35399.89	47863.91
PWY-7400: L-arginine biosynthesis IV (archae	19112.82	19946.8	20047.79	10867.6	10478.06	18110.18	26625.1	23531.24	43065.65	27734.11	60428.86	67681.36
PWY-5154: L-arginine biosynthesis III (via N-a	18876.01	20076.03	21857.28	12126.72	14529.55	20077.84	25290.5	17390.94	41347.41	22583.36	57810.9	66182.95
ARGSYNBSUB-PWY: L-arginine biosynthesis	18679.19	17499.62	22641.69	10424.71	9339.616	17518	24678.14	23382.55	42816.82	27077.14	61854.52	73459.2
PWY-7237: myo-, chiro- and scyllo-inositol de	18402.82	17451.56	23764.62	0	12165.03	20073.01	25483.36	14731.67	30674.29	14503.68	0	31641.22
PWY-7282: 4-amino-2-methyl-5-diphosphome	18382.74	18202.33	18599.2	10705.65	14031.09	19873.75	23222.75	5150.638	25405.48	11347.71	52316.3	68832.84
PWY-7208: superpathway of pyrimidine nucle	17969.27	22644.36	20967.61	11496.86	14049.69	18050.01	21414.75	11426.69	35952.25	11131.79	38545.46	43144.43
THISYNARA-PWY: superpathway of thiamine	17929.75	16661.27	18026.72	8283.365	12376.25	17373.22	18488.26	10646.21	16463.66	9238.118	290.0643	8.578068
ANAEROFRUCAT-PWY: homolactic fermenta	17906.67	17177.3	15806.15	15188.68	19270.08	15287.85	25585.18	11856.62	37026.93	15993.36	50165.75	63927.72
PWY0-845: superpathway of pyridoxal 5 -pho	17088.54	18334.63	17253.41	17603.29	24124.87	24194.22	25120.13	2005.789	23671.34	6124.182	52645.61	66937.2
PWY-8187: L-arginine degradation XIII (reduc	16907.03	18491.05	12628.7	10907.53	6388.135	9285.624	18681.21	4420.336	23695.09	5980.096	49481.93	53745.82
PWY-8178: pentose phosphate pathway (non	16802.41	23300.62	22248.55	19248.39	19776.58	21713.72	38400.58	46023.74	58426.08	49578.37	127333	149877.5
GLUTORN-PWY: L-ornithine biosynthesis I	16337.87	17160.26	20289.74	9786.848	11710.35	16430.25	25690.36	17598.99	39119.89	19565.28	61415.07	73395.94
PWY-6901: superpathway of glucose and xylo	15875.13	18352.09	19322.44	16626.82	16728.19	16396.57	28768.1	16867.09	38941.4	21853.67	63692.33	92103.2
PWY-1269: CMP-3-deoxy-D-manno-octuloso	15393.7	13688.68	13980.22	14363.67	15513.31	16394.66	24399.97	5161.08	32755.8	6215.093	73570.94	86168.79
PWY-5659: GDP-mannose biosynthesis	15391.41	15133.89	11318.23	11078.29	18647.09	18697.74	20411.35	11495.53	26718.34	13413.46	1867.245	30895
PYRIDOXSYN-PWY: pyridoxal 5 -phosphate	15347.34	16450.28	16412.24	16534.93	37500.28	25223.65	22332.52	1385.259	22317.81	4298.142	42648.53	53423.19
PENTOSE-P-PWY: pentose phosphate pathw	15337.93	21113.28	18368.69	19107.09	20091.46	20692.04	31813.67	27511.85	41903.64	31614.82	104670.8	126466.7
PWY4FS-7: phosphatidylglycerol biosynthesis	15159.4	14147.26	16055.93	11506.46	13465.23	12340.2	29214.91	8951.687	40239.91	12323.38	89561.28	118622.8

PWY4FS-8: phosphatidylglycerol biosynthesis	15159.4	14141.16	16055.21	11506.46	13464.32	12339.48	29214.91	8951.687	40239.91	12322.77	89561.28	118622.5
PWY-7392: taxadiene biosynthesis (engineer	15107.04	16731.34	15094.44	15507.83	7614.118	11602.1	17289.42	284.7671	13109.64	2564.002	1230.918	5786.494
OANTIGEN-PWY: O-antigen building blocks l	14752.76	18753.41	15099.37	11122.38	12547.85	19188.57	24669.73	19304.41	35834.61	18846.68	34131.55	15732
NONOXIPENT-PWY: pentose phosphate pat	14297.79	20615.91	19520.53	18625.25	17695.28	20483.57	44553.44	43342.35	68223.12	44908.41	162560.8	186417
PWY-4984: urea cycle	13729.13	15409.41	11449.61	10829.52	9930.477	16610.68	7254.835	1086.371	5382.222	591.2103	377.7763	0
NAGLIPASYN-PWY: lipid IVA biosynthesis (E	13438.14	12209.23	7704.658	13024.82	23478.9	18714.67	20183.54	1738.162	28238.62	5718.005	51299.56	60370.34
PWY-8073: lipid IVA biosynthesis (P. putida)	13438.14	12209.23	7704.658	13024.82	23478.9	18714.67	20183.54	1738.162	28238.62	5718.005	51299.56	60370.34
PWY-621: sucrose degradation III (sucrose in	12591.97	13593.45	14438.98	11420.52	8388.811	7714.682	22120.55	12462.6	29868.85	16923	49027.83	42782.91
PWY-6902: chitin degradation II (Vibrio)	12289.24	9898.697	8811.848	10317.55	15715.05	13549.32	1438.006	550.0515	2636.429	3549.605	34883.15	51912.46
PWY-6317: D-galactose degradation I (Leloir	12263.33	14196.28	18103.34	7955.698	6022.505	13648.96	23750.82	22480.09	31610.75	23246.04	64034.15	78794.75
PWY-2941: L-lysine biosynthesis II	12238.43	15704.71	15623.2	15943.05	25322.25	22001.4	13641.08	17491.76	15422.85	15534.32	100.9189	1859.283
PWY-7111: pyruvate fermentation to isobutan	11589.53	20681.3	16904.15	19403.13	20286.72	20734.87	42044.61	28686.97	64979.87	34561.27	98128.36	121647.6
HSERMETANA-PWY: L-methionine biosynthe	11561.9	19276.08	14172.81	14200.48	19774.35	13697.82	25111.21	14093.9	21986.47	15085.84	568.818	539.9102
POLYISOPRENSYN-PWY: polyisoprenoid bi	11075.84	12808	11597.32	13198.14	7359.683	8860.929	15498.47	2823.875	21366.26	6067.261	46940.18	50663.46
COLANSYN-PWY: colanic acid building block	11066.5	14747.71	11760.93	12037.42	14289.85	14893.98	21988.89	13314.05	28310.87	12821.88	2021.694	24622.31
PWY-6731: starch degradation III	10517.89	12386.89	9438.658	9315.48	18216.51	12323.3	17454.12	7760.814	21001.63	5874.761	47450.93	53407
GLYCOCAT-PWY: glycogen degradation I	10399.59	7760.504	4370.169	1990.579	6427.742	11400.65	6170.962	1440.454	9700.5	4952.43	23050.09	45654.37
COBALSYN-PWY: superpathway of adenosyl	10378.68	9952.177	10448.16	9670.84	15320.9	13910.4	13692.35	8111.072	13391.2	8842.322	65982.81	64615.43
BIOTIN-BIOSYNTHESIS-PWY: biotin biosynt	10102.17	16440.63	13753.96	16373.68	16368.97	16186.81	29573.81	3598.898	30077.81	7713.959	49728.65	62090.88
FASYN-ELONG-PWY: fatty acid elongation --	9947.333	16784.41	14121.85	16880.4	14899.17	15089.51	31684.48	3720.055	34507.04	11588.24	53303.31	65692.72
PWY-7345: superpathway of anaerobic sucro	9520.883	16084.38	12169.65	15787.84	16805.84	14005.79	27998.3	6292.569	40513.56	13870.72	59870.29	79071.93
PWY-6519: 8-amino-7-oxononanoate biosynt	9316.983	15643.67	13148.72	15728.73	15064.88	15191.8	29577.99	3201.248	29362.44	7272.736	49155.22	61312.63
PWY-5384: sucrose degradation IV (sucrose	9312.316	12462.08	12711.42	11199.74	8432.894	6972.97	19246.94	13102.64	28374.09	18291.74	55742.76	73785.88
PWY-6527: stachyose degradation	9238.344	11924.08	15503.3	8124.191	6265.316	10650.82	25394.69	21891.76	39049.76	19033.48	65203.87	74555.68
PWY-7664: oleate biosynthesis IV (anaerobic	9106.16	15759.98	13119.98	15941.12	13612.09	14014.2	31131.52	3433.642	32771.97	11235.17	52077.07	63616.9
GLUCONEO-PWY: gluconeogenesis I	9073.772	17935.29	13856.43	17369.2	25498.51	18170.79	29446.5	6539.793	36114.06	15275.74	61802.05	75113.42
UDPNAGSYN-PWY: UDP-N-acetyl-D-glucos	8992.15	12140.53	9394.153	6942.146	7442.777	13866.42	21793.07	15752.52	42227.9	14955.8	63435.88	83692.73
PWY-7323: superpathway of GDP-mannose-	8973.942	11296.04	10550.32	10526.26	11347.18	8956.473	19748.66	7452.25	22453.27	6545.315	0	2029.674
PWY0-162: superpathway of pyrimidine ribon	8960.812	18149.92	11118.01	17854.27	20639.77	11938.34	28847.92	2691.79	31571.84	10605.7	34540.6	43948.79
PWY0-1261: anhydromuropeptides recycling	8899.196	11014.1	6697.129	4455.155	5759.783	13749.72	16302.05	8231.566	24781.95	7340.05	34225.47	45973.19
PWY-6936: seleno-amino acid biosynthesis (	8862.349	16920.3	11567.96	11735.21	16635.61	11042.06	25190.14	13211.62	41121.79	17825.58	71764.17	88812.03
P41-PWY: pyruvate fermentation to acetate a	8605.045	10505.14	12693.92	3413.883	2476.513	7160.659	9090.983	12567.61	17347.02	18409.73	3050.757	9230.119
PWY-5100: pyruvate fermentation to acetate	8605.045	10505.14	12693.92	5153.266	4634.102	7160.659	13081.02	12812.07	20440.39	18409.73	33548.72	40932.25
PWY-5989: stearate biosynthesis II (bacteria	8210.431	14627.36	11896.08	15134.78	12124.47	12629.38	29208.57	2975.815	28216.15	9971.164	41737.98	51306.55
PWY0-862: (5Z)-dodecenoate biosynthesis I	8183.472	14573.96	11986.18	14840.11	12206.17	12798.16	30423.57	3113.976	30712.91	10796.57	58272.29	69127.44

PWY-5188: tetrapyrrole biosynthesis I (from g	8155.568	7763.042	8425.323	5280.874	6126.944	9849.11	15132.21	6046.402	28712.44	9189.04	56552.52	67363.16
PWY-6282: palmitoleate biosynthesis I (from	8154.131	14482.34	11910.78	14954.26	12269.84	12778.16	30163.37	3033.695	30757.8	10418.58	45106.52	55544.69
PWY-6859: all-trans-farnesol biosynthesis	8044.617	9445.605	8449.47	10469.52	4359.318	6435.49	11347.11	289.5498	11699.45	1864.085	1808.65	5440.358
PWY-7328: superpathway of UDP-glucose-de	7819.664	12159.83	8117.609	10033.35	9906.239	11010.82	18715.2	20731.82	23788.05	20282.59	50.19118	16777.57
GLUCOSE1PMETAB-PWY: glucose and gluc	7528.647	10835.33	11171.72	11157.1	11537.81	7613.283	17825.41	19961.45	25135.09	21032.17	54697.87	82113.03
PWY-6906: chitin derivatives degradation	7504.508	9110.324	5823.835	355.3429	12549.83	16092.16	2638.276	1296.824	2500.937	1001.52	0	0
PWY-6823: molybdopterin biosynthesis	7403.132	8835.246	7566.941	3699.777	2614.866	8714.546	11609.84	14745.98	17691.45	16088.78	29969.26	32746.38
PWY-7456: β-(1,4)-mannan degradation	7382.851	9355.127	7127.886	7891.143	8438.62	7156.411	14535.27	2751.186	10390.35	837.2547	0	0
FUC-RHAMCAT-PWY: superpathway of fuco	7315.17	7199.638	8806.036	5844.59	4707.446	9436.901	19038.87	2887.166	25892.41	6886.007	49774.36	64039.84
PWY-7197: pyrimidine deoxyribonucleotide pl	7042.824	11844.77	9212.039	5696.688	4910.696	11049.73	13679.95	8086.378	23880.81	7506.738	30075.73	35385.44
PWY-6628: superpathway of L-phenylalanine	6951.925	11516.06	8596.245	3844.352	3851.773	13769.56	16658.44	13357.52	24131.65	13708.57	31846.86	50080.8
GLYCOLYSIS-E-D: superpathway of glycolysi	6654.493	14084.36	9359.585	13351.79	16092.21	10005.08	21765.09	4137.881	28637.74	11864.51	58909.26	75160.99
PWY-6608: guanosine nucleotides degradatic	6331.58	8072.763	10697.68	12813.26	15997.16	9093.364	42528.25	14160.56	62503.95	18029.18	89510.87	115178
GLUCUROCAT-PWY: superpathway of &beta	6301.694	6774.226	8613.477	6615.395	11440.21	8827.703	14959.28	4292.834	17055.94	8417.019	33601.31	51493.36
PWY-5971: palmitate biosynthesis (type II fatt	6054.164	15266.7	7288.696	16664.49	15368.52	14456.31	31119.61	3856.262	33547.66	10505.83	51341.78	63316.49
PWY-7184: pyrimidine deoxyribonucleotides c	5841.196	3341.615	9476.953	5770.88	4440.886	6299.057	16833.4	5480.117	26778.96	8516.322	37449.16	48380.12
PWY-7210: pyrimidine deoxyribonucleotides b	5591.814	3531.119	8825.97	992.8898	447.5999	2663.175	12440.62	2003.426	17444.59	7038.932	3073.171	6100.304
PWY-8004: Entner-Doudoroff pathway I	5557.623	13237.51	8555.152	12957.41	15273.62	8624.6	19396.43	3394.137	23894.24	12302.33	56717.49	70952.14
PWY-6545: pyrimidine deoxyribonucleotides c	5520.012	3125.16	8720.5	1564.664	2322.994	5477.988	12260.6	4931.542	23085.73	6474.166	2788.882	5605.858
PWY-7198: pyrimidine deoxyribonucleotides c	5098.185	6766.91	8009.708	933.8362	597.1274	1993.049	11226.62	10533.69	14909.24	10407.99	2195.856	4446.182
PWY-7242: D-fructuronate degradation	4706.014	5284.887	6638.197	5548.432	10269	6952.005	13355.91	3205.791	15272.63	9779.357	30687.64	56793.43
GLCMANNANAUT-PWY: superpathway of N-	4614.147	9483.548	7286.869	10569.94	7967.142	10651.96	29640.89	17998.01	36375.55	15142.97	63517.18	73264.96
PWY-6305: superpathway of putrescine biosy	4611.503	6677.583	8190.991	10441.37	11586.51	12132.84	12830.94	1589.623	18704.44	7682.741	61322.56	93495.52
PWY-6353: purine nucleotides degradation II	4590.976	9526.136	4359.287	12126.94	11015.85	11081.77	27228.32	14454.3	42059.22	19553.39	80267.47	98096.35
PWY-6168: flavin biosynthesis III (fungi)	4352.019	8989.307	7311.692	15636.63	10482.5	16798.99	16314.7	2514.858	22623.47	6525.919	5321.44	26786.31
PWY0-1479: tRNA processing	4346.99	5519.788	5912.139	6626.125	7096.792	6442.499	18971.38	1896.546	28320.32	8705.351	40807.3	47539.85
PWY66-399: gluconeogenesis III	4203.683	5114.102	4212.338	1078.268	583.0247	1469.508	6099.613	1626.544	5050.118	5375.58	1096.546	0
FUCCAT-PWY: fucose degradation	4124.715	4324.611	5093.073	3557.422	2597.22	5940.294	17093.07	1481.611	24945.69	4386.661	47406.83	61450.54
PWY0-1241: ADP-L-glycero-β-D-manno	4104.34	5714.003	3659.791	1911.23	4794.478	7266.845	6352.765	2835.737	18068.58	3919.419	28079.22	34873.71
PWY-7234: inosine-5 -phosphate biosynthesis	4094.547	10236.97	7249.385	8799.981	9451.24	7642.715	24144.58	6216.237	28903.86	9669.209	31432.62	39306.63
PWY-I9: L-cysteine biosynthesis VI (from L-m	3934.988	8253.476	8259.274	9714.729	11665.38	7559.172	24765.51	8697.87	30206.89	14809.81	39323.11	56534.45
P164-PWY: purine nucleobases degradation	3511.688	5227.212	7460.564	5221.136	5419.877	7016.408	7650.844	15409.77	18252.16	17815.59	191.004	2696.08
PWY-6470: peptidoglycan biosynthesis V (&b	3481.724	9390.506	4367.272	2038.121	3730.678	3966.197	3143.495	5724.534	3644.063	1589.868	0	1387.954
PWY-7211: superpathway of pyrimidine deox	3468.74	3970.159	5469.442	1688.833	782.7419	3897.454	16945.81	2502.545	22248.61	8846.99	5185.021	10035.01
PWY0-1477: ethanolamine utilization	3358.349	5756.187	6492.615	6311.475	5775.343	6535.721	25167.33	16165.54	46949.11	22216.27	104471.4	129509.8

PWY-702: L-methionine biosynthesis II	3158.495	6299.186	5353.165	4716.919	5277.177	4827.569	15937.83	5813.361	27790.19	10338.21	65037.48	64760.41
GALACT-GLUCUROCAT-PWY: superpathwa	3152.94	4722.014	4290.465	6910.663	8395.76	6802.009	12227.41	3358.635	16923.13	5840.267	29033.76	39170.39
PWY-6630: superpathway of L-tyrosine biosy	3132.579	6366.793	5381.767	2974.361	2037.584	4483.705	9422.791	12280.38	17824.68	13624.49	19390.27	26137.87
PWY-6292: superpathway of L-cysteine biosy	2974.093	3205.84	4471.884	30.59035	93.34084	237.13	3249.486	232.5332	0	3079.385	114.5396	3961.124
GALACTUROCAT-PWY: D-galacturonate de	2952.91	4918.694	4264.656	7934.842	11124.33	8232.934	14231.31	6739.279	16306.44	9598.674	33815.3	56179.47
DAPLYSINESYN-PWY: L-lysine biosynthesis	2927.172	8747.064	5929.825	9859.787	10806.78	7519.415	19862.13	1602.357	29846.1	12442.22	37743.67	57877.22
PWY-6590: superpathway of Clostridium acet	2888.393	3104.012	1734.165	1050.262	743.7644	1290.473	1014.115	3769.91	1847.336	1161.251	0	0
PWY-4041: γ-glutamyl cycle	2828.976	6337.247	5737.907	9798.144	9153.472	9396.421	24606.53	5748.71	29973.55	9228.279	79342.62	96224.97
PWY-5505: L-glutamate and L-glutamine bios	2816.174	4105.433	4184.808	795.2417	482.8011	1570.057	5566.336	1193.09	5362.261	1544.703	0	0
PWY0-1061: superpathway of L-alanine biosy	2786.182	3445.343	6184.609	3405.016	1754.62	2700.722	10499.12	4820.755	15677.47	6646.887	23254.25	53426.63
PWY-6507: 4-deoxy-L-threo-hex-4-enopyranu	2726.233	4245.505	5569.643	6829.35	10318.69	6201.792	13183.03	6242.155	15339.05	10284.53	31304.45	55835.28
P441-PWY: superpathway of N-acetylneuram	2664.764	8965.78	7252.953	11895.7	12434.22	11222.67	28340.92	11827.95	38730.01	15641	64326.93	78972.33
PWY66-409: superpathway of purine nucleoti	2601.092	10590.99	7506.231	9531.037	9141.391	6899.016	22897.76	15814.12	30277.15	19300.53	45634.84	53959.64
PWY-5676: acetyl-CoA fermentation to butan	2577.194	4565.789	1247.414	3696.529	2424.202	5347.883	7486.444	6377.814	9608.829	7217.091	52.61457	0
SALVADEHYPOX-PWY: adenosine nucleotid	2538.944	6184.543	2244.512	11450.46	14151.54	7869.051	40663.65	12822.53	61589.11	17222.71	99401.65	125058.9
PWY-6606: guanosine nucleotides degradatic	2511.714	5304.873	2443.607	6863.819	5275.416	6235.916	15603.56	10492.26	26946.42	13775.79	77949.77	89696.79
PWY-7383: anaerobic energy metabolism (in	2458.925	2540.122	2878.641	586.5501	334.8548	842.0193	4030.747	1200.75	3253.259	5124.4	1539.685	0
PWY-5497: purine nucleobases degradation I	2354.556	5314.817	4366.5	4288.862	9761.678	8616.213	8182.324	2185.344	16952.15	7469.197	34892.76	27688.14
PWY-6895: superpathway of thiamine diphos	2337.276	7797.46	5700.283	9783.393	11374.35	12428.78	24251.08	3030.829	24503.8	8104.037	35656.27	42551.16
CENTFERM-PWY: pyruvate fermentation to b	2331.863	2427.675	1341.172	799.5543	560.7136	992.8029	767.183	3052.932	1404.025	884.1143	0	0
FERMENTATION-PWY: mixed acid fermenta	2190.416	4784.02	6611.494	7987.02	8044.278	9562.253	15805.72	1281.206	21527.8	9261.341	37779.39	73408.54
GLUDEG-I-PWY: GABA shunt	2038.036	3604.868	3933.28	5490.435	6343.413	2544.4	16442.34	1131.258	10923.21	961.5587	15843.65	19838.57
PWY-5022: 4-aminobutanoate degradation V	2032.798	3560.61	3864.943	5313.257	6382.709	2541.321	15379.99	2978.679	11769.14	3135.38	0	0
PWY-5005: biotin biosynthesis II	1915.85	1929.925	1651.374	352.5114	1115.796	5865.57	2422.613	2501.745	14091.16	846.1734	574.4051	0
ARG+POLYAMINE-SYN: superpathway of ar	1813.359	6793.669	7641.529	8242.115	7407.194	11322.09	21231.27	6424.117	34870.18	16832.22	60851.97	70993.48
PWY-6969: TCA cycle V (2-oxoglutarate synt	1809.436	4733.79	4244.45	5196.977	4695.078	6215.164	10729.96	678.2487	14880.53	5880.155	26335.07	54102.15
PWY-7356: thiamine diphosphate salvage IV	1697.755	3425.244	3031.759	119.9089	214.4275	475.4966	1110.844	1209.678	2239.908	4736.754	17.34893	10183.48
P42-PWY: incomplete reductive TCA cycle	1690.498	4283.292	1670.975	2463.157	2209.572	2353.885	3768.214	302.7727	6120.45	3093.574	828.5411	525.1456
PWY-5136: fatty acid β-oxidation II (plan	1674.748	5080.033	3460.689	7689.384	9952.297	8767.208	25105.39	2766.256	40181.73	9069.36	132521.4	150568.9
PWY-7883: anhydromuropeptides recycling II	1665.122	3353.806	2533.175	1414.082	2355.474	2508.849	6054.786	368.0208	10328.01	3585.219	10391.08	12986.3
PWY-8131: 5 -deoxyadenosine degradation II	1639.601	3698.653	2143.359	1187.38	349.7165	4327.373	1242.519	16293.54	5065.844	17694.86	22459.83	9901.255
PWY-7115: C4 photosynthetic carbon assimil	1614.89	3636.513	5072.104	2367.628	1979.487	3135.037	11858.88	2666.012	16117.23	6422.028	25276.39	48387.78
PWY-5838: superpathway of menaquinol-8 bi	1603.4	8726.819	9568.464	8758.734	7993.437	8373.621	19039.15	1725.624	22550.33	4637.362	52483.33	56799.27
PWY-7117: C4 photosynthetic carbon assimil	1593.924	7329.72	4662.38	8702.536	9496.951	6624.333	20766.58	1619.537	30486.08	9306.877	76886.58	98301.62
HEMESYN2-PWY: heme b biosynthesis II (ox	1513.859	3995.307	2256.726	3126.268	3876.3	4129.462	14596.64	3889.658	22065.73	7476.519	42303.7	52761.05

PWY-5845: superpathway of menaquinol-9 bi	1423.779	7517.572	5175.848	8295.836	7997.105	8190.49	14666.3	1307.837	18261.46	4380.552	47124.93	53762.72
PWY-5897: superpathway of menaquinol-11 b	1415.564	8028.188	9043.756	8105.206	7218.733	7603.612	18497.92	1808.895	22007.49	4701.566	50488.53	56018.74
PWY-5898: superpathway of menaquinol-12 b	1415.564	8028.188	9043.756	8105.206	7218.733	7603.612	18497.92	1808.895	22007.49	4701.566	50488.53	56018.74
PWY-5899: superpathway of menaquinol-13 b	1415.564	8028.188	9043.756	8105.206	7218.733	7603.612	18497.92	1808.895	22007.49	4701.566	50488.53	56018.74
PWY-6992: 1,5-anhydrofructose degradation	1377.945	4889.925	840.9259	5337.675	5707.53	1845.599	5276.173	35.79685	5928.651	381.1948	16499.34	20858.27
PWY-7013: (S)-propane-1,2-diol degradation	1359.212	1316.656	4774.276	4384.383	3853.428	881.5941	267.5935	101.7664	0	12099.33	0	24663.42
PWY-241: C4 photosynthetic carbon assimila	1330.597	5967.811	3998.695	6734.325	8421.175	5709.669	16537.78	1575.794	19681.79	8856.608	98.35489	257.3362
PWY-6549: L-glutamine biosynthesis III	1316.875	3325.111	3239.476	1344.843	869.3633	2475.482	5609.552	1973.122	7791.285	7647.256	0	0
PWY-5840: superpathway of menaquinol-7 bi	1278.173	3962.57	5175.848	4909.913	4302.13	5644.599	14666.3	1307.837	18261.46	4380.552	47124.93	53762.72
PWY0-1297: superpathway of purine deoxyrib	1271.627	5148.002	4894.694	6953.944	7172.872	8274.695	27603.81	14705.93	42492.19	25908.7	48617.21	64704.73
PWY-5690: TCA cycle II (plants and fungi)	1192.227	2315.56	1888.476	477.409	224.6043	169.9868	602.3524	659.2387	730.4059	1555.47	0	0
PWY66-391: fatty acid β-oxidation VI (m	1169.238	1636.092	189.8142	1177.52	415.3207	1955.691	1849.232	931.3689	1745.719	697.2608	177.8706	513.2662
METH-ACETATE-PWY: methanogenesis fror	1110.502	860.8157	1109.79	95.27877	308.6162	784.0494	905.2196	4905.295	3066.651	0	0	0
PWY-5861: superpathway of demethylmenaq	1096.737	6634.919	7530.31	6613.462	5737.047	6172.492	16224.23	1666.825	20133.14	4248.744	51025.25	53908.31
PWY-5345: superpathway of L-methionine bic	1085.019	9823.949	10367.65	12928.93	14289.46	9598.795	19470.19	4663.802	25580.62	9495.284	2039.715	1946.303
PWY-7761: NAD salvage pathway II (PNC IV	1075.603	5621.65	3389.179	4420.935	5032.834	7460.471	10312.69	10234.17	17620.16	9198.228	19039.37	13160.54
PWY4LZ-257: superpathway of fermentation	1030.734	4183.678	3789.692	5990.725	5211.153	6203.966	17788.99	8652.881	28996.1	10116.48	56191.78	71139.42
PWY-5862: superpathway of demethylmenaq	971.0453	5606.44	3761.671	6220.347	5739.881	6023.575	11747.24	1139.44	15315.88	3931.953	43767.67	49896.22
POLYAMSYN-PWY: superpathway of polyam	951.6606	4090.491	4707.9	6630.812	5724.364	8218.747	17619.14	3716.328	29246.03	12053.28	60807.59	74803.37
PWY-6807: xyloglucan degradation II (exoglu	940.2578	775.3045	1165.957	0	0	0	0	0	123.598	0	0	0
PWY-622: starch biosynthesis	913.4584	1902.801	417.0152	0	0	0	0	0	0	0	0	0
SULFATE-CYS-PWY: superpathway of sulfat	891.1226	8304.838	9056.338	12050.35	13726.98	8905.006	26447.58	4324.627	33687.69	7856.532	60675.38	73869.87
HEME-BIOSYNTHESIS-II: heme b biosynthes	882.6747	3497.462	1348.549	4949.692	5190.287	3136.488	12398.59	837.3552	19512.58	4473.308	36339.65	45236.31
HEXITOLDEGSUPER-PWY: superpathway o	840.2646	2870.514	432.6503	6459.773	6962.249	6517.753	17967.28	3881.941	29161.62	4457.316	51078.85	29164.15
P161-PWY: acetylene degradation (anaerobic	817.9038	3467.341	3070.841	5549.595	5775.343	5576.225	23185.76	10890.86	37944.47	16726.75	60524.69	92850.22
PWY0-1298: superpathway of pyrimidine deo	796.687	3803.214	3552.208	5452.969	5462.148	5898.819	22898.43	12738.62	35831.89	17093.86	45795.86	59326.75
GOLPDLCAT-PWY: superpathway of glycero	788.036	1116.482	2194.813	1011.875	137.8457	1428.05	315.2455	1884.785	3453.618	5547.66	12.96219	8678.147
PWY-6572: chondroitin sulfate degradation I (	748.7331	927.0338	1945.104	2153.709	2490.825	1088.243	282.2276	194.4527	700.0893	0	0	0
PWY-5913: partial TCA cycle (obligate autotro	748.5829	3343.063	2563.064	5650.497	5589.082	3748.81	16203.22	925.416	25836.06	7535.422	86050.79	96358.07
PWY-5918: superpathway of heme b biosynth	726.8857	3291.773	1868	4128.629	4993.227	3650.257	12808.59	1240.052	22154.76	4888.817	45920.74	55385.4
PWY-1861: formaldehyde assimilation II (assi	716.1515	1631.009	2755.104	637.0054	77.94249	2936.361	1932.055	4696.653	8261.262	10593.73	0	3648.873
PWY-6595: superpathway of guanosine nucle	700.9965	2390.481	842.312	498.1847	243.3196	322.5721	407.6752	9111.592	1547.934	10662.74	19.51644	567.2018
PWY-6607: guanosine nucleotides degradatic	697.6592	2476.86	817.6438	504.0699	245.8944	324.3697	410.0156	9084.841	1565.477	10853.08	19.51723	567.7567
P461-PWY: hexitol fermentation to lactate, for	697.5638	3816.045	3016.733	7530.991	8533.906	8356.856	15454.36	5177.833	28426.41	9029.12	91619.8	122588.1
PWY-5130: 2-oxobutanoate degradation I	689.8597	2219.773	1024.29	2849.864	305.7611	2120.723	1616.771	1285.405	589.5136	633.421	0	0

PWY-6285: superpathway of fatty acids biosy	671.2693	5486.364	1227.688	8414.865	7621.05	5770.51	21732.05	1965.509	26534.08	5195.393	45581.77	56500.91
P124-PWY: Bifidobacterium shunt	668.0308	2040.351	214.224	0	88.61258	721.28	0	0	0	187.192	0	0
PWY1ZNC-1: assimilatory sulfate reduction IV	667.2692	1818.86	2658.408	3902.563	3942.708	1790.286	10912.6	449.269	15917.2	4164.246	45219.32	52275.74
PWY0-781: aspartate superpathway	663.3378	4018.567	4474.947	5913.745	6250.548	4262.984	15120.57	1127.966	23261.62	10422.48	36465.45	59053.28
PWY-6531: mannitol cycle	645.8117	1785.636	1870.655	2838.673	2335.158	3764.995	9514.24	6041.872	17693.98	6037.273	59107.24	74524.53
P4-PWY: superpathway of L-lysine, L-threonin	629.1104	3848.74	4312.011	5736.024	6043.37	4112.384	14877.83	1069.528	22707.98	10074.34	45090.86	60431.04
TCA: TCA cycle I (prokaryotic)	609.9558	2938.12	4244.45	5196.977	4695.078	6215.164	10729.96	678.2487	15009.33	5880.155	27960.24	57747.37
PWY-5850: superpathway of menaquinol-6 bi	601.7126	3962.57	5175.848	4909.913	4302.13	5644.599	14666.3	1307.837	18261.46	4380.552	47124.93	53762.72
PWY-5896: superpathway of menaquinol-10 b	601.7126	3962.57	5175.848	4909.913	4302.13	5644.599	14666.3	1307.837	18261.46	4380.552	47124.93	53762.72
POLYAMINSYN3-PWY: superpathway of poly	598.6967	2120.987	2824.098	0	345.1837	244.6134	1282.234	0	0	216.159	0	0
GALACTARDEG-PWY: D-galactarate degrad	575.1295	2423.201	1719.448	6152.182	4620.726	5428.081	19184.19	6375.256	31944.84	14738.75	83419.75	117160
GLUCARGALACTSUPER-PWY: superpathwa	575.1295	2423.201	1719.448	6152.182	4620.726	5428.081	19184.19	6375.256	31944.84	14738.75	83419.75	117160
PWY-6588: pyruvate fermentation to acetone	567.2921	1696.834	702.6993	2582.678	2102.311	4412.825	2217.388	5533.713	4079.677	1882.493	356.9714	4643.3
LIPASYN-PWY: phospholipases	545.1416	1825.96	1272.222	2329.326	2479.826	0	6726.47	367.312	12762.34	3421.766	27588.14	41258.48
PWY-7315: dTDP-N-acetylthomosamine bios	524.9171	950.7635	1763.445	7639.805	1226.491	1030.105	9355.234	477.4707	13872.1	4564.057	25453.39	33819.08
PWY-5837: 2-carboxy-1,4-naphthoquinol bios	491.5623	3390.351	4179.241	3497.769	2751.685	3043.908	10549.75	1886.479	13721.99	4219.838	41915.36	45470.81
PPGPPMET-PWY: ppGpp metabolism	481.329	2272.871	1762.419	1006.999	1165.837	597.3591	5315.845	3157.456	10907.56	3616.496	13591.66	17116.57
PWY0-166: superpathway of pyrimidine deoxy	472.5363	3188.693	3719.895	5359.958	4224.482	6459.63	15461.93	5629.028	25009.56	8358.094	32593.38	44248.67
PWY-6478: GDP-D-glycero-α-D-manno	467.8034	731.8268	497.4363	2009.705	1527.987	773.2968	819.9426	79.18058	1174.59	0	0	0
FOLSYN-PWY: superpathway of tetrahydrofo	440.6484	6448.495	1151.413	2638.461	1835.084	3164.434	12502.07	4395.157	19623.65	7943.255	26592.03	42813
PWY-6284: superpathway of unsaturated fatty	421.3767	3197.453	636.8253	5586.034	5101.002	3465.227	16759.09	1242.555	21847.84	3283.904	42606.38	52937.5
FAO-PWY: fatty acid β-oxidation I (gene	420.789	2347.48	2719.281	4682.174	5143.763	4483.846	18285.24	1196.193	29655.62	7502.777	91610.8	90645.87
PWY-821: superpathway of sulfur amino acid	416.9492	3975.622	4852.936	6384.259	6726.825	3616.017	14108.51	1310.601	14388.18	6851.119	1105.881	1247.145
SO4ASSIM-PWY: assimilatory sulfate reducti	410.3583	4929.264	5599.796	8762.994	8660.923	5804.628	29233.69	2411.864	30524.9	5565.949	64542.68	74914.29
PWY-561: superpathway of glyoxylate cycle a	405.9263	2000.529	2760.549	5012.245	5024.488	4066.616	12490.58	802.821	18191.37	6081.187	35118.52	67213.14
PWY-5860: superpathway of demethylmenaq	404.9947	2798.173	3761.671	3503.197	2982.259	4022.269	11747.24	1139.44	15315.88	3931.953	43767.67	49896.22
GLUCARDEG-PWY: D-glucarate degradation	376.1939	1501.231	1305.296	3361.585	3076.903	2050.576	11882.4	1415.995	20215.08	12417.11	62405.59	77743.03
PWY-5723: Rubisco shunt	371.86	3209.329	2768.591	8556.118	6453.645	5187.403	23040.66	1602.996	38364.08	10166.98	68410.34	103448.9
PYRIDNUCSAL-PWY: NAD salvage pathway	355.3732	2458.01	1606.426	3487.131	3798.956	3337.926	10437.32	1276.006	18163.39	4848.874	364.9317	0
TCA-GLYOX-BYPASS: superpathway of glyo	350.1634	1788.158	2729.418	4755.752	4526.035	3650.056	11279.52	685.7	16368.98	5621.991	31731.41	62539.53
PWY-5347: superpathway of L-methionine bio	348.3784	3553.181	3671.195	8983.987	9825.049	4862.486	24031.6	2127.828	37822.01	10252.34	67281.92	87810.13
MET-SAM-PWY: superpathway of S-adenosy	347.1391	3499.384	3590.791	8515.22	9311.281	4695.831	22631.52	2299.113	37902.45	10872.99	64478	82830.5
HEME-BIOSYNTHESIS-II-1: heme b biosynth	344.4466	1862.456	1348.549	2934.415	3612.48	3136.488	12398.59	1409.831	19512.58	5192.477	37101.99	45236.31
ALLANTOINDEG-PWY: superpathway of alla	329.5012	96.47041	750.7185	36.65548	47.22878	17.81526	795.8169	470.5074	244.4553	1274	133.3009	73.69244
GALACTITOLCAT-PWY: galactitol degradatio	318.794	1083.764	129.6965	2824.034	2851.537	2829.154	12130.78	3269.932	20404.05	1780.532	50478.86	11417.8

PWY-5138: fatty acid β-oxidation IV (uns	308.6213	1217.663	1658.007	1997.476	2374.39	2258.27	8186.514	651.5502	13657.65	4815.785	32739.5	33553.42
PWY-7254: TCA cycle VII (acetate-producers	307.7764	2001.316	1041.707	208.1361	0	102.9111	42.26315	0	54.75078	0	0	0
GLYOXYLATE-BYPASS: glyoxylate cycle	307.2574	1471.444	1777.621	4044.505	4457.122	3628.964	13384.3	738.2373	20954.95	5018.081	46770.48	65049.54
METSYN-PWY: superpathway of L-homoseri	305.2462	3147.569	3258.84	8144.621	8833.635	4334.096	22898.46	2048.865	37655.27	10054.75	65603.19	84454.02
PWY-6612: superpathway of tetrahydrofolate	294.9275	4551.938	775.1863	1812.743	1237.365	2163.272	9407.962	3087.849	15108.08	5962.857	21422.48	39009.32
PWY-5920: superpathway of heme b biosynth	294.3228	1883.616	946.5685	3293.787	3597.449	2403.234	6148.793	315.3919	4829.512	2273.617	27329.7	28382.14
PWY3O-4107: NAD salvage pathway V (PNC	287.8282	2082.775	1379.523	3492.773	3737.454	2845.699	12506.4	952.3871	20075.58	4148.284	261.413	0
PWY-5675: nitrate reduction V (assimilatory)	284.7035	2099.57	1884.323	4750.517	4821.122	2795.202	21310.71	1091.601	32753.86	6931.987	70050.89	79098.95
PWY-6922: L-Nδ-acetylornithine biosynt	283.648	1276.604	1403.67	1651.748	1532.149	847.0639	482.6939	34.22542	1703.039	584.2638	452.2174	0
PWY-5677: succinate fermentation to butanoa	261.6202	544.2517	57.47408	203.5764	74.69009	650.3577	467.5062	1311.417	442.3986	667.8299	0	0
P108-PWY: pyruvate fermentation to propanc	258.4626	3277.097	2094.414	3209.891	3634.708	2027.227	3842.103	473.8089	3830.486	4279.344	472.5269	2210.311
RUMP-PWY: formaldehyde oxidation I	255.7891	787.6037	99.20932	245.169	0	36.14041	202.9247	2094.442	37.35418	3488.773	0	0
ORNDEG-PWY: superpathway of ornithine de	235.6712	1368.267	1216.798	4979.313	4118.036	2814.623	15098.84	837.0815	23939.7	4964.09	126275.6	145417.4
PWY-7200: superpathway of pyrimidine deoxy	227.4985	539.2295	427.6288	0	0	0	0	0	0	0	0	0
PWY-7118: chitin deacetylation	220.4601	1905.807	1541.456	3099.553	2502.95	2694.826	14472.07	1559.054	22598.42	6058.873	72326.3	104373.7
PWY-5104: L-isoleucine biosynthesis IV	213.6489	2301.933	672.0086	5246.604	4402.694	3722.538	12584.7	1425.841	13309.99	3394.816	0	0
PWY0-301: L-ascorbate degradation I (bacter	212.1654	1572.649	1488.8	2890.314	3870.16	2079.246	15899.54	935.2914	27820.36	4827.036	59160.55	72191.44
PWY-5189: tetrapyrrole biosynthesis II (from g	209.3278	1523.131	787.3834	2916.167	3008.877	2485.746	9711.068	883.171	17936.22	3559.167	51431.15	58866.47
PROPFERM-PWY: superpathway of L-alanine	208.5578	381.3639	56.44385	0	0	0	237.977	86.91934	512.5202	198.9731	0	0
PWY-8188: L-alanine degradation VI (reductiv	208.5578	381.3639	56.44385	0	0	0	237.977	86.91934	512.5202	198.9731	0	0
PWY-8189: L-alanine degradation V (oxidative	208.5578	381.3639	56.44385	0	0	0	237.977	86.91934	512.5202	198.9731	0	0
PWY-7209: superpathway of pyrimidine ribon	205.7194	235.1692	98.80182	25.7358	133.1201	0	874.7845	48.25259	1238.804	408.7333	16093.48	17388.22
PWY-6803: phosphatidylcholine acyl editing	199.7979	1608.612	2444.911	4339.595	4618.013	3499.62	10898.12	644.7079	16312.81	5328.814	36025.65	49950.77
METHGLYUT-PWY: superpathway of methylg	183.0398	1658.191	930.3531	1688.883	2177.118	1091.418	3595.898	149.8616	2807.859	2234.746	9096.564	13240.92
PWY-5656: mannosylglycerate biosynthesis I	180.3752	947.5502	20.57231	2898.061	2913.075	2400.993	11251.94	614.916	14631.77	897.7892	0	3243.795
PWY-7385: 1,3-propanediol biosynthesis (eng	176.883	1363.253	63.18598	2192.896	647.6225	2326.417	565.1852	150.7825	8587.456	2761.649	0	24945.91
KETOGLUCONMET-PWY: ketogluconate me	175.7047	1729.466	454.0529	4610.659	4596.671	5140.562	5968.22	646.6999	10185.45	1144.065	58300.9	67905.67
HOMOSER-METSYN-PWY: L-methionine bic	175.1887	1874.456	1944.045	5313.717	5479.204	2603.475	17111.66	1222.33	29091.03	6731.408	58625.23	74056.1
PWY-7434: terminal O-glycans residues mod	167.2372	380.9603	210.3492	10.32481	84.23577	186.1607	0	0	7.544507	9.785472	0	0
PWY-6876: isopropanol biosynthesis (enginee	164.5651	207.0797	0	516.4829	93.70169	459.8946	0	0	0	136.0086	0	0
P23-PWY: reductive TCA cycle I	155.0043	2338.979	1283.296	343.8277	0	174.8389	73.34289	0	95.16204	0	0	0
FASYN-INITIAL-PWY: superpathway of fatty a	151.7817	329.0536	75.07201	0	0	0	0	0	0	0	189.4029	0
PWY0-1415: superpathway of heme b biosynt	150.5151	1790.865	1426.067	2789.585	3147.874	2531.967	10209.61	1795.762	17278.42	5967.586	38880.51	46785.2
P105-PWY: TCA cycle IV (2-oxoglutarate dec	149.0161	1359.617	3200.723	2373.604	2445.82	2243.419	9716.257	601.3793	13671.72	5501.27	22763.12	50445.98
LACTOSECAT-PWY: lactose and galactose d	147.3821	2097.47	1054.996	237.9015	1056.054	2278.388	456.3843	8213.709	3328.794	1819.763	145.2954	1466.468

PWY-7992: superpathway of menaquinol-8 bi	142.7529	408.6581	151.2503	322.4093	0	4986.387	349.7531	0	142.037	0	0	0
PWY-7204: pyridoxal 5 -phosphate salvage II	140.4039	985.5752	44.75867	2932.794	2193.899	1908.976	10384.51	512.1297	13158.74	2046.698	38565.47	48042.42
PWY-6961: L-ascorbate degradation II (bacte	136.7682	1590.265	741.4077	3003.919	3917.078	2025.388	13366.62	1138.702	25648.69	5551.406	56224.32	66916.06
REDCITCYC: TCA cycle VI (Helicobacter)	133.5335	1499.185	1267.374	833.868	978.0001	285.8439	3108.374	253.6644	4954.911	3426.828	2645.127	10502.33
PWY-5265: peptidoglycan biosynthesis II (sta	131.7435	1897.04	384.723	366.3103	0	167.3989	66.24932	7799.074	1927.703	3674.107	0	960.3699
PWY-5367: petroselinate biosynthesis	129.9277	878.5955	135.5188	1897.564	1690.421	1036.535	7269.473	417.1415	11153.36	887.3802	28350.19	36691.64
PWY-5392: reductive TCA cycle II	125.4456	543.4784	894.8865	300.2466	0	139.0825	72.61253	0	93.44476	0	0	0
P185-PWY: formaldehyde assimilation III (dih	122.1989	317.9427	2172.263	83.31954	43.60948	149.4314	109.2764	2116.636	95.89668	5634.663	98.37203	10954.08
PWY0-1277: 3-phenylpropanoate and 3-(3-hy	121.0761	967.6384	1116.131	2506.312	2432.974	2026.868	11285.45	990.331	18079.63	5084.087	43814.43	54304.6
PWY-5994: palmitate biosynthesis (type I fatty	112.9848	158.5685	79.21983	0	0	0	0	0	0	0	0	0
PWY-7858: (5Z)-dodecenoate biosynthesis II	109.9191	2961.571	2780.451	3951.653	3728.426	2602.314	12960.74	845.138	17763.7	6354.645	37939.69	46168.18
PWY-7340: 9-cis, 11-trans-octadecadienoyl-C	107.7998	605.9625	141.7218	770.3024	305.0685	1194.942	1319.123	284.997	1296.072	523.5963	138.0095	396.8092
PWY0-1338: polymyxin resistance	104.0367	836.3787	1280.349	1803.312	1620.622	1356.714	6438.229	333.2275	10185.34	3380.12	32685.88	38432.28
PWY-5981: CDP-diacylglycerol biosynthesis I	103.2764	1489.43	1582.511	1976.239	1935.785	1573.573	3955.682	4316.258	6225.731	4012.573	16331.16	26478.87
P163-PWY: L-lysine fermentation to acetate a	102.1003	144.9358	125.6027	0	0	0	0	0	0	0	0	0
GLYCOLYSIS-TCA-GLYOX-BYPASS: superp	99.38393	919.1176	1676.187	955.6585	948.9488	1205.955	9749.58	580.9301	15962.03	4870.326	12052.77	14287.03
PWY-7942: 5-oxo-L-proline metabolism	98.61014	1097.079	1569.042	863.5411	1895.82	1714.025	5103.148	1211.636	3086.459	5696.737	141.3135	7443.127
NAD-BIOSYNTHESIS-II: NAD salvage pathwa	97.66262	2604.802	1558.005	1413.783	1781.793	1093.979	8481.856	402.2271	14028.24	2698.395	24894.61	39156.57
PWY-5494: pyruvate fermentation to propano	95.9755	177.5445	25.25519	0	0	0	107.0045	38.99688	231.6808	90.06045	0	0
PWY-7688: dTDP-α-D-ravidosamine ar	95.97427	72.87155	613.2437	21.65573	476.2767	0	4187.897	35.45336	3348.159	0	0	0
PWY490-3: nitrate reduction VI (assimilatory)	91.54872	38.22464	211.0877	0	0	0	270.2628	0	0	0	0	0
PWY-6293: superpathway of L-cysteine biosy	85.48127	894.6436	1677.385	75.22081	221.5023	491.4639	4886.641	356.64	0	2523.251	394.3532	842.8849
P122-PWY: heterolactic fermentation	85.38055	2435.935	1254.243	2330.128	1293.152	603.0923	4648.175	206.4605	6777.765	4123.055	2260.256	20381.77
PWY0-42: 2-methylcitrate cycle I	76.77686	590.89	37.32598	1427.839	1350.905	1405.51	6483.475	387.6135	11309.11	1555.617	37741.33	40561.47
LPSSYN-PWY: superpathway of lipopolysacc	72.7125	602.1382	0	730.5074	0	1290.471	0	0	0	1385.153	21043.6	32330.53
HCAMHPDEG-PWY: 3-phenylpropanoate and	72.21094	544.8013	715.0858	2223.768	2253.151	1606.531	8691.227	523.767	13869.86	3593.542	37017.52	43472.87
PWY-6690: cinnamate and 3-hydroxycinnama	72.21094	544.8013	715.0858	2223.768	2253.151	1606.531	8691.227	523.767	13869.86	3593.542	37017.52	43472.87
PWY-7039: phosphatidate metabolism, as a s	71.61807	436.8648	0	695.4319	638.8322	0	4291.307	170.7154	6665.272	615.1506	96.82216	1280.181
PWY-7316: dTDP-N-acetylviosamine biosynth	70.02874	128.0603	57.69814	648.0948	10260.15	1255.163	4145.122	89.89245	3310.912	591.5377	0	0
PWY-7389: superpathway of anaerobic energ	67.5691	521.964	0	506.0304	288.0238	0	2394.394	122.6744	1388.829	812.5025	0	0
PWY0-1337: oleate β-oxidation	67.3363	429.2757	990.9656	725.0855	727.4708	2461.567	2709.341	164.0138	4186.312	2714.522	4856.529	4726.249
AST-PWY: L-arginine degradation II (AST pat	66.58075	593.051	1435.102	2086.898	1869.111	1384.5	5302.76	229.9819	8681.414	3957.643	42168.05	50823.4
PWY-7094: fatty acid salvage	63.15907	397.9024	969.3473	641.1248	635.5103	2585.69	2409.453	149.0152	3678.705	2570.568	4113.248	3980.594
PWY66-367: ketogenesis	57.57033	817.0385	470.319	108.0426	143.909	375.959	0	0	156.6971	413.2829	0	0
PWY-5972: stearate biosynthesis I (animals)	57.10338	395.9062	47.39622	701.732	260.6267	745.1659	1185.528	327.1883	1131.3	376.8878	270.7612	341.1438

PWY-6749: CMP-legionaminate biosynthesis	55.80272	294.5699	24.47537	2368.47	1503.907	810.9404	1065.555	124.7347	605.9566	309.9389	0	0
PWY-7371: 1,4-dihydroxy-6-naphthoate biosy	53.76052	155.0283	57.02668	122.0117	0	2099.23	132.2857	0	53.436	0	0	0
PWY-8086: (S)-lactate fermentation to propar	51.75432	304.5656	0	674.9826	284.6141	0	1779.023	68.97862	824.9146	483.5082	0	0
PWY-801: homocysteine and cysteine interco	51.48642	552.7963	1074.63	45.28474	134.0949	304.3377	3619.343	216.4473	0	1679.359	466.5965	7751.125
PWY66-389: phytol degradation	50.83437	346.7952	1667.861	1079.347	2131.206	94.09322	5412.178	5229.545	23516.62	3997.079	64123.26	52015.22
PWY-5088: L-glutamate degradation VIII (to p	50.19033	375.2719	0	987.3914	618.6523	0	768.5635	132.826	970.2169	797.0913	0	0
PWY-5705: allantoin degradation to glyoxylate	48.24326	366.8511	85.73281	1112.592	1018.443	837.7067	2812.161	159.6103	4415.43	650.5146	21544.95	29478.53
KDO-NAGLIPASYN-PWY: superpathway of (I	44.84052	683.5516	1129.314	1278.485	882.5495	629.6744	3607.052	77.57616	5071.125	953.6475	10025.11	18307.67
LIPA-CORESYN-PWY: lipid A-core biosynthe	43.50676	216.3629	0	211.1242	0	1530.942	0	0	0	589.4381	20369.56	30610.14
PWY0-461: L-lysine degradation I	42.74809	357.0924	0	1460.678	1554.728	302.7258	1167.446	14.99939	1771.496	247.2967	25080.24	10463.94
PWY-7384: anaerobic energy metabolism (inv	42.02514	348.7735	0	466.0447	305.014	0	1909.817	78.57968	1022.563	532.4733	0	0
PRPP-PWY: superpathway of histidine, purine	41.81825	4033.551	4216.871	4099.454	3965.308	7538.92	3179.149	0	6113.395	6366.016	6793.738	11287.88
PWY1G-0: mycothiol biosynthesis	38.62802	150.8319	87.35309	0	26.63767	0	0	0	0	34.13166	210.7946	0
ECASYN-PWY: enterobacterial common antig	37.96933	312.6377	1207.938	1143.396	1179.99	945.8006	2988.065	178.8636	5145.346	2884.764	17540.56	23385.48
P221-PWY: octane oxidation	37.78318	308.0082	1011.573	356.5437	491.5526	85.96106	2061.181	3883.614	4298.151	3286.746	16605.69	21436.32
PWY-7294: D-xylose degradation IV	32.45503	314.9935	91.77834	60.52349	0	776.8146	0	0	0	373.5793	0	0
PWY-7159: 3,8-divinyl-chlorophyllide a biosyn	31.88101	72.57675	13.45117	0	0	110.101	97.37855	40.73045	85.75245	338.9762	0	0
PWY-5531: 3,8-divinyl-chlorophyllide a biosyn	30.24812	72.58148	13.48765	0	0	110.7639	97.81086	44.08424	86.51607	362.4633	0	0
PWY-6953: dTDP-3-acetamido-α-D-fuc	30.24098	18.96779	367.4353	0	0	0	3192.449	25.1042	2196.671	0	0	0
PWY-5747: 2-methylcitrate cycle II	28.28176	224.3679	21.79314	330.2587	336.2686	217.4004	4523.601	226.2606	7530.129	1460.163	6123.652	8957.485
PWY-7731: superpathway of photosynthetic h	27.9092	0	0	0	0	0	0	0	0	13.60659	0	0
PWY-7374: 1,4-dihydroxy-6-naphthoate biosy	27.64346	131.9179	41.10402	0	0	0	0	0	0	0	0	0
PWY0-41: allantoin degradation IV (anaerobic	26.78538	211.0954	0	701.9716	555.7891	280.0377	2534.283	124.2923	3925.882	445.8759	8792.574	16755.26
DENOVOPURINE2-PWY: superpathway of pu	24.27118	3628.053	4215.402	3712.483	3507.532	7444.688	2268.469	0	4143.433	6071.434	6066.996	10149.57
PWY-5692: allantoin degradation to glyoxylate	22.70653	174.4003	0	476.1292	51.79451	230.8009	1439.644	60.48181	2377.074	310.1973	7477.268	12339.91
URDEGR-PWY: superpathway of allantoin de	22.70653	174.4003	0	476.1292	51.79451	230.8009	1439.644	60.48181	2377.074	310.1973	7477.268	12339.91
PWY-6210: 2-aminophenol degradation	21.78653	47.07395	161.6188	0	0	0	7.137882	0	4.929312	286.696	0	2136.47
PWY-5055: nicotinate degradation III	21.20156	159.0407	18.39668	50.40647	0	0	0	0	0	0	0	0
PWY-7409: phospholipid remodeling (phosph	21.15757	160.8844	0	747.2288	0	0	0	0	93.41951	454.17	9103.026	18464.51
PWY-7723: bacterial bioluminescence	20.18409	209.0918	132.7911	0	0	0	0	0	0	0	0	0
PWY-7187: pyrimidine deoxyribonucleotides c	20.13092	1532.908	1685.478	2010.668	1775.21	2841.271	1853.937	0	3419.027	3890.353	5081.466	8603.813
ARGDEG-PWY: superpathway of L-arginine,	19.22773	78.22507	588.1083	1064.858	678.3785	0	2518.772	116.8905	4001.403	2047.198	30897.79	45745.08
ORNARGDEG-PWY: superpathway of L-argi	19.22773	78.22507	588.1083	1064.858	678.3785	0	2518.772	116.8905	4001.403	2047.198	30897.79	45745.08
3-HYDROXYPHENYLACETATE-DEGRADAT	18.98332	207.721	707.9767	0	0	0	4463.363	205.5105	7288.423	2215.382	17671.68	6092.153
PWY-101: photosynthesis light reactions	18.76313	0	0	0	0	0	0	0	0	9.074058	0	0

PWY-7446: sulfoquinovose degradation I	18.01258	168.5847	50.56139	610.8605	626.5195	369.8509	2034.453	94.21985	3340.906	96.49613	8247.616	17956.43
PWY-7805: (aminomethyl)phosphonate degra	17.91037	199.115	0	583.4644	89.04147	154.3269	0	0	729.8711	636.7573	11691.03	9660.574
CARNMET-PWY: L-carnitine degradation I	16.72558	137.0197	0	451.2913	334.8296	483.0946	37.85296	0	406.3592	235.4838	5151.081	8908.189
PWY-7807: glyphosate degradation III	16.57856	189.6355	3.79851	542.0897	78.90842	137.481	0	0	915.9612	683.9902	15231.65	9514.592
CATECHOL-ORTHO-CLEAVAGE-PWY: cate	13.86142	35.59753	806.483	0	0	9.482519	0	0	0	1852.544	0	2780.02
PWY-5724: superpathway of atrazine degrada	13.36637	54.26865	0	0	0	50.09117	100.5881	0	205.7062	0	0	0
PWY-7616: methanol oxidation to carbon diox	11.55553	6.851527	130.1652	0	68.88466	0	716.8474	130.6047	1311.922	591.4825	843.3208	4744.453
THREOCAT-PWY: superpathway of L-threon	10.81966	99.81856	2186.352	279.4385	227.9668	76.78172	7395.869	1432.882	9029.134	4666.792	0	9755.484
PWY-7399: methylphosphonate degradation I	10.78082	14.60599	0	0	0	0	23.17928	82.19125	0	0	0	0
PWY0-1533: methylphosphonate degradation	9.619339	112.4504	2.171946	322.131	45.30671	79.48322	0	0	531.4108	405.1139	10332.77	5863.212
PWY-6318: L-phenylalanine degradation IV (r	9.422751	119.2893	0	130.6849	51.21751	109.1291	413.7994	113.1441	577.4296	263.157	88.65741	3000.881
PWY-7820: teichuronic acid biosynthesis (B. s	9.033016	2.512259	260.8617	1967.249	1524.059	0	18.0453	196.2618	264.3121	0	0	0
GLYCOL-GLYOXDEG-PWY: superpathway o	8.235992	265.8044	67.1582	437.2379	323.622	499.0506	3658.693	184.8496	5266.457	703.3706	11003.39	13864.84
P125-PWY: superpathway of (R,R)-butanedio	8.19228	595.0864	182.4119	52.60977	177.1977	411.0585	75.79377	403.3574	214.1641	427.9004	8.521794	547.5897
UBISYN-PWY: superpathway of ubiquinol-8 b	8.031584	223.5944	0	467.0186	436.022	311.6091	0	0	1759.908	185.9371	672.5077	5880.946
PWY-7218: photosynthetic 3-hydroxybutanoat	7.638283	0	0	0	0	0	0	0	0	0	0	0
PWY-6708: ubiquinol-8 biosynthesis (early de	7.156774	203.4936	0	421.2141	393.6658	279.5904	0	0	1586.148	166.3863	604.956	5440.046
PWY-7124: ethene biosynthesis V (engineere	6.545946	0	0	0	0	0	0	0	0	0	0	0
PWY0-1221: putrescine degradation II	6.112989	13.88631	0	437.9246	243.3838	0	1601.163	78.63851	2527.812	254.6107	15873.27	18343.68
DARABCATK12-PWY: D-arabinose degradat	5.229156	266.2866	67.60921	415.9893	304.4577	491.2549	3546.429	181.302	5130.414	680.2538	10733.2	13728.61
PWY-5855: ubiquinol-7 biosynthesis (early de	5.174275	285.4831	874.862	407.0157	428.7596	290.8867	4488.62	205.4882	7307.95	2163.985	16516.6	18592.27
PWY-5654: 2-amino-3-carboxymuconate sem	4.098187	2.544635	161.6188	0	0	0	0	0	0	52.27581	0	2136.47
PHOTOALL-PWY: oxygenic photosynthesis	4.044161	0	0	0	0	0	0	0	0	0	0	0
PWY-5910: superpathway of geranylgeranyld	0	1082.438	399.3725	57.86125	357.5647	459.7215	0	0	269.4004	144.5402	139.6596	446.0098
PWY-5180: toluene degradation I (aerobic) (v	0	1046.296	1089.695	2493.242	2362.493	2058.57	0	0	0	5029.117	0	56533.53
PWY-922: mevalonate pathway I (eukaryotes	0	778.1895	283.6466	40.56863	258.2693	328.6033	0	0	190.8304	105.1022	105.5885	328.5605
PWY-5464: superpathway of cytosolic glycoly	0	718.0712	489.8751	332.6975	403.9736	305.5198	220.0012	1139.837	1327.968	2176.677	0	0
PWY-2201: folate transformations I	0	323.1148	0	0	0	0	0	0	0	0	0	0
DENITRIFICATION-PWY: nitrate reduction I (	0	305.0645	44.48274	0	0	0	0	0	0	0	0	0
PWY-7269: mitochondrial NADPH production	0	206.821	1272.756	833.1897	1018.401	0	7288.302	473.0016	13675.07	3488.759	31504.07	29823.1
AEROBACTINSYN-PWY: aerobactin biosynth	0	200.5699	0	1442.337	1199.916	403.4348	680.9555	369.3389	10988.2	523.9379	1345.303	665.8172
PWY-6562: norspermidine biosynthesis	0	186.2796	1110.669	0	0	0	0	0	0	79.50143	0	0
PWY-5415: catechol degradation I (meta-clea	0	184.708	36.03127	33.52413	178.3255	245.6878	0	0	0	198.3234	0	1205.134
P621-PWY: nylon-6 oligomer degradation	0	159.818	68.17939	45.19178	0	70.13636	0	0	0	249.1861	0	760.3606
PWY-8190: L-glutamate degradation XI (redu	0	102.066	33.12795	89.94231	29.18585	114.3243	142.4626	60.65847	649.9754	1705.349	0	0

PWY-822: fructan biosynthesis	0	94.00093	48.07386	45.17096	0	119.6534	0	0	0	21.34956	0	0
UDPNACETYLGALSYN-PWY: UDP-N-acetyl	0	86.81395	18.73639	0	0	23.34754	0	0	13.9826	0	185.2146	0
PROTOCATECHUATE-ORTHO-CLEAVAGE	0	78.65924	927.152	8.441394	0	11.48434	0	0	79.67455	3605.376	0	3846.598
PWY-7165: L-ascorbate biosynthesis VIII (eng	0	67.45574	800.8795	0	0	23.33329	0	0	0	1978.721	0	13568.79
PWY-2221: Entner-Doudoroff pathway III (ser	0	47.32384	414.1463	0	0	0	0	0	0	2395.257	0	0
PWY-7874: L-threonate degradation	0	46.79727	0	35.19644	0	78.28473	987.7724	45.06038	1629.8	322.6966	13119.53	14061.96
PWY-5417: catechol degradation III (ortho-cle	0	42.31726	956.8871	0	0	0	0	0	0	2438.605	0	3817.489
PWY-5431: aromatic compounds degradation	0	42.31726	956.8871	0	0	0	0	0	0	2438.605	0	3817.489
PWY-6143: CMP-pseudaminate biosynthesis	0	37.31062	0	16.56658	0	21.10327	0	0	0	0	0	0
PWY-7245: superpathway of NAD/NADP - NA	0	34.95527	686.2011	0	0	0	0	0	0	2190.848	0	90.35271
PWY-7268: cytosolic NADPH production (yea	0	28.62845	700.1232	0	0	0	0	0	0	2310.728	0	67.84345
PWY-7873: D-erythronate degradation II	0	28.35518	0	29.41463	0	50.55723	444.239	0	723.6912	322.6966	13119.53	14061.96
12DICHLORETHDEG-PWY: 1,2-dichloroetha	0	18.58941	56.49476	0	0	0	0	0	0	0	0	0
PWY-7031: protein N-glycosylation (bacterial)	0	18.13711	0	0	0	0	0	0	0	0	0	0
PWY-6215: 4-chlorobenzoate degradation	0	13.76764	0	11.84104	0	0	0	0	0	470.5059	0	2043.96
URSIN-PWY: ureide biosynthesis	0	13.653	296.6621	0	0	0	0	0	0	316.5659	252.0856	3225.679
PWY66-430: myristate biosynthesis (mitochor	0	13.05246	795.0676	1276.971	1196.769	0	9284.956	502.3864	15409.54	2475.756	258.655	6894.263
PWY-7388: octanoyl-[acyl-carrier protein] bios	0	12.44786	863.9586	1241.06	1167.206	0	9198.466	498.3515	15299.57	2525.474	246.643	6605.334
PWY-7332: superpathway of UDP-N-acetylglu	0	12.40969	0	45.83345	0	0	0	0	0	0	0	0
PWY-6182: superpathway of salicylate degrad	0	12.32326	831.7186	0	0	0	0	0	0	2133.01	0	3569.24
PWY-6518: bile acids epimerization	0	11.80232	0	0	0	0	0	0	0	0	0	0
PWY-3801: sucrose degradation II (sucrose s	0	11.23167	611.8129	0	0	0	0	0	0	1504.513	398.7361	3839.838
TYRFUMCAT-PWY: L-tyrosine degradation I	0	11.21408	0	0	0	0	0	0	0	33.57623	0	0
PWY-6185: 4-methylcatechol degradation (or	0	9.74982	880.9864	0	0	0	0	0	0	3118.658	0	3888.558
PWY-7396: butanol and isobutanol biosynthes	0	9.52681	332.1914	0	0	0	0	0	0	980.8728	366.8293	3133.804
PWY-5514: UDP-N-acetyl-D-galactosamine b	0	4.592011	18.42691	0	0	0	0	0	13.86191	0	103.1516	0
PWY5F9-12: biphenyl degradation	0	0	132.1926	0	0	0	0	0	0	1154.674	0	46.25453
P562-PWY: myo-inositol degradation I	0	0	77.95796	0	0	0	0	0	0	76.60909	0	0
PWY-1501: mandelate degradation I	0	0	25.39769	0	0	0	0	0	0	99.00024	0	0
PWY-6641: superpathway of sulfolactate degr	0	0	17.25006	0	0	0	0	0	0	213.9546	0	0
PWY-7312: dTDP-β-D-fucofuranose bios	0	0	0	3123.305	27534.86	14651.76	0	0	0	0	0	0
PWY-8173: anteiso-branched-chain fatty acid	0	0	0	399.3012	517.7758	0	208.8509	2499.312	2754.164	0	0	0
PWY-8174: odd iso-branched-chain fatty acid	0	0	0	399.3012	517.7758	0	208.8509	2499.312	2754.164	0	0	0
PWY-8175: even iso-branched-chain fatty aci	0	0	0	399.3012	517.7758	0	208.8509	2499.312	2754.164	0	0	0
PWY-6328: L-lysine degradation X	0	0	0	41.16755	0	22.67666	1167.446	14.99939	1771.496	147.7009	3343.751	8206.473

PWY-6471: peptidoglycan biosynthesis IV (Er	0	0	0	0	352.5439	0	0	0	0	0	0	0
CHLOROPHYLL-SYN: 3,8-divinyl-chlorophylli	0	0	0	0	0	51.3098	0	0	0	0	0	0
PWY-6071: superpathway of phenylethylamin	0	0	0	0	0	0	3539.983	180.9362	5231.875	271.4914	10271.36	20429.46
PWY0-321: phenylacetate degradation I (aerc	0	0	0	0	0	0	3247.955	169.0679	4809.606	226.1605	9252.339	19562.99
PWY-7317: superpathway of dTDP-glucose-d	0	0	0	0	0	0	164.6303	0	0	0	0	0
PWY-7754: bile acid 7α-dehydroxylatior	0	0	0	0	0	0	28.15583	0	48.21985	0	0	0
PWY-8134: bile acid 7β-dehydroxylation	0	0	0	0	0	0	26.4055	0	49.1192	0	0	0
PWY-7318: dTDP-3-acetamido-3,6-dideoxy-&	0	0	0	0	0	0	0	513.8114	0	0	0	0
PWY-6138: CMP-N-acetylneuraminate biosyr	0	0	0	0	0	0	0	21.99548	0	0	0	0
PWY-6396: superpathway of 2,3-butanediol b	0	0	0	0	0	0	0	0	129.3935	299.1605	0	720.7866
PWY-7391: isoprene biosynthesis II (engineer	0	0	0	0	0	0	0	0	117.6379	53.9474	83.30726	177.2701
PWY-7196: superpathway of pyrimidine ribon	0	0	0	0	0	0	0	0	0	252.6116	0	0
PWY-6957: mandelate degradation to acetyl-(	0	0	0	0	0	0	0	0	0	161.991	0	0
PWY-5178: toluene degradation IV (aerobic) (	0	0	0	0	0	0	0	0	0	80.33159	0	0
DHGLUCONATE-PYR-CAT-PWY: glucose de	0	0	0	0	0	0	0	0	0	73.2614	0	0
PWY-3781: aerobic respiration I (cytochrome	0	0	0	0	0	0	0	0	0	65.19508	0	0
PWY-6565: superpathway of polyamine biosy	0	0	0	0	0	0	0	0	0	64.9031	0	0
PWY-7279: aerobic respiration II (cytochrome	0	0	0	0	0	0	0	0	0	64.89074	0	0
PWY-7528: L-methionine salvage cycle I (bac	0	0	0	0	0	0	0	0	0	48.97896	0	0
PWY-7527: L-methionine salvage cycle III	0	0	0	0	0	0	0	0	0	44.94649	0	0
PWY-7783: plasmalogen degradation	0	0	0	0	0	0	0	0	0	40.13119	0	0
PWY-5028: L-histidine degradation II	0	0	0	0	0	0	0	0	0	33.61818	0	0
PWY-4361: S-methyl-5-thio-α-D-ribose	0	0	0	0	0	0	0	0	0	28.84202	0	0
PWY-5004: superpathway of L-citrulline meta	0	0	0	0	0	0	0	0	0	24.64144	0	0
ALL-CHORISMATE-PWY: superpathway of c	0	0	0	0	0	0	0	0	0	0	1277.591	0
PWY-7411: phosphatidate biosynthesis (yeas	0	0	0	0	0	0	0	0	0	0	288.3001	0
PWY-6981: chitin biosynthesis	0	0	0	0	0	0	0	0	0	0	260.04	0
PWY-6421: arsenate detoxification IV (mycotl	0	0	0	0	0	0	0	0	0	0	257.1514	0
PWY-5870: ubiquinol-8 biosynthesis (late dec	0	0	0	0	0	0	0	0	0	0	190.4147	0
PWY-5873: ubiquinol-7 biosynthesis (late dec	0	0	0	0	0	0	0	0	0	0	132.2947	0
PWY-7511: protein ubiquitination	0	0	0	0	0	0	0	0	0	0	106.1037	0
NADSYN-PWY: NAD de novo biosynthesis II	0	0	0	0	0	0	0	0	0	0	81.46002	0
PWY-5080: very long chain fatty acid biosynth	0	0	0	0	0	0	0	0	0	0	73.60372	0
PWY3DJ-12: ceramide de novo biosynthesis	0	0	0	0	0	0	0	0	0	0	65.18898	0
PWY-5651: L-tryptophan degradation to 2-am	0	0	0	0	0	0	0	0	0	0	56.40117	0

PWY-6351: D-myo-inositol (1,4,5)-trisphospha	0	0	0	0	0	0	0	0	0	0	44.96503	0
PWY-6313: serotonin degradation	0	0	0	0	0	0	0	0	0	0	0	2.118582
PWY-7431: aromatic biogenic amine degrada	0	0	0	0	0	0	0	0	0	0	0	2.11852
PWY-6307: L-tryptophan degradation X (mam	0	0	0	0	0	0	0	0	0	0	0	1.588965
CRNFORCAT-PWY: creatinine degradation I	0	0	0	0	0	0	0	0	0	0	0	0
P162-PWY: L-glutamate degradation V (via hy	0	0	0	0	0	0	0	0	0	0	0	0
PWY-7000: kanamycin biosynthesis	0	0	0	0	0	0	0	0	0	0	0	0
PWY-7003: glycerol degradation to butanol	0	0	0	0	0	0	0	0	0	0	0	0
PWY-6604: superpathway of Clostridium acet	0	0	0	0	0	0	0	0	0	0	0	0
PWY-6594: superpathway of Clostridium acet	0	0	0	0	0	0	0	0	0	0	0	0
GLUDEG-II-PWY: L-glutamate degradation V	0	0	0	0	0	0	0	0	0	0	0	0
PWY-6583: pyruvate fermentation to butanol	0	0	0	0	0	0	0	0	0	0	0	0
PWY-6435: 4-hydroxybenzoate biosynthesis I	0	0	0	0	0	0	0	0	0	0	0	0
PWY-6920: 6-gingerol analog biosynthesis (e	0	0	0	0	0	0	0	0	0	0	0	0
PWY-7090: UDP-2,3-diacetamido-2,3-dideoxy	0	0	0	0	0	0	0	0	0	0	0	0
PWY-6167: flavin biosynthesis II (archaea)	0	0	0	0	0	0	0	0	0	0	0	0
PWY-6165: chorismate biosynthesis II (archa	0	0	0	0	0	0	0	0	0	0	0	0
PWY66-388: ceramide degradation by &alpha	0	0	0	0	0	0	0	0	0	0	0	0
PWY-6349: CDP-archaeol biosynthesis	0	0	0	0	0	0	0	0	0	0	0	0
PWY-7286: 7-(3-amino-3-carboxypropyl)-wyo	0	0	0	0	0	0	0	0	0	0	0	0
PWY-6350: archaetidylinositol biosynthesis	0	0	0	0	0	0	0	0	0	0	0	0
METHANOGENESIS-PWY: methanogenesis	0	0	0	0	0	0	0	0	0	0	0	0
PWY-5198: factor 420 biosynthesis II (mycob	0	0	0	0	0	0	0	0	0	0	0	0
PWY-6160: 3-dehydroquinate biosynthesis II	0	0	0	0	0	0	0	0	0	0	0	0
PWY-8112: factor 420 biosynthesis I (archaea	0	0	0	0	0	0	0	0	0	0	0	0
PWY-8113: 3PG-factor 420 biosynthesis	0	0	0	0	0	0	0	0	0	0	0	0
PWY-5209: methyl-coenzyme M oxidation to	0	0	0	0	0	0	0	0	0	0	0	0
PWY-6174: mevalonate pathway II (haloarcha	0	0	0	0	0	0	0	0	0	0	0	0
PWY-8125: mevalonate pathway IV (archaea)	0	0	0	0	0	0	0	0	0	0	0	0
P241-PWY: coenzyme B biosynthesis	0	0	0	0	0	0	0	0	0	0	0	0
PWY-8055: palmitoyl ethanolamide biosynthe	0	0	0	0	0	0	0	0	0	0	0	0
PWY-7337: 10-cis-heptadecenoyl-CoA degra	0	0	0	0	0	0	0	0	0	0	0	0
PWY-7338: 10-trans-heptadecenoyl-CoA deg	0	0	0	0	0	0	0	0	0	0	0	0
PWY-6748: nitrate reduction VII (denitrificatio	0	0	0	0	0	0	0	0	0	0	0	0
PWY-6837: fatty acid β-oxidation V (uns	0	0	0	0	0	0	0	0	0	0	0	0

PWY-5647: 2-nitrobenzoate degradation I	0	0	0	0	0	0	0	0	0	0	0	0
PWY-7420: monoacylglycerol metabolism (ye	0	0	0	0	0	0	0	0	0	0	0	0
PWY-6113: superpathway of mycolate biosyn	0	0	0	0	0	0	0	0	0	0	0	0
PWY-5156: superpathway of fatty acid biosyn	0	0	0	0	0	0	0	0	0	0	0	0
PWY-6467: Kdo transfer to lipid IVA III (Chlan	0	0	0	0	0	0	0	0	0	0	0	0
PWY-181: photorespiration	0	0	0	0	0	0	0	0	0	0	0	0
PWY-1622: formaldehyde assimilation I (serir	0	0	0	0	0	0	0	0	0	0	0	0
PWY-8011: L-serine biosynthesis II	0	0	0	0	0	0	0	0	0	0	0	0
PWY-7290: Escherichia coli serotype O86 O-a	0	0	0	0	0	0	0	0	0	0	0	0
PWY-7413: dTDP-6-deoxy-α-D-allose b	0	0	0	0	0	0	0	0	0	0	0	0
PWY-6662: superpathway of quinolone and a	0	0	0	0	0	0	0	0	0	0	0	0
PWY-5742: L-arginine degradation IX (argini	0	0	0	0	0	0	0	0	0	0	0	0
PWY-7905: Escherichia coli serotype O9a O-a	0	0	0	0	0	0	0	0	0	0	0	0
PWY-6797: 6-hydroxymethyl-dihydropterin dip	0	0	0	0	0	0	0	0	0	0	0	0
PWY-5420: catechol degradation II (meta-clea	0	0	0	0	0	0	0	0	0	0	0	0
PWY-5419: catechol degradation to 2-hydroxy	0	0	0	0	0	0	0	0	0	0	0	0
P261-PWY: coenzyme M biosynthesis I	0	0	0	0	0	0	0	0	0	0	0	0

AP005_1	AP005_2	AP005_3	AP006_1	AP006_2	AP006_3	AP007_1	AP007_2	AP007_3	AP008_1	AP008_2	AP008_3	AP009_1	AP009_2	AP009_3	AP010_1
12842055	12362441	21392890	14030415	12891976	11403728	18677988	16535315	21510438	14359149	21071966	19248656	9926887	10542605	14080028	9571212
56703574	66741665	77568424	73749543	65050197	65746684	1.02E+08	1.13E+08	1.01E+08	1.13E+08	1.26E+08	1.19E+08	46171296	52629102	36696849	52774220
76478.64	57055.03	66014.88	68436.1	71468.85	50710.52	49036.2	43950.43	44107.13	21423.03	64752.63	44031.03	38331.35	36803.83	34219.26	34729.04
35790.03	29840.76	54615.29	50399.37	44191.51	38689.84	40483.42	41987.22	43007.95	34849.01	51252.91	46958.38	30069.76	28219.66	28596.42	32085.71
35123.24	31325.69	66612.32	55026.51	45849.92	40833.28	53053.22	61463.3	58056.04	53228.2	66386.19	65839.69	31442.4	32869.96	28012.77	28642.28
35251.19	27428.21	62250.39	51566.05	42942.07	33737.05	46664.93	38245.6	47723.81	36274.27	44775.48	38863.54	30391.65	29374.07	25389.84	30322.36
35251.19	27428.21	62250.39	51566.05	42942.07	33737.05	46664.93	38245.6	47723.81	36274.27	44775.48	38863.54	30391.65	29374.07	25389.84	30322.36
35251.19	27428.21	62250.39	51566.05	42942.07	33737.05	46664.93	38245.6	47723.81	36274.27	44775.48	38863.54	30391.65	29374.07	25389.84	30322.36
33901.42	36139.7	62370.45	47007.89	40957.32	36150.19	51734.89	58154.33	51264.93	60118.91	64739.16	63813.4	34469.67	32526.29	33977.12	34502.88
32780.94	28224.43	60799.34	47052.42	31134.98	34947.09	45298.71	37234.37	46264.98	35818.15	47187.55	50757.71	30831.19	30253.66	31465.74	29856.51
32809.08	27708.18	53716.07	48003	40601.41	33467.17	41859.21	37985.44	43201.09	40993	51700.37	43819.9	28189.26	26049.1	27226.27	28256.63
31310.08	23459.29	55984.94	48191.6	42161.73	35269.61	42592.05	34928.09	42382.98	34255.88	46203.3	43004.6	28700.6	26136.2	28158.74	27564.36
30422.26	21457.73	51714.08	49064.85	37544.54	33444.48	33469.51	31069.11	35617.4	26293.25	40703.07	36620.85	25565.25	22539.3	20880.35	25717.42
31269.86	25842	59898.18	47110.54	42175.84	35894.76	47355.92	43276.38	50903.89	42897.3	53704.67	52076.89	28703.96	26374.12	29111.13	28527.51
30675.77	22893.48	60405.83	45622.2	40033.87	34271.82	46582.18	42059.31	51094.15	40137.67	51341.82	49789.97	28127.84	26101.97	28031.98	28626.63
35916.85	27791.3	59198.77	43723.72	43197.2	35575.53	37786.47	39393.05	37749.81	35465.62	54763.13	48038.72	27575.25	22710.08	24659.46	30503.51
34903.42	27583.77	53135.73	48671.44	47398.17	37075.79	42294.11	36057.89	44087.89	36024.67	49171.93	50138.62	29376.82	28062.3	27615.49	28763.71
27627.14	22258.81	55417.91	45462.41	41292.77	33236.96	37051.98	34245.98	41677.95	30527.61	46313.1	40921.5	26512.08	22105.71	26215.94	25452.42
30556.44	24314.95	59463.88	45551.02	41871.69	34443.65	46265.07	42148.81	50670.9	41198.51	52060.77	52245.13	28303.11	26089.64	28797.2	28411.43
34909.77	29574.04	50109.54	48179.6	37987.28	36891.61	44494.48	39901.41	47664.73	39712.77	52313.23	54587.65	29233.94	29521.55	30135.83	28364.71
29650.63	23940.37	51282.74	45512.42	38918.65	35200.96	43459.44	40744.94	42792.18	38119.34	49093.1	48846.26	25080.24	24980.16	20967.5	27215.34
30533.83	24539.89	59440.35	45332.97	41534.87	34496.29	45471.84	41078.6	49452.8	40139.32	51064.17	49268.42	28258.18	26085.25	29154.8	28120.82
29450.6	21301.51	57245.93	44671.47	41887.59	34035.08	45666.89	41095.32	50742.12	39055.69	49944.39	49044.3	28074.49	25730.04	28013.8	28362.3
29149.05	24484.71	53095.91	44245.74	37098.11	33794.38	43371.08	40083.62	42324.44	39828.86	49099.83	48444.74	25203.74	24891.59	23353.67	26891.17
29149.05	24484.71	53095.91	44245.74	37098.11	33794.38	43371.08	40083.62	42324.44	39828.86	49099.83	48444.74	25203.74	24891.59	23353.67	26891.17
26016.02	49675.43	58782.88	45987.47	27247.2	46014.81	75137.17	90926.79	77121.04	116352.3	97901.65	126143.6	29686.82	30005.48	23199.95	27780.84
30607.68	24061.92	51389.8	43411.16	36861.94	30729.22	38450.3	34308.63	37739.31	29133.25	43253.34	37798.34	27227.31	25093.59	22532.9	26289.75
31519.27	28202.03	46874.42	43618.21	41272.22	31698.86	42190.37	39660.54	43571.97	43838.42	52090.85	45177.47	26514.17	24995.03	23801.93	27250.02
30403.36	25812.68	51577.64	41640.78	37890.57	31672.82	44906.78	40076.26	45461.03	40397.23	49692.32	49495.56	26325.32	25705.98	25036.5	25123.62
33422.15	26157.29	44795.36	42702.14	35462.21	32155.93	43751.86	41935.82	46815.6	47837.94	50896.77	54269.16	27226.19	26836.83	25723.78	26464.71
33422.15	26157.29	44795.36	42702.14	35462.21	32155.93	43751.86	41939.17	46815.6	47873.69	51056.44	54288.25	27226.19	26836.83	25737.01	26464.71
31408.23	26560.04	48951.13	42778.73	42105.51	33177.14	44167.05	39886	45615.08	35905.88	48322.65	43503.96	26827.44	24903.15	24094.72	25726.44
29653.05	23116.03	52144.68	42296.06	40979.37	31582.75	38441.7	33576.47	38577.64	29053.97	42291.83	37251.28	25878.59	24035.31	21946.41	25353.82

29653.05	23116.03	52144.68	41945.51	34733.19	29748.66	36412.65	32065.5	35123.57	26718.42	41387.78	35397.92	25878.59	23692.22	21946.41	25353.82
34836.65	36733.24	47616.49	42794.86	43797.32	36305.61	42525.44	48234.75	38154.75	59103.28	60224.59	51887.37	27112.93	24785.76	23431.68	25903.01
20931.38	20737.3	36370.22	40629.82	16471.07	24859.14	55049.1	46566.29	63115.09	50354.69	40308.23	46437.58	18321.74	22179.46	12545.59	24388.87
29455.25	24552.9	50243.4	40485.94	38151.7	29358.27	38634.7	36739.39	39982.52	43073.4	45857.04	38422.38	25586.52	23975.78	22574.41	26518.9
29154.39	23157.97	50916.03	39570.58	39722.33	31862.54	38736.52	32998.72	39869.28	44359.69	43955.33	40765.4	24201.75	23731.66	21565.1	26870.73
33175.58	25997.17	52818.7	37937.52	39916.21	32460.63	30928.64	36289.7	30749.91	33119.08	52248.17	43482.49	24102.55	18574.76	20691.48	27870.15
27155.03	25601.81	44859.84	40440.39	37052.32	27914.99	38880.1	39602.04	41867.72	44387.96	51499.56	44062.47	23233.89	23081.02	21491.82	25155.97
30954.18	28497.65	33966.44	41245.72	39326.24	36488.27	31533.44	37604.89	28686.72	31050.78	50759.12	39559.36	22957.73	22605.68	19827.96	23060.04
28259.27	23856.39	49839.11	37788.25	40245.67	31445.42	36364.31	36334.22	38075.48	34117.45	41180.42	41871	23881.61	21689.97	20755.24	21146.08
30989.92	22972.37	33816.62	39510.45	40014.03	33716.5	32480.77	28089.79	27583.39	27785.28	42846.53	38091.71	23359.87	22513.25	19073.45	22058.96
26866.35	25923.11	44670.35	34543.39	39459.87	28871.83	41958.82	44494.56	42136.02	45077.13	49467.71	49355.99	24697.67	24965.64	23077.67	24553.24
25633.72	20016.42	40974.22	39232.92	30760.56	29328.29	35288.07	32227.39	33116.11	27972.46	42837.68	39874.43	25066.38	21346.77	25357.5	22133.82
30872.71	27147.47	38899.4	35974.82	40330.82	31748.73	41531.17	40907.52	42476.62	38926.74	50050.35	45046.9	26864.36	25330.88	24176.27	25125.74
28757.37	23550.77	50171.36	39014.7	40018.8	30756.33	35059.72	32959.24	35487.73	29404.55	39527.16	38066.01	24165.23	21900.5	21201.35	22140.96
30669.86	22761.6	31046.13	38214.09	39551.42	33212.73	31586.65	27592.91	25980.31	22617.78	41930.87	37175.08	22585.61	22040.94	17792.33	21279.25
28131.5	28886.99	42282.84	42258.76	42542.15	33080.51	45343.95	41839.24	43097.35	53182.99	59567.19	57081.1	25030.82	23233.49	18523.74	21200.02
17055.12	19519.23	38375.88	42187.25	16801.54	29041.87	32032.73	32055.36	40976.51	39819.06	38894.96	42445.31	21696.59	18452.8	21526.28	24422.63
29339.15	27505.66	30275.7	38610.05	37843.2	34628.68	31424.59	47472.48	33213.01	59021.54	51030.48	48210.56	21300.39	20841.32	17885.71	21202.48
24843.23	24568.31	39799.57	37993.08	32117.13	28848.92	36177.16	39019.85	36235.98	42783.77	49782.83	47834.01	23343.98	19386.26	15464.87	23848.42
24067.1	19740.46	42386.04	32820.98	32163.33	23607.99	29956.4	28784.85	31579.26	20001.58	37015.41	32654.71	19764.14	17148.33	15605.16	19932.33
32198.94	26185.68	47124.19	35028.11	40194.78	31583.7	27755.02	35352.6	27700.38	34674.25	51476.64	43330.74	22216.31	17068.29	18276.27	26047.43
26987.95	28466.79	38948.02	40117.18	41674.08	31790.03	42868.27	38583.51	39993.02	51358.67	57081.01	54752.17	23871.84	21800.14	17177.74	20214.23
26128.22	21935.95	43426.87	37816.74	32681.3	26109.48	35025.34	32747.51	36957.22	38471.04	42496.52	34102.31	21931.53	21158.33	17743.89	23286.79
23041.78	19836.86	39588.54	36034.45	37668.43	29364.99	27719.25	25481.57	26794.7	21113.99	38590.59	30585.96	21975.22	13444.44	23812.35	17578.48
27982.05	23038.84	42495.34	34285.09	34786.46	25570.86	31409.76	33575.87	30563.57	31240.55	44824.74	41529.06	21104.5	19147.43	17295.91	23761.85
16111.96	20417.92	37073.96	34855.56	30316.45	28549.5	34346.85	44221.86	31918.02	49153.56	45538.05	44358.47	18858.87	16753.79	13478.22	21717.32
30396.77	24778.94	44164.45	32024.94	37686.57	29540.6	24672.43	33285.81	24535.12	32524.9	49655.63	40101.99	20413.45	15080.2	16537.37	24577.35
25463.57	19962.46	35252.5	34275.01	32196.44	29918.72	32281.56	27711.86	30592.32	28391.58	33623.8	33706.63	20528.05	18871.61	17809.95	21410.87
24676.59	26272.84	33995.74	36777.55	40145.09	29124.47	40168.07	34157.43	36317.47	48431.1	53429.05	50830.84	21805.89	19433.93	15045.85	17964.9
25770.89	25569.05	48412.91	33498.77	25842.24	25567.5	34942.42	36810.58	32752.6	33698.13	38509.34	39305.85	18557.58	20756.5	10975.35	19604.63
27363.11	31518.79	47664.84	30364.31	31622.63	24110.15	46238.18	51871.57	46305	50602.93	53061.98	52979.52	21833.76	27065.99	15881.26	25210.3
20938.25	14831.01	28463.85	32765.86	25936.05	27732.11	15923.53	27047.16	21273.17	16694.2	27331.25	26536.27	19338.11	11276.62	20077.4	17610.88
20576.62	24414.35	38344.66	27931.84	17122.27	14041.34	39699.96	31571.1	47204.48	48462.23	37350	45602.59	18088.62	19751.46	15146.79	21370.39
28680.35	24419.4	40009.16	26836.74	33939.78	26753.23	20248.84	33271.61	19950.94	32671.69	52179.11	37518.98	17476.47	12114.87	13647.97	23195.18
28680.35	24419.4	40009.16	26836.74	33939.78	26753.23	20248.84	33271.61	19950.94	32671.69	52179.11	37518.98	17476.47	12114.87	13647.97	23195.18

27776.49	22531.3	38053.3	32742.94	35831.57	27382.41	30711.83	28510.76	31981.6	28752.74	41875.8	38066.59	12566.83	14295.11	11522.83	16509.67
22196.9	19836.67	33270.77	33745.76	32129.67	30173.86	21958.22	23932.64	19915.84	27291.15	41847.01	34745.11	13729.31	10650.46	14798.72	15639.02
13750.37	17184.47	23619.43	25791.07	16529.97	28214.26	18033.76	30101.09	18445.43	38013.24	31046.1	31888.42	12293.96	13737.88	10920.22	15391.73
28110.6	23459.29	37388.53	31805.93	35460.18	26857.5	30543.56	28952.99	31870.5	34255.88	42815.93	39187.5	11719.1	13655.39	10667.84	15769.84
21431.1	25067.55	36265.33	27108.15	16893.11	17300.03	34730.94	36143.31	32121.37	44617.89	46716.53	44973.34	15355.35	20032.71	12300.21	15380.03
19976.92	22360.4	22835.23	29317.33	16322.07	19160.99	42749.57	38530.06	48068.62	52689.91	40398.13	46528.89	16673.43	18750.18	11843.93	18507.98
15483.74	16069.01	31137.6	29193.52	27374.74	20858.55	26326.65	22946.98	24059.6	23636.79	29839.45	27903.46	16086.18	14972.58	13479.41	15794.33
11380.15	18734.58	20415.96	29991.31	3741.23	17002.32	34074.45	38180.71	34813.75	43624.88	42093.55	49394.06	11531.77	11548.62	15285.58	12832.31
23356.73	27467.28	25783.38	29692.81	26642.27	25552.56	17597.64	16904.18	10631.52	14044.38	28634.45	16979.61	12263.81	13481.79	8039.337	12723.39
16087.03	18777.01	41451.7	23913.69	13276.25	14492.16	35758.38	33041.77	40107.32	34922.1	33201.23	43488.5	14625.01	20438.13	15286.73	14642.95
18394.58	20427.48	41314.8	27373.2	24179.2	14897.79	36371.59	33262.57	39899.53	37866.12	30841.63	37051.76	12786.7	20094.52	8135.555	16314.75
23746.04	23603.46	30884.16	31885.4	28727.37	26851.73	34633.64	41152.74	36290.14	41311.24	45047.33	43656.19	9073.534	10644.84	12181.65	12751.73
12634.6	5377.004	21732.49	26270.74	18225.84	24447.98	14539.8	16077.15	9890.536	4898.324	23737.91	16519.97	12348.97	11054.17	8445.003	12573.1
15568.38	7648.629	27853.8	21263.25	20845.2	20739.43	16871.43	25457.85	19842.1	17988.81	29530.92	27627.32	14180.65	11036.75	13658.78	17742.57
23549.01	22590.38	28998	31187.04	28717.57	27022.21	33397.85	39277.29	33905.27	37372.76	43390.84	42390.64	8771.34	10382.98	11327.19	12643.84
15752.68	10676.07	31370.7	18788.73	21234.43	14785.84	20427.4	30459.32	25956.69	20972.45	24306.22	29296.56	10874.28	12079.19	7506.87	16421.52
12380.5	13324.21	32629.57	28001.45	23602.18	17559.9	25235.14	21062.36	26211.85	24272.96	26691.23	24946.93	14268.13	12642.15	10902.19	15784.12
17830.53	20180.34	32871.18	27076.91	22463.17	14779.5	35583.64	32989.36	38174.14	37542.41	30673.2	36210.27	10581.77	19914.01	7087.667	14780.19
17063.71	21431.73	37625.58	26941.08	22621.04	18618.73	27521.31	29546.54	27677.03	39452.19	27977.87	36298.02	13379.17	17466.77	8629.883	16411.33
16436.15	20175.55	38839.25	24292.47	22368.62	13425.68	35123.3	31903.19	39714.94	40071.68	29315.57	37127.43	11864.04	18686.74	7008.875	16052.52
12865.84	37812.23	30182.16	32674.88	30300.61	34584.3	18150.02	17939.56	28247.77	32252.29	44483.27	37953.18	9052.206	9483.374	6006.669	16066.25
9118.312	14956.55	18496.91	26264.28	14217.48	19211.24	24017.1	17555.01	22374.89	27536.51	30535.05	30261.23	16558.21	12048.02	16600.13	10436.73
18725.4	19730.64	40645.9	23340.13	27370.84	13126.79	21068.47	25993.19	21102.89	29084.52	39992.92	30753.54	12721.76	12005.26	12086.17	16704.14
7023.765	10301.06	19371.86	25706.77	11912.65	16446.35	18825.46	15378.91	19525.08	10916.06	22199.15	20462.74	15094.54	9714.628	16962.14	9167.125
15136.22	18606.49	26257.31	24335.91	17659.04	15074.54	28114.44	26600.48	31550.41	35291.74	34623.85	37039.61	9221.267	10969.44	10232.93	12794.24
17331.18	19501.36	13052.44	27111.8	27240.16	24024.35	21135.99	17046.1	17831.21	30505.38	36467.6	29169.33	17396.32	12918.52	12822.47	11464.19
10386.91	4334.202	25982.92	15544.39	15320.21	13551.92	16301.94	28348.64	21122.04	18300.86	21254.3	25484.24	9600.504	9533.488	6836.615	15434.6
24072.47	34514.5	39125.31	22431.51	28787.22	17515.92	43816.43	55224.17	44423.17	72891.15	60524.04	61766.29	16010.05	24700.7	10045.33	21120.79
14666.62	24300.9	35587.09	22929.46	18531.29	13078.37	29309.38	28245.28	34620.24	38328.66	26531.38	36654.75	10800.37	16315.91	6044.771	14875.75
17848.24	22030.62	27131.92	20813.89	21856.69	16130.46	29642.5	36829.77	30782.45	42910.62	42117.29	40512.74	9953.801	15622.85	6571.663	16886.74
6273.839	19240.39	18119	21707.14	10158.7	19472	23310.67	25459.61	18584.72	30563.84	30975.49	31578.03	8217.856	7689.414	7648.72	8369.545
11075.8	10085.5	19861.93	16900.57	15195.08	14964.43	20906.52	18254.99	20330.27	20267.76	24808.81	34586.17	8534.036	10680.73	6471.493	9275.43
15947.63	18567.19	10340.11	27566.69	31049.59	24420.04	18942.85	24399.73	16904.35	27811.44	32756.61	25347.16	17575.94	12402.02	12235.49	9728.606
22028.36	27589.93	29298.61	19526.22	24891.59	17667.74	30172.32	43004.12	32145.11	53638.9	51577.64	49720.27	12320.91	18357.85	7319.341	16632.14
6563.129	15615.34	14243.51	24120.4	2228.584	12226.59	28392.99	31305.43	31022.67	42183.25	37365.8	41553.54	7667.513	7622.132	10893.94	8609.584

6563.129	15615.34	14243.51	24120.4	2228.584	12226.59	28392.99	31304.18	31022.67	42174	37337.22	41546.08	7667.513	7622.132	10892.35	8609.584
12994.48	6146.058	24303.72	18909.9	16137.16	19114.59	13562.62	22934.17	15131.4	12393.33	25790.33	22159.26	8509.899	8823.791	7786.146	12200.24
12006.03	20363.92	38418.88	18241.57	5230.478	11119.6	26061.49	28742.68	26698.68	25870.99	37055.31	37195.59	12580.3	10685.67	15216.46	11840.39
23826.88	36169.11	34258.25	17553.93	26442.79	15241.84	44830.92	58888.64	49833.89	83910.07	61324.58	66101.45	12998.82	24587.19	7632.376	18170.73
14087.71	9361.1	24406.08	15178.64	19055.81	11740.54	9743.814	20755.4	9987.192	6153.142	19229.3	17990.05	3691.998	10657.36	2279.674	14013.33
17453.05	18243.76	5143.957	22408.72	5374.705	19413.96	18610.76	24619.97	16987.38	28632.84	33332.4	28400.42	3322.526	4066.506	2729.503	6924.081
17453.05	18243.76	5143.957	22408.72	5374.705	19413.96	18610.76	24619.97	16987.38	28632.84	33332.4	28400.42	3322.526	4066.506	2729.503	6924.081
7814.136	16297.92	12777.91	12308.06	11086.73	13582.03	19320.02	15302.25	17387.13	35242.17	31572.13	32109.77	3620.113	5077.948	2614.148	8841.319
5433.031	13159.42	5515.494	25845.79	11675.73	23856.06	11173.6	21273.1	15065.82	16377.71	16663.18	10893.04	1472.154	4589.818	1499.506	10403.7
13158.34	16325.37	17756.24	20376.1	6821.376	7556.98	26585.46	23722.57	28079.53	31540.27	31496.13	36667.72	11420.1	13872.71	9999.17	11948.75
20327.11	16779.65	12750.2	24599.94	34067.45	21221.86	11217.9	4669.794	9767.008	7383.908	30206.92	20737.47	11277.83	13964.88	10321.17	12491.84
15217.62	31275.35	45768.25	18493.63	6573.209	10762.56	44159.24	51930.01	46158.59	56449.87	55250.01	54612.47	15877.27	23952.82	8753.354	18107.11
21995.44	17765.87	34061.97	23897.01	34469.87	9943.795	26588.54	25301.6	27676.58	12780.88	39788.62	30638.94	9803.254	14709.55	9987.426	16697.81
11467.42	16707.15	17744.3	13751.98	13485.6	14904.73	12690.08	20757.87	14358.75	25738.56	24436.27	25985.68	6086.591	6326.581	5548.204	9275.471
10761.6	13288.03	16130.32	12727.21	6930.986	9983.482	22108.05	21637.35	22645.25	23889.6	29063.67	36799.18	9046.933	11194.97	8237.218	9419.462
14861.91	11229.44	7049.293	18585.46	14938.07	12914.61	20114.86	14104.19	18589.62	18782.21	17592.71	37564.89	8212.945	6613.662	5216.196	8658.257
11234.43	12630.83	3898.453	16912.33	4010.103	11456.53	15865.94	10694.86	11522.82	19627.25	8376.473	18587.76	3034.255	14468.54	1861.51	4299.606
5943.376	15393.09	8459.452	21633.24	8448.355	14098.99	29725.26	25162.25	20228.67	22700.46	16019.06	19722.02	6555.468	9693.229	4959.214	6166.096
15169.26	22144.49	17223.6	13163.21	7985.572	19594.58	25028.34	34106.29	23156.96	34766.22	43237.56	35902.43	4863.724	12976.54	2100.876	15551.69
15875.6	26942.53	17192.95	13030.92	7688.094	20222.58	30467.66	40751.29	29186.14	40600.4	46662	42701.63	4640.112	15310.2	1969.184	16234.29
11109.4	20329	16609.84	17275.36	11932.97	14724.74	28772.35	32564.59	27713.6	37672.91	37671.16	39444.34	5814.388	8260.371	4549.836	10939.79
14510.33	22331.85	15756.11	12045.29	7003.597	18857.12	25865.63	34585.3	23684.23	35696.16	43689.46	37627.43	4288.898	13037.01	1808.746	15449.35
8408.25	22182.65	9413.082	12469.02	9807.488	10097.29	19027.97	20227	17746.45	36714.55	31445.95	34494.01	3638.146	4989.783	3977.59	8222.107
8798.211	12812.94	13375.16	13600.18	4940.656	6235.19	25943.5	25927.37	31889.43	36297.39	29898.93	42453.09	9937.077	13139.54	8859.6	11435.5
14842.47	26250.01	16323.87	11859.48	6892.977	18881.41	30007.28	39460.65	28556.49	38071.26	45541.74	41619.61	4175.101	14633.37	1748.389	15725.62
20309.62	21896.13	16571.42	18693.82	25655.76	17492.29	32645.92	33997.65	32388.09	36845.56	38023.29	37638.89	8216.354	11782.43	9659.185	12272.44
7774.435	16027.24	32674.69	11535.4	2766.616	6589.749	24130.28	28529.64	28305.38	37177.82	30996.19	42831.25	7879.794	6609.166	10000.05	7648.114
9161.291	9814.787	10364.8	11417.29	5670.06	5527.381	18053.69	17130.33	18462.15	16363.54	21350.3	32056.67	6916.312	10254.32	6552	6906.618
26169.09	23924.54	13657.37	20405.03	33646.47	19729.37	27944.39	27950.88	25067.17	32686.01	38593.8	31317.32	14884.9	15634.4	16236.02	9189.501
3017.456	13600.57	3881.656	7763.785	2570.612	5947.955	17185.6	19067.18	16313.59	28550.79	25313.61	30899.61	1889.792	4370.748	2161.802	5700.049
15589.13	21065.09	29294.06	10461.34	14393.96	6191.771	26952.25	33553.11	31615.53	43596.18	46913.26	34509.47	5960.563	12652.45	3993.603	14725.71
9699.168	15907.78	16277.33	15449.22	4617.609	2534.513	17824.07	10740.19	20339.31	27662.38	19370.16	22333.39	9911.318	8602.305	7574.732	9000.204
9699.168	15907.78	16277.33	15449.22	4617.609	2534.513	17824.07	13988.93	20339.31	27662.38	19370.16	22333.39	9911.318	8602.305	7574.732	9000.204
13635.96	23961.64	15053.86	10699.59	6065.944	17800.73	28988.65	37466.96	26687.31	33499.24	42986.32	38954.98	3663.671	13608.57	1505.82	15285.14
13657.43	25380.2	15293.15	10590.12	6057.652	17347.43	29414.64	37864.37	27758.05	35151.64	44129.15	40259.43	3682.98	13818.84	1520.999	15095

3464.41	9436.42	17586.18	4373.423	11875.31	5820.286	16363.35	17970.35	20573.99	25176.38	21761.59	28100.8	3039.781	4889.882	2265.057	4876.383
13658.62	25571.49	14897.53	10602.71	6068.936	17620.18	28866.87	38295.27	27094.91	36217.16	44006.93	40028.63	3668.83	13690.19	1516.131	14872.89
7693.793	3521.944	13461	10425.46	8920.02	11804.23	8202.774	15772.43	10277.06	10244.75	18100.64	16993.56	3920.927	4473.046	3512.325	6325.396
7129.599	6694.054	12127.79	6270.556	2955.077	6717.523	18672.01	22362.89	19935.13	34477.35	31943.98	25476.4	7261.376	9581.642	9455.047	7571.73
12983.88	19841.38	7342.89	11592.86	11585.8	8242.396	18509.03	18261.18	20271.92	39504.59	27506.97	32464.74	9867.548	9638.14	7756.927	7322.188
2853.158	3248.037	2155.465	14400.72	7620.099	12148.88	10506.8	277.4271	5730.295	68.93136	1536.663	12376.97	1528.033	4550.015	1305.092	4965.241
4819.61	9360.167	17682.67	7995.123	3555.808	3392.446	10549.03	8361.708	11854.71	17294.46	14053.95	16784.45	5514.772	6585.548	5327.21	4791.129
8232.243	8909.779	7554.158	9940.173	4689.501	4513.801	13901.57	3406.178	9778.81	6180.939	16561.1	19971.08	5076.482	7220.178	4993.674	4974.165
2824.475	12177.47	4501.078	4089.477	2772.888	8079.512	15853.53	23768.42	14822.88	30334.09	22532.52	26982.34	4182.731	5963.362	3631.206	5319.065
10141.71	13160.85	26189.31	8386.304	19090.84	8428.216	15447.9	15158.37	14807.26	20205.06	25389.14	21585.21	7525.462	8667.517	7107.319	6781.551
7198.76	16379.76	31020.99	10332.91	5732.392	4793.564	21829.65	20975.85	17200.1	27366.88	25369.25	24487.25	8054.334	7789.871	10905.23	7428.159
10485.1	15013.03	12452.03	14389.11	17201	13693.64	19594.7	22963.73	21337.89	29782.1	26138.91	29249.83	4927.276	7490.354	3399.407	7686.179
4567.697	21361.83	9361.168	4976.997	4988.929	13935.36	33099.59	37735.3	40611.86	35527.85	28271.4	53016.26	7713.776	11021.79	2764.086	10031.71
5047.616	2641.087	5788.873	10284.19	6426.847	9053.169	13495.93	10231.4	14975.66	12862.46	12406.19	18621.7	4071.687	6805.115	1648.541	4042.485
9761.276	1192.368	14807.35	3002.511	5401.104	18243.52	29685.69	38913.59	28701.35	38144.65	43238.36	41699.23	4756.299	13187.27	2024.085	13875.7
3902.236	17390.85	2651.991	651.3065	794.3893	5400.582	15563.9	21156.38	17100.7	25316.7	20523.86	24809.01	1511.799	3893.554	2438.252	3007.41
1605.042	4677.405	2912.658	452.914	0	1229.022	7772.697	9305.591	5821.583	13181.03	13856.16	13037.08	814.4811	310.5949	2542.049	505.4372
18955.57	14496.56	10382.13	12217.04	23239.55	11856.18	16754.1	20565.62	17676.55	26014.17	23865.95	25477.18	4388.13	7787.378	2718.766	6958.13
3704.149	6590.869	2651.935	647.7919	778.6095	4735.214	13226.53	13019.07	15475.67	12449.9	11565.9	11905.74	1473.893	3587.859	2252.555	2773.242
2297.811	3569.668	7849.606	1707.729	1941.222	2680.75	6355.341	7657.188	4593.959	10923.27	11058.82	10772.92	1942.677	8087.772	1962.794	3207.471
3994.644	1983.619	4924.623	7567.994	4635.151	6717.278	11176.36	8487.332	12929.85	13064.7	10584.04	16748.17	3291.827	5419.737	1200.269	3059.442
7462.533	16791.17	9406.268	16474.8	10128.25	6484.537	19991.66	25314.54	20773.75	23581.85	30742.43	35880.62	6294.036	11545.09	7648.251	8334.075
9825.132	11542.54	6660.388	6271.269	5055.841	5737.436	21204.7	33706.98	17443.33	34174.88	18312.16	19269.18	2497.622	8098.453	1493.276	6035.063
3452.378	7389.025	11737.68	3306.258	5920.714	11619.1	24412.14	31712.24	26874.58	36436.38	27444.2	39140.29	6331.438	12078.68	576.1262	11935.66
5711.643	2834.93	6867.768	15098.39	19296.53	16860.63	22026.78	22236.91	25209.94	16800.49	25973.69	27360.34	15030.98	11370.35	7027.56	14346.8
1892.697	12080.98	3158.152	4923.031	586.3385	5106.768	18348.68	22744.8	17831.29	31008.65	26030.68	25504.74	3012.933	3771.457	1927.934	4474.641
7151.3	954.196	7349.132	6354.859	16840.76	8105.347	12315.73	1907.351	14236.48	915.4761	5923.617	2198.955	2775.367	2346.953	762.1039	6231.889
1452.328	11042.49	2264.942	1989.605	1370.909	4395.641	13688.33	19035.64	11597.53	26213.13	17228.63	22485.96	2422.14	3912.776	2179.149	3135.365
2548.954	9143.161	13547.22	6711.847	10012.3	8470.107	8551.16	9194.74	7680.672	15849.15	15695.55	14070.59	1152.437	1337.561	2705.252	2676.733
11213.03	20287.7	4569.663	2455.6	635.7355	3827.41	25660.65	22788.27	16175.01	23185.09	31562.53	20376.03	5038.08	12297.15	2354.518	3141.386
3091.371	13343.86	6136.203	3734.556	1447.791	6272.62	23724.09	27674.47	26187.54	30547.93	28363.6	34845.2	4984.6	5356.892	3982.188	6932.131
3208.293	10117.72	7221.801	3046.065	2369.439	5893.991	10715.39	3751.043	10162.41	14557.15	14732.88	16017.14	4667.501	5846.955	1303.051	5322.115
3945.067	681.2379	10782.07	4388.604	908.5644	1872.22	4944.778	4248.135	1583.117	2291.452	11444.92	2735.536	4900.6	1529.529	2281.798	3975.484
2583.214	7399.456	3960.791	1150.364	195.6369	2021.6	11439.53	13831.22	9066.952	18239.69	18121.23	17830.42	2345.691	1947.567	504.1749	3930.714
6745.858	21174	17604.4	3178.844	1854.078	4372.254	25977.9	37148.47	23928.49	68700.1	39379.42	31379.48	5750.44	7622.241	159.4299	7902.41

3245.315	12638.17	6765.556	3143.032	1416.772	3176.458	16188.73	19836.99	15225.28	29532.9	24146.34	22267.72	2947.551	5069.955	2265.185	4491.277
3921.765	1764.302	4318.443	2651.552	1937.629	6057.479	13289.17	10179.33	12368.02	13398.84	10499.89	18954.2	3332.613	6586.114	546.2358	3556.36
5525.268	15254.77	13351.82	5269.159	2357.767	3256.153	17376.26	15374.59	11899.14	21954.74	20445.08	20836.18	3142.422	6526.013	4556.119	5948.154
3997.308	11962.39	519.9855	5686.095	3463.458	2686.23	3904.708	5112.366	2260.743	10860.77	10107.71	810.8783	1935.76	4820.451	223.248	982.8658
4523.529	9374.244	8566.403	3859.734	5238.06	6499.641	12309.83	9927.706	13637.21	16267.88	13700.47	17788.11	3432.065	6979.801	1272.924	4337.215
5733.58	18825.22	4062.064	1421.874	1410.615	7659.168	23851.71	27643.94	24388.35	35954.71	28505.79	31317.79	2402.117	5874.431	1622.239	6981.337
1677.14	717.8305	4718.978	1014.551	587.3407	193.8172	3890.458	4275.644	3325.214	1515.746	1724.951	15938.45	2336.665	2859.929	67.9065	2330.245
4517.929	22150.52	6602.79	5576.049	3769.048	9611.492	17553.65	28789.38	20245.77	32997.36	32094.28	37151.51	2146.399	5786.666	1402.914	5111.48
3191.299	1996.218	2031.157	8393.265	1701.415	3671.901	8156.335	2440.29	7143.349	1341.132	4203.419	11769.72	3411.955	4924.372	1139.774	2654.673
3023.278	18867.72	717.5486	224.4785	207.4145	1694.864	12752.32	13678.24	14877.21	23327.31	18562.83	21088.74	526.5254	1196.438	1072.433	902.4423
5070.571	9618.853	7588.222	3217.818	4650.346	6568.083	11434.5	8788.383	12997	13957.97	13359.9	18130.68	3009.669	5986.246	1047.483	3341.912
8708.908	19966.12	12274.14	3987.44	3985.475	8908.347	24700.75	29986.28	25288.49	33645.54	32453.66	37593.62	4192.639	8530.514	2659.658	9069.322
7441.859	18659.8	6838.124	1748.547	1786.726	5881.976	19730.33	22399.5	20932.13	28646.57	27771.22	29875.44	2415.652	8744.124	1181.114	7240.293
2705.298	2806.586	8225.481	1272.872	849.2646	3567.963	9999.536	12414.86	12245.32	4297.092	6715.789	11969.14	4469.806	5237.502	276.3117	5139.404
1898.183	3952.531	7696.526	1756.851	4251.623	12303.21	30697.99	35648.16	38380.79	32434.99	19391.27	51123.16	3708.254	9703.664	274.0802	8839.453
1857.304	3558.252	6400.9	1761.008	3018.739	5951.414	13927.98	22094.57	15188.54	32284.94	18548.28	25531.08	3582.054	6938.422	311.3945	7229.31
3390.79	664.2986	3808.895	3036.855	20014.96	4571.551	8854.513	1201.957	11764.28	644.6495	3885.162	1443.529	1285.751	1001.419	431.8218	3277.471
2285.72	2445.38	4604.268	2734.672	2417.212	8392.596	10671.32	12232.2	9958.328	16028.57	8828.143	15902.82	3755.274	3480.805	639.063	4069.702
3468.853	2053.833	9878.912	943.3025	1391.113	12725.57	25465.01	22646.55	24093.54	23092.61	29415.69	29600.48	8771.818	10666.83	10885.84	7987.163
1275.775	540.9058	3758.972	771.2133	442.7612	146.1284	3031.621	3313.698	2589.604	1144.57	1305.116	13422.14	1823.681	2309.304	50.97414	1853.908
5627.765	19473.53	3637.831	4073.766	1189.102	5378.06	22840.84	31914.8	19579.54	45880.41	33050.01	26167.77	2720.205	6497.421	2241.523	4572.64
1682.719	9856.544	2364.873	404.4602	479.1097	4992.177	11940	16759.59	10400.45	9231.037	20694.5	18241.12	1560.422	2856.781	72.29172	3035.092
1659.992	7843.039	2387.609	404.4529	477.5642	4948.238	11318.31	15012.58	9901.494	7053.282	18464.81	17640.1	1565.516	2725.623	99.55717	3006.456
2145.779	947.0814	15102.11	1426.795	724.504	2506.144	1347.937	1790.826	2389.911	84.38815	6973.164	1185.756	0	0	202.675	1151.378
6874.273	15688.32	9263.698	4310.73	2676.077	8681.018	26060.55	31035.74	27442.69	35220.31	25240.41	29473.72	4635.294	9031.712	2792.506	6963.656
3918.505	9871.156	12892.03	3503.607	788.9407	3433.263	15946.56	23648	13988.62	35221.88	23997.21	18129.73	1674.437	4172.447	153.1488	2900.818
2953.507	13904.97	987.2453	391.5814	776.3043	133.9991	8484.718	8696.877	5639.347	12226.89	13251.6	9596.802	1240.436	755.9226	1646.27	1145.751
1612.841	2546.62	9013.274	2617.993	726.1721	624.1065	6916.325	5257.277	3421.536	3082.204	11691.73	8734.602	505.3772	1902.679	1249.842	892.987
3124.561	17194.64	4511.339	3377.207	1076.002	5285.822	25677.44	35312.03	26059.43	56710.44	25925.77	45719.55	2618.809	4202.491	258.8799	4923.011
1504.482	13339	669.0412	165.9401	74.53144	767.2142	5492.998	7419.8	4435.802	10254.09	11647.75	12019.21	210.8489	689.441	797.1022	480.853
2754.39	10965.92	7463.014	1023.341	1171.547	1728.708	3730.181	7029.732	1377.339	15092.23	9539.034	2393.315	2203.376	5513.768	3928.587	2304.189
2741.811	17834.55	876.3796	291.3234	273.2966	2094.861	13158.35	16444.3	14105.06	23656.11	19591.94	21503.02	751.3794	1595.999	1279.764	1284.44
5697.624	14949.17	6485.583	6912.165	1644.063	8830.931	17790.74	23874.55	16018.57	27420.08	33332.39	27981.96	2055.587	6299.83	3053.427	4821.499
3168.442	22922.01	3878.223	1319.151	1331.766	5873.194	21202.95	33080.04	20765.35	47179.08	35106.25	38056.29	2489.191	3676.381	1747.388	5025.369
1648.011	9684.569	2086.053	1049.004	604.522	3268.047	11071.77	15823.93	10826.93	25967.26	21531.38	17153.47	899.7081	2275.425	948.8822	1436.192

5691.912	14229.14	5592.839	6201.559	1635.164	6363.035	14828.22	19990.03	13983.84	25307.89	23910.84	23314.2	1991.885	4638.889	2323.213	3075.65
5249.413	14813.05	5879.746	6204.284	1449.379	8009.17	17204.91	23238.17	15347.57	27450.33	33673.24	27063.41	1825.865	5822.899	2745.358	4380.851
5249.413	14813.05	5879.746	6204.284	1449.379	8009.17	17204.91	23238.17	15347.57	27450.33	33673.24	27063.41	1825.865	5822.899	2745.358	4380.851
5249.413	14813.05	5879.746	6204.284	1449.379	8009.17	17204.91	23238.17	15347.57	27450.33	33673.24	27063.41	1825.865	5822.899	2745.358	4380.851
65.49387	169.9778	3421.07	5597.897	6697.488	2960.498	1238.209	826.2212	1277.036	1350.429	940.0947	3067.383	144.7557	130.5275	6.065764	303.5618
4260.452	26618.38	4789.765	284.3293	491.5777	530.8758	9996.037	24766.59	9722.221	58658.01	32992.65	17000.1	2328.592	2190.448	70.1805	2540.63
2627.146	14168.91	3295.836	1103.73	1111.932	4844.614	18655.86	21908.62	18773.76	14225.84	23872.72	25090.22	2107.655	3147.103	1405.569	4091.673
2448.368	3286.513	2599.042	1762.136	698.5681	2561.036	6403.156	4402.527	6038.39	5748.73	6997.892	13682.18	1719.71	3667.068	650.4665	2643.011
4174.534	14229.14	5319.743	1226.429	1138.849	6363.035	14828.22	19990.03	13983.84	25307.89	23910.84	23314.2	1730.384	4638.889	672.06	3163.528
5201.314	17847.46	12582.78	1679.888	1740.789	7414.736	21624.32	25506.07	22738.21	31402.71	22591.78	32516.23	2411.953	6434.616	428.5903	6768.611
144.3212	327.6819	236.4628	36.60707	269.0613	36.84246	1653.856	1139.902	1139.071	4104.797	1292.262	918.8499	800.3427	1016.551	108.2635	1002.704
1737.731	967.448	5329.055	846.1642	199.4715	43.52592	241.7548	909.9733	178.8192	90.98939	3552.307	168.8993	1892.124	1390.322	106.8148	3564.138
358.0637	383.5447	1600.755	773.2923	329.3537	112.4824	1539.679	305.7029	1698.023	1345.356	0	973.7476	589.2491	70.08555	373.6593	1404.862
4078.083	12411.18	4620.105	4987.553	1112.194	6571.492	14918.8	21423.88	13655.84	25404.83	28662.78	23344.3	1407.351	4606.149	2129.982	3603.573
8443.805	19422.76	6968.98	2009.981	2745.157	10322.42	21376.06	26422.01	19404.92	27641.39	30364.39	28206.37	4499.067	8743.722	1855.358	8169.64
2887.067	11920.46	10776.38	1518.363	4528.722	2459.58	15502.38	14371.22	14462.17	13329.39	19671.4	16465.85	3156.497	2988.399	4859.137	3089.801
3104.412	4358.619	8906.648	1359.243	1325.738	3488.801	15719.78	20613.42	16572.04	29439.67	17908.42	21747.6	1905.927	4812.359	127.0541	5756.365
4073.694	11675.43	3946.903	4437.244	1106.086	4586.073	11922.58	16981.99	11513.56	22764.41	19004.1	18667.33	1362.598	3307.395	1602.837	2202.17
4256.608	12799.55	5379.122	2341.507	1422.225	6128.51	20509.53	29209.81	21350.49	33014.91	21407	24776.77	2957.816	5835.236	1735.693	4543.148
760.3199	832.7186	448.876	8116.89	1412.386	7643.673	4282.226	655.8803	2564.336	226.0812	1457.376	2880.754	0	0	0	726.1978
0	0	0	504.9868	330.0624	75.42501	1658.127	0	278.5816	482.0985	2533.707	256.7995	0	80.13986	0	1111.455
6974.862	22050.95	5182.342	1637.335	2216.2	8576.329	26831.58	30512.97	26212.31	37252.94	33931.64	33544.54	3873.844	9226.691	1501.059	6921.944
1869.147	8788.543	1740.865	1022.239	604.0818	2402.163	9616.13	14387.17	9995.113	22159.89	14622.47	14951.32	717.1547	1894.645	637.3019	1242.62
96.09089	630.9123	2262.091	339.9148	1016.787	4228.14	15297.44	17765.81	17250.63	19459.96	10942.01	23521.31	1738.988	3607.733	162.3382	3312.566
3369.006	21174	8761.166	1098.67	1027.567	4372.254	17349.08	23527.76	18239.14	37624.08	19219.69	27929.84	1617.198	4552.223	101.739	4876.547
3730.477	17756.45	7577.75	1419.357	1072.213	5246.975	18019.82	22989.34	19040.32	30702.29	19609.62	28312.66	2162.406	4831.855	469.8395	4530.263
1521.668	3151.604	3040.45	205.027	271.148	49.50703	4430.928	11183.44	869.2396	23877.46	13621.89	1427.141	485.2155	1014.287	247.1186	1623.145
714.6057	302.0399	405.3545	5165.644	4899.35	7730.772	1964.578	158.2511	1347.166	192.3999	3738.05	2359.617	450.8793	716.2108	506.2803	684.8642
2039.293	17909.99	2119.856	750.4676	539.3763	3440.927	15755.86	24789.14	15920.37	39678.42	23803.76	29920.89	1294.994	2490.839	241.9369	2852.571
1931.001	8838.079	2969.384	674.1703	701.1705	3269.262	10985.38	14599.89	13127.76	22408.91	16328.75	19328.76	1053.787	2480.132	375.7099	1938.434
753.9051	2457.292	1148.377	184.9261	143.7276	238.4314	1396.802	4833.446	242.0506	11002.82	5913.792	74.39123	1091.754	3871.81	844.8345	1254.931
966.378	1350.538	1047.322	176.9372	330.3345	155.8767	3584.037	920.6937	1197.942	4369.408	3069.893	345.0585	271.8816	2578.903	21.20426	1068.798
960.8162	1289.396	1063.128	176.4526	335.2725	156.6783	3778.174	926.9517	1218.568	4403.149	3085.655	345.944	271.8703	2700.136	21.02945	1083.924
5321.707	18375.73	10168.29	526.7243	683.5059	3969.468	21208.21	25169.96	24501.97	37928.07	22081.12	28943.37	1927.259	6585.935	265.1202	3872.981
2777.916	2563.292	3372.222	953.1556	3979.737	1059.105	5381.405	839.9063	1467.687	934.7683	6208.426	1089.222	2678.156	3701.078	767.8929	1723.539

1884.972	123.995	3928.947	334.1522	900.3876	6140.15	19203.94	25285.35	21258.02	25814.48	21634.98	30498.51	1997.887	4221.54	904.2512	4463.885
122.9609	0	36.15978	386.8118	150.671	45.64554	1623.13	39.56143	301.0109	180.9109	8971.538	2090.378	0	44.49084	1266.347	954.5151
1731.363	12997.29	1078.114	301.5958	274.6128	1918.662	9337.486	16141.92	10815.97	32505.06	18114.03	19559.26	815.2723	3215.161	133.1949	1792.85
3502.956	16061.91	3032.433	753.6028	912.0714	3896.878	16292.09	21042.36	18653.25	29629.93	23272.27	25744.89	1439.454	2946.112	103.2616	4005.148
894.8189	2157.524	2853.988	425.226	458.2163	2421.717	9232.37	13473.28	9912.06	20628.67	11786.9	15308.86	1340.765	3415.977	295.2254	2058.216
3350.575	16198.31	2877.146	714.5882	864.5401	3814.032	15858.85	20800.68	18112.3	33135.82	22635.63	25369.24	1378.558	2818.611	97.66915	3840.533
3918.505	9871.156	2217.558	1353.757	710.0247	3433.263	15946.56	23648	13988.62	36333.86	23997.21	18129.73	1674.437	4172.447	120.8516	2900.818
4174.534	14229.14	1389.338	1226.429	1138.849	6363.035	14828.22	19990.03	13983.84	25307.89	23910.84	23314.2	1094.193	4638.889	92.867	3075.65
4174.534	14229.14	1389.338	1226.429	1138.849	6363.035	14828.22	19990.03	13983.84	25307.89	23910.84	23314.2	1094.193	4638.889	92.867	3075.65
70.45574	0	132.4634	570.8969	0	0	6756.351	3348.837	8966.89	1017.712	58.99266	487.0567	793.4323	320.1231	1884.234	2177.033
1969.499	11996.01	3762.952	2585.68	3263.633	5227.763	19727.24	30574.86	21452.19	37701.73	19120.67	34308.5	1215.647	4342.89	283.0038	3615.588
1969.499	11996.01	3762.952	2585.68	3263.633	5227.763	19727.24	30574.86	21452.19	37701.73	19120.67	34308.5	1215.647	4342.89	283.0038	3615.588
541.7569	257.4564	2708.023	348.7754	148.7219	476.0619	2244.236	1554.306	1033.26	4661.596	5053.763	1311.127	2420.292	1837.516	2634.9	1443.387
970.4575	8914.239	0	2309.443	6762.368	1143.326	6875.366	13204.22	7138.683	23201.98	11087.77	12078.54	362.5749	0	80.51057	831.5473
21588.4	25110.66	2652.382	118.8733	205.1906	1060.405	6721.024	13194.6	7386.922	21308.32	17802.56	13890.13	680.9059	2858.341	165.214	606.288
2036.818	8809.795	2303.822	2349.422	495.8624	3221.855	9462.063	15458.14	8652.431	21630.34	23519.44	16086.33	643.8994	2432.282	998.4913	1786.484
1556.795	11064	290.05	196.3274	318.964	1152.403	5181.358	8459.024	4170.366	22668.04	14609.91	20892.58	398.2017	443.0013	653.0203	828.5936
4037.414	15889.11	2118.308	713.2358	876.3878	5346.701	15604.27	19255.55	16729.21	22955.03	19710.66	23211.66	1623.402	4082.548	1043.257	3066.784
2858.678	2603.383	556.0031	2768.337	5003.892	1833.742	644.523	0	96.83777	353.5308	4614.29	1120.104	0	573.7253	0	610.5584
964.1733	88.24757	2007.96	130.4657	598.6736	1440.656	11175.72	13948.65	13048.49	8034.602	24300.68	14311.76	726.8183	1550.958	2768.374	2154.511
994.8044	62.14083	2160.415	168.1287	467.4677	3615.454	13851.73	18874.14	16391.82	19510.95	14119.64	24078.98	1197.993	2407.009	548.9698	2542.85
2595.563	16465.63	1931.913	898.5625	485.5831	2841.722	16827.01	25886.54	18790.64	42244.64	18271.68	32876.59	1171.144	2850.292	74.83166	2718.944
3738.68	14546.97	2573.11	734.7401	746.8815	3638.027	13906.26	12351	13970.64	17328.8	22006.8	10576.02	1743.969	4944.88	105.7503	3628.667
3871.532	21198.45	3077.788	762.2756	1049.086	6439.047	24268.51	35224.58	25262.59	40431.65	37801.7	35197.88	2021.565	6010.495	741.9027	4391.58
2114.49	10295.18	2186.324	536.4074	545.4354	3350.196	16036.18	24835.81	14713.54	38601.94	21465.57	21115.85	1427.14	2931.225	53.83411	2724.015
2930.255	11675.43	939.1308	828.807	766.9482	4586.073	11922.58	16981.99	11513.56	22764.41	19004.1	18667.33	739.8039	3307.395	61.99486	2202.17
1594.457	10608.27	2454.517	766.1298	849.2854	3364.675	9598.862	16394.77	11884.41	28535.4	13200	18589.38	953.5382	2428.633	271.9487	1964.219
2435.41	16734.7	2712.356	719.8827	635.6815	4368.944	20027.71	29827.43	23421.63	45698.59	23969.61	34978.58	1603.537	6369.328	40.16842	2784.271
1361.019	9409.621	1374.181	492.6686	471.5269	2306.9	12297.64	12458.03	12867.07	14068.89	12476.34	16295.16	1301.796	2606.83	423.7491	2506.012
2044.119	9723.703	2013.943	453.3638	497.4011	3121.013	14691.2	22687.79	13555.09	35354.46	20918.02	18647.79	1321.459	2770.428	46.08358	2539.318
2607.173	17371.73	3748.828	471.6922	707.5993	4860.315	22522.93	31563.57	23491.63	44400.3	29924.35	34215.64	1750.381	2761.644	450.5459	3960.289
2588.582	16395.34	3744.114	469.7731	704.8581	4485.941	22139.21	29011.36	23597.47	42075.35	29100.1	33108.53	1700.563	2742.446	445.3351	3906.005
1257.296	9181.414	1563.815	298.5432	336.0096	2402.163	9616.13	14387.17	9995.113	22159.89	15377.52	14951.32	631.6401	1894.645	644.2243	1232.799
997.3762	2071.986	402.0298	832.231	107.6324	505.1782	1227.015	1614.397	624.2333	390.7564	102.78	343.7144	94.95234	137.5641	5.038108	34.31174
27.71047	184.8851	750.749	109.1862	392.3788	1699.318	7640.217	8775.038	9210.108	9269.476	4192.574	12756.54	697.7315	1505.102	51.81967	1376.782

1950.647	15954.77	1423.128	480.8631	185.2579	1273.373	7519.288	13357.4	9291.414	20703.21	8661.367	15992.11	896.063	1242.306	47.26727	2097.197
1131.478	981.6391	2093.257	679.8026	371.2777	0	5362.472	1401.092	2008.025	115.4394	686.8468	418.6409	291.3339	1283.354	69.04237	12.13617
1364.668	10862.4	1750.617	433.5995	305.0625	2755.363	12495.6	20305.93	12203.55	31181.94	16444.49	20609.3	892.5788	2470.136	28.81851	1925.431
2313.489	16148.47	3323.097	413.3656	620.6698	4317.011	21310.51	29459.07	22390.75	42112.58	27723.99	32931.15	1544.807	2465.92	395.3675	3534.673
648.8168	58.90384	1357.128	87.04432	400.675	971.0339	8155.575	10572.24	9632.73	5816.645	19255.85	10631.23	488.9405	1054.956	1930.168	1473.828
551.7531	4822.132	1515.788	258.0372	301.1433	2168.22	4068.776	6303.634	1763.333	15332.31	12028.27	10081.37	690.1391	1775.865	18.19429	1362.672
1167.865	8920.311	1037.316	397.9524	349.9096	2492.82	12109.97	12562.17	13249.34	12357.22	11087.39	17379.68	1104.272	2741.76	314.2493	2576.279
1360.28	11661.93	1496.923	231.9316	513.3007	3754.489	15931.44	19860.29	18576.91	24977.58	18191.66	29722.17	1122.061	2600.26	64.47532	2643.686
1477.202	4620.406	546.0339	660.8028	132.2261	303.1229	1211.019	1997.955	842.6304	6190.957	6385.487	6023.012	346.3711	516.6711	0	475.5386
113.9411	497.7673	847.8416	208.2485	96.13082	25.96735	71.91242	146.7993	17.64561	110.8233	827.2628	24.86826	506.5146	1184.399	69.14999	972.3873
3142.318	15634.23	1368.116	364.6915	154.2145	838.6756	3248.976	2803.52	3256.235	3598.569	7945.125	2532.679	353.4424	1799.759	392.8534	1198.004
223.8777	179.4513	190.4067	36.57476	24.17249	91.77743	678.7499	52.28441	43.67706	218.9886	163.4405	27.15136	233.6971	1481.123	0	217.8925
1148.697	8606.517	1956.137	288.9151	781.1285	2601.691	13461.09	20717.57	14133.08	29641.79	16319.85	24168.57	948.2012	2100.362	645.5535	2428.492
0	0	0	159.9101	509.4856	0	0	0	0	0	0	0	91.98354	103.2728	0	0
1029.461	9429.899	1049.222	234.3107	272.2001	2013.428	11940.27	18560.82	12540.09	29956.74	16651.88	24438.03	1132.009	2933.762	26.67104	2291.772
63.19185	13.14516	2272.843	239.3611	547.871	3251.224	9215.281	15115.85	10429.26	6436.678	8317.402	14313.92	1285.492	3375.913	28.25969	1933.201
1079.519	9889.884	1682.512	237.7731	258.4998	1695.975	13132.89	19869.68	13568.81	33002.37	13473.87	26696.19	632.565	1605.217	16.27282	1365.334
335.6291	3396.796	1367.187	174.0213	377.5677	2006.624	8508.348	11194.1	11649.31	18463.39	10673.65	16933.69	709.6341	1760.262	189.4031	1515.729
366.9852	95.51421	323.1084	59.45735	32.11655	0	420.0144	127.7064	140.3321	19.21975	54.07501	62.93319	126.3761	0	38.39841	103.3209
366.9852	95.51421	323.1084	59.45735	32.11655	0	420.0144	127.7064	140.3321	19.21975	54.07501	62.93319	126.3761	0	38.39841	103.3209
366.9852	95.51421	323.1084	59.45735	32.11655	0	420.0144	127.7064	140.3321	19.21975	54.07501	62.93319	126.3761	0	38.39841	103.3209
25.34998	0	110.916	234.2749	85.60323	153.6705	1581.792	1329.209	1388.737	2173.219	1172.363	1614.61	213.8144	161.8141	0	684.6329
1880.602	12571.5	1314.439	328.7143	555.0368	2225.37	11632.3	20197.9	11318.68	27689.89	18021.54	18930.5	713.088	1897.976	53.07344	1617.626
1252.446	8082.657	378.4775	309.6326	573.3184	600.2576	2403.142	4045.373	3443.86	7611.596	4072.027	6620.908	172.7453	598.6362	237.5186	515.2248
13.35035	0	919.8032	63.99972	325.1016	1700.737	6832.484	9090.216	8375.134	6713.838	4032.015	12965.13	668.7316	1767.3	7.891193	1564.439
46.80458	0	1167.459	248.186	333.0756	172.4743	8632.356	12977.32	1600.624	15728.74	8006.234	4054.961	612.2618	1304.926	43.25947	2461.502
39.9905	15.34837	2713.558	190.8696	170.1542	4144.687	5368.763	5829.869	5490.5	14051.74	3389.638	14179.45	420.9002	2215.188	29.60473	2241.688
1362.722	11432.28	1954.222	237.2408	357.1859	2629.22	14998.22	22522.42	15894.17	34279.42	20069.28	24616.81	906.995	1469.77	228.3185	2135.113
0	0	70.12967	10.32061	0	0	585.0101	180.807	443.214	52.67616	513.7466	14.92418	746.4531	99.72852	363.2929	9.508848
524.4925	247.5072	2432.718	0	207.0658	0	0	0	12.82861	11.28028	1063.917	0	74.79776	13.85625	0	1273.429
1380.628	1642.74	982.1825	216.5801	85.78479	0	3152.147	2040.547	2436.698	200.451	1163.605	713.2737	201.4171	977.8514	146.1451	21.00935
0	0	0	21.74958	16.01441	0	17.58658	0	0	41.56046	32.60977	58.17912	0	0	0	27.15588
1416.262	9926.596	977.2941	180.8373	368.4061	2132.698	8527.836	13741.49	8777.8	22625.44	15912.83	14288.8	588.2908	1764.157	668.2413	1260.774
2046.569	7888.22	1144.401	354.7518	522.8764	2675.646	13754.09	21394.19	11870.09	32202.52	20334.77	15939.56	930.9584	2100.184	29.80596	1593.326
486.0282	37.59597	947.7397	135.2231	118.2129	53.9628	255.5956	1225.649	163.4249	1247.973	4540.316	4851.688	526.6729	1812.632	852.0371	593.2894

2403.675	1923.708	10493.98	0	0	0	2570.797	48.10301	332.7084	0	135.1651	0	685.8567	0	0	247.2932
514.2429	1452.992	828.9716	118.9328	256.7694	1694.18	8397.309	8247.845	9557.625	11291.11	6347.17	14699.34	715.3603	3180.926	11.60916	1321.35
973.0051	3847.059	1923.178	252.9639	284.0246	1791.213	9613.423	13026.9	12083.95	17807.34	7631.529	20169.5	634.2504	1808.171	19.89077	1443.3
1321.145	8267.15	763.0593	359.1205	142.0012	472.5975	5698.503	5001.897	2301.005	11238.01	11100.56	8760.705	185.2975	878.4979	68.51938	74.99737
918.6634	411.189	604.0717	0	512.796	132.46	362.0349	0	0	569.4497	4666.727	213.5151	428.151	2049.259	101.4345	693.0625
220.7677	12.45633	684.8498	37.5847	134.9988	1042.489	5316.857	7125.681	7525.068	6902.776	3954.888	10426.69	384.5797	663.1113	145.6579	711.8595
242.625	953.2138	110.7542	108.5548	69.66321	0	2147.416	755.4792	1597.875	193.9659	1042.359	659.9149	144.9159	654.8427	0	20.67986
517.3104	3066.531	385.7941	130.3961	311.1604	65.80753	4818.099	13898.86	470.2094	26762.69	16971.13	1151.834	149.9338	641.9958	50.79491	490.4138
1022.845	7563.359	1535.655	132.9001	228.9845	1780.439	9046.418	12798.69	10260.1	19961.35	9881.769	13729.16	700.6545	1379.715	10.64385	1415.088
0	0	0	0	5.196305	20.64852	20.58627	0	0	103.5031	306.4504	287.5304	0	0	0	70.92184
2396.413	12566.81	1087.451	256.8083	254.8913	2719.879	11230.15	17544.66	12205.32	28193.32	22957.59	21496.39	748.5728	2024.153	797.8099	2002.643
971.3116	750.2498	916.0857	183.0719	79.40383	33.36135	185.8486	695.0871	138.3331	70.66224	2388.433	130.8848	399.1757	789.9145	18.88249	827.6375
1255.255	10196.73	724.6058	255.472	143.4369	1379.524	5467.208	8148.823	6319.737	13102.11	7039.668	12032.33	397.9863	1168.193	0	1057.524
1570.86	13151.76	415.9162	2097.32	16744.34	4470.213	6352.717	7200.14	5858.931	14817.16	10485.22	10024.01	309.6776	799.95	469.8412	710.9852
0	0	0	0	0	0	162.302	0	9.253331	0	1927.835	48.20044	0	0	0	0
2335.752	12281.36	321.5735	308.0667	10.87215	1548.124	7890.101	11015.36	8896.048	15397.15	5827.359	14175.62	267.6606	488.7127	0	451.2112
1473.135	7039.45	921.5583	211.4546	324.2903	1416.415	7176.067	9448.952	4837.255	14657.19	11164.96	11123.75	761.8591	1561.299	15.44733	1077.835
1174.946	9801.433	983.6482	217.3345	234.95	1976.985	7468.745	11134.65	8880.495	13703.49	12527.66	15994.51	266.6158	715.0878	325.0373	999.2955
175.5926	42.96718	170.2263	28.81229	15.00203	0	190.4279	57.03609	62.75451	8.546029	24.07702	28.02759	60.87042	0	17.27625	47.81877
0	0	88.27828	2112.528	189.9139	1753.497	471.8232	0	72.2947	0	0	0	14.65265	10.32112	0	1151.144
0	0	0	0	0	0	489.5094	46.29201	243.3474	29.15797	0	59.41711	33.201	0	51.28779	284.9586
1505.365	1496.886	668.2741	96.89328	201.5706	1668.263	5330.677	5741.731	4163.219	8028.078	7339.687	1679.057	582.4892	1262.276	19.0812	1028.899
981.2987	8277.934	657.781	185.819	91.32811	422.5472	5600.159	10072.41	6329.319	17179.31	14096.96	13595.87	281.0388	564.8041	1243.085	868.3314
47.14028	7.915472	711.0468	53.23056	124.6258	1182.254	5915.485	8514.174	6643.936	9100.192	3968.476	9113.131	525.1505	910.6784	9.416329	601.2456
0	0	0	51.77892	149.0668	143.7804	3575.985	8187.083	7585.068	0	1132.396	791.811	455.5321	571.1566	0	0
803.863	6575.69	789.2245	67.72631	145.2522	1214.741	6952.283	10424.78	7694.809	14733.92	7196.069	10200.27	495.8051	1005.859	7.853525	887.9908
803.863	6575.69	789.2245	67.72631	145.2522	1214.741	6952.283	10424.78	7694.809	14733.92	7196.069	10200.27	495.8051	1005.859	7.853525	887.9908
0	0	0	12.17846	127.1207	561.6399	386.0284	112.9926	4585.168	2956.801	0	356.2289	0	0	0	0
9098.142	7331.841	1116.034	1150.929	80.71488	1203.442	602.2032	0	83.41497	1383.873	5022.414	532.9295	86.43672	144.5439	0	627.1408
12.04467	0	193.2101	13.88752	11.01541	492.4636	3249.737	1085.466	2653.303	687.8439	2257.787	1361.219	160.0768	284.7348	0	255.403
1318.342	12007.03	240.5924	129.782	59.09592	1002.691	2186.916	2901.354	2555.812	8194.028	1431.894	8010.761	224.3741	712.3547	17.98514	227.27
1348.953	12035.16	571.2544	116.3326	136.5543	862.3678	6224.957	11390.5	5117.998	21372.04	10658.97	8058.869	306.7372	1061.194	0	864.6451
1375.825	12210.07	208.9547	121.7446	54.35306	1006.48	1917.554	2489.624	2220.91	7365.18	1221.598	7401.707	206.5268	667.2825	18.00078	196.7386
40.64117	393.5973	66.47864	0	34.25372	0	198.5494	28.8899	33.84757	21.97202	1473.429	28.37378	58.71248	35.30814	0	161.239
16.48549	12.3413	615.2848	36.57479	70.03479	28.7739	159.6688	590.5522	118.7651	60.43953	1818.719	112.2441	357.1686	510.9983	0	578.5647

858.5797	1019.976	1820.914	5792.067	886.8175	3941.018	2347.94	2414.633	2057.392	64.84407	4265.54	1867.581	398.2611	2697.856	449.711	982.8184
982.8018	768.8883	5077.183	0	0	0	1029.565	18.05669	125.8827	0	50.79911	0	262.5395	0	0	93.68456
5.57852	0	126.2127	6.662842	68.87651	324.4625	2580.642	1083.488	1774.672	1197.404	1498.727	1428.403	157.1516	197.5241	0	162.2667
934.0708	949.2972	410.1914	58.24922	121.2751	1101.516	3864.439	5800.304	2982.654	9820.286	5457.477	1188.444	360.9139	829.0727	11.46003	651.3298
1243.539	9592.682	370.5018	77.5263	218.4082	564.1328	10026.18	17355.54	12869.75	23837.01	16280.22	9029.201	218.4128	1834.203	46.53412	1102.912
9.046588	0	185.5221	10.79002	69.30476	318.5313	0	16.64319	82.36293	27.09814	345.6246	26.57255	196.2771	285.9504	0	236.1505
129.1072	0	285.549	16.38302	93.35906	395.3871	2372.234	2615.659	2669.025	3560.5	1856.869	5282.142	100.2959	401.8307	5.159981	422.1234
1305.017	1111.128	276.0938	138.3715	149.7638	707.7621	4918.46	8176.328	5604.489	6248.353	8036.631	8967.243	216.2686	533.2985	0	532.4815
0	0	0	17.06231	96.03661	35.42085	1154.575	3716.332	4827.176	0	277.5443	183.1605	346.457	257.6929	0	0
382.3731	0	369.9782	16.96566	54.44052	422.6748	1323.556	2798.465	2230.52	1283.022	579.3743	1735.623	53.12998	1578.344	0	464.5069
7.423142	0	127.7668	8.663664	6.854082	329.2985	2328.533	1300.409	1787.894	1457.753	1789.348	1762.836	107.7978	196.7361	0	169.4724
3635.973	16947.11	1173.282	732.2856	195.3799	2189.936	5955.244	9989.681	5988.692	19652.98	21831.17	18819.68	597.2482	2570.879	889.9466	3014.363
366.0397	94.47941	219.0164	46.56827	0	0	0	14.85783	0	0	81.13312	52.13214	0	0	48.78028	29.42352
1539.104	12900.84	396.8372	134.5294	146.2406	497.4178	5565.167	8160.472	4251.222	15127.33	9408.829	8145.705	172.971	468.611	0	430.4661
953.3549	7968.295	252.8037	59.67343	178.6516	169.9265	3311.387	6273.409	1540.684	14319.79	8363.825	7419.218	137.2653	1348.709	5.66701	544.0734
0	0	0	74.84814	20.29905	0	15.30595	0	0	1859.696	122.6625	2004.235	21.7608	662.09	0	297.9232
346.727	257.0536	66.12793	0	38.80648	0	46.79909	0	30.08699	0	30.66164	0	84.39412	1193.252	0	229.1885
344.0374	257.7807	65.44037	0	39.99337	0	47.00263	0	30.37286	0	30.67367	0	84.81232	1261.602	0	232.044
0	0	41.23393	0	0	0	337.9271	0	34.43089	0	0	0	0	0	0	174.3299
38.16206	7.915472	150.7032	42.03694	47.90544	359.6388	3615.289	5488.444	3298.758	9100.192	3968.476	6433.156	103.3801	216.9371	0	219.2041
0	11.66513	0	0	0	120.7336	0	0	34.4348	0	0	19.66864	0	0	0	0
0	0	0	0	0	0	0	0	0	0	0	0	0	0	0	0
0	0	125.2248	13.31053	39.14486	286.9588	2065.046	2410.28	2434.782	3065.801	1567.352	4660.873	73.02994	254.3628	0	343.3698
3451.087	17159.36	713.4154	468.1002	144.9703	1923.497	4641.238	9208.612	4882.056	19221.26	21686.25	18041.47	366.8181	2220.638	2085.115	2199.398
0	0	82.7602	4.470855	34.85797	239.1828	1239.508	1420.558	1546.751	1855.511	857.3654	3110.397	58.2018	195.3476	0	295.1083
0	0	82.7602	4.470855	34.85797	239.1828	1239.508	1420.558	1546.751	1855.511	857.3654	3110.397	58.2018	195.3476	0	295.1083
101.8726	218.1712	0	20.52051	3.839142	0	1024.039	2266.396	54.53843	4900.204	2593.289	100.4064	26.52552	0	0	0
102.6738	0	22.44635	0	43.09877	0	26.78642	0	0	0	72.81289	0	0	40.09125	0	57.96877
12.7007	0	55.25534	24.53783	51.54345	30.43228	2576.553	3378.341	3309.96	3262.769	1543.676	5226.651	70.45778	96.08864	0	177.6566
0	0	0	39.66924	59.82246	18.69062	1327.138	118.9744	89.14039	545.0861	310.1965	319.9115	0	0	0	0
1869.627	11801.19	451.8625	249.8879	108.6884	1247.485	3567.753	6729.322	3829.498	14284.76	11972.48	12776.09	244.5423	1229.926	595.376	1184.112
186.0699	746.1055	206.0802	0	134.7734	432.2727	4400.51	8567.564	4385.481	15126.91	7745.022	3867.916	184.3376	148.9334	0	436.9322
186.0699	746.1055	206.0802	0	134.7734	432.2727	4400.51	8567.564	4385.481	15126.91	7745.022	3867.916	184.3376	148.9334	0	436.9322
753.4607	6999.559	337.3155	62.34148	41.85697	827.1322	3932.473	6823.396	4573.558	7769.037	3120.996	2047.149	83.24624	224.2369	0	0
0	7.777957	0	0	0	80.99744	0	0	22.96842	0	0	13.11465	0	0	0	0

7.423041	0	97.30886	65.9817	58.16859	365.2218	2559.751	2578.276	3748.637	5378.119	1547.203	7226.847	110.0833	343.5026	0	467.1521
0	0	241.6745	0	72.70363	23.04617	3578.213	3628.044	5120.881	3646.26	1726.725	5626.097	98.18697	191.1365	0	223.2363
0	0	83.17596	0	18.32167	84.96402	989.3612	1426.706	1404.084	1678.514	767.5142	2690.405	26.16778	122.4178	0	8.885538
0	0	252.6015	0	86.47392	169.2466	3752.974	3642.578	5477.489	3676.102	1776.482	5669.876	103.5368	188.5804	0	243.9504
769.4372	4796.607	0	31.85714	86.29448	0	1157.244	2969.951	81.42115	8420.633	4255.38	194.6247	0	13.96908	0	0
16.64527	0	245.3019	36.59589	34.46083	0	18.23655	0	0	0	22.94586	41.25351	52.15578	28.1598	0	71.43573
0	0	12.52183	14.83417	5.69856	42.84373	1127.886	2019.736	470.7558	3168.844	1616.375	1646.229	66.30263	40.12521	0	120.1386
1920.01	11172.72	230.9525	131.1321	201.2526	1484.49	8714.773	10960.53	7442.032	18815.89	11139.32	11364.4	373.8344	16.4979	0	1169.64
31.35139	0	87.62776	11.89742	50.99986	0	22.73743	0	0	22.86498	30.12018	15.27668	21.01616	62.29181	0	118.6741
0	0	147.8565	0	51.15057	98.02317	2373.72	2267.48	3609.433	2235.836	1057.527	3569.534	61.36818	110.3726	0	145.0401
0	0	216.9752	0	46.0154	20.94273	785.0606	1857.035	146.0718	1976.006	943.7952	286.4949	156.8915	149.8557	0	269.2927
80.00247	145.0682	338.5453	11.17416	20.47155	290.1241	222.4133	0	24.81188	36.028	1064.88	0	20.39332	23.08779	0	217.0388
4.57424	16.93403	93.50097	0	13.96808	570.3674	3487.693	4363.196	3693.149	6613.712	2468.756	7125.623	133.5489	280.2696	3.211753	369.5345
27.46938	682.7647	156.4446	45.81039	116.4517	14.86326	135.5406	404.1065	49.64086	111.2279	803.5515	53.03671	57.13385	42.58893	192.4476	71.40012
0	0	71.99786	9.51167	30.94646	262.1573	1421.819	1731.701	1601.785	2211.146	802.8562	2789.944	47.0741	227.9569	0	150.6592
0	0	0	0	0	22.16391	0	0	10.94904	0	0	15.87592	0	0	0	0
0	0	64.36516	8.514008	27.78464	235.7733	1287.416	1559.014	1451.253	1993.208	717.9233	2531.099	42.13567	205.0661	0	135.4916
0	0	0	0	0	0	0	0	10.87001	0	0	15.81899	0	0	0	0
4.311964	0	68.12901	4.973075	34.82446	226.6615	1527.417	3020.055	2863.568	1084.935	429.5598	1517.957	62.53637	45.30173	0	154.8888
0	16.87479	92.32278	0	67.18272	552.4103	3360.933	4203.261	3374.539	6524.354	2415.775	6749.532	133.5673	276.8715	0	354.9119
985.5389	8199.837	144.4407	66.62617	91.4882	279.0067	3817.005	6421.378	4894.765	9673.126	4305.869	8304.593	186.6592	308.8116	5.340348	305.7984
0	0	0	20.52051	0	0	1024.039	2266.396	54.53843	4900.204	2593.289	100.4064	0	0	0	0
0	0	0	0	0	11.19228	0	0	5.478622	0	0	7.94317	0	0	0	0
17.26984	0	122.2164	0	0	0	315.8217	175.2859	29.03631	334.9144	1960.263	218.5154	0	14.99144	262.7091	33.33036
0	0	1831.138	0	0	0	9772.46	0	11345.35	21946.49	10964.52	14214.18	0	1363.47	50.68409	0
12.09802	0	85.75932	0	0	0	224.0672	123.0506	20.34668	238.0466	1425.414	153.7062	0	10.50201	185.8555	23.35214
170.5998	410.5962	135.9252	52.8769	241.3787	0	0	571.554	72.09191	4653.919	1739.535	1660.394	534.1217	179.5967	159.3789	186.8358
45.56784	0	0	0	0	0	0	0	0	0	162.3387	0	0	0	0	0
0	26.86475	13.09619	0	0	0	25.6537	26.99125	0	0	3964.271	1694.798	0	0	2.336138	0
1224.57	11296.9	240.9673	76.00903	162.5334	1035.442	6845.059	9652.635	8344.585	15873.47	7148.419	9646.88	294.5945	590.7065	0	395.8502
87.98731	0	421.3596	0	37.14295	948.0982	4725.858	5551.879	6557.534	955.8553	0	14.93395	0	359.6736	0	471.637
1452.983	4819.169	0	51.18231	62.22957	0	1165.733	678.7253	151.0128	5385.989	5655.127	416.0138	0	0	0	0
0	0	38.48985	0	0	22.56871	203.4891	0	21.39208	74.41496	716.632	42.10925	0	31.52971	10.65258	0
0	0	80.38773	68.23124	0	0	183.5446	0	16.735	55.04326	826.749	107.6493	16.83232	0	19.59147	34.73334
580.6704	573.2954	8915.384	1270.243	96.89724	0	85.54518	0	79.74397	0	2766.299	414.2655	43.17908	390.3596	0	132.3925

0	0	7.583835	40.17981	0	6.519115	193.7785	6.880518	7.240896	52.40859	118.6243	55.6924	24.9802	0	34.73578	19.88955
0	0	43.01295	0	0	0	0	16.65335	0	21.11111	112.5664	0	19.10964	0	16.93058	0
1067.628	7156.897	0	70.62938	81.57425	0	1777.451	4590.827	169.6032	10685.3	5893.039	317.1166	0	12.00959	0	0
0	38.2855	0	56.44563	114.313	0	4370.59	8079.71	350.1789	13558.15	4443.139	814.2414	0	0	0	0
0	0	0	0	0	0	343.1399	0	29.41368	0	0	599.2825	0	0	0	0
0	0	9.46289	0	40.99691	129.5878	1843.853	2338.269	2620.565	2006.035	943.3995	3120.692	42.68344	64.40262	0	295.5595
925.197	6084.233	0	39.52294	103.6548	0	1524.537	3859.741	102.6812	10713.33	5415.916	260.1458	0	14.01199	0	0
925.197	6084.233	0	39.52294	103.6548	0	1524.537	3859.741	102.6812	10713.33	5415.916	260.1458	0	14.01199	0	0
42.58173	0	0	0	0	0	0	0	41.45299	0	0	0	37.75862	183.022	0	1339.712
1156.966	11014.94	0	59.72839	28.77158	0	0	0	0	342.4003	38.88346	376.6042	0	0	0	20.77428
1614.367	15448.34	0	73.7465	23.66955	0	0	0	0	259.0679	29.23604	287.408	0	0	0	15.99727
0	0	9.46289	0	40.99691	55.17053	1843.853	2338.269	2620.565	1629.904	645.8854	2469.334	42.68344	44.61066	0	295.5595
0	0	0	0	0	0	0	0	0	0	15.25834	0	0	0	0	0
730.8274	77.18953	108.8774	823.9443	76.86911	472.0295	0	35.09774	0	0	112.1049	15.13216	15.75107	1061.216	0	27.59658
5.991813	0	10.10789	5.285741	0	0	1746.969	2360.391	262.0093	2415.364	1223.998	492.9086	0	0	0	0
39.81883	0	0	0	0	0	1015.197	3037.196	49.45116	3910.728	1948.952	35.44723	0	0	0	0
135.8935	118.3133	105.4694	81.58599	192.9682	809.8778	7453.798	9948.347	2485.643	21459.45	11240.1	11422.66	195.6317	53.0153	0	485.205
131.2647	139.2805	100.9097	81.36723	189.9809	786.8744	7394.537	9830.881	2393.395	22073.84	11507.15	11240.47	189.4067	50.63479	0	469.8274
400.1427	0	0	0	0	0	0	0	115.5513	0	0	0	110.2662	914.9549	0	172.8142
864.8088	4983.104	0	36.27319	80.37236	0	1328.051	3374.529	75.82491	9645.768	4992.645	238.5549	0	0	0	0
11.11719	0	126.2107	0	96.46686	0	0	0	0	0	0	0	0	78.82728	0	124.4785
375.2402	904.6274	0	0	94.51003	0	1224.934	3802.583	56.97546	14586.5	8834.785	0	0	0	0	0
0	0	0	0	0	0	0	0	54.86243	0	0	0	0	0	0	0
888.8072	6133.432	0	28.83021	74.62284	0	1332.892	3480.047	73.68717	9698.498	5056.212	327.3719	0	11.72215	0	0
273.536	765.7576	0	0	25.43456	0	860.6997	2487.606	48.36811	6793.299	3692.042	0	0	0	0	0
0	0	42.16796	0	0	0	0	16.62758	0	21.09748	111.6684	0	18.62005	0	0	0
116.4485	0	0	0	0	0	0	0	0	528.4389	135.4909	363.5587	0	0	0	0
0	0	0	26.19721	0	0	0	0	0	0	0	0	0	0	0	0
0	0	0	0	0	0	14.32029	0	0	0	0	0	0	0	0	0
30.15467	44.10686	0	0	0	0	26.27666	0	0	0	24.16346	0	0	0	0	0
346.5292	0	735.9232	0	0	0	1452.885	616.9418	3206.401	1908.232	464.8332	1851.004	2656.315	831.9006	504.0196	0
0	121.8748	0	0	0	0	0	0	0	3902.47	58.4448	0	0	0	0	0
0	121.8748	0	0	0	0	0	0	0	3902.47	58.4448	0	0	0	0	0
0	121.8748	0	0	0	0	0	0	0	3902.47	58.4448	0	0	0	0	0
382.3731	0	0	0	31.04481	153.9574	1323.556	2798.465	2230.52	1283.022	579.3743	1735.623	30.48826	1578.344	0	43.75481

0	0	0	1569.072	343.4993	335.1663	0	0	0	34.83341	0	0	0	0	0	87.22231
346.727	0	0	0	0	0	0	0	0	0	0	0	43.89904	1193.252	0	0
0	0	0	0	16.90133	180.4818	2292.213	2937.347	1950.968	5388.214	2513.041	5103.415	43.85533	4.97746	0	108.5121
0	0	0	0	14.91128	157.5503	2031.964	2558.791	1739.569	4653.57	2163.421	4770.489	38.59866	4.995422	0	92.47435
0	0	47.01615	0	0	0	178.0349	0	0	0	0	0	0	0	0	26.69245
0	0	0	0	0	0	0	0	0	26.60389	0	0	0	0	0	0
0	0	11.72679	0	0	0	0	0	0	22.75331	0	0	0	0	0	7.057727
0	0	0	0	0	0	0	0	0	0	0	0	0	0	0	0
0	0	0	0	0	0	0	0	0	0	1379.093	0	0	0	0	0
0	0	0	0	0	0	0	0	0	0	0	0	0	0	0	0
0	0	0	0	0	0	143.6566	48.54407	17.55961	0	221.0085	0	0	0	0	0
0	0	0	735.5463	78.07844	0	2057.307	0	637.6753	0	0	0	0	0	0	1184.432
0	0	0	0	0	0	53.9097	0	0	0	0	0	0	0	0	0
0	0	0	0	0	0	33.84999	0	0	55.53921	162.0993	40.87208	0	0	0	0
0	0	0	17.78025	0	0	0	0	0	39.43398	0	0	0	0	0	0
0	0	0	0	0	19.43061	0	1057.719	7.943451	0	82.29981	2.322878	0	0	0	0
0	0	0	0	0	0	0	0	0	0	0	0	0	0	0	0
0	0	0	0	0	19.52293	0	1046.415	7.945487	0	82.04813	2.322891	0	0	0	0
0	0	0	0	0	0	0	0	31.9643	0	0	0	0	0	0	0
0	0	0	0	0	0	0	0	0	0	0	0	0	0	0	0
0	0	0	18.85101	0	0	0	0	0	112.9986	54.34164	1037.732	11.95439	0	17.13066	17.69591
1501.577	13493.21	0	75.07562	0	0	0	0	0	15.11366	0	41.44784	0	0	0	0
0	0	0	0	0	0	0	0	23.01882	0	0	0	0	0	0	0
0	0	61.1733	30.15061	79.17937	0	52.89793	0	0	757.0373	465.3185	265.1481	0	0	0	0
0	0	0	0	0	0	0	0	0	0	0	0	85.85738	212.615	0	172.0701
0	0	0	0	0	0	0	0	0	0	0	0	0	0	0	0
0	0	0	0	0	0	0	17.39762	0	0	0	0	0	0	0	0
0	0	0	0	0	0	0	0	0	0	0	0	0	0	0	0
0	0	0	0	0	0	0	0	0	0	0	0	14.8091	31.50558	0	27.00451
0	0	0	0	0	0	0	0	0	0	0	0	0	0	0	0
0	0	0	0	0	0	0	12.7344	0	0	0	0	0	0	0	0
0	0	0	0	0	0	0	0	0	0	0	0	0	0	0	0
0	0	0	0	0	0	0	0	0	0	0	0	0	0	0	0
0	0	0	0	0	0	0	0	0	0	0	0	0	0	0	0
0	0	0	0	0	0	0	0	0	0	0	0	0	0	0	0

0	0	0	0	0	0	0	0	0	0	0	0	0	0	0	0
0	12.60202	0	0	0	0	0	0	0	0	0	0	0	0	0	0
0	12.60522	0	0	0	0	0	0	0	0	0	0	0	0	0	0
0	9.457309	0	0	0	0	0	0	0	0	0	0	0	0	0	0
825.4381	6633.815	0	0	0	0	0	0	0	0	0	487.9547	0	0	0	0
250.3167	367.521	355.3238	134.2612	51.09771	0	0	0	0	0	0	320.223	0	0	0	0
213.1849	39.77547	65.00012	0	0	0	0	0	0	0	0	0	0	0	0	77.23585
0	91.84067	0	0	0	0	0	0	0	0	0	0	0	0	0	0
0	81.54373	0	0	0	0	0	0	0	0	0	0	0	0	0	0
0	60.04723	0	0	0	0	0	0	0	0	0	0	0	0	0	0
0	50.68102	0	0	0	0	0	0	0	0	0	0	0	0	0	0
0	39.60484	0	0	0	0	0	0	0	0	0	0	0	0	0	0
0	0	0	141.3172	41.41956	0	407.6149	0	90.61792	0	0	0	0	0	0	191.3147
0	0	0	103.0588	29.65541	0	152.6968	17.64006	57.21204	0	0	0	0	0	0	144.8835
0	0	0	0	50.30865	0	0	0	0	0	0	0	0	0	0	0
0	0	0	0	0	0	1352.061	48.62694	85.55833	0	0	0	0	0	0	0
0	0	0	0	0	0	806.8134	42.24957	42.94189	0	0	0	0	0	0	0
0	0	0	0	0	0	552.9446	11.78337	285.8483	0	0	0	0	0	0	0
0	0	0	0	0	0	380.8978	0	17.07992	0	0	0	0	0	0	0
0	0	0	0	0	0	336.5897	18.31712	19.30913	0	0	0	0	0	0	0
0	0	0	0	0	0	335.6734	0	0	0	0	0	0	0	0	0
0	0	0	0	0	0	312.8141	13.3312	17.75386	0	0	0	0	0	0	0
0	0	0	0	0	0	310.0746	15.59519	11.6495	0	0	0	0	0	0	0
0	0	0	0	0	0	306.2488	15.85481	16.11328	0	0	0	0	0	0	0
0	0	0	0	0	0	301.3047	16.65398	11.17345	0	0	0	0	0	0	0
0	0	0	0	0	0	290.9204	0	10.875	0	0	0	0	0	0	0
0	0	0	0	0	0	253.6233	0	12.06441	0	0	0	0	0	0	0
0	0	0	0	0	0	157.4492	37.87554	19.65139	0	0	0	0	0	0	0
0	0	0	0	0	0	157.4492	37.87554	19.65139	0	0	0	0	0	0	0
0	0	0	0	0	0	16.9595	0	0	0	0	0	0	0	0	0
0	0	0	0	0	0	0	41.99285	0	0	0	0	0	0	0	0
0	0	0	0	0	0	0	34.26786	0	0	0	0	0	0	0	0
0	0	0	0	0	0	0	34.26786	0	0	0	0	0	0	0	0
0	0	0	0	0	0	0	24.97107	0	0	96.95577	16.30834	0	0	0	0
0	0	0	0	0	0	0	23.70688	0	0	0	0	0	0	0	0

0	0	0	0	0	0	0	10.29501	0	0	0	0	0	0	0	0
0	0	0	0	0	0	0	5.522684	0	0	0	0	0	0	0	0
0	0	0	0	0	0	0	0	0	320.3418	962.0752	0	0	0	0	0
0	0	0	0	0	0	0	0	0	0	314.883	0	0	0	0	0
0	0	0	0	0	0	0	0	0	0	261.1874	146.3315	61.3944	42.23474	0	0
0	0	0	0	0	0	0	0	0	0	0	117.5187	0	0	0	0
0	0	0	0	0	0	0	0	0	0	0	73.55523	0	0	0	0
0	0	0	0	0	0	0	0	0	0	0	19.67243	0	0	0	0
0	0	0	0	0	0	0	0	0	0	0	0	12.53214	0	0	0
0	0	0	0	0	0	0	0	0	0	0	0	0	0	0	46.07563
0	0	0	0	0	0	0	0	0	0	0	0	0	0	0	0
0	0	0	0	0	0	0	0	0	0	0	0	0	0	0	0
0	0	0	0	0	0	0	0	0	0	0	0	0	0	0	0
0	0	0	0	0	0	0	0	0	0	0	0	0	0	0	0
0	0	0	0	0	0	0	0	0	0	0	0	0	0	0	0
0	0	0	0	0	0	0	0	0	0	0	0	0	0	0	0
0	0	0	0	0	0	0	0	0	0	0	0	0	0	0	0

AP010_2	AP010_3	AP011_1	AP011_2	AP011_3	AP013_1	AP013_2	AP013_3	AP014_1	AP014_2	AP014_3	AP015_1	AP015_2	AP015_3	AP016_1	AP016_2
6319100	18383590	13622054	20462495	15069308	17386411	19003576	15847472	10804151	18686749	12808587	13457366	18034658	11381907	12881934	10739537
37988016	78841815	64598239	49869801	66906617	60802393	48651538	48747714	53314065	69900718	50566968	52085767	57623161	46003904	94810936	87206329
25357.64	41513.13	60191.2	43040.88	59632.26	47594.94	43271.66	40615.25	46580.2	55491.14	42486.77	37766.85	39312.03	44845.31	60035.79	70509.02
13741.16	45092.1	36764.02	28812.21	41937.36	37490.49	32832.26	32178.53	21513.81	35351.31	22807.32	37702.02	32667.31	31044.84	33826.22	39307.46
16947.26	42559.18	41796.31	31858.62	46725.35	35521.8	32583.63	26137	24911.25	39249.02	26106.95	38246.22	42369.12	34672.05	40046.87	34686.53
14693.19	38028.43	43398.95	38567.82	44851.47	39047.4	35657.69	27693.02	22551.5	36744.82	27752.36	36887.87	41010.57	30580.54	28858.88	28777.31
14693.19	38028.43	43398.95	38567.82	44851.47	39047.4	35657.69	27693.02	22551.5	36744.82	27752.36	36887.87	41010.57	30580.54	28858.88	28777.31
14693.19	38028.43	43398.95	38567.82	44851.47	39047.4	35657.69	27693.02	22551.5	36744.82	27752.36	36887.87	41010.57	30580.54	28858.88	28777.31
16391.98	51275.22	36267.04	32322.01	44716.4	40829.92	30863.1	27106.44	22518.19	38840.91	24005.09	43620.99	45516.79	34341.8	40766.35	44201.36
15626.05	43190.42	40096.93	34691.62	37946.1	38413.58	30968.12	27819.85	21998.53	35264.79	26907.08	36537.53	44756.2	31759.91	31380.4	31858.95
14552.38	37885.88	37246.71	33482.77	41184.47	35761.05	30385.09	24922.39	20954.04	38041.14	24136.24	32525	40258.03	29973.94	29199.92	29108.52
12251.87	37575.63	40509.39	34717.26	42661.07	37283.72	32827.3	26964.12	21286.77	34472.04	25427.93	34173.9	36001.73	30117.69	29090.5	30320.58
11977.25	33559.91	37995.27	30244.23	41664.21	30291.8	27531.1	26046.04	20116.79	26230	24562.06	31148.37	29318.92	29100.66	27233.65	26028.46
15080.86	40646.73	37947.07	32639.87	41096.86	37838.34	33009.11	26856.87	21666.98	36151.85	25061.83	33317.51	35675.91	31916.23	33643.35	33302.74
14405.69	40286.54	37724.49	32525.26	40960.85	38106.29	33012.76	25842.21	20866.66	36785.99	23874.83	33296.51	38740	30490.82	31644.17	31713.7
15383.3	42886.2	29598.17	24579.2	39762.51	34183.77	31219.62	26922.26	23887.07	34212.22	24829.07	36033.91	30046.38	25840.48	33796.55	35212.25
14145.65	39693.92	39169.05	31796.75	42539.97	39125.3	37444.22	28623.19	23933.26	37926.77	27618.21	36005.25	38613.05	30568.53	31584.41	32152.05
10255.37	36521.5	36430.92	27766.27	42988.66	33091.41	27745.03	28280.88	20551.89	33229.77	23918.16	31849.54	35113.78	33166.31	29105.43	32410.65
14373.11	40083.24	37698.92	32751.75	41097.25	37946.13	34395.26	25937.18	20891.93	36289.13	24284.14	33261.8	38387.03	30888.55	32392.15	32353.68
16741.73	41250.62	37550.75	29824.11	39448.92	37956.05	32251.74	28706.44	23995.19	39600.8	27819.89	33846.74	32415.96	31064.05	34523.82	35515.68
13829.59	40266.42	35893.54	32018.01	40387.81	34424.04	31463.11	26306.74	20160.13	33079.37	23453.35	32857.13	31461.78	23881.79	33361.6	34210.75
14097.89	39752.15	37797.89	32918.67	41027.11	38065.72	34507.27	25848.01	20785.07	34970.88	24194.82	33563.33	37373.37	31058.98	32107.55	32078.75
13436.8	39306.72	37020.32	32764.38	41068.37	37833.3	34369.13	25313.08	20424.32	34240.79	23141.04	32952.35	34427.14	30668.47	31613.68	31813.45
13665.03	39359.45	34796.01	31111.91	39299.3	33883.35	30956.08	25774.75	19701.03	32596.38	23123.49	33150.37	30693.08	27856.29	32989.99	33332.53
13665.03	39359.45	34796.01	31111.91	39299.3	33883.35	30956.08	25774.75	19701.03	32596.38	23123.49	33150.37	30693.08	27856.29	32989.99	33332.53
22166.39	50083.36	28243.7	18114.32	33430.37	29992.41	19331.52	28322.49	24531.51	35780.68	20214.45	32693.37	30238.2	31024.14	64914.21	56516.74
12509.04	34712.15	36731.75	30923.69	38399.56	33377.49	27827.27	25318.8	20789.56	34576.43	24719.81	33035.7	36670.73	29066.56	27308.13	27652.82
13975.36	37183.34	35961.75	30121.89	40004.37	34548.5	30348.31	23041.57	20302.03	35044.12	23354.3	31192.66	38420.42	23713.61	29716.67	28579.83
13116.06	35931.27	37322.02	32815.3	39286.53	37476.18	32621.67	25638.91	20217.58	34139.86	24628.18	31940.45	34127.72	28072.4	30103	29645.86
17201.79	38116.31	36458.47	29222.79	38116.6	32238.59	24992.44	23550.56	21042.43	33570.94	25035.9	31845.47	36716.48	30679.34	37170.69	35721.53
17201.79	38122.5	36458.47	29222.79	38116.6	32238.59	24992.44	23550.56	21042.43	33570.94	25035.9	31845.47	36716.48	30679.34	37173.39	35721.72
13604.81	34410.9	37346.97	32224.2	43336.41	36809.33	32416.67	27132.93	21819.75	35060.72	27548.94	32242.58	33735.71	30055.55	29410.81	29785.32
12273.41	34094.18	36027.76	29925.14	39164.24	32054.2	27217.75	24366.63	20425.67	32623.76	23667.56	31697.87	33725.86	27870.71	27128.33	27320.04

11763.37	33451.88	34822.86	29334.17	35661.03	31986.02	26354.23	24366.63	19986.63	32479.64	23667.56	30837.73	33725.86	27630.55	25835.94	26501.35
15059.98	39127.15	36149.05	30647.26	42266.5	36945.4	32529	26508.04	22187.4	34187.33	25199.64	32919.74	30030.24	31587.68	32334.52	31784.68
12351.48	34094.92	35989.33	26260.09	31052.5	29595.98	17165.77	18963.5	14748.52	30730.94	21340.21	24903.48	49430.07	15264.18	30439.19	15694.57
14558.05	35738.98	33131.47	29762.73	36644.22	32150.25	28856.22	23259.91	19097.73	30486.67	22684.01	30730.02	33893.9	28957.3	25521.63	25866.79
14285.18	39419.36	32448.05	28933.85	40287.02	32203.04	30573.4	23843.93	18990.14	29833.91	21881.8	31807.4	31078.96	27635.4	26113.09	29112.16
13739.59	39892.65	24154.43	19677.64	34774.27	29098.99	27181.87	24020.58	22449.15	29852.98	21749.32	32069.87	23920.96	21420.06	32159.81	33953.76
13671.71	33008.21	33050.2	28034.6	35672.88	31446.75	28079.51	20880.25	19216.22	30883.97	21927.32	26555.65	34939.74	19272.67	29477.88	28224.82
10505.75	36836.69	32985.19	25549.24	38145.32	32389.55	29609.74	26979.01	20287.83	25551.88	22369.53	27627.62	20137.32	23301.59	30158.59	35518.98
12772.73	33129.08	31939.5	27451.91	38505.09	31231.92	28762.09	24544.18	20205.47	28266.68	23354.13	30755.88	24702.29	31519.02	27497.49	29259.82
11163.54	35945.89	35193.28	34326.07	39974.14	31959.66	30232.73	24514.03	20119.25	30441.5	22983	28179.78	24777.25	20415	26603.16	28036.12
12613.76	38931.66	33417.55	30041.63	31695.09	31903.02	26967.81	24932.01	20173.58	33867.69	22746.49	33028.67	34278.89	25275.47	31459.75	31242.58
11211.15	34574.94	31301.5	24738.44	33645.37	32509.48	25406.12	23240.63	17532.09	26807.96	22433.2	29222.66	30052.29	30380.03	28001.08	31680.81
13840.44	34978.65	34645.9	29642.99	43159.38	34614.82	30740.14	23558.28	21171.27	32167.68	24722.55	32714.96	30274.98	30379.53	30884.38	32397.62
12055.23	32742.93	33388.44	27922.75	37784.19	31435.47	28189.88	24368.16	19808.2	29081.39	23331.24	30077.73	26245.98	28976.42	26243.23	27166.67
10779.35	35485.97	34730.03	34192.14	39504.99	31660.97	29908.23	24301.76	19862.72	29300.47	22585.53	27262.17	23387.84	19035.93	26255.8	27838.44
14547.8	32536.82	35034.16	26255.08	38673.04	31526.54	29759.42	23434.98	21349.9	31302.71	21662.59	26035.18	31293.81	24189.1	29042.85	20978.37
14343.16	35952.57	27216.73	19557.15	28632.35	23379.21	13833	20392.03	10138.79	21700.97	15386.77	28929.4	33376.84	22275.15	22239.35	25031.91
9959.218	34021.58	31098.17	24186.01	36059.33	30548.88	28222.71	25218.52	20021.76	23753.2	22014.99	25231.58	18012.4	21832.41	29109.54	33637.35
11507.87	35542.25	26791.13	20539.57	35003.45	27403.48	22800.45	21159.71	17816.93	24594.12	18816.6	28191.66	21860.5	24081.4	32372.62	32119.19
9338.898	32236.27	23239.79	17770.71	29570.54	25106.79	21150.62	17884.51	17184.99	27893	17617.04	25034.73	25292.36	14365.25	24569.83	28874.06
12920.52	38254.08	21823.76	17515.2	32049.31	26832.04	25857.67	22819.72	22136.22	28065.86	20166.8	29723.39	21281.89	19374.05	32698.55	34724.29
13470.37	31181.77	33920.48	25476.6	37518.44	30969.31	29448.48	22931.45	20626.5	29650.1	20774.36	24379.93	29260.2	22583.52	27311.84	19548.02
13295.84	30740.41	29715.1	23888.96	32187.94	27977.12	24175.12	20583.9	16337.42	26591.09	19955.87	26290.05	28997.93	24337.07	23989.76	20958.53
8284.394	28719.73	23775.91	21060.07	33296.62	25130.78	23432.8	20646.13	17288.62	19683.99	20715.76	24063.03	14386.1	27757.92	22605.05	26544.05
12427.55	33384.89	25454.05	21536.76	32710.16	28285.38	26799.15	20376.56	17660.43	28185.11	19578.61	26996.55	23798.84	15880.43	29707.19	28260.64
9348.309	35949.05	20762.13	16261.18	29789.58	26012.81	19910.16	20520.37	15021.83	22212.8	18382.2	24610.77	15400.23	20129.72	31292.88	36249.36
11989.98	36380.31	19336.14	15412.24	29459.56	24250.86	23539.13	21117.2	21104.5	25651.42	18532.46	27599.82	18673.19	17314.08	31302.48	33563.93
11098.19	32717.86	26943.84	21991.38	32215.49	28473.85	25036.28	21244.63	17140.95	22929.84	19322.73	23314.61	21931.49	20020.88	27646.53	29686
12801.99	27961.14	31658.07	23456.99	34726.29	28891.88	27794.93	21541.74	19151.45	27275.29	19252.48	21602.28	26489.32	20061.97	24209.59	16639.33
12019.65	27353.01	25727.07	17580.97	30308.8	26859.85	22707.74	20650.55	16867.03	22172.41	20139.31	24986.67	23239.53	17388.38	26908.11	26019.38
18360.48	30688.5	24115.62	15938.37	27598.98	28820.1	24626.22	18796.45	18775.76	30581.74	18864.52	29444.83	36845.58	19528.99	33795.84	31775.22
7975.027	28456.44	21167.35	15305.18	28731.92	19099.16	16819.41	17906.44	13739.4	14131.31	17269.78	23315.81	12476.38	26913.92	25998.86	30893.88
13257.12	30406.78	24472.45	18907.64	17967.72	21627.13	14327.08	12024.46	14244.51	26422.31	15106.28	26401.27	37036.15	7926.13	21865.64	13181.7
11418.79	35491.2	14873.03	11794.58	25244.52	20068.01	19457.8	18494.65	19936.49	21936.52	15602.14	24726.73	14555.97	14079.39	32566.35	35978.41
11418.79	35491.2	14873.03	11794.58	25244.52	20068.01	19457.8	18494.65	19936.49	21936.52	15602.14	24726.73	14555.97	14079.39	32566.35	35978.41

11437.18	27067.48	21798.15	13698.78	22521.49	23392.64	21162.09	19189.7	18321.08	24956.3	17742.04	11089.2	12891.93	19263.42	25242.34	23465.63
7134.53	27291.96	20651.84	13461.93	23776.75	22874.27	16939.62	21497.76	14879.66	17894.69	16751.89	15845.45	9262.738	9265.441	24051.27	27975.43
4789.403	30989.51	18223.04	11592.02	18656.74	20492.25	15451.59	16908.91	12383.89	13106.01	11157.73	16223.55	12278.87	17552.74	28890.04	30364.89
12123.58	26304.55	20683.23	12667.22	21248.58	22455.12	20162.96	18570.01	18402.69	24494.04	17313.32	10161.81	11994.63	18476.16	25767.95	23191.82
9382.342	23870.48	24001.94	18849.91	20267.43	23493.73	18819.45	16899.34	12622.4	22197.94	14922.94	14623.38	22283.86	13022.3	27609.88	21521.49
12808.59	25803.47	27093.34	18978.35	19757.16	21109.94	13220.83	13444.22	13503.55	22187.19	14582.94	17607.48	24192.52	12554.47	27056.61	14423.33
6267.458	23162	23429.65	16065.89	23597.75	18800.4	17271.07	18366.66	10831.62	21721.14	17242.56	14352.96	17854.49	16013.63	18995.98	15885.51
11105.31	22604.08	21197.32	15245.27	15645.71	17124.58	10647.55	9262.168	11131.52	15009.62	7731.959	9363.545	11908.07	15573.14	30016	27421.97
7238.149	24752.81	21961.61	21348.52	25837.99	25226.75	21064.13	21034.19	13507.75	20745.23	17424.02	18443.92	15676.57	13232.07	15820.87	21311.68
13402.33	25767.73	18535.87	13884.48	11603.85	19466.1	14630.71	12470.44	12593.86	32034.02	14953.83	22649.18	39916.01	9772.428	22680.74	16210.89
9933.505	19979.98	24373.66	19478.76	21676.79	22517.96	19329.76	11979.06	11520.16	25520.96	13864.01	16885.55	34998.54	5912.59	20580.14	12445.46
11872.67	22897.06	18625.19	11703.01	10913.15	17062.82	15315.08	17995.87	14753.33	19169.28	13336.24	5583.345	8056.257	8133.804	29015.07	27314.74
508.9788	24726.96	18682.93	17809.25	22875.15	21008.69	17137.59	16942.18	11133.21	15555.11	10979.73	17846.71	10937.81	12762.53	16739.79	20772.15
2781.328	28915.37	7939.332	5812.428	20830.3	16241.54	12861.56	12118.99	10045.32	18501.71	7492.496	19953.05	10902.02	11008.63	21040.37	26397.54
11202.63	22917.26	18429.08	11674.01	10910.46	16946.62	15247.09	17923.67	14603.12	18631.01	13057.83	5535.821	7778.539	7989.606	27740.69	26698.43
4572.96	23929.38	16595.76	20633.8	20434.45	19806.55	17648.05	11409.48	9360.854	16676.92	8139.309	16155.79	17527.31	6994.645	24912.95	20096.58
6912.652	20549.06	20581.31	13051.02	20054.27	15008.29	14198.35	16475.02	8685.09	20846.23	15700.09	11756.24	22458.99	12588.81	16985.69	12311.78
9899.671	19638.4	23600.57	18233.66	18247.8	22287.2	19033.83	11854.88	11086.38	23014.9	13489.33	15150.56	28973.79	5864.018	20428.44	12331.31
8983.895	20634.69	22347.07	17151.72	19610.76	19346.81	14438.86	13168.02	10808.2	18657.63	12646.92	16892.96	18057.62	7513.602	19340.79	14895.52
9708.945	19365.02	22656.28	17804.24	22431.87	21104.97	21015.38	10960.13	10559.81	23939.05	11819.24	16325.16	34977.62	5843.696	20800.86	12080.51
7182.554	17931.93	20805.26	9863.077	21417.27	18254.9	22174.62	20591	29794.89	24295.99	16638.59	11941.08	25255.44	4361.145	0	14838.75
8073.884	20668.88	20546.56	14639.31	14232.78	13910.73	7854.747	14748.5	10468.54	19556.08	15176.66	14133.17	18367.23	20270.22	16368.62	12233.22
8227.142	17757.82	16101.08	13499.94	19899.49	20121.73	19923.42	11959.75	12371.01	18683.66	14248.51	18619.07	15898.94	5934.645	15293.33	10321.52
5934.543	20707.04	18850.79	12437.6	11558.78	12526.06	5657.52	15061.26	7409.098	18201.56	13100.16	11410.99	19721.64	15546.47	12931.36	10335.21
8514.14	19656.7	17456.47	12254.98	10777.39	15875.43	12710.72	10785.54	13263.9	17375.69	10612.38	6625.159	9201.322	6923.503	21607.39	14580.48
9686.332	19224.95	20781.49	13397.02	24131.86	14664.76	17660.59	12875.18	15287.87	17972.36	15589.82	18504.93	13727.96	18753.96	18692.8	15843.85
3536.852	21555.08	4275.344	4677.5	17192.98	16155.33	13897.73	10006.98	5831.96	9619.308	2204.602	14845.23	9536.163	5070.128	22980.95	16375.9
19039.01	23098.26	17384.34	9613.575	19521.45	21965.45	19862.59	13826.91	16790.8	26988.79	14765.63	24322.12	34728.06	12841.93	35107	30011.96
9634.128	16637.76	18813.96	14107.31	15476.96	15017.92	10828.23	10649.45	9194.221	20398.3	9729.8	12755.38	28424.95	5310.956	18996.92	13456.37
11379.43	20489.3	14845.67	10372.06	16472.13	18070.4	14516.91	11408.14	12428.93	17793.16	10672.19	15770.73	14234.27	8484.158	25678.86	21693.28
7722.293	16516.1	14638.24	8654.528	9199.822	10869.12	7391.145	14692.5	11958.85	11001.65	9843.786	3598.642	2460.625	15605.65	23562.65	23269.85
5840.356	16162.15	12779.54	6768.354	11850.59	14215.64	10478.42	13876.34	9440.22	14862.72	12516.99	12379.65	15603.66	8828.275	20481.62	23159.27
8203.647	17117.11	21462.86	12289.86	26738.81	14049.35	18333.09	12558.16	15010.6	16717.7	18230.06	17130.64	11638.23	19616.02	16249.33	13895.28
14427.24	21191.31	15757.57	10162.37	18103.09	18657.26	17167.56	12973.31	15955.06	20830.1	13380.84	18562.13	20163.45	10176.58	31826.57	29063.05
9535.337	15820.43	14860.12	9708.208	9037.879	11089.66	6378.396	6333.415	7696.879	9379.845	4750.973	5914.355	8143.074	9841.664	25993.24	19087.32

9535.337	15819.72	14860.12	9708.208	9037.879	11089.66	6378.396	6333.415	7696.879	9379.845	4750.973	5914.355	8143.074	9841.664	25992.36	19087.28
1731.918	21974.74	6416.837	4845.967	14897.74	14201.92	11417.66	10727.33	7299.014	10421.09	1602.891	8067.985	7742.94	9220.325	19704.22	20431.01
7455.376	18366.79	6962.548	7485.379	10742.98	14946.39	11908.79	8346.445	8291.589	12285.32	7945.89	15104.5	20531.71	11174.32	23098.12	17773.04
19489.8	20234.69	15555.76	8465.448	16345.93	19150.08	18262.3	11584.33	16899.21	23866.64	12056.27	19175.53	29010.27	10268.92	36585.07	30970.44
381.9884	18685.28	14501.72	18927.72	17681.56	17457.61	15578.19	7807.006	8058.035	14532.76	7256.315	14117.42	15681.2	5452.559	15975.96	11616.07
8195.99	13201.07	13207.53	7790.414	13106.46	10657.04	5710.114	15550.29	4888.721	5376.073	7938.344	4544.909	3022.342	7243.107	16183.7	14455.14
8195.99	13201.07	13207.53	7790.414	13106.46	10657.04	5710.114	15550.29	4888.721	5376.073	7938.344	4544.909	3022.342	7243.107	16183.7	14455.14
3949.596	20853.6	8753.738	6625.669	10539.86	10250.64	7292.367	7638.717	9512.711	9332.505	5383.972	7631.527	13077.49	6425.831	17677.68	16116.77
1098.115	19557.55	13616.3	8661.306	17967.6	10907.21	8547.693	13816.02	9045.96	5435.602	8679.276	9316.146	5445.036	7088.57	19543.58	13695.79
11424.06	17946.58	16125.21	12858.99	10516.33	14284.58	7686.722	8235.088	8641.768	20751.21	9021.996	15078.24	25307.18	5053.785	19675.96	13212.27
11067.87	19736.27	12457.85	6321.919	16228.65	14918.14	16364.17	12330.08	16390.16	20369.71	12568.88	9116.75	12434.89	11205.29	15731.63	6349.4
16304.74	28274.07	18724.2	17264.65	16187.23	21011.41	13536.95	16598.2	12716.09	22512.64	9678.046	19691.4	32738.54	13315.3	39471.45	29499.99
11818.51	17651.19	16617.46	16585.11	19573.65	15061.17	21000.24	8736.032	9812.369	18625.6	10652.36	14230.24	20435.48	7547.601	20552.05	17841.03
5468.931	18092.16	4981.741	3410.677	10832.4	10217.61	8345.033	8168.286	6207.644	11568.19	4637.877	5779.977	5610.567	7314.539	17768.69	18255.47
7265.399	14591.68	12017.34	7441.86	9346.169	12237.05	8281.809	8620.597	8516.669	13671.89	9826.734	11814.68	14424.54	9185.565	18681.59	13868.21
9336.523	13361.29	16679.45	10061.87	10373.94	10355.51	6078.196	8169.882	7633.549	9091.087	8530.375	7855.698	7971.804	5890.043	11080.7	9297.729
2000.361	8850.141	19407.34	14291.65	5132.422	12555.48	7234.224	10408.03	4315.012	6437.648	10951.18	1698.555	5088.453	1843.175	4294.116	3188.224
4035.92	10526.58	11336.35	7631.327	7234.807	9550.271	6361.532	12058.82	9207.416	21617.91	11157.02	8298.431	14690.74	9281.823	23349.57	21042.54
8750.243	26608.24	14176.1	10763.87	16914.21	18776.44	14515.93	13924.6	8668.737	10876.53	2444.832	6931.289	4288.91	11475.2	26108.14	26567.94
9437.313	27927.75	14892.08	11181.72	16827.99	21071.24	15499.07	14751.02	8732.119	11177.32	2318.457	6655.533	4226.87	10952.03	28086.49	27722.18
8483.588	17189.76	10055.03	8286.775	10225.94	14579.28	11139.61	10580.85	12008.15	14249.7	8649.131	5647.76	6371.638	7201.885	24417.55	18284.97
8613.445	26431.87	13540.43	10008.48	15596.4	18640.79	13587.6	13632.26	7969.735	10033.99	2125.357	6148.253	3805.752	10379.42	26265.39	26344.73
5191.089	12557.03	8343.888	5902.632	8372.3	9211.002	6306.953	6118.04	8601.435	8058.661	4698.574	6466.296	8547.629	5663.182	16622.34	9635.901
11272.46	15050.63	10350.43	11245.37	3887.522	9189.854	5277.329	5557.974	8697.776	18230.81	6808.843	14768.67	23048.26	5099.111	19466	13875.03
9330.58	27146.26	13819.2	10381.71	15645.28	20001.25	14487.55	13802.39	8004.622	10334.71	2053.567	6030.553	3807.895	10225.69	27700.19	26805.18
9929.211	15967.58	11228.03	10026.99	14293.82	18707.69	15894.97	11821.91	14406.82	20590.97	13293.7	7679.071	10941.77	10051.35	22151.81	19437.53
5694.039	12809.8	3854.217	4343.1	6110.802	9789.59	8114.598	5012.312	5154.736	7615.654	5124.236	9704.668	14800.3	6578.615	17011.93	11157.91
4386.333	13892.46	10687.85	6591.999	5093.125	11777.93	7709.178	8462.353	6598.63	13285.15	9972.75	6297.198	15167.61	6275.047	7596.386	1332.674
11669.1	12660.7	22660.86	17807.71	26880.67	23296.72	23738.63	13598.43	15306.44	20513.7	16067.5	7345.946	7099.134	19925.83	21652.05	15120.54
3161.235	9526.304	3551.585	4152.346	3820.54	9103.872	5093.301	5442.026	3617.945	5267.543	5065.325	1579.295	1601.862	2969.219	11801.69	5814.158
15654.35	14537.27	11879.53	9231.687	14999.28	11372.08	4655.634	5771.949	7368.298	15541.43	7477.414	11145.2	19233.3	5246.294	20098.04	15146.03
7903.161	8010.127	11865.59	6439.284	8394.882	11273.36	7010.758	4858.98	4514.616	11493.66	5918.371	11451.99	10641	1283.749	6346.173	2144.184
7903.161	8010.127	11865.59	6439.284	8406.662	11273.36	9270.034	4858.98	4514.616	11493.66	5918.371	11451.99	10641	3442.254	6398.258	4830.901
8723.373	26853.73	12495.78	9424.712	14329.11	18734.04	13138.02	12793.21	7121.897	9372.978	1777.053	5339.275	3317.911	9103.963	27510.45	27077.63
9191.969	26169.85	12608.1	9477.595	14304.78	18748.73	13327.79	12712.35	7244.736	9415.407	1782.089	5359.514	3363.383	9394.917	27201.34	25672.89

3821.746	7604.272	5345.227	4112.017	10159.54	7423.489	8979.597	3546.639	3465.469	4182.995	2440.802	5351.745	7947.061	3644.756	11152.77	8079.724
8870.563	26037.89	12563.12	9420.34	14269.34	18800.18	13366.11	12785.5	7209.933	9312.066	1782.167	5323.563	3331.486	9298.03	26900.81	25897.81
1182.171	12874.16	3207.427	2328.48	7419.385	7666.458	6192.598	6070.114	3897.803	4753.064	592.7362	3374.346	3238.465	5216.523	14175.54	13029.43
6577.37	10620.63	3775.945	4238.16	6378.991	8514.781	6033.809	5057.688	6738.736	7841.133	4862.699	8945.06	9303.877	7175.53	15903.11	9450.068
11354.94	10068.61	11802.64	7347.267	6628.81	8083.48	5597.284	5089.918	8556.1	9064.949	5540.544	6367.749	6721.354	5366.067	14248.4	12027.64
116.8174	12243.05	15108.96	8178.06	1919.417	8811.719	3208.944	11113.59	3474.563	2962.599	6394.82	630.2118	948.7484	0	797.4068	38.35255
5376.131	8030.083	6502.277	4444.359	4775.45	5270.695	2884.104	3233.909	4042.868	13247.05	5281.949	8251.956	19399.8	1990.322	6849.315	3637.935
5327.747	9743.464	9837.56	6473.2	3727.858	10373	6195.157	7715.603	5340.486	11042.28	8483.186	4595.725	8893.525	3582.842	4137.16	1014.788
4693.645	11011	5628.095	3792.898	7407.375	7099.755	4918.953	7673.875	4309.443	6256.87	1500.727	3615.555	1548.025	6223.227	15855.16	12589.26
3523.616	11164.45	11145.17	9424.193	15638.55	15898.01	15858.19	7218.069	6637.605	15428.81	8665.424	9790.81	11403.99	4409.191	11502.02	6937.237
4693.584	10398.63	8279.354	14187.61	9558.872	10636.1	6496.736	5047.76	6513.459	11979.86	9208.728	8227.668	17222.24	5023.807	16298.86	7335.724
8890.566	13084.18	8094.378	6525.376	8791.871	13045.08	10855.92	9027.629	11974.65	15270.11	10283.74	4650.068	4877.984	6188.778	17044.52	13041.52
12270.07	23247.39	5021.38	2062.458	4996.847	5493.007	3767.94	6322.154	9142.715	18905.46	6294.017	9679.055	12398.64	9993.263	34523.82	28922.43
4511.408	12565.22	6886.979	3692.55	8159.096	4232.119	3495.884	4688.109	6865.111	14913.35	10049.55	5758.867	12568.42	6689.978	10314.25	8807.204
9231.007	26752.78	3374.711	5575.588	0	17627.57	11179.91	11302.48	8910.383	11119.07	2391.575	3179.775	4159.279	11259.18	27634.19	27260.34
3918.036	5195.08	0	205.6455	25.40306	6684.739	2108.176	2881.268	4661.175	5065.845	512.5632	495.5899	271.9712	5075.881	13105.22	9566.487
654.1818	2639.649	0	0	28.21729	6948.21	2294.458	2975.578	674.9477	363.9204	66.26682	456.9177	256.5531	4546.826	4465.57	2948.882
9322.588	11588.96	6961.118	5960.449	8918.45	12465.63	11230.29	7856.045	11353.37	15810.43	9712.598	4822.743	7169.641	6055.739	15543.94	13735.24
3556.436	4574.584	0	204.1274	25.38957	6324.93	2050.72	2774.478	4211.683	4987.948	508.9272	491.8406	271.8437	3559.693	5206.945	2669.772
3745.445	2267.203	3241.044	4526.7	5249.611	5547.213	2282.909	2310.255	2671.316	7415.927	3724.368	5016.159	8982.14	4230.587	7047.182	2275.729
4010.242	9956.965	5123.456	2726.903	6634.808	3130.729	2563.672	3418.037	5362.73	13134.37	8376.048	4616.742	16002.17	6973.216	8399.46	7308.03
7910.242	13339.96	6514.04	4404.096	8750.627	8183.036	5700.375	9967.388	7193.302	9527.183	3863.729	7719.785	16537.31	5077.546	19530.66	16527.55
6532.988	11617.5	7833.663	7105.202	4890.751	10143.31	6663.583	4224.023	5808.091	10455.43	4780.972	4192.706	10404.89	6365.783	17120.36	21750.14
9445.831	19534.89	3540.44	1796.708	3539.493	6163.681	3228.663	6291.339	9439.002	15295.57	6478.703	3249.397	13197.41	11850.11	22974.64	19658.26
4552.591	11691.83	8617.671	4623.071	14250.96	10087.45	13418.4	5371.528	15414.31	18893.92	10596.41	12648.15	3611.032	5393.505	19200.72	12954.91
5114.974	7204.095	1312.898	465.303	2938.507	5914.734	1510.441	2096.275	2900.284	4183.522	372.8969	1317.57	1892.487	4028.587	13703.3	10116.85
306.5181	2528.874	6660.306	6015.452	11468.49	12851.83	13974.52	1128.505	2101.646	6286.025	4573.205	4316.967	4948.097	909.7639	173.285	553.4749
4573.267	6509.971	3570.461	2148.916	4659.594	4200.496	2803.013	4639.622	2569.018	3966.149	798.1272	1961.204	741.3051	3837.76	11086.27	8502.906
1271.204	6039.005	3875.258	2571.864	7830.355	5783.732	7721.252	4993.475	2218.433	2186.166	1932.161	2288.299	1491.339	2307.739	5435.354	3839.689
3548.489	6778.726	13068.98	13060.79	6120.268	17166.06	10151.16	7845.34	4025.096	8613.274	6737.401	1570.872	4165.351	5516.374	12831.12	9397.022
7436.68	12208.64	2580.199	1077.782	4514.452	6555.004	3410.038	3127.246	4145.874	6399.775	1474.102	4705.421	6266.847	6341.737	20789.88	17875.15
3042.204	9366.488	2800.972	1359.154	2694.346	4313.696	2340.322	4778.446	4066.018	5576.709	3237.271	2886.497	7391.987	7008.636	6760.418	3847.153
318.4669	4172.069	4720.26	1996.543	5704.227	1499.487	1002.106	869.56	142.4056	58.12326	566.2127	337.0844	1165.691	81.61755	1039.146	176.4052
1115.753	3746.939	0	375.5898	50.58233	5309.541	1726.672	3595.817	1738.88	4198.117	936.6356	888.4722	511.4316	6179.763	6893.052	4723.075
6795.978	10873.55	3014.029	770.0305	5499.23	9423.788	6698.786	5566.058	4923.552	7618.272	3379.46	6879.803	9250.587	4053.018	18588.5	12730.36

4434.355	6817.761	2526.228	1160.166	3462.225	4992.527	2434.715	2425.555	2913.389	5382.338	2095.849	3020.718	5464.727	2785.085	14622.33	9769.323
3327.086	9900.851	1822.819	958.6202	3547.453	4029.102	2855.636	2911.338	5602.675	10486.02	4947.467	1388.293	8509.324	6497.506	9593.774	7258.803
4534.871	7610.538	4518.48	3775.165	3781.535	7882.577	3298.874	3135.038	4363.602	9726.585	7412.234	3540.609	10275.73	1668.184	7613.255	3168.726
510.7456	5169.635	5713.924	3221.49	3915.028	1843.244	660.502	777.5948	2357.728	9535.049	3243.542	170.2762	7184.911	30.12816	2747.476	259.5749
5490.627	9432.509	4378.874	1795.833	7347.352	5073.914	3589.306	3052.708	5377.437	13560.44	8915.329	5241.024	15507.85	7978.504	9381.943	8610.245
6753.743	12582.97	2860.721	5023.053	3470.939	6326.828	2026.198	3939.118	6087.51	8291.688	5107.957	938.7376	3342.963	6867.149	19200.03	15953.56
20.23358	1133.714	3237.604	2403.09	3000.133	2138.597	2778.541	2841.738	1777.568	4848.552	1688.84	1708.656	2342.774	0	0	41.52602
7007.903	9871.262	3171.813	3142.962	4663.834	5337.031	1824.831	4508.764	5170.111	5878.838	3687.536	1871.786	2057.684	6333.903	20689.61	16927.72
2893.023	5452.083	3656.577	2062.743	3088.551	2366.337	1145.924	1323.551	2217.553	4583.105	2621.321	540.1301	6966.82	94.84096	4326.556	101.5608
1816.746	1253.538	1328.361	234.7104	436.1442	2483.645	823.2308	2626.405	1781.3	2019.069	197.9633	122.616	354.5696	2521.332	5615.421	3217.812
5772.637	8144.412	3769.129	1507.021	6642.919	5276.716	2259.889	2972.348	4818.565	13318.77	8534.22	4608.107	15797.75	4998.313	8625.779	7517.454
9304.497	12860.57	1968.15	981.3477	5001.349	9684.2	5613.855	7330.702	7634.173	10891.1	4132.2	5917.853	6844.848	6833.542	20885.12	16420.9
8772.77	16160.28	3273.321	1056.156	2154.243	8935.317	4766.99	4642.414	7080.172	11903.86	4514.054	15597.16	12260.39	5417.2	17094.65	15666.75
4644.123	5887.662	2144.814	679.6945	4794.455	4043.349	4356.042	5115.266	4272.478	7033.593	3183.516	3765.145	5840.121	2446.689	7898.856	725.6
11639.59	21002.9	1918.151	994.7081	1917.278	3791.616	1816.427	3842.826	8111.833	17022.45	5439.778	1632.335	10982.19	8767.934	33647.48	27284.41
5581.765	10940.03	1901.703	934.179	1900.953	3307.686	1721.203	3512.701	5402.664	8132.798	3322.681	2709.051	7407.181	6997.678	14345.87	11300.18
202.5829	1030.294	5663.289	4341.369	12569.57	11098.33	16434.58	535.3524	1072.259	2932.049	2784.105	2695.833	2959.262	427.1768	107.9476	333.9699
1973.318	10752.58	2038.777	1320.82	1772.634	4178.369	1592.93	4935.95	2909.972	3602.482	3602.686	2218.09	5163.941	7373.027	8104.986	6080.42
5978.956	18744.75	5372.15	4436.632	1621.137	11055.83	2615.919	6184.82	6426.415	10938.68	635.6	8225.711	2941.673	15811.93	18578.68	16343.93
15.18055	874.8028	2548.479	1960.355	2381.881	1656.405	2194.839	2283.154	1385.198	3808.322	1305.765	1321.365	1850.471	0	0	31.16876
5268.049	7515.694	4038.244	10241.22	3942.627	8612.886	4405.82	4026.06	5065.067	6237.04	1270.235	2065.678	1899.722	5355.897	8716.766	7423.486
4849.132	4567.599	129.9425	252.261	77.42218	5103.121	1463.825	1838.606	2939.469	3879.957	429.541	458.5979	438.057	4604.414	15590.41	14094.37
4235.691	4547.173	229.8133	251.4531	1883.471	4849.331	1424.581	1833.578	2861.84	3722.94	1094.866	1340.108	1779.779	4773.96	13853.41	13240.95
15.55149	3901.037	50.87612	21.8958	49.28705	888.704	1532.821	1593.902	1329.565	2544.328	1916.658	2750.749	3947.585	946.6417	737.0136	244.471
6312.058	12606.16	998.2524	1117.572	864.6382	7768.337	3291.999	5186.656	5594.212	9091.844	2715.734	2204.125	4275.314	4315.986	17988.48	12150.38
3221.078	4718.886	3577.477	9819.697	2462.338	6958.708	4147.554	736.27	3366.891	6638.594	5003.492	1476.18	1979.569	3386.629	5744.133	4701.361
359.087	1711.11	3714.151	2170.875	2816.334	3860.73	2656.412	782.6018	685.7636	1070.352	1145.696	967.7375	1328.584	15.0243	2324.962	514.1366
667.9672	1567.256	334.9054	307.7794	838.578	5360.144	2984.653	2065.207	2109.508	5076.313	3168.759	1410.851	1836.953	1894.426	2301.235	1697.978
6484.643	9496.271	1642.339	395.9391	2125.487	5193.261	2093.768	2955.285	5896.03	7112.143	1811.973	1102.598	2395.759	5242.819	23652.44	17245.32
671.8988	987.3403	326.2409	43.92319	1658.511	1498.611	685.6308	2376.586	654.6031	657.2776	320.4961	0	136.9326	530.96	2202.719	1584.014
2977.166	2418.68	2290.317	1638.315	3870.909	3660.817	2922.934	820.4367	1814.599	2576.928	1956.468	4607.225	7042.953	1662.498	6148.506	5534.02
2297.173	1845.125	1169.498	352.33	566.5975	2949.904	938.8506	1041.128	1951.714	2312.813	221.6093	191.6517	492.0793	1039.53	7612.725	4791.645
4407.537	11092.57	1049.901	1158.977	1944.597	8239.856	5267.013	7357.756	4788.696	6816.38	1267.098	962.9205	1060.564	5875.358	16068.37	15053.49
5949.98	11391.85	1756.771	3443.566	2697.633	5710.925	1873.618	2754.721	3652.368	5268.239	526.3115	1083.302	2482.207	5019.322	22254.06	18085.26
3720.475	2953.454	216.7198	451.1715	949.2841	2330.561	841.5985	2639.302	1832.098	3016.911	472.5484	601.2029	1084.86	1764.558	8535.009	5743.493

4375.836	5157.853	1024.316	1003.448	1935.719	6873.465	4500.76	4484.037	4382.419	6024.699	1259.214	956.7538	1054.064	5834.094	12973.28	11529.2
4187.575	10160.51	924.2918	1023.828	1721.218	7567.82	4811.196	6733.86	4438.16	6350.659	1122.693	848.0757	940.6063	5456.753	15177.58	14014.43
4187.575	10160.51	924.2918	1023.828	1721.218	7567.82	4811.196	6733.86	4438.16	6350.659	1122.693	848.0757	940.6063	5456.753	15177.58	14014.43
4187.575	10160.51	924.2918	1023.828	1721.218	7567.82	4811.196	6733.86	4438.16	6350.659	1122.693	848.0757	940.6063	5456.753	15177.58	14014.43
734.9453	411.3319	2874.742	63.30926	1220.859	3805.875	2956.11	1777.756	3526.648	6156.322	4442.388	85.096	32.62643	133.2218	3738.068	3096.583
1540.847	803.4947	352.4767	155.2372	2191.32	6154.739	5227.478	2420.178	358.8177	387.3136	781.7573	1896.406	2142.739	193.6112	48.21899	15.85173
5134.86	8730.448	1499.805	3027.971	2292.364	4862.565	1601.976	2321.458	3155.036	4592.312	442.4591	899.3229	2160.41	4464.348	13969.9	9458.948
2998.102	2544.765	1885.324	2683.782	1876.832	3162.624	1942.769	238.3737	1713.249	3056.925	729.1591	1070.747	1386.692	143.8165	2186.088	197.9074
3837.401	5157.853	854.2001	883.7423	1683.65	5515.121	4401.176	4484.037	3228.206	4105.862	1090.602	902.2697	1007.527	3599.876	12973.28	11529.2
8283.457	12810.18	1232.298	381.8838	2437.971	6504.511	3135.863	4703.586	5037.116	7473.355	2406.331	7640.531	6703.967	5483.552	14189.91	9850.957
492.0994	1930.898	1281.428	489.2649	69.87084	120.3771	328.4335	0	485.0267	203.2635	581.0819	155.4245	512.7006	85.52417	1488.858	53.11452
57.15475	2096.503	661.36	155.3491	1861.409	792.8182	959.2397	276.6129	674.4423	1181.674	1236.869	1242.403	2202.272	0	0	267.4778
0	1502.914	1592.318	1076.033	633.7117	998.2301	93.06098	437.9333	291.8246	122.6976	365.6854	386.0448	710.6124	619.9006	0	0
3528.403	8671.706	709.0695	785.1923	1322.964	6113.473	3756.642	5610.893	3479.718	4980.634	861.4113	649.8265	720.5682	4184.25	13958.87	12545.58
9648.175	12008.68	652.9645	690.2582	420.0387	11811.96	5544.366	6947.233	7166.582	9621.851	850.5748	4055.86	4813.867	5969.32	17536.58	15007.85
3571.409	4812.021	1573.63	5389.467	1304.593	8408.338	5073.44	2990.612	3353.677	8343.6	3181.521	4791.928	6650.914	3346.749	8752.885	6442.49
4172.053	5935.327	664.3719	315.0391	2029.48	5058.149	2452.78	3392.014	3627.928	5391.866	1731.465	4799.306	4831.94	3850.611	10520.31	7832.424
3497.974	3691.255	691.5706	678.3457	1316.8	5005.965	3177.824	3237.522	3160.37	4353.607	855.9464	645.6142	716.0675	4152.872	10648.47	9076.955
4630.299	9265.025	509.869	576.342	442.6376	4698.806	1799.647	3315.362	3737.145	5653.905	1509.767	1187.577	2305.239	3407.627	16021.53	11892.23
0	8771.276	5646.452	2911.211	3185.363	579.4883	245.2049	2796.635	206.6067	653.0493	581.9504	146.4434	129.8266	22.23993	0	0
0	1507.612	2070.976	3273.699	4546.782	0	0	302.2166	0	0	0	0	2418.07	0	8460.832	2477.113
8268.188	12229.29	537.1023	566.096	342.4713	10721.2	4511.681	6442.875	6090.082	8052.843	702.3997	2222.029	3584.093	6891.185	20839.91	16345.42
3565.875	2739.856	215.2258	450.2809	822.5431	2148.662	824.4408	2195.852	2032.345	2953.366	468.2294	408.0753	345.1109	1796.152	7031.701	4730.678
4665.61	6161.123	112.9035	279.7835	0	1274.325	734.1102	68.42696	3366.576	4281.509	412.5709	839.0687	1114.195	4096.156	14773.29	10725.94
6795.978	8854.003	539.3832	255.3364	1650.96	4445.752	2040.877	3351.053	3337.527	4781.24	1499.707	4300.808	4516.037	3812.914	12830.56	8416.437
6502.889	8495.03	773.0006	436.8616	992.7632	4917.443	3376.434	3001.319	3800.668	5885.452	1790.337	3472.382	6124.045	4195.436	12039.57	8171.704
495.4738	2374.455	366.3731	287.0726	875.7469	3966.426	2274.222	595.2294	448.4326	1598.794	671.8115	3490.058	4070.351	524.1622	512.2331	129.8701
28.43588	533.0923	2291.731	949.1578	3698.179	2352.644	3597.842	1695.656	5153.179	3521.742	3738.549	384.9363	430.1778	0	4452.126	129.2184
4046.642	6355.991	988.0723	2460.068	1362.026	3326.161	1115.897	1391.003	2278.449	3202.021	284.9213	558.946	958.4751	3644.374	16815.69	12749.52
3466.053	4002.071	296.6853	405.0614	130.6353	2951.631	1158.312	2192.229	2269.521	3273.571	690.3901	567.442	408.9364	2385.707	8822.834	6199.326
1358.882	2301.241	881.3068	930.8337	207.3529	2237.271	711.601	45.0037	1183.509	915.9067	174.9721	468.8449	1536.598	260.2414	2231.75	0
3037.512	1155.341	137.7605	174.214	356.1139	1486.754	616.4321	412.384	1602.909	2158.73	2570.926	1439.318	3928.43	13.06106	49.52626	122.4024
3358.135	1177.942	137.5569	174.7081	354.8017	1530.839	618.6338	414.2584	1672.012	2273.445	2930.188	1191.586	4200.035	13.06355	49.56204	122.6912
5269.569	8866.661	1022.821	870.0227	1528.594	6669.925	3365.02	3512.286	3247.293	4563.862	2094.261	2553.569	3346.807	7141.856	18988.32	14268.94
53.03439	829.1025	4056.902	3204.492	3679.876	6278.118	5468.155	3176.435	4402.79	7192.62	2972.619	1983.635	3056.333	252.1097	1568.144	4598.336

5763.167	12487.12	0	743.1421	0	5378.102	2337.215	2811.672	4508.702	5079.608	900.9667	450.986	1332.794	5926.918	18187.49	15359.63
0	875.3258	1534.747	3384.641	2380.811	235.5544	143.3323	405.954	71.9642	143.7077	95.27558	103.7505	1275.266	0	4197.343	879.2996
2675.549	3707.209	571.2148	252.4179	281.8115	2180.378	895.3252	2177.008	1570.116	1750.977	61.94288	221.6745	400.3032	1862.904	10336.29	8366.553
4098.486	5894.06	124.9909	411.5057	1184.816	5014.86	1551.481	3462.507	3068.516	3737.438	321.6221	412.9262	1479.148	4276.56	14734.79	11814.07
1822.662	5613.42	2190.104	1397.604	1015.377	2274.488	558.4292	1741.206	2112.131	2826.484	462.2512	1705.741	2221.724	3854.375	13792.72	10482.13
3922.43	5692.388	118.1156	389.6041	1124.159	4852.305	1492.406	3383.444	2967.265	3572.416	304.4726	390.7592	1401.351	4243.114	14567.16	11807.74
3221.078	4718.886	0	646.3399	0	5726.387	2972.088	158.5855	3366.891	3882.295	694.1368	958.3397	629.5733	3386.629	5744.133	4701.361
3837.401	5157.853	0	834.9854	188.1221	5515.121	2480.489	4484.037	3228.206	4105.862	891.5031	418.97	0	3599.876	12973.28	11529.2
3837.401	5157.853	0	834.9854	188.1221	5515.121	2480.489	4484.037	3228.206	4105.862	891.5031	418.97	0	3599.876	12973.28	11529.2
454.4592	0	29.39185	321.1482	0	1311.651	31.81178	790.6309	111.7401	167.2935	257.3635	551.3112	44.48907	28.14757	0	0
4720.486	6652.709	1215.986	703.4045	2412.621	3548.673	2649.94	2972.256	4715.337	5492.556	1625.376	2093.162	3792.005	4478.177	15449.63	11122.39
4720.486	6652.709	1215.986	703.4045	2412.621	3548.673	2649.94	2972.256	4715.337	5492.556	1625.376	2093.162	3792.005	4478.177	15449.63	11122.39
562.7663	1057.744	1329.636	1114.343	1822.961	2312.398	3835.977	553.1492	1934.581	2460.531	1309.814	2758.51	1959.745	1729.845	795.0803	157.762
0	2723.918	0	29.80836	0	1632.91	465.0971	0	907.8216	1191.389	170.9438	0	0	0	5257.729	3936.362
1321.792	1568.247	3435.472	491.172	2591.898	2261.479	911.1173	2097.371	1921.294	1222.572	4035.54	807.1787	1255.22	737.2741	5412.122	4396.798
2066.409	4436.84	314.8391	350.6068	594.4076	3078.909	1826.303	2816.738	1786.305	2579.892	388.1423	289.6938	327.2922	2070.803	8728.46	7123.578
527.1856	1110.739	499.2971	426.3208	444.5943	2086.075	693.674	1652.336	557.4663	1332.257	736.4909	107.1541	206.2483	513.5146	2806.51	1708.581
3559.29	5283.969	0	228.027	0	3787.346	1217.145	2901.069	4582.038	5384.724	564.5713	545.7503	301.4114	4183.153	11983.63	8318.055
1502.994	123.7479	1485.184	54.68394	824.8101	1770.697	2327.216	946.8524	5569.056	4344.182	4613.757	171.9423	164.7701	0	2983.622	120.6544
3274.323	6542.583	338.1941	498.8967	1311.598	1050.864	252.2128	1375.344	1991.813	3266.887	1564.408	226.1078	1453.937	978.3679	4928.17	4159.408
4030.179	8068.054	140.2674	378.8158	0	3047.669	1255.464	1533.686	2915.182	3124.286	525.0852	230.584	765.8786	4049.268	13775.11	11488.4
4115.076	6359.403	0	147.809	0	3458.022	1226.48	2371.258	2507.628	3342.012	206.0958	355.3539	423.5989	3391.344	16346.04	12475.18
5218.844	6069.95	0	210.1878	40.64255	4535.848	1946.515	3387.648	3078.492	3873.161	179.5469	662.0187	1126.227	2941.272	10874.71	9388.886
7462.434	9744.985	241.94	256.4001	154.2308	6488.189	2345.941	3736.265	3883.889	4841.571	321.1895	1725.763	2104.51	6996.288	22240.68	19348.06
3438.115	5276.89	0	191.7096	0	4060.533	1499.334	232.9002	2748.129	3430.729	200.4138	608.8046	366.5733	3528.295	7339.162	5925.337
2994.212	3691.255	0	563.1368	125.6589	3944.607	1706.126	3237.522	2278.962	2889.749	602.5442	280.7948	0	2497.696	10648.47	9076.955
3445.02	4555.937	379.1991	199.5835	886.5229	2470.5	1495.278	2161.824	3123.005	3617.116	860.5492	1076.276	3231.834	2292.212	10289.36	7593.925
5563.167	6360.319	0	198.7738	0	4550.898	1504.519	2503.544	4202.704	4471.804	282.7231	287.9259	349.1653	5432.397	18899.3	14157.45
3684.463	4472.203	60.35585	524.4346	0	2209.494	759.779	1573.513	2263.452	3262.478	131.4639	523.5142	429.6881	2454.798	8617.945	6055.087
3236.005	4810.697	0	172.6716	0	3994.525	1453.389	193.8618	2498.904	3106.643	167.5999	561.1621	309.7926	3364.526	6379.031	5139.821
5286.451	8301.069	57.17778	192.7877	653.4592	4406.571	1276.506	3522.756	3241.336	4053.189	342.529	300.4549	947.3013	6289.221	18712.93	15231.9
5513.379	7950.406	57.16283	192.5349	651.1574	4281.086	1242.404	3331.366	3118.18	4021.229	341.4232	299.8349	950.8454	5544.365	17321.09	13755.36
2632.069	2739.856	48.71448	171.1902	197.9367	1674.219	519.7908	1124.919	1432.549	2256.403	144.9253	293.9699	609.9211	1796.152	7031.701	4949.37
18.29705	11.24872	606.3429	153.8635	1143.077	276.2169	49.64065	53.74324	76.29176	171.3685	69.59386	33.02861	127.2601	16.97047	3.218993	1.744548
3253.235	2612.768	32.99057	85.50208	0	395.2318	228.4072	19.68409	1366.012	1745.096	130.3145	304.2239	457.1365	1985.185	8107.873	5849.92

1743.468	2871.274	0	89.64733	0	1739.723	768.9529	1821.536	1034.613	1459.609	142.3815	285.6931	292.9124	1584.726	6393.383	4665.953
0	0	0	180.1863	0	3638.961	1367.451	148.1365	1480.661	2913.466	419.054	493.1662	334.3149	103.7579	0	0
3267.647	5530.689	14.52756	154.1259	258.2622	2645.703	1052.357	1113.859	1845.487	2083.338	258.9615	560.4771	475.2769	3542.589	11137.62	8781.308
5024.411	7456.644	50.04743	168.9061	573.019	3926.317	1123.899	3142.764	2882.137	3623.203	300.4384	263.2066	835.7654	5643.308	17216.11	13813.51
2347.633	4603.601	226.05	334.1623	882.3072	706.8764	168.5431	930.7828	1365.777	2241.509	1061.712	151.0757	981.8033	661.2759	3464.844	2901.729
2694.914	2002.531	0	208.6078	0	1679.713	558.7689	1381.998	1569.807	2476.544	243.3602	250.3876	224.2707	765.0368	4591.657	3837.576
4512.369	4800.426	43.71213	389.0017	0	1751.912	575.95	1381.139	2125.929	2717.426	95.30357	391.0588	314.4113	2434.55	10172.37	7445.77
5337.675	5388.994	17.70292	465.0978	0	1568.53	670.1861	1616.112	2498.195	3623.372	147.8125	408.3249	311.1697	2830.8	16610.51	12968.23
396.7708	606.9611	0	39.6889	537.0927	1410.026	856.9606	1508.15	390.474	749.762	169.3025	505.4004	301.4799	1168.499	898.1061	602.6189
30.59462	452.7603	188.3395	18.18589	507.6166	61.88993	125.0251	34.51378	156.1832	325.6494	139.4707	244.0222	606.3779	0	0	15.78953
1404.609	826.7184	32.72967	211.7337	427.884	2885.456	1447.042	1771.876	1366.075	1563.996	104.8335	294.1996	1283.856	246.4846	736.1498	520.597
369.5459	626.393	341.4295	301.8248	60.53199	166.0586	105.2321	18.04819	39.367	25.40121	71.62639	165.8371	289.5792	98.19606	602.4772	0
4641.327	5043.815	17.03389	185.9028	0	2115.749	980.3017	1193.101	1951.621	2668.88	373.9116	594.8935	546.5314	4135.409	16765.75	14646.83
0	0	57.58387	0	0	340.933	462.7641	91.85458	0	908.5467	544.929	0	0	0	0	0
3613.744	3862.145	13.1491	88.48049	164.8207	2281.112	700.2636	830.5325	1577.674	2020.934	147.0195	617.7741	460.6759	2395.575	11418.93	8604.093
1459.824	5308.874	13.15071	179.525	0	639.2792	430.4012	4611.458	2433.497	3875.27	328.819	149.0402	87.18785	274.6134	14387.39	11845.95
2812.902	4087.875	24.57125	109.2788	0	1954.399	1458.666	11.57377	1551.261	1562.009	325.5937	254.2659	320.1967	4027.386	10837.39	8192.701
2281.143	3437.81	170.5849	167.0017	54.65366	1447.013	450.3611	1503.246	1347.743	2152.937	446.238	197.57	196.3229	2025.911	8486.781	6428.559
0	221.1916	89.49924	41.27019	231.4278	348.9177	83.77523	126.8451	33.07367	72.66407	114.5348	0	94.21466	0	0	0
0	221.1916	89.49924	41.27019	231.4278	348.9177	83.77523	126.8451	33.07367	72.66407	114.5348	0	94.21466	0	0	0
0	221.1916	89.49924	41.27019	231.4278	348.9177	83.77523	126.8451	33.07367	72.66407	114.5348	0	94.21466	0	0	0
690.1643	573.8783	482.001	953.9483	49.22271	612.6112	759.3471	214.3993	198.3487	465.3354	13.81865	106.2638	405.8111	16.10483	2363.213	1983.91
2963.158	5152.947	45.93784	59.54754	0	3105.954	912.6113	1873.699	1729.904	2294.344	338.8966	299.7466	200.6409	3389.977	9252.838	7342.255
403.5526	437.0805	433.8019	273.7028	789.7245	2205.468	1456.114	1117.954	1838.999	2734.316	627.7872	270.27	1522.932	575.483	1423.525	1140.072
1719.008	2742.81	0	0	0	473.2667	146.8189	0	1677.116	2106.415	59.21536	218.3607	242.5753	1899.034	8558.037	7325.582
2359.615	6524.318	0	153.4906	0	555.7135	334.7034	0	775.6313	2366.965	36.04778	458.5935	309.7825	921.9667	996.4644	764.7179
2085.425	7092.782	0	118.968	0	1240.721	474.6632	255.8634	1795.236	1850.826	245.437	278.3271	257.7025	1791.44	9969.466	7306.069
3286.241	4648.616	28.61603	96.75423	329.4107	2353.075	652.418	1903.332	1763.009	2173.07	172.7221	150.9185	482.3824	3570.975	12551.68	9690.482
0	20.61866	0	6.338697	0	0	65.41067	129.1168	0	0	35.57732	0	0	0	0	109.059
0	502.7045	27.44608	761.9314	903.7228	21.58133	36.35203	60.58974	31.33937	34.36076	350.2567	1810.524	964.9159	0	0	0
0	0	18.62774	106.2619	236.9241	1903.823	881.0229	661.3736	899.5559	996.7096	61.68378	171.1562	786.733	175.2298	0	0
0	115.1724	270.3487	227.8572	846.8697	0	0	0	0	0	0	0	423.1343	0	2034.017	504.6418
2861.436	2898.557	50.56804	89.09972	201.9517	1454.091	529.6886	48.24386	1422.083	1920.778	104.1197	253.451	661.39	1714.038	6977.822	5186.646
2335.019	3199.429	269.3521	872.7153	45.00693	4162.134	1692.845	153.1638	1901.502	2248.052	560.2289	215.7873	182.0996	1899.289	4922.563	3716.67
151.5643	1173.523	382.3721	905.2274	400.304	289.3401	396.9189	123.4751	200.6018	470.9719	373.8465	401.8736	1279.96	326.5837	195.2824	590.5726

0	0	2476.717	2369.592	738.2234	733.3207	2239.68	0	749.1303	1316.586	2305.957	2013.681	5050.638	0	0	0
1995.341	2298.428	127.6409	61.7346	106.5957	520.714	364.6715	80.19356	1826.539	1996.847	193.9171	207.8508	686.6534	2419.045	8108.287	6255.258
2968.74	4138.068	30.18014	108.3172	0	1458.049	1178.671	14.30253	1585.928	1654.233	381.5143	306.0323	393.1892	3221.168	10216.95	7652.843
243.9764	59.13337	18.2845	99.95592	189.8531	2633.294	990.7145	322.5871	784.6468	977.025	360.2202	147.5008	297.7474	170.7693	1681.013	119.878
760.1482	2159.197	297.8614	525.5072	162.4901	499.6926	81.57753	52.87198	680.5536	1232.186	550.7599	1993.608	1277.251	0	2521.121	974.1897
1478.541	3025.879	42.34114	100.6475	0	793.3262	324.9031	375.0221	979.4043	980.7827	174.7103	54.06481	259.0906	1493.131	5741.345	4316.128
0	0	17.39244	56.44486	188.0731	1096.787	424.7754	161.9031	426.6304	507.0115	42.79691	116.584	729.9406	130.8742	0	0
272.7298	621.1885	151.2038	300.1295	247.7882	3147.549	802.5284	102.5153	343.5519	2051.695	249.5784	170.1741	389.6588	92.63056	1225.291	151.6739
2437.543	2569.739	0	78.29485	0	1582.931	645.5325	24.45397	1263.418	1855.549	156.4002	442.7832	239.5763	1580.444	7307.78	5199.623
59.71163	42.87383	184.5638	72.89713	0	0	0	0	0	0	0	0	315.8793	0	1572.846	363.4816
3229.694	3998.725	0	249.155	51.88462	3180.944	1047.808	1912.004	2029.716	2292.496	279.31	269.2376	344.8715	2421.373	10081.89	8359.292
43.91813	1258.821	0	21.57309	0	521.2862	371.8924	205.8937	410.9198	708.2945	69.22811	135.7944	142.226	0	0	203.0822
2100.315	1999.848	0	15.79262	0	1229.723	393.3075	1497.875	771.4121	1107.006	51.22464	153.3705	70.10689	1358.497	5924.809	4330.82
927.9369	1480.229	5210.847	3072.912	13257.97	5520.704	10943.48	967.3767	919.069	711.5079	233.0745	209.4495	350.7328	1171.425	3033.295	2334.585
0	0	232.205	0	53.08303	71.21136	0	0	190.7479	298.5587	107.6002	443.2941	0	0	0	0
628.4041	956.3032	0	15.62952	0	2246.271	703.2647	390.8874	707.5374	697.2914	30.2791	14.15702	14.47074	519.5829	3118.394	1397.371
1664.245	1871.75	42.44435	100.3141	0	2084.898	607.0433	1226.422	677.5995	1146.912	46.6299	346.2637	81.58852	940.224	4351.356	1402.66
1384.546	2156.662	14.81661	64.29226	0	1422.843	448.7882	773.3253	661.4516	840.4826	35.32547	128.6999	125.3651	849.91	8123.803	6543
0	105.7421	40.75158	19.41431	123.2017	164.6313	38.51433	59.53871	14.81569	32.7544	62.10679	0	45.74377	0	0	0
10280.74	100.8731	2375.067	105.7773	1112.993	2947.904	2368.696	4514.676	213.8376	0	300.4301	0	43.55807	0	121.2194	67.00747
0	875.1413	212.125	13.0918	0	56.35878	0	0	27.90163	429.0715	310.2048	0	0	0	0	0
953.7284	1885.343	0	47.67747	0	1183.615	301.1211	30.87851	1401.607	1652.915	98.76429	169.0044	109.6982	72.9267	4092.421	606.1075
1384.388	1946.343	0	11.92059	0	1802.394	644.5029	834.3514	1029.385	354.6687	14.86941	75.82843	56.29788	1196.809	1581.314	657.4684
607.1758	1736.408	0	44.58819	0	333.6006	187.1893	909.0203	805.2249	1245.939	76.95408	48.58477	59.45632	5.553266	5757.254	4809.966
33.25306	308.7882	0	0	0	68.33813	0	0	1177.727	912.9103	58.66666	0	0	0	5050.8	4677.932
2310.233	1837.336	0	34.14503	0	1000.507	334.229	10.67694	1013.656	1438.509	88.41046	222.8904	121.5158	1439.735	5796.319	4285.649
2310.233	1837.336	0	34.14503	0	1000.507	334.229	10.67694	1013.656	1438.509	88.41046	222.8904	121.5158	1439.735	5796.319	4285.649
0	0	0	13.05048	0	94.40347	17.96559	0	30.65439	55.73988	8.308919	0	0	0	2923.284	1648.509
2889.473	62.17903	1874.924	31.69988	905.1435	3026.125	2750.716	1383.896	3226.207	3443.222	4407.015	36.97668	914.9946	0	0	0
112.2939	459.5844	0	10.91325	0	103.7323	13.1407	66.0597	382.4277	341.8735	8.859055	14.07067	14.2742	285.6589	133.014	358.4167
728.8518	674.8101	0	6.70574	0	999.3426	307.6634	1515.869	1066.643	1265.654	93.12833	63.53803	64.06765	907.9813	2185.583	1910.146
1493.277	1555.311	0	34.69991	0	1620.292	425.7537	534.5051	1062.905	1386.28	58.88743	115.8518	97.01486	1211.446	6215.031	4793.142
659.1359	586.0953	0	5.835987	0	960.9898	293.0455	1637.493	1129.532	1258.242	105.5947	57.86652	57.91669	832.1727	1951.026	1743.047
29.71788	162.1519	0	94.22068	70.67942	87.28992	90.65421	16.3016	137.3762	111.3036	338.9652	609.184	1267.883	0	0	0
37.7999	937.6047	0	49.78653	0	426.7245	251.3595	155.7469	373.2095	604.9559	64.84918	53.7054	48.96327	0	0	176.3254

1298.971	536.9257	93.46148	0	119.5332	1232.359	2459.29	941.0663	6396.402	3388.748	679.7056	239.6915	402.0938	0	1880.197	0
0	0	995.6464	970.2244	282.1593	280.4947	907.0799	0	291.03	517.8959	953.6625	801.0731	2495.421	0	0	0
71.32443	336.4769	0	7.274892	0	49.30632	6.125013	32.29962	272.3621	203.4709	4.543467	60.04791	33.69313	296.6432	978.3038	765.7679
600.2775	1271.675	0	28.64634	0	740.1517	181.8964	18.5617	908.4214	1045.838	59.54308	102.2431	66.09773	44.2005	3270.544	378.4471
260.235	583.9721	0	109.866	63.1361	1362.309	719.1986	70.12237	164.1546	92.26533	95.74209	146.4026	593.8392	124.3555	3376.211	1940.441
122.9262	470.5422	0	11.78644	0	77.99065	9.963993	52.7603	0	0	0	87.86168	53.26578	37.24127	22.87936	13.6247
786.7414	892.5286	0	52.30356	0	138.1733	87.77083	0	583.6809	650.3116	32.64089	43.32532	28.07449	891.7146	3408.814	2977.418
397.5396	1041.558	0	0	0	1434.759	387.6086	722.5188	559.7852	499.9132	22.6066	66.55963	24.91387	874.0918	3992.027	3182.195
7.627639	84.30613	0	12.19719	0	16.46722	0	0	1092.812	603.8781	64.65675	0	0	0	3197.158	3513.149
520.8388	1363.551	0	0	0	73.12943	64.98541	634.4934	180.6958	151.382	57.54529	60.94552	0	859.6197	3124.943	2754.885
87.82876	341.3925	0	6.783759	0	64.49895	8.114355	42.67758	272.7554	223.0999	5.505561	8.876526	8.865719	236.6579	997.2072	608.2616
2676.468	7210.005	0	0	0	5216.634	1640.507	0	3571.249	4600.555	520.706	240.4821	0	268.5897	11566.89	10343.18
0	0	376.4537	26.41692	82.0739	188.9536	165.763	129.7168	0	0	29.46129	0	34.79628	0	51.28235	28.45599
910.4059	774.2552	0	36.42253	0	1327.967	419.3284	1706.473	836.0712	838.0748	44.11419	56.48988	52.25355	533.4906	3318.597	2768.596
249.7284	468.6136	0	45.86792	51.1475	1008.779	510.7445	68.85648	136.2716	87.77599	38.94218	139.7759	523.4748	110.9647	2112.045	1415.244
405.7792	0	0	20.83685	0	315.7687	135.3262	661.8141	498.1551	524.4265	43.7673	78.21293	62.72961	0	2190.515	2079.962
0	24.27961	0	0	0	143.6774	182.1811	76.51157	0	41.65239	47.32628	100.7672	213.0032	0	0	0
0	24.3567	49.30282	0	83.17008	142.8337	180.9091	36.40989	0	41.56887	44.20379	101.5814	478.71	0	0	0
937.1849	0	1746.192	41.87524	849.9606	2453.132	1820.659	1359.828	0	0	0	0	0	0	0	0
270.9171	227.904	0	7.743629	0	323.4686	157.4849	882.8262	200.1902	196.4224	6.411771	25.93421	8.224119	5.553266	1398.309	659.9432
0	0	0	0	0	0	0	0	0	0	0	0	0	0	0	0
0	0	0	0	0	115.4074	497.8825	0	0	0	0	0	0	0	0	0
551.5391	739.4641	0	20.35151	0	77.13587	53.53582	0	292.0941	200.5693	7.194256	33.25863	20.64721	557.0835	1474.062	1036.864
2397.088	7096.823	0	0	0	4643.901	1520.842	0	3243.328	3687.101	339.7548	142.0642	0	232.1981	10806.9	9822.676
420.3966	626.7003	0	16.68559	0	61.73978	41.38343	0	215.6051	159.7915	5.792053	9.295113	16.90534	368.4453	1242.095	876.6723
420.3966	626.7003	0	16.68559	0	61.73978	41.38343	0	215.6051	159.7915	5.792053	9.295113	16.90534	368.4453	1242.095	876.6723
0	0	0	0	0	338.8152	91.64727	0	0	5.397047	0	11.81385	65.11849	18.32935	0	0
0	0	0	0	0	0	48.6469	0	0	0	0	252.1188	41.32002	0	0	0
343.6229	416.8174	0	18.60168	0	124.8838	45.20244	666.1403	256.6035	178.8658	0	0	0	366.6435	1744.987	1408.113
0	0	0	32.14512	0	127.157	0	33.99102	0	0	0	24.11798	33.86801	0	154.8975	0
1489.74	2826.137	0	0	0	1898.319	621.6955	0	1677.967	1944.173	165.4874	100.699	0	191.5298	6269.952	4672.932
1398.933	219.9042	0	0	0	1224.907	343.4123	80.03093	347.014	661.874	16.89097	86.25814	60.86676	651.7917	4865.412	3627.602
1398.933	219.9042	0	0	0	1224.907	343.4123	80.03093	347.014	661.874	16.89097	86.25814	60.86676	651.7917	4865.412	3627.602
16.50135	67.10358	0	18.06651	0	423.5274	89.5476	0	88.24602	73.11597	0	47.31952	20.26902	118.2858	0	0
0	0	0	0	0	0	0	0	0	0	0	0	0	0	0	0

682.0033	1115.766	0	0	0	341.8714	110.5051	820.5229	300.5383	370.4338	20.21823	47.44629	20.24316	363.6453	1554.934	1199.602
385.1621	344.9713	0	0	0	133.3787	57.44134	0	425.8412	598.5212	9.652274	10.92866	5.729706	487.206	1954.205	1648.869
164.8397	187.7383	0	0	0	54.17803	27.72543	415.6479	248.0621	281.4908	9.899497	21.10906	0	299.0743	805.9964	599.3929
406.8943	343.3402	0	0	0	133.2088	67.82335	11.34542	408.6596	602.1721	8.66656	9.802035	5.231241	459.8454	2601.626	2138.239
0	0	0	17.57389	0	711.0906	199.1939	737.6274	73.44927	16.45371	0	0	0	3.661266	0	0
0	0	0	0	0	72.28446	29.87921	0	27.20325	28.14705	33.83751	0	15.27495	0	0	0
347.2105	64.39976	0	36.98659	0	242.5036	95.93498	0	0	253.1793	4.762636	21.01062	28.64317	27.71019	43.22053	0
1613.73	2096.228	0	0	0	2413.146	726.0851	134.8679	729.069	809.4836	106.5271	45.6898	122.0989	0	478.7181	425.8945
62.19799	0	0	7.003571	0	21.5941	87.75537	0	0	36.66295	27.18195	0	3.734098	0	125.3177	0
241.7653	201.9832	0	0	0	77.26967	39.41403	6.496449	243.2381	358.8293	4.997448	5.618573	2.995782	276.926	1586.093	1321.942
90.77222	346.4786	0	12.3953	0	55.07271	18.0779	55.40167	117.8129	102.0168	12.88777	42.40257	20.65088	0	0	45.58294
19.16789	184.7969	0	0	0	339.9155	165.6909	70.69934	120.9884	55.22082	54.42472	0	0	3.448694	494.8899	18.91758
838.8745	598.1862	0	3.049344	0	180.8468	57.88095	848.5143	411.8136	370.5392	28.18176	423.7376	420.1509	231.529	1786.191	1108.496
16.67916	30.86275	12.91871	40.60491	22.44893	76.6495	142.06	23.58654	32.43705	6.075227	44.47236	31.34703	266.628	11.14078	0	112.3737
355.0223	204.9993	0	0	0	101.8749	56.19791	0	296.9594	228.3361	0	33.29731	12.58474	357.9957	1029.626	176.0935
0	0	0	0	0	0	0	0	0	0	0	0	0	0	0	0
322.8188	183.2307	0	0	0	90.93286	50.2946	0	268.1086	205.0744	0	30.14535	11.30987	323.0995	927.2332	156.956
0	0	0	0	0	0	0	0	0	0	0	0	0	0	0	0
595.7674	66.17024	0	0	0	23.95962	39.85068	0	118.2966	239.463	5.61181	36.52513	24.5844	250.3921	2151.924	1826.151
795.5825	578.7423	0	3.092509	0	180.5336	58.60003	960.7562	394.5244	365.0519	28.87812	34.70577	59.79305	226.5158	1728.102	1071.804
686.0103	506.0089	0	36.62044	0	863.5995	297.2191	1059.736	366.031	505.4624	12.13223	38.08125	9.271003	309.0783	3325.897	2440.585
0	0	0	0	0	338.8152	90.11469	0	0	0	0	0	0	18.32935	0	0
0	0	0	0	0	0	0	0	0	0	0	0	0	0	0	0
0	61.02359	0	111.7587	20.53333	37.08418	27.8945	0	103.1063	47.03411	171.7137	16.58164	136.7607	112.2182	155.9858	223.6901
0	0	0	112.7481	0	1717.92	861.6136	0	1279.558	1942.467	158.4101	0	250.1323	0	7724.894	5382.738
0	42.75337	0	79.40426	14.38098	25.99254	19.55228	0	72.76593	33.00051	128.5784	11.61195	96.3958	79.09583	109.5457	157.0621
0	1106.914	0	239.8332	0	219.4116	0	0	558.9701	266.2918	196.5732	0	558.312	0	0	0
0	0	0	0	0	0	0	0	0	0	0	0	0	0	0	0
0	65.85281	0	0	0	37.54037	0	0	41.59149	21.87452	0	0	0	0	0	0
1062.937	366.6646	0	40.3953	0	1189.524	421.3685	131.9411	331.7225	467.7244	5.137903	58.91035	79.28642	281.0522	4907.203	3819.986
414.3303	1498.659	0	0	0	32.91072	61.35497	11.31328	490.1016	530.9085	28.84033	0	0	938.2503	760.2511	35.35172
0	0	0	0	0	1491.376	355.0095	25.67944	10.58466	0	0	0	0	0	0	0
0	0	0	65.31543	0	14.44349	10.6867	0	131.4836	110.6823	105.0714	15.11502	151.5445	0	98.6525	171.549
12.86519	92.78439	0	151.2898	16.80547	48.69026	13.80967	36.62327	119.5004	96.51571	104.0387	50.2401	203.4226	0	90.41025	166.2505
325.017	0	0	4.955503	0	620.9829	295.4536	19.89849	183.717	337.1155	7.592243	50.43281	40.82876	0	0	0

0	90.10435	7.966297	14.09684	0	41.9554	0	24.25197	10.49382	0	0	27.13944	20.27599	0	14.81096	0
0	0	0	44.20222	0	0	0	0	0	0	0	0	0	18.04413	133.6274	0
0	0	0	0	0	895.295	294.0319	748.9942	97.65007	26.78091	0	0	0	0	0	0
0	0	0	0	0	1172.092	357.6038	55.12965	55.98134	21.93114	0	0	0	0	0	0
0	0	0	0	25.90904	0	0	0	52.37283	92.19312	86.94293	0	0	0	0	0
490.7611	797.4855	0	0	0	66.33348	16.29335	0	256.6621	185.2705	0	5.826559	0	0	1810.66	1570.324
0	0	0	10.40445	0	886.5596	245.7642	701.7255	75.00658	19.66844	0	0	0	4.944218	0	0
0	0	0	10.40445	0	886.5596	245.7642	701.7255	75.00658	19.66844	0	0	0	4.944218	0	0
0	2500.718	4562.994	2439.856	850.1279	114.8418	130.6872	53.70232	22.02426	75.21809	41.61947	147.3871	129.8933	0	0	0
0	0	0	0	0	82.15146	0	0	76.67971	50.56827	0	0	0	37.72849	0	0
0	0	0	0	0	63.78018	0	0	64.95841	40.07771	0	0	0	30.32208	0	0
490.7611	797.4855	0	0	0	66.33348	9.299678	0	249.8115	125.2935	0	5.826559	0	0	1810.66	1570.324
0	0	0	0	0	39.00332	0	0	19.01618	10.08691	0	0	0	0	0	0
0	0	123.2306	29.77331	427.2715	62.06651	0	494.1941	60.96567	32.42053	135.9309	31.85021	15.80668	0	0	0
0	0	0	0	0	43.7625	52.34257	0	42.60791	38.42765	0	0	0	0	0	0
0	0	0	33.84711	0	720.3042	157.634	13.66802	49.89709	56.99661	0	0	0	0	0	0
1941.727	328.5674	0	59.75104	0	2192.51	481.9375	0	508.9805	531.7443	30.03719	0	0	29.28592	1886.102	129.5014
1922.113	314.1189	0	57.67667	0	2230.104	474.7915	0	493.2789	514.9475	28.78484	0	0	27.93697	1815.435	123.5633
0	0	0	0	0	308.3252	389.5418	92.37225	296.8802	267.5716	208.7737	0	0	0	0	0
0	0	0	0	0	797.1995	171.9702	228.8544	37.25307	14.58041	0	0	0	0	0	0
0	0	0	25.12727	0	27.88073	95.71678	0	13.65049	48.40677	14.44354	0	144.5526	0	149.0639	0
0	0	0	124.3321	0	1102.552	288.5879	0	21.21402	0	0	0	0	0	0	0
0	0	0	0	0	26.56236	0	0	12.63935	28.0648	0	0	0	0	0	0
0	0	0	0	0	816.8286	164.4363	182.2332	30.67296	16.39899	5.834884	0	0	0	0	0
0	0	0	5.82119	0	364.4106	141.5012	0	17.89124	0	0	0	0	0	0	0
0	0	0	0	0	0	0	0	0	0	0	0	0	5.711891	125.2229	0
0	0	0	0	0	0	28.82964	0	8.388832	0	0	0	0	0	0	0
0	0	0	0	0	347.6241	151.7689	0	0	0	0	0	0	0	0	0
0	0	0	0	0	0	0	0	0	0	0	0	0	0	0	0
0	0	0	0	0	158.9113	105.376	7.409894	0	0	0	0	0	0	0	0
0	0	1719.889	346.4574	10436.89	0	0	0	157.4539	92.56234	2458.341	7012.901	5479.115	9026.29	0	0
0	0	0	0	0	110.2002	0	0	587.6689	130.709	136.8038	0	0	0	0	0
0	0	0	0	0	110.2002	0	0	587.6689	130.709	136.8038	0	0	0	0	0
0	0	0	0	0	110.2002	0	0	587.6689	130.709	136.8038	0	0	0	0	0
87.66059	0	8.026994	0	0	45.17899	1.839226	0	47.65079	48.43433	57.54529	60.94552	0	0	418.8566	199.1672

0	0	0	0	0	72.58902	33.61	0	0	0	0	0	0	0	377.8127	0
0	0	0	0	0	0	0	0	0	41.65239	47.32628	100.7672	0	0	0	0
109.1716	272.6587	0	0	0	77.75872	0	0	150.1396	162.4726	0	0	0	0	1447.905	1113.326
92.22589	234.0342	0	0	0	64.57376	0	0	140.5922	151.2593	0	0	0	0	1319.701	1001.765
0	0	63.62879	0	230.5834	0	0	0	0	0	0	0	0	0	0	0
0	0	0	0	0	15.24223	59.03255	0	0	27.35908	0	0	13.84347	0	0	0
0	0	0	0	0	15.93421	70.97844	0	0	28.92394	0	0	13.32466	0	0	0
0	0	0	0	0	0	0	0	0	0	0	0	0	0	0	0
0	0	0	42.60102	15.98137	0	0	0	11.15359	32.75947	18.88313	0	0	0	0	0
0	0	12.18852	0	0	0	0	0	0	0	0	0	0	0	0	0
0	0	0	69.45659	0	0	15.85007	0	53.91179	27.54251	82.21393	0	64.96358	0	0	54.08866
0	795.6534	1188.937	869.9197	288.589	93.38845	0	486.6958	0	0	0	0	244.7171	0	0	0
0	0	0	0	0	0	0	0	0	0	0	0	0	0	0	0
0	0	0	0	0	0	0	0	0	0	0	0	0	0	0	0
0	0	0	0	0	0	37.52508	0	0	0	0	0	0	0	0	0
0	14.09782	0	0	0	0	0	0	0	9.481243	0	0	0	49.81446	0	74.35232
0	0	0	0	0	0	0	0	0	0	0	0	0	0	0	0
0	13.72587	0	0	0	0	0	0	0	9.399611	0	0	0	45.22201	0	74.91506
0	0	0	0	0	0	0	0	0	0	0	0	0	0	0	0
0	0	0	0	0	0	0	0	0	0	0	0	0	0	0	0
0	101.0483	0	0	0	40.33574	0	0	16.60517	17.65097	0	0	0	0	0	0
0	0	0	0	0	0	0	0	0	0	0	0	0	0	0	0
0	0	0	0	0	0	0	0	0	0	0	0	0	0	0	0
143.8817	38.06375	0	14.68838	0	25.43411	57.36246	0	38.06237	866.5981	30.36439	67.95649	600.536	0	32.18127	25.24219
0	0	0	0	0	0	0	0	0	0	0	0	0	0	0	0
0	0	0	51.73105	0	0	0	0	0	0	0	0	0	0	0	0
0	0	0	52.21482	0	0	0	0	0	0	0	0	0	0	40.17237	0
0	0	0	0	0	0	0	0	0	0	0	0	0	0	0	0
0	0	0	17.30578	0	0	0	0	0	0	0	0	0	0	0	0
0	0	0	34.71204	0	0	0	0	0	0	0	0	0	0	0	0
0	0	0	33.52971	0	0	0	0	0	0	0	0	0	0	0	0
0	0	0	0	0	0	0	0	0	0	0	0	0	0	0	0
0	0	0	12.78308	0	0	0	0	0	0	0	0	0	0	0	0
0	0	0	19.13626	0	0	0	0	0	0	0	0	0	0	0	0
0	0	0	0	0	0	0	0	0	0	0	0	0	0	0	0

0	0	0	0	0	0	0	0	0	0	0	0	0	0	0	0
0	0	0	0	0	0	0	0	0	0	0	0	0	0	0	0
0	0	0	0	0	0	0	0	0	0	0	0	0	0	0	0
0	0	0	0	0	0	0	0	0	0	0	0	0	0	0	0
0	0	19.15128	0	0	1001.885	223.5227	0	0	0	20.02218	0	0	0	0	0
0	0	0	0	0	0	0	0	0	0	0	45.73355	0	0	0	0
0	12.52894	0	0	0	34.50574	14.15002	203.8253	0	0	0	0	0	0	0	0
0	0	0	0	0	0	0	0	0	0	0	0	0	0	0	0
0	0	0	0	0	0	0	0	0	0	0	0	0	0	0	0
0	0	0	0	0	0	0	0	0	0	0	0	0	0	0	0
0	0	0	0	0	0	0	0	0	0	0	0	0	0	0	0
0	0	0	0	0	0	0	0	0	0	0	0	0	0	0	0
0	142.569	246.846	311.0638	29.31402	21.0953	0	65.61979	0	0	0	0	52.42948	0	16.03552	0
0	105.4066	181.4064	160.2901	22.1184	15.717	0	46.01126	0	0	0	0	39.55458	0	0	0
0	0	0	0	0	0	0	0	0	0	0	0	43.5222	0	0	0
0	0	0	0	0	0	0	0	0	0	0	0	0	0	0	0
0	0	0	0	0	0	0	0	0	0	0	0	0	0	0	0
0	0	0	0	0	0	0	0	112.4792	35.53665	0	73.94903	113.8511	0	0	0
0	0	0	0	0	0	0	0	0	0	0	0	0	0	0	0
0	0	0	0	0	0	0	0	0	0	0	0	0	0	0	0
0	0	0	0	0	0	0	0	0	0	0	0	0	0	0	0
0	0	0	0	0	0	0	0	0	0	0	0	0	0	0	0
0	0	0	0	0	0	0	0	0	0	0	0	0	0	0	0
0	0	0	0	0	0	0	0	0	0	0	0	0	0	0	0
0	0	0	0	0	0	0	0	0	0	0	0	0	0	0	0
0	0	0	0	0	0	0	0	0	0	0	0	0	0	0	0
0	0	0	0	0	0	0	0	0	0	0	0	0	0	0	0
0	0	0	0	0	0	0	0	0	0	0	0	0	0	0	0
0	0	0	0	0	0	0	0	0	0	0	0	0	0	0	0
0	0	0	0	0	0	0	0	0	6.445334	0	0	0	0	0	0
0	0	0	0	0	0	0	0	0	0	0	0	0	0	0	0
0	0	0	0	0	0	0	0	0	0	0	0	0	0	0	0
0	0	0	0	0	0	0	0	0	0	0	0	0	0	0	0
0	0	0	0	0	20.30062	0	0	24.53863	11.2123	0	0	0	0	0	0
0	0	0	0	0	0	0	0	0	0	0	0	0	0	0	0

0	0	0	0	0	0	0	0	0	0	0	0	0	0	0	0
0	0	0	0	0	0	0	0	0	0	0	0	0	0	0	0
0	0	0	0	0	0	0	0	0	0	0	0	0	0	0	0
0	0	0	0	0	0	0	0	0	0	0	0	0	0	0	0
0	0	0	0	0	0	0	0	0	0	0	0	0	0	0	0
0	0	0	0	0	0	0	0	0	0	0	0	0	0	0	0
0	0	0	0	0	0	0	0	0	0	0	0	0	0	0	0
0	0	0	0	0	0	0	0	0	0	0	0	0	0	0	0
0	0	0	0	0	0	0	0	0	0	0	0	0	0	0	0
0	0	0	0	0	0	18.29	0	0	0	0	0	0	0	0	0
0	0	0	0	0	17.5628	0	0	14.32683	20.22211	0	0	0	0	0	0
0	0	0	0	0	11.29514	0	0	0	0	0	0	0	0	0	0
0	0	0	0	0	0	0	0	0	0	0	0	0	0	195.6224	0
0	0	0	0	0	0	0	0	0	0	0	0	0	0	184.1658	0
0	0	0	0	0	0	0	0	0	0	0	0	0	0	120.1933	31.72486
0	0	0	0	0	0	0	0	0	0	0	0	0	0	69.12677	18.17263
0	0	0	0	0	0	0	0	0	0	0	0	0	0	0	0

AP016_3	AP017_1	AP017_2	AP017_3	AP018_1	AP018_2	AP018_3	AP019_1	AP019_2	AP019_3	AP020_1	AP020_2	AP020_3
10675109	7314196	10751330	14464088	16515073	26760654	20629126	19078423	14407470	19613216	22230899	18903819	13527581
67841868	41417511	56806555	66779927	52097473	55456224	68614485	1.08E+08	87167869	1.25E+08	94277613	78742919	57302519
47966.41	42881.44	63353.12	57270.96	33691.61	31243.95	44349.64	49399.36	50220.48	58522.14	86297.46	65401.2	50187.08
33298.02	23218.38	30241.54	27498.15	27121.55	25815.29	35530.43	41979.1	31645.39	53941.28	69786.08	54409.26	40130.23
31895.84	23376.92	33355.51	56649.06	32802.16	35136.34	42027.75	57601.15	38959.98	68536.78	68522.04	59149.71	41829.9
28697.71	21624.09	30209.68	42553.2	36729.57	33661.47	48409.38	38500.12	30683.4	46924.98	67333.56	56680.56	41220.47
28697.71	21624.09	30209.68	42553.2	36729.57	33661.47	48409.38	38500.12	30683.4	46924.98	67333.56	56680.56	41220.47
28697.71	21624.09	30209.68	42553.2	36729.57	33661.47	48409.38	38500.12	30683.4	46924.98	67333.56	56680.56	41220.47
41956.4	23977.29	31828.93	38787.44	35865.7	35809.64	46082.18	50159.4	36853.73	60605.06	65109.04	50134.13	38241.76
28861.38	23904.92	33448.23	40177.62	39491.77	29847.49	51747.67	42694.8	34066.9	51538.23	65214.49	58868.38	40798.67
31152.71	22368.16	30995.58	44194.43	33796.95	27395.13	44053.21	40392.69	33314.7	47331.29	62321.01	53767.32	37464.76
26879.45	21364.14	28839.67	37820.7	31727.66	25690	42289.36	39341.32	30483.49	46414.89	64276.97	53809.55	39256.9
22929.22	19114.47	25231.4	38465.76	25419.38	24722.25	32925	33653.83	26895.68	40411.01	64410.22	55207.88	39678.82
29825.88	21428.81	29203.4	41658.28	37520.88	26995.56	48329.38	43166.31	32513.29	51473.14	60348.53	51073.36	37082.9
29779.03	21280.17	29437.72	41767.17	38038.72	26830.1	49399.42	41104.18	30692.31	49023.33	58055.96	48573.24	36051.93
29244.19	23618.01	31058.82	34496.46	20326.01	27439.82	25272.3	41109.61	32187.94	49932.23	67212.47	56667.23	39517.46
27597.72	22822.6	31993.49	45563.09	31881.96	27022.35	41128.9	43523.91	34369.2	52618.32	67213.52	57009.25	41013.26
28031.57	21928.01	28217.31	30637.65	35857.82	28720.42	47350.04	35478.29	30609.41	40616.35	62403.61	57152.19	37414.05
29708.84	21106.54	29126.28	41361.23	37844.67	26647.14	49075.39	41485.97	30750.91	49283.92	58652.48	49340.89	36086.6
28002.35	25607.81	38466.53	42638.83	29680.23	27386.32	38430.89	45342.17	35052.43	55628.27	66061.39	57127.81	40063.19
27074.02	20535.05	27744.44	30675.27	27344.91	24589.21	35485.9	40654.05	31592.25	50067.51	60815.05	49553.78	37093.62
28789.35	21019.19	27604.63	41293.5	37817.08	26403.67	48799.97	40626.24	30866.13	48816.14	59220.73	50206.12	36471.3
28413.69	20345.02	26865.35	35891.89	37886.44	25694.99	48334.8	41948.95	30264.72	49827.31	56757.68	47060.61	35042.77
26558.7	19849.41	27570.84	32937.72	27067.84	26038.28	34690.44	40089.67	30425.99	49703.44	61226.5	49671.45	37259.29
26558.7	19849.41	27570.84	32937.72	27067.84	26038.28	34690.44	40089.67	30425.99	49703.44	61226.5	49671.45	37259.29
36165.13	22426.44	27503.64	32284.45	27136.46	31185.36	36317.68	66913.86	42873.98	80845.73	55832.65	39009.46	33578.53
24384.09	21120.24	30233.08	37792.93	29581.11	23246.38	39259.34	36898.77	27901.36	44190.13	61994.92	51841.68	37832.38
30874.51	21448.41	28376.27	46016.04	30031.57	26912.93	39886.85	39363.5	31521.31	46256.85	58420.42	47854.28	34837.68
27185.01	20026.97	27914.41	35999.62	38556.35	28309.41	49032.58	40023.45	30666.75	49559.7	57860.35	48852.02	35051.27
27274.03	20574.69	30484.83	31348.92	28747.54	22595.35	38816.59	41153.28	32646.22	50675.14	59130.98	50149.24	35413.52
27274.03	20585.13	30486.1	31381.43	28747.54	22618.35	38816.59	41153.28	32646.22	50675.14	59130.98	50240.36	35413.52
27204.18	20220.57	27237.47	34719.35	33643.78	28172.19	43095.31	40732.84	30612.79	49480.04	58345.93	50738.9	35361.59
23389	20277.08	28645.9	35812.12	27915.49	21379.32	36996.67	36456.86	26722.87	43708.54	59279.21	49742.16	35940.82

23389	20277.08	28551.76	35812.12	27915.49	21379.32	36996.67	34365.44	26011.46	41325.28	59279.21	49742.16	35940.82
31525.47	21991.51	30679.76	31607.42	25636.53	26912.3	32640.38	39521.69	32352.65	49732.67	60120.99	46540.62	36599.2
26133.37	16274.38	24051.56	49851.25	35325.24	21632.2	47767.47	35891.67	23185.67	43944.22	36894.09	27353.2	26325.53
26202.07	18298.72	27020.95	38104.32	29722.58	27812.61	38171.33	36878.3	28601.97	45237.44	54644.08	43292.98	33578.26
26028.54	17998.04	24531.72	37888.74	28230.2	26138.53	36870.96	36518.11	29934.44	44924.13	56577.01	45653.94	34613.42
26849.18	21927.83	28064.93	28123.43	14861.39	23175.99	18301.95	36775.87	29680.27	44598.55	63062.94	52787.21	36607.87
30887.75	18742.58	27345.87	43949.85	27484.38	24480.13	35970.12	38445.61	29525	45397.6	46706.35	43136.58	29856.16
27668.1	20789.49	24096.85	20137.41	21301.41	28634.64	26462.37	35332.85	29744.15	43015.19	59269.16	48900.96	34447.27
25404.05	19242.05	26372.08	28222.32	24224.79	21234.4	31516.16	40138.25	29286.54	48812.34	56651.46	47656.37	34334.07
22237.9	20437.22	26878.88	34379.23	20186.87	24035.94	26874.8	32399.12	27761.1	39692.28	58390.48	51635.77	34261.59
28436.9	18954.43	26511.93	36180.78	29664.65	26645.69	39399.51	40333.25	33281.94	49319.6	57043.52	45886.31	34018.84
24540.13	18971.18	23181.5	21321.5	28589.68	23091.16	36962.46	33794.77	23215.84	40621.83	62966.13	51764.39	37047.43
28210.28	19702.06	25275.95	30469.99	27036.76	26120.92	34563.02	41002.26	30709.3	49378.94	58115.23	47672.81	34532.39
23401.5	19205.72	26728.22	27863.02	24790.33	21518.28	32711.59	36203.81	27003.26	43386.2	56737.07	48257.96	34036.09
21495.21	20260.72	26785.51	34465.41	18989.83	23286.93	25307.83	31660.92	27240.63	38680.78	57454.33	51133.89	33696.07
23815.99	18591.48	27985.86	52398.14	28045.16	31308.1	36212.83	48074.18	33747.66	56909.37	49233.59	46460.76	29257.21
24776.06	15187.68	18164.73	31253.46	26957.86	24442.18	36114.91	25882.81	11343.42	33180.42	46841.11	37069.28	29086.66
25883.11	19865.38	22972.77	18622.62	19666.36	27957.05	24148.05	33766.37	29403.3	42852.74	56154.22	46917.31	32636.15
26856.68	18600.34	23346.51	32001.04	22347.27	21826.23	28831.05	39812.85	27543.32	48404.11	52467.24	40944.8	32275.97
21758.45	16563.08	20210.14	17286.81	18678.38	14594.72	25874.33	29526.47	25507.17	34775.1	47939.72	35422.1	27630
25766.46	21067.85	27230.28	26073.07	12784.43	20517.74	15730.4	35949.62	30007.92	43394.84	61030.82	50229.74	35263.89
22500.2	17864.18	26719.63	48894.46	26720.77	29807.44	34555.83	45335.23	32599.54	53554.55	46833.72	44497.74	27681.86
23970.67	16198.65	23977.72	33808.32	25832.07	22960.56	33726.92	35067.43	25018.37	42929.23	39789.01	35269	26218.46
22592.31	18410.75	19566.46	23981.54	17499.39	17210.66	22267.57	28470.7	24638.68	31630.23	55900.66	50836.84	33605.38
23660.55	17380.16	24859.33	28122.6	16130.36	21954.34	20211.5	34917.94	25082.38	42545.18	42268.43	39577.62	27643.53
24664.39	17077.59	20932.1	17821.91	14973.74	16127.77	19422.11	38173.22	21565.45	46775.87	45023.5	32756.13	26646.47
24271.21	20001.16	25308.88	22943.72	10850.16	18626.62	13295.7	33129.64	28068.61	39986.7	58312.9	47886.91	33408.13
20628.3	15780.27	20977.91	20198.49	19022.97	16104.27	24514.67	32886.38	26838.01	38843.32	49656.7	39267.51	28848.96
19903.17	16200.33	24835.99	47921.41	25152.29	28494.41	32540.23	42656.44	30348.35	50359.57	41555.01	40461.77	24578.24
20916.97	14828.42	20623.22	27735.02	16582.4	16099.28	21671.66	37901.62	27701.92	45964.58	43604.18	35361.95	26322.32
29494.03	17696.54	25543.17	34873.24	26145.49	28825	33624.44	40438.13	30977.58	50906.94	36309.96	31584.65	22416.74
18600.39	16066.27	18160.67	12041.71	11947.93	13551.59	14830.98	22009.46	15386.2	26138.41	44742.42	35341.83	26343.24
25173.45	13443.27	28132.81	35776.28	28059.08	19471.4	38034.23	31289.63	20352.62	38936.93	29678.63	18370.68	20360.09
23204.52	19044.34	22855.92	17990.54	7875.754	16125.34	9582.655	30045.29	25725.84	36342.76	54547.9	44262.28	30207.57
23204.52	19044.34	22855.92	17990.54	7875.754	16125.34	9582.655	30045.29	25725.84	36342.76	54547.9	44262.28	30207.57

19924.67	17748.72	24490.43	27617	17708.44	15212.12	21781.98	31379.42	26914.12	36302.96	40296.6	34911.79	23224.49
19594.96	17427.7	18064.17	17432.24	12099.3	14769.54	15977.39	25868.27	21824.03	29013.82	40026.25	30685.9	22874.61
21079.79	12813.14	13408.39	4409.441	11056.71	13302.87	14222.46	29804.4	23982.04	33036.18	33258.77	19753.21	18336.45
19541.83	17739.93	25085.58	26904.13	16881.01	14392.23	20658.55	31370.39	26843.35	36194.35	38664.54	33462.43	22206.68
21855.75	14528.76	19472.91	36471.15	19586.76	21359.04	25336.49	30922.11	20368.81	37036.52	24539.41	34259.15	15481.79
23169.64	14038.4	19836.18	44536.16	23489.74	17812.73	31338.68	31762.42	21219.34	40086.61	28527.58	22632.95	17845.35
16774.06	12492.98	18784.09	21131.66	17528.45	12442.35	22687.28	22714.58	13813.01	28261.99	27914.82	27273.9	17377.12
16586.21	8292.721	10137.51	10944.47	14609.81	14901.33	19324.73	34887.08	22962.14	41269.21	28180.35	12636.83	16005.83
15491.12	12946.72	16012.38	8497.002	10829.27	11477.13	14458.14	20957.43	21463.94	23448.66	40671.91	34705.78	24171.34
19596.43	19591.93	33243.92	29047.62	21751.47	19702.34	29620.24	26662.87	17544.76	35134.33	19068.88	24565.95	14761.25
20212.25	10641.38	20270.54	35048.41	26236.53	20006.8	34097.92	28044.52	15545.37	34790.86	19330.17	16875.97	14793.81
21614.26	12015.26	17842.73	32986.19	14287.98	15806.54	17187.65	31692.43	26678.07	39583.92	16720.59	30327.62	10631.98
14754.66	12561.96	13847.82	5214.051	8194.251	8023.334	10973.37	18468.65	17960.67	20830.35	37238.08	30157.37	21914.88
20232.92	14705.26	19186.52	11642.96	6279.26	12261.43	8588.246	22521.83	9697.29	25895.37	34416.85	18578.36	17243.2
20982.42	11979.9	17481.38	30921.81	13312.77	14855.97	16139.27	31244.49	26635.44	38500.33	16724.47	29991.23	10630.22
19641.89	10085.53	10381.42	7218.354	5284.132	8666.96	7277.02	25137.69	18913.25	29633.48	26607.88	17935.38	18059.89
16963.89	10786.09	18130.81	25882.71	18305.07	11705.86	24489.4	19526.12	10722.7	24880.04	21682.43	22300.12	13865.26
19420.12	7306.342	17733.35	20794.39	23550.88	19843.16	30804.86	27814.85	15258.35	34192.58	18710.28	15277.63	14153.83
18048.69	10141.56	18279.13	14971.49	16277.54	15708.32	20578.38	24391.98	15506.74	29019.27	21093.27	16757.03	15363.41
20024.27	9398.013	19236.71	32552.94	26563.75	19595.03	34534.56	29136.26	15267.75	36074.84	17993.33	10034.96	13850.73
22303.38	22729.11	21076.41	17118.78	13796.63	11746.83	20326.97	27755.29	69004.72	25318.01	40439.82	33956.46	22669.66
11513.58	9996.87	15906.06	16030.48	14896.83	15041.77	19813.15	24703.89	13135.64	30223.21	25583.7	22416.25	15642.2
19734.54	12793.14	17918.33	20389.59	9687.673	16027.37	11864.97	24235.22	17247.88	30176.97	21242.75	30355.06	15177.06
10167.39	9290.766	16763.79	16725.33	10451.88	8564.078	15804.64	18224.08	8844.576	22440.6	21448.45	17494.49	13235.49
19485.4	10200.88	13415.2	29659.38	14048.36	13989.06	17506.03	27815.23	23636.79	33928.19	16893.67	20889.9	10981.2
12850.19	11550.18	15207.24	11971.85	12244.51	18808.53	16578.54	27414.99	20787.91	33000.74	34989.06	30845.41	21065.88
18846.67	8575.56	8082.525	5122.511	9427.423	8259.906	11256.09	22010.43	9859.35	25786.3	21927.85	13565.9	15233.81
31169.89	15349.27	24099.96	30122.2	19555.68	25744.64	25660.37	39748.13	27818.16	50206.18	24472.9	21146.14	15403.72
18852.32	8323.221	17820.95	29045.6	20413.74	14022.19	26899.11	25699.48	15571.08	32577.39	15976.63	12013	11718.11
20724.9	11203.48	15412.26	26097.17	12816.71	15552.83	16640.04	29937.51	24401.68	36209.3	23329.6	17198.39	14403.18
11583.84	7598.555	8265.453	13085.67	7731.529	9053.663	10166.46	28688.59	18713.63	32438.42	20314.63	21221.36	10435.06
16117.77	11559.54	12022.51	5595.013	7257.556	8757.572	9174.359	21004.9	12607.9	24325.48	14558.02	12788.45	10276.25
10985.8	11309.97	14959.93	8827.634	10783.84	18033.88	14447.32	23778.75	19345.31	28344.04	35206.66	29935.5	21708.33
22637.83	13270.87	18737.35	26909.07	11161.67	16966.5	15305.36	31952.79	26846.03	38512.77	24908.39	20749.84	15002.02
16618.36	5348.709	6071.283	7816.488	10924.62	11376.65	15052.36	28297.67	17370.82	31940.87	16485.52	7732.581	9850.642

16618.36	5348.474	6071.249	7815.815	10924.62	11372.77	15052.36	28297.67	17370.82	31940.87	16485.52	7731.861	9850.642
16026.71	12740.23	11499.08	6444.948	4571.245	9756.623	6538.835	19693.52	6983.825	22224.54	27244.02	16111.18	14306.76
17172.44	9788.211	9496.011	37461.62	19333.89	16370.42	23824.69	26777.96	14651.39	31946.27	13812.3	22940.76	8257.467
28849.95	13483.08	21712.61	28420.39	16662.14	23900.03	21981.82	40663.62	29931.47	50572.42	20273.75	17187.61	12482.32
15259.67	8808.948	8500.28	5455.725	4478.727	2291.081	6175.362	16418.58	14108.55	17179.03	19113.63	15744.01	14267.72
11169.2	2173.069	1967.579	3283.205	7728.735	9714.041	10138.78	15917.13	8565.105	20419.23	17220.86	18307.12	12147.46
11169.2	2173.069	1967.579	3283.205	7728.735	9714.041	10138.78	15917.13	8565.105	20419.23	17220.86	18307.12	12147.46
15806.51	9974.439	11002.57	8570.222	8403.304	6991.834	10852.13	19801.08	15149.69	20001.03	19346.46	10766.52	9301.216
10802.39	13440.34	11743.77	7311.639	5343.157	7137.278	6773.462	10786.22	21327.36	9631.269	24408.59	18979.33	14826.48
14435.74	8812.779	16878.99	19936.88	15493.22	11980.41	20784.55	21876.78	13140.34	28280.41	15125.32	8017.666	11066.68
10897.35	14912.15	21554.43	21565.16	5241.262	6500.383	6844.509	16221.3	20535.46	19914.95	31112.91	26563.85	16932.95
25323.18	10296.57	21326.87	44153.64	21469.81	23359.73	27772.4	50093.32	31035	61998.13	27353.36	34058.18	16760.49
21745.23	11122.76	17804.09	30031	16507.4	12738.13	21256.42	24028.72	15205.37	31419.25	20280.61	20450.12	12453.46
13209.95	10526.7	11506.91	6013.242	3988.535	7088.885	4838.142	17303.26	9320.918	19600.36	20200.09	12214.75	10342.11
15724.13	8226.933	12348.15	8724.02	8754.06	10616.21	11062.44	22699	15370.58	27777.63	15172.98	15796.17	7943.801
4991.6	5427.74	9913.872	10608.29	10528.35	7729.131	14618.53	13571.09	6985.226	17597.03	15585.32	14130.36	9165.086
3208.027	973.3552	1816.011	3039.393	7072.931	3533.585	9786.996	5727.394	1893.005	6486.995	5611.256	6945.896	4210.742
11089.79	5569.065	11597.29	5859.157	8059.768	6826.647	11022.19	32549.06	15590.19	29073.35	11957.61	8556.551	8432.525
21379.9	5846.141	6140.398	11270.14	9590.57	16688.85	11663.38	31020.3	19124.5	36875.79	21813.24	14914.26	11893.18
24248.41	5719.526	6016.828	13555.37	11053.24	21899.82	13204.02	33530.04	19697.24	41749.56	21524.59	14657.41	11752.72
17622.63	10107.43	13728.18	18909.93	12020.41	12110.41	14511.86	28124.31	22253.78	32833.16	15895.03	17761.65	9183.642
21933.01	5254.788	5482.52	10994.12	9395.535	16799.91	11410.7	31039.66	18058.16	37382.24	20107.61	13478.7	10887.44
14692.51	8773.756	9928.277	25347.77	7983.167	6713.876	10183.01	17696.41	13002.18	18812.53	14251.84	9445.324	7424.536
12353.56	6948.56	13828.63	14876.78	14229.37	8073.761	19284.01	19502.34	11273	27276.49	10391.61	9710.506	7449.577
23690.03	5189.489	5435.725	12995.92	10373.75	19857.21	12366.07	33168.5	18667.61	41185.66	19760.52	13353.64	10754.71
20472.8	11524.76	18182.78	18770.84	15702.1	15228.53	18822.73	27584.24	22756.09	33975.53	21114.34	20219.24	12150.96
11234.69	6106.14	5614.896	32783.31	15699.66	11817.66	18553.89	20618.56	9854.821	24939.61	7770.963	15855.78	4660.393
9598.122	7193.646	11300.71	4470.136	6801.313	8235.381	8737.893	17998.13	10718.86	21686.3	14019.53	10320.47	7246.845
16197.13	15225.89	22541.54	26916	14459.13	20899.81	17644.36	25264.91	19837.94	29969.05	11593.77	12764.86	6716.888
7639.509	1866.999	1861.523	2624.539	7421.664	9039.627	9789.969	15712.84	7281.905	19260.14	6875.576	16575.48	4589.046
22053.18	8817.791	14620.62	25705.14	14973.17	11010.08	19421.5	22932.64	11625.49	31667.01	8365.322	12095.34	4845.04
16217.46	5203.336	7650.338	29505.89	15818.63	11447.24	19337.92	14751.31	7424.88	19934.32	10537.94	8687.603	7403.331
16217.46	5203.336	7650.338	29505.89	15818.63	11447.24	19337.92	14751.31	7424.88	19934.32	10537.94	8687.603	7403.331
23525.57	4605.287	4814.937	12538.29	9272.459	18022.13	11163.07	31801.58	16946.82	39033.1	17918.14	11848.42	9666.423
22984.33	4618.784	4815.607	12318.08	9587.868	18732.29	11401.34	32698.41	17451.3	40457.07	17813.91	11937.82	9660.869

10790.19	2739.712	2387.294	12449.92	7783.47	7630.956	9111.788	16533.27	8488.735	20553.4	5856.777	9481.856	3523.839
23102.37	4602.25	4796.272	12021.88	9478.257	18764.6	11241.13	32146.04	17412.2	39966.47	17795.89	11902.94	9643.087
9658.778	7992.961	5705.05	2871.718	2167.166	5104.881	3121.673	12883.07	4421.331	14599.99	14600.47	8625.912	7531.654
13431.26	5822.646	5326.889	31860.5	9544.507	10830.33	11640.3	21144.28	15098.74	28022.07	6925.159	14829.33	4790.125
11716.06	7371.377	10103.19	29537.97	8447.274	8542.225	11195.95	16057.15	12603.59	21700.26	11395.31	10539.24	6242.293
911.0482	301.1558	627.9116	44.80719	6212.162	6703.966	8876.223	6479.754	383.2488	8086.213	6398.411	6669.574	4798.538
6262.641	5523.2	11196.36	18104.42	7917.315	6001.626	10464.72	10553.29	6313.246	13617.8	6578.093	9347.165	4777.613
4153.45	5871.948	10646.79	5601.481	5156.998	4306.756	6935.382	14526.07	7105.931	17739.46	9589.722	7491.78	5269.8
10182.56	1898.897	2982.966	2487.601	5149.069	8234.315	6258.697	19290.52	10417.13	21385.28	7320.211	4580.008	4043.742
10321.57	8229.366	12529.21	14778.17	6833.518	10081.11	8307.627	13832.92	7283.388	16366.83	7711.95	12305.84	6612.532
11723.3	9031.361	12084.76	34718.05	8532.582	13476.26	10963.34	14543.21	9979.257	17968.39	4581.453	10905.03	3303.514
8232.693	10080.2	13579.77	20927.4	9552.501	11708.49	11670.54	22034.32	15956.71	26549.94	13548.51	12256.74	8121.585
13121.12	5546.1	13084.56	6856.343	7308.644	13195.34	8852.025	30701.54	18926.51	36823.02	14161.66	9615.425	6866.755
3828.57	6637.978	12986.89	6061.373	4027.05	6157.586	5360.048	12561.09	10088.89	15892.23	11974.48	7414.807	8827.404
14989.92	5760.135	5384.875	10246.97	8747.573	11732.2	12196.28	32132.4	19457.63	39531.34	12149.29	13242.48	2107.873
12101.87	744.1028	1226.548	3126.502	4717.862	6756.893	5265.087	16726.22	8793.472	19998.43	8819.402	8105.181	3853.316
9938.204	386.838	203.6716	0	604.7525	761.431	606.1861	3180.329	3593.378	4684.698	7921.097	7974.76	3833.196
7236.945	9677.133	15271.44	22696.29	8192.822	10339.76	10164.1	19882.62	14423.52	23642.18	13801.5	10475.69	8090.096
10503.41	730.3265	1215.3	2857.921	4662.992	6317.707	5223.256	11382.02	3214.595	15777.72	7398.078	5882.686	3530.014
9073.545	2567.141	3456.778	20049.98	2673.793	5359.272	2858.127	2908.809	6368.896	3884.033	7090.684	9063.878	3075.947
3078.792	5369.643	11653.29	11409.83	2996.197	5185.019	3990.826	9871.9	7591.895	12709.14	9102.809	5470.031	6941.424
8543.108	2811.498	2963.672	11622.35	5895.93	9374.444	6953.336	20970.62	17395.84	23925.15	13051.44	10618.69	8418.933
13233.48	4186.883	6675.741	3257.012	4665.642	5950.087	4696.814	19233.07	18263.51	23368.04	8007.554	4770.886	5786.592
4895.534	4933.525	7920.592	6825.413	4873.372	12609.64	6603.972	28828.6	17389.38	33042.13	6278.267	5484.478	3071.312
2611.843	12241.55	14780.16	3829.171	6360.075	5110.879	6785.499	25430.27	22801.02	29276.32	33551.66	21251.7	17354.63
9584.112	2030.527	1611.58	7519.279	6630.248	8353.651	7272.325	15308.43	7351.204	20289.31	2448.271	1515.836	1466.025
1004.024	736.273	1833.156	700.8644	8884.958	12358.77	11000.47	13242.67	602.0762	16732.86	6975.951	1209.13	4745.788
6526.884	942.8652	1560.195	1500.154	3108.335	5637.963	3701.865	15680.89	8350.028	16986.49	3741.279	2343.965	2050.123
4410.352	1760.014	1682.624	10041.79	3316.398	4344.977	4092.647	7893.636	3048.922	9024.221	2852.677	10937.73	1809.915
15976.6	4034.24	5837.682	21367.13	12660.12	12034.81	15424.06	16018.59	7586.223	19583.37	3127.44	6003.006	1554.68
13210.99	3461.99	2328.255	18317.31	4833.009	7490.58	6117.506	18473.65	8318.191	22755.21	4936.971	10138.23	2564.757
7280.224	1761.7	2542.765	1605.453	5002.216	7526.68	6172.247	11556.42	8879.937	13170.22	7269.665	6500.92	4499.646
4737.828	5862.712	1520.276	6195.166	2865.398	4100.125	4283.155	12265.31	2821.811	13948.72	3491.813	7963.075	2987.038
12524.98	892.8695	1291.503	2417.857	3194.192	2896.398	4374.128	11201.15	5663.52	13087.4	4481.871	2387.706	3448.412
16644.4	1734.035	3683.159	3673.611	11168.52	14728.8	13034.25	30986.25	13544.27	40320.34	5403.895	4410.501	3926.642

9720.531	2921.732	2355.092	17927.34	4159.077	5503.679	5277.185	11157.79	5659.462	13932.4	3203.204	6756.261	1790.872
2491.827	2388.376	5182.218	4553.996	3453.299	2593.677	4453.905	11213.51	8870.495	13119.8	5350.752	3917.209	2961.476
10045.82	4491.837	6054.263	12418.55	5628.791	6737.99	6894.61	8893.007	8208.344	12075.41	3471.889	9338.368	2469.439
4532.493	3788.994	8585.778	5025.039	3268.923	5396.387	5152.416	71.86118	178.2206	152.5889	4183.382	1283.921	3791.632
3950.426	5919.055	12308.59	12263.09	4758.029	6884.943	5390.426	11633.71	9080.028	14889.28	10374.44	6181.985	7680.97
13730.93	2625.355	3082.588	26414.89	6514.398	9708.492	7496.663	22316.37	14105.6	27703.84	3399.782	2993.471	1169.507
588.3252	333.8321	811.1001	2513.158	2908.845	5361.377	4281.265	4318.949	720.9348	6495.979	1155.268	383.3094	1132.395
12332.1	2844.807	2315.276	19185.39	4851.086	7614.833	6183.019	24930.77	16492.89	30355.71	2930.559	3467.297	738.4552
2824.775	1961.333	3889.672	1848.196	2859.196	2980.571	3916.802	5732.036	3783.346	7899.327	2451.678	2290.197	2271.183
5921.724	869.5732	923.6004	16772.06	3800.859	3151.027	5245.804	8724.574	4858.347	10539.03	639.86	723.6092	127.1143
3584.494	5413.891	11720.11	10481.06	4800.943	7827.978	5858.444	10234.01	7291.898	12839.89	9157.645	5508.126	7007.924
12732.58	2523.018	4083.092	10214.31	7173.398	10481.25	8747.664	24405.38	15640.03	29815.79	6837.884	7117.621	3070.361
12103.39	4393.422	2576.695	12460	6186.586	8790.875	8031.218	18169.05	11496.2	23527.66	3488.659	5353.895	2894.575
3091.744	1421.909	2137.895	2702.072	2602.009	4422.613	3288.402	10020.35	6201.301	14003.61	4062.253	1646.872	2890.966
2547.148	4777.719	11559.92	5877.208	2694.726	8654.102	3727.476	28381.12	17243.21	33987.91	3220.774	2890.817	1565.483
2456.027	2378.375	3616.616	5455.796	2708.433	8112.27	3654.24	18908.85	10306.5	20690.67	3460.017	2987.605	1674.981
621.7413	375.9042	744.3161	441.4427	9848.785	15409.16	11875.36	7431.157	378.0861	9317.107	3088.436	501.8796	2300.387
3201.815	1937.275	2113.833	1136.249	3608.206	4239.169	4748.283	7912.855	4967.196	9533.301	4729.054	4346.862	2470.514
12714.64	2219.645	3415.178	8686.295	10459.65	9284.269	12165.13	22495.93	11020.72	27339.61	14020.36	1999.182	9110.889
444.1525	251.4884	612.5877	1912.131	2271.904	4299.946	3376.291	3442.599	551.9469	5201.623	883.4017	289.7494	874.192
14242.66	1968.222	2101.091	2686.975	9024.58	11480.9	11017.54	22403.3	12467.76	25921.37	5231.461	2064.921	4157.803
8234.077	1023.021	1000.872	8894.293	5783.496	6959.693	6785.176	8881.907	4624.096	12308.55	754.2617	6719.668	468.1397
8211.284	1024.484	998.7601	8179.779	5621.057	7240.272	6517.404	8280.635	4294.625	11193.72	767.4915	6673.86	573.0315
1533.558	2018.329	1291.375	2430.472	1457.858	534.5011	2073.124	2492.705	1093.495	2489.521	1362.488	6007.637	1645.105
15793.47	3199.987	4663.02	7095.104	9318.012	10826.53	10620.65	20638.94	12304.59	25666.51	5966.108	5664.926	2288.758
11636.43	1582.807	1797.377	1602.841	6929.368	10189.54	8159.13	17393.65	10220.81	19226.66	4673.145	4683.29	3707.976
6008.707	939.253	586.1852	15214.14	7369.278	5663.9	9259.296	1272.776	457.166	1592.347	1571.162	2663.587	1614.408
4255.747	752.334	1407.426	459.8683	2408.886	2985.13	2724.411	6417.76	2926.168	7462.387	602.2981	4018.325	534.1836
8077.018	2168.464	2684.733	3180.683	4854.023	9649.693	5556.848	24710.31	14338.8	29446.85	3873.332	2183.547	1599.882
2577.711	1135.79	1089.876	1109.712	2289.528	3722.529	2134.221	3825.47	1659.615	4636.099	779.8986	454.8153	421.4619
4539.716	329.9428	1405.619	896.534	2980.495	4686.679	3724.906	8119.048	6477.376	8989.614	2121.464	1843.221	1753.784
7251.674	1033.284	1013.188	12334.74	3478.18	3901.36	4609.484	9123.494	4544.384	11318.3	720.232	924.9701	170.0277
11349.49	5555.06	3764.896	3427.619	4970.561	6367.712	5918.738	18743.55	10992.79	22365.09	5549.787	12336.22	2677.049
15629.12	2952.001	2919.259	25943.33	6036.842	10732.1	6927.927	16521.77	5680.823	21111.24	1679.145	3566.236	451.7151
6294.911	2102.507	1655.672	3500.001	2618.8	4368.803	2680.293	9108.793	5710.171	10860.55	1077.598	3936.574	306.4078

8591.872	5284.091	3755.652	3491.256	4192.986	5113.16	4998.36	14179.49	8044.669	16505.91	5373.276	11662.14	2632.756
10855.18	5148.288	3448.249	3179.779	4570.972	5942.166	5443.686	17769.16	10241.87	21245.89	4943.174	11470.68	2380.264
10855.18	5148.288	3448.249	3179.779	4570.972	5942.166	5443.686	17769.16	10241.87	21245.89	4943.174	11470.68	2380.264
10855.18	5148.288	3448.249	3179.779	4570.972	5942.166	5443.686	17769.16	10241.87	21245.89	4943.174	11470.68	2380.264
643.4586	4305.373	7594.234	10087.52	16.01071	32.87965	7.005946	2888.813	265.5074	5841.792	132.2894	264.3248	84.99483
15595.7	409.5117	686.6426	153.6961	8092.62	9693.203	10082.9	5811.246	934.2023	9720.247	138.0695	661.1477	109.7039
12070.66	2421.426	2467.053	10603.67	5604.059	8825.434	6405.515	13442.2	5511.061	17569.55	1404.064	3056.524	377.4167
5245.084	1387.345	1398.068	2076.618	3909.714	4852.356	4934.718	8322.059	4952.531	10942.89	1694.01	3111.731	754.0377
8591.872	755.4786	1690.759	1517.156	3924.217	5113.16	4186.473	14179.49	8044.669	16505.91	2682.506	10515.94	1716.567
9474.38	1774.776	2127.492	5665.811	4942.024	8108.266	6183.161	20468.1	13806.07	25395.36	3276.468	3700.557	1233.111
494.4665	963.1797	852.6815	1586.22	581.4117	193.1532	763.6386	184.9496	662.7618	221.6719	494.1705	1200.497	544.7624
545.7106	288.5566	718.7876	444.8474	276.4781	4764.226	335.0262	12748.5	433.5619	16866.75	1699.546	129.881	1139.957
895.0491	97.62348	0	1630.645	1399.891	392.9192	1661.331	889.8152	639.2029	1173.812	410.1289	588.6384	423.3128
9812.53	4159.953	2638.159	2412.477	3615.902	4761.369	4265.919	15077.17	8548.012	18232.84	3849.419	9076.919	1845.664
14012.38	2709.906	3191.734	7521.062	10735.75	12530.9	13814.93	18525.07	15372.77	25634.93	3749.525	3680.214	903.0226
8051.721	2836.248	3019.231	6529.958	10765.85	9591.557	14499.52	10099.18	7216.588	12218.19	2357.235	8670.782	1884.588
8297.828	843.4973	1650.977	2525.939	3640.801	5678.547	4559.378	18371.05	9041.998	22563.98	2742.063	2895.743	1011.592
6928.585	3933.381	2631.351	2459.813	3007.341	3733.839	3557.679	10859.59	5988.308	12713.94	3722.202	8532.587	1814.102
13503.86	2624.592	2680.775	4253.428	5791.63	7425.548	6401.869	16357.15	10257.41	20478.65	3546.727	3472.388	1244.533
0	3264.255	2205.054	146.2653	723.5776	454.407	909.4951	4196.495	0	4114.351	2181.483	1097.61	1653.599
737.4388	759.2243	888.3617	26725.71	0	0	0	0	0	0	141.1178	62.57131	110.6763
14838.67	2262.193	2665.223	6318.979	10904.2	13299.58	13124.62	22272.93	10031.97	29557.41	3071.627	2938.748	735.5238
4055.175	2000.173	1628.117	3405.374	1842.505	4052.35	2050.986	7752.738	5062.277	9671.597	920.8962	3496.224	282.5147
33.9839	794.5047	1296.085	1782.816	197.066	388.543	244.9779	16755.73	11023.11	19363.92	2103.379	1133.879	320.9016
7197.57	681.4112	1402.987	3673.611	3360.309	5780.65	4181.897	18304.04	8408.196	23046.91	2224.247	2534.295	822.2853
9563.353	1214.493	2034.069	3064.685	5332.147	7809.569	6985.965	16666.02	10105.5	20199.56	2598.451	3984.582	1135.281
8508.461	371.6469	860.3339	338.3621	4342.59	5428.432	5734.411	3208.799	267.4883	4489.218	445.0735	767.2111	412.1081
576.6844	2668.611	1598.787	0	32.20179	1002.709	63.84865	2595.103	8370.039	2071.157	4292.383	5166.5	2557.012
9626.062	1227.189	1236.525	4939.523	4061.207	7754.419	4580.292	13347.01	5951.865	17553.28	965.9655	1390.778	287.8579
5054.865	1048.817	1201.857	3021.884	2380.064	5080.759	2814.813	9482.418	6060.017	12450.8	1327.46	1267.493	415.4652
2220.103	465.1086	1134.468	1449.723	2030.481	1635.953	1390.539	9998.446	5125.249	13259.72	1611.27	1254.51	382.7285
1578.462	371.9592	791.8856	3018.517	1155.367	1248.138	1352.508	1384.071	3317.839	2275.393	408.0942	469.2339	417.0927
1477.222	383.4516	836.0337	5455.796	1139.067	1251.495	1344.243	1398.143	3489.531	2314.161	404.4285	465.6127	409.4806
10534.07	1320.414	1662.575	4313.329	4271.227	4656.204	5082.836	23081.39	10448.05	28248.95	2126.488	1927.043	857.621
1079.126	1026.616	2396.764	1269.947	3549.42	202.6085	4620.557	7161.284	4784.644	7636.455	5434.638	7286.139	3799.827

3204.107	2227.669	1537.837	2404.517	2408.74	2224.801	4662.404	18785.17	10276.45	22000.55	1829.543	3322.044	215.0567
839.4605	436.3285	849.6709	21266.46	0	4297.45	0	369.6709	87.34988	446.8196	0	173.187	63.28474
7814.33	434.8497	386.1687	1070.359	2535.078	5616.238	3210.554	7544.208	3617.872	8713.253	421.5818	940.7056	101.9376
10826.81	1234.475	1151.535	3339.482	5064.76	8185.693	6233.422	13565	6578.314	16500.83	1314.191	1784.621	383.3047
4767.844	1453.413	1376.261	1305.332	2376.323	2048.473	2842.239	8874.016	5166.676	10423.87	1829.144	1538.617	710.4856
10566.49	1175.236	1092.074	3167.815	4850.598	8045.669	5965.144	13285.72	6419.81	16071.72	1252.273	1699.005	363.6295
11636.43	1396.699	1235.967	1602.841	6292.154	10189.54	7287.016	17393.65	10220.81	19226.66	2673.736	1238.072	580.7649
8591.872	668.3943	1690.759	1517.156	3924.217	5113.16	4186.473	14179.49	8044.669	16505.91	2682.506	10515.94	814.2857
8591.872	668.3943	1690.759	1517.156	3924.217	5113.16	4186.473	14179.49	8044.669	16505.91	2682.506	10515.94	814.2857
3182.812	521.7179	0	112.2693	3527.688	2426.988	4778.414	782.9192	0	1379.651	399.475	120.3871	397.5384
5327.194	3583.875	4628.503	1500.416	3049.571	5241.361	3693.166	23368.75	10687.98	25001.15	1619.452	1487.057	572.6842
5327.194	3583.875	4628.503	1500.416	3049.571	5241.361	3693.166	23368.75	10687.98	25001.15	1619.452	1487.057	572.6842
669.4568	374.4639	517.136	219.8428	1133.948	1786.884	1240.713	10036.15	4401.527	11866.65	1263.535	1728.637	859.75
0	190.9647	270.9529	558.0348	2193.307	2657.788	2645.626	7317.874	0	6991.135	452.8821	394.1321	147.1596
4551.778	116.7451	138.7478	296.8251	2051.077	4709.103	2385.206	2928.117	908.7024	3909.691	169.533	196.0739	87.17321
6282.764	2197.543	1294.61	1162.732	1859.372	2553.502	2179.725	9094.301	4854.749	11187.36	1765.364	4661.217	839.7005
4086.788	523.9952	198.65	605.3345	2129.871	3884.134	2554.176	1919.962	1145.368	2797.143	619.2513	1479.398	459.441
11154.74	812.9109	1339.213	3408.569	5077.536	6826.157	5683.666	15370.92	7920.208	18321.01	1874.017	1462.64	383.6489
337.7104	2496.716	4005.285	1814.69	61.25888	444.6007	44.94534	1592.651	7653.806	1850.142	618.4244	1264.833	471.7359
4764.647	3058.943	1023.092	6066.498	1636.66	2294.361	2672.597	6700.688	4400.016	8275.282	565.9264	8703.069	428.7757
1712.225	1313.054	850.3002	1298.463	1326.607	1172.048	2735.33	13111.34	6887.766	14929.9	941.9693	1909.487	107.7033
6340.248	709.818	1004.673	1857.443	3232.423	6967.12	3574.649	16314.57	7443.142	19662.44	1220.764	897.2955	348.8474
9872.439	1020.141	999.1986	2701.583	4959.137	7489.697	6263.775	11182	6605.82	14392.32	1083.97	1857.223	267.8482
16852.91	1145.719	1291.551	3212.103	7962.574	10738.21	9047.747	20202.96	10097.85	25952.58	1472.142	1452.205	338.1761
8567.992	668.6826	831.1751	1551.576	4242.288	6974.894	4869.385	13562.41	6735.874	16861.45	1375.608	895.9642	302.8471
6928.585	451.5595	1152.326	1035.645	2800.954	3733.839	2947.413	10859.59	5988.308	12713.94	1822.564	7620.97	548.3668
4250.701	748.568	1245.55	1103.021	2139.416	4330.338	2697.284	9114.456	8059.302	10041.28	804.2619	857.3154	401.1393
8106.239	730.9223	998.034	2233.366	3983.885	4750.817	4313.4	22733.27	9557.918	27113.1	1269.503	1296.873	238.4318
4415.875	1517.714	932.4366	4953.597	2341.507	2621.469	1919.347	9571.298	5425.27	11933.6	829.9023	2143.279	265.5265
8595.882	583.9833	723.2564	1427.397	4155.045	6731.456	4714.856	12445.92	6184.483	15502.23	1219.233	798.1462	257.7415
11557.53	908.1866	718.7427	1605.177	5226.786	8147.774	6591.915	13300.1	6474.715	17172.88	1285.931	2261.786	387.3957
11077.55	900.3811	717.3994	1615.058	5260.707	7790.808	6638.777	12753.55	6169.091	16686.29	1264.124	2225.47	383.991
4917.943	535.139	638.1992	1455.381	1842.505	4052.35	2050.986	7752.738	4682.655	9671.597	683.9752	707.437	145.2164
19.87333	56.15838	80.22298	85.75102	139.9945	1447.717	152.9289	56.94212	15.54408	77.06707	201.4199	51.94957	190.403
9.726826	260.6535	447.5281	577.4254	57.2132	114.6864	71.18207	9214.68	5699.919	10116.38	776.9733	369.3684	102.8952

3432.219	249.1448	501.0702	708.9735	1404.443	4584.986	1467.934	8730.418	3573.744	11339.53	795.2987	368.7018	250.8128
0	477.624	777.7862	7.993399	1743.847	1394.905	1922.641	504.8102	1358.104	396.7022	238.9052	977.7718	223.2045
6697.847	648.2779	596.6786	1209.144	3137.328	5380.326	3579.487	11541.04	6191.487	12719.88	1701.382	601.4863	534.975
10471.17	798.2478	631.3814	1419.936	4757.174	7440.234	5996.892	12071.01	5791.933	15683.12	1127.727	1991.789	339.3994
3366.88	2126.418	689.3094	4304.077	1109.894	1577.113	1819.236	4714.68	3055.291	5826.963	378.4311	6126.755	286.8912
3425.966	492.6411	575.1642	1585.469	1413.287	1934.808	1748.028	6005.193	3961.497	8210.748	790.2786	616.0531	216.6763
3998.017	1343.969	748.5719	4827.504	1827.738	2088.546	1443.075	10373.83	5249.228	13552.07	679.4914	1693.131	199.8568
1922.562	695.491	807.3929	1326.925	988.8626	4430.132	1006.657	11562.72	5198.844	14954.47	834.6036	781.9981	176.3068
2773.549	157.8975	138.1792	38.09425	897.8453	2259.101	1081.961	2736.968	1312.757	2952.692	894.3554	499.3531	501.9521
102.6061	164.9506	256.248	196.5265	109.4786	344.2698	128.0004	2882.664	85.59987	4299.235	534.0897	33.55781	443.3184
4872.903	1091.284	887.2031	802.4356	1493.06	2559.367	1806.303	5872.182	1368.886	7586.394	709.8913	912.8345	319.3074
77.00015	152.9195	394.6965	436.9034	706.1259	271.6987	585.8895	152.9385	20.70353	222.3345	442.9707	70.71374	97.80408
6341.718	487.9026	794.0213	1400.887	2230.01	5127.453	2470.401	11233.47	5008.939	14865.97	2539.171	2693.237	713.1338
0	0	137.0829	0	719.3115	68.92892	770.8971	182.2053	0	262.553	572.986	685.7938	360.8327
5442.108	541.4433	589.7608	1364.29	1971.908	3905.495	2118.98	8155.723	3917.94	10821.97	580.2914	686.8603	175.6649
154.2445	659.6329	848.1646	1425.772	2054.175	4876.686	2214.729	12333.17	6192.898	16307.57	940.3278	577.3361	202.7794
4654.68	255.2608	449.6468	1742.714	2162.796	3318.816	2501.983	8707.926	3555.131	10804.1	647.1456	407.9839	140.575
3639.16	341.5445	398.796	1252.312	1662.12	4245.202	2092.995	7341.197	3335.941	10069.09	749.1945	450.2763	264.2944
109.5197	20.477	0	78.92794	68.98475	177.0757	259.2224	0	0	0	0	91.08435	0
109.5197	20.477	0	78.92794	68.98475	177.0757	259.2224	0	0	0	0	91.08435	0
109.5197	20.477	0	78.92794	68.98475	177.0757	259.2224	0	0	0	0	91.08435	0
586.498	204.0705	347.0792	1515.964	166.6788	294.5049	242.8463	509.8675	0	826.8126	74.48543	198.8901	139.3213
8785.653	378.5575	536.8918	1100.723	3880.832	4740.283	4787.254	12147.32	5833.603	11949.25	899.6706	781.1277	293.1226
992.9767	1145.015	1629.348	1501.798	573.6127	1339.503	643.074	4030.039	2052.496	4821.687	1126.673	2525.049	561.2164
0	517.3715	369.4595	691.6077	173.4387	45.70944	142.5347	8467.578	4565.904	9691.927	529.4395	452.8925	164.6381
91.95081	587.8428	827.2361	891.7483	131.581	236.3781	44.31539	6921.491	3423.314	8797.016	583.5618	431.8697	172.179
1626.237	498.0346	980.3942	770.6186	1779.711	3309.753	1957.704	13024.94	6727.016	14715.42	785.9186	546.3155	188.3027
6970.338	464.2271	364.1763	821.9457	2903.824	4849.67	3653.299	7764.771	3592.446	10150.15	650.1396	1159.689	194.7566
51.98588	21.43403	0	21.72662	18.2311	88.19796	24.37241	0	0	0	0	361.2807	12.6829
102.9434	0	0	0	82.01057	37.00752	102.431	3290.566	105.7055	4685.665	0	0	0
0	540.4245	481.3349	13.80333	1034.487	1838.547	1160.871	834.8229	1858.115	667.6317	322.8276	807.4445	193.2349
93.52568	37.3946	54.32094	5734.925	0	0	0	0	0	0	25.28287	0	24.62929
4957.363	465.822	669.3899	955.2418	1863.529	2961.405	2154.675	7402.399	4465.947	9268.989	630.2991	711.2673	143.1704
9544.133	432.0582	622.1565	816.2083	6097.694	8708.161	7223.594	9497.195	4326.883	11181.71	574.7476	817.5655	191.519
806.8148	898.3602	306.938	2642.211	485.1683	1176.336	651.061	957.3787	973.9005	1445.215	37.1605	2114.781	77.5566

0	1233.634	847.7624	2068.33	645.7943	302.9182	1044.008	423.7916	185.5127	462.022	1933.467	2600.028	1303.776
184.4019	278.205	501.0704	681.3881	219.0289	1431.989	132.6777	8254.186	3421.782	10377.97	747.2152	415.1472	101.8686
1417.803	271.1977	521.4174	1484.994	720.4159	3214.906	838.9546	8969.752	3278.088	11000.3	474.4143	473.5981	95.0559
3882.921	402.7057	369.9679	28.83242	2050.932	2887.208	2300.621	1158.542	1697.903	1640.224	290.8377	539.2999	146.2871
81.78825	0	392.1169	3154.382	123.3234	2629.62	322.6142	1144.494	309.4663	1133.693	745.9264	2417.142	81.5028
371.2719	451.032	233.9364	339.7134	309.1565	248.8826	727.9065	4506.593	2247.267	5100.547	242.1479	624.1792	25.01986
0	18.41364	237.7233	12.63622	66.25482	374.747	126.3192	682.9374	1346.108	600.4035	110.5121	588.1933	114.148
11230.43	72.90287	245.8659	198.414	3557.373	4969.754	4166.863	189.4292	16.21667	409.3739	175.5911	804.6019	31.29315
2855.767	131.2356	322.8998	697.4007	1457.028	2327.126	1792.368	6301.987	4121.267	6547.755	665.4814	490.825	150.1079
85.06178	0	0	1042.513	0	0	0	0	0	0	42.82354	0	0
8055.256	671.1109	647.1542	1092.806	2753.888	7505.351	3175.977	11839.67	5855.256	12274.02	715.0681	789.2681	120.5768
402.6571	143.369	236.8548	264.0306	198.407	2640.39	235.8343	6401.676	325.5478	8144.895	447.0278	87.48828	106.7952
2852.564	185.3367	263.1939	633.478	1015.578	2683.855	1069.146	4972.921	2226.124	6505.005	286.1391	269.6379	25.83723
5463.243	1431.757	256.6821	5245.58	1293.871	8069.113	1614.594	4254.86	2365.887	4416.171	251.8656	1870.587	41.64679
0	38.40567	0	0	0	96.92849	0	0	0	0	42.30951	438.9665	64.5316
2702.302	105.7457	148.1111	305.9272	823.6733	1414.944	1099.997	3326.592	1797.778	4990.594	302.4965	157.1457	17.97548
4114.358	451.458	421.7138	534.6819	2425.186	3680.06	3011.008	2582.79	2269.422	3538.419	495.3413	524.264	155.8411
2548.798	675.7534	300.2355	755.0356	1305.484	1684.712	1343.898	4140.795	1696.299	5731.074	316.8956	1953.761	102.805
49.10142	9.199751	0	35.51987	30.94213	80.71459	118.3279	0	0	0	0	42.25708	0
183.7627	15583.87	30172.74	31301.35	39.14856	257.4219	90.84736	2332.023	14.13212	4445.364	1771.328	2654.093	1442.372
0	194.9582	79.54831	221.2492	62.4344	0	74.50681	0	0	0	77.61182	12.09702	212.2483
3687.979	224.2891	332.5185	588.6224	1932.719	2310.166	2420.833	176.7246	412.1111	371.1407	470.9823	374.3977	155.1648
4376.572	706.2842	291.0419	375.2193	1031.014	3991.575	1104.173	5539.098	1046.371	6276.426	161.4546	533.5597	12.61794
191.1682	219.6045	304.0017	356.9714	191.0716	863.0812	302.1046	4928.824	2577.088	5820.892	311.1115	193.4804	90.29181
0	172.6156	201.5682	477.3234	0	0	0	5890.858	2027.735	6629.587	294.021	377.2152	63.18709
2699.35	61.86862	193.5821	640.7098	1016.691	1784.7	1274.988	4648.705	3114.786	4884.972	369.6219	331.6053	76.98527
2699.35	61.86862	193.5821	640.7098	1016.691	1784.7	1274.988	4648.705	3114.786	4884.972	369.6219	331.6053	76.98527
0	83.78357	143.9019	0	11.89769	5.057642	4.313252	110.9236	0	218.6724	32.44421	83.63185	34.63112
0	0	80.25139	30.96382	48.73597	2439.351	151.4023	3251.015	0	7050.753	109.3754	103.8405	126.9868
0	63.51491	55.52712	161.4687	0	0	0	730.9852	0	1700.171	13.48912	51.79514	9.405312
423.7061	206.2409	260.7002	275.1628	53.08426	86.81386	4.87751	5605.549	4305.372	6898.454	597.9512	302.0549	133.8222
6328.472	180.5174	266.6836	503.3944	1807.074	4679.954	2171.84	5668.779	3101.251	6623	362.226	193.3642	78.05992
358.1265	207.8428	259.8656	250.3232	43.85967	2909.238	3.994179	5340.859	4585.813	6578.765	646.6829	302.3547	147.098
125.0676	0	53.38963	270.7407	125.3207	863.1427	163.3724	137.9747	341.7787	147.8534	0	145.6429	0
249.5622	96.50208	129.2267	214.8493	53.60507	41.97831	38.37505	3824.778	272.3857	4462.707	225.3001	69.45115	24.80852

416.0485	4700.42	4992.012	86.3266	115.9224	1104.637	66.97899	2779.662	215.3751	3704.759	853.418	1878.414	547.9446
0	489.0682	329.8651	890.5561	248.5427	115.0141	405.0916	160.1893	69.89407	174.5754	746.6643	1028.185	507.1961
0	37.56606	29.14476	119.091	0	0	0	373.3153	0	946.4098	6.345158	26.93608	4.939533
2772.263	136.6288	201.8537	355.7306	1297.19	1563.292	1606.112	106.7721	250.6839	224.917	286.0929	226.6834	93.71127
5351.088	107.6592	96.93444	86.07849	2263.072	5398.029	2808.38	683.567	12.41269	1062.425	47.63334	319.8351	42.93103
0	54.94397	45.98906	169.0171	0	0	0	481.6683	0	1025.114	10.19334	43.22318	7.73716
0	143.9189	164.533	380.3403	60.85185	42.82584	48.42321	2825.658	1464.923	2955.554	180.9588	160.636	42.48424
3456.967	95.78153	118.5823	279.9004	1055.198	2014.999	1329.852	3368.542	830.9053	4212.343	141.362	229.8728	19.81601
0	152.3337	148.3516	311.0405	0	0	0	5255.541	3158.203	4513.28	311.1991	179.2682	70.56722
107.6454	67.45937	111.5695	299.1392	48.99025	1694.561	32.4424	1641.911	547.8847	2214.714	72.57914	48.56232	16.31695
0	41.80273	35.17995	115.6329	0	0	0	467.5253	0	1125.233	8.343963	33.19141	5.881681
9513.55	1536.72	1134.482	1680.636	3287.256	3710.262	3696.078	17245.28	10550.73	19131.79	2825.715	1730.533	584.4223
50.31107	273.3345	51.0696	15.89999	0	0	22.7551	56.78904	0	0	57.19425	236.6998	55.05547
2940.681	94.91486	88.90671	210.5257	1460.865	1821.216	1812.374	2698.916	810.7348	3075.09	194.089	195.4679	50.89207
4091.029	69.28868	76.57301	76.53358	1621.763	4115.546	1950.222	590.0982	12.35808	926.6203	41.54177	228.9865	36.29136
0	0	48.30205	42.57527	0	0	0	1986.314	1476.901	1426.109	151.8939	101.3702	23.30742
0	0	99.27628	36.15486	25.9907	772.697	48.56218	109.7141	0	98.95068	61.80945	51.52445	106.577
0	0	99.29902	35.91051	26.11651	875.3533	48.89458	110.1166	0	99.12362	61.74638	51.8895	105.2706
0	9382.956	19682.78	20596.83	17.00158	108.8804	46.68625	1877.48	0	3529.58	1294.004	1989.319	1093.372
110.2279	114.2873	74.53012	61.23468	191.0716	267.0494	302.1046	1227.83	365.6138	1803.971	51.01012	80.72732	8.76314
0	0	0	5.633487	0	0	0	0	0	0	87.9718	0	0
0	0	0	0	38.66023	0	70.08499	20.13058	59.81314	53.39335	206.0719	140.1761	104.946
0	52.85299	75.63919	262.1749	18.83939	16.12048	7.794289	1340.851	549.7719	1514.695	72.24252	56.1553	9.23873
9182.393	1518.755	828.7657	1256.234	2612.837	3141.051	2766.076	16751.44	10082.82	18627	2124.643	1645.87	371.1195
0	40.75381	61.89904	221.4725	22.73954	15.75935	26.5717	811.9042	388.8743	1173.965	58.81901	45.10184	7.482122
0	40.75381	61.89904	221.4725	22.73954	15.75935	26.5717	811.9042	388.8743	1173.965	58.81901	45.10184	7.482122
73.72832	0	17.58284	0	34.75051	599.9153	49.067	67.90434	0	75.99951	26.54187	28.86587	30.49636
0	0	0	0	73.93765	0	101.9718	23.08629	0	71.77299	0	62.20336	0
39.95208	35.5182	8.668414	65.82553	0	0	0	1215.796	0	1604.747	9.874871	88.32157	9.377052
386.9168	206.221	24.22902	331.7322	0	0	0	0	0	0	0	104.5238	0
5397.612	450.6737	385.7004	732.3877	1591.089	2075.926	1699.179	8869.923	4265.776	10294.92	769.4484	629.3751	138.2655
3922.632	83.6911	116.2701	326.9343	1632.92	2593.072	1958.396	602.4629	0	1021.824	0	42.58827	0
3922.632	83.6911	116.2701	326.9343	1632.92	2593.072	1958.396	602.4629	0	1021.824	0	42.58827	0
1146.384	56.77999	103.3252	67.84464	534.5379	759.1905	509.7849	342.4648	0	772.0151	12.2077	55.13986	0
0	0	0	3.756559	0	0	0	0	0	0	59.28397	0	0

144.3324	59.0312	86.51467	73.1851	0	0	0	2122.297	628.6759	2099.503	88.83511	55.32225	14.90942
0	57.66313	37.40986	60.09505	83.29356	24.12521	24.54543	968.3727	562.2835	1602.522	48.85692	104.7843	13.13523
69.53369	0	34.13917	0	33.28236	21.43576	19.81306	2070.5	891.9962	2227.033	112.1167	62.4189	20.36474
0	55.74355	35.26513	64.05029	93.29645	2095.439	23.36292	875.9453	510.2849	1481.061	43.81634	102.4146	11.87176
2729.234	0	0	0	1020.051	3623.091	1240.662	37.54668	0	108.6388	0	89.12582	0
0	0	0	0	14.66799	66.54092	0	164.5909	0	203.3856	54.60371	28.02247	46.14973
657.2993	15.27612	33.92593	85.87672	273.374	1030.821	296.2017	192.3228	0	347.1147	52.70891	11.62659	27.24159
4273.853	94.42368	36.03195	87.51534	1732.233	3057.82	2167.776	1940.102	982.2748	3739.702	24.52249	187.065	15.71694
0	30.59798	72.54446	0	10.65407	0	30.84644	37.4697	0	45.30523	0	0	0
0	32.75854	20.36661	37.38589	53.86383	1383.458	13.3804	513.7296	299.4692	880.6418	25.25924	60.40231	6.831342
102.2654	31.17348	45.70021	80.87626	0	0	3.919214	572.3227	84.3584	866.9859	49.79092	16.87139	8.609071
39.37892	4.419984	141.8618	0	39.04927	799.1177	82.13666	288.8081	218.7368	473.3894	0	86.90887	0
70.96641	34.15128	31.92248	55.87088	399.5819	517.2143	524.6986	2830.278	1164.351	3392.672	82.83591	90.52869	5.060905
82.90524	195.2666	65.95707	654.5255	106.8748	1430.261	145.8182	141.4516	126.6491	230.4683	24.82033	497.1431	27.92227
0	54.20572	37.84889	23.69127	80.65909	29.65738	18.706	1630.146	520.9953	2053.333	24.47317	76.21593	16.08879
0	0	0	0	0	0	0	0	0	0	0	0	0
0	49.10536	33.899	21.13223	71.97155	26.38053	16.64021	1471.574	468.2796	1858.165	21.79784	68.69311	14.45083
0	0	0	0	0	0	0	0	0	0	0	0	0
0	29.24698	39.31963	44.10321	0	0	0	141.5084	0	321.3537	2.582703	3.537448	3.392021
70.85427	30.29688	31.8698	55.24576	392.6317	515.5481	516.2385	2752.33	1146.691	3263.934	83.28773	91.13143	5.074918
864.3442	70.57753	27.23504	16.56298	610.7229	3003.737	745.5835	1441.196	482.8496	1815.445	16.04396	79.82071	14.54814
73.72832	0	0	0	0	0	0	0	0	14.77107	0	22.39888	0
0	0	0	0	0	0	0	0	0	0	0	0	0
286.0689	644.8339	78.80271	685.7999	111.4473	579.2787	112.9886	105.9631	155.6225	114.1037	0	973.8805	0
2664.006	198.182	378.723	797.6699	1580.877	2447.93	1877.514	6636.4	4337.084	0	0	494.1694	166.2655
201.4361	461.2794	55.35029	514.3348	79.30109	416.3636	79.99277	74.36841	110.5439	80.07368	0	706.7564	0
58.11721	168.1215	403.4347	2081.975	461.3462	353.8123	762.2753	339.3281	1064.717	407.3706	68.36209	1277.98	0
0	0	0	0	0	0	0	0	0	0	0	74.11502	0
404.7491	122.1652	30.0536	19.57678	54.88684	1874.372	40.51028	0	0	0	0	86.42831	0
3314.735	81.8381	103.7141	134.4717	1270.782	2502.212	1465.202	672.4995	0	1375.318	5.807404	79.06901	14.81912
0	3.801635	7.287814	0	1075.075	1578.466	1137.794	1563.286	827.0476	1539.811	0	0	0
957.117	0	0	0	344.8432	770.6971	494.5344	102.5771	0	261.6852	0	80.51602	0
82.68275	167.6705	44.5527	583.8865	14.3616	33.31253	24.53358	24.58379	178.3096	0	0	107.6692	96.79043
934.2878	270.6398	48.50356	1622.674	39.5984	15.19603	42.70596	0	182.2037	12.833	0	13.05346	0
0	444.1041	675.476	66.32446	52.92369	77.43936	0	126.7657	958.9455	123.2502	103.0534	1425.715	29.95287

954.6578	294.4377	7.585566	1602.789	30.75392	12.672	39.11169	0	0	0	0	16.292	0
0	0	19.09201	141.7552	0	0	24.27995	0	0	16.75614	0	0	0
3436.519	65.34185	62.39334	12.53232	1185.436	4493.284	1426.851	237.3877	240.557	332.9256	0	98.42423	0
464.9586	0	0	0	435.1209	3472.114	367.1524	182.0051	0	581.307	0	99.37046	0
0	0	0	0	0	123.6967	0	0	0	0	0	0	0
0	20.28791	24.9783	7.330808	31.04744	9.716543	17.1449	1473.862	128.4702	1888.541	28.94382	47.31487	0
3178.055	0	0	0	1251.487	4206.023	1484.095	52.72282	0	145.5888	0	99.63846	0
3178.055	0	0	0	1251.487	4206.023	1484.095	52.72282	0	145.5888	0	99.63846	0
0	0	0	0	1528.934	1278.265	2268.332	125.9922	32.38226	178.8071	1716.489	2244.086	1241.175
0	0	0	0	0	1744.219	0	0	0	23.16441	0	0	0
0	0	0	0	0	3561.758	0	0	0	17.51861	0	0	0
0	12.94718	17.01327	7.330808	28.76778	0	0	1215.555	0	1625.45	20.70458	40.46559	0
0	0	0	0	0	58.90252	0	0	0	0	0	0	0
0	35.02041	0	0	20.95004	270.8817	28.88489	25.84895	0	47.14685	329.3878	87.13577	300.0134
0	0	0	0	40.05512	0	105.3325	47.679	0	226.9852	0	23.152	0
2377.105	0	0	0	936.292	3633.07	1155.391	84.6658	0	187.9734	0	135.3741	0
3636.075	60.86585	153.1629	32.39	2399.887	2926.038	3096.256	229.6985	0	908.2646	26.61745	98.6331	0
4349.253	58.25365	148.4075	30.94301	2771.108	3692.626	3303.521	219.2697	0	869.7583	25.43445	94.39861	0
0	0	0	0	90.42069	628.048	127.1512	347.1486	75.56781	502.8421	0	427.3652	0
2933.458	0	0	0	1165.562	3834.613	1344.922	38.69789	0	128.4572	0	42.47345	0
0	33.10761	152.1539	0	0	0	17.02455	36.58176	24.80885	55.98186	0	10.39629	0
2960.795	0	0	0	2059.558	2220.871	1939.127	55.11748	0	67.12049	0	0	0
16.75883	0	0	0	0	1421.366	0	0	0	0	0	0	0
2815.917	0	0	0	1127.565	4432.711	1285.96	80.80666	0	147.4176	0	88.9934	0
712.1981	0	0	0	806.2413	1336.443	647.3199	47.04494	0	57.26325	0	0	0
0	0	18.44615	135.3166	0	0	23.98904	0	0	16.60879	0	0	0
75.18717	0	0	0	0	0	0	0	0	0	0	16.24657	0
83.43029	0	0	0	0	0	0	0	125.148	0	0	0	0
0	0	0	0	27.95847	116.7454	24.00251	21.5846	0	40.19866	0	0	0
0	0	0	0	37.17531	728.837	58.39011	24.98735	0	29.73027	0	0	0
0	1554.248	819.6802	78.85065	1194.505	933.075	1521.272	0	0	0	9736.497	9650.749	7534.038
0	0	0	0	0	2292.817	0	0	0	0	0	0	0
0	0	0	0	0	2292.817	0	0	0	0	0	0	0
0	0	0	0	0	2292.817	0	0	0	0	0	0	0
2.191195	8.657402	15.63801	18.02528	24.32534	1694.561	32.4424	194.175	0	468.063	0	4.10496	0

0	0	0	0	0	0	0	0	0	0	0	0	0
0	0	0	0	25.9907	269.1563	47.90676	38.05897	0	79.07849	0	0	0
0	7.133506	0	0	0	0	0	221.5795	0	505.8364	0	0	0
0	6.192257	0	0	0	0	0	197.5226	0	451.1526	0	0	0
0	0	180.6223	35.60414	12.91303	162.9085	61.2604	191.3805	0	234.3299	160.7938	195.1654	191.6004
0	0	0	0	0	0	0	0	0	19.50044	66.34126	0	24.19535
0	0	0	0	0	0	0	0	0	17.97183	70.78037	0	23.09137
0	0	0	0	0	0	0	0	0	0	0	0	0
0	0	0	0	0	0	0	0	0	0	0	66.98882	0
0	0	0	279.3865	0	0	0	168.2682	142.5712	280.7326	0	0	0
0	20.85478	0	0	58.07482	268.3521	61.03127	17.25693	46.82826	0	0	0	0
519.9266	0	107.4185	1190.233	0	0	0	0	0	36.91209	0	0	0
0	0	0	0	0	0	0	0	0	0	0	0	0
0	0	0	0	0	0	0	0	0	0	0	0	0
0	0	0	0	0	0	0	0	0	0	0	0	0
0	0	0	0	0	0	0	46.53104	0	11.77192	2.607955	8.262157	0
0	0	0	0	0	0	31.56684	0	0	0	0	0	0
0	0	0	0	0	0	0	44.53498	0	11.71648	1.830479	7.710499	0
0	0	0	0	0	0	0	0	0	0	0	0	0
0	0	0	0	0	0	0	0	0	0	0	0	0
0	0	0	265.868	0	1233.613	0	0	0	0	0	0	0
0	0	0	0	0	0	0	0	0	0	0	0	0
0	0	0	0	0	0	0	0	0	0	0	0	0
0	0	0	0	22.7003	52.09415	71.76541	0	679.5094	0	0	0	0
0	0	0	0	0	0	0	0	0	0	0	0	0
0	0	0	0	0	0	0	0	0	0	0	0	0
0	0	0	0	0	0	0	0	0	0	0	0	0
0	0	0	0	0	0	0	0	0	0	0	0	0
0	0	0	0	0	0	0	0	0	0	0	0	0
0	0	0	0	0	0	0	0	0	0	0	0	0
0	0	0	0	0	0	0	0	0	0	0	0	0
0	0	0	0	0	0	0	0	0	0	0	0	0
0	0	0	0	0	0	0	0	0	0	0	0	0
0	0	0	0	0	0	0	0	0	0	0	0	0
0	0	0	0	0	0	0	0	0	0	0	0	0

0	0	0	0	0	0	0	0	0	0	0	0	0
0	0	0	0	0	0	0	0	0	0	0	0	0
0	0	0	0	0	0	0	0	0	0	0	0	0
0	0	0	0	0	0	0	0	0	0	0	0	0
0	0	0	0	0	13.37673	0	0	0	0	0	0	0
0	371.3183	262.4128	0	0	0	0	34.25019	0	84.77692	38.08246	633.0005	25.1716
0	0	0	0	0	0	0	0	0	0	246.6441	713.9955	184.9262
0	0	0	0	0	0	0	0	0	0	0	0	0
0	0	0	0	0	0	0	0	0	0	0	0	0
0	0	0	0	0	0	0	0	0	0	0	0	0
0	0	0	0	0	0	0	0	0	0	0	0	0
0	0	0	0	0	0	0	0	0	0	0	0	0
68.49778	20.55241	15.82267	1615.569	0	0	0	0	0	0	0	0	0
49.35823	15.12239	11.82156	401.1863	0	0	0	0	0	0	0	0	0
0	0	0	0	0	1582.311	0	0	0	0	0	0	0
0	0	0	0	2211.44	1627.14	1469.598	0	0	0	0	0	0
0	0	0	0	1541.882	959.1522	928.906	0	0	0	0	0	0
0	0	0	0	641.2793	913.9715	1145.867	0	0	0	0	0	0
0	0	0	0	588.593	446.7393	465.3842	0	0	0	0	0	0
0	0	0	0	687.9274	580.8126	449.9861	0	0	0	0	0	0
0	0	0	0	511.8786	393.2709	415.7737	0	0	0	0	0	0
0	0	0	0	525.8775	405.6335	401.6357	0	0	0	0	0	0
0	0	0	0	588.5582	486.7993	425.1959	0	0	0	0	0	0
0	0	0	0	596.6162	368.512	353.3676	0	0	0	0	0	0
0	0	0	0	571.363	454.6636	410.2595	0	0	0	0	0	0
0	0	0	0	544.9503	454.6636	387.3175	0	0	0	0	0	0
0	0	0	0	430.284	335.7701	340.1528	0	0	0	0	0	0
0	0	0	0	131.2993	499.2472	110.5112	0	0	0	0	0	0
0	0	0	0	131.2993	499.2472	110.5112	0	0	0	0	0	0
0	0	0	0	0	26.38078	0	0	0	0	0	0	0
0	0	0	0	0	0	0	0	0	0	0	0	0
0	0	0	0	0	0	0	0	0	0	0	0	0
0	0	0	0	0	0	0	0	0	0	0	0	0
0	42.01205	0	0	0	680.0527	0	0	0	0	0	0	0
0	0	0	0	0	0	0	0	0	13.05902	0	0	0

0	0	0	0	0	0	0	0	0	16.65868	0	0	0
0	0	0	0	0	0	0	0	0	0	0	0	0
0	0	0	0	0	0	0	0	0	0	0	0	0
0	0	0	0	0	0	0	0	0	0	0	0	0
0	0	0	0	0	0	0	0	0	101.512	0	0	0
0	0	0	0	0	0	0	0	0	0	0	0	0
0	0	0	0	0	0	0	0	0	0	0	0	0
0	0	0	0	0	0	0	0	0	0	0	0	0
0	0	0	0	0	0	0	0	0	0	0	0	0
0	0	0	0	0	0	0	0	0	0	0	0	0
0	0	0	0	0	1366.932	0	0	0	0	0	0	0
0	0	0	0	0	887.6377	0	0	0	0	0	0	0
0	0	0	0	0	0	0	0	0	0	0	0	0
0	0	0	0	0	0	0	0	0	0	0	0	0
0	0	0	0	0	0	0	0	0	0	0	0	0
0	0	0	0	0	0	0	0	0	0	0	0	0
0	0	0	0	0	0	7.608587	0	0	0	0	0	0

Table S5. Differential pathways of MP and SP

SampleID	MP [1] mean rel. freq. (%)	MP std. dev. (%)	SP [2] mean rel. freq. (%)	SP std. dev. (%)
CITRULBIO-PWY__L-citrulline_biosynthesis	0.021159034	0.003706412	0.013424167	0.006534992
PHOSLIPSYN-PWY__superpathway_of_phospholipid_biosynthesis_I_(bacteria)	0.024598431	0.005740694	0.018865418	0.004391376
PWY-3001__superpathway_of_L-isoleucine_biosynthesis_I	0.027153344	0.003672868	0.022410985	0.003675898
PWY-5724__superpathway_of_atrazine_degradation	4.75665E-05	4.33309E-05	1.2572E-05	1.48982E-05
PWY-6147__6-hydroxymethyl-dihydropterin_diphosphate_biosynthesis_I	0.026769073	0.005984499	0.018191989	0.006053484
PWY-6270__isoprene_biosynthesis_I	0.028339074	0.005793414	0.02230317	0.00450116
PWY-6470__peptidoglycan_biosynthesis_V_(β-lactam_resistance)	0.005099759	0.003050499	0.00212527	0.001478329
PWY-6859__all-trans-farnesol_biosynthesis	0.010041589	0.002865498	0.005805721	0.003402999
PWY-6897__thiamine_diphosphate_salvage_II	0.023426136	0.005226355	0.018353139	0.003887325
PWY-7391__isoprene_biosynthesis_II_(engineered)	4.13212E-06	1.11252E-05	5.54879E-05	4.22582E-05
PWY-7392__taxadiene_biosynthesis_(engineered)	0.017136158	0.004958658	0.010189544	0.004908223
PWY-7399__methylphosphonate_degradation_II	4.23326E-05	4.89119E-05	5.44044E-06	7.74347E-06
PWY-7560__methylerythritol_phosphate_pathway_II	0.028076995	0.005491129	0.021877777	0.004754633
PWY-7663__gondoate_biosynthesis_(anaerobic)	0.036566332	0.006767871	0.027639163	0.006701987
PWY1G-0__mycothiol_biosynthesis	0.000155096	0.00019664	6.19886E-06	1.38611E-05
RHAMCAT-PWY__L-rhamnose_degradation_I	0.024521188	0.00401655	0.01787698	0.00378012
THISYNARA-PWY__superpathway_of_thiamine_diphosphate_biosynthesis_III_(eukaryotes)	0.017496563	0.006500459	0.012840899	0.002574305

[1] MAP: mild acute pancreatitis

[2] SAP: severe acute pancreatitis

p-values	Difference between means	95.0% lower CI	95.0% upper CI
0.045106178	0.007734867	0.000228735	0.015240999
0.045352695	0.005733013	0.000139318	0.011326709
0.037380656	0.004742358	0.000342368	0.009142349
0.024744211	3.49945E-05	5.03482E-06	6.49543E-05
0.024983113	0.008577084	0.00134341	0.015810759
0.039873936	0.006035903	0.000329479	0.011742328
0.01533738	0.002974489	0.000647486	0.005301493
0.039877345	0.004235868	0.000249785	0.008221951
0.047065932	0.005072998	7.688E-05	0.010069115
0.041431019	-5.13558E-05	-9.98278E-05	-2.88373E-06
0.025413398	0.006946614	0.001060899	0.012832329
0.02434185	3.68922E-05	5.57285E-06	6.82115E-05
0.039912087	0.006199218	0.000343305	0.012055131
0.032991668	0.008927169	0.000891044	0.016963294
0.022539126	0.000148897	2.47994E-05	0.000272994
0.008967939	0.006644208	0.00207027	0.011218146
0.049556722	0.004655664	1.01117E-05	0.009301216

Table S6. Differential pathways of BP and HP

SampleID	BP [1] mean rel. freq. (%)	BP std. dev. (%)	HP [2] mean rel. freq. (%)	HP std. dev. (%)
ARGDEG-PWY__superpathway_of_L-arginine,_putrescine,_and_4-aminobutanoate_degradatio	0.002165699	0.002769785	7.60173E-05	0.000101631
GLYCOLYSIS-TCA-GLYOX-BYPASS__superpathway_of_glycolysis,_pyruvate_dehydrogenase,	0.003108903	0.003238529	0.000759234	0.001224408
P125-PWY__superpathway_of_(R,R)-butanediol_biosynthesis	0.00012687	0.000128038	3.3783E-05	1.34956E-05
P4-PWY__superpathway_of_L-lysine,_L-threonine_and_L-methionine_biosynthesis_I	0.008436237	0.005822861	0.001418797	0.001607912
P461-PWY__hexitol_fermentation_to_lactate,_formate,_ethanol_and_acetate	0.010125787	0.007130266	0.002911391	0.002422156
PRPP-PWY__superpathway_of_histidine,_purine,_and_pyrimidine_biosynthesis	0.005832252	0.004015023	0.001685457	0.001823126
PWY-241__C4_photosynthetic_carbon_assimilation_cycle,_NADP-ME_type	0.008123295	0.003661533	0.001815976	0.000921509
PWY-5138__fatty_acid_β-oxidation_IV_(unsaturated,_even_number)	0.004206018	0.003721562	0.000851014	0.000934201
PWY-5464__superpathway_of_cytosolic_glycolysis_(plants),_pyruvate_dehydrogenase_and_TC	0.000706457	0.000955348	6.92997E-05	8.58292E-05
PWY-5837__2-carboxy-1,4-naphthoquinol_biosynthesis	0.00521974	0.003771746	0.001516086	0.001014942
PWY-6318__L-phenylalanine_degradation_IV_(mammalian,_via_side_chain)	0.000305106	0.000364949	2.04943E-05	2.59652E-05
PWY-6961__L-ascorbate_degradation_II_(bacterial,_aerobic)	0.00471578	0.00401642	0.000495049	0.000440703
PWY-7039__phosphatidate_metabolism,_as_a_signaling_molecule	0.000755165	0.001093534	7.94508E-06	1.06072E-05
PWY-7115__C4_photosynthetic_carbon_assimilation_cycle,_NAD-ME_type	0.005703364	0.004266004	0.001272676	0.001306303
PWY-7117__C4_photosynthetic_carbon_assimilation_cycle,_PEPCK_type	0.0115534	0.008056271	0.002172106	0.001115017
PWY-724__superpathway_of_L-lysine,_L-threonine_and_L-methionine_biosynthesis_II	0.030228163	0.00570155	0.036768144	0.002787232
PWY-7384__anaerobic_energy_metabolism_(invertebrates,_mitochondrial)	0.000563639	0.000551089	7.8568E-06	4.38355E-06
PWY-7385__1,3-propanediol_biosynthesis_(engineered)	0.002767547	0.003098647	0.000295572	0.00025031
PWY-7388__octanoyl-[acyl-carrier_protein]_biosynthesis_(mitochondria,_yeast)	0.003136693	0.004223304	5.76872E-05	6.89727E-05
PWY-7409__phospholipid_remodeling_(phosphatidylethanolamine,_yeast)	0.000664101	0.000764712	1.04035E-05	1.03126E-05
PWY-8086__(S)-lactate_fermentation_to_propanoate,_acetate_and_hydrogen	0.000568838	0.000566088	2.14944E-05	3.30833E-05
PWY0-301__L-ascorbate_degradation_I_(bacterial,_anaerobic)	0.00599546	0.006299551	0.000532705	0.00049909
AEROBACTINSYN-PWY__aerobactin_biosynthesis	0.00086473	0.000945911	2.40676E-05	4.81352E-05
ANAGLYCOLYSIS-PWY__glycolysis_III_(from_glucose)	0.034962533	0.005367186	0.04195172	0.00466697
ARG+POLYAMINE-SYN__superpathway_of_arginine_and_polyamine_biosynthesis	0.01296546	0.006004334	0.004675901	0.00270059
ARGININE-SYN4-PWY__L-ornithine_biosynthesis_II	0.021047701	0.008819158	0.032108769	0.004141699
ARO-PWY__chorismate_biosynthesis_I	0.035886119	0.007348439	0.045502356	0.001805422
AST-PWY__L-arginine_degradation_II_(AST_pathway)	0.003557747	0.003774464	0.000487176	0.000685444
BRANCHED-CHAIN-AA-SYN-PWY__superpathway_of_branched_chain_amino_acid_biosynthe	0.033285204	0.005498978	0.039125992	0.002926403
CARNMET-PWY__L-carnitine_degradation_I	0.000405138	0.000481673	2.44893E-05	3.56558E-05
COA-PWY-1__superpathway_of_coenzyme_A_biosynthesis_III_(mammals)	0.033814407	0.009031048	0.046517962	0.002986842
COA-PWY__coenzyme_A_biosynthesis_I_(prokaryotic)	0.03281977	0.008353899	0.045009322	0.00263839

COMPLETE-ARO-PWY__superpathway_of_aromatic_amino_acid_biosynthesis	0.033327019	0.006134028	0.039641681	0.002326273
DAPLYSINESYN-PWY__L-lysine_biosynthesis_I	0.01219013	0.006092228	0.003405331	0.002354379
DARABCATK12-PWY__D-arabinose_degradation_I	0.001237033	0.001339701	2.37327E-05	2.95941E-05
DENOVOPURINE2-PWY__superpathway_of_purine_nucleotides_de_novo_biosynthesis_II	0.005279634	0.004031289	0.001434318	0.001749955
DTDPRHAMSYN-PWY__dTDP-β-L-rhamnose_biosynthesis	0.052051795	0.018360696	0.075574574	0.016105196
ECASYN-PWY__enterobacterial_common_antigen_biosynthesis	0.002390164	0.00269509	0.000498336	0.000805294
FAO-PWY__fatty_acid_β-oxidation_I_(generic)	0.008581724	0.007822897	0.001207769	0.00122635
FERMENTATION-PWY__mixed_acid_fermentation	0.011292899	0.007516619	0.004866456	0.001550686
FOLSYN-PWY__superpathway_of_tetrahydrofolate_biosynthesis_and_salvage	0.004551286	0.0028932	0.000533115	0.000407788
FUC-RHAMCAT-PWY__superpathway_of_fucose_and_rhamnose_degradation	0.010418161	0.004709665	0.005284887	0.001076823
FUCCAT-PWY__fucose_degradation	0.007496646	0.004708682	0.00288439	0.000844301
GALACT-GLUCUROCAT-PWY__superpathway_of_hexuronide_and_hexuronate_degradation	0.007182317	0.002050692	0.003372339	0.001292944
GALACTARDEG-PWY__D-galactarate_degradation_I	0.010316187	0.007239478	0.002270532	0.000717292
GALACTITOLCAT-PWY__galactitol_degradation	0.003494662	0.003076415	0.000254999	0.000241693
GLUCARDEG-PWY__D-glucarate_degradation_I	0.006352149	0.005385259	0.001139788	0.000640606
GLUCARGALACTSUPER-PWY__superpathway_of_D-glucarate_and_D-galactarate_degradatic	0.010316187	0.007239478	0.002270532	0.000717292
GLUDEG-I-PWY__GABA_shunt	0.006334703	0.003713241	0.000836595	0.000753745
GLYCOL-GLYOXDEG-PWY__superpathway_of_glycol_metabolism_and_degradation	0.001271806	0.001366357	0.000138207	0.000240764
GLYOXYLATE-BYPASS__glyoxylate_cycle	0.006350418	0.005657803	0.0009123	0.000655085
HCAMHPDEG-PWY__3-phenylpropanoate_and_3-(3-hydroxyphenyl)propanoate_degradation_t	0.003220125	0.002930772	0.000359931	0.000390667
HEME-BIOSYNTHESIS-II-1__heme_b_biosynthesis_V_(aerobic)	0.004738799	0.004192032	0.000617985	0.000574751
HEME-BIOSYNTHESIS-II__heme_b_biosynthesis_I_(aerobic)	0.005238325	0.003915329	0.001054776	0.000798406
HEMESYN2-PWY__heme_b_biosynthesis_II_(oxygen-independent)	0.006091146	0.004640277	0.001082329	0.000652195
HEXITOLDEGSUPER-PWY__superpathway_of_hexitol_degradation_(bacteria)	0.006916166	0.005128051	0.000715965	0.00064214
HISDEG-PWY__L-histidine_degradation_I	0.018624219	0.00692709	0.030214282	0.002780766
HISTSYN-PWY__L-histidine_biosynthesis	0.031850331	0.009306337	0.047846047	0.004435592
HOMOSER-METSYN-PWY__L-methionine_biosynthesis_I	0.006924609	0.006487952	0.000581437	0.000660541
ILEUSYN-PWY__L-isoleucine_biosynthesis_I_(from_threonine)	0.034946062	0.005508684	0.041049551	0.002913155
KDO-NAGLIPASYN-PWY__superpathway_of_(Kdo)2-lipid_A_biosynthesis	0.001820267	0.001390732	0.000429443	0.000679522
KETOGLUCONMET-PWY__ketogluconate_metabolism	0.004056512	0.003288608	0.000261902	0.000236756
LIPASYN-PWY__phospholipases	0.003860612	0.004068724	0.000839674	0.000961965
LPSSYN-PWY__superpathway_of_lipopolysaccharide_biosynthesis	0.001225107	0.001540188	5.93136E-05	9.31602E-05
MET-SAM-PWY__superpathway_of_S-adenosyl-L-methionine_biosynthesis	0.010134531	0.007902107	0.001117903	0.001249712
METHGLYUT-PWY__superpathway_of_methylglyoxal_degradation	0.002069196	0.00134375	0.000778743	0.000509874
METSYN-PWY__superpathway_of_L-homoserine_and_L-methionine_biosynthesis	0.009782272	0.007983908	0.000995719	0.001118582

NAD-BIOSYNTHESIS-II__NAD_salvage_pathway_III_(to_nicotinamide_riboside)	0.003264144	0.002900905	0.000461401	0.000578936
NONMEVIPP-PWY__methylerythritol_phosphate_pathway_I	0.036115982	0.009533291	0.049373857	0.003499856
ORNARGDEG-PWY__superpathway_of_L-arginine_and_L-ornithine_degradation	0.002165699	0.002769785	7.60173E-05	0.000101631
ORNDEG-PWY__superpathway_of_ornithine_degradation	0.006744571	0.005950284	0.000969586	0.000765535
P105-PWY__TCA_cycle_IV_(2-oxoglutarate_decarboxylase)	0.006006466	0.005736605	0.000859364	0.000971375
P122-PWY__heterolactic_fermentation	0.003006976	0.003004581	0.000357157	0.000496696
P161-PWY__acetylene_degradation_(anaerobic)	0.009845579	0.007457707	0.002907745	0.002160364
P164-PWY__purine_nucleobases_degradation_I_(anaerobic)	0.007782676	0.003653876	0.004252035	0.000947239
P42-PWY__incomplete_reductive_TCA_cycle	0.003092855	0.001531958	0.001599421	0.000973828
P441-PWY__superpathway_of_N-acetylneuraminate_degradation	0.014171885	0.006112692	0.006569042	0.003349056
PANTO-PWY__phosphopantothenate_biosynthesis_I	0.031513772	0.007770404	0.041947986	0.003087997
PANTOSYN-PWY__superpathway_of_coenzyme_A_biosynthesis_I_(bacteria)	0.030576537	0.008409132	0.042520638	0.002445194
PEPTIDOGLYCANSYN-PWY__peptidoglycan_biosynthesis_I_(meso-diaminopimelate_containi	0.037536306	0.009035896	0.046874457	0.002754402
POLYAMINSYN3-PWY__superpathway_of_polyamine_biosynthesis_II	0.001514915	0.001677623	0.000376001	0.000295654
POLYAMSYN-PWY__superpathway_of_polyamine_biosynthesis_I	0.010005722	0.005769044	0.002722161	0.001735773
PWY-1861__formaldehyde_assimilation_II_(assimilatory_RuMP_Cycle)	0.00326008	0.003181725	0.0008619	0.000388078
PWY-2942__L-lysine_biosynthesis_III	0.034075647	0.006514541	0.042997214	0.002919716
PWY-3841__folate_transformations_II_(plants)	0.036394777	0.007661073	0.045922481	0.001632908
PWY-4041__γ-glutamyl_cycle	0.01136976	0.006319512	0.004243467	0.00160078
PWY-4984__urea_cycle	0.011731182	0.006582538	0.017924786	0.001801792
PWY-5022__4-aminobutanoate_degradation_V	0.006086959	0.00315399	0.001113681	0.000827731
PWY-5030__L-histidine_degradation_III	0.015783038	0.008126553	0.025002942	0.004624169
PWY-5088__L-glutamate_degradation_VIII_(to_propanoate)	0.000289649	0.000356353	3.20665E-05	4.81374E-05
PWY-5097__L-lysine_biosynthesis_VI	0.0341227	0.00703892	0.042814872	0.002002611
PWY-5103__L-isoleucine_biosynthesis_III	0.030572433	0.00533914	0.035544826	0.003182588
PWY-5104__L-isoleucine_biosynthesis_IV	0.004867948	0.003630687	0.000269574	0.000264675
PWY-5136__fatty_acid_β-oxidation_II_(plant_peroxisome)	0.012957238	0.010107066	0.002942391	0.00099934
PWY-5180__toluene_degradation_I_(aerobic)_(via_o-cresol)	0.00361892	0.004299087	0	0
PWY-5188__tetrapyrrole_biosynthesis_I_(from_glutamate)	0.009861135	0.003666032	0.00571847	0.00124562
PWY-5189__tetrapyrrole_biosynthesis_II_(from_glycine)	0.004220387	0.003530375	0.000351306	0.000161833
PWY-5345__superpathway_of_L-methionine_biosynthesis_(by_sulfhydrylation)	0.012518588	0.004770895	0.004699197	0.003810844
PWY-5347__superpathway_of_L-methionine_biosynthesis_(transsulfuration)	0.010501916	0.008355954	0.001127156	0.001259034
PWY-5367__petroselinate_biosynthesis	0.002322129	0.001911276	0.00013424	0.000100828
PWY-5384__sucrose_degradation_IV_(sucrose_phosphorylase)	0.014614169	0.004580235	0.01125517	0.001397241
PWY-5415__catechol_degradation_I_(meta-cleavage_pathway)	8.1348E-05	9.96973E-05	4.4019E-06	8.8038E-06

PWY-5497__purine_nucleobases_degradation_II_(anaerobic)	0.006265439	0.002066687	0.003135583	0.000444892
PWY-561__superpathway_of_glyoxylate_cycle_and_fatty_acid_degradation	0.00741565	0.006660459	0.001096894	0.000973318
PWY-5656__mannosylglycerate_biosynthesis_I	0.003279573	0.002810423	0.000167718	0.000175008
PWY-5667__CDP-diacylglycerol_biosynthesis_I	0.0364088	0.006450074	0.046252298	0.001232808
PWY-5675__nitrate_reduction_V_(assimilatory)	0.007104794	0.006004014	0.000682092	0.0006353
PWY-5676__acetyl-CoA_fermentation_to_butanoate_II	0.005792106	0.001834401	0.003301172	0.001347329
PWY-5686__UMP_biosynthesis_I	0.038520792	0.010222778	0.053179635	0.002669465
PWY-5692__allantoin_degradation_to_glyoxylate_II	0.000515094	0.000446623	1.32977E-05	1.80959E-05
PWY-5695__inosine_5'-phosphate_degradation	0.038839676	0.008363251	0.049829096	0.002938641
PWY-5705__allantoin_degradation_to_glyoxylate_III	0.001159192	0.000994843	8.10847E-05	7.00147E-05
PWY-5723__Rubisco_shunt	0.010669912	0.008673465	0.001113603	0.001162384
PWY-5747__2-methylcitrate_cycle_II	0.001361177	0.001806737	3.54399E-05	1.83473E-05
PWY-5838__superpathway_of_menaquinol-8_biosynthesis_I	0.010120303	0.004850699	0.00450046	0.00281748
PWY-5840__superpathway_of_menaquinol-7_biosynthesis	0.007365415	0.004631632	0.002317954	0.001739405
PWY-5845__superpathway_of_menaquinol-9_biosynthesis	0.008596019	0.004192688	0.004308081	0.002672903
PWY-5850__superpathway_of_menaquinol-6_biosynthesis	0.007190089	0.004825037	0.001968335	0.002008775
PWY-5855__ubiquinol-7_biosynthesis_(early_decarboxylation)	0.001712336	0.001882709	0.000297723	0.000525753
PWY-5860__superpathway_of_demethylmenaquinol-6_biosynthesis_I	0.005704721	0.004279808	0.00136095	0.00141388
PWY-5861__superpathway_of_demethylmenaquinol-8_biosynthesis_I	0.00822421	0.004533689	0.003187973	0.002050333
PWY-5862__superpathway_of_demethylmenaquinol-9_biosynthesis	0.006771287	0.003776745	0.003041281	0.001936372
PWY-5896__superpathway_of_menaquinol-10_biosynthesis	0.007190089	0.004825037	0.001968335	0.002008775
PWY-5897__superpathway_of_menaquinol-11_biosynthesis	0.009608681	0.004842498	0.004061162	0.002583101
PWY-5898__superpathway_of_menaquinol-12_biosynthesis	0.009608681	0.004842498	0.004061162	0.002583101
PWY-5899__superpathway_of_menaquinol-13_biosynthesis	0.009608681	0.004842498	0.004061162	0.002583101
PWY-5913__partial_TCA_cycle_(obligate_autotrophs)	0.008408866	0.007118206	0.001282749	0.000769177
PWY-5918__superpathway_of_heme_b_biosynthesis_from_glutamate	0.005634052	0.004010202	0.00112916	0.000790693
PWY-5920__superpathway_of_heme_b_biosynthesis_from_glycine	0.003247456	0.002577914	0.000409009	0.000269025
PWY-5971__palmitate_biosynthesis_(type_II_fatty_acid_synthase)	0.018107692	0.006531561	0.007059057	0.003920958
PWY-5972__stearate_biosynthesis_I_(animals)	0.000583566	0.000709052	6.49285E-05	6.49256E-05
PWY-5973__cis-vaccenate_biosynthesis	0.030954675	0.008591861	0.043206789	0.003093263
PWY-6071__superpathway_of_phenylethylamine_degradation	0.000780813	0.001206604	0	0
PWY-6121__5-aminoimidazole_ribonucleotide_biosynthesis_I	0.035450177	0.00725157	0.046261238	0.003521545
PWY-6122__5-aminoimidazole_ribonucleotide_biosynthesis_II	0.035189842	0.006854347	0.04573407	0.00386505
PWY-6123__inosine-5'-phosphate_biosynthesis_I	0.029768934	0.008956018	0.043397751	0.002288255
PWY-6124__inosine-5'-phosphate_biosynthesis_II	0.028768205	0.009205309	0.042495811	0.002434662

PWY-6126__superpathway_of_adenosine_nucleotides_de_novo_biosynthesis_II	0.031948945	0.008638071	0.042853044	0.007456343
PWY-6147__6-hydroxymethyl-dihydropterin_diphosphate_biosynthesis_I	0.022084959	0.006864512	0.029592092	0.004901626
PWY-6163__chorismate_biosynthesis_from_3-dehydroquinate	0.037108937	0.008901647	0.048145063	0.002857701
PWY-6277__superpathway_of_5-aminoimidazole_ribonucleotide_biosynthesis	0.035189842	0.006854347	0.04573407	0.00386505
PWY-6293__superpathway_of_L-cysteine_biosynthesis_(fungi)	0.002141332	0.001725652	0.000559022	0.000761094
PWY-6353__purine_nucleotides_degradation_II_(aerobic)	0.015716609	0.006272979	0.004499461	0.000525814
PWY-6385__peptidoglycan_biosynthesis_III_(mycobacteria)	0.038611619	0.008584356	0.047610125	0.002842348
PWY-6386__UDP-N-acetylmuramoyl-pentapeptide_biosynthesis_II_(lysine-containing)	0.037734926	0.009292192	0.046689714	0.002605612
PWY-6387__UDP-N-acetylmuramoyl-pentapeptide_biosynthesis_I_(meso-diaminopimelate_cor	0.037865484	0.008999407	0.046722883	0.002682397
PWY-6531__mannitol_cycle	0.005531803	0.003905716	0.001668372	0.00082096
PWY-6545__pyrimidine_deoxyribonucleotides_de_novo_biosynthesis_III	0.006346778	0.002874659	0.002507027	0.002524446
PWY-6549__L-glutamine_biosynthesis_III	0.003963877	0.001806678	0.002101283	0.000698655
PWY-6606__guanosine_nucleotides_degradation_II	0.009928691	0.005398159	0.002709395	0.000687196
PWY-6608__guanosine_nucleotides_degradation_III	0.018202996	0.008202763	0.008690649	0.003491379
PWY-6609__adenine_and_adenosine_salvage_III	0.040328474	0.009776397	0.050270359	0.003150387
PWY-6612__superpathway_of_tetrahydrofolate_biosynthesis	0.003264301	0.002190244	0.000357505	0.000274901
PWY-6630__superpathway_of_L-tyrosine_biosynthesis	0.008663733	0.00362863	0.005356402	0.00150762
PWY-6690__cinnamate_and_3-hydroxycinnamate_degradation_to_2-hydroxypentadienoate	0.003220125	0.002930772	0.000359931	0.000390667
PWY-6700__queuosine_biosynthesis_I_(de_novo)	0.034281358	0.010195481	0.045794756	0.004591482
PWY-6703__preQ0_biosynthesis	0.025574171	0.008854503	0.036072892	0.005878137
PWY-6708__ubiquinol-8_biosynthesis_(early_decarboxylation)	0.000434523	0.000458192	1.41889E-05	1.62672E-05
PWY-6803__phosphatidylcholine_acyl_editing	0.005934512	0.005003141	0.000831305	0.000896885
PWY-6895__superpathway_of_thiamine_diphosphate_biosynthesis_II	0.013084158	0.004878037	0.007155945	0.004138619
PWY-6969__TCA_cycle_V_(2-oxoglutarate_synthase)	0.008156029	0.00585866	0.003899758	0.001042629
PWY-702__L-methionine_biosynthesis_II	0.00887794	0.004701588	0.003589816	0.00072121
PWY-7111__pyruvate_fermentation_to_isobutanol_(engineered)	0.029361135	0.00724671	0.022955504	0.003040245
PWY-7118__chitin_deacetylation	0.006022501	0.005578056	0.000610396	0.00049368
PWY-7184__pyrimidine_deoxyribonucleotides_de_novo_biosynthesis_I	0.008875571	0.004434197	0.002795585	0.00291437
PWY-7187__pyrimidine_deoxyribonucleotides_de_novo_biosynthesis_II	0.003129352	0.002706832	0.000720728	0.000941494
PWY-7199__pyrimidine_deoxyribonucleosides_salvage	0.030999496	0.008727523	0.041973197	0.005531296
PWY-7204__pyridoxal_5'-phosphate_salvage_II_(plants)	0.003575775	0.002982554	0.000380403	0.000235471
PWY-7209__superpathway_of_pyrimidine_ribonucleosides_degradation	0.000707127	0.000566468	0.00021866	0.000200706
PWY-7211__superpathway_of_pyrimidine_deoxyribonucleotides_de_novo_biosynthesis	0.006638064	0.003666528	0.001947681	0.00142689
PWY-7221__guanosine_ribonucleotides_de_novo_biosynthesis	0.038344024	0.008277945	0.051146678	0.002863369
PWY-7229__superpathway_of_adenosine_nucleotides_de_novo_biosynthesis_I	0.036030336	0.008875371	0.048020316	0.006479506

PWY-7269__mitochondrial_NADPH_production_(yeast)	0.002681471	0.003155609	0.000369529	0.000653665
PWY-7294__D-xylose_degradation_IV	0.000496974	0.000624883	6.38359E-05	5.40385E-05
PWY-7328__superpathway_of_UDP-glucose-derived_O-antigen_building_blocks_biosynthesis	0.014323918	0.004212664	0.0079694	0.003055519
PWY-7345__superpathway_of_anaerobic_sucrose_degradation	0.018818578	0.004133476	0.013483734	0.003449906
PWY-7389__superpathway_of_anaerobic_energy_metabolism_(invertebrates)	0.000624292	0.00071225	1.26066E-05	6.99125E-06
PWY-7391__isoprene_biosynthesis_II_(engineered)	2.76175E-05	3.81171E-05	0	0
PWY-7446__sulfoquinovose_degradation_I	0.000951276	0.001054654	4.467E-05	3.25076E-05
PWY-7616__methanol_oxidation_to_carbon_dioxide	0.000402646	0.000596512	1.79543E-05	1.69771E-05
PWY-7663__gondoate_biosynthesis_(anaerobic)	0.031326434	0.007693756	0.040525446	0.003213722
PWY-7761__NAD_salvage_pathway_II_(PNC_IV_cycle)	0.007748391	0.003348204	0.003284484	0.002022991
PWY-7790__UMP_biosynthesis_II	0.038520792	0.010222778	0.053179635	0.002669465
PWY-7791__UMP_biosynthesis_III	0.038520792	0.010222778	0.053179635	0.002669465
PWY-7805__(aminomethyl)phosphonate_degradation	0.000778638	0.000912583	1.11755E-05	1.56582E-05
PWY-7807__glyphosate_degradation_III	0.000827728	0.000988465	1.00228E-05	1.40428E-05
PWY-7851__coenzyme_A_biosynthesis_II_(eukaryotic)	0.032156123	0.008877666	0.044388292	0.002687805
PWY-7858__(5Z)-dodecenoate_biosynthesis_II	0.006143561	0.005126816	0.000906508	0.001200537
PWY-7873__D-erythronate_degradation_II	0.000496171	0.000540307	5.08402E-06	6.77598E-06
PWY-7874__L-threonate_degradation	0.000563561	0.000581375	6.43192E-06	9.22667E-06
PWY-7883__anhydromuropeptides_recycling_II	0.002791527	0.001835957	0.000654824	0.000733247
PWY-7942__5-oxo-L-proline_metabolism	0.003174968	0.002746887	0.000641002	0.000704044
PWY-7953__UDP-N-acetylmuramoyl-pentapeptide_biosynthesis_III_(meso-diaminopimelate_cc	0.036709318	0.009935097	0.045663894	0.002913579
PWY-7977__L-methionine_biosynthesis_IV	0.036916998	0.007002873	0.047058447	0.001692191
PWY-801__homocysteine_and_cysteine_interconversion	0.001717595	0.001842866	0.000344695	0.000473044
PWY-821__superpathway_of_sulfur_amino_acid_biosynthesis_(Saccharomyces_cerevisiae)	0.006882962	0.00346676	0.001551904	0.001813954
PWY-841__superpathway_of_purine_nucleotides_de_novo_biosynthesis_I	0.028504403	0.006538493	0.036079879	0.00293906
PWY-I9__L-cysteine_biosynthesis_VI_(from_L-methionine)	0.012433055	0.005532343	0.004463271	0.00125135
PWY0-1061__superpathway_of_L-alanine_biosynthesis	0.005557539	0.004130487	0.001340783	0.001490706
PWY0-1221__putrescine_degradation_II	0.000479961	0.000574091	1.33161E-05	2.00517E-05
PWY0-1261__anhydromuropeptides_recycling_I	0.009972215	0.004612924	0.004959877	0.002023518
PWY0-1277__3-phenylpropanoate_and_3-(3-hydroxyphenyl)propanoate_degradation	0.004304269	0.003877483	0.000546384	0.00048929
PWY0-1297__superpathway_of_purine_deoxyribonucleosides_degradation	0.012075226	0.007753548	0.004849	0.003730469
PWY0-1298__superpathway_of_pyrimidine_deoxyribonucleosides_degradation	0.009772891	0.00634875	0.00295315	0.001775625
PWY0-1319__CDP-diacylglycerol_biosynthesis_II	0.036412531	0.006450044	0.046252298	0.001232808
PWY0-1337__oleate_β-oxidation	0.001715064	0.001650314	0.000505044	0.000669809
PWY0-1338__polymyxin_resistance	0.00291536	0.00249975	0.000490157	0.000620845

PWY0-1415__superpathway_of_heme_b_biosynthesis_from_uroporphyrinogen-III	0.004549783	0.004153254	0.000615849	0.000678194
PWY0-1477__ethanolamine_utilization	0.0162519	0.011456033	0.006158261	0.002857432
PWY0-1479__tRNA_processing	0.009792071	0.004940605	0.002689113	0.00135851
PWY0-1533__methylphosphonate_degradation_I	0.000504307	0.000614041	5.76857E-06	8.09258E-06
PWY0-321__phenylacetate_degradation_I_(aerobic)	0.000693239	0.001065588	0	0
PWY0-41__allantoin_degradation_IV_(anaerobic)	0.000779287	0.000734175	2.43869E-05	2.47849E-05
PWY0-42__2-methylcitrate_cycle_I	0.002357241	0.002151155	8.94676E-05	8.62816E-05
PWY0-461__L-lysine_degradation_I	0.000740997	0.000761328	0.000137889	0.000195018
PWY0-781__aspartate_superpathway	0.008442207	0.005341362	0.001487193	0.001679306
PWY0-845__superpathway_of_pyridoxal_5'-phosphate_biosynthesis_and_salvage	0.019901896	0.005239526	0.026806661	0.002068725
PWY1ZNC-1__assimilatory_sulfate_reduction_IV	0.00561438	0.005697771	0.000812101	0.000790832
PWY3O-4107__NAD_salvage_pathway_V_(PNC_V_cycle)	0.00506605	0.003186209	0.000641369	0.000513064
PWY4LZ-257__superpathway_of_fermentation_(Chlamydomonas_reinhardtii)	0.008684328	0.005384088	0.003152249	0.002237793
PWY66-389__phytol_degradation	0.003306585	0.004471947	0.000407369	0.000650666
PWY66-429__fatty_acid_biosynthesis_initiation_(mitochondria)	0.038150166	0.010085941	0.052090323	0.0044691
PWY66-430__myristate_biosynthesis_(mitochondria)	0.003081377	0.004121781	5.91943E-05	7.11359E-05
PYRIDNUCSAL-PWY__NAD_salvage_pathway_I_(PNC_VI_cycle)	0.005113522	0.002902073	0.000781966	0.000589836
PYRIDNUCSYN-PWY__NAD_de_novo_biosynthesis_I_(from_aspartate)	0.027641753	0.005941213	0.035889493	0.002736483
PYRIDOXSYN-PWY__pyridoxal_5'-phosphate_biosynthesis_I	0.018268409	0.005645019	0.0263287	0.002857398
REDCITCYC__TCA_cycle_VI_(Helicobacter)	0.00221309	0.002142979	0.000534157	0.000646945
RIBOSYN2-PWY__flavin_biosynthesis_I_(bacteria_and_plants)	0.026220349	0.007271282	0.034506966	0.003651701
SALVADEHYPOX-PWY__adenosine_nucleotides_degradation_II	0.01557131	0.009337596	0.002370991	0.000260953
SER-GLYSYN-PWY__superpathway_of_L-serine_and_glycine_biosynthesis_I	0.02814391	0.003941	0.035004715	0.001822876
SO4ASSIM-PWY__assimilatory_sulfate_reduction_I	0.01154145	0.008375677	0.00202666	0.001790748
SULFATE-CYS-PWY__superpathway_of_sulfate_assimilation_and_cysteine_biosynthesis	0.013447638	0.006958533	0.003543398	0.003116943
TCA-GLYOX-BYPASS__superpathway_of_glyoxylate_bypass_and_TCA	0.006829661	0.006087386	0.001020204	0.000950272
TCA__TCA_cycle_I_(prokaryotic)	0.007882868	0.006187929	0.002081521	0.00178533
THREOCAT-PWY__superpathway_of_L-threonine_metabolism	0.003070301	0.00367332	0.000570905	0.001028681
THRESYN-PWY__superpathway_of_L-threonine_biosynthesis	0.031747383	0.00573963	0.03836777	0.004155772
TRNA-CHARGING-PWY__tRNA_charging	0.036394938	0.008658162	0.04522765	0.001976327
UBISYN-PWY__superpathway_of_ubiquinol-8_biosynthesis_(early_decarboxylation)	0.000481871	0.000507505	1.57606E-05	1.79759E-05
UDPNAGSYN-PWY__UDP-N-acetyl-D-glucosamine_biosynthesis_I	0.01511802	0.005014984	0.009664663	0.003587339
URDEGR-PWY__superpathway_of_allantoin_degradation_in_plants	0.000515094	0.000446623	1.32977E-05	1.80959E-05
VALSYN-PWY__L-valine_biosynthesis	0.043056383	0.006436889	0.054092173	0.004053338

[1] BP: biliary acute pancreatitis

[2] HP: hypertriglyceridemia-induced pancreatitis

p-values	Difference between means	95.0% lower CI	95.0% upper CI
0.017591784	0.002089682	0.000427918	0.003751447
0.045766676	0.002349669	4.97691E-05	0.004649569
0.022111749	9.30875E-05	1.55064E-05	0.000170669
0.001191661	0.00701744	0.003209127	0.010825753
0.006397785	0.007214396	0.00231893	0.012109862
0.011573215	0.004146796	0.001074798	0.007218793
3.10296E-05	0.006307319	0.003950369	0.008664269
0.008914233	0.003355004	0.00096037	0.005749638
0.032858515	0.000637157	6.01111E-05	0.001214204
0.005473947	0.003703654	0.001248515	0.006158794
0.014971181	0.000284612	6.48494E-05	0.000504374
0.002307242	0.004220731	0.001784714	0.006656749
0.028480403	0.00074722	9.19383E-05	0.001402501
0.004432184	0.004430688	0.001579299	0.007282077
0.001066738	0.009381294	0.004457563	0.014305026
0.007867954	-0.006539981	-0.011064985	-0.002014977
0.00301572	0.000555782	0.000225561	0.000886002
0.013300497	0.002471974	0.000603291	0.004340657
0.020862164	0.003079006	0.000547842	0.005610171
0.008747168	0.000653697	0.000195418	0.001111977
0.004049257	0.000547344	0.000207021	0.000887667
0.008191441	0.005462755	0.00166468	0.009260829
0.006960921	0.000840662	0.00027246	0.001408865
0.03723882	-0.006989187	-0.013441243	-0.000537131
0.001547672	0.008289558	0.003715827	0.01286329
0.003770565	-0.011061067	-0.017919104	-0.004203031
0.000498913	-0.009616237	-0.014329277	-0.004903197
0.013563418	0.003070571	0.00072755	0.005413592
0.016337232	-0.005840788	-0.010415028	-0.001266548
0.013953147	0.000380648	9.04761E-05	0.000670821
0.000433472	-0.012703555	-0.018858064	-0.006549046
0.000271128	-0.012189553	-0.017814932	-0.006564174

0.00726548	-0.006314662	-0.010675524	-0.0019538
0.000573	0.008784799	0.004424572	0.013145025
0.006151572	0.001213301	0.000410202	0.002016399
0.016015375	0.003845315	0.000823201	0.006867429
0.04080926	-0.023522779	-0.045767164	-0.001278394
0.039664856	0.001891828	0.000100444	0.003683212
0.005219004	0.007373955	0.002564071	0.012183839
0.010750221	0.006426443	0.001707838	0.011145048
0.000230073	0.00401817	0.002248563	0.005787777
0.002184796	0.005133274	0.002142174	0.008124374
0.004187167	0.004612257	0.001692102	0.007532411
0.001108227	0.003809978	0.001906716	0.00571324
0.001506254	0.008045655	0.003665	0.01242631
0.002239467	0.003239663	0.001385053	0.005094274
0.004161655	0.005212362	0.001938812	0.008485911
0.001506254	0.008045655	0.003665	0.01242631
0.000132325	0.005498108	0.003170833	0.007825384
0.011987551	0.0011336	0.000287306	0.001979893
0.004350697	0.005438118	0.002001618	0.008874618
0.003991862	0.002860193	0.001071707	0.00464868
0.003813906	0.004120814	0.001559781	0.006681847
0.00235824	0.004183549	0.001728518	0.00663858
0.001939661	0.005008817	0.002170969	0.007846666
0.000776068	0.006200201	0.003077892	0.009322509
0.00016568	-0.011590063	-0.016618631	-0.006561496
0.000330051	-0.015995716	-0.023286169	-0.008705263
0.003831726	0.006343172	0.00241503	0.010271314
0.012737313	-0.006103489	-0.01066902	-0.001537958
0.01717755	0.001390824	0.000287369	0.002494279
0.001118485	0.00379461	0.001814058	0.005775162
0.025209085	0.003020937	0.000425541	0.005616334
0.017448878	0.001165793	0.000239633	0.002091953
0.00128507	0.009016628	0.004155665	0.013877591
0.011201432	0.001290453	0.000334965	0.002245942
0.001671323	0.008786553	0.003904515	0.013668591

0.004824922	0.002802743	0.000987547	0.004617939
0.000659364	-0.013257875	-0.019961083	-0.006554667
0.017591784	0.002089682	0.000427918	0.003751447
0.004124867	0.005774985	0.00214864	0.00940133
0.007354494	0.005147102	0.001603609	0.008690596
0.008133546	0.002649819	0.000796723	0.004502915
0.00854516	0.006937835	0.002013736	0.011861934
0.005861498	0.00353064	0.001167427	0.005893853
0.042205011	0.001493435	6.33376E-05	0.002923532
0.007485042	0.007602843	0.00242924	0.012776446
0.001176561	-0.010434214	-0.016053273	-0.004815155
0.000293984	-0.011944101	-0.017500785	-0.006387418
0.004556744	-0.009338151	-0.015371387	-0.003304916
0.033738884	0.001138914	9.98187E-05	0.002178009
0.000925027	0.007283561	0.003443031	0.011124091
0.018713147	0.00239818	0.000462596	0.004333764
0.001623635	-0.008921567	-0.013875844	-0.003967289
0.000700044	-0.009527704	-0.014353425	-0.004701984
0.001840512	0.007126293	0.003054268	0.011198317
0.007415051	-0.006193604	-0.010491702	-0.001895506
8.59156E-05	0.004973278	0.002929184	0.007017371
0.014575798	-0.009219903	-0.016261218	-0.002178589
0.023516958	0.000257582	4.00067E-05	0.000475158
0.001014465	-0.008692172	-0.013322653	-0.004061692
0.042343247	-0.004972393	-0.009740438	-0.000204348
0.000523246	0.004598374	0.002411516	0.006785231
0.003486702	0.010014847	0.003899254	0.01613044
0.009570268	0.00361892	0.001042999	0.00619484
0.002928995	0.004142665	0.001625504	0.006659826
0.001658262	0.003869082	0.001749427	0.005988736
0.00942757	0.007819391	0.002492682	0.0131461
0.001455627	0.009374761	0.004247852	0.014501669
0.001189562	0.002187889	0.001039579	0.003336199
0.033243476	0.003358999	0.000300259	0.006417739
0.016007812	7.69461E-05	1.67442E-05	0.000137148

0.000108489	0.003129856	0.001826632	0.004433079
0.004919128	0.006318756	0.002238242	0.01039927
0.001536655	0.003111856	0.001421498	0.004802213
9.54232E-05	-0.009843498	-0.013863964	-0.005823031
0.002017706	0.006422703	0.002784405	0.010061
0.016267798	0.002490934	0.000580142	0.004401726
0.000225096	-0.014658843	-0.021278608	-0.008039078
0.001373682	0.000501796	0.000233762	0.00076983
0.000946153	-0.01098942	-0.016788873	-0.005189968
0.001808675	0.001078107	0.000479105	0.00167711
0.001646631	0.009556309	0.004262274	0.014850343
0.020185248	0.001325738	0.000243072	0.002408403
0.01408107	0.005619843	0.00136117	0.009878516
0.004844036	0.005047461	0.001765829	0.008329093
0.03495717	0.004287938	0.000365957	0.008209919
0.006731871	0.005221754	0.001668074	0.008775433
0.027098347	0.001414613	0.000180629	0.002648598
0.005939736	0.00434377	0.001428035	0.007259505
0.007354821	0.005036236	0.001573951	0.008498522
0.021112604	0.003730006	0.00065451	0.006805502
0.006731871	0.005221754	0.001668074	0.008775433
0.010899471	0.005547519	0.001513819	0.00958122
0.010899471	0.005547519	0.001513819	0.00958122
0.010899471	0.005547519	0.001513819	0.00958122
0.003265489	0.007126117	0.00281047	0.011441764
0.001569285	0.004504892	0.001998379	0.007011406
0.00162135	0.002838446	0.001276788	0.004400105
0.00157695	0.011048635	0.005188089	0.016909181
0.021198179	0.000518638	9.02208E-05	0.000947055
0.000492567	-0.012252114	-0.018255311	-0.006248917
0.036345	0.000780813	5.78422E-05	0.001503785
0.001215667	-0.010811061	-0.016546421	-0.0050757
0.002142506	-0.010544228	-0.016449387	-0.004639068
0.000120192	-0.013628818	-0.019407595	-0.007850041
0.000154835	-0.013727606	-0.019701471	-0.007753741

0.040852335	-0.0109041	-0.021220855	-0.000587345
0.037986465	-0.007507134	-0.014498945	-0.000515322
0.001234823	-0.011036126	-0.017055066	-0.005017185
0.002142506	-0.010544228	-0.016449387	-0.004639068
0.020541543	0.00158231	0.000279502	0.002885119
1.9907E-05	0.011217148	0.007432212	0.015002084
0.004780245	-0.008998506	-0.014850414	-0.003146599
0.006524736	-0.008954787	-0.015050052	-0.002859523
0.006156566	-0.008857399	-0.014835897	-0.002878902
0.004216968	0.003863431	0.00140687	0.006319993
0.034966205	0.003839751	0.000353431	0.007326072
0.007548804	0.001862594	0.000569246	0.003155942
0.000324208	0.007219296	0.003930667	0.010507924
0.004541193	0.009512347	0.003414977	0.015609717
0.00560089	-0.009941885	-0.016558706	-0.003325065
0.000347174	0.002906796	0.001573121	0.004240471
0.018424665	0.00330733	0.00063703	0.005977631
0.003991862	0.002860193	0.001071707	0.00464868
0.006531972	-0.011513398	-0.019284254	-0.003742543
0.020736463	-0.010498721	-0.019028995	-0.001968447
0.005681887	0.000420334	0.00014546	0.000695209
0.00314035	0.005103207	0.002000495	0.008205919
0.044420061	0.005928213	0.00019036	0.011666066
0.024536227	0.00425627	0.000624947	0.007887594
0.001431915	0.005288125	0.002400742	0.008175508
0.022086651	0.006405631	0.001051445	0.011759817
0.004003876	0.005412105	0.002043689	0.00878052
0.009509683	0.006079985	0.001839557	0.010320414
0.014724092	0.002408624	0.000537324	0.004279925
0.012909095	-0.010973702	-0.01910362	-0.002843783
0.001973378	0.003195372	0.001397235	0.00499351
0.018085922	0.000488467	9.46496E-05	0.000882284
0.00162432	0.004690384	0.002059569	0.007321198
0.000198374	-0.012802654	-0.018516018	-0.00708929
0.01625623	-0.011989979	-0.021190283	-0.002789676

0.025010136	0.002311942	0.00033017	0.004293714
0.027440788	0.000433138	5.59276E-05	0.000810349
0.009048163	0.006354518	0.002010252	0.010698784
0.033366282	0.005334844	0.00054205	0.010127638
0.008506783	0.000611685	0.00018488	0.00103849
0.021494964	2.76175E-05	4.77855E-06	5.04564E-05
0.008480551	0.000906606	0.000274098	0.001539113
0.037004637	0.000384692	2.69941E-05	0.000742389
0.003440934	-0.009199012	-0.014873243	-0.003524782
0.007596353	0.004463907	0.001446561	0.007481252
0.000225096	-0.014658843	-0.021278608	-0.008039078
0.000225096	-0.014658843	-0.021278608	-0.008039078
0.00963306	0.000767462	0.000220507	0.001314418
0.01059241	0.000817705	0.000225323	0.001410088
0.000425812	-0.012232169	-0.018150505	-0.006313833
0.003617792	0.005237054	0.00197093	0.008503177
0.006008988	0.000491087	0.000167298	0.000814877
0.004267625	0.000557129	0.000208697	0.000905561
0.003619445	0.002136703	0.000806548	0.003466857
0.00785814	0.002533966	0.000760667	0.004307265
0.010562886	-0.008954576	-0.015531434	-0.002377718
0.000186455	-0.010141449	-0.014622069	-0.00566083
0.026292455	0.001372901	0.00018292	0.002562881
0.001421789	0.005331059	0.00247538	0.008186737
0.005489858	-0.007575475	-0.012554701	-0.002596249
0.000185393	0.007969785	0.004460929	0.01147864
0.006842605	0.004216756	0.001328506	0.007105006
0.011744832	0.000466645	0.000122255	0.000811035
0.007673212	0.005012338	0.001538167	0.008486509
0.004154408	0.003757884	0.00139639	0.006119378
0.02358112	0.007226225	0.001123103	0.013329348
0.003025103	0.006819741	0.002657346	0.010982137
9.57878E-05	-0.009839767	-0.013860216	-0.005819318
0.048851864	0.00121002	6.88192E-06	0.002413158
0.005468627	0.002425203	0.000819356	0.00403105

0.005108106	0.003933934	0.001374309	0.006493559
0.010210127	0.010093639	0.002729484	0.017457793
0.000236768	0.007102957	0.00387424	0.010331675
0.011780179	0.000498539	0.000130557	0.000866521
0.035512087	0.000693239	5.47614E-05	0.001331716
0.002636723	0.0007549	0.000314512	0.001195288
0.00220638	0.002267774	0.000976833	0.003558715
0.019157092	0.000603108	0.000111667	0.001094549
0.000772875	0.006955013	0.003362254	0.010547772
0.001341826	-0.006904765	-0.010684391	-0.003125139
0.010271882	0.004802279	0.001319571	0.008284987
0.000232615	0.004424682	0.002462668	0.006386696
0.00929414	0.005532078	0.00156935	0.009494806
0.039465172	0.002899215	0.000160048	0.005638383
0.001437471	-0.013940157	-0.021570663	-0.006309651
0.02027642	0.003022183	0.000551791	0.005492576
0.000122165	0.004331556	0.002512455	0.006150656
0.001647582	-0.00824774	-0.012824358	-0.003671122
0.002081021	-0.008060291	-0.012624725	-0.003495857
0.023852895	0.001678933	0.00025124	0.003106626
0.009015739	-0.008286617	-0.014140819	-0.002432415
0.000203891	0.013200319	0.007601203	0.018799435
0.000245124	-0.006860805	-0.009902796	-0.003818815
0.001501629	0.00951479	0.004237167	0.014792413
0.001191756	0.00990424	0.004613685	0.015194795
0.004797765	0.005809457	0.002067511	0.009551402
0.008092408	0.005801347	0.001719055	0.009883639
0.042840761	0.002499395	9.04755E-05	0.004908315
0.031994709	-0.006620387	-0.012531082	-0.000709693
0.003579144	-0.008832712	-0.014330344	-0.00333508
0.005640171	0.00046611	0.000161654	0.000770566
0.039082026	0.005453358	0.000338216	0.010568499
0.001373682	0.000501796	0.000233762	0.00076983
0.001927424	-0.01103579	-0.017004651	-0.005066928

Table S7. Co-occurrence network characteristics

Analysis indicators	Pre-treatment	On-treatment	Post-treatment	BAP	HTG-AP	MAP	SAP
The size of the graph	173	155	162	364	751	416	750
Order (number of vertices) of a graph	101	103	107	160	176	170	172
Connectance	0.034	0.03	0.029	0.029	0.049	0.029	0.051
Average degree	1.966	1.761	1.841	4.136	8.534	4.727	8.523
Average path length	3.286	4.039	3.762	3.904	2.816	3.557	2.855
Diameter	8	9	9	10	6	10	6
Average clustering coefficient	0.225	0.195	0.162	0.130	0.122	0.078	0.14
Eigenvector Centrality	0.003	0.009	0.007	0.007	0.018	0.010	0.010

Table S8. Functions of species co-occurrence networks of different etiologies and severities

KEGG pathway	Description	# of genes from speices in the MAP network	# of genes from speices in the MAP network
1501	beta-Lactam resistance	0	39
1502	Vancomycin resistance	0	16
1503	Cationic antimicrobial peptide	0	41
2020	Two-component system	0	263
2024	Quorum sensing	0	113
2025	Biofilm formation - Pseudomonas aeruginosa	0	53
2026	Biofilm formation - Escherichia coli	0	50
2030	Bacterial chemotaxis	0	24
2040	Flagellar assembly	0	45
2060	Phosphotransferase system	0	58
3070	Bacterial secretion system	0	45
4112	Cell cycle - Caulobacter	0	12
4146	Peroxisome	0	10
5111	Biofilm formation - Vibrio cholerae	0	32
5171	Coronavirus disease - COVID-19	0	22
5208	Chemical carcinogenesis - reactive oxygen species	0	10
